# Illuminating the biosynthesis pathway genes involved in bioactive specific monoterpene glycosides in *Paeonia veitchii* Lynch by a combination of sequencing platforms

**DOI:** 10.1186/s12864-023-09138-2

**Published:** 2023-01-26

**Authors:** Shaoshan Zhang, Jun-zhang Qu-Bie, Ming-kang Feng, A-xiang Qu-Bie, Yanfei Huang, Zhi-feng Zhang, Xin-jia Yan, Yuan Liu

**Affiliations:** 1 Tibetan Plateau Ethnic Medicinal Resources Protection and Utilization Key Laboratory of National Ethnic Affairs Commission of the People’s Republic of China, Chengdu, 610225 China; 2 Sichuan Provincial Qiang-Yi Medicinal Resources Protection and Utilization Technology and Engineering Laboratory, Chengdu, 610225 China; 3 College of Pharmacy, Southwest Minzu University, Chengdu, 610041 China

**Keywords:** *Paeonia veitchii* Lynch, Next-generation sequencing, Single-molecule real-time sequencing, Monoterpene glycoside, Terpene synthase, Cytochrome P450, UDP-glycosyltransferase, BAHD acyltransferase

## Abstract

**Background:**

*Paeonia veitchii* Lynch, a well-known herb from the Qinghai-Tibet Plateau south of the Himalayas, can synthesize specific monoterpene glycosides (PMGs) with multiple pharmacological activities, and its rhizome has become an indispensable ingredient in many clinical drugs. However, little is known about the molecular background of *P. veitchii*, especially the genes involved in the biosynthetic pathway of PMGs.

**Results:**

A corrective full-length transcriptome with 30,827 unigenes was generated by combining next-generation sequencing (NGS) and single-molecule real-time sequencing (SMRT) of six tissues (leaf, stem, petal, ovary, phloem and xylem). The enzymes terpene synthase (TPS), cytochrome P450 (CYP), UDP-glycosyltransferase (UGT), and BAHD acyltransferase, which participate in the biosynthesis of PMGs, were systematically characterized, and their functions related to PMG biosynthesis were analysed. With further insight into TPSs, CYPs, UGTs and BAHDs involved in PMG biosynthesis, the weighted gene coexpression network analysis (WGCNA) method was used to identify the relationships between these genes and PMGs. Finally, 8 TPSs, 22 CYPs, 7 UGTs, and 2 BAHD genes were obtained, and these putative genes were very likely to be involved in the biosynthesis of PMGs. In addition, the expression patterns of the putative genes and the accumulation of PMGs in tissues suggested that all tissues are capable of biosynthesizing PMGs and that aerial plant parts could also be used to extract PMGs.

**Conclusion:**

We generated a large-scale transcriptome database across the major tissues in *P. veitchii*, providing valuable support for further research investigating *P. veitchii* and understanding the genetic information of plants from the Qinghai-Tibet Plateau. TPSs, CYPs, UGTs and BAHDs further contribute to a better understanding of the biology and complexity of PMGs in *P. veitchii*. Our study will help reveal the mechanisms underlying the biosynthesis pathway of these specific monoterpene glycosides and aid in the comprehensive utilization of this multifunctional plant.

**Supplementary Information:**

The online version contains supplementary material available at 10.1186/s12864-023-09138-2.

## Introduction

*Paeonia veitchii* of the Paeoniaceae family is a perennial pharmaceutical and ornamental flowering herb inhabiting specific ecological niches of mostly inaccessible locales of the Qinghai-Tibet Plateau in the southern Himalayas. Because of its beautiful flower, *P. veitchii* is considered a dazzling star in China. The dried rhizomes of *P. veitchii*, named *Chishao*, are widely used in traditional Chinese medicine, and they have analgesic effects, regulate menstruation, tonify the blood and act as anti-inflammatory agents [1–3].

Previous phytochemical studies of *Paeonia* revealed that the active compounds in its roots are primarily monoterpene derivatives with a “cage-like” skeleton, including paeoniflorin, albiflorin, oxypaeoniflorin, benzoylpaeoniflorin, oxybenzoylpaeoniflorin, lactiflorin, galloylpaeoniflorin, and paeonin [4–6]. Most of these monoterpene derivatives are monoterpene glycosides (PMGs), especially paeoniflorin [7–9]. As the most abundant component, paeoniflorin has diverse biological functions, including anti-inflammatory, antioxidant, antithrombotic, anticonvulsive, analgesic, cardioprotective, neuroprotective, hepatoprotective, antidepressant-like, antitumoral, and immunoregulatory activities, as well as enhancing cognition and attenuating learning impairment [10–13]. In terms of chemical structures, we speculate that many PMGs originate from paeoniflorin, such as benzoylpaeoniflorin and galloylpaeoniflorin.

Unfortunately, the accumulation of PMGs in *Paeonia* requires approximately 5–6 years [14]. Given their complex molecular structures, PMGs are difficult to chemically synthesize for commercial use. Therefore, metabolic engineering may be an effective method for obtaining increased yields of these components. However, this strategy largely relies on the characterization of their biosynthetic pathway, which still has not been completely elucidated. With the discovery of new PMGs and advances in pharmacological research on PMGs, elucidating the biosynthetic pathways and regulatory mechanisms of active PMGs has attracted the attention of scientists.

**Fig. 1:**
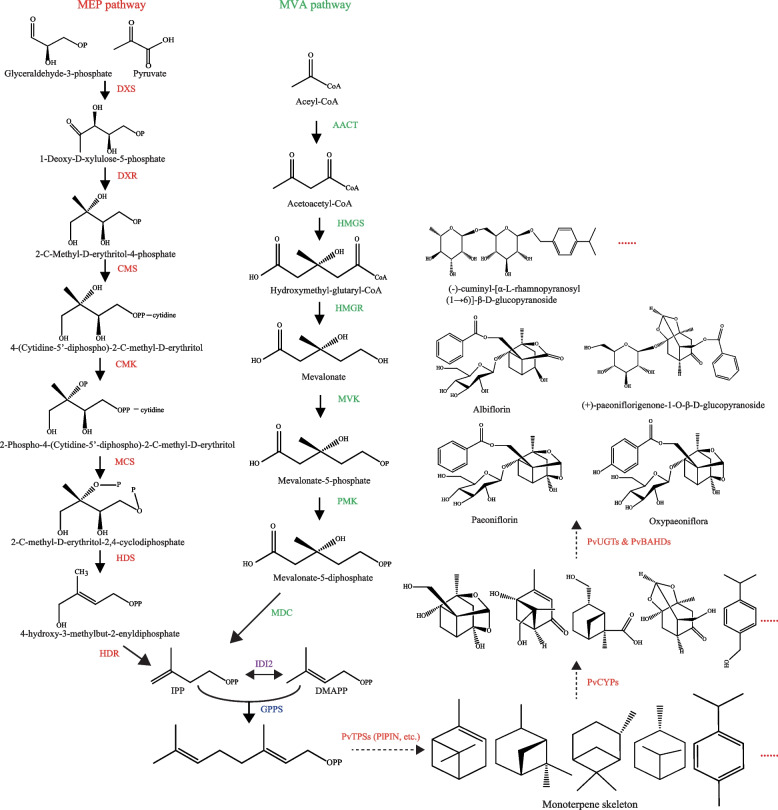
Possible biosynthetic pathways of monoterpene glycosides in *P. veitchii*. The established metabolic pathways are represented by solid line arrows, while the speculated metabolic pathways are represented by dotted line arrows

The biosynthesis of PMGs begins with terpene pathways, including the 1-deoxy-D-xylulose-5-phosphate/methyl-erythritol-4-phosphate (DXP/MEP) and mevalonate (MVA) pathways [15, 16]. Both pathways result in the biosynthesis of one molecule of isopentenyl diphosphate (IPP) and one molecule of dimethylallyl diphosphate (DMAPP), which is then catalysed by geranyl diphosphate synthase (GPPS) to form geranyl diphosphate (GPP, C10) [17, 18]. In plants, terpene synthases (TPSs) are responsible for the biosynthesis of numerous terpene skeletons from polyisoprene diphosphate precursors, leading to isoprene (C5), monoterpenes (C10), sesquiterpenes (C15), and diterpenes (C20) [19]. An early report used transcriptome data of *Paeonia lactiflora* (another well-known pharmaceutical and ornamental flowering plant of *Paeonia*) to successfully identify one pinene synthase (PlPIN, KU187411) involved in the conversion of GPP to α-pinene, which is one of the skeletons of PMGs [14]. In the biosynthesis of terpenes in plants, scaffolds catalysed by enzymes of the TPS family are then oxidized through the action of cytochrome P450s (CYPs). Modifications introduced by CYPs significantly increase the structural diversity of terpenoids and provide anchoring points for further linkage of sugar residues, alkylations, or esterifications by other transferases [20, 21]. UGTs can transfer sugar moieties from activated donor molecules to acceptor aglycones [22]. The enzymes involved in the acylation reaction of plant secondary metabolites belong to large acyltransferase families. Among these acyltransferases, the BAHD acyltransferase family can utilize acyl-activated coenzyme A thioesters and catalyse the formation of diverse acyl ester derivatives, such as benzoyl, acetyl, and hydroxycinnamoyl [23, 24]. Based on the chemical structure of PMGs, it can be speculated that their biosynthesis should involve enzymes belonging to UGTs, BAHDs and CYPs families (Fig. 1). Although a number of studies have reported functional genes in *Paeonia*, they have mainly concentrated on codon usage patterns [25], thermotolerance-related differentially expressed genes [26], fatty acids [27] and anthocyanin biosynthetic genes [28] based on transcriptome sequencing. However, the genes involved in glycosylation, benzoylation, oxidation and reduction of PMGs are unknown.

To date, the methods for mining genes associated with the biosynthesis of natural products mainly depend on the following three strategies: (1) an enzyme activity-guided approach, such as the identification of four BAHD acyltransferases (EpHTT, EpHQT, EpHCT, EpCAS) that complete chicoric acid biosynthesis in purple coneflower [29]; (2) a molecular probe-guided approach, such as discovering two UGTs (SrUGT76G3 & SrUGT73E1) involved in the steviol glycoside biosynthesis pathway from *Stevia rebuadiana* by the development of a photoaffinity probe [30]; and (3) omics analysis, such as using transcriptome mining in *Podophyllum hexandrum* to identify six tissue-specific expression genes (*CYP71CU1*, *OMT1,2-ODD*, *CYP71BE54*, *CYP82D61*, *OMT3*) that complete the biosynthetic pathway of podophyllotoxin [31].

With the rapid development of high-throughput sequencing technology and large-scale data analysis methods, omics analysis has become the most popular method. Large amounts of genomic and transcriptional data from medicinal plants have been submitted to and generated from public databases, but the progress of candidate gene characterization in specialized plant metabolites remains slow due to the following factors. The divergent evolution of some superfamilies (CYP, UGT, etc.) generated massive numbers of closely related individual members that can modify diverse types of natural metabolites [32, 33]. Compared to microorganisms, the genes involved in plant metabolite biosynthesis are generally scattered throughout the plant genome, further increasing the difficulty of accurately identifying candidate genes through the physical distance of metabolic pathway-related genes [32, 34]. Moreover, the PMG biosynthesis pathway requires the participation of genes of superfamilies such as *CYPs* and *UGTs*, making it even more difficult to resolve the genes in its biosynthesis pathway.

Due to their growth in inaccessible places in the Himalayas, no research has examined *P. veitchii* at the molecular level*.* The lack of molecular background information about *P. veitchii* is a huge knowledge gap for scientists trying to understand plateau plants, especially in the *Paeonia* genus. Therefore, providing the available full-length sequence for each RNA, especially those corrected by NGS reads, is vital for researchers to study *P. veitchii* at the molecular level and improve knowledge of the existing PMG biosynthesis pathways. In the current study, SMRT long-read sequencing technology and NGS short-read sequencing technology were combined to sequence six different tissues from *P. veitchii*. WGCNA combining the contents of PMGs, quantitative real-time polymerase chain reaction (qRT–PCR), and phylogenetic analysis was further used to predict the putative genes involved in the biosynthesis of PMGs, especially *TPSs*, *UGTs*, *CYPs*, and *BAHDs*.

## Results

### Content of the main PMGs in tissues

**Fig. 2:**
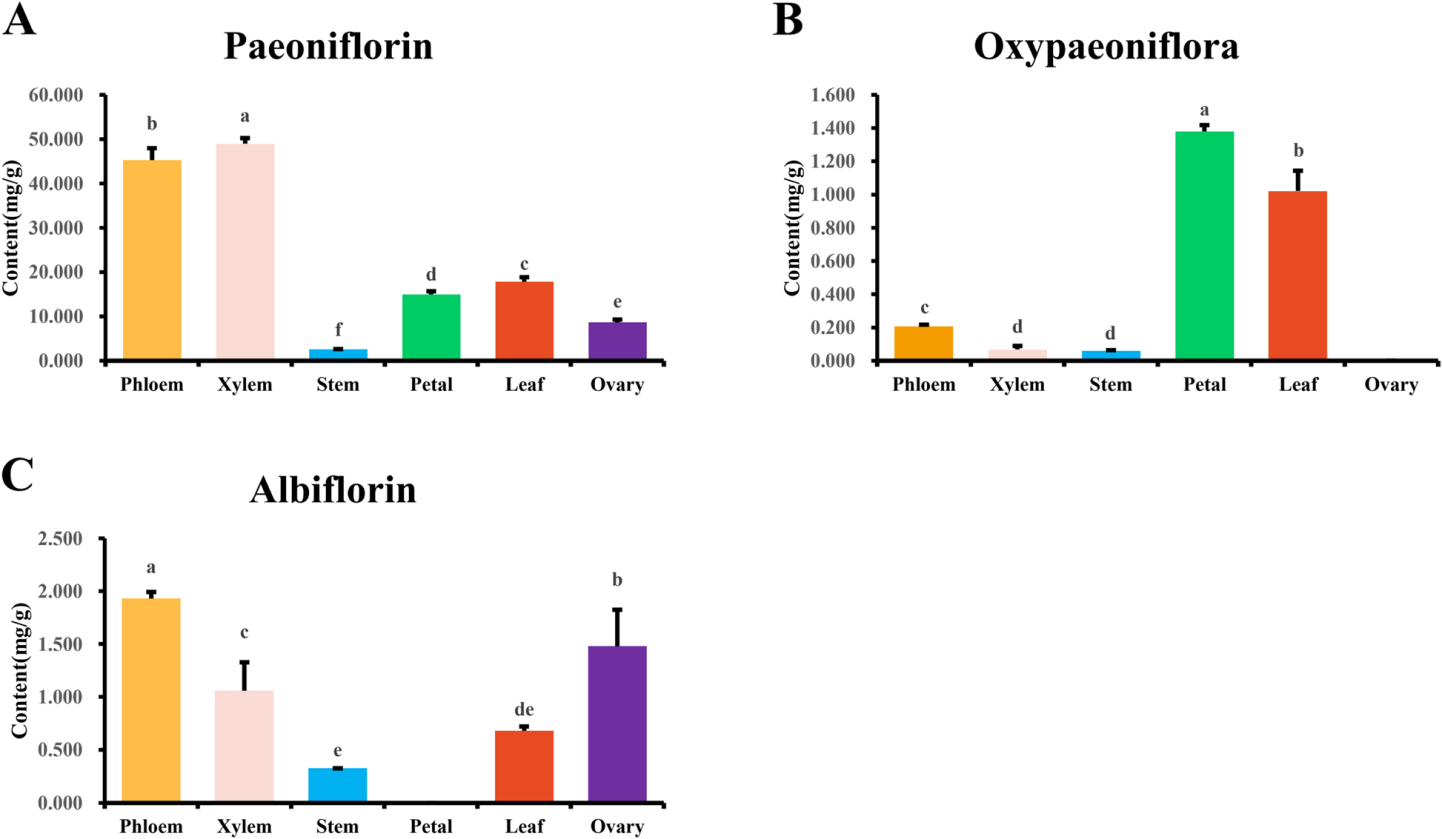
Contents of three PMGs (oxypaeoniflora, albiflorin, and paeoniflorin) in different tissues of *P. veitchii*. The contents of PMGs were quantified in three separate experiments. The error line represents the standard deviation. The significance level was detected at *α* = 0.05

Among the numerous PMGs, the concentrations of paeoniflorin were higher than those of other monoterpene glycosides [7–9]. In addition, the standards of oxypaeoniflora and albiflorin (also isolated from *Paeonia*) were easily obtained. Therefore, analysis of the three glycosides in all samples was performed using the HPLC-UV method. All validation projects met the quantitative requirements (Table S1). Then, the production profiles of the 3 metabolites in different tissues (leaves, stems, petals, ovaries, phloem and xylem) of *P. veitchii* were determined (Fig. 2). Paeoniflorin accumulated in all tested tissues, especially in the xylem and phloem (accounting for more than 45.0 mg/g), while the accumulation levels in stem was only 2.6 mg/g. Albiflorin was hardly detected in petal (0.0 mg/g) and highly accumulated in phloem (1.9 mg/g), xylem (1.1 mg/g), and ovary (1.5 mg/g). The distribution of oxypaeoniflora was undetectable in the ovary (0.0 mg/g) and significantly higher in the phloem (1.4 mg/g) and leaf (1.0 mg/g) than in the other tissues. The analysis suggested that the content of the analyzed PMGs displays variation in different tissues. This variation of content should be useful in predicting the genes involved in the biosynthesis of PMGs.

### Transcriptome sequencing and functional annotation

First, leaves, stems, petals, ovaries, phloem and xylem with different concentrations of the PMGs of *P. veitchii* were used to generate short-read libraries using an Illumina HiSeq X Ten platform, and each sample was sequenced with three biological replicates. After filtering out the adapter sequences and low-quality and contaminated reads, a total of 128.93 Gb clean reads (from 134.55 Gb raw reads) for 18 sequencing libraries, including three biological replicates of leaf, stem, petal, ovary, phloem and xylem, were generated (Table S2). After *de novo* assembly, a set of 92,776 unigenes was obtained, with a total length, average length, N50, and GC content of 100,517,455 bp, 1,083 bp, 1,475 bp and 44.64%, respectively. According to the statistics, 55,041 (59.3%) unigenes were ≤ 500 bp in length (Fig. S1).

Due to the technical limitations of *de novo* transcriptome assembly, the unigenes from Illumina sequencing are often fragmented or misassembled [35]. Therefore, the SMRT sequencing platform, which is considered the most reliable means for accessing full-length cDNA molecules [36], was utilized to generate full-length complementary cDNA reads for each RNA extracted from *P. veitchii*. Finally, a total of 18,603,493 raw reads (approximately 72.2 billion bases) were obtained from the Pacific Biosciences Sequel II platform. After performing the IsoSeq protocols [37], 39,347 high-quality consensus sequences were obtained. Furthermore, Illumina short reads were used to correct the low-quality consensus sequences. Finally, 30,827 full-length transcript sequences with an average length of 2,346 bp were obtained by removing redundant sequences for the high-quality consensus sequences and the corrected low-quality consensus sequences. In addition, 28,468 (92.3%) unigenes were > 1,000 bp, but only 2,359 (7.6%) unigenes were ≤ 1,000 bp in length (Fig. S2). Comparing the unigenes between the Illumina and PacBio Sequel platforms, it was obvious that the unigenes generated with the PacBio Sequel platform more accurately represented the real cDNAs in *P. veitchii*. Thus, the unigenes obtained by SMRT sequencing were selected as the reference transcriptome for subsequent analyses. In addition, we evaluated the integrity of the Illumina and PacBio transcriptomes using BUSCO [38], and the results showed that complete and single-copy duplicated transcript sequences accounted for 82.6% and 57.3%, respectively.

After annotation in the seven public databases, 28,441 (92.26%), 24,602 (79.81%), 4,850 (15.73%), 18,826 (61.07%), 28,027 (90.92%), 22,356 (72.52%), and 26,288 (85.28%) unigenes from SMRT sequencing were annotated in NR, Swiss-Prot, KEGG, KOG, eggNOG, GO, and Pfam, respectively (Table S3). Most of the unigenes were successfully annotated in at least one of the seven databases. In GO analysis, unigenes annotated in “metabolic process” and “catalytic activity” accounted for relatively higher proportions than unigenes belonging to other GO subcategories (Fig. S3). In KEGG pathway annotation, a large group of unigenes (177) were annotated as “metabolism of terpenoids and polyketides”, which was consistent with the richness of terpenoids in *P. veitchii* (Fig. S4) [39–41].

### Identification and functional analysis of the TPS gene family

**Fig. 3:**
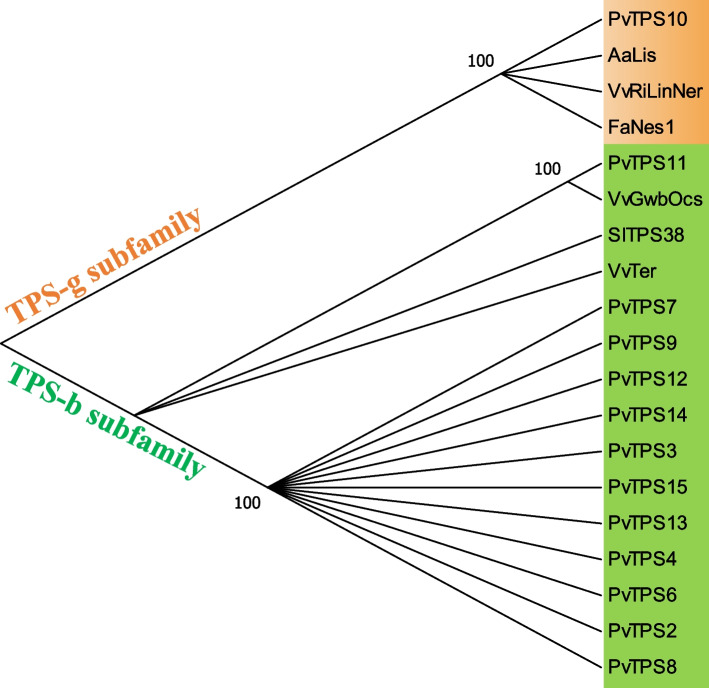
Phylogenetic tree illustrating most of the PvTPSs as the members of TPS-b subfamily. Grouping into subfamilies is based on previous study [19, 42]. Sequence abbreviations: AaLis, linalool synthase (ADD81294); FaNes1, nerolidol synthase1 (CAD57083); VvGwbOcs, Gewürztraminer (E)-beta-ocimene synthase (ADR74204); VvRiLinNer, Riesling linalool/nerolidol synthase (JQ062931); VvTer, (−)-α-terpineol synthase (AAS79352); SITPS38, a-Bergamotene synthase (AEP82768.1). The full-length amino acid sequence of PvTPSs described in additional file 3. The tree was derived by neighbour-joining distance analysis based on a ClustalW protein sequence alignment in MEGA7. Quality of the tree was analyzed by bootstrap analyses consisted of 1000 replicates

Based on the 30,827 full-length transcript sequences from PacBio sequencing, 18 nonredundant *TPSs* were identified using Pfam annotation and the BLAST algorithm. We designated these 18 gene models as *TPS1* through *TPS18*, among which *TPS1*, *TPS5*, *TPS16*, *TPS17*, and *TPS18* appeared to have fragmented open reading frames (ORFs) because they encode for proteins shorter than 450 amino acid residues or do not contain the highly conserved aspartate-rich motif ‘DDxxD’ in their C-terminal domain. Generally, *TPS* genes in plants are divided into seven clades, with some plant lineages having a majority of their TPS genes in one or two clades [19]. The phylogenetic tree of TPSs with complete ORFs from *P. veitchii* and other species indicated that these PvTPSs were classified into the TPS-b and TPS-g subfamilies (Fig. 3). However, most of the PvTPSs were grouped into the TPS-b subfamily, which contains the RR(X_8_) W motif in the N-terminal domain, a key feature of the vast majority of monoterpene synthases [20, 42–44]. According to the results of the quantitative analysis of the main PMGs in *P. veitchii*, monoterpene derivatives account for a large proportion of metabolites in *P. veitchii*. Therefore, we speculated that the genes of the TPS-b subfamily are involved in the formation of monoterpene skeletons in *P. veitchii*.

### Identification and functional analysis of the CYP gene family

**Fig. 4:**
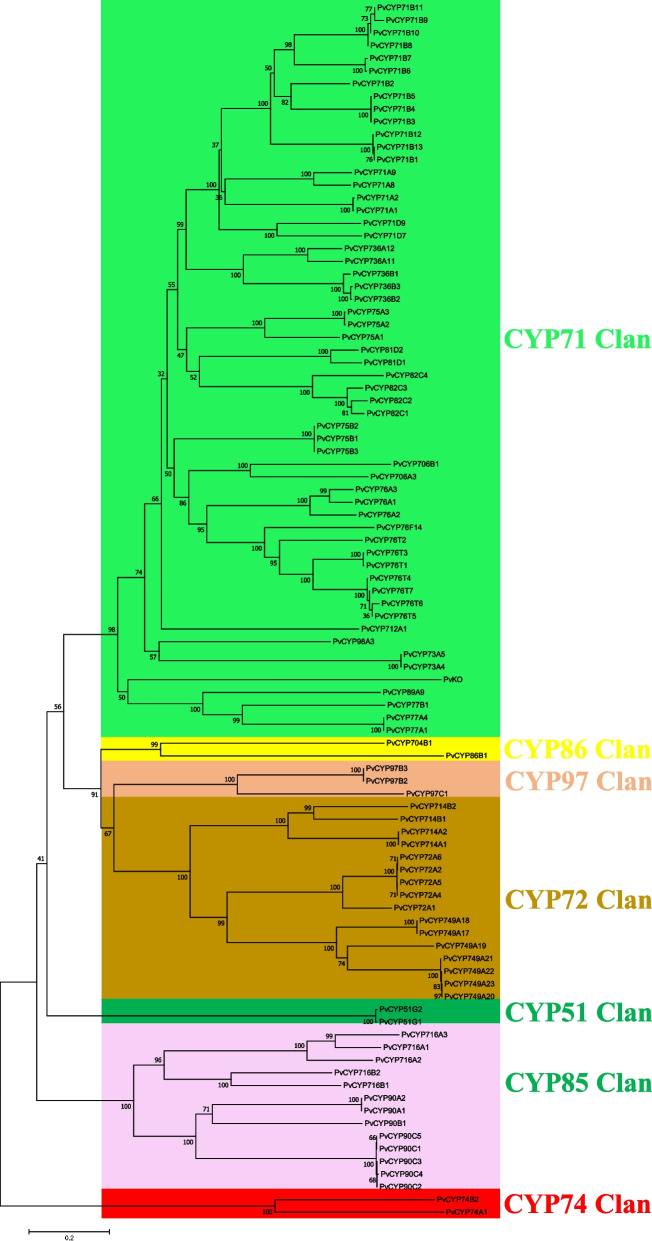
Phylogenetic tree illustrating the PvCYPs were separated into different clans. The full-length amino acid sequence of PvCYPs described in additional file 4. Clan separation referenced to the data in website: http://www.p450.kvl.dk/cyp_allsubfam_NJ_102103.pdf. Phylogenetic relationship was derived by neighbour-joining distance analysis based on a ClustalW protein sequence alignment in MEGA7. Quality of the tree was analyzed by bootstrap analyses consisted of 1000 replicates

In this study, 126 CYPs were characterized from the full-length transcript sequences from PacBio sequencing. According to the cross-kingdom nomenclature system of CYPs [45, 46] with reference to the classification of CYPs in *Arabidopsis,* these CYPs were assigned to 23 families with the numbers 51 (3), 71 (32), 72 (10), 73 (2), 74 (2), 75 (6), 76 (12), 77 (3), 78 (1), 81 (3), 82 (5), 86 (1), 89 (1), 90 (8), 97 (4), 98 (1), 704 (1), 706 (2), 712 (1), 714 (5), 716 (5), 736 (5), and 749 (12), and one annotated as ent-kaurene oxidase (PvKO). The numbers in brackets are the number of genes in each family. Further analysis showed that the amino sequences of 30 CYPs lacked the heme-binding motif (FxxGxxxCxG) and electron-transfer channel motif (PERF), which are well-conserved motifs in P450s [47]. To predict the enzyme function of this family, the nonredundant CYPs with complete ORFs were further separated into clans defined by the phylogenetic tree (Fig. 4). All functionally characterized plant P450s with activity as monoterpene oxidases belong to the CYP71 clan [46, 48]. In our phylogenetic tree, a total of 58 different PvCYPs belonged to the CYP71 clan. Previous studies have found that among CYP71clan, the vast majority of functionally characterized members identified as involved in monoterpene metabolism in plants belong to the CYP71 and CYP76 subfamilies [46, 49, 50], which made these members interesting candidates for a possible role in PMG biosynthesis.

### Identification and functional analysis of the UGT gene family

**Fig. 5:**
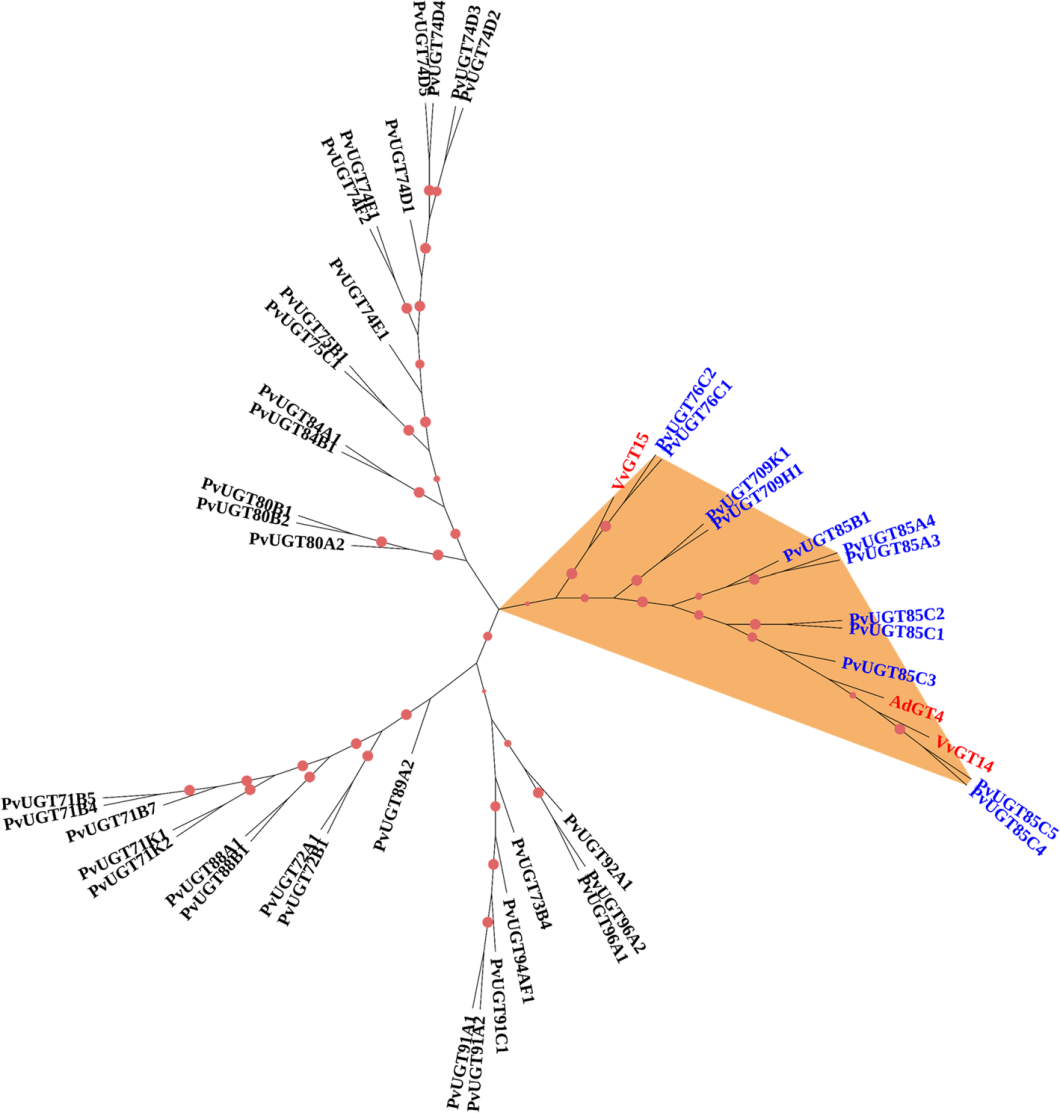
Phylogenetic tree of functionally characterized UGT members plus the PvUGT proteins. Glucosyltransferases from *Actinidia deliciosa* (AdGT4, KF954944), *Vitis vinifera* (VvGT14, XM_002285734.2), and *Vitis vinifera* (VvGT15, XM_002281477.2) with known function toward monoterpenol are also shown. The tree was derived by neighbour-joining distance analysis of the full-length amino acid sequence described in additional file 5. Distance bootstrap analyses consisted of 1000 replicates, where values over 50% are indicated above the nodes using symbol

In this work, 53 UGTs were characterized from the full-length transcript sequences from PacBio sequencing. According to the GT nomenclature committee [51] and according to the classification of UGTs in *Arabidopsis,* these UGTs were classified into 16 subfamilies with the numbers 71 (7), 72 (2), 73 (1), 74 (10), 75 (2), 76 (2), 80 (4), 84 (3), 85 (8), 88 (2), 89 (1), 91 (3), 92 (1), 94 (2), 96 (2), and 709 (3). Among these UGTs, the amino sequence of 8 UGTs was found to be without the PSPG box (plant secondary product glycosyltransferase), which contains a conserved 44 amino acid motif (WAP... DQ) [52]. In addition, a fragmented ORF was found in 9 UGTs. To date, monoterpenoid UGT genes have rarely been reported, whereas diterpenoid and triterpenoid-specific UGTs have been well described. Recently, VvGT14 and VvGT15 from *Vitis vinifera* and AdGT4 from *Actinidia delicosa* were reported to be monoterpenol-specific UGTs [53, 54]. Hence, these three monoterpenol-specific UGTs were aligned with the forty-five nonredundant UGTs with complete ORFs, and then a composite phylogenetic tree was constructed (Fig. 5). Interestingly, AdGT4 and VvGT14 were clustered in the UGT85 and UGT709 subfamilies with a very high bootstrap value (over 70%). VvGT15 was clustered in the UGT76 subfamily and had a very high bootstrap value (100%). These results implied that the genes in the UGT85, UGT709, and UGT76 subfamilies may be involved in the glucosylation of monoterpenes in *P. veitchii*. This work prompted us to further characterize *UGT* genes associated with the formation of glycosylated PMGs.

### Identification and functional analysis of the BAHD acyltransferase family

According to the annotation information and the two well-conserved motifs in BAHD (HXXXD & DFGWG) [55], 14 *BAHD* genes in *P. veitchii* from the transcriptome database of PacBio sequencing were identified. We designated these 14 gene models *PvBAHD1* through *PvBAHD14*. Previous studies have shown that BAHD members are all monomeric enzymes with molecular masses ranging from 48 to 55 kDa, and the average number of amino acids is approximately 445 [23]. Among the 14 identified PvBAHDs, the molecular mass and number of amino acids in PvBAHD13 are 31 kD and 275, respectively. Hence, the PvBAHD13 gene may be a pseudogene.

**Fig. 6:**
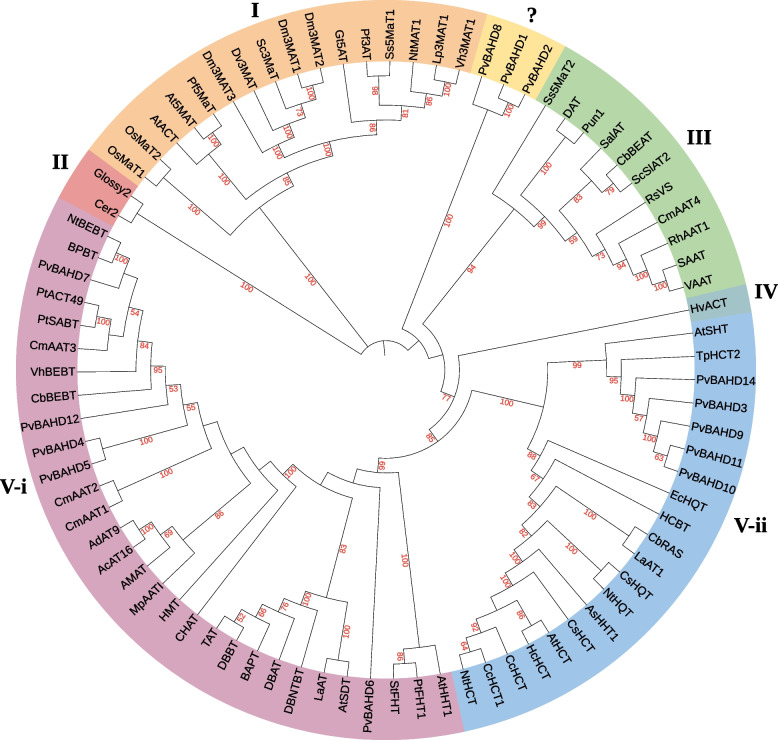
Phylogenetic tree of functionally characterized BAHD members plus the 13 PvBAHD1 proteins. The five major clades (I-V) referenced to the work by D’Auria (2006) [55]. The following proteins and their corresponding GenBank Accession numbers were used: AcAT16 (HO772640), AdAT9 (HO772637), AMAT (AAW22989), AsHHT1 (BAC78633), At5MAT (Q9LJB4), AtACT (Q9FNP9), AtHCT (NP_199704), AtHHT1 (ACY78659), AtSDT (NP_179932), AtSHT (AEC06845), BAPT (AAL92459), BPBT (AAU06226), CbBEAT (AAF04787), CbBEBT (AAN09796), CbRAS (CAK55166), CcHCT (DQ104740), CcHCT1 (EF137954), Cer2 (AAB17946), CHAT (AAN09797), CmAAT1 (CAA94432), CmAAT2 (AAL77060), CmAAT3 (AAW51125), CmAAT4 (AAW51126), CsHCT (AEJ88365), CsHQT (DQ915589), DAT (AAC99311), DBAT (AAF27621), DBBT (Q9FPW3), DBNTBT (AAM75818), Dm3MAT1 (AAQ63615), Dm3MAT2 (AAQ63616), Dm3MAT3 (BAF50706), Dv3MAT (Q8GSN8), EcHQT (AFF19202), Glossy2 (DAA36076), Gt5AT (Q9ZWR8), HCBT (CAB06430), HcHCT (JQ779021), HMT (BAD89275), HvACT (AAO730710), LaAT (AB581532), LaAT1 (DQ886904), Lp3MAT1 (AAS77404), MpAATI (AAU14879), NtBEBT (AAN09798), NtHCT (CAD478300), NtHQT (CAE46932), NtMAT1 (2XR7_A), OsMaT1 (BAD21952), OsMaT2 (NP001046855), Pf3AT (Q9MBC1), Pf5MaT (Q9LJB4), PtFHT1 (XP_002298644), Pun1 (ADN97116), RhAAT1 (AAW31948), SAAT (AAG13130), SalAT (Q94FT4), Sc3MaT (AAO38058), ScSlAT2 (AFM77971), Ss5MaT1 (Q8W1W9), Ss5MaT2 (AAR26385), StFHT (FJ825138), TAT (AAF34254), TpHCT2 (ACI16631), VAAT (AX025504), Vh3MAT1 (AAS77402), VhBEBT (BAE72881), RsVS (CAD89104). PtSABT (Potri.013G074500) and PtACT49 (Potri.019G043600) obtained from Phytozome v10 (http://www.phytozome.net/). The tree was derived by neighbour-joining distance analysis of the full-length amino acid sequence described in additional file 6. Distance bootstrap analyses consisted of 1000 replicates, where values over 50% are indicated above the nodes

In an attempt to identify the candidate *PvBAHD* genes involved in the benzoylation of PMGs, phylogenetic analysis using full-length amino acid sequences of 13 complete PvBAHDs with a number of functionally characterized BAHDs from multiple species was conducted to determine the relationships of the PvBAHDs with the five major BAHD clades previously described [23] (Fig. 6). The results showed that five proteins (PvBAHD4/5/6/7/12) cluster with BAHD clade V-i, which is mainly responsible for utilizing benzoyl-CoA as the major acyl donor to synthesize benzenoid ester production, including CbBEBT from *Clarkia breweri* [55], NtBEBT from *Nicotiana tabacum* [55], VhBEBT from *Verbena x hybrida* [56], BPBT from *Petunia x hybrida* [57], DBNTBT from *Taxus canadensis* [58], DBBT from *Taxus cuspidata* [59], and PtACT49 and PtSABT from *Populus trichocarpa* [60]. Therefore, these five putative genes may be involved in the benzoylation of PMGs. Interestingly, a new clade consisting of 3 BAHD family members (PvBAHD1/2/8) was generated and has high bootstrap support (100%). Considering the particularity of the growth environment of *P. veitchii*, the genes in this new clade may have special functions, such as helping plants adapt to the harsh environment or participating in the biosynthesis of other special compounds.

### Metabolite and gene coexpression analysis to further predict functional genes

**Fig. 7:**
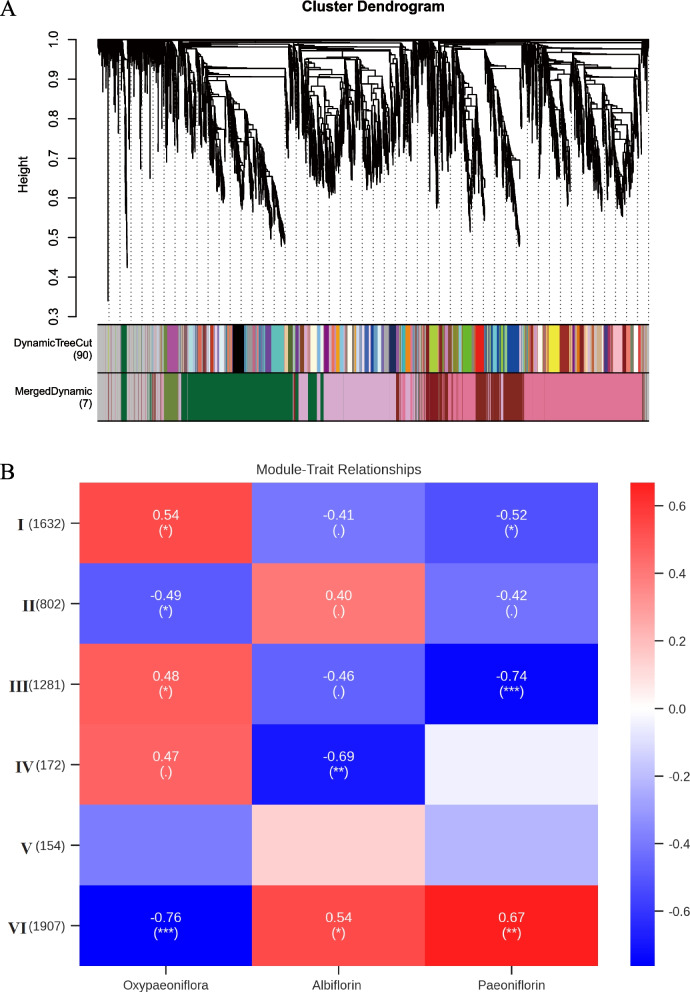
WGCNA analysis to predict that the key genes involved in the biosynthesis of PMGs. A: Clustering dendrograms of 6,560 transcripts (after filtering the low fluctuation expression (standard deviation < 1) transcripts). Each clade corresponding to a module was represented by different color. Modules with certain correlations were merged into the same module. B: Module-PMGs relationships. Each row represents a module of characteristic genes, numbers in bracket are the amount of genes in each module. Each column represents a metabolite. Each cell represents the correlation and the significance (*, **, and *** significant at 10%, 5%, and 1% level, respectively). The list of transcripts in each modules and the expression levels of transcripts in each tissues were provided in the additional file 7 and 8

WGCNA has been proven to be an effective analytical tool in systematically describing the correlation relationship between clusters of highly correlated genes or modules and external conditions or sample traits [61]. Consequently, this method was selected to further analyse the potential genes involved in the biosynthesis of PMGs. First, 30,827 transcripts from PacBio sequencing were mapped by the NGS clean reads in each tissue, and then the FPKM value of each transcript in tissues was obtained. Then, these 30,827 transcripts were used as raw input data for WGCNA. Based on the FPKM value, the genes with low fluctuation expression (standard deviation < 1) were filtered, and 6,560 genes remained. As shown in Fig. S5, a soft threshold β=30 was used to build the weighted coexpression network. Then, hierarchical clustering on dissimilar matrices was performed by the function hclust, and Dynamic Tree Cut was used to cut the generated cluster tree. Transcripts with a strong correlation were assigned to the same module with different colours. Finally, 6,560 transcripts were divided into 7 modules, and the number of transcripts in each module was 154-1907. Transcripts that failed to be assigned to any module were placed in the grey module, which has no reference significance. The hierarchical clustering dendrogram of gene networks is visualized in Fig. 7a.

Then, six generated modules were correlated with the traits (the content of the aforementioned three PMGs in each tissue) to identify potential modules that are significantly related to the PMG content. Based on the absolute value of the Pearson correlation coefficient > 0.3 and *p* value < 0.05, the modules associated with each PMG were obtained. The module-PMG relationships are colour-coded in Fig. 7b. The larger the absolute value of the Pearson correlation coefficient, the greater the correlation between the genes in the module and the PMG. Depending on the correlation coefficient of the module-PMG, we observed that oxypaeoniflora was positively correlated with the I, III, and IV modules. Albiflorin was positively correlated with the II and VI modules, whereas paeoniflorin clustered well with the VI module (Fig. 7b). The clustering results indicated that the genes in the I, II, III, IV, and VI modules are potentially involved in the biosynthesis of PMGs.

**Fig. 8:**
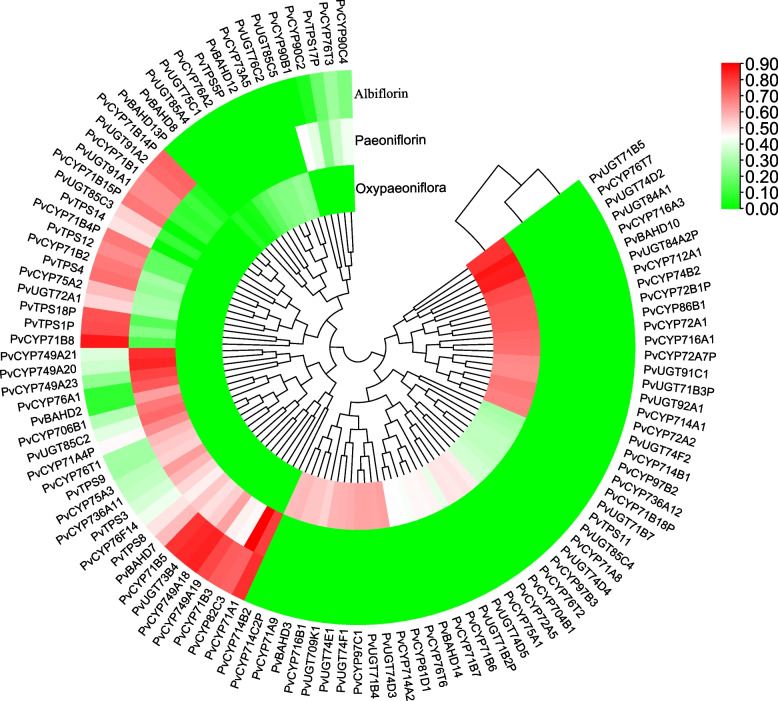
The correlation coefficient between genes in modules (I, II, III, IV, and VI) and metabolites

Based on the five predicted modules, 12 TPSs (including 4 pseudogenes), 80 CYPs (including 15 pseudogenes), 8 BAHDs (including 1 pseudogene), and 30 UGTs (including 3 pseudogenes) were obtained (Table S4). The Pearson correlation coefficients between these genes and the PMGs are shown in Fig. 8. Combined with the above analysis of each family: (1) TPSs in the TPS-b subfamily are involved in the formation of the monoterpene skeleton; (2) P450s that can be hydroxylated in the monoterpene skeleton often belong to the CYP71 and CYP76 subfamilies; (3) UGTs in the UGT85 and UGT76 subfamilies are likely to participate in the glucosylation of monoterpenes; and (4) BAHDs clustering with clade V-i may be involved in the benzoylation of metabolites. Genes that have fragmented ORFs were excluded. Finally, we narrowed the *TPSs*, *CYPs*, *UGTs*, and *BAHDs* to 8, 22, 7, and 2 candidate genes, respectively (Table S5). Notably, *PvTPS3*, one of the 8 *TPSs*, was the same as pinene synthase (PlPIN), which proved to be involved in the conversion of GPP to a-pinene, one of the skeletons of PMGs [14]. This further demonstrated the reliability of our analytical result.

**Fig. 9:**
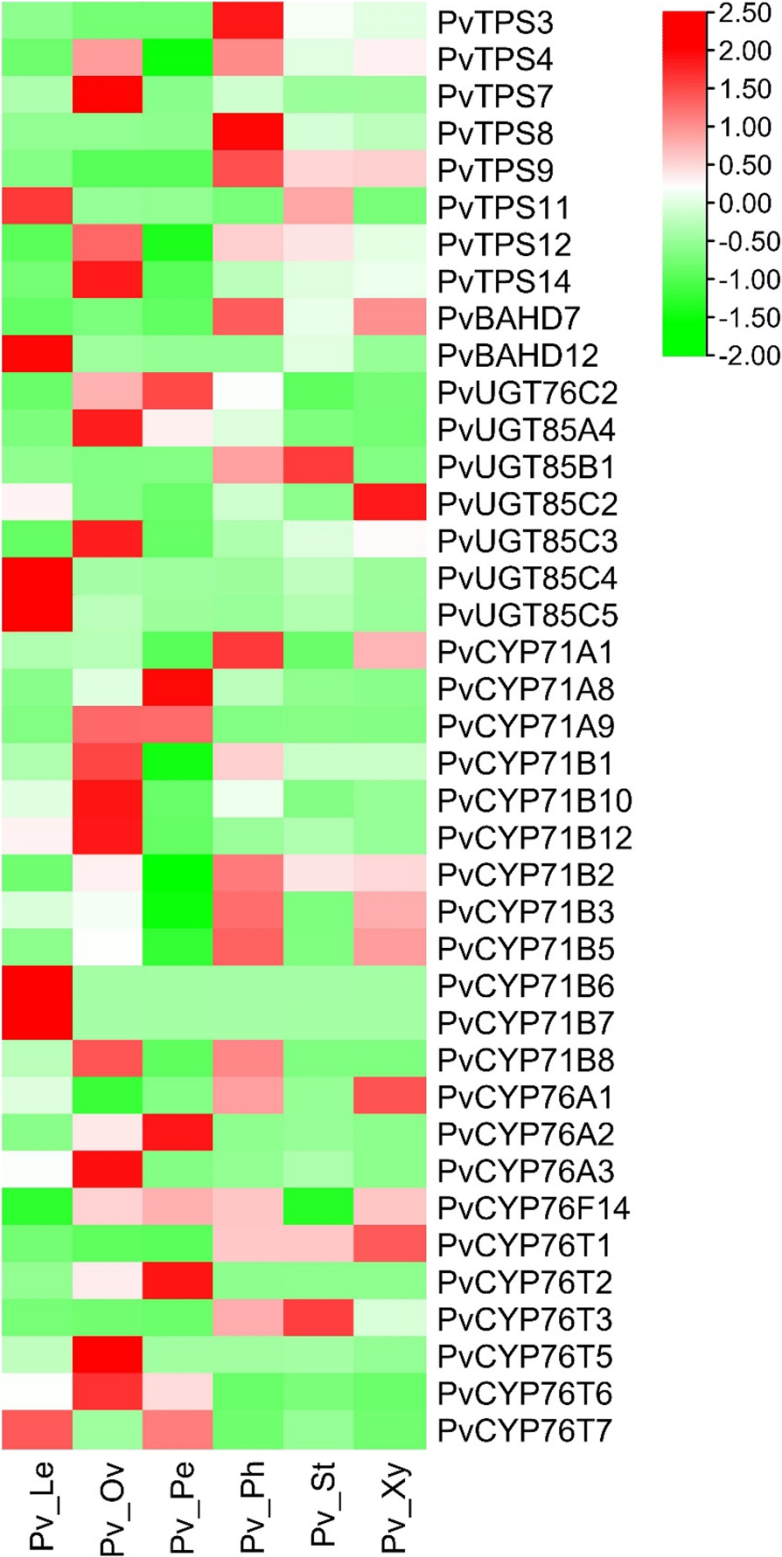
Heatmaps of expression levels of candidate genes (table S5) in different tissues of *P. veitchii*. All genes are arranged from top to bottom according to the total expression level

The expression levels of these putative genes in leaves, stems, petals, ovaries, phloem and xylem are shown in Fig. 9, and there are significant differences in their expression patterns in different tissues. It is reasonable to believe that these genes are among those most likely to participate in the biosynthesis of monoterpene glycosides, and we will systematically verify them in subsequent research work.

### Validation of gene expression profiles by qRT–PCR

**Fig. 10:**
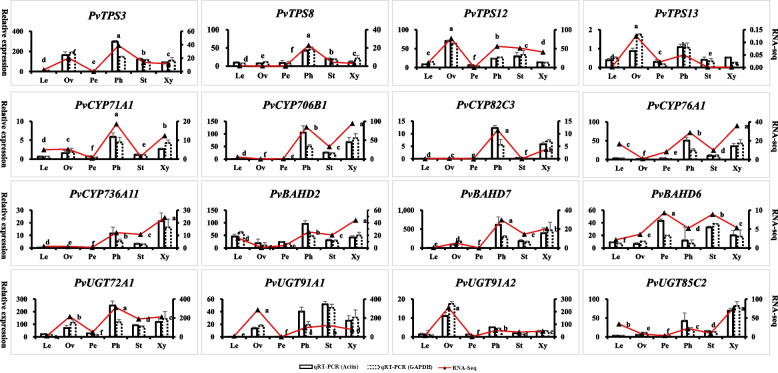
qRT-PCR validation of RNA sequencing data. Expression profiles of sixteen selected genes were determined by transcriptome and qRT-PCR data. The left vertical axis represents the relative expression based on qRT-PCR. The right vertical axis represents the expression level based on RNA sequencing. The letter denotes statistical significance based on the RNA-seq data (different letters denote *P* < 0.05). The Le, Ov, Pe, Ph, St, and Xy represented the leaf, ovary, petal, phloem, xylem, and stem, respectively

To confirm the reliability of the RNA sequencing results, sixteen genes from *PvTPSs*, *PvCYPs*, *PvBAHDs* and *PvUGTs* were selected for relative quantitative analysis. The primers used in qRT–PCR were listed in Table S6. Regardless of whether *actin* or *GAPDH* was used as a housekeeping gene, the relative expression of the sixteen selected genes showed that the tendency of these genes to be expressed was similar between the qRT–PCR and transcriptomic analyses, confirming that the transcriptomic results were reliable (Fig. 10). In addition, the qRT–PCR results of the sixteen genes were significantly different in different tissues, which is consistent with the results of the PMG content analysis.

## Discussion

To adapt to the local ecological niche, each plant species may synthesize a different set of secondary metabolites to help facilitate interactions with the local biotic and abiotic environment [18]. Among plant metabolites, terpenoids are the most chemically and structurally diverse family of natural products, encompassing over 80,000 individual compounds, most of which have specialized roles in plant-environment interactions [62–64]. Furthermore, a variety of molecules with pharmacological properties, both from medicinal plants and clinical drugs derived from natural products, are terpenoids.

The Qinghai-Tibet Plateau, which is located in the southern Himalayas, is the highest altitude plateau in the world and is called the “Roof of the World”. The environment in the Qinghai-Tibet Plateau has a special plateau mountain climate because of the altitude and terrain [65]. Therefore, terpenoids synthesized by plants in this region should be specialized metabolites that are not wholly shared by all plant lineages. The genetic information of plants in this region, especially the genes involved in the biosynthesis of specialized terpenoids, is also unique. *P. veitchii* is usually distributed at altitudes ranging from 3,000 to 4,000 metres and is a very representative medicinally perennial plant on the Qinghai-Tibet Plateau, but there are no reports about its transcriptome or other omics. In the present study, we used short-read NGS and long-read SMRT together to sequence *P. veitchii* and then obtained a high-quality full-length transcriptome (total of unigenes = 30,827, average length = 2,346 bp) and a large-scale NGS assembled transcriptome (total of unigenes = 92,776, average length = 1,083 bp). To the best of our knowledge, this report is the first public study to characterize the structure of transcripts in *P. veitchii*. These transcriptome data will provide strong support for the further study of plants in the Himalayas, especially on the Qinghai-Tibet Plateau.

Studying the biosynthetic pathway of metabolites originating from medicinal plants is still restricted owing to the complexity of their genomes and the absence of genomic information. As described in the introduction, functional genes involved in the biosynthesis of metabolites in plants are usually diffusely distributed in the chromosomes, and gene redundancy and strict genetic regulation in plants lead to difficulty in resolving metabolic pathways [32, 34, 66]. At present, the traditional methods for analysing the biological pathways of metabolites are limited and often yield a large number of false-positive results, especially for large datasets from heterogeneous sources [32, 61].

Chromatography analysis (HPLC, LC–MS/MS, etc.) of *P. veitchii* indicated that the majority of its constituents were classified as monoterpenoids, which have many biological activities [67–69]. *P. lactiflora*, another *Paeonia* plant, contains monoterpenoids similar to those of *P. veitchii*. Several studies have reported the transcriptome data of *P. lactiflora,* and some of them were focused on the biosynthesis of monoterpenoids [7, 26, 70, 71]. Due to the existence of hybridization between the cultivars in different regions, the genetic background of *P. lactiflora* was mixed, and all of these transcriptome measurements of *P. lactiflora* were dependent on the Illumina platform. Therefore, these studies hardly reflected the complete transcriptome information involved in the biosynthesis of terpenoids. Moreover, studies on the biosynthesis of monoterpenoids have mainly focused on upstream genes in terpene pathways, such as genes in the MEP and MVA pathways [7].

SMRT long-read sequencing technology was proven to be the most reliable means of sequencing full-length cDNA molecules and was widely used to offer access to more complete transcriptome data due to its long reads (average 4-8 kb), higher throughput, faster detection speed and fewer systematic errors caused by in vitro reverse transcription [36]. In this study, the genes *TPS*, *CYP*, *UGT*, and *BAHD* were systematically characterized through full-length transcriptome sequencing, and their functions related to PMG biosynthesis were successfully analysed. With further insight into *TPSs*, *CYPs*, *UGTs* and *BAHDs* involved in PMG biosynthesis, we constructed a coexpression network of metabolites with gene expression levels in different tissues to further determine the relationships between these genes and PMGs. Finally, 8 TPSs, 22 CYPs, 7 UGTs, and 2 BAHD putative genes were found to be involved in the biosynthesis of PMGs. Compared with previous reports, our study could provide useful information for the accurate prediction of the biosynthetic pathway of PMGs. It should be noted that the filtered CYP genes in this study were mainly from the CYP71 and CYP76 families. However, a few members of the CYP750, CYP82 and CYP736 families have also been shown to be involved in the metabolism of monoterpenes in plants [46], so genes screened by WGCNA in these families are also worthy of attention. In addition, CYP71D family genes were reported to be involved in the oxidation of cyclic monoterpenes such as limonene, menthol, and carvone [72]. Therefore, *PvCYP71D7* and *PvCYP71D9* identified in this study are also worthy of attention. Prokaryotic expression systems (e.g., *Escherichia coli*) have proven to be an efficient way to heterologously express plant TPS [73], UGT [74], and BAHD [60] family genes, while plant CYP family genes (especially for CYP71 subfamily) seem to be preferentially expressed in eukaryotic systems (e.g., yeast) [75]. Interestingly, in some studies, a prokaryotic system was also used to heterologously express plant CYP genes, but N-terminal modification and codon optimization were required [75]. Therefore, the genes filtered in this study can be further analysed for their functional attributes.

According to the phytochemical results, the aerial plant part was also found to be capable of producing PMGs. Consequently, the aerial parts (especially leaves) could be profitable as an alternative resource to increase PMG yields in the future. It would be beneficial to protect this precious plateau plant, whose resources have been threatened due to its excessive overexploitation in the herbal market as well as its short reproductive phase and low germination rate [1–3, 14]. In short, this finding significantly improves our understanding of the biosynthetic pathways of monoterpenoids in *Paeonia* and provides an efficient strategy to elucidate the complex pathway of secondary metabolites in other species.

## Methods

### Plant material

Four-year-old *P. veitchii* plants were cultivated in an experimental field on the Qinghai-Tibet Plateau of Southwest Minzu University (Hongyuan, Sichuan Province in China). Thirty independent healthy plants in the later period of blooming were harvested and equally divided into three portions. The leaves, stems, petals, ovaries, phloem and xylem were collected from each portion. Since the xylem and phloem can be easily separated from the root of *P. veitchii*, we used sharp blade to quickly separate the xylem and phloem from the clean roots. To obtain ideal full-length transcriptome unigenes, samples of the above six tissues were separately collected from the seedling, adult and flowering stages, and three independent healthy plants were selected in each stage. All samples were equally divided into two portions. One portion used for transcriptome sequencing was frozen in liquid nitrogen immediately and stored at -80 °C. Another portion used for PMG analysis was dried at 40 °C. Material collection was conducted in accordance with local legislation, and there was no need for permission from other organizations. We complied with the Convention on the Trade in Endangered Species of Wild Fauna and Flora.

### Quantitative analysis of the main PMGs

The authentic standards of oxypaeoniflora (purity≥98%, Pufeidebio, China), albiflorin (purity≥98%, Pufeidebio) and paeoniflorin (purity≥98%, Pufeidebio) were accurately weighed and then mixed with methanol. Powdered tissues (0.5 g) were accurately weighed and transferred to a conical bottle (100 mL). Then, 25 mL 80% methanol (1:50 ratio, g/mL) was added. After soaking for 2 hours, the bottle was placed in an ultrasonic machine (KQ-300DE, Kun-shan Ultrasonic Instrument Co., Ltd., China) to extract for 45 min at 60 °C and 300 W. Then, the lost weight was made up with 80% methanol and shaken well. Finally, the mixture was filtered with a 0.45 μm microporous membrane. HPLC analysis was performed using an Agilent HC-C18 column (250×4.6 mm, Agilent) with the following conditions: injection volume, 10 μL; flow rate, 1 mL/min; column temperature, 30 °C; and wavelength, 230 nm. The mobile phases of acetonitrile (A) and water with 0.1% phosphoric acid (B) were used for elution under the following conditions: 0-5 min, 5%-10% A; 5-20 min, 10%-15% A; 20-25 min, 15%-17% A; 25-40 min, 17%-19% A; 40-45 min, 19%-40% A; 45-46 min, 40%-70% A; and 46-60 min, 70% A. The method was validated by the limit of detection (LOD), limit of quantitation (LOQ), calibration curve, mean correlation coefficient, linear range, accuracy, injection precision, stability, and system suitability.

### RNA isolation, library preparation and transcriptome sequencing

Total RNA was isolated using the mirVana miRNA Isolation Kit (Cat. AM1561, Invitrogen, Thermo Fisher Scientific Inc., USA) following the manufacturer’s protocol. RNA quality and quantification were verified by an Agilent 2100 Bioanalyzer (Agilent Technologies, Santa Clara, CA, USA) and a NanoDrop 2000 spectrophotometer (Thermo Fisher Scientific, Waltham, MA, USA). Then, libraries for NGS were constructed using the TruSeq Stranded mRNA LT Sample Prep Kit (Illumina, San Diego, CA, USA) according to the manufacturer’s instructions. Eighteen samples of the six different tissues (each in three replicates) were sequenced using the Illumina HiSeq X Ten platform at OE Biotech Co., Ltd. (Shanghai, China). Equal amounts of different tissues from the seedling, adulting and flowering stages were mixed and used for cDNA synthesis using a SMARTer PCR cDNA Synthesis kit (Clontech, USA). A total of 12 PCR cycles of amplification were performed using PrimeSTAR GXL DNA Polymerase (Clontech). After purification with AMPure PB Beads, the cDNA products were then subjected to the construction of SMRTbell template libraries using the SMRTbell Template Prep Kit 1.0 (PacBio, Menlo Park, USA). Finally, the SMRT cells were sequenced using the Pacific Biosciences Sequel II platform at OE Biotech Co., Ltd.

### Gene function annotation and expression analysis

The NGS raw reads in FASTQ format were first processed to obtain clean reads using Trimmomatic. Then, the clean reads were *de novo* assembled into transcripts by Trinity (version: 2.4) with the paired-end method. The assembled transcripts were clustered using CD-HIT (identify=98%) to generate nonredundant unigenes. The SMRT raw reads were processed using PacBio SMRT Link v6.0.0 analysis package, and the high quality isoform sequences with a predicted accuracy greater than 99% were generated. High-quality isoforms were further clustered by CD-HIT (identify=98%) to obtain nonredundant unigenes. Based on the Illumina clean reads, the per kilobase per million (FPKM) values of unigenes in each tissue were obtained using Bowtie2 and eXpress softwares.

The unigenes were functionally annotated by alignment with the NCBI nonredundant (NR), Swiss-Prot, evolutionary genealogy of genes: Nonsupervised Orthologous Groups (eggNOG) and Clusters of Orthologous Groups for eukaryotic complete genomes (KOG) databases using diamond with a threshold E-value of 10^-5^. The proteins with the highest hits to the unigenes were used to assign functional annotations. The unigenes were also mapped to the Kyoto Encyclopedia of Genes and Genomes (KEGG) database to annotate the potential metabolic pathways. Gene Ontology (GO) classification was performed through the mapping relation between Swiss-Prot and GO terms. The annotation of protein families in the Pfam database was realized by HMMER3 analysis [76].

### Gene expression analysis using qRT–PCR

qRT–PCR was used to verify the reliability of the transcriptional data. Extracted RNA from eighteen sequencing samples was reverse-transcribed into cDNA using a HiScript® II Q RT SuperMix for qPCR (+gDNA wiper) Kit (Vazyme, China). All qRT–PCR primers were designed using Primer6.0 (Premier Biosoft, Canada) and synthesized by Beijing Tsingke Biotechnology Co., Ltd. The sequences were shown in Table S6. The specificity of the primers was checked by RT–PCR. A 2 × Taq Pro Universal SYBR qPCR Master Mix kit (Vazyme, China) was used for qRT–PCR in triplicate. The PCR cycles were as follows: 95 °C for 30 s (hold stage); 40 cycles at 95 °C for 5 s and 57 °C for 30 s (PCR stage); 95 °C for 15 s, 60 °C for 60 s, and 95 °C for 15 s (melting curve stage). The 2^-ΔΔCt^ comparative threshold cycle (Ct) method was used to calculate the relative expression levels [77]. Both *Actin* and *GAPDH* were used as housekeeping genes.

### Phylogenetic analysis

Unigenes belonging to *TPSs*, *CYPs*, *BAHDs*, and *UGTs* were identified using Pfam annotation and BLAST algorithm. Unigenes with a length under 1000 bp were removed. Guided by the nomenclature system [45, 51], we systematically classified and named the *CYPs* and *UGTs*. Functional sequences from other plants were used to construct a phylogenetic tree using MEGA-X (MEGA, http://www.megasoftware.net/). The phylogenetic trees of TPSs, CYPs, BAHDs, and UGTs were constructed using the neighbour-joining clustering method with the amino acid sequences of the ORFs. The topology of phylogeny was evaluated by a bootstrap resampling analysis with 1000 replicates.

### Coexpression network analysis

For further identify unigenes potentially involved in the biosynthesis of PMGs, the unsigned, weighted correlation networks were constructed by R package WGCNA [78]. First, unigenes with low fluctuation expression (standard deviation ≤ 1.0) were filtered. The power value of adjacency functions for unsigned networks was selected based on the scale-free topology criterion. Next, WGCNA network construction and module detection were conducted using an unsigned type of topological overlap matrix (TOM). The modules related to each trait were identified according to the absolute value of the correlation coefficient (≥0.3) and *p* value (<0.05). Network was visualized by R language, Python, and heatmaps [76, 78].

## Supplementary Information

**Additional file 1: Table S1.** Validation method parameters for quantification of three monoterpene glycosides. **Table S2.** Summary of transcriptome data sequenced by Illumina HiSeq X Ten platform and their pretreatment. **Table S3.** BLAST analysis of SMRT unigenes against seven public databases. **Table S4.** List of TPSs, CYPs, BAHDs, and UGTs in I, II, III, IV, and VI modules. **Table S5.** List of TPSs, CYPs, BAHDs, and UGTs, which are likely to participate in biosynthesis of PMGs. **Table S6.** Primers and annealing length in qRT-PCR.

**Additional file 2: Fig. S1.** Assembled sequence length distribution from Illumina HiSeq X Ten platform. **Fig. S2.** Non-redundant read length distribution from Pacific Biosciences Sequel platform. **Fig. S3.** SMRT unigenes distribution in GO categories under Biological process, Cellular component and Molecular function. **Fig. S4.** KEGG functional classification of unigenes from SMRT sequencing. **Fig. S5.** The network construction parameters of WGCNA.

**Additional file 3.** The full-length amino acid sequence of PvTPSs in this study.

**Additional file 4.** The full-length amino acid sequence of PvCYPs in this study.

**Additional file 5.** The full-length amino acid sequence of PvUGTs in this study.

**Additional file 6.** The full-length amino acid sequence of PvBAHDs in this study.

**Additional file 7. **List of transcripts in modules after WGCNA analysis.

**Additional file 8.** The nucleotide sequence of unigenes from SMRT sequencing.

## Data Availability

All data sets supporting the conclusions of this study are included within the article (and additional files). The raw data from the Illumina HiSeq X Ten platform have been submitted to the Sequence Read Archive (SRA) of the NCBI under accession numbers SRR19175075, SRR19175069, SRR19175060, SRR19175059, SRR19175058, SRR19175074, SRR19175073, SRR19175072, SRR19175071, SRR19175070, SRR19175068, SRR19175067, SRR19175066, SRR19175064, SRR19175065, SRR19175063, SRR19175062, and SRR19175061. The accession number of the raw data from Pacific Biosciences Sequel II platform was SRR19240505.

## Abbreviations

PMGs: *Paeonia* monoterpene glycosides
TPS: Terpene synthase
CYP: Cytochrome P450
UGT: UDP-glycosyltransferase
BAHDs: BAHD acyltransferases
SMRT: Single-molecule real-time sequencing
NGS: Next-generation sequencing
HPLC: High-performance liquid chromatography
WGCNA: Weighted gene coexpression network analysis
DXP/MEP: 1-deoxy-D-xylulose-5-phosphate/methyl-erythritol-4-phosphate
MVA: Mevalonate
IPP: Isopentenyl diphosphate
DMAPP: Dimethylallyl diphosphate
GPPS: Geranyl diphosphate synthase
GPP: Geranyl diphosphate
PlPIN: Pinene synthase
qRT–PCR: Quantitative real-time polymerase chain reaction
ORF: Open reading frame
LOD: Limit of detection
LOQ: Limit of quantitation
CCS: Circular consensus sequences
FLNC: Full-length nonchimeric
FPKM: Fragments per kilobase per million
PSPG: Plant secondary product glycosyltransferase
ACCT: Acetyl-coenzymeA (CoA) C-acetyltransferase
HMGS: 3-hydroxy-3-methylglutaryl-CoA synthase
HMGR: 3-hydroxy-3-methylglutaryl-CoA reductase
MVK: Mevalonic acid kinase
PMK: Phosphomevalonate kinase
MDC: Mevalonate-5-diphosphate decarboxylase
IDI2: IPP isomerase
DXS: Deoxyxyulose-5-phosphate synthase
DXR: Deoxyxyulose-5-phosphate reductoisomerase
CMS: 4-diphosphocytidyl-2-C-methyl-D-erythritol synthase
CMK: 4-diphosphocytidyl-2-C-methyl-D-erythritol kinase
MCS: 4-diphosphocytidyl-2-C-methyl-D-erythritol 2,4-cyclodiphosphate synthase
HDS: 1-hydroxy-2-methyl-2(E)-butenyl 4-diphosphate synthase
HDR: 1-hydroxy-2-methyl-2(E)-butenyl 4-diphosphate reductase
NR: NCBI nonredundant
eggNOG: Nonsupervised orthologous groups
KOG: Clusters of orthologous groups for eukaryotic complete genomes
KEGG: Kyoto encyclopedia of genes and genomes
GO: Gene ontology
