# Supplementary material for: Illuminating the biosynthesis pathway genes involved in bioactive specific monoterpene glycosides in Paeonia veitchii Lynch by a combination of sequencing platforms
Source: BMC Genomics. 2023 Jan 26;24:45. doi: 10.1186/s12864-023-09138-2 (PMC9878870; doi:10.1186/s12864-023-09138-2)
Supplement: Supplementary file 1 — Additional file 1: Table S1. Validation method parameters for quantification of three monoterpene glycosides. Table S2. Summary of transcriptome data sequenced by Illumina HiSeq X Ten platform and their pretreatment. Table S3. BLAST analysis of SMRT unigenes against seven public databases. Table S4. List of TPSs, CYPs, BAHDs, and UGTs in I, II, III, IV, and VI modules. Table S5. List of TPSs, CYPs, BAHDs, and UGTs, which are likely to participate in biosynthesis of PMGs. Table S6. Primers and annealing length in qRT-PCR. [file 12864_2023_9138_MOESM1_ESM.docx]

Supplementary Tables

**Table S1** Validation method parameters for quantification of three monoterpene glycosides

| **Parameters** | **oxypaeoniflora** | **albiflorin** | **paeoniflorin** |
| --- | --- | --- | --- |
| LOD (μg/ml) | 0.8 | 1.1 | 1.6 |
| LOQ (μg/ml) | 1.5 | 1.7 | 2.1 |
| Calibration curve | y=2462994.43x | y=9597000x-120520 | y=12539991.54x-354074.85 |
| Mean correlation coefficient (R^2^) | 0.9996 | 0.9996 | 1 |
| Linear range (μg/ml) | 2.0~201.0 | 2.1~212.0 | 6.7~666.0 |
| Accuracy (%, n=3) | 96.48 | 95.98 | 109.39 |
| Injection precision (RSD%, n=6) | 1.84 | 1.98 | 1.73 |
| Stability (RSD%) | 2.01 | 1.92 | 1.76 |
| System suitability (RSD%, n=6) | 1.65 | 1.8 | 2 |

**Table S2 Summary of transcriptome data sequenced by** **Illumina HiSeq X Ten platform and their pretreatment.**

| **Sample** | **Raw_Reads** | **Raw_Bases** | **Clean_Reads** | **Clean_Bases** | **Valid_Bases** | **Q30** | **GC** |
| --- | --- | --- | --- | --- | --- | --- | --- |
| Pv_Le_1 | 51.52M | 7.73G | 50.76M | 7.39G | 95.65% | 94.92% | 46.28% |
| Pv_Le_2 | 50.95M | 7.64G | 50.18M | 7.30G | 95.52% | 94.84% | 44.98% |
| Pv_Le_3 | 47.32M | 7.10G | 46.63M | 6.82G | 96.08% | 95.05% | 45.03% |
| Pv_Ov_1 | 50.68M | 7.60G | 49.96M | 7.31G | 96.16% | 94.94% | 44.57% |
| Pv_Ov_2 | 49.69M | 7.45G | 49.04M | 7.16G | 96.05% | 94.99% | 44.57% |
| Pv_Ov_3 | 50.80M | 7.62G | 50.08M | 7.25G | 95.19% | 94.92% | 44.57% |
| Pv_Pe_1 | 47.12M | 7.07G | 46.24M | 6.75G | 95.54% | 94.25% | 44.65% |
| Pv_Pe_2 | 51.50M | 7.72G | 50.50M | 7.35G | 95.19% | 94.14% | 44.55% |
| Pv_Pe_3 | 50.88M | 7.63G | 49.90M | 7.27G | 95.21% | 94.23% | 44.57% |
| Pv_Ph_1 | 50.87M | 7.63G | 50.09M | 7.32G | 95.90% | 94.67% | 44.33% |
| Pv_Ph_2 | 51.31M | 7.70G | 50.58M | 7.39G | 96.00% | 94.87% | 44.38% |
| Pv_Ph_3 | 51.10M | 7.67G | 50.39M | 7.34G | 95.72% | 94.96% | 44.30% |
| Pv_St_1 | 49.18M | 7.38G | 48.46M | 7.12G | 96.51% | 94.68% | 44.51% |
| Pv_St_2 | 48.16M | 7.22G | 47.42M | 6.94G | 96.06% | 94.65% | 44.53% |
| Pv_St_3 | 49.85M | 7.48G | 49.15M | 7.21G | 96.45% | 94.69% | 44.52% |
| Pv_Xy_1 | 48.45M | 7.27G | 47.73M | 6.95G | 95.62% | 94.92% | 44.35% |
| Pv_Xy_2 | 48.21M | 7.23G | 47.54M | 6.94G | 95.95% | 95.03% | 44.39% |
| Pv_Xy_3 | 49.43M | 7.41G | 48.73M | 7.12G | 95.96% | 94.99% | 44.41% |

Note: (1) raw_reads: the number of original reads; (2) raw_bases: the amount of original sequencing, i.e. the base number; (3) clean_reads: the number of clean reads after filtration; (4) clean_bases: the amount of sequencing after filtration, i.e. the base number; (5) valid_base: the percentage of effective bases; (6) Q30: the percentage of the base with the Phred value greater than 30; (7) GC: the percentage of the total number of G and C bases in clean bases.

**Table S3 BLAST analysis of SMRT unigenes against seven public databases**

| **Anno_Database** | **Annotated_Number** | **300<=length<1000** | **length>=1000** |
| --- | --- | --- | --- |
| NR | 28441(92.26 %) | 1988(6.45 %) | 26426(85.72 %) |
| Swissprot | 24602(79.81 %) | 1659(5.38 %) | 22923(74.36 %) |
| KEGG | 4850(15.73 %) | 621(2.01 %) | 4223(13.70 %) |
| KOG | 18826(61.07 %) | 1172(3.80 %) | 17647(57.25 %) |
| eggNOG | 28027(90.92 %) | 1931(6.26 %) | 26070(84.57 %) |
| GO | 22356(72.52 %) | 1588(5.15 %) | 20748(67.30 %) |
| Pfam | 26288(85.28 %) | 1705(5.53 %) | 24583(79.75 %) |

**Table S4** List of TPSs, CYPs, BAHDs, and UGTs in I, II, III, IV, and VI modules.

| Name | Sequencing_ID | Name | Sequencing_ID |
| --- | --- | --- | --- |
| PvTPS1P | transcript_10655 | PvCYP71A9 | transcript_20717 |
| PvTPS3 | transcript_18005 | PvCYP71B1 | transcript_26859 |
| PvTPS4 | transcript_19626 | PvCYP71B10 | transcript_27613 |
| PvTPS5P | transcript_21833 | PvCYP71B12 | transcript_30429 |
| PvTPS7 | transcript_22581 | PvCYP71B14P | transcript_26692 |
| PvTPS8 | transcript_24035 | PvCYP71B15P | transcript_26459 |
| PvTPS9 | transcript_24267 | PvCYP71B18P | transcript_26569 |
| PvTPS11 | transcript_25120 | PvCYP71B19P | transcript_35064 |
| PvTPS12 | transcript_26824 | PvCYP71B2 | transcript_28323 |
| PvTPS14 | transcript_33280 | PvCYP71B3 | transcript_25828 |
| PvTPS17P | transcript_37260 | PvCYP71B4P | transcript_29524 |
| PvTPS18P | transcript_37763 | PvCYP71B5 | transcript_28128 |
| PvBAHD2 | transcript_29492 | PvCYP71B6 | transcript_28619 |
| PvBAHD3 | transcript_27276 | PvCYP71B7 | transcript_29365 |
| PvBAHD7 | transcript_29257 | PvCYP71B8 | transcript_28613 |
| PvBAHD8 | transcript_29397 | PvCYP71D10P | transcript_18242 |
| PvBAHD10 | transcript_30955 | PvCYP71D8P | transcript_35203 |
| PvBAHD12 | transcript_31264 | PvCYP72A1 | transcript_25891 |
| PvBAHD13P | transcript_32662 | PvCYP72A2 | transcript_25030 |
| PvBAHD14 | transcript_32398 | PvCYP72A5 | transcript_27549 |
| PvUGT71B3P | transcript_28577 | PvCYP72A7P | transcript_26671 |
| PvUGT71B4 | transcript 29093 | PvCYP72B1P | transcript_15669 |
| PvUGT71B5 | transcript 30518 | PvCYP736A11 | transcript_26998 |
| PvUGT72A1 | transcript 29273 | PvCYP736A12 | transcript_23512 |
| PvUGT73B4 | transcript_30136 | PvCYP736B1 | transcript_28289 |
| PvUGT76C2 | transcript_28460 | PvCYP736B2 | transcript_27006 |
| PvUGT85A4 | transcript_29034 | PvCYP736B3 | transcript_27393 |
| PvUGT85B1 | transcript_31865 | PvCYP73A5 | transcript_27575 |
| PvUGT85C2 | transcript_30114 | PvCYP749A16P | transcript_31110 |
| PvUGT85C3 | transcript_28844 | PvCYP749A17P | transcript_37212 |
| PvUGT85C4 | transcript_27842 | PvCYP749A18 | transcript_27935 |
| PvUGT85C5 | transcript_28414 | PvCYP749A19 | transcript_24358 |
| PvUGT84A1 | transcript_28456 | PvCYP749A20 | transcript_25195 |
| PvUGT84A2P | transcript_35508 | PvCYP749A21 | transcript_27132 |
| PvUGT75C1 | transcript_29984 | PvCYP749A23 | transcript_28734 |
| PvUGT74F2 | transcript_31811 | PvCYP74B2 | transcript_29544 |
| PvUGT74F1 | transcript_31006 | PvCYP75A1 | transcript_27851 |
| PvUGT74E1 | transcript_31200 | PvCYP75A2 | transcript_22124 |
| PvUGT74D2 | transcript_31979 | PvCYP75A3 | transcript_28980 |
| PvUGT74D3 | transcript_30777 | PvCYP75B3 | transcript_29263 |
| PvUGT74D4 | transcript_30980 | PvCYP76A1 | transcript_29509 |
| PvUGT74D5 | transcript_32256 | PvCYP76A2 | transcript_29311 |
| PvUGT88B1 | transcript_31547 | PvCYP76A3 | transcript_28046 |
| PvUGT92A1 | transcript_28749 | PvCYP76F14 | transcript_28585 |
| PvUGT91A1 | transcript 29741 | PvCYP76T1 | transcript_25276 |
| PvUGT91A2 | transcript 30159 | PvCYP76T2 | transcript_28348 |
| PvUGT71B2P | transcript_36615-P | PvCYP76T3 | transcript_27291 |
| PvUGT709K1 | transcript 29955 | PvCYP76T5 | transcript_28248 |
| PvUGT91C1 | transcript 30404 | PvCYP76T6 | transcript_29270 |
| PvUGT71B7 | transcript 30852 | PvCYP76T7 | transcript_29437 |
| PvCYP714C2P | transcript_37286 | PvCYP76T8P | transcript_28298 |
| PvCYP704B1 | transcript_27069 | PvCYP77B1 | transcript_28523 |
| PvCYP706B1 | transcript_26956 | PvCYP81D1 | transcript_28317 |
| PvCYP712A1 | transcript_27940 | PvCYP81D2 | transcript_26021 |
| PvCYP714A1 | transcript_27046 | PvCYP82C2 | transcript_28034 |
| PvCYP714A2 | transcript_28097 | PvCYP82C3 | transcript_28549 |
| PvCYP714B1 | transcript_27604 | PvCYP86B1 | transcript_29241 |
| PvCYP714B2 | transcript_27682 | PvCYP89A9 | transcript_28678 |
| PvCYP716A1 | transcript_26918 | PvCYP90B1 | transcript_27332 |
| PvCYP716A3 | transcript_28306 | PvCYP90C2 | transcript_24571 |
| PvCYP716B1 | transcript_30717 | PvCYP90C4 | transcript_26279 |
| PvCYP71A1 | transcript_26155 | PvCYP97B2 | transcript_24556 |
| PvCYP71A3P | transcript_34162 | PvCYP97C1 | transcript_24018 |
| PvCYP71A4P | transcript_30076 | PvKO | transcript_28048 |
| PvCYP71A8 | transcript_30277 | PvCYP97B3 | transcript_23689 |

**Table S5** List of TPSs, CYPs, BAHDs, and UGTs, which are likely to participate in biosynthesis of PMGs.

| Name | Name | Name |
| --- | --- | --- |
| PvTPS3 | PvUGT85C3 | PvCYP71B7 |
| PvTPS4 | PvUGT85C4 | PvCYP71B8 |
| PvTPS7 | PvUGT85C5 | PvCYP76A1 |
| PvTPS8 | PvCYP706B1 | PvCYP76A2 |
| PvTPS9 | PvCYP71A1 | PvCYP76A3 |
| PvTPS11 | PvCYP71A8 | PvCYP76F14 |
| PvTPS12 | PvCYP71A9 | PvCYP76T1 |
| PvTPS14 | PvCYP71B1 | PvCYP76T2 |
| PvBAHD7 | PvCYP71B10 | PvCYP76T3 |
| PvBAHD12 | PvCYP71B12 | PvCYP76T5 |
| PvUGT76C2 | PvCYP71B2 | PvCYP76T6 |
| PvUGT85A4 | PvCYP71B3 | PvCYP76T7 |
| PvUGT85B1 | PvCYP71B5 |  |
| PvUGT85C2 | PvCYP71B6 |  |

**Table S6 Primers and annealing length in qRT-PCR.**

| **Gene name** | **F-primer(5'to3')** | **R-primer(5'to3')** | **length(bp)** |
| --- | --- | --- | --- |
| *PvTPS3* | GCACTGGACATCTTATGTCTTCC | TGTGCAGACGGGCTTCTTCT | 140 |
| *PvTPS8* | TTCAATCAAGTCCCCTGAGCC | TGCACCAACAATCGACCGAA | 169 |
| *PvTPS13* | ATCCTACAAACAGGCGGTCG | GGCCTAACCGTTGGATGTCA | 207 |
| *PvTPS12* | ATGATCGACACCATCCAGCG | AGGCGAAATGTAAGCGCAAC | 140 |
| *PvCYP71A1* | GCTTCCAACCCTTCCTATTTGC | CTGCCAAGCGACAGAGAGAT | 177 |
| *PvCYP706B1* | GGCCTGCCAGTAGTTGGATA | CATGGTCCCGAACCACCTC | 166 |
| *PvCYP82C3* | CAGAAAAACAGCTCCCAAAGCA | ACGGGTCCAAATTGGTCTGC | 120 |
| *PvCYP76A1* | AGCTGCTCTAGTTGTGGTTGT | CGAGTCTTAACCAGAGGACCG | 163 |
| *PvCYP736A11* | GGCACCACATGAGCATCAAA | CACAAAGCCAAAGCGCATGT | 236 |
| *PvBAHD2* | ACTGGCAAAAGCCACATCAAG | ACCCATCCTTCAACTTGCTCA | 160 |
| *PvBAHD7* | GAAGGCCTCCGGTTCCAAAT | TCCACAACAAGCTTACGCCC | 170 |
| *PvBAHD6* | TCCAACCCCAACCCATCTTC | TCAACCTCCCAGCTAAAGGG | 167 |
| *PvUGT85C2* | CTACGAGACCATTCCCGACG | AAGCCAAAGACCCATCGGAG | 191 |
| *PvUGT72A1* | GATGACATGACCGTCGTTGC | GGTGGATGCAGTGAAAAACGA | 183 |
| *PvUGT91A1* | CTGATGATACTGGCGTGTCT | GAAGCTCACCAACCGGAAGA | 156 |
| *PvUGT91A2* | TGTTTGGAGACAAGGACAGACA | GCACCACATGAGTACATGACC | 127 |
| *Actin* | ACGGCTACTCATTCCCTCATCT | GAACCGCTCAGTGCCAACTTTA | 265 |
| *GAPDH* | TGTTCACCGACAAAGACAAGG | TAGCCAAGGGAGCAAGACAAT | 183 |
