# Supplementary material for: Illuminating the biosynthesis pathway genes involved in bioactive specific monoterpene glycosides in Paeonia veitchii Lynch by a combination of sequencing platforms
Source: BMC Genomics. 2023 Jan 26;24:45. doi: 10.1186/s12864-023-09138-2 (PMC9878870; doi:10.1186/s12864-023-09138-2)
Supplement: Supplementary file 2 — Additional file 2: Fig. S1. Assembled sequence length distribution from Illumina HiSeq X Ten platform. Fig. S2. Non-redundant read length distribution from Pacific Biosciences Sequel platform. Fig. S3. SMRT unigenes distribution in GO categories under Biological process, Cellular component and Molecular function. Fig. S4. KEGG functional classification of unigenes from SMRT sequencing. Fig. S5. The network construction parameters of WGCNA. [file 12864_2023_9138_MOESM2_ESM.docx]

Supplementary Figures


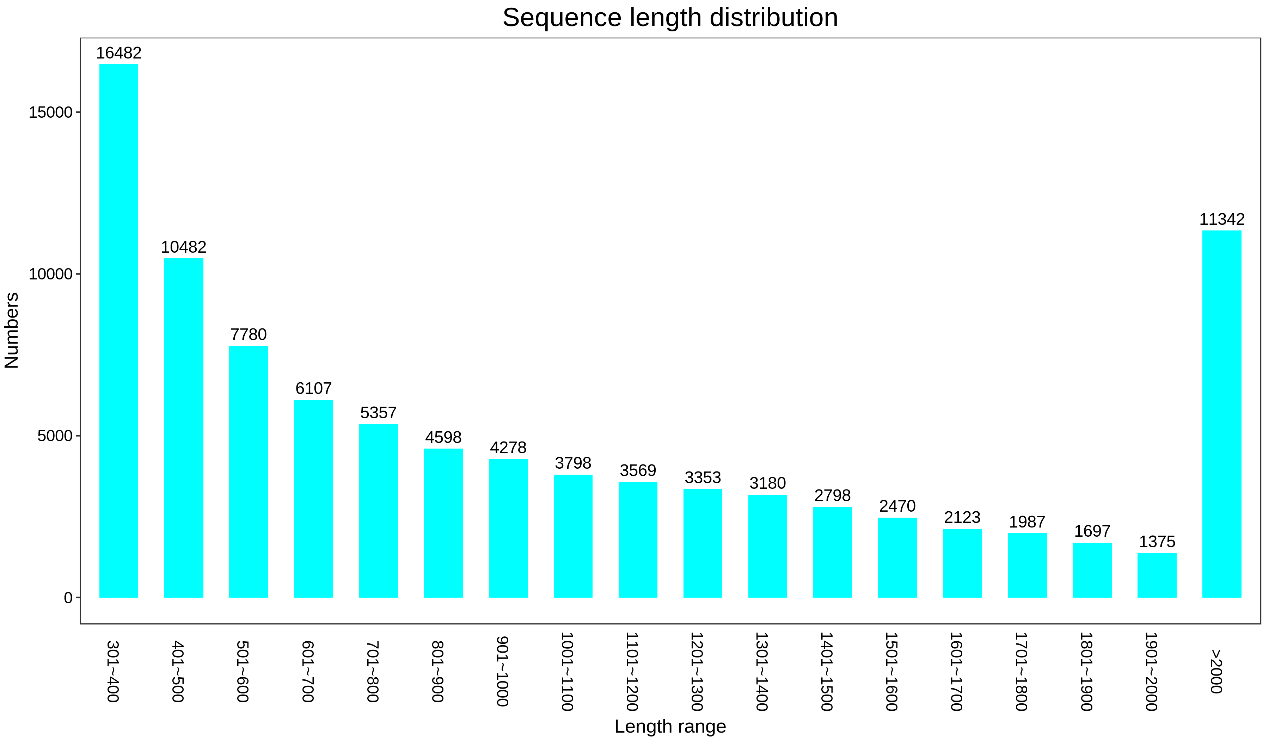


**Fig. S1 Assembled sequence length distribution from Illumina HiSeq X Ten platform.**


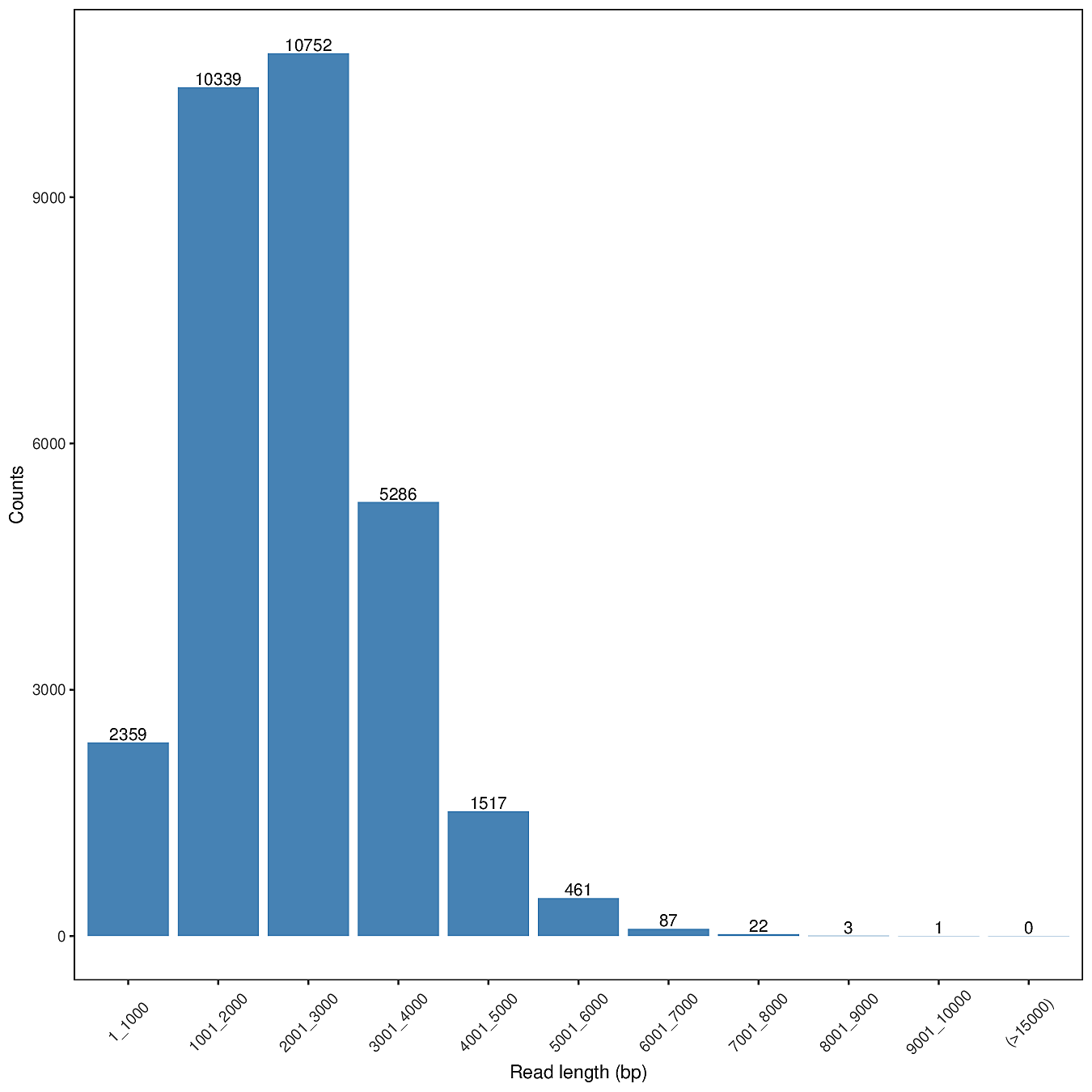


**Fig. S2 Non-redundant read length distribution from Pacific Biosciences Sequel platform.**


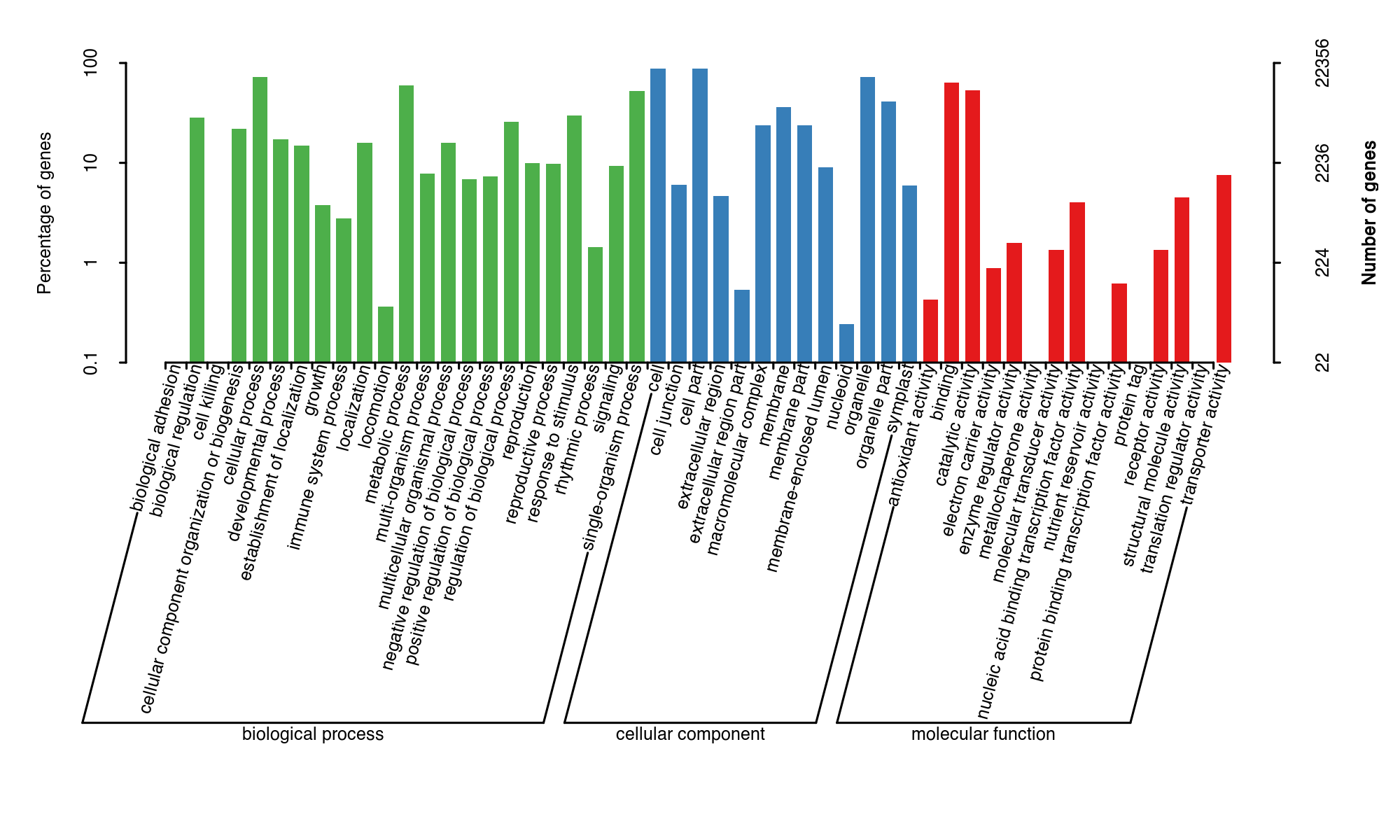
Fig. S3 SMRT unigenes distribution in GO categories under Biological process, Cellular component and Molecular function.


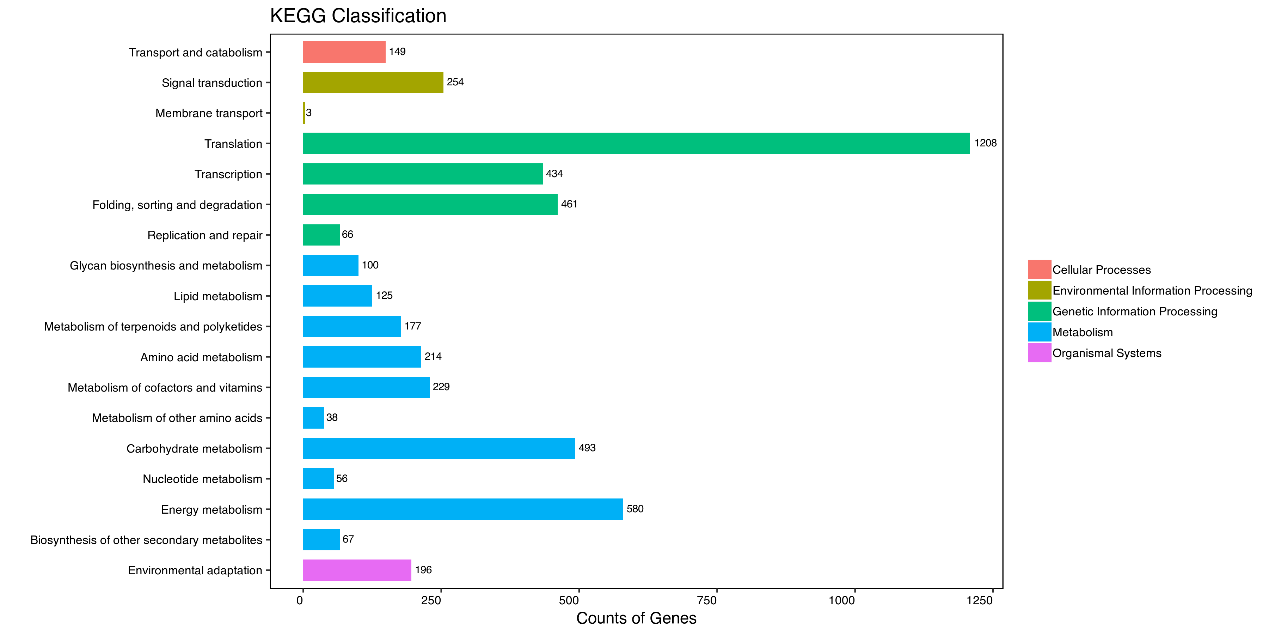


Fig. S4 KEGG functional classification of unigenes from SMRT sequencing.

Fig. S5 The network construction parameters of WGCNA. A: the correlation coefficients corresponding to different power values. B: the average connectivity of the network constructed with different power values. A and B showed that when the power value is 30, the correlation coefficient and the average connection degree of the constructed network are both higher. C: when the power value is 30, the distribution of network connectivity. D: when the power value is 30, the result of power-law distribution. A and B showed that there is a negative correlation between k and p (the correlation coefficient is 0.73), indicating that the selected power value (30) can establish a gene scale-free network.
