# Supplementary material for: Illuminating the biosynthesis pathway genes involved in bioactive specific monoterpene glycosides in Paeonia veitchii Lynch by a combination of sequencing platforms
Source: BMC Genomics. 2023 Jan 26;24:45. doi: 10.1186/s12864-023-09138-2 (PMC9878870; doi:10.1186/s12864-023-09138-2)
Supplement: Supplementary file 3 — Additional file 3. The full-length amino acid sequence of PvTPSs in this study. [file 12864_2023_9138_MOESM3_ESM.docx]

**The full-length amino acid sequence of PvTPSs in this study**

>PvTPS2

MATRLVASSPLCSFVKFQPVKAPSQKYFSNASFPKPVFRCVATSTTEVIERRSGNYQPCIWDIEFLQTVDSDYSKEIYKKRSENLKRQVKTMLVSEVEPLVQLELIDVLQRLCLGYEFETEIKSILQSIYNNHSSSSGKYKSPQDLHAAALEFRLLRQHHYIVPQEIFESFRDENGFKKSIYDDVKGMLSLYEASFFAFEDESIMEEAWEFTSEKLKSLKTEDMDPTLAMLVPHALEIPLHWRLPRFDARWFIEVYEKSRDMNPILLELGKLDFNIVQSMYQEDLRVMSSWWKEIALREKLDFARDRLVPSYLWGVGVSSEPQYAYSRRIIGMDIAIINMIDDIYDIYGTLEEVELFTDAVKKWDINAMKQLPDYMKIAYLAFYNTVNEMIYVILKEHGVDVTDHLTKAWLGLLNGYLTEARWYHTKHKPSLAEYMKNACMSIAGPLIATLAYLTTHKSITEEEMKYLESIPDVMHWTSYVFRISDDYGTSSDELKRGDVPKAIQCYMHDSGVSEEDARLHMRKLVRNGWKKVNKYRFMESQESPLTPTL

VEMMQNLTRVSQNLYEHGDGHGIEDGETKDRVLRLLFQPIPM

>PvTPS3

MATRLVASSPLCSFVKFQPVKAPSQKYSSNASFPKPVFRCVATSTTEVIERRSGNYQPCIWDIEFLQTVDSDYSKEIYKKRSENLKRQVKTMLVSEVEPLVQLELIDVLQRLCLGYEFETEIKSILQSIYNNNSSSSGKYKSPQDLHAAALEFRLLRQHHYIVPQEIFESFRDENGFKKSIYDDVKGMLSLYEASFFAFEDESIMEEAWEFTSEKLKSLKTEDMDPTLAMLVPHALEIPLHWRLPRFDARWFIEVYEKSRDMNPILLELGKLDFNIVQSMYQEDLRVMSSWWKEIALREKLDFARDRLVPSYLWGVGVSSEPQYAYSRRIIGMDIAIINMIDDIYDIYGTLEEVELFTDAVKKWDINAMKQLPDYMKIAYLAFYNTVNEMIYVILKEHGVDVTDHLTKAWLGLLNGYLTEARWYHTKHKPSLAEYMKNACMSIAGPLIATLAYLTTHKSITEEEMKYLESIPDVMHWTSYVFRISDDYGTSSDELKRGDVPKAIQCYMHDSGVSEEEARLHMRKLVRNGWKKVNKYRFMESQESPLTPTLVEMMQNLTRVSQNLYEHGDGHGIEDGETKDRVLRLLFQPIPM

>PvTPS4

MATRLVASSPLCSFVKFQPVKAPSQKYSSNASFPKPVFRCVATSTTEVIERRSGNYQPCIWDIEFLQTVDSDYSKEIYKKRSENLKRQVKTMLVSEVEPLVQLELIDVLQRLCLGYEFETEIKSILQSIYNNHSSSSGKYKSSQDLHAAALEFRLLRQHHYIVPQEIFESFRDENGFKKSIYDDVKGMLSLYEASFFAFEDESIMEEAWEFTSEKLKSLKTEDMDPTLAMLVPHALEIPLHWRLPRFDARWFIEVYEKSRDMNPILLELGKLDFNIVQSMYQEDLRVMSSWWKEIALREKLDFARDRLVPSYLWGVGVSSEPQYAYSRRIIGMDIAIINMIDDIYDIYGTLEEVELFTDAVKKWDINAMKQLPDYMKIAYLAFYNTVNEMIYVILKEHGVDVTDHLTKAWLGLLNGYLTEARWYHTKHKPSLAEYMKNACMSIAGPLIATLAYLTTHKSITEEEMKYLESIPDVMHWTSYVFRISDDYGTSSDELKRGDVPKAIQCYMHDSGVSEEDARLHMRKLVRNGWKKVNKYRFMESQESPLTPTLVEMMQNLTRVSQNLYEHGDGHGIEDGETKDRVLRLLFQPIPM

>PvTPS6

MATRLVASSPLCSFVKFQPVKAPSQKYSSNASFPKPVFRCVATSTTEVIERRSGNYQPCIWDIEFLQTVDSDYSKEIYKKRSENLKRQVKTMLVSEVEPLVQLELIDVLQRLCLGYEFETEIKSILQSIYNNHSSSSGKYKSSQDLHAAALEFRLLRQHHYIVPQEIFESFRDENGFKKSIYDDVKGMLSLYEASFFAFEDESIMEEAWEFTSEKLKSLKTEDMDPTLAMLVPHALEIPLHWRLPRFDARWFIEVYEKSRDMNPILLELGKLDFNIVQSMYQEDLRVMSSWWKEIALREKLDFARDRLVPSYLWGVGVSSEPQYAYSRRIIGMDIAIINMIDDIYDIYGTLEEVELFTDAVKKWDINAMKQLPDYMKIAYLAFYNTVNEMIYVILKEHGVDVTDHLTKAWLGLLNGYLTEARWYHTKHKPSLAEYMKNACMSIAGPLIATLAYLTTHKSITEEEMKYLESIPDVMHWTSYVFRISDDYGTSSDELKRGDVPKAIQCYMHDSGVSEEDARLHMRKLNLTRVSQNLYEHGDGHGIEDGETKD

RVLRLLFQPIPM

>PvTPS7

MLVSEVEPLVQLELIDVLQRLCLGYEFETEIKSILQSIYNNHSSSSGKYKSSQDLHAAALEFRLLRQHHYIVPQEIFESFRDENGFKKSIYDDVKGMLSLYEASFFAFEDESIMEEAWEFTSEKLKSLKTEDMDPTLAMLVPHALEIPLHWRLPRFDARWFIEVYEKSRDMNPILLELGKLDFNIVQSMYQEDLRVMSSWWKEIALREKLDFARDRLVPSYLWGVGVSSEPQYAYSRRIIGMDIAIINMIDDIYDIYGTLEEVELFTDAVKKWDINAMKQLPDYMKIAYLAFYNTVNEMIYVILKEHGVDVTDHLTKAWLGLLNGYLTEARWYHTKHKPSLAEYMKNACMSIAGPLIATLAYLTTHKSITEEEMKYLESIPDVMHWTSYVFRISDDYGTSSDELKRGDVPKAIQCYMHDSGVSEEDARLHMRKLVRNGWKKVNKYRFMESQESPLTPTLVEMMQNLTRVSQNLYEHGDGHGIEDGETKDRVLRLLFQPIPM

>PvTPS8

MATRLVASSPLCSFVKFQPVKAPSQKYSSNASFPKPVFRCVATSTTEVIERRSGNYQPCIWDIEFLQTVDSDYSKEIYKKRSENLKRQVKTMLVSEVEPLVQLELIDVLQRLCLGYEFETEIKSILQSIYNNHSSSSGKYKSPQDLHAAALEFRLLRQHHYIVPQEIFESFRDENGFKKSIYDDVKGMLSLYEASFFAFEDESIMEEAWEFTSEKLKSLKTEDMDPTLAMLVPHALEIPLHWRLPRFDARWFIEVYEKSRDMNPILLELGKLDFNIVQSMYQEDLRVMSSWWKEIALREKLDFARDRLVPSYLWGVGVSSEPQYAYSRRIIGMDIAIINMIDDIYDIYGTLEEVELFTDAVKKWDINAMKQLPDYMKIAYLAFYNTVNEMIYVILKEHGVDVTDHLTKAWLGLLNGYLTEARWYHTKHKPSLAEYMKNACMSIAGPLIATLAYLTTHKSITEEEMKYLESIPDVMHWTSYVFRISDDYGTSSDELKRGDVPKAIQCYMHDSGVSEEDARLHMRKLVRNGWKKVNKYRFMESQESPLTPTLVEMMQNLTRVSQNLYEHGDGHGIEDGETKDRVLRLLFQPIPM

>PvTPS9

MLVSEVEPLVQLELIDVLQRLCLGYEFETEIKSILQSIYNNNSSSSGKYKSPQDLHAAALEFRLLRQHHYIVPQEIFESFRDENGFKKSIYDDVKGMLSLYEASFFAFEDESIMEEAWEFTSEKLKSLKTEDMDPTLAMLVPHALEIPLHWRLPRFDARWFIEVYEKSRDMNPILLELGKLDFNIVQSMYQEDLRVMSSWWKEIALREKLDFARDRLVPSYLWGVGVSSEPQYAYSRRIIGMDIAIINMIDDIYDIYGTLEEVELFTDAVKKWDINAMKQLPDYMKIAYLAFYNTVNEMIYVILKEHGVDVTDHLTKAWLGLLNGYLTEARWYHTKHKPSLAEYMKNACMSIAGPLIATLAYLTTHKSITEEEMKYLESIPDVMHWTSYVFRISDDYGTSSDELKRGDVPKAIQCYMHDSGVSEEEARLHMRKLVRNGWKKVNKYRFMESQESPLTPTLVEMMQNLTRVSQNLYEHGDGHGIEDGETKDRVLRLLFQPIPM

>PvTPS10

MAYFNTSSALRHSQISTNPTDITSKPTAKKWNLAQDHSSIATPPLQDKIKEARHVLMSKKVEDPFQSLVMIDTIQRLGIAHHFQEDVKTLLHKEYMGGHCLNRHHLHEVALTFRLLRQQGYHVPADVFNKYMDKNGQLEEKLSEDLRGLIGLYEAAQLSIPGEDILDQAANLCNNLLSACLSGLNLNESEARVVETTLKHPYHKSLSRFMPQNFISGSQGEEEWIHVVQDLAILDYKMVQPIYHKEMRQISLWWKDLGLAKELKFARNQPLKWYMWSTAVLTDPRFTEQRVDLTKPISLIYIIDDIFDVYGTLDELTLFTETVTRWDISKVNHLPDYMKTCFKALYDITNEIAYKFCVEHGWNPIETLKKTWVSLCNAFLLESKWFNSKDLPEAEEYLRNGIISSGIHVVLVHLFFLLGGEGISKESIHVIKGDTMPGIIFFTAKILRLYDDLGSAKDENQDGHDGSYVECYMKEHQGCSVESAREYTLHMISDAWKCLNKECLSPNPFSTSFINASLNAARMVPLMYTYDDNHNLSSLKEHMKSFLYGTLSL

>PvTPS11

MSQYLQCIANATPNYNPTNRRSANYQPSIWSQDFLESVQTDYYAENTYMEWAKKLEEEVRNTMTNDNLGQLEMLELITDIQRLGLERLFINDTRRALENLVSLERSNAKTENDLYATALRFRLLRHYGYQIYPDVFQSFKDSRGFFMGCLGKDVKGMLSLYEASHLALEGEKILEEAMEFASIHLKDIKEYISNKHVKLVLDHVLELPLHRRTQMFDARWYIEAYNKKEDANETLLELAKLNFNMVQATIQRDLRDVSRWWKDIGLKNKLNFTRDRLMECFFWTFGIAPEPQLSDCRRGLTKVAALITTIDDIYDVYGSLDELELFTDGVERWDVNALKSLPEYMQLSFLALFNTVNEMAYDTLKEHGVNSIPYLAKAWANLCKAFLVEAKWNYHKCIPTFNDYLNNGWQSVSGAVVLIHAYFLVTQNISYDALECLVKDHDLLRWPSMIFRLCNDLGTSKAELKREEIVSSVMCYMVETGASEEIAHKHVKDLINKSWKKLNRYLVDDSEFENPFIKIAINLARIVQCIYMYGDGHGAPDMRTKSQILSLIVEPIPVMERYVVNTNVN

>PvTPS12

MLSLYEASFFAFEDESIMEEAWEFTSEKLKSLKTEDMDPTLAMLVPHALEIPLHWRLPRFDARWFIEVYEKSRDMNPILLELGKLDFNIVQSMYQEDLRVMSSWWKEIALREKLDFARDRLVPSYLWGVGVSSEPQYAYSRRIIGMDIAIINMIDDIYDIYGTLEEVELFTDAVKKWDINAMKQLPDYMKIAYLAFYNTVNEMIYVILKEHGVDVTDHLTKAWLGLLNGYLTEARWYHTKHKPSLAEYMKNACMSIAGPLIATLAYLTTHKSITEEEMKYLESIPDVMHWTSYVFRISDDYGTSSDELKRGDVPKAIQCYMHDSGVSEEDARLHMRKLVRNGWKKVNKYRFMESQESPLTPTLVEMMQNLTRVSQNLYEHGDGHGIEDGETKDRVLRLLFQPIPM

>PvTPS13

MEEAWEFTSEKLKSLKTEDMDPTLAMLVPHALEIPLHWRLPRFDARWFIEVYEKSRDMNPILLELGKLDFNIVQSMYQEDLRVMSSWWKEIALREKLDFARDRLVPSYLWGVGVSSEPQYAYSRRIIGMDIAIINMIDDIYDIYGTLEEVELFTDAVKKWDINAMKQLPDYMKIAYLAFYNTVNEMIYVILKEHGVDVTDHLTKAWLGLLNGYLTEARWYHTKHKPSLAEYMKNACMSIAGPLIATLAYLTTHKSITEEEMKYLESIPDVMHWTSYVFRISDDYGTSSDELKRGDVPKAIQCYMHDSGVSEEEARLHMRKLVRNGWKKVNKYRFMESQESPLTPTLVEMMQNLTRVSQNLYEHGDGHGIEDGETKDRVLRLLFQPIPM

>PvTPS14

MNPILLELGKLDFNIVQSMYQEDLRVMSSWWKEIALREKLDFARDRLVPSYLWGVGVSSEPQYAYSRRIIGMDIAIINMIDDIYDIYGTLEEVELFTDAVKKWDINAMKQLPDYMKIAYLAFYNTVNEMIYVILKEHGVDVTDHLTKAWLGLLNGYLTEARWYHTKHKPSLAEYMKNACMSIAGPLIATLAYLTTHKSITEEEMKYLESIPDVMHWTSYVFRISDDYGTSSDELKRGDVPKAIQCYMHDSGVSEEDARLHMRKLVRNGWKKVNKYRFMESQESPLTPTLVEMMQNLTRVSQNLYEHGDGHGIEDGETKDRVLRLLFQPIPM

>PvTPS15

MDIAIINMIDDIYDIYGTLEEVELFTDAVKKWDINAMKQLPDYMKIAYLAFYNTVNEMIYVILKEHGVDVTDHLTKAWLGLLNGYLTEARWYHTKHKPSLAEYMKNACMSIAGPLIATLAYLTTHKSITEEEMKYLESIPDVMHWTSYVFRISDDYGTSSDELKRGDVPKAIQCYMHDSGVSEEEARLHMRKLVRNGWKKVNKYRFMESQESPLTPTLVEMMQNLTRVSQNLYEHGDGHGIEDGETKDRVLRLLFQPIPM
