# Supplementary material for: Illuminating the biosynthesis pathway genes involved in bioactive specific monoterpene glycosides in Paeonia veitchii Lynch by a combination of sequencing platforms
Source: BMC Genomics. 2023 Jan 26;24:45. doi: 10.1186/s12864-023-09138-2 (PMC9878870; doi:10.1186/s12864-023-09138-2)
Supplement: Supplementary file 4 — Additional file 4. The full-length amino acid sequence of PvCYPs in this study. [file 12864_2023_9138_MOESM4_ESM.docx]

**The full-length amino acid sequence of PvCYPs in this study**

>PvCYP51G1

MVAMDSESKFFNVGLLIVATLVVAKLISLLIMPRSTKRLPPVIKAWPVIG

GLVRFLKGPIVMLREEYPKLGSVFTLNLFTRNITFLIGPEVSAHFFKASE

SDLSQQEVYQFNVPTFGPGVVFDVDYTVRQEQFRFFTEALRVNKLKGYVD

QMILEAEDYFSKWGDSGEVDLKYELEHLIILTASRCLLGREVRDKLFDDV

SALFHDLDNGMLPISVIFPYLPIPAHRRRDQARKRLSEIFANIITSRKCT

GKSENDVLQCFIDSKYKDGRPTTEGEVTGLLIAALFAGQHTSSITSTWTG

AYLLCNKEYLSAVLDEQKNLMGKHGNKVDHDILAEMDVLYRCIKEALRLH

PPLIMLLRSSHSDFTVKAKEGKEYDIPKGHIVATSPAFANRLPHVFKEPD

RYDPDRFAVGREEDKAAGAFSYISFGGGRHGCLGEPFAYLQIKAIWSHLL

RNFEFELISPFPEIDWNAMVVGVKGKVMVRYKRRVLSVN

>PvCYP51G2

MVAMDSESKFFNVGLLIVATLVVAKLISLLIMPRSTKRLPPVIKAWPVIG

GLVRFLKGPIVMLREEYPKLGSVFTLNLFTRNITFLIGPEVSAHFFKASE

SDLSQQEVYQFNVPTFGPGVVFDVDYTVRQEQFRFFTEALRVNKLKGYVD

QMILEAEDYFSKWGDSGEVDLKYELEHLIILTASRCLLGREVRDKLFDDV

SALFHDLDNGMLPISVIFPYLPIPAHRRRDQARKRLSEIFANIITSRKCT

GKSENDVLQCFIDSTYKDGRPTTEGEVTGLLIAALFAGQHTSSITSTWTG

AYLLSNKEYLSAVLDEQKNLMGKHGNKVDHDILAEMDVLYRCIKEALRLH

PPLIMLLRSSHSDFTVKTKEGKEYDIPKGHIVATSPAFANRLPHVFKEPD

RYDPDRFAVGREEDKAAGAFSYISFGGGRHGCLGEPFAYLQIKAIWSHLL

RNFEFELISPFPEIDWNAMVVGVKGKVMVRYKRRVLSVN

>PvCYP71A1

MSLFQWLKEGFQPFLFASICLAVVVKLLLKLKSNERRHNLPPSPPKLPII

GNLHQLGNMPHISLCRLAEKFGPIIYLQLGQIPTVVVSSSRMAKEVMKTH

DLALSSRPKIFSAKHLFYNCTDMVFSPYGAYWRHIRKICILELLSAKRVQ

SFSFVREEEVARLVERIAVSSPGTTDLSKLIGLYANSVVCRVAFSKDYSE

GGDYDRYGFQKLLEDYQTLLGGFSTGDFFPSLEFIHSLTGMKSKLQNTFR

RFDQFFDEMIKEHLDPEREKQEHKDLVDVLLDIQKNGSQEMPLTMDNVKA

IILDMFAAGTDTTFITLDWGMTELIMNPKVMERAQAEVRGIVGKRRIVLE

SDLPQLHYMTATIKEIFRLHPPAPVLVPRESMEDITIDGYDIPVKTRIFV

NAWAIGRNSESWENPEKFEPERFMGSTIDFKGTDFELIPFGAGRRSCPAI

TFGTASVELALAQLLHSFDWELPPGIKSEDLDMTEVFGITMHRKSHLAVI

AKPRFHG

>PvCYP71A2

MSLFQWLKEGFQPFLFASVCLAVVVKLLLKLKSNERRHNLPPSPPKLPII

GNLHQLGNMPHISLCRLAEKFGPIIYLQLGQIPTVVVSSSRMAKEVMKTH

DLALSSRPKIFSAKHLFYNCTDMVFSPYGAYWRHIRKICILELLSAKRVQ

SFSFVREEEVARLVERVAVSSPGTTDLSKLIGLYANSVVCRVAFSKDYSE

GGDYDRYGFQKLLEDYQTLLGGFSTGDFFPSLEFIHSLTGMKSKLQNTFR

RFDQFFDEMIKEHLDPEREKQEHKDLVDVLLDIQKNRSQEMPLTMDNVKA

IILDMFAAGTDTTFITLDWGMTELIMNPKVMERAQAEVRGIVGKRRIVLE

SDLPQLHYMTATIKEIFRLHPPAPVLVPRESMEDITIDGYDIPVKTRIFV

NAWAIGRNSESWENPEKFEPERFMGSTIDFKGTDFELIPFGAGRRSCPAI

TFGTASVELALAQLLHSFDWELPPGIKSEDLDMTEVFGITMHRKSHLAVI

AKPRFHG

>PvCYP71A8

MVLSSSSSWLLASSFILLIFLMMNQMKKTRRLPPGPKKLPLIGHLHHLRD

LPHRSLRHLSQKYGPIMFLQLGSIPTLIISSAGIARQVFKTHDLIFSNRP

ILYAASKLSYGCSDVSFARYGEYWRQVRKIVILELLSLKRVQSFEAVRDE

ELKIMLHAIAQSSSSSSVAFNLSQAALLLTNNIICRIVFGRKFDGGEENR

GASKFQEMLEEILDLLAGFNIADLFPWIKWVSKFNGFEAKVDKNFRQMDE

LFDQVIEEHLDPQRPEREDKDLVDILLGLQKDQNQAFTLTDRNIKGVLMD

MFVAGTDTSAATLIWIMAELMKNPSVMRRAQDEVRRVGKSKGKVEEEDLS

QLGYLKLVVKEGLRLHPVVPLLLPRETMEDCVISGYEIPAKTRVIVNALS

IGSDPECWENPTQFQPERFLDGSIDFKGQNYELLPFGAGRRGCPGINFAI

LLIESVLANLLYCFDWKLPEGMQREDLDMEEGFGLTMHKKTPLCLIATSY

K

>PvCYP71A9

MMLSSILATCFIIFIFLMMNKMRKSRRLPPGPNRLPIIGNLHQLRDLPHR

WLHHLSQKYGPIMFLRLGSIPTLIISSADIAREIFKTHDRIFSNRPILYA

ASKLSYGCADLAFAPYGEYWREVRKIVILELLSLKRVQSFEVVRDEELRN

MLDAIAQSSVPFNLSQAALLLTNSVICRVVFGRKFDGEKELHGARKFQEM

LRETQDLLGGFSVADFFPWMDWLNKFNGFEARVDKNFRQIDELFDQVIEE

HLDPQRPEPEDKDLVDILLRLQKDQNQAFTFTNEQIKGVLVDMFIAGTDT

SAATLVWVMAELIKNPSVMKRAQDEVRRVVKSKGNVQEHHLSQLSYLKLV

VKEGSRLHPVVPLLVPRETMEDCVISGYEVPAKTRVFINALSIGMDPKCW

ENPAEFRPERFLDGSIDFNFKGQNYELLPFGVGRRVCPGINFAILLIELV

LANLLFSFDWKFPDGMGTADLNMEEAFGLTVHKKTPLCLIATSYSHNHQ

>PvCYP71B1

MALLPLLQQQWQELQATATPFHFCVLSFLLLLALKFFLNLSRGGKLKLPP

SPPKLPIIGHLHLIGDLPHRSVRALSEKYGPLMMLQLGTVPTLVVSSVET

AREVMKTHDIICSDRFQTRASNSIYLGSTDVAFATYGEYWRQVKKICVLE

LLTVKKVQSFHMVREEEVCELVEKIRQSSKTGASVNLSELLVGISNNIVS

KAVLGRKLEGGTGKDSIGELTRRAMELFGVFSFQDVFPILGWLDVLTGLT

SNLKRTSKKLHDFLGQVIEDHKNSKSNSDKMDFVDILLKHQRDNDLPVVL

SDDNVKAVLLDMIVGGTDTTAATLEWAMAELVKNPNMMKKARDEVKRVVG

KKGKVDEADCNQMKYIQCIIKETLRIYAVSPFLAPRKNTASIKLGGYDIP

QNTRIFVAAWAIQRDSKVWDKPEVFLPERFLSTSIDFKGQDFEFIPFGAG

RRICAGVTFSVAEAELILANLLYWFNWELPDGATAEGMDMTEAWGQVIHK

KTPLLLIPRLASSF

>PvCYP71B2

MALLPLLQQWWQELQATAPFNLFLLALPLLFVLVFFLNLPRGSKLNLPPS

PPKLPLIGNLHQLGILPHRSLETLSKKYGPLMLLQLGSAPTLVVSSAETA

KEIMKTHDIVFADRVQTKVSNSLYFGSSDVAFSPYGDYWRQVRKICVLEL

LSLKRVQSYQSVREEEVAELMENLRRSSKVGASVNLAELLVNLSNNIVSK

AALGRKFEVEAGEKSFGESARRVMELFGEFSFEDFFPSLAWLDSLTGLTS

SIKKTSNSLHSYLDQVIDEHKLTKTDGDLASGKKDFVEILLHLQKHGMLD

INLSQDNLKAIILDMFIGGTDTTSVTMEWAMAEVVRNPTILKKAQDEVRR

IVGKKARIEETDINQMNYLKCIIKEALRLYAVLPFLAPRESTATVKVGGY

DIPAKTRVFVNVWAIQRDPTLWDRPLEFIPERFDNNPIDYKGQDFQYLPF

GAGRRGCPGITFAMAQAEYVLANLLYWFDWELPGGARGEDMDMTEVYGLV

IHKKIPLEVVPLSPKPF

>PvCYP71B3

MALPLSLQQLWQELQRASLFNYCVLALAFFALLWVLKRIRGSKLKLPPSP

PKLPLIGNLHQLGTVPHRSLHTLSQKYGAVMLLQMGSAPTLVITSADSAR

DILKTHDIVFANRVQTKASKCIFGNNDVAFSPYGDYWRTVKKICVLELLS

LKRVQSFQVAREEEVAEMMDNIRRCGAKGVPADLGEMFVNISFDIVSRAV

LGRKFKKEDGKKSFGEAARDAMELFAAFAFEDLFPKLRWLDYFTGLVSRL

NKTKGDLNGYLDQVIKEHKISDIDEEKKDFVDILLHLQKDGKLDINLTQD

NLRAILTDMFIAGTDTTSTTLEWAMSELIKKPSAMKKAQEEVRRIIGKKS

KIEESDLGQMTYMKWVLKETLRLHAPVPFLAPRESSADVKVEGFDVPPKT

RVFVNVWAIQRDPKMWDKAEQFIPERFDNSPIDFKGQDFQYLPFGAGRRG

CPGITFGMAEAELVLANVLHWFDWKMPNGASGDDLDMEDVYGLVVHRKNN

LKLIPIARQSF

>PvCYP71B4

MALPLSLQQLWQELQRASLFNYCVLALAFFALLWVLKRIRGSKLKLPPSP

PKLPLIGNLHQLGTVPHRSLHTLSQKYGAVMLLQMGSAPTLVITSADSAR

DILKTHDIVFANRVQTKASKCIFGNNDVAFSPYGDYWRTVKKICVLELLS

LKRVQSFQVAREEEVAEMMDNIRRCGAKGVPADLGEMFVNISFDIVSRAV

LGRKFKKEDGEKSFGEAARDAMELFAAFAFEDLFPKLRWLDYFTGLVSRL

NKTKGDLNGYLDQVIKEHKISDIDEEKKDFVDILLHLQKDGKLDINLTQD

NLRAILTDMFIAGTDTTSTTLEWAMSELIKKPSAMKKAQEEVRRIIGKKS

KIEESDLGQMTYMKWVLKETLRLHAPVPFLAPRESSADVKVEGFDVPPKT

RVFVNVWAIQRDPKMWDKAEQFIPERFDNSPIDFKGQDFQYLPFGAGRRG

CPGITFGMAEAELVLANVLHWFDWKMPNGASGDDLDMEDVYGLVVHRKNN

LKLIPIARQSF

>PvCYP71B5

MALPLSLQQLWQELQRASLFNYCVLALAFFALLWVLKRIRGSKLKLPPSP

PKLPLIGNLHQLGTVPHRSLHTLSQKYGAVMLLQMGSAPTLVITSADSAR

DILKTHDIVFANRVQTKASKCIFGNNDVAFSPYGDYWRTVKKICVLELLS

LKRVQSFQVAREEEVAEMMDNIRRCGAKGVPADLGEMFVNISFDIVSRAV

LGRKFKKEDGEKSFGEAARDAMELFAAFAFEDLFPKLRWLDYFTGLVSRL

NKTKGDLNGYLDQVIKEHKISDIDEEKKDFVDILLHLQKDGKLDINLTQD

NLRAILTDMFIAGTDTTSTTLEWAMSELIKKPSAMKKAQEEVRRIIGKKS

KIEESDLGQMTYMKWVLKETLRLHAPVPFLAPRESSADVKVEGFDVPPKT

RVFVNVWAIQRDPKMWDKAEQFIPERFDNSPIDFKGQDFQYLPFGAGRRG

CPGITFGMAEAELVLANVLHWFDWKMPNGASGDDLDMEDVYGLVVHRKNN

LKLIPIARQSF

>PvCYP71B6

MALQPLLLLQQLWQELQKTSTFNFLFLPLVLLFSFLLLIKPLHRGDNLKL

PPSPPRLPIIGNLHQLGKQPHRSLRAISNKYGPLVLLHLGNAPTLLVSSA

HAAREIMKTHDVLFANKFQSKAADAFSYGCTDVVFSTYGEYWRQVRKICV

LELLSLKMVNSYQHVREEEVGELVDKIKTLCVDGASVNLSEMLVKISFDI

VSKVVIGRKYEVEDGKKSFGELVKKEVQLLAAFCFQDFFPSFGWLDVLTG

MTARVKETSEAIHAFLDQVFDEHKISNTSDDYQSDRKDFIQILLRLKKDG

MLQNLSQDNLKAILMDMFVGGTDTTSVTIEWAMAELAKNPAIMKKAQDEV

RRVVGKKSKVDETDLNQMDYLKCIVKETLRLYPPVPFLVPRESMENVKLE

GYDIPPRTRVFINAWAIQRNPELWDRPEEFFPERFTNNPIDYRGQDFQFI

PFGAGRRGCPGIAFGIIEIEHVLANLLYWFDWELPHGVTEKNLDMSDSYG

MVIHKKTPLKLVPVLHSF

>PvCYP71B7

MALQPLLLLQQLWQELQKTSTFNFLFLPLVLLFSFLLLIKPLHRGDKLKL

PPSPPRLPIIGNLHQLGKQPHRSLRAISNKYGPLVLLHLGNAPTLLVSSA

HAAREIMKTHDVLFANKFQSKAADAFSYGCTDVVFSTYGEYWRQVRKICV

LELLSLKMVNSYQHVREEEVGELVDKIKTLCVDGTSVNLSEMLVKISFDI

VSKVVIGRKYQVEDGKKSFGELVKKEVQLLAAFCFQDFFPSFGWLDVLTG

LTARVKETSEAIHAFLDQVFDEHKISNTSDDYQSDRKDFIQILLRLKKDG

MLQNLSQDNLKAILMDMFVGGTDTTSVTIEWAMAELAKNPAIMKKAQDEV

RRVAGKKSKVDETDLNQMDYLKCIVKETLRLYPPVPFLVPRESMENVKLE

GYDIPPRTRVFINAWAIQRNPELWDRPEEFFPERFTNNPIDYRGQDFQFI

PFGAGRRGCPGIAFGIIEIEHVLANLLYWFDWELPHGVTEKNLDMSDSYG

MVIHKKTPLKLVPVLHSF

>PvCYP71B8

MSLLSLLQQLWGDLQRITNFNLFILSFIFLFTLLLLFNFFPRASGGRGNL

NLPPSPPKLPLIGNLHQLGQLPHRSLRDLSEKYGPLMLLQLGKAPTLVVS

SPQFAREIMKTHDTVFANRVRIQAAEIFYNGCTNLSFSPYGEYWRQVRKI

CVLELLSLRRVQSFQLGREEVVAELIQSIRSSCTSRASVNLSELLHHTSS

CIASKAIIGRKFEDGKENFGALAKKETQLLGAFCFQDFFPSLGWLDGVTG

LTARFKETSKELNSFLDRVFDEHLNTKSSGNDQSDETNFVAILLQLEKDG

RSGNLTRDNLRSILVDIFLAATDTTGTTMGWAMAELMKNPATMKKAQEQV

RQVLGKKSKLEEVDINKIDYLKCIIKETLRLHPPVPLLVPRESSEKVTLE

GYDIPPKTRVFINVWAIQRDPKVWERPEEFLPERFEKNSIDFMDQDFQLI

PFGGGRRGCPGMTFGMVEMEHVLASLLYWFDWELPDGATAENLDMSELSG

VVIHKKTNLQLVPLLHSF

>PvCYP71B9

MLLQLGKAPTLVVSSPQFAREIMKTHDTVFANRVRIQAAEIFYNGCTSLS

FSPYGEYWRQVRKICVLELLSLRRVQSFQLGREEVVAELIQSIRSSCTSR

ASVNLSELLHHTSSCIASKAIIGRKFEDGKENFGALAKKETQLLGAFCFQ

DFFPSLGWLDGVTGLTARFKETSKELNSFLDRVFDEQLNTKSSGNDQSDE

TNFVAILLQLEKDGRSGNLTRDNLRSILVDIFLAATDTSGTTMGWAMAEL

MKNPATMKKAQEQVRQVLGKKSKLEEVDINKIDYLKCIIKETLRLHPPVP

LLVPRESSEKVTLEGYDIPPKTRVFINVWAIQRDPKVWERPEEFLPERFE

KNSIDFMDQDFQLVPFGGGRRGCPGMNFGMVEMEHVLASLLYWFDWELPD

DATAENLDMSELSGVVIHKKTNLQLVPLLHSF

>PvCYP71B10

MSLLSLLQQLWGDLQRITNFNLFILSFIFLFTLLLLFNFFPRASGGRGNL

NLPPSPPKLPLIGNLHQLGQLPHRSLRDLSEKYGPLMLLQLGKAPTLVVS

SPQFAREIMKTHDTVFANRVRIQAAEIFYNGCTNISFSPYGEYWRQVRKI

CVLELLSLRRVQSFQLGREEVVAELIQSIRSSCTSRASVNLSELLNHTSS

CIASKAIIGRKFEDGKENFGALAKKETQLLGAFCFQDFFPSLGWLDGVTG

LTARFKETSKELNSFLDRVFDEHLNTKSSGNDQSDETNFVAILLQLEKDG

RSGNLTRDNLRSILVDIFLAATDTTGTTMGWAMAELMKNPATMKKAQEQV

RQVLGKKSKLEEVDINKIDYLKCIIKETLRLHPPVPLLVPRESSEKVTLE

GYDIPPKTRVFINVWAIQRDPKVWERPEEFLPERFEKNSIDFMDQDFQLV

PFGGGRRGCPGMNFGMVEMEHVLASLLYWFDWELPDDATAENLDMSELSG

VVIHKKTNLQLVPLLHSF

>PvCYP71B11

MSLLSLLQQLWGDLQRITNFNLFILSFIFLFTLLLLFNFFPRASGGRGNL

NLPPSPPKLPLIGNLHQLGQLPHRSLRDLSEKYGPLMLLQLGKAPTLVVS

SPQFAREIMKTHDTVFANRVRIQAAEIFYNGCTNISFSPYGEYWRQVRKI

CVLELLSLRRVQSFQLGREEVVAELIQSIRSSCTSRASVNLSELLHHTSS

CIASKAIIGRKFEDGKENFGALAKKETQLLGAFCFQDFFPSLGWLDGVTG

LTARFKETSKELNSFLDRVFDEHLNTKSSGNDQSDETNFVAILLQLEKDG

RSGNLTRDNLRSILVDIFLAATDTTGTTMGWAMAELMKNPATMKKAQEQV

RQVLGKKSKLEEVDINKIDYLKCIIKETLRLHPPVPLLVPRESSEKVTLE

GYDIPPKTRVFINVWAIQRDPKVWERPEEFLPERFEKNSIDFMDQDFQLV

PFGGGRRGCPGMNFGMVEMEHVLASLLYWFDWELPDDATAENLDMSELSG

VVIHKKTNLQLVPLLHSF

>PvCYP71B12

MALLPLLQQQWQELQATATPFHFCVLSFLLLLALKFFLNLSRGGKLKLPP

SPPKLPIIGHLHLIGDLPHRSVRALSEKYGPLMMLQLGTVPTLVVSSVET

AREVMKTHDIICSDRFQTRASNSIYLGSTDVAFATYGEYWRQVKKICVLE

LLTVKKVQSFHMVREEEVCELVEKIRQSSKTGASVNLSELLVGISNNIVS

KAVLGRKLEGGTGKDSIGELTRRAMELFGVFSFQDVFPILGWLDVLTGLT

SNLKRTSKKLHDFLGQDMIVGGTDTTAATLEWAMAELVKNPNMMKKARDE

VKRVVGKKGKVDEADCNQMKYIQCIIKETLRIYAVSPFLAPRKNTASIKL

GGYDIPQNTRIFVAAWAIQRDSKVWDKPEVFLPERFLSTSIDFKGQDFEF

IPFGAGRRICAGVTFSVAEAELILANLLYWFNWELPDGATAEGMDMTEAW

GQVIHKKTPLLLIPRLASSF

>PvCYP71B13

MALLPLLQQQWQELQATATPFHFCVLSFLLLLALKFFLNLSRGGKLKLPP

SPPKLPIIGHLHLIGDLPHRSVRALSEKYGPLMMLQLGTVPTLVVSSVET

AREVMKTHDIICSDRFQTRASNSIYLGSTDVAFATYGEYWRQVKKICVLE

LLTVKKVQSFHMVREEEVCELVEKIRQSSKTGASVNLSELLVGISNNIVS

KAVLGRKLEGGTGKDSIGELTRRAMELFGVFSFQDVFPILGWLDVLTGLT

SNLKRTSKKLHDFLGQVIEDHKDSKSNSDKMDFVDILLKHQRDNDLPVVL

SDDNVKAVLLDMIVGGTDTTAATLEWAMAELVKNPNMMKKARDEVKRVVG

KKGKVDEADCNQMKYIQCIIKETLRIYAVSPFLAPRKNTASIKLGGYDIP

QNTRIFVAAWAIQRDSKVWDKPEVFLPERFLSTSIDFKGQDFEFIPFGAG

RRICAGVTFSVAEAELILANLLYWFNWELPDGATAEGMDMTEAWGQVIHK

KTPLLLIPRLASSF

>PvCYP71D7

MEFQFPGFQTLFSFFLCIFMVLKTVKRRSKSNMSTPNLPPGPFKLPLIGN

IHQLVGGIPHRALRDVAKKYGPLMHLKLGEVSTIVVSSAETAQEVMKTHD

ITFASRPHILVTKILSYDSADLAFAPYGDYWRQMRKICVLELLSMKRVQS

FRAIREEEVSNLMESISCKVGSPVNLSELIFPMTYSITSRAAFGKKNNDQ

ETFLVAVKEIVDLASGFSVSDLFPSIDLLHALSGARPRLEKVHKEIDRMF

ESIICDHKARRLAATSDGEGAAEDLVDVLLNVQEEDGDLQSHISTKNIKA

VILDIFFAGSETSSTVVDWAMVEMVKNPRVMKKAQAEVRALFDGKRKIDE

TEIQGLEYLKLVIKETMRLHPPFPLLLPRECREKCEIHGYDIPLKTKVII

NAWAIGRDPKYWPEPESFNPERFLDSSIGYKGLDFEYLPFGAGRRICPGV

AFGMMNVELPLAHFLYHFDWKLPNGTKPEDLDMTEAFGASVRRKQPLQLV

PIAYRPI

>PvCYP71D9

MELQLPSFPLLLAFILFVFIIAKKLNRPKAKNSPPKLPPGPWKLPLIGNM

HQLVGSLPHHSLRGLAMEYGPLMHLQLGQVPTIIVSSPQVAQEVMKTHDI

IFSDRPYLLASWIMNYNSTDIVFSPYGNYWRHLRKICVIELLTLKRVQSF

RMIREEEVSNLIRTISLNARSTVNLSEKIFSLTYGITSRAAFGEKSKDKE

AFKSVMKEAVELAGGFSVADMYPSVKWLALISGMGPKLEKLHQEADRIVQ

NIVNEHRENLATTRMDKEEAEEDLVDVLLRVQNSGELEFPLTDDNIKATI

TNIFSAGSETSSTAVEWAMSEMLKNPRVLKKAQAEVRKVFGSKGSVDETS

LHELNYLKSVIQETLRLHPSAPLLVPRECRERCEINGYEIPAKAKVIVNA

WAIGRDPDYWTEAERFYPERFIDSPIDYNGTDFKYIPFGAGRRICPGIAF

ALANIELPLAQLLYHFDWRIPDGVKNEELDMTEAFGVTVRRAKDLHLISI

PYHPFPSE

>PvCYP72A1

MEITILTYASSFGLVVILTWLLLVVVNWLWLKPKKLERCLRKQGLTGNSY

TLFYGDLKESSMMLREATSKPIKLSHDIAPRVNPFIHLTIQKYGKNSFVW

NGPTPRVVIMDPELIKDIFTKYRHFQKLVTNPLNKLLATGVVNYEGDKWA

KHRKIINPAFHLEKLKKMQPAFYQCVSDLMSKWEEMVSVEGSCELDVWPF

FETLAGDVISRSAFGSNFEEGRKIFHLQRQQAHLIIENSQSVYIPGMRFL

PTKINKKMKNIEKEVQSLLRGIINRKEKAMQAGEAANDDLLGILMDSNFK

EIQQHENDKNVGMSIRDVIEECKLFYFAGQETTSVLLSWTMVLLSKYSDW

QEHAREEVLHVFGKNKPHIEGLNHLKIVTMILYEVLRLYPPGHLLTRSLY

EDTKLGKLSLPKGVEVLLPAILVHQDHEFWGEDANEFKPERFSGGVSKAA

KNQASFFPFGGGPRICVGQNFAMIEAKLALAMILQRFSVQLSPSYAHAPV

DVITVQPQYGVHLILNKA

>PvCYP72A2

MEIPLAKWIPLVGILAWVFMRVVNWLWLKPKKLEKCLRQQGFTGNSYTLF

YGDLKEHSIMLSEATSKPIKLNHDISPRVYPLIHHTIQKYGKNSFIWGGP

MPRVIIMNPELIRDVFTKHGDFHRPLTNPLINLLITGVIDYEGDKWVKHR

KIVNSAFHLEKLKKMQPAFYQSCNDLMRKWEKMVTSEGSCELDVWPFFET

LTGDVISRTAFGSSYEEGTKIFHLQREQVPLIIQYSQSVYIPGMRFLPTK

MNKRMKEIDEEVQALLMGIVNKREKAMKAGEVPNDDLLGILMDFNFKEIQ

KHGNDKNIGLSTQEVIEECKLFYFAGQETTSVLLSWTMVMLSTYPDWQVR

AREEVFQVFENRKPDIEGLNHLKIVTMILYEVLRLYPPGPLLTRALFEET

KLGKLSLPGGIHLLIPTVLVHRDHDLWGEDANEFKPERFSEGVSKATKNQ

ASFFPFGGGPRICVGQNFAMIEVKMTLAMILQRFSFQLSPSYAHAPVDAI

TLQPQYGVHLILQKK

>PvCYP72A4

MEIPLAKWIPLVGILAWVFMRVVNWLWLKPKKLEKCLRQQGFTGNSYTLF

YGDLKEHSIMLSEATSKPIKLNHDISPRVYPLIHHTIQKYGKNSFIWGGP

MPRVIIMNPELIRDVFTKHGDFHRPLTNPLINLLITGVIDYEGDKWVKHR

KIVNSAFHLEKLKKMQPAFYQSCNDLMRKWEKMVTSEGSCELDVWPFFET

LTGDVISRTAFGSSYEEGTKIFHLQREQVPLIIQYSQSVYIPGMRFLPTK

MNKRMKEIDEEVQALLMGIVNKREKAMKAGEVPNDDLLGILMDFNFKEIQ

KHGNDKNIGLSTQEVIEECKLFYFAGQETTSVLLSWTMVMLSTYPDWQVR

AREEVFQVFENRKPDIEGLNHLKIVTMILYEVLRLYPPGPLLTRALFEET

KLGKLSLPGGIHLLIPTVLVHRDHDLWGEDANEFKPERFSEGVSKATKNQ

ASFFPFGGGPRICVGQNFAMIEVKMALAMILQRFSFQLSPSYAHAPVDAI

TLQPQYGVHLILQKK

>PvCYP72A5

MEIPLAKWIPLVGILAWVFMRVVNWLWLKPKKLEKCLRQQGFTGNSYTLF

YGDLKEHSIMLSEATSKPIKLNHDISPRVYPLIHHTIQKYGKNSFIWGGP

MPRVIIMNPELIRDVFTKHGDFHRPLTNPLINLLITGVIDYEGDKWVKHR

KIVNSAFHLEKLKKMQPAFYQSCNDLMRKWEKMVTSEGSCELDVWPFFET

LTGDVISRTAFGSSYEEGTKIFHLQREQVPLIIQYSQSVYIPGMRFLPTK

MNKRMKEIDEEVQALLMGIVNKREKAMKAGEVPNDDLLGILMDFNFKEIQ

KHGNDKNIGLSTQEVIEECKLFYFAGQETTSVLLSWTMVMLSTYPDWQVR

AREEVFQVFENRKPDIEGLNHLKIVTMILYEVLRLYPPGPLLTRALFEET

KLGKLSLPGGIHLLIPTVLVHRDHDLWGEDANEFKPERFSEGVSKATKNQ

ASFFPFGGGPRICVGQNFAMIEVKMALAMILQRFSFQLSPSYAHAPVDAI

TLQPQYGVHLILQKK

>PvCYP72A6

MEIPLAKWIPLVGILAWVFMRVVNWLWLKPKKLEKCLRQQGFTGNSYTLF

YGDLKEHSIMLSEATSKPIKLNHDISPRVYPLIHHTIQKYGKNSFIWGGP

MPRVIIMNPELIRDVFTKHGDFHRPLTNPLINLLITGVIDYEGDKWVKHR

KIVNSAFHLEKLKKMQPAFYQSCNDLMRKWEKMVTSEGSCELDVWPFFET

LTGDVISRTAFGSSYEEGTKIFHLQREQVPLIIQYSQSVYIPGMRFLPTK

MNKRMKEIDEEVQALLMGIVNKREKAMKAGEVPNDDLLGILMDFNFKEIQ

KHGNDKNIGLSTQEVIEECKLFYFAGQETTSVLLSWTMVMLSTYPDWQVR

AREEVFQVFENRKPDIEGLNHLKIVTMILYEVLRLYPPGPLLTRALFEET

KLGKLSLPGGIHLLIPTVLVHRDHDLWGEDANEFKPERFSEGVSKATKNQ

ASFFPFGGGPRICVGQNFAMIEVKMTLAMILQRFSFQLSPSYAHAPVDAI

TLQPQYGVHLILQKK

>PvCYP73A4

MFNFTPPQLYTSTAMDLFLLEKALLGLFIAMITAITVSKLRGKRFKLPPG

PLPVPIFGNWLQVGDDLNHRNLADFAKKFGEVLLLRMGQRNLVVVSSPDL

AKDVLHTQGVEFGSRTRNVVFDIFTGKGQDMVFTVYGEHWRKMRRIMTVP

FFTNKVVQQYRYGWEDEAARVVEDVKKNPEAARGGIVLRKRLQLMMYNNM

YRIMFDRRFENEEDPLFVKLKALNGERSRLAQSFDYNYGDFIPILRPLLR

GYLKICKEVKERRFELFKDFFVEERKKLASVTNNGLKCAIDHIMEAEQKG

EINEDNVLYIVENINVAAIETTLWSIEWGIAELVNHPEIQKKLRNEIDTV

LGPGVQVTEPDTHKLPYLQAVIKETLRLRMAIPLLVPHMNINDAKLGGFD

IPAESKVLVNAWWLANNPAHWKNPEEFRPERFLEEESKVEANGNDFRYLP

FGVGRRSCPGIILALPILGITIGRLVQNFELLPPPGRAKLDTTEKGGQFS

LHILKHSTIVAKPRSF

>PvCYP73A5

MDLFLLEKALLGLFIAMITAITVSKLRGKRFKLPPGPLPVPIFGNWLQVG

DDLNHRNLADFAKKFGEVLLLRMGQRNLVVVSSPDLAKDVLHTQGVEFGS

RTRNVVFDIFTGKGQDMVFTVYGEHWRKMRRIMTVPFFTNKVVQQYRYGW

EDEAARVVEDVKKNPEAARGGIVLRKRLQLMMYNNMYRIMFDRRFENEED

PLFVKLKALNGERSRLAQSFDYNYGDFIPILRPLLRGYLKICKEVKERRF

ELFKDFFVEERKKLASVTNNGLKCAIDHIMEAEQKGEINEDNVLYIVENI

NVAAIETTLWSIEWGIAELVNHPEIQKKLRNEIDTVLGPGVQVTEPDTHK

LPYLQAVIKETLRLRMAIPLLVPHMNINDAKLGGFDIPAESKVLVNAWWL

ANNPAHWKNPEEFRPERFLEEESKVEANGNDFRYLPFGVGRRSCPGIILA

LPILGITIGRLVQNFELLPPPGQAKLDTTEKGGQFSLHILKHSTIVAKPR

SF

>PvCYP74A1

MASSTLTFTPLKLHQQPSSQTQRRRSTYPSTRRFIARPISASVSERPPVP

VVPVTPPTPTTLPIRKIPGNYGVPLIGPLKDRLDYFYNQGRDEFFKSKIQ

QYRSTVFRANMPPGPFISKNPNVIVLLDGKSFPILFDVTKVEKKNLFTGT

YMPSTELTGGYRMLSYLDPSEPNHSKLKKLMFFLLKSSRDKVMPEFHSSY

TELFETLESELAAKGKANFGDANDQAAFTFLARSLFGVNPADTKLGLDAP

KLIQKWVLFQLSPILTLGLPKFLEEATIHTFPLPPALVKKDYQRLYEFFY

DSSSSILEEAVRLGVSKEEACHNLLFATCFNSFGGMKILFPNMIKLIGRA

GAQLHTRLAQEIRSVVKSCGGKITMAGMEQMPLMKSVVYESYRMEPPVAL

QYGRAKRDIVVESHDSAFEVKEGELMFGFQPFATKDPKIFERAEEFVPDR

FVGEGEKLLKHVLWSNGPETESPTVGNKQCAGKDFVVLVSRLLVVELFLR

YDSFEIEVGKSPLGSAVTVTSLKRASF

>PvCYP74B2

MMTNMMSTLPGTPSTSRSPPVASPSTLPVRTVPGSYGLPLLGPISDRLDY

FWFSGQEKFFRNRIEKHKSTVFRTNVPPTFPLFTSVNPKVVAVLDCKSFA

HMFDMDIVEKKNVLVGDFMPSVSFTGDIRVCAYLDPTEPAHSKVKNFAMD

ILKRSSTVWVTELLSNLDTMWDTLELDLSNSSNSSASYIFPLQQFIFQWL

IKSLVGADPATSPEIAKSGYAMLDKWLALQLLPTVAIGILQPFEEIFLHS

FAYPFALVSGDYKKLESFVEKEGQEVIQRGESEFQLSKEETIHNLLFILG

FNAFGGFSVFLPTLMSTIGSDTKLQERLREEVRSKQGESTLSFDSVSQME

LVQSVVYETLRLNPPVPLQYGRARKDFLLSSHDSAYDIKKGELLCGYQKL

VMRDPKVFYDPETFVPDRFTGEKGRELLNYLYWSNGPQTGTPSASNKQCA

AKDYVTLSACLFVAYMFRRYDSITCSSSSITAFEKAT

>PvCYP75A1

MESLFSWSVLVIACFALLTLVSKILSRSRYNQKQYHPPGPKPWPIIGNLN

LMGSLPHHALDKLAQTYGPIMQLKFGSFPVVVASSGEMASQFLKTHDHIF

ASRPQTAAGKYTTYNYSNITWSPYGPYWRQGRKLFLTELFSSKRLESYEY

IRVEELQAFVSRLYASCGKPVVLKHHILHFTLSIISRIVLGKKYVSESQS

KNLKMTPEEFQEMLDELFVLNGVLNIGDWVPWLEFLDLQGYVKRMKALCK

KFDRFLDNVFDEHKARRKSEKDDFVAKDMVDVLLQLADDPNLEVTLTNDG

VKGFTLDLIAGGTETSVTSIEWAMSELLKQPHIIKKARQEMDGVIGRERW

VNEKDIPRLPYLDAIVKETMRLHPAVVLIVPHLALQDCNVAGYNIRKGTR

VFINAWSLGRDPTVWKAPLEFRPERFLGKAIDVKGQNFELLPFGSGRRMC

PGYNLGLKMIQMSLANMIHGFDWKLPDNMKVEDLSMEEVYGLTTPRKFPL

VAVMEARLPLHLY

>PvCYP75A2

MESLTWATYAVAWLATVSLLLLSTHLRRKKLLLPPGPKPWPIIGNLNLIG

PLPHRSIHALAQKYGPIMQLQFGSFPVMVGSSVDMAKAILKTHDLIFASR

PKTAAGKYTTYNYSDITWSQYGSYWRQARKMCLVELFSAKRLESYEYIRV

EELKAILKDMCSSAGKKIVLKDYLSTLSLNVISRMVLGKKYTDETEDSIV

TPEEFKKMLDELFLLSGVLNIGDSIPWIDFLDLQGYVKRMKALSKKLDRF

LEHVLDEHNDKRNRVKDYVAKDMVDVLLQLADDPDLEVKLERSGVKAFTQ

DLIAGGTESSAVTVEWAMSEILRKPEVFTKATEELDQVIGRERWVEEKDI

PNLPYIEAIVKETMRMHPVAPMLVPRMAREDIQVAGYDIAKGTRVLVNTW

TILRDPELWDKPDEFCPDRFVGKAMDVKGQDFELLPFGSGRRMCPGYSLG

LKVIQASLANLLHGFVWKLAGDVKREELNMEEIFGLSTPKKFPLEVVIEP

RLPLHVYGL

>PvCYP75A3

MESLTWATYAVAWLATVSLLLLSTHLRRKKLLLPPGPKPWPIIGNLNLIG

PLPHRSIHALAQKYGPIMQLQFGSFPVMVGSSVDMAKAILKTHDLIFASR

PKTAAGKYTTYNYSDITWSQYGSYWRQARKMCLVELFSAKRLESYEYIRV

EELKAILKDMCSSAGKKIVLKDYLSTLSLNVISRMVLGKKYTDETEDSIV

TPEEFKKMLDELFLLSGVLNIGDSIPWIDFLDLQGYVKRMKALSKKLDRF

LEHVLDEHNDKRNRVKDYVAKDMVDVLLQLADDPDLEVKLERSGVKAFTQ

DLIAGGTESSAVTVEWAMSEILRKPEVFTKATEELDQVIGRERWVEEKDI

PNLPYIEAIVKETMRMHPVAPMLVPRMAREDIQVAGYDIAKGTRVLVNTW

TILRDPELWDKPDEFCPDRFVGKAMDVKGQDFELLPFGSGRRMCPGYSLG

LKVIQASLANLLHGFVWKLAGDVKREELNMEEIFGLSTPKKLPLEVVIEP

RLPLHVYGL

>PvCYP75B1

LHSHTKQPPLLSFIERMTPVTFLLSTLAVICFVYLFFSLVNSHPRRLPPG

PRPWPIIGNLPHLGPKPHQSLASLARSYGPLMHLRLGFVDVVVAASASVA

AQFLKTNDANFVNRPPNSGAKYIAYNYQDLVFAPYGPRWRLLRKVSSLHL

FSGKALDDFRHLRQEEVAVLVHALASASNSLVNLGQLLNVCTTNALARVM

LGKRVFGDGSGGVDPKADEFKDMVVEVMVLAGVFNLGDFVPALERFDLQG

VATKMKNLHARFDSFLGNILKEHKMNSDGVKQQNDFLSKLISLKDDVDGE

GGKLTDIEIKALLLNMFTAGTDTSSSTVEWGIAELIRHPKILAQAQQEID

SVVGRDRLVTELDLPNLPFLQAVVKETFRLHPSTPLSLPRMASQSCEING

YYIPKGSTLLVNVWAIARDPNVWAEPLEFRPDRFLPGGEKPNIDIKGNDF

EVIPFGAGRRICAGMSLGLRMVQLLTATLVHAFDWGLPEGQIPEKLQMEE

AYGLTLQRAVPLVLYPQPRLSSHVY

>PvCYP75B2

MHSHTKQPPLLSFIERMTPVTFLLSTLAVICFVYLFFSLVNSHPRRLPPG

PRPWPIIGNLPHLGPKPHQSLASLARSYGPLMHLRLGFVDVVVAASASVA

AQFLKTNDANFVNRPPNSGAKYIAYNYQDLVFAPYGPRWRLLRKVSSLHL

FSGKALDDFRHLRQEEVAVLVHALASASNSLVNLGQLLNVCTTNALARVM

LGKRVFGDGSGGVDPKADEFKDMVVEVMVLAGVFNLGDFVPALERFDLQG

VATKMKNLHARFDSFLGNILKEHKMNSDGVKQQNDFLSKLISLKDDVDGE

GGKLTDIEIKALLLNMFTAGTDTSSSTVEWGIAELIRHPKILAQAQQEID

SVVGRDRLVTELDLPNLPFLQAVVKETFRLHPSTPLSLPRMASQSCEING

YYIPKGSTLLVNVWAIARDPNVWAEPLEFRPDRFLPGGEKPNIDIKGNDF

EVIPFGAGRRICAGMSLGLRMVQLLTATLVHAFDWGLPEGQIPEKLQMEE

AYGLTLQRAVPLVLYPQPRLSSHVY

>PvCYP75B3

MTPVTFLLSTLAVICFVYLFFSLVNSHPRRLPPGPRPWPIIGNLPHLGPK

PHQSLASLARSYGPLMHLRLGFVDVVVAASASVAAQFLKTNDANFVNRPP

NSGAKYIAYNYQDLVFAPYGPRWRLLRKVSSLHLFSGKALDDFRHLRQEE

VAVLVHALASASNSLVNLGQLLNVCTTNALARVMLGKRVFGDGSGGVDPK

ADEFKDMVVEVMVLAGVFNLGDFVPALERFDLQGVATKMKNLHARFDSFL

GNILKEHKMNSDGVKQQNDFLSKLISLKDDVDGEGGKLTDIEIKALLLNM

FTAGTDTSSSTVEWGIAELIRHPKILAQAQQEIDSVVGRDRLVTELDLPN

LPFLQAVVKETFRLHPSTPLSLPRMASQSCEINGYYIPKGSTLLVNVWAI

ARDPNVWAEPLEFRPDRFLPGGEKPNIDIKGNDFEVIPFGAGRRICAGMS

LGLRMVQLLTATLVHAFDWGLPEGQIPEKLQMEEAYGLTLQRAVPLVLYP

QPRLSSHVY

>PvCYP76A1

MFSLGWSSLVLGGFLLLAALVVVVSKRENKQRPPGPPGWPVIGNLFDLGT

MPHQSLYHLRTKYGPVLWLRLGSMNTMVIQSPEAAAQLFKNHDLNFSDRK

CPDVLKAHDYFRGSLAFGGYGPYWRVLRRLCSSEMLVTKRINEVAPLRQK

CIDAMIGWIEEDAREKESGEVNLSHFLFLMAFNVLGNLMLSRDLLGLNSK

EGHDFYNATNGVMEGVGTPNLADFLPWLRWLDLQGVHRRMMHDMGQAMSL

VGGFVKERVQGQQFGQEKERKDFLDALLEFEGDAKEWPHKITDRNTNIII

LEMFFAGSDTTSSTIEWGMTELLRQPEIMKRVKEELERVIGPNKKVEESD

MDKLSYLQAVVKETLRLHPPVPLLLPRNALQDVNFMGFDIPKDTQVFVNA

WAIGRDPDSWSDPLSFKPERFFDSNVDYKGQNFELLPFGSGRRICVGMML

AHRLVHLSLASLLHTFHWDLPNNITKETIDMNERMGVTLRKLVPLKAIPK

KRIIS

>PvCYP76A2

MVIPLSSLMWPILIFLVALLLLLRKRNPKNNSQLPPGPPGWPVIGNMLNL

GTMPHQSLYHLVPKYGPVIWLKLGSINTMIIQSPKAATQLFKTHDLNFSD

RKCPGALKARDYNEGSLAVGRYGAYWRMLRRLCSMELLVTKRINDVAPLR

RKCTDDMIRSIDEEATAARLRGESGEVNLAHFLFLMAFNVVGNLMVSRDL

LDANSKEGHDFYHAMNGIMEGAGKPNVADFLPFLKWFDPQGIQRQMTRDM

GRAMSIVSGFVKERVGSQQVGEEKTRRDFLDALLEFEGDGKEWPHKMTER

NTNIIILEMFFAGSETTSSTIEWGMAELLRNPELIKNVKEELERVVGPNR

KVEESDIDDLPYLQAVVKETLRLHPAIPLLLPRNALQDANFMGYVIPKDT

QVFVNAWAIGRDSDSWDDPLSFKPERFLDSDIDYKGQNFELLPFGSGRRV

CVGISLAHRVVHLGLASLLHTFDWKLPDNITPETIDMNERMGITLRKLVP

LKAIPNKRIIW

>PvCYP76A3

MFSLDWTSLVWGGFLLAALVVMVSKRENKQRPPGPPGWPVIGNLFDLGTM

PHQSLYHLRSKYGPVLWLKFGSMNTMVIQSPKAAAQLFKNHDLNFSDRKS

PGVLKAHDYFQGSLAFGGYGPYWRVLRRLCSSEMLVTKRINEIAPLRQKC

IDAMIGWIEKDSREKESGEVNLPDFLFPMTFNVVGNLMLSRDLLDLNSKE

GHDFYNAMGGVMEGAGTPNLADFFPWLRWLDLQGLHRGMVRDMGQAMSIV

GGFVKERVQDQQFGQEKERKDFLDALLEFEGDAKEWPHKITDRNTNIIIL

EMFFAGSETTSSTIEWGMAEIIRKPEIMKKVKEELERVIIPNKKVEESDI

EKLPYLQAVVKETLRLHPALPLMLPRNALQDVNFMGFDIPKDTQVFVNTW

AIGRDPDSWDDPLSFKPERFLDSNVEYKGQNFEFLPFGSGRRICVGMVLA

DRVVHLALASLLHTFDWELPNNITQETLDMNERMGITLRKLVPLKAIPKK

RIIS

>PvCYP76F14

MDFLSCILCLLLAWSLAQALLSTLRRSKTCHSKLPPGPFPFPVIGNLLKL

GDKPHRSLAELAKTHGPIMRLNLGHVTTIVISSATVAREVLQKDDLSFSN

RFIPDSIRAPKHDQHSVAWLPVSTPWRNLRRICNSHIFTTPKLDSNQHLR

RHKVADLLKDVEQSCQSGKAVDIGGAAFKTTLNLLSNTIFSVDLADSSSD

TAREFKEVVGNIMEDVGKPNFVDYFPALRKMDPQGIRRRMAHHFEKMFGL

FDGMIKPRLQQRNEDGYVTSNDVMDILLSIIEDKAEKVDRADIDHLLFDL

FAAGTDTSSSTLEWAMAELLHNPETLTKAKLELEQTIGKGNRFVESDISR

LPYLRAVVKETFRLHPAVPLLLPRTVEADTELCGFIVPKGAQVLVNAWAI

GRDPTTWVNPNSFVPERFLDSDMDVKGQDFELIPFGGGRRICPGLPLAIR

MIHLMLGSLVHSFDWKLEDGVTPENMGMEDKFGIAVQKARPLRAVPSV

>PvCYP76T1

MDYILVSVFLISVWTCIKFVSNRRKSKLPPGPRPFPIIGNLFELGNKPHH

SLTKLSKTYGPIITLQTGSITTIVISSSHTAKQVLQKHDQSLSGRTVPDT

MHAMNHHQFSIVFLPPAHQWRKLRKICNSQIFTVQRLDAGQALRYKKVQQ

LVDYVLECCAKGEAVDVNQVIFIATLNLISNSIFSTDLAHYGSNTSQEFK

DPIWGILEETGKPNIADFFPVFKLLDPQGVRRRVAGHYKKLMEIFDGIIT

QRLKLQNSSAGSGSCDMLDAALNLTNENDHQLSCNDLKHLLVDLFVAGSD

TSSSTMEWAMAELLRNPKAMVKVQAEVREVVGKDRAVEESDMARLPYLQA

VVKETFRMHPTAPFLVPHKAEEEVEIDGFTVPKGAQILVNAWAIGRDPAT

WADPNTFIPERFLDKTKSTIDFKGRDFELIPFGAGRRICPGLPLAHRMVH

LVLASLLHSFDWKLQNGMKPQDMDMSDKFGFTLPKAEPLLAIPIKV

>PvCYP76T2

MDYMLLILAIILASVFIHIFSSSTSNHLKSKLPPGPQPSPVIGNLLELGS

QPHRSLFKLSKTYGPLITLKLGKITTIVVSSSDSAKQIFQHNDLSFSSRT

IPDSVGGLNQSANSMVWLPVSDRWRSLRKICALQLFTTQRLDAGQSLRME

KIQELVNYVKESCESGEAVDIGRAAFTTTLNLISNSFFSMDLANHHSSSS

QEFKDLVFGVMEEIGRPNIADYFPALRFIDPQGIRRGVENYFKKLFKLFD

SIIDQRKQSPSSTPKNDFLEALLNATEQADHDLSCYDIKHLLLDMFLAGT

DTSSSTVEWAMVELLRNPTKLTKAQIELQQVIGKNKSVQESDISKLPFLQ

AIVKETFRLHPVAPFLIPHKANTEVEINGFSVPKDAQIWVNVWGIGRDST

IWAEPESFLPERFLDCKIDFKGHDFELIPFGAGRRICPGLPLGHRMVHLM

LVSLLHSFEWKLEAGMKPEDVDMGEKFGLTLHKAVPLRAIPIKL

>PvCYP76T3

MDYILVSVFLISVWTCIKFVSNRRKSKLPPGPRPFPIIGNLFELGNKPHH

SLTKLSKTYGPIITLQTGSITTIVISSSHTAKQVLQKHDQSLSGRTVPDT

MHAMNHHQFSIVFLPPAHQWRKLRKICNSQIFTVQRLDAGQALRYKKVQQ

LVDYVLECCAKGEAVDVNQVTFIATLNLISNSIFSTDLAHYGSNTSQEFK

DPIWGILEETGKPNIADFFPVFKLLDPQGVRRRVAGHYKKLMEIFDGIIT

QRLKLQNSSAGSGSCDMLDAALNLTNENDHQLSCNDLKHLLVDLFVAGSD

TSSSTMEWAMAELLRNPKAMVKVQAEVREVVGKDRAVEESDMARLPYLQA

VVKETFRMHPTAPFLVPHKAEEEVEIDGFTVPKGAQILVNAWAIGRDPAT

WADPNTFIPERFLDKTKSTIDFKGQDFELIPFGAGRRICPGLPLAHRMVH

LVLASLLHSFDWKLQNGMKPQDMDMSDKFGFTLPKAEPLLAIPIKV

>PvCYP76T4

MDYLLVSLFVIFVVWIWIPTVRWKSKLPPGPHPFPIIGNLLELGNKPHHS

LTKLSKIYGPIITLQTGNITTIVVSSSHTAQQVLKKHDQSLSSRTIPDAM

NAINHHQFSIVFLPASAQWRKLRKICNSHIFTMQRLDGGEALRHKKMQEL

MDHVRDSCTSGEAIDIGRVAFTTTLNLISNSIFSTDLAHYNSNLTQEFKD

LVWSVLEETATPNVADFFPFLRLLDPQGVRRRVTISCNKLMEIFDGIISQ

RSKLRSSSLAATNNRDMLDAALDLTELSYNELKHLLFDLFVAGSDTTSGT

IEWVMAELLHNPITMAKVQAEVRRVISMDKSAQESDITNLPYLQAVVKET

LRLHPPVPLLVPHKAETDVEINGFVVPKDAQVLVNVWAIGRDPSLWADPN

SFLPERFSKQKIDFKGQDFELIPFGAGRRLCPGLPLANRMVHLVLASLLH

SFDWKLENGIKAEEMDMSDKFGFTLPKAEPLRAIPIKVGRS

>PvCYP76T5

MDYLLVSLFVIFVVWIWIPTVRWKSKLPPGPHPFPIIGNLLELGNKPHHS

LTKLSKIYGPIITLQTGNITTIVVSSSHTAQQVLKKHDQSLSSRTIPDAM

NAINHHQFSIVFLPASAQWRKLRKICNSHIFTMQRLDGGEALRHKKMQEL

MDHVRDSCTSGEAIDIGRVAFTTTLNLISNSIFSTDLAHYNSNLTQEFKD

LVWSVLEETATPNVADFFPFLRLLDPQGVRRRVTISCNKLMEIFDGIISQ

RSKLRSSSLAATNNRDMLDAALDLTELSYNELKHLLFDLFVAGSDTTSGT

IEWVMAELLHNPITMAKVQAEVRRVISMDKSAQESDITNLPYLQAVVKET

LRLHPPVPLLVPHKAETDVEINGFVVPKDAQILVNVWAIGRDPSLWADPN

SFLPERFSKQKIDFKGQDFELIPFGAGRRLCPGLPLANRMVHLVLASLLH

SFDWKLENGIKAEEMDMSDKFGFTLPKAEPLRAIPIKVGRS

>PvCYP76T6

MNAINHHQFSIVFLPASAQWRKLRKICNSHIFTMQRLDGGEALRYKKMQE

LMDHVRDSCTSGEAIDIGRVAFTTTLNLISNSIFSTDLAHYNSNLTQEFK

DLVWSVLEETATPNVADFFPFLRLLDPQGVRRRVTISCNKLMEIFDGIIS

QRSKLRSSSLAATNNRDMLDAALDLTELSYNELKHLLFDLFVAGSDTTSG

TIEWVMAELLHNPITMAKVQAEVRRVISMDKSAQESDITNLPYLQAVVKE

TLRLHPPVPLLVPHKAETDVEINGFVVPKDAQILVNVWAIGRDPSLWADP

NSFLPERFSKQKIDFKGQDFELIPFGAGRRLCPGLPLANRMVHLVLASLL

HSFDWKLENGIKAEEMDMSDKFGFTLPKAEPLRAIPIKVGRS

>PvCYP76T7

MDYLLVSLFVIFVVWIWIPTVRWKSKLPPGPHPFPIIGNLLELGNKPHHS

LTKLSKIYGPIITLQTGNITTIVVSSSHTAQQVLKKHDQSLSSRTIPDAM

NAINHHQFSIVFLPASAQWRKLRKICNSHIFTMQRLDGGEALRHKKMQEL

MDHVRDSCTSGEAIDIGRVAFTTTLNLISNSIFSTDLAHYNSNLTQEFKD

LVWSVLEETATPNVADFFPFLRLLDPQGVRRRVTISCNKLMEIFDGIISQ

RSKLRSSSLAATNNRDMLDAALDLTELSYNELKHLLFDLFVAGSDTTSGT

IEWVMAELLHNPITMAKVQAEVRRVISMDKSAQESDITNLPYLQAVVKET

LRLHPPVPLLVPHKAETDVEINGFVVPKDAQILVNVWAIGRDPSLWADPN

SFLPERFSKQKIDFKGQDFELIPFGAGRRLCPGLPLANRMVHLVLASLLH

SFDWKLENGIKAEEMDMSDKFGFTLPKAEPLRAIPIKVGRS

>PvCYP77A1

MAPVSSSSLSSYYHLIFSALALLVSALIFLLSRSAKSKRLNLPPGPPGWP

VVGNLFQVARSGKPFFEYVSDLRPKYGSIFTLKMGTRTMIIVSSAELAHQ

ALIERGQVFATRPRENPTRNIFSSNKFTVNASLYGPVWRSLRRNMVQNML

SANRLKEFVGVRNIAMDRLINKLKMEADANGGVVWVLKNSRFAAFYILLS

MCFGVEMDEETIKRMDDMMKTVLITLDPRIDDYLPILSPFFSKQRKRALE

VRQQQIETIVPFIEKRRSILQNPGSDKTAASFSYLDTLFDLKVEGRKSSP

SNAEIVTLCSEFLNGGTDTTGTALEWAIARLIENPEIQSKLYEEIKSTAG

DRKVDEKDVEKMVYLNAVVKELLRKHPPTYFSLTHAVTEPTKLGGYDIPT

DANVELYLPGISEDPKLWSNPEKFDPDRFLSGKEDADITGVTGIKMMPFG

VGRRICPGLSMATVHVNLMLARMIQEFEWTAYPENSKIDFTGKLEFTVVM

KNSLRARIKPRV

>PvCYP77A4

MAAVSSSSLSSYYHLIFSALALLVSALIFLLSRSAKSKRLNLPPGPPGWP

VVGNLFQVARSGKPFFEYVSDLRPKYGSIFTLKMGTRTMIIVSSAELAHQ

ALIERGQVFATRPRENPTRNIFSSNKFTVNASLYGPVWRSLRRNMVQNML

SANRLKEFVGVRNIAMDRLINKLKMEAEANCGVVWVLKNSRFAAFYILLS

MCFGVEMDEETIKRMDDMMKTVLITLDPRIDDYLPILSPFFSKQRKRALE

VRQQQIETIVPFIEKRRSILQNPGSDKTAASFSYLDTLFDLKVEGRKSSP

SNAEIVTLCSEFLNGGTDTTGTALEWAIARLIENPEIQSKLYEEIKSTAG

DRKVDEKDVEKMVYLNAVVKELLRKHPPTYFSLTHAVTEPTKLGGYDIPT

DANVELYLPGISEDPKLWSNPEKFDPDRFLSGKEDADITGVTGIKMMPFG

VGRRICPGLSMATVHVNLMLARMIQEFEWTAYPENSKIDFTGKLEFTVVM

KNSLRARIKPRV

>PvCYP77B1

MELIDLLIFCFSSVLLCFWWCYWAVPDSRLKNLPPGPRGWPFVGNLVQVI

LQRRPFMYVVRDLRTKYGPIFTMHMGQRTLIIITSSDLIHEALVQKGPIF

ASRPPDSPIRLVFSVGKCAINSAEYGPLWRTLRRNFVTELINPTRIKQCS

WIREWAFEKHMERIKNDASENGFVEVMSNCRLTICSVLICICFGAKISQD

RIKKIEQVLKEVMLMTTLKLPDFLPVLTPLFRPQLRKAKELRKKQMECLV

PLIRQRKVFVESGGKPSPNCSEMASPIGAAYIDSLFGLEPVGRGRLGEEE

LVTLCSEVINAGTDTSATTVEWALLHLVMNQEIQEKLYMEIVKQVGKRGL

ATEADVEKMPYLTAVVKETFRRHPPSHFVLSHAATKETELGGYTIPANVN

VEFYTAWVTEDPTVWKDPGEFRPERFLDGDGVDVDVTGTKGVKMIPFGAG

RRICPAMTLGTLHVNLLLTRMVHAFKWLPVPDAPPDPEETFAFTVVMKNP

LKAIILPR

>PvCYP81D1

MEDIWLLYSSLSLVFALLAFKLFQAKPHKNLPPSPPCLPVIGHFHLLKGP

LHRALYHLSQKHGPIMSLRFGSRLVVVVSSPSAVEECFTKNDVIFANRPR

LTFGKHISYNYTTISTASYGGHWRNLRRLSSLEIFSSDRLNNFSSIRKDE

IKLLLVKLSRDSSSRHKVELKSMLSELTFNNIMRMVSGKRYGEEGEGKHF

RKIMREIFECADASNPGDFVPLLKWIDYKGFEKKVKRLGREADAFLQGVI

NEHRNSLESRNSMIDHLFSFQESEPEYYTDDIIKGLILIMLNAGTDTSAV

TIEWAMSLLLNHPEVLKKARDEIDTHVGNDRLIDEPDVSKLPYLQNIISE

TLRLFPATPLLLPHVSSDDCKVGGFDVPCDTLLLVNAWAIHRDPKFWDDS

MSFKPERFENSEKGHETHTLMPFGFGRRACPGAALANRMVGLALGSLIQC

FEWERIGEEEVNMAEGKGITMPKSEPLEAFCKVRPIMNTVLSNASINN

>PvCYP81D2

MEDGWLLYTSLSLIFVFLAFKLLLKTQKHHKNLPPSPPSLPIIGHLHLLK

EPIHRVLYHLSQKHGPIMSLRFGSRLVVVVSSPSAVEECFTKNDVIFANR

PRLIFGKHIGYNYTTIATASYGDHWRNLRRISSIEIFSSHRLNNFLSIRK

DNIKWLLVKLSRDTREDFAKVELKSMLGELTFNNIMRMVSGKRYYGEEGD

SEKGKHFRRIMREIFECGDASNPADFVPMLKWIDYQGFEKRVKRLHREAD

AFLQGLINEHRNSLESRNSLIDHLLSFQESEPEYYTDVIIKGIILIMLNA

GTDTSAVTIEWAMSLLLNHPEVVKKARAEIDKHVGHDRLIDEHDIAKLPY

LQSIISETLRLFPAAPLLLPHVSSDDCKVGGFDVPRGTILLVNAWAIHRD

PKLWDDSTSFKPERFESSGEGRDTHKLMPFGYGRRACPGAGLANRVVGLA

LGSLIQCFEWKRIGEEEVKMDEGKGLTMPKEEPLEAFCKARPIMNMVLSD

AAIKK

>PvCYP82C1

MEFLPSFPTVVVACISVCLIFVYHLVWKPRSAQKTQTSIRKAPEAAGAWP

IIGHLHLVGGSKLLHKILGSMADQFGPFFTFKLGMKPAIVVSSWELAKEC

LTTHDRIFATRPKSVAFEVLGYNYALFALAPYGNYWRQVRKIATLELLSQ

RRLEQLGHIRQSEVMTWMKELHEQWINKKKNNGGVLVEMKRWFGDLTLNV

IVRMLAGKRSQTEDEESIRFQKVLREFFEFLGMFMVSDAIPSLRWLDLGG

YEKAMKWTAKELDNILGSWLEEHKKRRATDPSDNNTEQDFIDVMLSILTG

DVPGYDADTIIKSMCLVVLAGGTDTTSITLTWALSLLLNNPHALKKAQEE

LDLHVGRDRRVEESDMKKLVYLQAIFKETMRLYPAAPLSAPHESMEDCTV

GGYHVPKGTRLVVNLWKIQRDPRVWTDPCEFKPERFLTRHKDVDVRGQDF

ELLPFGSGRRMCPGMNLSLALMPLTLASLLHGFEVTTVLNEPVDMSESFG

LTNLKATPLEVLVTPRLSSNLYG

>PvCYP82C2

MGFLPSFLVACISVCILFVYYLVWKPRSAQTQTSIRKAPEAAGAWPIIGH

LHLVGGYKLLHIILGSMADQFGPFFTFKLGMKPAIVVSSWELAKECLTTH

DRIFATRPKSVAFEVLGYNYALFVLAPYGNYWRQVRKFATLELLSLRRLE

QLGHIRQSEVMTSIKELHEQWVNKKKNNGGVLVEMKRWFRDLTLNVIVRM

LEGKRYTGEDEESIRFQKALREFFEFVGMLMVSDVIPSLRWLDLGGYEKA

MKRTAKELDNILGSWLEEHKKRRATDPGLVKGNNTEQDFIHVMLSDSILT

GDFPGYDADTIIKAMCLLVLGGGTNTTSITLTWALSLLLNNPHALKKAQE

ELDLHVGRDRRVEESDMNKLVYLQAILKETMRLYPAAPLSAPHESMEDCT

VGGYHVPKGTRLVVNLWKIQRDPRVWADPCEFKPERFLTRHKGVDVRGQD

FELLPFGSGRRMCPGMNMALAVMSLTLASLLHGFEVTTVLNEAVDMSESF

GLTNLKATPLEVLVTPRLSSNLYG

>PvCYP82C3

MEFLDSFSTVVLACIFACLLSVYYLVSKRISARTQICIRKTAPKAAGGWP

IIGHLHLVGGTKILHKILGCMADQFGPVFTLKLGMKPAIVLSSWELAKEC

LTTHDRIFATRPKSVAVEVLGYNYAFFALAPYGNYWRQVRKFAMLELLSQ

RRLEQLGHIRQSEVMTSIKELHEQWVKKKKNNGGVLVEMKRWFGDLTLNV

IVRTLAGKRYTGEDEESIRFQKAIREFFEYVGMFMVSDGIPSLRWLDLGG

YEKAMKRTAKELDNIIGSWLEEHRKREATEPSLAKGNTEQDFIDVMLSIL

TGDLPGYDADTVNKSICLVVLAGGTDTTSITLTWALSLLLNNPHALKKAQ

EELDLHVGRDRQVEESDMKKLVYLQAIFKETLRLYPAAPLSAPHESMEDC

TVGGYHVPKGTRLVVNLWKIQRDPRVWTDPCEFKPERFLTKHKGVDVRGQ

DFELLPFGSGRRMCPGMNLALAVMPLTLASLLHGFEVTTVLNEAVDMSES

FGLTNLKATPLEVLITPRLSSNLYG

>PvCYP82C4

MQFLFSVQTSLAAIFASFVLVYLLQKLRNAHIRTPPEAPGAWPIIGHLHM

LGGTSILHQILGSMADKLGPIFTVKLGLQPTLVVSNWEIAKECFTTHDKA

FATRPKSIAAEVMGYKYATFGLAPYDNYWRDLRKMTTRELLSQSRLAMLS

HIRQSEVKTSINEMYEQWAEKKNKDGWMSVEMNRWFADITLNVIARMLAG

KRYKGETQESVRFKKAMKEFVRFLGVFLVSDGIPFLRWLDLGGHEQAMKR

TSIELDSILESWLDEHKEKRLNNCGGMVKESEQDFMDVMLSILTEGIGGF

DVDTMNKAMCLVVLSGGTDTTAITMSWALSLLLNNRHVLKKAIQELDTQV

GRDRRIEESDIKNLVYLDAILMETMRLYPGAPLSLPHESMEDCTVAGYHI

PKGTRLVVNISKIQSNPSVWSDPHEFRPERFLTTHKDVDVKGKHFELIPF

GSGRRMCPGMNLSLRVMPLTLASLLHGFEFTTLSDEPIDMAESFGLTNHK

ANPLDVLVTPRLSSNLYG

>PvCYP86B1

MSFSYVCNLIPHQLEVKLALMAVVGIWETLVAILCFLVLYRWRSIKKSPI

TNWPLLGMLPAVIGNIFHIHEWAVHVLRQTSYTIEFKGPWFTNMNYLVTS

DPENIRHTYIKNPSNFLKGPAFKSILEVLGDGIFIAESDSWKSQRKLTQS

ALKHTSFQLLLVKITQRKVETGLLPILENASEQGTEVDLQDLFQRFTFDT

TCRLLFGFDPCCLSVESPDVPAVKAFDQIEEVLFYRHAQPEICWKLQKWL

QIGHEKKLTRAWETLDNFLAQHIPLRGGAEQEEGGYSDLLTSYAQEAELT

GAFKISDKFLRDTVVNLLLAGRDTISAAITWFFWLVATNPSVEINILEEM

KAIMKEKQDQKWQFFSATEVNKLVYLHGALCETLRLYPSVPLNHKDVVQP

DILPSGHRVDQNTKMLFFLYSTGRMEEVWGEDCLEFKPQRWITERGGIKY

VPSYKFIAFNAGPRSCLGKEISFVQMKIIATAVLYRFHVQVVEGHPVSPS

NSIILHMKHGLKVRVSKRHV

>PvCYP89A9

METWFIIIATLCISTLLKSIFNLFSPSKTKPRLPPGPPAVPVLTNFLWLR

KSFSDLEPILRNLHAKYGPVVTLRIGSRPAIFVSDRTITHQALIQNGSVF

SDRPNGLPASKILNRNQHNISSASYGPTWRLFRRNITSEILHPSRIKSYA

PARKWVLEILINRLKSQSKSGGQIVERVVDHFQYAMFCLLVFMCFGDKVN

ETQIDAIETIQRDMLLNFNRFNILNFWPSVGKIVFRKRWEELFHLIKTQE

SILIPLIRARNKARQEKQSKAREDPIPHKGDESGFSLAYVDTLFDLELPD

EKRKLNEGEIVSLASEFLNAGTDTTSTALQWIMANLVKNQHIQAKIFQEI

NQVLGPGKEEVTEEDLQYLPYLKAVILEGLRRHPPGHFVLPHAVSEDVKL

DGYFVPKDGTINFMVAEMGWDPSVWADPMEFKPERFLGGGEVFDITGSRE

IKMMPFGVGRRICPGYGLAMLHLEYFVANLILNFEWKAVAGDDIDLSEKQ

EFTVVMKNPLKAQISSRFH

>PvCYP90A1

MEFSPYLVVCFFVCIIVLFLYNPRRLTRRRHRLPPGNLGLPFVGETLQLI

AAYKTENPEPFIDERVNRYGSIFTTHVFGEPTVFSADPETNRFILQNEGK

LFECSYPGSISNLLGRHSLLLMKGSLHKRMHSMTMSFANSSIIRDHLLVD

IDRLIRLNLQSWTDRIFLMEEAKKITFELTVKQLMSFDPGEWTESLRKEY

VLVIEGFFTVPLPFFSTTYRRAIKARSKVAEALTLIVRERREQSEGRERK

NDMLAALLSGDNCFSDEEIVDFLLALLVAGYETTSTIMTLAIKFLTETPL

ALAQLKQEHDEIRATKDDSETLGWNDYKSMPFTQCVVNETLRVANIISGI

FRRAMTDVHIKGYTIPKGWKVFASFRAVHLDQDHFKDARTFNPWRWQNNS

GVTSPVNVFTPFGGGPRLCPGYELARVILSVFLHHMVTGFSWVPAEEDKL

VFFPTTRTQKRYPIYVHRKDNSKQCKKKKKKKKKKKKKK

>PvCYP90A2

MMEFSPYLVVCFFVCIIVLFLYNPRRLTRRRHRLPPGNLGLPFVGETLQL

IAAYKTENPEPFIDERVNRYGSIFTTHVFGEPTVFSADPETNRFILQNEG

KLFECSYPGSISNLLGRHSLLLMKGSLHKRMHSMTMSFANSSIIRDHLLV

DIDRLIRLNLQSWTDRIFLMEEAKKITFELTVKQLMSFDPGEWTESLRKE

YVLVIEGFFTVPLPFFSTTYRRAIKARSKVAEALTLIVRERREQSEGRER

KNDMLAALLSGDNCFSDEEIVDFLLALLVAGYETTSTIMTLAIKFLTETP

LALAQLKQEHDEIRATKDDSETLGWNDYKSMPFTQCVVNETLRVANIISG

IFRRAMTDVHIKGYTIPKGWKVFASFRAVHLDQDHFKDARTFNPWRWQNN

SGVTSPVNVFTPFGGGPRLCPGYELARVILSVFLHHMVTGFSWVPAEEDK

LVFFPTTRTQKRYPIYVHRKDNSKQCKE

>PvCYP90B1

MSDLEFLLYLVPSILAVFLIRNFMKKKKQAVFNLPPGSMGWPFLGETIGY

LTPYSATTIGDFMEQHISRYGKIYKSNLFGEPTIVSADAGLNRFILQNEG

RLFECSYPRSIGGILGKWSMLVLVGDMHRDMRIISLNFLSHARLRTSLLR

EVEKHTLLVLNSWKENCTFSAQEEAKKYTFNLMAKHIMSLDPGKPETEQL

KKEYITFMKGVVSAPLNLPGTAYRKALQSRSTILKFIELKMEERIKKMEE

GCDSMEEDDLLGWVLKHSNLSKEQILDLVLSLLFAGHETSSVSIALAIYF

LQACPAAVHQLKEEHLEIAEAKKQSGKLELTWDDYKKMDFTQCVISETLR

LGNVVRFLHRKAIKDVRYKGYDIPSGWKVLPVIAAVHLDSSLFDQPQEFN

PWRWQNSNSGSSTWPNTTTTSSTFMPFGGGPRLCAGSELAKLEMAVFIHH

LVLNYHWELADTDNAVAFPFVDFPNGLPIRVQHHPLL

>PvCYP90C1

MKACKKKMRVILGTWVLVLSVVFGLCCWYDIKKKKKKKKMMLMIKTEKSG

VPKGNSFWPLIGETLDFITCGYSSRPVSFMEKRKSLYGKVFKTHLLGKPI

IVSMDPDVNKMVLQNHGNIFMPSYPKSITELLGESSILQMNGTRHKRLHG

LIAGFLKSPQLKSKIIQDIENSVKLSLASWQHMHPIYVQDQTKKFTFEVL

VRVLMSVGPGEDLEFLKIEFGECIKGLICLPINLPGTRLYKSLKAKERLL

KMVRKIVEQRKLAMDDDRAPVNDVVDLLLRDTSDSYETQRIPLDFISGNI

IEMMIPGEDTVPMVMTLAVKFLGDNPVALHQLMEENMELKRQKTHSCDDY

AWNDYMSLPFTQNVISETLRMANIINGVWRKALKDVEIKGYLIPRGWCVL

ASFISVHMDEENYENPFQFDPWRWEKKGAVNSSNFTPFGGGQRLCPGLEL

SRLEISIFLHHLVTTYRWVAEEDDITHFPTVKMKRKLPITVTPIDE

>PvCYP90C2

MKAWKKKMRVILGTWVLVLSVVFGLCCWYDIKKNKKKKKMMLMIKTEKSG

VPKGNSFWPLIGETLDFITCGYSSRPVSFMEKRKSLYGKVFKTHLLGKPI

IVSMDPDVNKMVLQNHGNIFMPSYPKSITELLGESSILQMNGTRHKRLHG

LIAGFLKSPQLKSKIIQDIENSVKLSLASWQHMHPIYVQDQTKKFTFEVL

VRVLMSVGPGEDLEFLKREFGECIKGLICLPINLPGTRLYKSLKAKERLL

KMVRKIVEQRKLAMDDDRAPVNDVVDLLLRDTSDSYETQRIPLDFISGNI

IEMMIPGEDTVPMVMTLAVKFLGDNPVALHQLMEENMELKRQKTHSCDDY

AWNDYMSLPFTQNVISETLRMANIINGVWRKALKDVEIKGYLIPRGWCVL

ASFISVHMDEENYENPFQFDPWRWEKKGAVNSSNFTPFGGGQRLCPGLEL

SRLEISIFLHHLVTTYRWVAEEDDITHFPTVKMKRKLPITVTPIDE

>PvCYP90C3

MRVILGTWVLVLSVVFGLCCWYDIKKNKKKKKMMLMIKTEKSGVPKGNSF

WPLIGETLDFITCGYSSRPVSFMEKRKSLYGKVFKTHLLGKPIIVSMDPD

VNKMVLQNHGNIFMPSYPKSITELLGESSILQMNGTRHKRLHGLIAGFLK

SPQLKSKIIQDIENSVKLSLASWQHMHPIYVQDQTKKFTFEVLVRVLMSV

GPGEDLEFLKREFGECIKGLICLPINLPGTRLYKSLKAKERLLKMVRKIV

EQRKLAMDDDRAPVNDVVDLLLRDTSDSYETQRIPLDFISGNIIEMMIPG

EDTVPMVMTLAVKFLGDNPVALHQLMEENMELKRQKTHSCDDYAWNDYMS

LPFTQNVISETLRMANIINGVWRKALKDVEIKGYLIPRGWCVLASFISVH

MDEENYENPFQFDPWRWEKKGAVNSSNFTPFGGGQRLCPGLELSRLEISI

FLHHLVTTYRWVAEEDDITHFPTVKMKRKLPITVTPIDE

>PvCYP90C4

MKAWKKKMRVILGTWVLVLSVVFGLCCWYDIKKNKKKKKMMLMIKTEKSG

VPKGNSFWPLIGETLDFITCGYSSRPVSFMEKRKSLYGKVFKTHLLGKPI

IVSMDPDVNKMVLQNHGNIFMPSYPKSITELLGESSILQMNGTRHKRLHG

LIAGFLKSPQFKSKIIQDIENSVKLSLASWQHMHPIYVQDQTKKFTFEVL

VRVLMSVGPGEDLEFLKREFGECIKGLICLPINLPGTRLYKSLKAKERLL

KMVRKIVEQRKLAMDDDRAPVNDVVDLLLRDTSDSYETQRIPLDFISGNI

IEMMIPGEDTVPMVMTLAVKFLGDNPVALHQLMEENMELKRQKTHSCDDY

AWNDYMSLPFTQNVISETLRMANIINGVWRKALKDVEIKGYLIPRGWCVL

ASFISVHMDEENYENPFQFDPWRWEKKGAVNSSNFTPFGGGQRLCPGLEL

SRLEISIFLHHLVTTYRWVAEEDDITHFPTVKMKRKLPITVTPIDE

>PvCYP90C5

MRVILGTWVLVLSVVFGLCCWYDIKKKKKKKMMLMIKTEKSGVPKGNSFW

PLIGETLDFITCGYSSRPVSFMEKRKSLYGKVFKTHLLGKPIIVSMDPDV

NKMVLQNHGNIFMPSYPKSITELLGESSILQMNGTRHKRLHGLIAGFLKS

PQLKSKIIQDIENSVKLSLASWQHMHPIYVQDQTKKFTFEVLVRVLMSVG

PGEDLEFLKIEFGECIKGLICLPINLPGTRLYKSLKAKERLLKMVRKIVE

QRKLAMDDDRAPVNDVVDLLLRDTSDSYETQRIPLDFISGNIIEMMIPGE

DTVPMVMTLAVKFLGDNPVALHQLMEENMELKRQKTHSCDDYAWNDYMSL

PFTQNVISETLRMANIINGVWRKALKDVEIKGYLIPRGWCVLASFISVHM

DEENYENPFQFDPWRWEKKGAVNSSNFTPFGGGQRLCPGLELSRLEISIF

LHHLVTTYRWVAEEDDITHFPTVKMKRKLPITVTPIDE

>PvCYP97B2

MATSLLQLLPPVVSGSIHLNDSFFTGVSKPPYLNSSATHFFSSKPKGCRI

LRCQSTSTDEPKTRNLLDNASNLLTNLMSGGSLGSMPIAEGAVSDLFGRP

LFFSLYDWFLEHGSVYKLAFGPKAFVVVSDPIVARHILRENAFCYDKGVL

ADILEPIMGKGLIPADLDTWKQRRRVIAPGFHALYLEAMVKIFTDCSERT

VLKFDKLLEAEDLHGGKTIELDLEAEFSSLALDIIGLGVFNYDFGSVTKE

SPVIKAVYGTLFEAEHRSTFYIPYWKIPLSRWIVPRQRKFRKDLKIINDC

LDRLIRNAKETREETDVEKLQQRDYSNLKDASLLRFFVDMRGADADDRQL

RDDLMTMLIAGHETTAAVLTWAVFLLAQNPSKMKKAQAEIDSVLGQGKPT

YELLKKLEYIRLIVVESLRLFPQPPLLIRRSLKPDLLPGGYKGDKDGYAV

PAGTDIFISVYNLHRSPYYWDRPDEFEPERFLEQKMSEGIEGWAGFDPSR

SLGALYPNEIISDFAFLPFGGGPRKCVGDQFALMESTVALAMLLQKFDVE

LKGSPEAVELVTGATIHTKSGLWCKLRKRSNVL

>PvCYP97B3

MGPACSWAMATSLLQLLPPVVSGSIHLNDSFFTGVSKPPYLNSSATHFFS

SKPKGCRILRCQSTSTDEPKTRNLLDNASNLLTNLMSGGSLGSMPIAEGA

VSDLFGRPLFFSLYDWFLEHGSVYKLAFGPKAFVVVSDPIVARHILRENA

FCYDKGVLADILEPIMGKGLIPADLDTWKQRRRVIAPGFHALYLEAMVKI

FTDCSERTVLKFDKLLEAEDLHGGKTIELDLEAEFSSLALDIIGLGVFNY

DFGSVTKESPVIKAVYGTLFEAEHRSTFYIPYWKIPLSRWIVPRQRKFRK

DLKIINDCLDRLIRNAKETREETDVEKLQQRDYSNLKDASLLRFFVDMRG

ADADDRQLRDDLMTMLIAGHETTAAVLTWAVFLLAQNPSKMKKAQAEIDS

VLGQGKPTYELLKKLEYIRLIVVESLRLFPQPPLLIRRSLKPDLLPGGYK

GDKDGYAVPAGTDIFISVYNLHRSPYYWDRPDEFEPERFLEQKMSEGIEG

WAGFDPSRSLGALYPNEIISDFAFLPFGGGPRKCVGDQFALMESTVALAM

LLQKFDVELKGSPEAVELVTGATIHTKSGLWCKLRKRSNVL

>PvCYP97C1

MSSSFYISSFSPLTPPHKSTTLSSNSKIPPRSLTVKSSIDKDSSTKANQT

TNAKTNSWVSPDWLTSLTRSLTVGQDDSGIPIASAKLDDVSELLGGALFL

PLFKWMNQYGPIYRLAAGPRNFVVVSDPAIAKHVLRNYGKYGKGLVSEVS

EFLFGSGFAIAEGSLWTVRRRAVVPSLHKKYLSIMLDRVFCKCAERLVEK

LQADALNGTAVNMEAKFSQLTLDVIGLSVFNYNFDSLAADSPVIDAVYTA

LKEAEARSTDLLPYWKFKALCKIIPRQIKAEKAVTLIRECVEDLIEKCKL

IVDREGERIDEEEYVNDADPSILRFLLASRENVSSVQLRDDLLSMLVAGH

ETTGSVLTWTLYLLSKNSSSLKKALEEIDRVLQGRPPTYEDIKELKFVTR

CINESMRLYPHPPVLIRRAQVADVLPGDYKVNAGQDIMISVYNVHHSSQV

WERAEEFLPERFDLDGPVPNETNTDFRYIPFSGGPRKCVGDQFALLEAIV

ALVIFLQHLNFELVPDQNISMTTGATIHTTNGLYMKLSQRQTKSAFAEVC

T

>PvCYP98A3

MALILPISIFLFLLLAYKLYQRLKFKLPPGPRPWPVVGNLYDIKPVRFRC

FFEWSQAYGPIISVWFGSTLNVVVSNSELAKEVLKENDQQLADRHRSRSA

AKFSRDGTDLIWADYGPHYVKVRKVCTLELFSPKRLEALRPIREDEVTAM

VESIFKDCAHPDNDGKSLLVKKYLGAVAFNNITRLAFGKRFENSEGKMDE

QGLEFKAIVSNGLKLGASLAMAEHIPWLRWMFPLEEEAFAKHGARRDRLT

RSIMEEHTLARQKSGVKQHFVDALLTLQEKYDLSEDTIIGLLWDMITAGM

DTTAISVEWGMAELIKNPRVQQKAQEELDRVIGSERILTEPDFANLPYLQ

CVAKETLRLHPPTPLMLPHRANASVKIGGYDIPKGSNVHINVWAIARDPA

VWRDPLEFRPERFMEEDVDMKGHDFRLLPFGAGRRVCPGAQLGINLVTSM

LGHLLHHFKWSPPEGVKSEEIDMSENPGLVTYMRTPLQAVAIPRLPAHLY

KRVAVDM

>PvCYP704B1

MISVAFLYNPVTFTVVALILSLFTIKFITRKLKRSQRKKYPPIAGTIFNQ

LLNFNRLHHYMTDLAGKHRTYRLLSPFRNEIYTADPANVEYILKTNFENF

GKGCYNYSLLKDLLGDGIFTVDGEMWRQQRKVSSYEFSTKILRDFSSVVF

RRNVAKLANIVSIAVASNQIIDIQDMFMKSTLDSIFKVGFGVELDSMCGS

SEEGASFSNAFDDSSAMILWRYVDIFWKIKKFLNTGSEATLKKSIKVVNS

FVYKLIQSKIDQMRNSEDDTCMKKEDILSRFLQLNETDLKYLRDIILNFI

IAGKDTTATTLSWFIYMLCKHSYIQEKVAKEIEEATNMEEITSFAEFAAR

MNEAVLEKMHYLHAALTETLRLYPAVPVDAKICFSDDTLPDGFSVRAGDM

VSYQPYAMGRMRFVWGDDAEVFRPERWLDEDGIFRPESPFKFTAFQAGPR

ICLGKEFAYRQMKIFAAVLLGCFIFKLSDEQKTVNYRTMINLHIDGGLHV

RALHRRPHC

>PvCYP706A3

MTSILNFIHNLAGLDKPWPQYLDNTNSKLIFSVAIAFLSISWIFIRKKNP

SLPPGPRGLPLIGNLASLDPELHTYFAHLAQTYGPILKLQFGKKIGIIIT

SPALAREVLKDNDITFANRDVPVAAAAGAYGGSDIVWSPYGAEWRMLRKV

CVVKMLSNATLDSVYDLRRQEMRQTVGYLYKRVGSPVNIGEQMFFCVLNV

ITNMLWGGTVSGEAKASLGDEFRQAVDEMTAGLLKPNLSDFFPGLARFDL

QGVAKKMKGLGLWFDRIFDSVIDQRLKMDGEEAKSTEVNARGSKDFLHFL

LKLKDEEDAKTPFTMNHLKALLLDMVVGGTDTSSNTIEFAMAEMMNNPEV

MKKVQQELETVVGKDKVVEESHINKLPYLHTVMKETLRMHPVLPLLVPHC

PSESCTVGGYTILKGVRVFINVWAIHRDPSVWENPSLFDPDRFSNGKWDY

NGNDFSYFPFGSGRRICAGTAMAERMVMFSLATLVHSFDWELAQGEKSDI

LEKFGIVLKKRTPLIAIPTPRLSDLALYE

>PvCYP706B1

MISHLLEKTARNQLTWWWEGSSKTHAVIQTVVTLSVVMLAIFWCLNKRLP

TLPPGPRGLPVVGYLPFLGTDLHRSFAQLASVYGPIYKLWLGRKLCVVIS

SPHLIKEVVRDHDLIFANRAPTVAGLVVSYGGLDIGFASYGPQWRNLRKV

FVRDLMSNNSLDACYYLRKQVVRKAIRDVYPKIGTPINIGKITFLTALNV

IMSLLGCSTIEGDDKSNATAEQFQEVTLKIVQLVAKPNISDFFPILARFD

MQGVEQEMRRLVLGAEDILNFIIDQRMNTDTSKGEAALKKKEKRDFLQIL

LEQRDQEDTETPITLTQIKAVLMDIVVGATDTTSTAAEWVIGEMIQHPEI

MEKVQEELEQVVGMNNIVEEFHLNQLTYLEAVVKEAFRLHPPAPLLIPRR

PSLSCSVGGYRVPKGTTIFLNVWAMHMDPHVWNNPTEFKPERFLNDTGTW

DFTGKNFEYLPFGSGRRICPGVPLAERMLMYVLASLLHSFNWRLPEGEEL

DLSDTFGIILKKRTPLIVILAQRLSKLELYT

>PvCYP712A1

MASSENALFYTFLFSIFLFLTLFIKSFINRTTVRRPPPSPLALPIIGHLH

LLTSDVLCKNFQTLSHRYGPLMELRIGASTSIIASDASVAKEIFKTHEQI

FSSRPEFGAEEYSIYAGSSFVTAPYGDYWRFLKKLCMTRLLSTTQLDRFV

HIRENEIKRMLKSLMNKSRELEPVDMRLQLNILTSNTICLMAMSTRCSDN

SNEANEILSFVKEVTELAGKLALGDAFGPLKRFNFFGRGEDLVSVLRRFD

KLVEGILKEHEVNGSNGREEDLMDILLEVQKDPSAEVKLSRNNIKGFLLD

IFMGGSDTASVAMQWIMAELINHPQIFKKLRQEINSVVGTERLVKESDVP

NLPYLRAVIKESLRLHPSVPLILRQCTEDCKINGYDVKAKSRIMVNAYAI

MRDPSLWKDPNEFVPERFLMDSDEKIGEHVMETKGQNFRFLPFGSGRRGC

PGSSFALTVMHATVGALVQCFDWKTKGVEKLDMKMGPGFAGEMGVPLVCY

PITHFNPFD

>PvCYP714A1

MEQATMTLVAKSFFSVASIAFAFFFLHLFNILWLKPRSVRSKLSSQGLRG

PSPSFLVGNLPEMKRIDAEFVRPPLKAGEVKHDYAYTIIPWFIQWTKQYG

SNYFFSLGNIPILCVGEHEAVKEINICTSLDLGKPSYLQKDRGPLLGKGI

LTSNGQSWTYQKKIIAPELSMEKVKGMVNVMVESATSLVSSWESMVEEGG

GVVDIRIDQDLKRFAADVISKACFGSSYSKGQHIFARTSALQVAMAKKSL

SGFPALKYLPIENNRKVWRLEKEIHQCILEVVKERKQDGNIHKDLLQAVI

DGANHSGLGPAAIDNFIVDNCKNIFLASHGTTAFSIGWALLLLAANPDWQ

ERVRDEVLQICGGRNPDADMLRQMKLLTMVLQETLRLYPSSVIMSKEVSE

DMTFGDIFVPKGVSIWIPTITLHRDPLSWGKDAEEFNPDRFANGTASATK

HPQSFLPFGAGPRICIGQSFAMVELKIIIALILANFSFSIAPNYKHSPVL

SLIIEPEYGMYMRITKLKKN

>PvCYP714A2

MEQATMTLVAKSFFSVASIAFAFFFLHLFNILWLKPRSVRSKLSRQGLRG

PSPSFLVGNLPEMKRIDAEFVRPPLKAGEVKHDYAYTIIPWFIQWTKQYG

SNYFFSLGNIPILCVGEHEAVKEINICTSLDLGKPSYLQKDRGPLLGKGI

LTSNGQSWTYQKKIIAPELSMEKVKGMVNVMVESATSLVSSWESMVEEGG

GVVDIRIDQDLKRFAADVISKACFGSSYSKGQHIFARTSALQVAMAKKSL

SGFPALKYLPIENNRKVWRLEKEIHQCILEVVKERKQDGNIHKDLLQAVI

DGANHSGLGPAAIDNFIVDNCKNIFLASHGTTAFSIGWALLLLAANPDWQ

ERVRDEVLQICGGRNPDADMLRQMKLLTMVLQETLRLYPSSVIMSKEVSE

DMTFGDIFVPKGVSIWIPTITLHRDPLSWGKDAEEFNPDRFANGTASATK

HPQSFLPFGAGPRICIGQSFAMVELKIIIALILANFSFSIAPNYKHSPVL

SLIIEPEYGMYMRITKLKKN

>PvCYP714B1

MDLFMAFKIFMSLALVGFLGLIVSLYNALVVKPGKLRSALSKQGITGPPP

TFLLGNIMEIKKSRSTTPKVPTKEPPVIHDCAGTLFPFFDQWREKYGKVF

MFSLGNTQILYVNQPDMVREITTCTSMDLGKPSYQQKERGPLLGTGILTS

NGPTWAHQRKILAPELYMEKVKGMLNLVTDSAVTLINSWKTRIEADGGIA

DIEIDQHMRSFSGDVISRACFGSNYSKGEDIFLKLWDLQEIMSKKSLSFG

IPGLRHLPTKTNREAWAMEKEVRNLILDVVKERKEAAYEKDLLQMVLEGA

KNSDLSQEQTNQFIVDNCKNVYLAGYETTAVSASWLMMLLAANPEWQERV

RAEVVEVCKGRTPDADMLRKMKQLQMCIHESLRLYPPVGVVSREAFNDMK

FGDIIVPKGVNIWTMMATLHTDPENWGPDATKFNPERFANGITGACKLPH

LYMPFGVGPRVCLGQNLAVVELKMVVSLILSNFSFSLSPKYIHSPTLKLV

IEPQHGVHLLIKKL

>PvCYP714B2

MADLVKMAVTLALVGIVSLVLYVCEFLLWKPARLRSKIVKQGITGPPPSF

LIGNLGALQKKLPPKVSERPSEGDAPVILSHDNTPTLFPIFEEWRKQYGP

IFNFSLGNIQVLYMQRHDVVKEMGTCTSMDFGKPAYLHHELGPLLGKGII

TSNGPKWSHQRKILAPELSMEKVKGMTKSMLEASTTLLSSWNNIIDNSKG

GVADINIDEHLRSFAGDVISRACFGSNYDKGEEIFLRLRVLIEASAKKMV

AIGLPGLRYLPTKSNRETWNLEKEIRSLILKAINERDQSASKNLLQMVLE

SAANSNLSPSEMDVFIVDNCKNIYLAAYETTAASADWLLMLLAAHPEWQE

RIRAEVREVCAGRMPDADMVRKMKSLTMALQESLRLYPTTALQVREAMKD

MKFGDIHVPKGVSVWTMIIALHQDVDNWGPDAHLFKPERFANGVTGACKF

PYMYMPFGVGPRMCLGQNFAMTDLKILVALIVSNYSFTLSPQYRHCPVMR

MNIEPEYGVILRIKKL

>PvCYP716A1

MEHFYLSLLFTFLASVFLGLSYLFIKHRSDFTGPNLPPGKIGWPMVGETL

EFLSTGWKGHPEKFVFDRMAKFSTQIFKTSLLGEPLAVVCGASGNKFLFT

NENRLVQSWWPNSVNKVFPSSQQTSSKEEAIKMRKMLPQFLKPEALQRYI

PIMDHIAQRHFAAGWDNKDEVTVFPLAKRYTFWVACRLFLSIEDPNHVAK

FADPFNLLASGLISVPIDLPGTPFYKAIKASDFIRKELYEIIKQRKIDLA

DKKASPTQDILSHMLLATDDNGKFMTEMDIADKILGLLIGGHDTASAAIT

FIVKYLAELPEIYQRVHKEIMEIVSTKSPGELLNWEDIQKMKYSWNVASE

VLRLAPPLQGAFREALTEFMYAGFSIPKGWKLYWSANSTHRNADCFPEPE

KFDPSRFEGSGPAPYTYVPFGGGPRMCPGKEYARLEILVFMYNVVKRFSW

DKLLPDEKIIVDPMPMPAKGLPVRLHAHKA

>PvCYP716A2

MELISLSLLVVLFVISFSLIYYRHRPKSATKLPPGNGGWPIIGESIEFLL

SGWRGHPEKYIFDRVTKYSSKIFKTTLFGEPAAVVSGAAGNKFLFSNEDK

LVIAWWPTSVKKIFPYLVQTETNEEAIKLRKLLPQFLKPEALIRYIGIMD

TTTQRHFARSWENNKQVTVYPLAKKYAFLVACRLFLSLEDPDQVDRLGDP

FNYIAAGIFSIPIDLPGTPFNRAIKEAAFVRKEIVAIIKQRKVDLAENKA

SPTQDVLSHMLLLSDENGEMMNEVDIANKLMAVLVGNHDSMSSAITSIVK

YLAELPHIYDQVYQEQMEIANSKAPGELLNWSDIQKMKYSWNVVCEVLRL

APPIQGTFRESISDFIISGFSIPKGWKLYWSANSTHRNPEYFPEPEKFDP

TRFQGAGPAPYTYVPFGGGPRMCPGNEYARFELLVFVFNIVTRYKWEKLI

PDEKTRVYPTPRPAMGLPIRLHPHKA

>PvCYP716A3

MELFFLSGLALFISVSVISLTIFFTKKTNGASRPPGTTGYPYIGESIEFF

SGGKKGHPEKFILDRMSKFSSQVFKTSLFLEPAAVFCGAAGNKFLFSNEN

KLVQAWWPKSVDKIFPSSLQTSSKEESIKMRKLLPQFMKPEALQKYISIM

DTIAQRHFEAGWEGKDEIVVFPLAKNFTFWLACKLFLSIDDPEQVAKFAD

PFECIASGIVSIPIDLPGTQFNQGIKASNLIRKDLRAIIKQRKIDLAEKK

ASPTQDILSHMLLTSDENGQFMNEADIADKILGLLIGGHDTASASCTFVV

KYLAELPHIYDEVYREQMEVAASKKPGELLNWDDIQKMKYSWNVACEVMR

LAPPVQGAFREAMNDFIYAGFSIPKGWKLYWSAHSTHRNPECFPEPEKFD

PSRFEGKGPAPYTYVPFGGGPRMCPGKEYARLEILVFIHNLVKRFKWEKV

LPEEKIIVNPMPIPAHGLPIRLHPHKN

>PvCYP716B1

MNPTIYFFITLLFFVLPLFHLLFRRRSSKKLPPGSMGFPIIGQSLTVLKA

MQTNTTEQWLQQRIKKYGPVSKLSLFGTPTVFLHGLAANKFIYTSDGNTL

ANDQPKSIRMILGAKNLMELKGNDHKRVRGALLSFLKPEALKQSVGKMDE

EIRLHINTHWHGHQKVSVMPSVKMLTFNVMCSLLFGIERGVKRETLVNLF

EIMVAGLMLMPINLPFTRFNQSLRAAAKVKTIIRDLIRERREALEKHGSS

PDQDLITCLLDMRGKDNSVVISDEEIVDNAILTMVAGYDTTSTLITFLVK

TLATDPSIYAAIVQEQEEIAKCKSPGEALTWDDLAKMKYTWRVATETLRM

TPPVLFSFRKTLKDIEYEGYTIPKGWQVVWATTMTHMDESIFPNPTKFDP

TRFEKKALAPPYSLVPFGAGQRICPGNEFARIESLSTIHYLVTHFTWKLD

CKDASFSRDPLPVFNEGLPIQITSKIK

>PvCYP716B2

MLLSNTITTMDTLLIICLFLVPVFILLVRRRSSKRLPPGSLGLPIIGQSL

GLLRAMKANTAEKWLQERIDKYGPVSKLSLFGTPTVFIHGQAANKMVFTS

DGSTLGNQQIESIQRILGDETLLKLSGKDHKRVRDALSSFLKPESLKQHV

KKMDEEIRSHLENNWQGQEKITVLNQMKTLTFDIICSLLFGLEQGPRRDS

FVHNLSLMIDGVWSIPLDLPFSRYRRSLKASDRVQNMVRDLIAEKRLGLE

KKGVSPNQDLITKLLSLRGEDNEEVMSETEIVHNVLLTMVAGHETSSVVI

TFFMRLLANNPTVYEAVLKEQEEVAKSKPSGELLTWEDLSKMKYTWRVAL

EIMRVDPPVFGGFRKALKDIEYEGYIIPKGWQIFWVASMTHMDSNIFSDP

TRFDPSRFENQGSVPPYTFIAFGAGYRICPGYEFAKMEILVAVHHLITRF

SWKLCHTDNRFSRDPSPIPTEGLPVFLFPKN

>PvCYP736A11

MLNRHHMSIKLAMSWPWIVFPFLAFVYLLQGWLRKNSKRLPPGPRRLPII

GNLHILGKFPHRDLHRLSNIHGPIMYMRFGFVPVVVVSSPQAAEQFLKTH

DLVFASRPPLEAGKHIFYDQKNMSFSPYGSYWRSMRKLCTLELLSNLKIN

SFKTMRKQELGLMIESLKEAACGRVAVDISAMVSTLSADMSCLMVLGKKY

LDREFDERGFKALIQDSMYLGAKLNIGDCIPQLAALDLQGLTKRMKAVSK

VFDSFFEKIIDEHVQSKDEKPTKDFVDTMLAIMNSQKIDFHIDRSNIKAT

ILDMLSASMDTSATAIEWALSELLKHPQVMKKVQNELEKVVGKERMVEES

DLESLKYLDMVVKETFRLHPVAPLLLPHESMEDCTINGFHIPKKSRVMIN

AWAIGRDPEVWMDAEKFLPERFIEDNIDIQGHDFRLIPFGSGRRGCPGMQ

LGITMVRLVVAQLVHCFDLELPNDLLPTELDMTEEFGLTIPRSKHLYAIP

TYRLYK

>PvCYP736A12

MTMAWAWAVFALLVGFAYLLRSWASKQKIKTKRLPPGPRRLPIIGNLHML

GEFPHRDLLRLANIHGPIMYMRMGFVPVVVVSTPQAAEQFLKTHDLVFAS

RPPHQAAYIISYEQRNLSFAPYGSYWRNMRKLCTLELLSNLKINSFKAMR

KEELGLVIESLKEAARGHVAVDVSAMVSTLSADMSCLMVFGKKYLDREFD

ERGFKAVIQDGMHLAAVANIGDYIPQLAALDLQGLTKRMKAIRKVFDAFF

EKIIDEHVQSKDEKQTKDFVDTMLDIMNSQDSEFPIDRTNIKANILDMLA

GSMDTTATAIEWAISELLKHPRIMKKVQNELEKVVGKGRMVEESDLESLE

YLDMVLKESLRLHPVAPLLIPHESIEDCTINGFHIPKKSRVVINAWAIGR

DPDAWTDAEKFIPERFVGNNVDIRGRDFRLLPFGSGRRGCPGMQLGLTVI

RLVVAQLVHCFDWELPNDMLPTELDMSEEFGLTVPRANPLLAIPSYRLHR

>PvCYP736B1

MLSYSTLTLILLFFLQVVWPLIFHLRSKPKSPPGPRPLPIIGNLHILTHP

HPHRTLSQLAKTYGDIMSLRLGSIPTIVVSSPKAAELFLKTHDAVFANRP

RVQAYDYLSFGSKGVAFTEYGPYWRDVRRLCTVELLSTAKVDSFASMRRD

ELGLLVQSLQKSAAARQLVNVSVKVGELIEDMTYRMVFGSGKENRFDLKA

VIREATSLAGAFNLADYMPWLRPLDLQGLNRRLKETRRLVDKILEEIIDE

HEQETSKNQATNCRSFIDVMLSLNKKSINIHNYEQSNTINRTNIKAILLD

MLVGTIETSAIAIEWALSELLKHPRVMVQVKNELTSIIGPKRKVEEKDLA

KLAYLDMVLKESFRLHVSPFLVPHESTEDVVINNFYIAKNTRIIINAWAI

GRDPEMWSENAEEFIPERFIGSNVDVKGHDFQLIPFGSGRRGCPGIQLGL

TTVKLVLAQLLHCFDWELPNGMKPNDLDMSERFGLTMPRENHLLAMPTYR

LLDNELLN

>PvCYP736B2

MLSYSTLTLILLFFLQVVWPLIFHLRSKPKSPPGPRPLPIIGNLHILTHP

HPHRTLSQLAKTYGDIMSLRLGSIPTVVVSSPKAAELFLKTHDAVFANRP

KVQAYDYLSFGSKGVAFTEYGPYWRDVRRLCTVELLSTAKVDSFASMRRE

ELGLLVQSLQKSAAARQLVNVSVKVAELIEDMTYRMVFGSRKESGFDWKA

VIREATSLAGAFNLADYMPWLRPLDLQGLNRRLKEIRRLVDKILEDIIDE

HEQETSKHQATNCRGFIDTMLSLNKKSINIHNYEQSNTINRTNIKAILLD

MLVGTIETSAIAIEWTLSELLKHPRVMVQVKNELMSVIGPNRKVEEKDLA

KLAYLDMVLKESFRLHVSPFLVPHESREDVVINNFYIAKNTRIIINAWAI

GRDPEMWSENAEEFIPERFIGSNVDVKGHDFQLIPFGSGRRGCPGIQLGL

TTVKLVLAQLLHCFDWELPNGMKPNDLDMSERFGLTMPRENHLLAMPTYR

LLDNELLN

>PvCYP736B3

MLSYSTLTLILLFFLQVVWPLIFHLRSKPKSPPGPRPLPIIGNLHILTHP

HPHRTLSQLAKTYGDIMSLRLGSIPTVVVSSPKAAELFLKTHDAIFANRP

KVQAYDYLSFGSKGVAFTEYGPYWRDVRRLCTVELLSTAKVDSFASMRRE

ELGLLVQSLQKSAAARQLVNVSVKVAELIEDMTYRMVFGSHKESGFDWKA

VIREATSLAGAFNLADYMPWLRPLDLQGLNRRLKEIRRLVDKILEDIIDE

HEQETSKHQATNCRGFIDTMLSLNKKSINIHNYEQSNTINRTNIKAILLD

MLVGTIETSAIAIEWTLSELLKHPRVMVQVKNELMSVIGPNRKVEEKDLA

KLAYLDMVLKESFRLHVSPFLVPHESREDVVINNFYIAKNTRIIINAWAI

GRDPEMWSENAEEFIPERFIGSNVDVKGHDFQLIPFGSGRRGCPGIQLGL

TTVKLVLAQLLHCFDWELPNGMKPNDLDMSERFGLTMPRENHLLAMPTYR

LLDNELLN

>PvCYP749A17

MGILLTSLLSSCLCVCLLLILIKFIYQVWWTPIRLQRALGKQGIRGPPYN

YFHGGNSKEIVKMRQEAISKPMNISSHYMFPRIHPYLYSWVKQYGPNVLH

WIGPQAFLYISETEMIKEILNKTEAGYPKLEAEEFEKKLLGEGLATSRGE

KWAKARKLANHEFHGDSLKNMIPDMITSVEIMVKRWEKYVGKEIEVFEEL

RLLTSEVISKTAFGSSFLQGEKIFEMLIELTEIFSRNEYKYRFPIISKLI

RSEDDIESEKLENAIKESVTEMIRSREKRVASGELENFGNDFLGKLVKAS

HDVDVKKRFSVDDMIDECKTFFVAGQETTNSLLSWAMLLLAIHPEWQEEA

RKEVFKLFGQGAPTSDGISKLKILHMIINESLRLYPPVVGVTRKVEREVR

VGKFTLPADLNFYISTLASHHDARIWGEDVHLFNPERFAEGVAKATRNNI

GAFFPFSLGPRTCVGNNFAMIESKITLSMILQRYSFTLSPSYVHAPIHVL

TLKPQQGVQLMLHAL

>PvCYP749A18

MGILLTSLLSSCLCVCLLLILIKFIYQVWWTPIRLQRALGKQGIRGPPYN

YFHGGNSKEIVKMRQEAISKPMNISSHYMFPRIHPYLYSWVKQYGPNVLH

WIGPQAFLYISETEMIKEILNKTEAGYPKLEAEEFEKKLLGEGLATSRGE

KWAKARKLANHEFHGDSLKNMIPDMITSVEIMVKRWEKYVGKEIEVFEEL

RLLTSEVISKTAFGSSFLQGEKIFEMLIELTEIFSRNEYKYRFPIISKLI

RSEDDIESEKLENAIKESVTEMIRSREKRVASGELENFGNDFLGKLVKAS

HDVDVKKRFSVDDMIDECKTFFVAGQETTNSLLSWAMLLLAIHPEWQEEA

RKEVFKLFGQGAPTSDGISKLKILHMIINESLRLYPPVVGVTRKVEREVR

VGKFTLPADLNFYISTLASHHDARIWGEDVHLFNPERFAEGVAKATRNNI

GAFFPFSLGPRTCVGNNFAMIESKITLSMILQRYSFTLSPSYVHAPIHVL

TLKPQQGVQLMLHAL

>PvCYP749A19

MDGKGMLISLLSSFLCLYLLFALIKFLHTVWWTPIRLQHALGKQGIKGPA

YKFLDGNSKEILSMRKESMSKPMNISSHYLFPRVQPHLYSWMEKYGTNVL

HWIGPQPHLFVSDTEMIREILNNKEGVYPKPDTQVFEKKLLGDGISTSKG

DKWARSRKLANHAFHGERLKSMFPDMIESSEMMLERWKAHVGKEIEVNDE

FRLLTSEVISKTAFGSSYVQGEKIFEMLNKLVEIFARNEYTVRFLSKFIK

SEDDIEADKLEKGVEESVIEMMKNREEKVISGKAESFGNDFLGLLIKANH

ESDKRNRLAVQDVVDECKTFYLAGQENINAMLSWTVFLLAIHTDWQEEAR

KEVLKVFGQGVPNSDGISKLRIITMIIHETLRLYPPATCVMRKVEREARI

GKIVLPANMNLYISALAVQHDPQIWGEDVDLFNPKRFSEGVANSVRNNMA

AFLPFGLGPRSCVGSNFATTEAKIVLSMILQRYSFTLSPSHVHSPLQVLT

ILPQHGVQVILHAL

>PvCYP749A20

MESTMGILVILLLSCPCAYLLFIFIKKVWWTPIRIQSMMRKQGIKGPSYR

SIYGSTKEIINMRKESLSKPMDLSHDMFPRIQPHVYSWVKLYGMNFLNWY

GPQAQLTVTEPEMIKEIMNNRDAQAKPESQVYVKKILGDGLVTSEGEKWA

KMRKLANHVFHGDSLKSMIPEMIASAEMMLERWKHQEGKEMEIFEEFKLF

TSEVISRTAFGSNYLQGKNIFEMLLKLAQITSRNAFKIRVPGISKILSRR

DDIESEKFAKEIQVSVMEMIKNREKKVENGEVDNFGNDFLGLLVKANHDA

DEKKRISVQDMIDECKTFYLAGQETTSTMLAWTVFLLSIHTNWQEEARKE

VLDIFGQHIPNSDGIARLKIMNMIIHESLRLYPPIASIGRKVKREVRIGK

LILPANLNLFMSTLALHHDPHIWGEDVHLFKPERFSDGIAKASKNNTTAF

LPFGMGPRNCVGFNFAATEAKIALSMILQRYTFTLSPDYVHSPFQFLTIR

PQHGIHVIFHTV

>PvCYP749A21

MMRKQGIKGPSYRSIYGSTKEIINMRKESLSKPMDLSHDMFPRIQPHVYS

WVKLYGMNFLNWYGPQAQLTVTEPEMIKEIMNNRDAQAKPESQVYVKKIL

GDGLVTSEGEKWAKMRKLANHVFHGDSLKSMIPEMIASAEMMLERWKHQE

GKEMEIFEEFKLFTSEVISRTAFGSNYLQGKNIFEMLLKLAQITSRNAFK

IRVPGISKILSRRDDIESEKFAKEIQVSVMEMIKNREKKVENGEVDNFGN

DFLGLLVKANHDADEKKRISVQDMIDECKTFYLAGQETTSTMLAWTVFLL

SIHTNWQEEARKEVLDIFGQHIPNSDGIARLKIMNMIIHESLRLYPPIAS

IGRKVKREVRIGKLILPANLNLFMSTLALHHDPHIWGEDVHLFKPERFSD

GIAKASKNNTTAFLPFGMGPRNCVGFNFAATEAKIALSMILQRYTFTLSP

DYVHSPFQFLTIRPQHGIQVIFHTV

>PvCYP749A22

MMRKQGIKGPSYRSIYGSTKEIINMRKESLSKPMDLSHDMFPRIQPHVYS

WVKLYGMNFLNWYGPQAQLTVTEPEMIKEIMNNRDAQAKPESQVYVKKIL

GDGLVTSEGEKWAKMRKLANHVFHGDSLKSMIPEMIASAEMMLERWKHQE

GKEMEIFEEFKLFTSEVISRTAFGSNYLQGKNIFEMLLKLAQITSRNAFK

IRVPGISKILSRRDDIESEKFAKEIQVSVMEMIKNREKKVENGEVDNFGN

DFLGLLVKANHDADEKKRISVQDMIDECKTFYLAGQETTSTMLAWTVFLL

SIHTNWQEEARKEVLDIFGQHIPNSDGIARLKIMNMIIHESLRLYPPIAS

IGRKVKREVRIGKLILPANLNLFMSTLALHHDPHIWGEDVHLFKPERFSD

GIAKASKNNTTAFLPFGMGPRNCVGFNFAATEAKIALSMILQRYTFTLSP

DYVHSPFQFLTIRPQHGIHVIFHTV

>PvCYP749A23

MGILVILLLSCPCAYLLFIFIKKVWWTPIRIQSMMRKQGIKGPSYRSIYG

STKEIINMRKESLSKPMDLSHDMFPRIQPHVYSWVKLYGMNFLNWYGPQA

QLTVTEPEMIKEIMNNRDAQAKPESQVYVKKILGDGLVTSEGEKWAKMRK

LANHVFHGDSLKSMIPEMIASAEMMLERWKHQEGKEMEIFEEFKLFTSEV

ISRTAFGSNYLQGKNIFEMLLKLAQITSRNAFKIRVPGISKILSRRDDIE

SEKFAKEIQVSVMEMIKNREKKVENGEVDNFGNDFLGLLVKANHDADEKK

RISVQDMIDECKTFYLAGQETTSTMLAWTVFLLSIHTNWQEEARKEVLDI

FGQHIPNSDGIARLKIMNMIIHESLRLYPPIASIGRKVKREVRIGKLILP

ANLNLFMSTLALHHDPHIWGEDVHLFKPERFSDGIAKASKNNTTAFLPFG

MGPRNCVGFNFAATEAKIALSMILQRYTFTLSPDYVHSPFQFLTIRPQHG

IHVIFHTV

>PvKO

MAFLQHFQETPFVISVALGVLSLLLYFFIKGFVSSQMGSLSKLPPPPEVP

GLPLIGNLLQLKEKKPHKTFSKWAEIYGPIFSIRTGASTMIVLNSVDVVK

EAMVTRYSSISTRKLSNALKILSSDKSMVAMSDYNDFHKMVKRHLITNLL

GTTAQKRQRNLRDTMIENTLKRLHAHVKRSPLEAVNFREIFESELFSLSM

KQTLGRDVESIYVEELASTLSKEEIFKLLVHDPMEGAIEVDWRDFFPYLK

WIPNKSLEMKIQRMNFHRKVVMKALMKEQKKQFAKGQGFNCYLHYLLSEA

KTLTEEQISMLVWEVIIETSDTTLVTTEWAMYELAKDPKWQDHFCQHIQN

ICGSDKVTEEILSQLPHIGAVFHETLRRHSPVPIIPLRYAHEETQIGGYT

IPAESEIAINIYACNLDKKQWEKPEEWLPERFLDKKYDPQDLYKTMAFGG

ESGLVLVLSRQ
