# Supplementary material for: Illuminating the biosynthesis pathway genes involved in bioactive specific monoterpene glycosides in Paeonia veitchii Lynch by a combination of sequencing platforms
Source: BMC Genomics. 2023 Jan 26;24:45. doi: 10.1186/s12864-023-09138-2 (PMC9878870; doi:10.1186/s12864-023-09138-2)
Supplement: Supplementary file 5 — Additional file 5. The full-length amino acid sequence of PvUGTs in this study. [file 12864_2023_9138_MOESM5_ESM.docx]

**The full-length amino acid sequence of PvUGTs in this study**

>PvUGT71B4

MSNNKVELVFVPAPGIGHLVSTVEIAKLLIDRDDRLSITVLIMKLSFDST

INAYTDSLAATGRIRYVHLPREDSDPSAQPGVNFLTSFAEKQKPHVKKTV

AELTRSELGSDSPRLAGFVVDMFSTCMIDVANEFGVPSYLFFTSSAASLG

LIFHLQKLTAEQNIDVTEFKNSDAELVVPSYVNPVPAKVLPSVVLDKNRG

SEEFIGLAKEFRETKGIIVNSFKELEPHALKSLSEGTTPKVYPVGPILNL

KSDAHQHAEIMTWLDDQPSASVVFLCFGSMGSFNEDQVKEIAHALERSGH

RFLWSLRKPAPANSLEFPKDYTNFDDVLPEGFLERTAGKGKVIGWAPQVA

VLAHEAVGGFVSHCGWNSTLEALWFGVPIATWPLYAEQQTNAFELVKELG

LGVEIKMDYRKGESKDVSAEEIEKGIRCVMEHDSELRKKVKDMKEKSRKA

LKDGGSSLIWLGRFTEDVLDSIP

>PvUGT71B5

MSNNKVELVFVPSPGIGHLVSTVEIAKLLIDRDDRLSITVLIMKLSFDST

ISAYTDSLAATGRIRYVHLPREDSDPSAQPGINFLTSFVEKQKPHVKKAV

AELTRSDSPRLAGFVVDMFCTCMIDVANEFGVPSYLFFTSSAACLGLMFH

LQKLTAEQNVDVTEFKNSDAELVVPYYVNPVPAKVLPSVVLDKSHGSEEF

IGLAKKFRETKGIIVNSFQELEPHALKSLSEGTTPKVYPVGPILNLKSDA

HQHAGIMTWLDDQPSASVVFLCFGSMGSFNEDQVKEIAHALERSGHRFLW

SLRQPTPANSIAFPKDYTNFDDVLPEGFLERTAGKGKVIGWAPQVAVLAH

EAVGGFVSHCGWNSTLEALWFGVPIATWPLYAEQQMNAFELVKELGLGVE

IKMDYRKGESKDVSAEEIEKGVRCVMEHDSELRKKVKDTKEKGRKALMNG

GSSLIWLGRFTEDVLDNIP

>PvUGT71B7

MNTGNKMMKKAELVFIPTPAIGHIISTVEMAKLLIDRDDRLSITVIIIKM

PHELSAITTYINSLSPSSRIRFSHLPALHFNPMEFDIRLFIDKHKPLVKE

VVSGLIQSPDSPPLAGFVLDMFNTCMMDVANEFDVPTYVFFTSGAASLGL

SFHVQAQHDEHGLNLMELEATGTELVVPSYAHPVPSKVLPAMMFEKDGPG

FVDVPRKLRPAKGIMVNTFTELESHAIKALSDGTVPKVYPVGPVLNLKGE

SQKQDDIMAWLDKQPPNSVVFLCFGSLGSFDAEQAKEIANGLEQSGHRFL

WSLRKPTRDGKFSPPTDYINLDEVLPEGFLDRMAGMGKVIGWAPQMAVLA

HEAVGGFVSHCGWNSVLESIWYGVPIAAWPMYAEQQLNAFELVVELGLAV

EIKMDYNKDECQLVSAGEIEKGVRQLMEGGSDMRKKVKEMKEKSRKALID

GGSSYTWMGRFIEDVLDNIS

>PvUGT71K1

MEKLELVIIPAPGMGHLVSTVELAKRLLDLCCGFSITVLVIKPPFVATIG

DYTKTIAASDPRIRYIELPRVDPPSPDLYKSSVEKYFSVFIESHKSHVKD

TIINLVSTESVRLVGVVVDLFCTSMIDVAHELGVPSYLYFTSGAAFLGLM

LHLPTRPDQVFKESDPDSIIPSYINPVPSNCLPSFAFNKDGYSAFSDHAR

KFKEMKGIIINTFLELESHAVESLMNDQTPPIYTVGPLLDLKCTSHSELN

RTYRDEIIPWLDDQPPSSVVFLCFGSMGSFGGPQLKEIALGLEQSGHRFL

WSVRQPPPPDKGGMPSDYTNLDEILPSGFLERTNGRGMLCGWAPQVEVLA

HPSVGGFVSHCGWNSILESVWFGVPIVTWPIYAEQQINAFEMVKELGLAV

ELKLNYRRESDVVMAAEIERALRCLMNDDKKVREKVKEMADKSRIVVMEG

GSSLTSLGRLIKDVLKNNS

>PvUGT71K2

MEKLELVIIPAPGMGHLVSTVELAKRLLDLCCGFSITVLVIKPPFVATIG

DYTKTIAASDPRIRYIELPRVDPPSPDLYKSSVEKYFSVFIESHKSHVKD

TIINLVSTESVRLVGVVVDLFCTSMIDVAHELGVPSYLYFTSGAAFLGLM

LHLPTRPDQVFKESDPDSIIPSYINPVPSNCLPSFAFNKDGYSAFSDHAR

KFKEMKGIIINTFLELESHAVESLMNDQTPPIYTVGPLLDLKCTSHSELN

RTYRDEIIPWLDDQPPSSVVFLCFGSMGSFGGPQLKEIALGLEQSGHRFL

WSVRQPPPPDKGGMPSDYTNLDEILPSGFLERTNGRGMLCGWAPQVEVLA

HPSVGGFVSHCGWNSILESVWFGVPIVTWPIYAEQQINAFEMVKELGLAV

ELKLNYRRESDVVMAAEIERALRCLNER

>PvUGT72A1

MTNSPHVAVLPSPGMGHVIPLLELAKNLVVHHNLKVTFLFITTEASPAQI

ELLRSPSLPSGLQVVDLPLVDISKIVHDDMTVVARLSIIVQESLRTLDQV

LIDLHYPKAVIIDLFCTDAIQICKKLSIPVYSFFTASTALLAFSLYLPTL

DREVECEYGELLEPIQMPGCSPIRTEDLLDQVQNRKIDEYKWYLLHVSRL

PQAAGIFVNSWESLEPVWLSALRNHSFFKKILINSPIHPVGPLIKKKEPI

TESDRACLKWLDDQPPDSVLFVSLGSGGTLTADQLTEMAWGLELSQQRFI

LVVRKPTDASGSGAFFNAGGGGEDNPISYLPEGFVKRTERIGLVIPSWAP

QVSVLRHESTGGFLSHCGWNSSLESIAHGVPIITWPLYAEQRMNATMLSE

EMGVGVRPVGVSGKGVVGREEIERVVRLLMEGDEGKVMRSKARELQRSSE

KALDIDGASYNSLSMVAEAWKANTNNKIEGA

>PvUGT72B1

METTPPRLTHVAILPSPGMGHLIPLVEFAKRLILHDNISVTFIIPTDGPP

PTVVKSTLNSLPTTAIDSIFLPPVALDDLPDGAKIETVISVTVVRSLPSL

RDVFKSLSPTALVVDLFGTDAFDVAKEFNVSPFIFFPSTAMALSLFLYLP

KLDEETGSCEYRDLPEPVKIPGSIPVHGKDLLDPVQDKKDEAYKWLLHHA

KRYSLAEGVMANSFMDLEPGPLKYLLEAKPGMPPVYPIGPLIKMDPGVEN

GSECLTWLDDQPLGSVLFVSFGSGGTLSHDQLNELALGLEMSEQRFIWVV

RSPNEKIANANFFSVQSQNDPFSFLPKGFVERTKGKGFVVSSWAPQPKIL

SHKSTGGFLTHCGWNSTLESVVNGVPLIAWPLYAEQKMNALMLADDIKVA

LKVKLNDKGLVEREEITYMVKSLMEGEEGKKLRYRMKTLKEAAAVVLSEN

GSSTKSLSELALKWKNQKRI

>PvUGT73B4

MELCKHLVHRNFKITLIIDSDISSSIPPSLQQSPLFEIADISSSPAPGSE

SQLGFQRQHQQMALGLESLLTRRRSENPDSTRPVCAIIDHIMSKTQEAFW

KFDIPTVVFITSGAAWAAIQHAAWKVQPGDMKPGETRVLPGLPEDTVLRY

ADLKQRRHWIPQRIGGGTGPNKSHDGGNSKPTRGPPFPGEPAPWVVEVEN

SAAILINTCNDLERPFLDYVANLTGKPVWGVGPLLPEQYWQSADSLLRDR

DIRPNRESNYTNDEIIEWLDSKPRGSVMYISFGSEVGPSEEEYPELAAGL

EESNRPFIWVIQRPSKNLGPGKPGSTDSSSYFPHGLDNKVGARGMVIHGW

APQLLILSHPSTGGFVSHCGWNSTVEAVVRGVPFLAWPIRGDQYYNAKLL

VSYLKVGIMVSDDYPADVVKKEDIIKGIERLVTDKEIKSRAEVLRGKFEH

GFSSKLVRFFRCLQEFHNTMRLLILCNVHVIF

>PvUGT74D1

MEKGKRSLPHCVVVPHPSQGHLNPLLQFSKRLAHKGVKVTLAVTHSIFSK

MKQGDEQLCNSITVETISDGYDQGGMSSAESLEAYLSRFRLFGSQTLGEL

LDKLHSSGCPVDCVIYDGFLPWALDVAKEHGLVGAAFFTQSGVVDCICYR

VHHGLVKLPPSPGSDNILIPGLPPLAPIDVPSFIYGEPKALFDMVMNQYE

NVDKADWVLCNTFYKLDEEAMDWMTKHLPLRTIGPTLPSMYLDKQIQDDK

SYGLSLFKPDSGVCMKWLNDKPDGSVVYVSFGSLATLQIEQMEELAWGLK

GSNNYFMWVVRPLEEVKLPKNFVEEASEKGLVVSWCPQLEVLAHKAVGCF

VTHCGWNSTLEALSLGVPMVAMPQWGDQTTSAKYLVDVWGVGLRAWRDDK

GIVRREMVEDCIREAMEGEKGKEIKINASKWRDLAREAVEEGGSSDRNID

EFVSTLTPNKDQHL

>PvUGT74D2

MGKEEKRGYKAHCLVLPYPVQGHINPMLQFSKRLEHKGVKVTLATTHFIS

KTIHGNQQSMAVETISDGYDQGAESEAESIEVYLKRFRLVGSQTLLELLE

KLRNSSCPVDCVIYDSVLPWALDVAKKFGLLGAVFLTQSCAVNSIYYHVH

QGLLKLPLSGKDVLLPGLPPLAPLEMPYFLVLPQSYPALLDLVVNQFENV

DKADWVLCNTFYQLEEKVADWLAKVWPLRTIGPTLPSMYLDKQILDDTNY

DLNLFKPDSGVCIKWLNNRPSGSVVYVSFGSLAALNVKQMEELACGLKGS

SSYFLWVVRASEKDKLPRDFVEETSEKGLVVSWCPQLEVLAHRAIGCFVT

HCGWNSTLEAISLGVPMVAMPQWTDQTTNAKYVTDVWGVGLKAWADQEGI

VTRKVTENCIREVMEGERGEDIKRNAIKWRNMARESVDQGGSSDKNIDEF

VSNLIHP

>PvUGT74D3

MGKEEKIAYKAHCLILPYPAQGHINPMLQFSKRLEHKGVKVTLATTHFIS

KTIHGNQQSIAVETISDGYDQGGISEAESIEVYLKRFRLVGSQTLLELLE

KLRNSGCPVDCVIYDSFLPWALDVAKKFGILGAVFLTQSCAVDSIYYHVH

QGLLKLPLSGREVLLPELPPLAPLEMPYFLVLPQSYPALLDMVVNQFENV

DKADWVLCNTFYQLEEKVADWLAKFWPLRTIGPTLPSMYLDKQIQDDTDY

GLNIFKPDSGVCIKWLNNRPNRSVVYVSFGSLAAPNVKQMEELACGLKDS

NCYFLWVVRASEKDKLPRNFVKETSQNGLVVTWCPQLEVLAHRAIGCFVT

HCGWNSTLEAISLGVPMVAMPQWTDQTTNAKYVTDVWEVGLRAWADQEGV

VTRKVTENCIRELMEGDRGEEIRKNAIKWRNVARESMDQGGSSDRNIDEF

VSKLIHS

>PvUGT74D4

MEKEEKRAYKAHCLVLPYPAQGHINPMLQFSERLEHKGVKITVATTHFIS

KTIHGDQQSIAVETISDGYDQGGISEAESIEVYLKRFRLVGSKTLLELLE

KLQNSGCPVDCVIYDAVLPWALDVAKKFGLLGAAFLTQSCAVKSIYYHAH

QGLLKFPLSEKEVLLPGLPPLTPLELPYFLVHPQSYPAFLDLVLNQFENF

DKADWVLCNTFYQLEEKVADWLAKFWPLRTIGPTLPSMYLDKKIQDDDLN

LFKQHSGDCMKWLSKRPNRSVVYVSFGSMAALNVKQMDELACGLKDSSSY

FLWVVRESEKDKLPRNFVEETSERGLVVSWCPQLDVLAHGAIGCFVTHCG

WNSTLEAISLGVPMVAMPQWTDQATNAKYITDVWRVGLRAWADEDGIVTR

KVTGNCIREVMEGERAEEIKRNAIKWRNVARESVDQGGSSDRNIDEFVSN

LIHS

>PvUGT74D5

MEKEEKRAYKAHCLVLPYPAQGHINPMLQFSERLEHKGVKITVATTHFIS

KTIHGDQQSIAVETISDGYDQGGISEAESIEVYLKRFRLVGSKTLLELLE

KLQNSGCPVDCVIYDAVLPWALDVAKKFGLLGAAFLTQSCAVKSIYYHAH

QGLLKFPLSEKEVLLPGLPPLTPLELPYFLVHPQSYPAFLDLVLNQFENF

DKADWVLCNTFYQLEEKVADWLAKFWPLRTIGPTLPSMYLDKKIQDDDLN

LFKQHSGDCMKWLSKRPNRSVVYVSFGSMAALNVKQMDELACGLKDSSSY

FLWVVRESEKDKLLRNFVEETSERGLVVSWCPQLDVLAHGAIGCFVTHCG

WNSTLEAISLGVPMVAMPQWTDQATNAKYITDVWRVGLRAWADEDGIVTR

KVTGNCIREVMEGERAEEIKRNAIKWRNVARESVDQGGSSDRNIDEFVSN

LIHS

>PvUGT74E1

MESQERGRHGHVIVIPYPGQGHINPMFQFSKRLASKGPKVTLLSTISDAN

SVHTHSGSITVESFSDKFDKPKNGSQDCDIEDTIDRFRVIMSKALPEFIA

KQDHPVSCIVYDAGMPWALDMAKRLGLVAASFFTQSCSVIAIYYHFHQGL

LRVPTQEEETISLPGLPPLKTCDLPSFIRDLDLYPYLLDLSLYQFLNIER

ADWIFFNSFESLEPQVVKYGMARQWPIKTIGPTIPSMYLDKRYMDDEKDY

GLNLFKPNINDCMKWLDTKETGSVVYVAFGSLAKLEEVQMVQLALGLKRI

KCYFIWVVRESELNKLPRNFLEEILEKGLVVTWCLQLDVLAHRAVGCFMT

HCGWNSTVEGLSFGVPMVVMPQWVDQMTNAKFVVDVWKVGVRVQVDEKGI

VRKEEVEKCINEVMEGERAIEIKENASMWKELAKEAVDEGGSSDKNIEEF

VASLVCNSQ

>PvUGT74F1

MDKEKKPYIGHVLALSYPTQGHINPILQFCKRLASKGVKATLATTLGITK

SMQSQPDSPVQIDAISDGGFHPADGIERYLAQLQAAGSKTLAELIIKYQK

TEHPIDCVVYDSFLPWVLDVAKQFNLVGAVLFTQPCAVNYIYYYVYHGLL

KLPVTSLPVQIPGMPVLDLGDMPSFVYLPEMYPAYFELVLNQFSNADKAD

FVLVNTFYKLEDEIIDSMSKLCPLMTIGPTIPSIYLDKRVENDNDYGLSL

FTIDQSTCINWLSTKPVGSVIYVSFGSMASLGEVQMEELAWGLKASNCYF

IWVVRASEVEKLPKNLAKEIVDKGLVVHWCPQLKVLANEAVGCFFTHCGW

NSTLEALSLGVPMVGMPQWTDQTTNAKLVEDIWKVGVRVKVNDEKKGIVP

REDIEWCIREVMEGERGKEMKKNARKWKELAIEAVDEGGTSDNNINEFIS

KLVKS

>PvUGT74F2

MDKEKKPYIGHVLALSYPTQGHINPILQFCKRLASKGVKATLATTLGITK

SMQSQPDSPVQIDAISDGGFHPADGIERYLAQLQAVGSKTLAELIIKYQK

TEHPIDCVVYDSFLPWVLDVAKQFNLVGAVLFTQPCAVNYIYYYVYHGLL

KLPVTSLPVQIPGMPVLDLGDMPSFVYLPEMYPAYFELVLNQFSNADKAD

FVLVNTFYKLEDEIIDSMSKLCPLMTIGPTIPSIYLDKRVENDNDYGLSL

FTIDQSTCINWLSTKPVGSVIYVSFGSMASLGEVQMEELAWGLKASNCYF

IWVVRASEVEKLPKNLAKEIVDKGLVVHWCPQLKVLANESVGCFFTHCGW

NSTLEALSLGVPMVGMPQWTDQTTNAKLVEDIWKVGVRVKVNDEKKGIVP

REDIEWCIREVMEGERGKEMKKNARKWKELAIEAVDEGGTSDNNINEFIS

KLVKS

>PvUGT75B1

MEHRHHFLLVTYPVQSHINPALRFAKRLLRIGVHITYAISISARRRMTRG

APTPEGLDFVEFSDGYDDGFKPSDDVGRFISELRRHGSQTLTDIIVSSAN

KGRPITCLVYTGLRLPWAAEVARVLHVPSALLWVQPATVLDIYYYYYFNE

DYKNTIINNSPLIELPGLPLLTKNDLPSFLDPSNDAHTFALPPFLEHLEA

LAKETNPNILANTFDELEPLALIAIEKLNLIAIGPLIPSAFLDGKDPSDT

CFGGDLFQCTKNYIEWLNTKPVSSVIYLSFGSISILSKQQMEEIAQGLLQ

SGRPFLWVIRAKDQNGEEEKDLQDKLINCLEELEQQGMIVPWCSQVEVLS

HPSLGCFVTHCGWNSTTESLVCGVPVVAFPQWADQGTNAKLVQDVWKTGV

RVRAAKVGGIVEAGEINRCVEIIMGGGEKGEEMRRNAKKWKDLAREAVKE

GGSSDKNLKAFVDGVVQGCY

>PvUGT75C1

MAHHHFLLITFPAQGHINPALQFAKRLIKLDAHVTFVTSISAHRQITKTT

PSLGNLSFATFSDGYDEGTKPGYDARHYMSELRRRSSEALPELIENCANE

GRPVTCLIYSLLLPWAGKVARELHIPSALLWIQPATILDIYYYYFNGYGN

VISDNIHKKDSGCIKLPGLPLLTVHDLPSHFITTPFALPSFKEHLETLCE

EANPKVLVNTFDALEHEALRAINKLSFIAIGPLIPSAFSDGEDLNDTSFG

GDLVSQSCSKNYIEWLDSKHENSVIYISFGSVSVLPKRQMEEMVRGLVDT

ALPFLWVVRVEENRDGDKEEEYKLSEDLEKQGMVVPWCNQLEVLSHKSVG

CFLTHCGWNSSLESLVCGVPVVAFPQWADQATNAKLIEDVWKTGVRMVVN

EDGVVEGCEIKRCLEMVMGGGERGEEMRRNVEKWKELAREAVKDGESSDK

NLKAFVNEVGKGGDL

>PvUGT76C1

MEKLPNTQMQESKHHYGRRLRLVLLPLPLQGHLNPMLQLASILHSKGFSI

TVIHTNFNAPNPSNYPHFTFLPISDGMSDGEATTKDIKGVIIFLSLLNLN

CIAPFRDCLSQLLSQEESVACLITDAMLHFSQAVADSFNLPRMVLRTSSI

ASFRAFAALPLLQEKGFLPIKECELESPIPELQPLRVKDIPTVNTSHEDS

IYKLIAGMVKETKASSGLIWNSYEALEESALAKLNQEFPIPSFPIGPFHK

YFPATSSSLLTPDQSCISWLDTQAPNSVLYVSFGSVVAIHESEFLDIAWA

LASSQQPFLWVVRPGLVRGYHWLEPLPSGFLEMMGGRACIVKWAPQQQVL

AHPATGGFWTHNGWNSTLESICEGVPMICLPCFGDQMGNARYVSDAWKVG

LRLENGVKRGEIEEAITRLMVEDEGKEMRERIMNLKEKVNLCLKPGGTSY

QSLESLISFILARALS

>PvUGT76C2

MEKLPNTQMQENKHHYGRRLRLVLLPLPLQGHLNPMLQLASILHSKGFSI

TVIHTNFNAPNPSNYPHFTFLPISNGMSDGEATTKDIKDVIIFLSLLNLN

CIAPFRDCLSQLLSQEESVACLITDAMLHFSQAVADSFNLPRMVLRTSSI

ASFRAFAALPLLQEKGFLPIKECELESPIPELQPLRVKDIPTVNTSHEDS

IYKLIAGMVKETKASSGLIWNSYEALEESALAKLNQEFPIPSFPIGPFHK

YFPATSSSLLTPDQSCISWLHTQAPNSVLYVSFGSVVAIHESEFLDIAWA

LASSQQPFLWVVRPGLVRGYHWLEPLPSGFLEMMGGRACIVKWAPQQQVL

AHPATGGFWTHNGWNSTLESICEGVPMICLPCFGDQMGNARYVSDAWKVG

LRLENGVKRGEIEEAITRLMVEDEGKEMRERIVNLKEKVNLCLKPGGTSY

QSLESLISFILARALS

>PvUGT80A2

MEESPGTHPRSFSASSGEVPTRLEPEITGESGSGGSELDSSGAVINGRSS

SSGTDGKILPRTNTLPVVIADTEKLGSTPSLLKLERSRTESHRPRNILAQ

EAAQIFDDKMSVQQKLKLLKRIATVKDDGTVEFEVPGDVEPQALSVGCGD

VYNEVVDDEPLDSTDLQYVPPMQIVMLIVGTRGDVQPFIAIGKRLQDYGH

RVRLATHSNFKEFVMTAGLEFFPLGGDPKVLAGYMVKNKGFLPSGPSEIP

IQRNQIKEIINSLLPACKDPDIDSSIPFNADAIIANPPAYGHIHVAESLK

VPLHIFFTMPWTPTSEFPHPLSRVKQPAGYRLSYQIVDSLIWLGIRDMIN

DVRKKKLKLRPVTYLSGSQGSDSDVPHGYIWSPHLVPKPKDWGPKVDVVG

FCFLDLASNYQPPQELVKWLEAGEKPIYIGFGSLPVQEPEKMTQIIVEAL

ETTGQRGIINKGWGGLGSLAEPKDSIYLLDNVPHDWLFLQCKAVVHHGGA

GTTAAGLKAACPTTIIPFFGDQPFWGERVHARGVGPLPIPVEEFSLPKLV

DAIKFMLDPKVKERALELAKSMENEDGVGGAVKAFLKHLPRKSLECDPEP

SSVRSSIFSISRCFGCS

>PvUGT80B1

MGNWVDHSSNGLKEGSSSSWEVGENAQTIGTEAPVEVNESPDGRLNVGEF

EEAEDRRRNSLRQKSVLEISQSKEIDVSSSPRRGLDHCITAPAGSHRTLL

MDSEDITFSRSMTEKKEALRHDLRLDRLSEREKKKLIVNLVKIQKDGTVE

VDIDKNAPVASELLELHGVEGAPINIDNTITVSNKSVPKLKIAMLVVGTR

GDVQPFLAMAKRLQEYGHHVRLATHANFRTFVRSAGVDFYPLGGDPRVLA

GYMARNKGFIPSGPGEISIQRKQIKAIIESLLPACTEPDLESGVPFRAQA

IIANPPAYGHAHVAEALGVPIHIFFTMPWTPTYEFPHPLARVPQTAGYWL

SYIVVDLLIWWGIRGYINDFRRRKLKLPPIAYFTMYNGSISHLPTGYMWS

SHLVPKPSDWGPLVDVVGYCFLNLGSKYQSREEFVQWIQKGSKPIYIGFG

SMPLEDPKKTTDVILEALKDTGQRGIIDRGWGDLGIFPEVPENIFLLEDC

PHDWLFPQCSAVVHHGGAGTTATGLRAGCPTTIVPFFGDQFFWGDRVHEK

GLGPAPIPISQLSIESLSNAIQFMLQPEVKSRAIELAKLIENEDGVAAAV

DAFHRHFPEQLPVPTASSEEDDQPNPLQWFFLQIEKWCCLPCSS

>PvUGT80B2

MGNWVDHSSNGLKEGSSSSWEVGENAQTIGTEAPVEVNESPDGRLNVGEF

EEAEDRRRNSLRQKSVLEISQSKEIDVSSSPRRGLDHCITAPAGSHRTLL

MDSEDITFSRSMTEKKEALRHDLRLDRLSEREKKKLIVNLVKIQKDGTVE

VDIDKNAPVASELLELHGVEGAPINIDNTITVSNKSVPKLKIAMLVVGTR

GDVQPFLAMAKRLQEYGHHVRLATHANFRTFVRSAGVDFYPLGGDPRVLA

GYMARNKGFIPSGPGEISIQRKQIKAIIESLLPACTEPDLESGVPFRAQA

IIANPPAYGHAHVAEALGVPIHIFFTMPWTPTYEFPHPLARVPQTAGYWL

SYIVVDLLIWWGIRGYINDFRRRKLKLPPIAYFTMYNGSISHLPTGYMWS

SHLVPKPSDWGPLVDVVGYCFLNLGSKYQSREEFVQWIQKGSKPIYIGFG

SMPLEDPKKTTDVILEALKDTGQRGIIDRGWGDLGIFPEVPENIFLLEDC

PHDWLFPQCSAVVHHGGAGTTATGLRAGCPTTIVPFFGDQFFWGDRVHEK

GLGPAPIPISQLSIESLSNAIQFMLQPEVKSRAIKLAKLIENEDGVAAAV

DAFHRHFPEQLPVPTASSEEDDQPNPLQWFFLQIEKWCCLPCSS

>PvUGT84A1

MVSDQARVHLLLVSFPGQGHVNPLLRLGKRLASKGLLVTFSAPESIGKQM

RKASNLTDQPTPVGDGYIRFKFFEDGWDEDEPKRQDLDLYLPQLELVGRE

IIARMIKKYAEEGRPVSCLINNPFIPWVSDVADDLGLPSAMLWVQSCACF

SAYYHYYHGLVPFPSESEPEIDVQLPFMPLLKYDEVPSFLHPTTPYPFLR

RAVLGQFKNLDKPFCVLMETFQELEHENIEYMSRFCPIKPVGPLFKDPKA

QTTTVRGDFMKADVSIIQWLNSKPPSSVVYISFGTVVYLKQEQVDEIAYG

LLNSGVSFLWVIKPPHKDSGFQVHVLPDGFLEKTADKGKVVEWSPQEQVL

AHPSTACFVTHCGWNSTMEALTSGMPVVAFPQWGDQVTDAKYLVDEFKVG

VRMCRGEAENKIITRDEVQKCLLEATTGPKAEEMKRNALKWKKTAEEAVA

EGGSSDRNLHQFVDEVKKRCVGITRNYKSNN

>PvUGT84B1

MVDFPTESEPERDIVLPHMPVLKWDEIPSFLHPFTREPFLRRAILGQYKN

LEKPFCVLMDTFDELEHEIIEYVSKFCPIKAVGPLFKDPKVKTTVRGDFM

KADDTIIQWLDSQPPSSVVYISFGTVVYLKQEQVDEIAYGLLSSGVSFLW

VMKPPHKDAGLELLVLPEGFLEKAGNKGKVVQWSPQEQVLVHPSTACFVT

HCGWNSTMESLTSGMPIVAFPQWGDQVTDAKYLVDVFKVGVRMCRGETEK

RIITRDEVENCLLEATTGPTAAELKKNALKWKKAAEEAGVEGGSSDRNLQ

AFVDEVRRRSVETAHMSAPSKIVEIVEPEIVESKIAEPKIVKSKLAEPEI

VEPEIVAPKIVEPEIVDPEIIEPMPLKGHPAPLEGHA

>PvUGT85A3

MMQLAKLLHSRGFHITFVNTEFNHRRLVRSKGPDSVKGLEDFKFETIPDG

LPPSDHDATQDVPALCASTRKNCLAPFLQLLAKLNSTPDVPRVTCIISDG

VMSFGIKAAEQFGVPEAQLWTASACSFMAYLHFTQLIQKGIIPFKDENCN

EVTLDTPIEWIAGMTNIRVKDIPNYINLTDPHNIMIDFMGGEAQNCLKAP

SIIFNTFDALEYEVLDAIKSKFTGNIYTAGPLISLQGSQNKPNNKVNSLE

SSLWKEDLTCLEWLDKREPGSVVYVNYGSVTVMTAQHLKEFAWGLANSKH

PFLWIVRQDVVMGDSAILPQDFLEEIKGRGMLASWCPQDRVLSHPSVGAF

LTHCGWNSMLETICQGVPVICWPFFADQLTNCRYACTTWQIGMEVNHDVK

RTEIKNLVKEIMEGEKGEGMKKKALEWKKKAEEATDVGGSSYNSIKRFIK

EALHYSE

>PvUGT85A4

MCSVAARKAHAVCVPYPSQGHVTPMMQLAKLLHSRGFHITFVNTEFNHRR

LFRSKEAASLEGFRFETIPDGLPPSDRDATQDVPALSASTRKNCLAPFLE

LLGMLNSSVDVPPVTCIISDGVMSFGIKAAQVLGIPQVQFWTASACGFMG

YLHYSELLKRGIVPFKDENFTSDGSLDAPIDWIPGMPDIQLKDIPSFIRT

TNPDDIMLNFLGGEAQNCLNAHAMIFNTFDALEHQVLEAIKYKFCSNIYI

SGPHSMLERQIPDNQFKSLRSSLWKEDKECLEWLDKREPNSVVYVNFGSV

TVMTGEQFKEFAWGLANSKHPFLWIVRPDIMDDDPVTIPREFLKETKNRG

LLASWCAQDQVLSHPSVGAFLTHCGWNSMMESICGGVPVICWPFFAEQQT

NCRYACDVWGIGLEINHDVKREEIESIVREMMQGEKGKEMKKNALELKMK

AEEATHIGGSSYNDFERFIEEALHYSS

>PvUGT85B1

MPPSDENATQSITGLLYYTKKHSPIPLRHLIEKLNSTEGVPPVSCILSDG

IMCFAIKVAQELGIPDVQFWTASTCGLMAYLQFGELVKRDIFPLKDVSYL

SNGYMNTHLDWIPGMKDMRIKDLPSFVRSTDPDDIAFNRWLEEGEDNLKA

DAIIFNTFTEFEQEVLDALAPISPRTYCVGPLSLLWKSIPQSQTKAIESS

LWKENTECLDWLDKQKPNSVVYVNYGSIAVMTDENLKEFAWGLANSGHPF

LWIVRSDLVMGGSVILPEEFFEVIKDRGMIVSWCPQDQVLKHPSVRVFLT

HSGWNSTIEGICGGVSMLCWPFFAEQQVNCRYACTTWGIGMEIDSKVTRE

EVKQLVKEMLEGEKGNKMREKALDWRKKAEASVVEGGSSFSDFNRLAEDL

MQLCLNGKYLG

>PvUGT85C1

MGSITGKKPHAVCMPYPFQGHINPMTKLAKLLHHKGFHITFVNTEYNHNR

LLKSSGPNSLDGLPDFRYETIPDGLPPTDSDRSQDTVSINAYFSAHCVVP

FRELVLKLNDHASKSDAPPVTCIVSDGSLAFPIVVSEQLGLPNAFLWTTS

VCGFMCFTQYQALIDKGLVPFKDENYLTNGDLDTIVDRVPGMKNIRLKDF

PSFIRTTDPNDFMVQVVLEDVRRAKRASAIMFNSFDALDQELLDNLSLSF

PQVHAIGPLHMLVNKIPQNPALDSIGSSLWKEDLECIQWLNSKEPNSVIY

VNFGSVTVMTNQQLVEFAYGLANSKQNFLWVIRADLVVGDSALLPPDFGE

ETKGRGLIAKWCPQEQVLNHSSIGGFLTHCGWNSTLESICCGVPMICWPF

WAEQQTNCRFVCVEWGIGMEIDSNVKRDVVEKQVTELLVGEKGKEMKKRA

MEWKKKAEEAIALSNMNLDKLVQEVLLPKHSG

>PvUGT85C2

MGSITGKKPHAVCMPYPFQGHINPMTKLAKLLHHKGFHITFVNTEYNHNR

LLKSSGPNSLDGLPGFRYETIPDGLPPTDSDRSQDTVSINAYFSAHCVVP

FRELVLKLNDHASKSDAPPVTCIVSDGSLAFPIVVSEQLGLPNAFLWTTS

VCGFMCFTQYQALIDKGLVPFKDENYLTNGDLDTIVDRVPGMKNIRLKDF

PSFIRTTDPNDFMVQVVLEDVRRAKRASAIMFNSFDALDQELLDNLSLSF

PQVHAIGPLHMLVNKIPQNPALDSIGSSLWKEDLECIQWLNSKEPNSVIY

VNFGSVTVMTNQQLVEFAYGLANSKQNFLWVIRADLVVGDSALLPPDFGE

ETKGRGLIAKWCPQEQVLNHSSIGGFLTHCGWNSTLESICCGVPMICWPF

WAEQQTNCRFVCVEWGIGMEIDSNVKRDVVEKQVTELLVGEKGKEMKKRA

MEWKKKAEEAIALSNMNLDKLVQEVLLPKHSG

>PvUGT85C3

MGSIEVANKPHAVFFPMPFQGHVTPFLTLAKLLHYRGFHVTFVNSEFNHQ

RLLKSRGPNSLDGLPDFRFEAIPDGLPPLDDLEATQDAFGLCEASRNNFL

APFRELLAKLNDSAASGVPPVTCIIADGVCTFTLDASQELGIPNVLFWTV

SACGFMGYKQIPQLIERGLTPLKDASYLTNGYLDTVVDWIPGMKSVRLRD

LPPFIRTTDPNDYMLGFTRGEAERTSKASAVIFNTLDAMEKDVLDALSSM

LPHVYAIGPLQSFINSIPDEEGTKSIGSNFWKEEPECVQWLNSKEPNSVV

YVNFGSIIIMTPEQLTEFAWGLANSNHSFLWIIRHDLVVGESAMLPKEFV

TETKGRGIIANWCSQEQVLSHPAIGGFLTHNGWNSTIESLLSGVPMVCWP

SFGDQQMNCKYTCNEWGVGMEIDNDVKRDGVEKIVRELMGGEKGKKMKEK

AMEWKKIAEEAARPDGLSSLNLDKLVNDVLLSKK

>PvUGT85C4

MLKLAKLLHHKGFHVTFVNTEFNHQRLLKSGGLNSLNGFSSSFQFEAIPD

GLPPSDPDATQNIIALCASTNKTCLTPFRQLLLKLNNTASSSVPPVTCIV

SDAAMPFTLKASQELGIPNVIFWTASACGYMGYAQFRNLLDKGFTPLKDE

TYVTNGYLDTVVDWIPGMKGIRLKDLPTFIRTTDPDDFFIHFVINEVIEK

HHGASAIIFNTFDDLEKEVLEALSSIFPPIYTLGPLQLLLNQIPDNDLNS

IKSNLWKEEPGCLEWLDSKEPESVVYVNFGSIAVMTPQQMVEFAWGLCNS

KQTFLWIIRPDLVVGESAMLPPEFVTETKNRGLLASWCNQEQVVSHPAIG

GFLTHCGWNSTLESLCSGVPLICWPFFGEQQTNCRYCCTQWGVGMEIDTD

VKRDEVENQVRNLMEGEKGREMRMKAREWKKKAEDAISCPTGSSYVNFQK

MVSRVLLPKDWNNKDNATGRSD

>PvUGT85C5

MDSIAHFGKPHAVCVPFPAQGHIGPMLKLAKLLHHKGFHVTFVNTEFNHQ

RLLKSGGLNSLNGFSSSFQFEAIPDGLPPSDPDATQNIIALCASTNKTCL

TPFRQLLLKLNNTASSSVPPVTCIVSDAAMPFTLKASQELGIPNVIFWTA

SACGYMGYAQYRNLLDKGFTPLKDETYLTNGYLDTVVDWIPGMKGIRLKD

LPTFIRTTDPDDFFIHFVINEVIENHHGASAIIFNTFDDLEKEVLEALSS

IFPPIYTLGPLQLLLNQIPDNDLNSIKSNLWKEEPGCLEWLDSKEPESVV

YVNFGSIAVMTPQQMVEFAWGLCNSKQTFLWIIRPDLVVGESAMLPPEFV

TETKNRGLLASWCNQEQVVSHPAIGGFLTHCGWNSTLESLCSGVPLICWP

FFGEQQTNCRYCCTQWGVGMEIDTDVKRDEVENQVRNLMEGEKGREMRMK

AREWKKKAEDAISCPTGSSYVNFQKMVSRVLLPKDWNNKDNATGRSD

>PvUGT88A1

MDEAIVLYPSSAIGHLISMVELGKLILTHRPSLSIHILVITFPYNSGSIT

PYINHVSATTSSITFHHLPTTSLPLDTSSSPHHETLAFEVIRLNNPNIHQ

AILSISQTHDIQALVFDFFCTPVLSIAAQLNIPAYYFFTSGAGVLASFLY

LPTMHRNTTESFKDLDINLDFPGLPPIPAADMLKPLLDRTDKAYHYFVEF

TTKFPKSDGIIVNTYESLEPRAIKAILDGVCVPDGPTPPIFCTGPLIIAG

NRKGGGSGDDCLRWLDSQPSQSVVFLCFGSLGLFSAQQLKEIATGLERSG

QRFLWVVRSPPPEEHNKHESAPPDPGLNLLLTEGFLETTRERGLVVKQWA

PQVAVLNHDSVGGFVTHCGWNSVLEAVCAGVPMVAWPLYAEQRMNRVLLV

EEMKLALPMNESKDGYVSAEEVEKRVGGMMESEEGCCIRKRIMAMKDGAK

DALSEGGSSRVALTKLIGSWKRE

>PvUGT88B1

MQQDTIVLYPAPGIGHVISMVELGKLLLHHYNHKFSITILLITDPLDTPN

TTSYIHRITQTHPSISFRHFPLVSVDTSPTTHSRAAIKFEFIRLNSTNVL

HSLNEISKVSTIKALVIDLFCTSALPIADELGIPTYYFFTSGASVLALFL

YFPVIHKQIKQSFKDLKITDILCPGLPPFPATHMPEPMLDRDDPAYDYML

YFCEHLSKSNGIIVNTFDDLEPIALKIITAGSCVPEEITPPIYCVGPLTA

NPNESTGNDCLSWLDMQPSRSVVFLCFGSRGSFSSVQVKEIANGLEASGQ

RFLWVLKIHGGEINMDLDALMPEGFLERTTGRGLVVKSWAPQVAVLNHES

VGGFVTHCGWNSVLEAVVAGVPMVAWPLYAEQHVNKAVLVEDMKMAIPLE

QREEDGWFVTADELEKRLRDLMDSDQGKELRERCWKMREMAEPAWRKFGS

SDTALAKLGEVWKHPKRFF

>PvUGT89A2

MSSSATGGAHILVFPYPAQGHILPLLDLTHQLALRNLTITILVTPKNLPI

LTPLLSANPSIQTLVLPFPSHPSLPSGVENVKDIGNRGNPLIISALSKLR

EPIIEWFKSHPTPPVAILSDFFLGWTHHLAHHLGVPRIVFFSSGAFLAAV

GEYLWRHIHTVRSLPVVTFDDLPGSPSFAEEHLPSVFRLYRESDPDWDIV

RDGMLANISSWGSVFNTFDALESEYLDYLRKKRGHPRVWGVGPLNLMGGL

ELEKRGNSVQESNSGVLTWLDDCPDDDSVVYVCFGSQKLLRNDQMAALAS

GLERSGIRFVWVVKPGTTEQMDDGFGVLPEGFEERVSGRGMVIKGWAPQV

LVLSHRAVGGFLSHCGFNSLLEGIMAGVMILAWPMEADQFVNAKLLVEDL

GVAVSVCAGEAAVPDSVELAQTISESLSGNIPQKMKAKELRERALAAAEE

GGSSARDLDGFVEELGKLEVK

>PvUGT91A1

MADSKKLHIVMFPWLAFGHMIPYLELAKLIAQKGHRVSFVSTPRNIHRLP

KLPPNLLPFITFVKIPLPRHDGNLPEGAEATSDVPYEMVQHLNNAYDALE

EPLSNFLQASEPDWLLHDFVSYWAGPMAARVGVPCAFFSIFIPAVLGFLG

PPSVLWDGIDDRYTPEEYTVSPKWVPFPSTVAYRYYEVKKIFDQMSPDDT

GVSTMQRFGGSLRRCDIILMRSCAEFDKEWLQVMEEINKKPVLPVGELPT

TTSNEEDEDDANTDIWHSTKAWLDNQPKRSVVYVAFGSEAKPTQEELTEI

ALGLELSGLPFFWVLRNRRGFADTVVIELPDGFEERTNGRGVICTSWVPQ

LKILTHESVGGILTHAGWSTVVEGFVCERALVLLPFLADQGLNARVLEEK

KLGYSIPRNKHDGLFTAKSVAESLRLVMVDEEGKIYRDNAKEMRGLFGDR

NRQERYVDNLLDYLGTHKFAKALEANGVGN

>PvUGT91A2

MAETRKLHIVMFPWLAFGHMIPYLELAKLIAQKGHRVSFVSTPRNIHRLP

KLPPNLLPFITFVKIPLPRQDGNLPEGAEATSDVPYDMVKHLNKAYDALE

VPLSNFLQASKPDWFLHDFVSYWAGPMAARVGVPCAFFSIFIPAALGFLG

LPSVLSDGIDDRSTPEEFTVSPKWVPFPSTVAYRFYEVKKIFDQASPDNT

GVSILQRFGGSLRGCDIILLRSCAEFDKEWLQLMEVITKKPVLPVGELPT

TTSNEEDDDIWKSTKAWLDNQSKRSVVYVAFGSEAKPTQEELTQIALGLE

LSGLPFFWVLRNRRGFADTEVIELPNGFEERTRGRGVICTTWAPQLKILS

HDSVGGILTHAGWSTVVEGFVCEQALIMLPFLADQGLNARVLEEKKLGYS

IPRNERDGFFTAKSVAESLRLVMVDEEGKIYRDNAKEMKGLFGDKDRQER

YVANLLDYLGTHKSVEELKQIELAKMVMYSCGASSR

>PvUGT91C1

MSETKKLHIVMFPWLAFGHILPYLELAKHIAEKGHQISFISTPRNIQRLP

KLPSSVTPLINFVNFPLPRDENLPESAEATTDLPQDKVQFLKKAFDLLQE

PMTRLLQSSTPDWIIYDFAPHWLPPIAAQLNISRAFFCIFNAWSMSFFGP

SEMLLHGNRTRKEVGDLTAPPTWFPFRSKIAYRPYEVKKFFDNVGQNVSG

VSDGFRLGSAIYGSDVVLIRSCVEIEDDWLNLFGDINRKPIIPIGLLPPS

VQDIEDNTWQEIRDWLEKHEKGSVVYIALGSEVTLTQDERTELALGLELS

GLPFFWALRNRPDLPCGFIDRIKGRGLVWTSWAPQLRILAHQSVGGFLTH

CGWSSIIEGLQLGRPLIMMPFLGDQGLNARFFDEKGVGIEIPRDEQDGSF

TRNSVVESLKMVMVEDEGKAYNEKAKELSKIFGDKERHQQYLDNFVKYFE

EYIPSQKRLISP

>PvUGT92A1

MKNTTQMDSNEHIVMVPFMAQGHLIPFLALARQIHQKSGFTVTIANTPKN

IQYLRNTISSNSNSSDSHIRLAELPFNSSDHGLPPNTENTEVLPFHQVIN

LFQASACLESSFHHLLTGIAEKEGKPPLCVIFDIFLGWANQVAESVASVG

VPFTTGGAYGTAAYISIWQNLPHRATESDEFGLPGFPDTVRFNRSQLHQY

LRAADGTDNWSKFFQPQISFSLNSHGWLCNTADEIEPLGLQILRNYIKIP

VWTIGPLLPPDLLNHSRKISPQRAGKEPGVTPEKCLEFLDLHPLGSVLYI

SFGSQNTISPSQMFELSLGLEESGKHFIWAIRPPIGFDINGPFRTEWLPQ

GFEERITKGGRGLLVHKWAPQLEILSHKSTGVFLSHCGWNSVVESLSQGV

PIIGWPMAAEQAYNSKMLVEDMGVSVELTRGGQSTIVGKEVKKVIELVMD

KKGKGGEMKKKAIQIGEQIRTAVKEEEEGQKGSSVKAMEDFLSFIISKKA

>PvUGT94AF1

MEISQDSIRVLLFPWLAHGHISPFLELAKKLSHRNFHIYFCSTPVNLCSI

KEEIDQNYSLSIQLVELHLPTLPELPPHYHTTKGLPPHLMPTLKKAFHMA

SPNFSNILKTLNPDLLIYDFLQPWAPAAALSYNIPAVQFYSTGVAATTYI

VHLCQNLGVRFPFPAIYFRENENNSFASMLKSHGVIDREGFLQCLRQSSK

LVLIKTFKAMEQKYIDFFSILCEKNVLPVGPLVQEPVEEDDQMEIMAWLD

KKDKNSAVFVSFGSEYFLSKAERDEIAHGLELSKVNFIWVVRFPVAENIS

VAEALPKGFLERVGERGMVIEGWAPQAKILRHRSIGGFVSHCGWSSVMES

IKFAVPIIAMSMHLDQPLNARLVEEVGVGVEVKRDKYGRIDKEDVAKVIK

EVVVEKSGDAVRRKARELSENMNEKEDEEIDGVVKELVKLSAARCRTYIE

>PvUGT96A1

MSQVVVVMVPFPAQGHLNQLLHLSRLLSSYNIPVHYVGSATHNHQAKLRI

HGWVPLGVEKIHFHNFNITPFPSPPPDPNAVIKFPKHLQPSFNASSSLRE

PVAALLRMLSAKAKRVVVIHDSMMASVVQDVVSIGKAESYTFHSASAFAV

YMFILEASGKPFNKFIPKDIPSLEGCFSKEFLNFMAYQYEYSKLSSGKIY

NTCKLVEGPYIDLLAKETCGGSNKLWAIGPLNPVAIKGTKHPHKCLEWLD

KQAPNSVIYVSFGTTTTLPDEQIKELAIGLEQSNQKFIWVLRDADKGDIF

DGGEVRIAQLPKGYEGRVKEMGLVVRDWIPQLEILGHSSTGGFMSHCGWN

SCMESISMGVPIAAWPMASDQPRNTVLLTQVLKLGIVIQEWSRREEIVTS

STVEKAVRRLMDSEEGKEMRKKAAEMGEAIRRSMDEGGVSRMELDSFIAY

VTK

>PvUGT96A2

MSQVVVVMVPFPAQGHLNQLLHLSRLLSSYNIPVHYVGSATHNHQAKLRI

HGWVPLGVEKIHFHNFNITPFPSPPPDPNAVIKFPKHLQPSFNASSSLRE

PVAALLRMLSAKAKRVVVIHDSMMASVVQDVVSIGKAESYTFHSASAFAV

YMFILEASGKPFNKFIPKDIPSLEGCFSEEFLNYMAYQYEYSKLSSGKIY

NTCKLVEGPYIDLLAKETCGGSNKLWAIGPLNPVAIKGTKHPHKCLEWLD

KQAPNSVIYVSFGTTTTLPDEQIKELAIGLEQSNQKFIWVLRDADKGDIF

DGGEVRIAQLPKGYEGRVKEMGLVVRDWIPQLEILGHSSTGGFMSHCGWN

SCMESISMGVPIAAWPMASDQPRNTVLLTQVLKLGIVIQEWSRREEIVTS

STVEKAVRRLMDSEEGKEMRKKAAEMGEAIRRSMDEGGVSRMELDSFIAY

VTK

>PvUGT709H1

MSPHVLIFPFPIQGHVNSMLKLAELLSIAGLHVTFLNTDYIQSRLLNNTD

IQSRFQGYPGFRFETISDGLPDDNPRSGDRIVELFNSINATTVPLFREMM

KSGRLSSNGLPVTCLIADGILSFAIDAAKEIGIMSISFRTISACCFWTYF

CTPHLVEAGALPFKGNDNLDELVTGVPGMEGFLRRRDLPSFFRVCNLDDQ

SFQFVISETRKTPEAHGFILNTFEELEAPILAHIRSHCPNLYPIGPLHAH

LKSRQLGSSSSQSSNSLWKEDRNCMTWLDAQPLKSVIYVSFGSMVMCTRE

QIIEFWHGLVNSNKLFLWVIRPDSVTGKDGRSQIPAELSAGTKDRGYMVE

WAPQEEVLAHPAIGAFLTHSGWNSTIESIIAGVPMICWPYFADQQVNSRF

VSEVWKVGVDMKDMCDRVTVEKMVKDVMEVRRDELYRSADKTTKLATGAV

SLGGSSHSNLDRLIQDIRLMRVARVPTELSGTC

>PvUGT709K1

MERQPHVVILPFPAQGHIKPMLQLAEALSQAGIRITFVNSQHIHDRILTV

MDIKSFYNRFPGFQFVSLPDGLLLPKAPGLDLNQLVTQMFLSTVSVTGPL

FRELMISSIGKESGSTCIIADGIMSFAIDVADEFDIPIISFRTYSASCTW

LYFHIAKLIEQGEIPFQEKDLDRSITCIPGFEDFLRCRDLPHFCRFQEVD

NPMTQFYLNQASAMRRASALILNTFEELEAPAISGLRPFFPKIYSIGPLN

TQESSDSSHGSLRKQDKSCMAWLDSQPSKSVLYVSFGSAVRLSRDQLLEF

WYGFVYSGKRFLWVIRPDLITGDDVGPTPAELVEGTKERGFIVEWAPQEE

ILAHPAIGGFFTHSGWNSTLESIYAGVPMICWPQIADQQVNSRCVSELWR

IGLDLKDTCSRGMVEKSVRDFMEDKREEIMRSVDKIAGMARQSVTESGSS

KCNLNKLIDDIRSMC
