# Supplementary material for: Illuminating the biosynthesis pathway genes involved in bioactive specific monoterpene glycosides in Paeonia veitchii Lynch by a combination of sequencing platforms
Source: BMC Genomics. 2023 Jan 26;24:45. doi: 10.1186/s12864-023-09138-2 (PMC9878870; doi:10.1186/s12864-023-09138-2)
Supplement: Supplementary file 6 — Additional file 6. The full-length amino acid sequence of PvBAHDs in this study. [file 12864_2023_9138_MOESM6_ESM.docx]

**The full-length amino acid sequence of PvBAHDs in this study**

>PvBAHD1

MAIEAEKNQQVSKVKVTGKSHIKPNKNLGRHECQLVTFDLPYLAFYYNQK

LLFYKGADFDDMVNKLKDGLGVVLEEFYQLAGKLGKDEDGVFRVEYDDEM

EGVEVLEAAADEITIADLMVEEGSGTLMKELVPYNRILNLEGLHRPLLAV

QFTKLNDGLVIGCALNHAILDGTSTWHFMSSWAEICRGANSISVPPFLDR

TKARNTRVKLDLVLPSTDAAPSNGDATSLREKIFRFSEETVDKIKSIVNS

NPPSDGCKPFSTFQSLSTHIWRHVTHARKLKPEDYTVYTVFADCRKRVDP

PVPDSYFGNCIQAIFTVTAAGLLTGNPPEFGAATIQKAINAHDAKTIDGR

NKEWETAPKIFQFKDAGVNCVAVGSSPRFKVYEVDFGWGKPESVRSGSNN

RFDGMVYLYQGKSGGRSIDVEICLEAEAMEELEKDKEFLMQV

>PvBAHD2

MAIEAEKNQQVSKVKVTGKSHIKPNKNLGRQECQLVTFDLPYLAFYYNQK

LLFYKGADFDDMVSKLKDGLGVVLEEFYQLAGKLGKDEDGVFRVEYDDEM

EGVEVLEAAADEITIADLMVEEGSGTLMKELVPYNRILNLEGLHRPLLAV

QFTKLNDGLVIGCALNHAILDGTSTWHFMSSWAEICRGANSISVPPFLDR

TKARNTRVKLDLVLPSTDAAPSNGDATSLREKIFRFSEETVDKIKSIVNS

NPPSDGCKPFSTFQSLSTHIWRHVTHARKLKPEDYTVYTVFADCRKRVDP

PVPDSYFGNCIQAIFTVTAAGPLTGNPPEFGAATIQKAINAHDAKTIDGR

NKEWETAPKIFQFKDAGVNCVAVGSSPRFKVYEVDFGWGKPESVRSGSNN

RFDGMVYLYQGKSGGRSIDVEICLEAEAMEELEKDKEFLMQV

>PvBAHD3

MISKKPSFMVFPAKPTPNVRLPLSECDQRHAWTHTPMIYFYKPFNNYHIS

SAIDTVAEALSRALVLHYPVAGRLHGIQGGRFELHCNAMGAQLSEAICEA

NMVDKYGDFTPTREMHLELFPSIDYNTTPIEMLPLFLVQLTKFGCGGLCL

GTLTNHTLFDGWGAINFINSWASFARGEEKLNVKPCYDRRLLQGNGSAPR

FRPIECEPLPLMNGCSDVKAEARMETKVAMLKLPKNLVEAIKKKSNDQGQ

NGDRRPYSTFEAITAHVWRCACKARAHDSDQLTMIRFMIDIRNKLQPPLP

PGYFGCSALPTVTPTCFSGDIVSRPLSYAASKIREGIERMTNEYIRSTLD

FLKSQGDMSELRTSFHTAGNNQGFFLGNPNLSCTSLTQFPVYDADFGWGK

PIHMGPGLVTSDGKVSILPAGPNGDGSLKVIVGLQSRHAYSFKMFFYRDL

IQEDAKM

>>PvBAHD4

MELVLPTSLVFAVTRHKSELVGPAKPTPKESKELSDLDDQEGLRFHTPIV

LFYGNNPAMKGRDPVKVIREALAEALVFYYPLAGRLREGPGRKLIVDCTG

EGVLLIEADAYDVTLEQFGDPLHPPFPCLDQLFCATPTPEEFLKSPMLLF

QVTRLKCGGYILTVLLNHAMCDGAGFGQFLIAAGEMARGASAPSVPPVWQ

RELLSARNPPRITCVHHEYDEVVDTKGTLAPHDDMVQHSFFFGPTNVNTL

RRLVPPHLSKSSTFELIASCMWRCRTIALQFHPDEVVRFACNVNARGKFD

PPLPAGYYGNVIASPAAKSTAGMISQKPLGYALELMRSAKNKVNMEYMRS

VTDLMVLKGRRPQFAAVNSYVVSDLTRARFRDADFGGGIANYGGPAIGGI

PEWTSFYTTFKNKRSGENGFVVAICLPAVTMKRFIVELDIMLDDGQGVIR

SAL

>PvBAHD5

MALAPPQNLFFTVTRRQPVLVVPAKPTPKDSKELSDLDDQHGLRHHMPIT

QFYGNNHAMAMKGSDPVQVIKEALAETLVYYYPLAGRLREGPDGKLTVDC

TGEGVVFTEAYADVSLDQFGDALHPPFPCLEELLPNIPNAGDHLKCPLLF

IQITRLKCGGFILAIQFSHVVCDGTGIVKFMMAVAEMARGASAPSVLPVW

QRELLNARNPPRITCTHHEYNQMADTKDALASHHDIIVQHSFLFGPTIIS

TLRKLVPSHLRACSTFELIAACLWRCRTIALQIDPAHKVRFGCVIDARTK

FIPPLPSGYYGNAIVSPAALTTAGELYQKSLGYALELIRNAKKEVTQEYV

KSVADLMVLRGRRPQIDTVNSYIVSDGSRAGFNEVDFGWGKAAYGGPPIG

AVSEVVSYYLSYKNKNGENEFVVPILLPATAMKRFIVEMDIVLKDRLPKS

SL

>PvBAHD6

MLRSEKELPDCLYSDQPVLISPNRPTPTHLLYLSNLDDQKFLRFSIKYLY

LFRKSVSFNTLKCSLSKLLVDYYPLAGRLRTNTDDGQKLEVDCNGEGAVF

AEAFMDFTADEFLEFSQKPNRSLRKLLYRVEARSFLDIPPLVVQVTNLRC

GGMVLCTAINHCLCDGIGTSQFLRAWAEIITKPNDDVLITPFHERLLLKP

RNPPQITLTHPGYTLKDNTQIDLNQYLKSQPLVPTSFTFTSCHILELKKQ

CVPSLKCTTFEVLASHTWRSWVKSLGLSPSLNVKLLFSMNVRKRVRPEIP

QGYYGNGFILGCAESTPKELVNANLHNGVKLVQKAKSSVNDECVKSIIDL

LEDITVKTDLHASLVISQWSKLGLEDLDFGEGKPLHMGPLFSDIYCLFLP

VIDDFDAVRVLMSMPENAVKKFEYYMTDFEGANDYQEEENGLL

>PvBAHD7

MAPPPTNLVFTVKKGEPELVAPAKPTPREFKELSDIDDQEGLRFQIPVIQ

FYRNDPSMRGRDPVKVIREAIAQTLVFYYPFAGRLREGAGRKLVVDCTGE

GIMFVEADADVSLEQFGDALQPPFPCLEELLYDVPGSGGVLNCPLLLIQV

TRLRCGGFIWALRLNHTMCDASGLVQFMTAVGEFARGAGAPSIPPVWKRE

LLNARDPPRVTCTHHEYDEVADTKGTIIPLDDLAHRSFFFGPTEISALRR

FLPPQLRGCSTYEILTACLWRLRTVSLQPEPTEEVRVLCIVNARTKFNPP

LPSGYYGNAFAFPVALTTAGKLCQNGLGYALELVRKAKNDVTEDYMKSVA

DLMVLKGRPHFTVVRSYLVSDVTHAGFEEVELGWGKPAYGGPAKGGVGAI

PGVASFYIPFKNSGGENGIVVPICLPAVSMERFVKDLDCMLRGHPTNRST

STFNIISAL

>PvBAHD8

MPSSSTVIVSKCTVYPNHKSEINTLKLSVSDLPMLSCQYIQKGVLLTRPP

HNIDYLISLLKHSLSQTLTHFPALAGRLITDTDGYVHILCNDAGVEFIEA

RAKHLYITDILSPIYVPNSFKEFFAFNGAPLSYSGHFKPLTGVQITELAD

GLFIGCTVNHAVTDGTSFWHFFNTFAEICKGAKKITNSPDFARNSVFNSP

AVLKFPDGGPKVTFSGDEPLRERIFHFTRDSILKLKQKANNTSSRKTITS

SEEYGKQRHDSWRTGKITSAVETLMKSQTAEISSFQSLSAQLWRSVTRAR

NLHPSKTTTFRMAVNCRHRLEPKLDAYYFGNLIQSIPTVASAGEVLSRDL

SWSADQLHKNVVAHSAATVRRGVEDWESNPRLFPLGNFDGAMITMGSSPR

FPMYDNDFGWGRPLAVRSGRANKFDGKISAFPGREGGGSVDLEVCLAPET

MAGLENDAEFMQYVSGSSV

>PvBAHD9

MAVSLKSSFMVIPAEPTPTGRLPLSECDQRHPWTHAPTVYFYKPANNCPI

NYSIETLTEALRAVLVHFYPVAGRLRRIEGGRFEVECNAMGAQLSEATSE

EDLDMYGDFTPTGEMQKLLPSVDYSNTPIEMIPLFLVQLTKFPCGGLSVG

VLMSHTLVDGVSAIHFINSWASFARGGDKLQVEPYLDRKILIEKYGEPSF

PHTEYNPLPLLIGCSDESREAKKETKVAMLKLTKDQVEELKKKANQGQSD

HDPRRGHPYSKFEAVSGHVWRCACKARAHTSDQLTMIRYMVDSRNRLQPP

LPPGYFGGTALPTVTHRCLSGDLVSEPLGYAAHQIRQGTEMMTNEYIRSA

LGFLAREEDISKFRTSFHIVGTSQGFFSGNPNLSITSWASMSLRDADFGW

GKPIHMGPALVSSDGKVFILPGSVGDGSLTIALRLQCQHMDSFKNFFYQD

LNANA

>PvBAHD10

MAVSLKSSFMVIPAEPTPTGRLPLSECDQRHPWTHAPTVYFYKPANNCPI

NYSIETLTEALRAVLVHFYPVAGRLRRIEGGRFEVECNAMGAQLSEATSE

EDLDMYGDFTPTGEMQKLLPSVDYSNTPIEMIPLFLVQLTKFPCGGLSVG

VLMSHTLVDGVSAIHFINSWASFARGGDKLQVEPYLDRKILIEKYGEPSF

PHTEYNPLPLLIGCSDESMEAKKETKVAMLKLTKDQVEELKKKANQGQSD

HDPRRGHPYSKFEAVSGHVWRCACKARAHTSDQLTMIRYMVDSRNRLQPP

LPPGYFGGTALPTVTPRCLSGDLVSEPLGYAAHQIRQGTEMMTNEYIRSA

LGFLAREEDISKFRTSFHIVGTSQGFFSGNPNLSITSWASMSLHDADFGW

GKPIHMGPALVSSDGKVFILPGSVGDGSLTIALRLQCQHMDSFKNFFYQD

LNANA

>PvBAHD11

MAVSLKSSFMVIPAEPTPTGRLPLSECDQRHPWTHAPTVYFYKPANNCPI

NYSIETLTEALRAVLVHFYPVAGRLRRIEGGRFEVECNAMGAQLSEATSE

KNLDMYGDFTPTGEMQKLLPSVDYSNTPIEMIPLFLVQLTKFPCGGLSVG

VLMSHTLVDGVSAIHFINSWASFARGGDKLQVEPYLDRKILIEKYGEPSF

PHTEYNPLPLLIGCSDESMEAKKETKVAMLKLTKDQVEELKKKANQGQSD

HDPRRGHPYSKFEAVSGHVWRCACKARAHTSDQLTMIRYMVDSRNRLQPP

LPPGYFGGTALPTVTPRCLSGDLVSEPLGYAAHQIRQGTEMMTNEYIRSA

LGFLAREEDISKFRTSFHIVGTSQGFFSGNPNLSITSWASMSLHDADFGW

GKPIHMGPALVSSDGKVFILPGSVGDGSLTIALRLQCQHMDSFKNFFYQD

LNANA

>PvBAHD12

MAPPTSLQLTVTRREPELLAPAKPTPHESKELSDIDDQRGLRFQVPIIQF

YRDSPLMKERDPVKVIREALAQTLVFYYPFAGRLREGPGSKLIVDCTGEG

VLFVEADADITLGQFGDQLEPPFPFFGELLYDVPGSGDILNCPLLLIQVT

RLQCGGFILGIRFNHTMSDGYGMAQFIVALGEMARGAHTPSIQPVWCREL

LNARNPPHITCTHPEYDEVTDSKYKLLPRDDMVRRSFFFGRAEISRIRRF

LPDHLISCTSFEVLTACLWRCRTRALQLDPTMDVWMIFTINVRSKLNPPL

PSGYYGNAIAIVAAFTSAGELCRNPLEYALELVRKAKNYVNQEYMKSVAD

LMVLKCRPHFTVVLGSYGVSDLRWAGLEGVDYGWGKAVYGGPAKGEIGDV

PGFANFYLRSPKKNRDDGILVPICLPAPVMEIFVKELDIML

>PvBAHD14

MVSLKSSCMVFPSKPTPNGFLQLSECDQLHPWTHIPIVFFYKPDTSNYPI

KYSIETMREALEQVLVHFYPVAGRLAPIEGARFEIECNAMGVQLTEATSE

ADMDKYGGDYTPTKEMQELLPSIDYNNTPIEMLPLFFVQLTKFRCGGLCV

GVKISHTLGDGMAIMHFINSWGKFARGDEKLEVKPFLTRTVVREKCCVTR

FPHMEFSQPPPLNGCRDGNAVGKKETTVAMLWLTKDQVQRLKNKANLEAQ

NGRRPYTRYEAVTGHMWRCACKARAHDYDQATRIRFMVDCRNRLQPPLPP

GYFGSCALPTVTPTCLSGDLVSKPLGYASSKVREGTEKMTDEYIRSALDF

ARSQGDVSRLRTSFHTVGTSQGFFSGNPNLNVTSWTGLPVYDADFGWGKP

VYAGPGLVNSDGKSFIVSGCDGSLAVALRLQTEHMDIFRKYFYEI
