# Supplementary material for: Illuminating the biosynthesis pathway genes involved in bioactive specific monoterpene glycosides in Paeonia veitchii Lynch by a combination of sequencing platforms
Source: BMC Genomics. 2023 Jan 26;24:45. doi: 10.1186/s12864-023-09138-2 (PMC9878870; doi:10.1186/s12864-023-09138-2)
Supplement: Supplementary file 8 — Additional file 8. The nucleotide sequence of unigenes from SMRT sequencing. [file 12864_2023_9138_MOESM8_ESM.docx]

>transcript_0 full_length_coverage=2;length=9418

GGGAGCGAGAAAAGAAACGAAAACAACTGAGGAAAACTCTGTTTCTCTTACTTCTTCTCTTTGTATTATTCACTTTTTCGCTGGGGACTCCGTATCGATCCATCGGGAAAGATTAGATTCCGGTAGGTTAGTCTCTCGCCGGCAGGTCAGTGATTTAGGGTTTATTTGTCGGAAGGTAGTCACTTTCTCTTTCTTTCTTTGATTGTTTTGATGAGAGTAACCCTAGAGTCGTCAACAAGCTGATGACCGTACGATTGGTGTTCTGTTGATCAACGGCCAGGATTTCTTCACCGGATGGAGGGAGGTCTACACCCATGGATTATGATGACAATGATTTTCACAGCCAGAATCCTCAGTTAGCTGGTGAAGGGAGCACCAATTTTTCTCCTGTTTTACGGCCATATGCTCTTCCCAAGTTTGATTTTGATGACAGCCTCCAGGGACATTTAAGGTTTGATAATTTGGTTGAAACCGAGGTTTTTCTTGGTATTGAAAGTCAAGAAGACAACCAGTGGATCGAGGATTTCTCTCGGGCAAGTAGTGGGATAGAGTTCAGTTCAAGTGCAGCAGAATCTTGCTCTATATCAAGACGCAACAATGTTTGGTCTGAGGCTACATCCTCGGAATCTGTTGAAATGCTATTGAAGTCTGTTGGGCAGGAAGAAATGATTCGGGGACAAACTATAATTGAAGAGCCTGATGCAGGTGATAAATTGGGCTGCTCAACCAAGCCTATGGAGTCTAGTTTGAAACATGATGATGGATCTCTCTCTAGACTTGGGGCTGTTTCAGGTTCTTCATTGTCACCAGTTGATTTTCTGGAATCATTGTCTGGATTAAATGAGGATTCAGGAGGCGCGCTGCCTCAGGTTGAAGATACTATACAAGCTCATGAAGGTGAGCTATCTGCTTATGGAAGTTCAAGTGATTTGGATCCTATTGCTGGTGGTGAAAACTGTAACTTACCTGTGACTCAGGGGAATCTATTAATAGTTGGTGATTCCAATGTTGAAAAACAGAGGGAAGTTGATACTTCAATTGATGATACTTTTGATAACAGAACACAGGAAGATTCTTCTGCTTCTGGGATGCAAATAGATCACGTGGTCACATCTACACAGATTATTGATACCAGCGTTGGGCAAATTAACATTCAAGAGGATGTTCAGGCAAATGATATTAGTGATGAGGATGTAGTTGGTTTAAGTAAAGACATTGGTGACAGAGGGGAGGAACACCATGTTCCGAACAAAGAGGTTCAAATGAATGATCAAAATCTGGTGGGACATGCAGTTGAAAGTGATGCTTATAATTTGGGTCCACATGCTGTTGGTGAAAAACATGACTTGCCTCAGGCCGAGGAGAATCTTTTAAGAAACAGTGAATGCAATGTTGTAAACCAGACAGAAGTTGATGATTCATCCAGTAAATCTCTGGATAGCAGAACACAAGAAGATTCTTCTACTTCCAGGATGCAGGCTGAATATCCATCCACATCGATATTGAATATTGACGCAAGCAATGCTATATTGAACAATCATGAAGATGCTAGTTATGAAAATGCAGACAGTTCAGGTAAAGACATTGGGAACGGAGGGGAGGAACACCATGTTCAGGGTAAAGAGGTTCATATGAATGATCAAAATATGGGGCGACATTCGGCTGGTGCCTATTATTTGGATTCAAGTGATTCAGATCCAAATGCTGATGGTGAAAAAAGGGACTTACCTGTGGAGGTGAATGATGTTAGTGATGAAAATGCAGACAGTTCAGGTAAATACATCCGTTCAAGTGATTCAGATCCAAATGTTGATGGTGGTGAAAAAAGGGACTTACCTGTGGAGGTGAATGACGTTACTGATGAAAATGCAGACAGTCTGGTTAAAGACATCTATTCAGTTAAAGACATCGGTGATGGAGGGGTGGAACACCATGGTCAGAGTAAAGAGATTCACACGAATGATCAAAATGTGGAGAAGCATCCAGGAACAGCATGTTCAGGTTCAGTTAAAGATATCGGTGATGGAGGGGAGGAACACCATGGTCAGAGTAAAGAGATTCACATGAATGATCAGAGTAAAGAGATTCACATGAATGATCAGAGTAAAGAGAGCCATCCAGTTGAACGTGATGCCTATTATTTGGATTCAAGTGATATGGATCCAAATGTTGATGGTGAAAAGAGAGGCTTACTTGTGCAGGTGAATGATGCTAGTGATAATGCAGATAGTTCAGGTAAAAACACTACTAACGAAGGGGAGAAACACCACCTTCTGAGTAAACAGATTCACATTACCGACCAAAATATGGTGAAACATCCATTTCAAAGTGGTGCCCATAATTTGGATCCAAATGATCCGGATCCAAATACTGATGGTGAAACGAGTGGCTTACATGTGCAGGTGAATGATGCTGGTGATGAAAATGCTGATACTTGTAGTAAAGACATTTGTGATGGAGGGGAGGAACCCCATGTTATGAGTAACGAGGTTCACATTAACAATCAAAATATGGAGAGGCATGCATTTGAAAGCGGTGCCTATTATTTGGATCCAAATGCTGATGGTGAAAAAAGTGGTATACCCGTGAAAGTGAATGATGCCAGTGATGAAAATGTAAATAGTCCAGGTAAACACATTGCTGACAGAGGGGAGGAGCACCATGCTTTGATTAAAGAGTTTCACATGAGTGACCAAAATATGGGGAGACATCCAGTTGAAAGCAGTGCCTATAATTTGGATCCAAATGCTGATGGTGAAATAAGTGGCTTACCTATGCAGGTGAATGGTGCCAGTGATGGCAATGCAGATAGTTCAGGTAAAGACATTGGTGACGGAGGGGACCATGTTCTGAGCGAAGATGTTCACATTAATGATCAAAATATGGAGAGGCTTCCATTTGAGAGTGGTGCCTATAATTTGGATCCAAATGCTGATGGTGAAAATATTGGCATGCCTGTACAGGTGAATGATGTCAGTGATGAAAATGCTGATAATCTAGGTAAAGACATTGCTGACAGAGGGGAGGAACGCCATAGTTCGAGTAAAGAGGTTCTCATGAACGATCAAAATGTGGAGAGAGATGCAGTTGAAAGCATTGTCTATAGTTTGGATCCAAATTGTTCAGATCAAAATGTTGATGGTGAAAAAGGTGGCTTATCTGTGACCGAATGCGATGTTGCAAATCAGATGGAAGTTGATATGACATCTGATGTATCTTTGGATAACAGGGCACAAGAGAATTCTTCTGCTTCTAGGATGCAAGTTGATCATCTAGTCACTTCTGCACAGGATATCGGTACAAGCATTATGCAGTTGAACAATCAAGAGGCCGTGCAGGTAAATGATTCTAGACCTTCTAGCGATGAGAACGAAGATGGTTTACTTAAAGGCAACGGTGAAGGAGGGGGGGTTCAACCTGTTCTGTGCGAAGTTGTTCAAATGAATGATCAGAATGAGGAACATGCAGTTGAAAATGGTGCTTATAATTTGGAGACCATTCCCAGTCTGGCCTCCAGGGTTAAATCTACCGGAGAACGACCTGCACTTGAAACTAGAATCAGTAATGCGGAGGAACCTTCTAGTATGATAATGAAGGGGGATCCTGCCTCACAGATTGTGGAAGGATGCAGTGAGGATACATGTGCATCGATGCCTGTTCAGGTCAGCAAATGTGAAGTAGTGACTTCGTGCAGAGACACAGAGTCGTGTGATCAGTTTAAACAAATCATGCATGAGAAATCACCCATAGCTTTGGGAGGTGATAACAGTTATAAGGGGAATGCCGTTGAGTTCAGCAACACGATATTGGGGATATGTGCTAGTCCAGGTCTGAAGAAGGATACTCTTGCAGAGATGTCATATGGGCAGGGTTCTGTTGAGAAAAGAGAGGATTTGTTAGAAAGAGGAAATGAAAAGGATAATGTTCCAGTCCGCAATTCTGAAGCATCTCTGTTATCTGTAAAAGGTGGCAAACCTTCTAAAGATCAATGTGATGGAAGCAGTTATCGTCAAGTGGGAGATATATCTAGTGTAAAGGTGGTTTTTTCTTCAGCTGAATTGCTTACGGAAACACATGCAACTAAACATTGTAAAGGTGTTCTTGATCCTTTTGGAGTTCGCAGAGAGGACTCAAATAGTGAAGACCTTGTCTCATCTTCTACTGTGGCAAAATCCATGGTATATGAAGATGATGTAGTCTCCCGGCAAGGTGTTGATGGTAACTTTGACCGAGATGTTTCTGTTATCGAAAAGGAGAATTTACAGTTGCCCACGGATTCTAGTGATGTGGGTTGTGAGATCGCCAGTTCCCTTATCATTCATAATAAGGCTGGATCCTCGTCTCCAGGTGACGAAACCCTAAATGTAAATGATAAAGAGGCGTCTCTAAAAGTTGCAGAGTCGATTCTAAACAAAGGCGATATGCTCACACAGCCTGTGCCGCCTTTAGAGGACTCATCCGATTTTGGCCAAATAGGTCAGAAAGATAGTGAAGGTATATTGGAACCTGCTGATAATAATTGCGGGCTGATTCCTATCCCAAGCAGTGAAGGTAATGCTTCATGTGCCCATGATGGTTCTTTGAGTTCTGTTTGTATGTCCGAATGTGAAGATAAGGTTCGGGTGCTGGAGGGTGGAAGTGTTTATGCAGATTCTGACAAACCTAATTGTGGATCTCCCACTGTTATTAGTTGCACTGAACTTTGTAAAGAAAAAGAAAATCAGCAGGGAGTCAAGGGGCCTTTGGATCAGATTGCCCCACATTCTAATATCAATGATGGTTCTGCCAAAAAAGTGTGCTCTACTTCCCAGGATATGAAAGAAGATGATGCCTTAGACGAGAAGAGTTTTTCTTTTGAAGTAAATTCATTGGCTGACTTGCCTGGAAGAGAGTCTGGCAAGGGTTGGCAGCCATTTACCACTATTCAAGATCCCAAAGTATCCATGATTGTAGAGGGATCTCCTACGATATCTGGTTCAGGCCAAATTGATTATAAGTTGGCACAAGAAGTTCCCCATGCAAGTCCTCGAGCGTCTAATGGAGTGAATATAGTTGGTGGTTCCAGAGCTACTCCTGAGCGTAAAACAAAACGAGGGTCTGGTAAGGCAGCAGGGAAAGAAAGTGCTAAGAAAGGAAATCGTACAAAAGAATTAACTCCCATGAAACAGCAACTAGACAGAGTTGAAAAATCATGTGTTGTGTTGCCGAGCCCACCTGGGACTTGCCTGCTTGTGCAATCCAAAGAGATGCAGCACTTTGGGATTATGGAACGCAATAACAAAAAACCATCTAATGCTTTTACTGCGACATCTGGTCTGCCAGATTTAAATACTTCAGCTTCTCTACCGGCAGTGTTTCAACAGCCTTTTACAGACATGCAACAAGTGCAACTGCGTGCTCAGATCTTTGTTTATGGATCTTTGATCCAAGGAGCAACACCTGACGAGGCATGTATGGCATCTGCTTTTGGGGACTGGATGGTGGAAGGAACATTTGGGAGAACGCATGGCGCACATCTATAGAAAGGCTTCACGGTCAAAAATTTCATCCCAGTACCCCTGAAACCCCTTTGCAGCAACGTTCAGGTGTTAGAGCTTCAGATCAAGCAGGTAAACAGGGCACACTTCAAAATAAAGTCATCTCTTCACCTGTTGGTCGAGCTAGCAGCAAGTGTACTCCGCCACCAGTTGCAAATCCTATGATACCTCTTTCATCACCACTTTGGAGTATTTCCACCCCATCATTTGATGGTATGCAATCTAGTGGCATGCCAAGAGGTTCAGTTATGGAACATAACCATGCAATTTCACCATTGCATCCTTTTAAGATCACACCTGTAAGGAATTTTGTTGGGCATAATGCTTCTTGGTCATCTCAGGCCCCTTCTCCTTGTTCTCGGGTTGCTTCTCCACAAACATCGGCCAGTTCCCGTTTTTCTGCATTGCCTATCACAGAAACAGTTAAATTAACTCCTGTTAGGGAATCATCCGTACCACTTGCCTCTGCTTTAAAGCTCGTATCTCCTAGTACTATTGTTCATAGTGGGGATCCTACTACTGTTTTTCCAGGGACTGCTTCCTTGCTTGATGTGAAAAAGGCAACATCATCACCTAGCTCAAATTCTGCAGATCCAAAGCCAAGGAAGAGAAAAAAAGTCCCAGTTCCTGAAAATCCTGGTCTTATCTCTTTGGCACCTCAAACTCGAATAGAACCAGTTTCTTTGACACCTCAACCTCGAACAGAACCAGTCTCTTTGACACCTCAACCTCGAACAGAACCAGTCTCTTTAACAGCTCGAACCCGTACAGAACCAGTCTCTTTGACCCCTCGAACTCGAAGTGAACCAGTTTTTTTGACCCCTCGAACTCGAGTAGAACCAGTCTCTTTGACACCTTGGACACGAACAGAACCAGCTTCTTTGCTGCCTCGAGCCCGGACAGAATCAGTTTCTACTCCAGATGCCTGCAATAGTCATTTATTTACAACTGTTACTGTCACAACTCCTAGTTGCACAGTGTCTCAAAGTAAATCTGGGAAATTTTGTACAGCAGCATCATCTCCAGCATCTTCCACTGATTGTCAGAAAATTGGGGATGTGGGTGTAGATCAGAGGGTTATATTATCAGAAGAATCCATTAATAATGTTAAAGAAGCTCAGTTGCAGGCGGAAAATGCTGCTGATCTTGCTGCTGCTGCAGTTAAGCACTGCCAAGGTATATGGAGTCAGCTGGATAAGCAGAAGATTTCTGGATTGATACCGGAGGATGAAGCTAAACTTTCTTATGCAGCTGTTGCAATTGCTGCCGCTGCTTCTGTTGCAAAGGCAGCAGCAGCTGTTGCTAAGGTTTCATTAGATGCTGCATTGCAGGAACAACTGATGGCTGAAGAAGTACTAGCTTCGAGAGGAAATGAAAATTCTTGTCAAAGTAACACGGTTCCTCTTTCTGATGGCGTGAATATTCTGGGGAAGGCTACATCTGCATCCATCTTAAAGGGTGAAGGTGCAACCAGCTGTTCCAGTTCAGTTATTGTTGCTGCCAGGGAGGCTGTTAGGAGGAAGGTTGAAGCTGCTTCGGCTGCTTCAAAGCGAGCTGAAAATATGGATGCTATAGTAAAAGCTGCAGAGCTGGCAGCGGAAGCGGCAACTCAGGCTGGAAAAATTGTTGCTATGAAAGATCCCTTGACTTTGACTGAATTAGTAAAAGCCGGTCCAGAGGGTTACTGGAAAATACCCCAAGTATCTTCTGAGCTGGTCCTGACGTCAAATAACACGAATAAAGGGCAATCTAACTTAGATAGCGTTGAAGATGATCGCGATGGTTCTGCTCAATGTTTGAAGGGTGAAACATTAAATGAAAAAGGAACACAGACCACATATGGGAAGCCATCGACTCAAAGTGATCTGTCCATGGAATCCATGGAGGGCCACACAAAATTGGTTGATGGCATTTCGAGTTCTGTAACAAACAGGGAAAAGGTTTTCAGAGCACAAAGGGGGCATAAAGTTTCTGACATGGCGAAAACCGTTGGTGTTGTTCCAGAATCTGAGATTGGATCAAAATCTACTTCCATCACTGTTCAAAATGAGTATGAAAAAGCGGGGGAGATTTCAAAAGAGAGCAACATCAAGGAGGGTTCACTTGTAGAGGTTTTTAAAGATGGGGATGGTATTAAAGCGGCGTGGTTCTCAGCCAAGGTGTTGAGCTTGAAGAATGGAGGGGCGTATGTATGTTATCCTGAAATACCATCAGAAGGCCAGGTAAAGGAGTGGGTGGCACTGAAAGGCGAAGGAGATCAGGCACCCAGAATACGAATTGCCCATCCTATGACCAGCATACCATTTGAGGGAACAAGGAAGAGACGCAGAGAAGCTGTGGGGGGGATTATGCTTGGTCTGTTGGAGATAGAGTTGATGCCAAGATCCGAGATTGTTGGTGGGAAGGAGTCATCACTGGAAAGAATGACGAAATCACACTCACTGTTCATTTTCCAGCCCAAGGAGAAACATCTTCTGTTAAACCTTGGCAGCTTCGGCCTTCTCTCATATGGAAGGATGGGGAATGGATCGAATGGTCCGGTTCAAGAGAAACCAAACGCTCTTCTCATGAGGGTATCGACGTGCCACAAGAAAAGCGGCTCAAGTTAGGCAGTCCTCTGGTAGATCCAAAAGGGAAGGACAAGATACCTGAACCTAGTATCTCTGAAGAGTTAAAACTAACAGGCTTATCATCAAAAGATAAAATATTCAATGTTGGCAAGAACGCTAGAGATGAAAACCAGCCTGACGGAGGAGGTAGAACAATGCGAACTGGTTTGCAAAAAGAAGGATCAAAAGTGATATTTGGTATTCCCAAGCCTGGAAAGAAGAGGAAATTCATGGATGTTAGCAAACATTACGTAGCAGAGAGGAGCACTAAACCTAAGCCGCCAGAAGAAGAAAATGATTCGATCAAGGTTAGTAAATATTTAATGCCACAAGGATCCAGCTCGCGTGGATGGAAAAATAATTCCAAAGTTGATTTGCGGGAGAAACGAGTTGCTGATTCAAAACCGAGTCGGGTTCTTAAATCCGGGAAACCACAAACCATTTCGACTTCATCCAGATCTTTACCTCAGAAGCAGCCTAATTTTACAGAGATCAAGGATTCTGGTCGGCATGGTGAGACTACATCAGGAAAGTCGAATGCGACCCCGTTTCGATCTTTTTCTAGTACCGAGGGTGCACCAGCTGGTCCGATGGTGTTTACTTCTACAGGTCTTATATCAGATAACCCTCCTTCCTCATCTAAGAAAGAAACCATGTCAAATTCAAAACGCCTGAATAAAGGAAGACTTGCACCTCCAAGTGGAAGGTCGGCTAGAACTGAACAGGAGAAAGTACATAATGGCAATTCTGTAAAATCGACTCCGGAAGTTGTTGAACCTCGTAGATCTTGTCGGAAAATTCAACCGACATCACGGTTATTAGAAGGGCTTCAGAGTTCATTGATCATCTCAAAAATACCTGCTGTGTCTCATGACAAAAGTCATAAAAACCAAAGCAGGAGTGGTTCTTCTAGAGGGAATAATAATCATGGTTGAGAGGAAATGACCGGGGCAATTTAAGGTTAATGACAGACAGAGGCACAACATCTCCCGTGTTGTTTTTGTGTATACTTGTGATAGAGAAAACCAGGAACGAAAATTCTCGCTTTCCTAAGGGTTTTAGTTGTAAAATGTGTTGAGTAGATGTTTATATATTTAATTTAGTTTTTGGTCTCGTCACAATTTATGATGATTTTAGTATTTCGAATGGGTTGGATGTATCTGGTAACTCTCATCTGGTAACTCATTGTAATTTGAGCTTATAATTGCAGGAAAATTTTAATCCATCCTTATGTTTTAAAAAAAAAAAAAAAAAAAAAAAAAAAAAAAAAGT

>transcript_1 full_length_coverage=2;length=8610

GGGGGGGATCGGATTTGCCCTCCCTATAAATCCCTCCGTTTCGGTCTCTTCGCTTCGAAGACTCATTCTTTTTCCCATCAAACACCATCGAATTTCAAGAATTGTATTTTAGGGTTATGTGCAGAGATTGCTTTTCACTCCATTTCTGGGTTTCGGATGGATCATCTGTAAATTTCGGAGTTTCGGAGATCGATAGAATACGATTATAATGGCAGCTTCGCCTGTCAAATTTTTGTTCCTTTTCCTAGTCGCGTCCATCCTTTTATGGGTGCTTCTCTTGTTGGTGGATTTGATTCTGAGACGGGTTTTTGGACTTTCTGTTGTATTCCGAGTTGGTGGATTGAAGTGTATCAGGGATGTTGCGGTGAACATCAGAAAGGGTTCCATTGAATCTGTATCAATCGGTGAAATCAGACTCAGCTTACGTGGTTTTATTTCGAAAGATCCAAAGCTGCAGGTGTTAATATGTGATTTGGAAATTGTGATGAGGCCCTCAGCGCCAAAGAAAAGCAGAAAGGGCAGTAGATCTCAGAAACCCCGCACTCTAGGTAGAGGAACGTGGATGGTTTTATCTAACATTGCAAGGCTTTTCTCATTTTCTGTGACAGACTTGGTACTGAAGATGCCCAAAGCTACTATTGAAATTAAAGAATTGACAGGGGATATATCTAAAGATAATGGATCCAATCCATCCCTGTTCGTTAAGCTACACATATTACCTATTGTTGTTCACATGGGTGAACCACGGGTTAGTTATGACCAATCATCCAACTTTAACAGTGGAGACAATTCTGACACACAGACATTCTCCGCCATGGTGGAAAGGTCCTCTGCCCCTTTTAGCTGTGAGGAATTCTCTCTTTCCAGTGATTTTGGTCACGATAGTGAAGTAGGTCTAATTATTAAGAATGTGGATGTCACATGTGGAGAAGTCACTATGAACCTGAACGAGCAGCTGTTTCTAAAAAAAACGAGTTCATCAAACACTTCTCACACAGATGAAGTCATAGGTTCTGCTGTAGATTCTGTGCCTGCTAGGAGGCCAGACAAAAAACAAGTGCTTGCATCGCTTACAAAGTACACTTCTATATTTCCCGAAAAGGTCTGGTTCAGTTTACCCAAGTTAGGTGTGAAGTTTTTGCACCGAGAACATGATATTTCTGTAGAGAATAACATCATGGGGATTCAGTTAAAAGTAATAAAATCGTGTTCCAGTGAAGATGTGGGCGACAGTACACGCCTTGATGTTCAAATGGATTTCAGTGAGATTCATCTTCTTAGAGAAGCTGGCACTTCTGTCTTGGAGATACTGAAAGTTGATGTTGTATCTTTTGTGTATATCCCAGTACAGCCAAGCTCACCTATTAGAGCTGAAGTTGATGTTAAGCTTGGTGGTACCCAGTGCAATATTATAGCGAGTAGATTAAAGCCATGGGCACGCCTCCATTTATCAAAAAAGAAAAAAATGGTTCTTCGGGAGGAAACAACTACCGTTGAAAATCCACATTCAACTGAACTCAAAGCCATCCTGTGGACCTGCACCGTTTCAGCTCCTGAGATGACTATTGTGCTTCACAGTATTAGTGGTTTGCCACTGTATCATGTTTGCTCACAATCTTCACATGTGTTTGCAAATAATATATCAAGTACTGGAACTGCGGTACACACAGAACTTGGTGAGCTAAATTTGCACATGGCAGATGAGTATCAAGAATGCTTGAAAGAAAGCCTGTTTGGCGTGGAAACAAATTCTGGTTCGTTAATTCATATAGCAAAGGTTAGTCTAGACTGGGGCAAAAAGGATATGGACTCATTTGAAGAAAATGGTCCTAGATGTAAATTGGTTCTCTCTGTTGATGTGACTGGCATGGGTGTTTACTTTACCTTTAAGCGTGTTGAATCACTTATATCAGCTGCTATGTCCTTTCAAGCTCTCTTTAAAAATCTGTCGGCTTCCGGAAAAAGAACCACACCGAATCAAGGAGGACGTATTTCCAAACCATCAGGAAAAGGGACCCGCCTTTTGAAATTTAATCTCGAGCGATGTTCTGTGAATTTCTCTGGTGATGCAGGCGTGGAAAACACGATTGTTGCGGACCCCAAACGTGTGAATTATGGTTCACAGGGTGGTCGAATTGTGATTAGTGAATCAGCTGATGGTACTCCGCGCACTGCAGATGTAATGTCTACAATATCTGATGAGTACAGGAAGATAAAGTATTCTATATCTCTTGATATCTTTCATTTCAGTTTATGCATGAACAAGGAGAAACAGTCCACCCAGATGGAACTTGAAAGAGCTAGATCCATCTATCAGGAACATGAAGAGGAGCATATGCTTGGAACAAAGGTGACATTGTTTGACATGCAGAATGCTAAATTTGTGCGTCGTTCAGGTGGTCATAAAGAGATTTCCGTCTGCTCGCTTTTTAGTGCTACTGATATTACAGTCAGGTGGGAGCCTGATGTGCATCTGTCATTAATTGAACTTGTTCTGCGATTAAAGTTACTGGTGCACAATCAGAAGCTTCATAACAAATGTATGGACGATGTCTCTAATATGGGAACTGTTGAAAAGAAGAAAGAACCCAGTACAGAATCCGGACAATTTGATAGACAACACAAGAAAAAGGAATCTGTTTTTGCCGTTGATGTGGAGATGCTGAATATATCTGCTGAGGTTGGAGATGGGGTTGATGTATCGGTTCAAGTTCAGTCAATTTTCTCTGAAAATGCTCGTATAGGAGTACTTCTTGAAGGACTTATGCTCAGTTTCAATGGATCTAGACTTCTGAAAAGTAGTCGGATGCAAATATCCCGCATTCCTAATGCCTCTATTAGTTCATCTGATTCAAAACTACCAGTAGCTACAGTGTGGGACTGGGTGATTCAAGGCCTTGATATTCATATTTGCATGCCTCACAGGTTAGAGTTGCGTGCCATTGAGGATTCTGTTGAGGATATGTTGCGAGCACTGAAGCTTATTACTGCTGCTAAAACCAATCTTATATTTCCTGTGAAGAAAGAAAGCTCAAAACCGAAAAAGCCTAGTTCAATGAATTTTGGATGTGTAAAATTTTGTATCCGTAAACTAGCTGCTGATATTGAGGAAGAACCTATCCAGGGATGGCTCGATGAACATTATCAGCTGCTGAAAAGCGAGGCCCGTGAATTAGCTGTCAGATTAAGTTTTATTGATGACGTTCTTTCAAAACTCAACCATTGTACTGGATCAGCTGAAATAAATCATTCTAGCCATGAAGGAAAGGTCCACTATAATGGCGTTGAAATCGATGTTCAAGATTCTTCTGCTGTTGATAAATTGAAGGAAGACATTTATAAACAGTCGTTTCGGTCGTATTACCAGGCATGTCAGAACCTTAAATCATCAGAAGGTTCAGGTGCTTGTAGGGAAGGTTTTCAGGCTGGTTTTAAGCCTAGCACTGCTAGGACATCTCTTCTTTCCATTTCTGCGACAGAGTTTGATGTAAGCTTAACAAAAATTGAAGGTGGTGATGATGGGATGATAGAAGTTGTAAAGAAACTTGATCCTGTTAGTCTAGAAAATAATATACCATTTTCTCGACTGTATGGGTGTAATATTCTCTTGCGCACAGGCTCTCTTTCTGCTCAGTTGAGAGATTACTCATTTCCTCTTTTTTCTGCATCTTCTGGTAAATGTGAAGGTCGTCTTGTACTAGCACAGCAGGCAACATTTTTTCAACCTCAAATCCGCCAAGAAGTCTACATTGGTAAATGGAGGAAGGTGCGTGTGCTGCGGTCAGCTACTGGCACAACTCCACCAATGAAAACTTACTCAGATCTGCCTATATATTTTCATAAAGCAGAAGTTGCCTTTGGGGTGGGGTTTGAACCAGCTTTTGCAGATGTAAGCTATGCATTTACCGTTGCTCTTCGCAGGGCTAATCTAAGTGTCAGAAATAAAAATGTACAACCTCTGCCGCCTAAAAAAGAAAAGAGTTTACCATGGTGGGATGATATGAGAAACTACATTCATGGAAACATTTCCTTACACTTCTCAGAAACCAGATGGAATGTTCTTGCAACTACTGATCCTTATGAAAAGCTTAACAAAGCTCAAATTCTTTCTGGTCCTATGGAGATCCAGCAGTCAGATGGCCGTGTTTATGTTTCTGCAAAGGATTTCAAGATTTTCTTGAGCAGTTTGGAGAGTTTGGTAAACAGTTGTAGCTTAAAACTTCCTACTGGTTCGCCAGGTGCTTTCGTAGACGTCCCAGTTTTTACCGTGGAAGTTACGATGGAGTGGGGATGTGACTCCGGGACTCCCCTGAATCATTTTTTGCACACCCTTCCCATTGAAGGGAAAACTCGTGAAAAAGTTTTGGATCCCTTCAGATCAACTTCTCTCTCCCTTTGCTTTAACTTCTCACTTAGACCCCCTGTTTCCTCAAGTGAGAAGCACACACCATCTACTACGGGAAATAGCAGTGTTGTGGATGGAACTGTCTATGGTCCTCCATGTAAGTCTGATAATGTTTCAGTTGTTTCTCCTACAGTGAAAGTTGGTGCTCATGATTTAGCATGGTTAAATAAATTTTGGAACTTAAATTACTTTCCTCCTCATAAATTACGTACTTTCTCTCGGTGGCCTCGTTTTGGAATTCCAAGAATTCCCAGATCAGGCAATTTGGCATTGGACAAGGTGATGACAGAATTTATGCTCCGTGTCGATGCGACTCCGACTTGTATAAAACATATGCCTTTAGATGATGATGATCCAGCCAAAGGATTGACATTTACCATGACAAAAATGAAATTTGAATTCTGCCAGAGTCGAGGTAAGCAAAAATATACTTTTGATTGCAAGCGTGAACCTCTCGATCTTGTTTACCAGGGTCTTGACCTTCATGTGCCCAAGGCTTTTTTAAACAGAGAAGATTCTATTAGTGGCACAACATCTGTTCAAAAGACTGGACATAGTTCAGAATCTCCCTCCATCGATAAAGTTCCCAAGGAAAAATGCAACTACATGAGTGGCTGCACCGAGAGGCATCGCGATGATGGATTTCTATTATCATCAGATTATTTTACAATTAGAAAGCAGGCTCCAAAAGCTGACCCTGAAAGGTTATTAGCATGGCAAGAAGCAGGAAGGAGAAATCTTGAGATGACATATGTGAGATCTGAGTTTGAAAATGGGAGTGAGAGTGATGAGCATACACGATCTGATCCAAGTGATGACGATGGATACAATGTGGTAATAGCTGACAATTGTCAGCGTGTTTTTGTTTATGGCCTCAAGCTTTTGTGGACTATCGAAAATAGAGATGCCGTTTGGTCATTTGTTGGTGGATTATCGAAAGCATTTGAACCTCCAAAGCCTTCTCCTTCTCGGCAGTATGCACAGAGGAAATTGGTGGAGGAGAATCAGAATGACGACGGGGCTGAAAAGCTCCAAGATGATATATCTAAACCACCTTCTACTAGCCATGAGGGAAACTCTCCATCTTCAGCAAATGAGGAGACTTTAGGTTCATTTCCTTCTCCATCCCATTCAGTTGAAGTGGAGAACTCATCATCTGTTGTGGATGCCAAAAATGGAAACAAAAATGACAAAGAGGAAGAGGGGACTCGCCATTTCATGGTGAATGTTATTGAGCCTCAGTTCAATCTTCACTCCGAAGATGCCAATGGTAGATTTCTGCTTGCTGCTGTCAGTGGTCGTGTTTTAGCCCGTTCATTTCATTCAGTCCTTCATGTTGGCTATGAGATGATAGAGAAAGCGCTTGGTACCGCAAATGTACAGATTCCAGAATGTGAACCCGAAATGACATGGAAACGCATGGAGTTTTCTGTGATGTTGGAGCATGTACAAGCTCATGTTGCACCAACGGATGTTGATCCAGGGGCTGGACTGCAGTGGCTCCCAAAAATTCTTAGGAGCTCTCCAAAAGTAAAGCGTACTGGTGCTTTACTAGAGAGAGTATTTATGCCTTGTGATATGTACTTTCGTTACACGAGGCACAAAGGCGGAACTTCAGATTTAAAGGTGAAGCCCTTAAAAGAGCTCACTTTCAATTCCCATAATATAACGGCAACAATGACTTCTCGCCAGTTTCAGGTTATGCTAGATGTGTTGACTAATCTTCTCTTTGCCCGGCTTCCCAAGCCTCGAAAAAGTAGTTTGTCATATCCTGCGGATGACGATGAAGATGTTGGAGAGGAGGCAGATGAGGTGGTTCCTGATGGTGTTGAAGAGGTAGAACTGTCTAGAGTCAAACTCGAACAGAAAGAGCGTGCGGTGAACTTGCTTCTTGATGACATCAGGAAATTGTCTCTTGGTAATGATACATCCATTGATCTAGAAAAGGAAGACAATTTATGGATGATAACTGGTGGAAGATCGACATTGGTGCAAGGACTAAAGAAACAGCTTTTAATTACTCAAAAAGCTAGAAAGGAAGCATCGGCATCATTAAGGTTGGCTTTGCAGAAAGCTGCACAGCTACGGCTGATGGAGAAGGAGAAGAACAAAAGTCCATCTTATGCTATGCGCATTTCTTTGCAAATAAACAAAGTGGTTTGGGGCATGCTTGTGGATGGTAAATCTTTTGCTGAAGCTGAGATAAATGATATGATATATGATTTTGACCGGGATTACAAAGATGTTGGTGTTGCTCAGTTTACGACTAAGTATTTTGTAGTGAGAAACTGCCTGCCTAATGCCAAATCTGATATGCTTTTATCAGCATGGAATCCGCCTCCTGAATGGGGAAAAAAAGTAATGTTGCGTGTAGATGCAAAGCAGGGAGCTCCAAAGGAGGGAAGCTCTCCTCTAGAGCTTTTTCAGGTAGAGATCTACCCCCTAAAGATACATTTAACAGAGACAATGTACAGAATGATGTGGGGATATTTCTTCCCAGAAGAAGAACAAGATTCACAGCGGCGGCAGGAAGTTTGGAAGGTTTCAACCACTGCTGGCTCTAAACGTGTGAAGAAAGGCTCGTTGGCCCAAGAAGCTCCCACATCAATTGGTCAATCAACAAAAGAATCCGAGGTTTTATCCAAATCGGGTGCTCCCGGACTATCATTTACTTCTGATTCTGCTCTAGTGCCAAAGTTACCAAACACGAAAGGCACCATGGTTTGTGGTTCAACCCCAGAGCTAAGACGAACATCTTCTTTCGACCGATCTTGGGAAGAAACTGTTGCAGAGTCTGTGGCTACTGAACTCGTCTTACAGGCTCACTCTTCAAGCATTTCTTCCTCAAAAAGTGGCCCCTTGTATTCAATTGAGCAACAGGATGAACCTTCTAAACAGAAATCAAAAGACCCCAAAGCCATCAAGTCTGGCCGGCCATCTCATGAAGAGAAAAAAGTAGGGAAGTCGCAGGAAGAGAAGAAAACTAGTAGGCCTCGGAAGATGATGGAGTTTCACAATATTAAGATAAGTCAGGTTGAGCTACTAGTTACTTATGAAGGATCAAGATTTGTTGTGAACGATCTTAAGTTGCTGATGGATACATTTACCCGTGTTGAATTCACGGGGACTTGGACGAGACTGTTCTCACGAGTTAAGAAACATATCATTTGGGGAGTCTTGAAGTCCGTGACTGGAATGCAGGGTAAAAAGTTTAAAGATAAAGCACATAGTAAAAGAGACCCAAGTAAAACTGTAGTTCCCGATACCGATCTCAACTTCAGTGACAACGAAGGACAGCTGACAAAATCTGATGAATACCCAATAGCCTTCCTCAAGCGGCCTACTGATGGAGCGGGTGATGGGTTTGTGACTTCCGTAAGGGGCCTATTCAATACTCAGCGCCGAAAGGCCAAGGCTTTTGTTCTTCGGACTATGAGAGGTGAAGCAGAGAATGATTTCAGCGGCGATTGGAGTGAAAGTGATGTTGAGTTCTCTCCCTTTGCCCGACAACTCACCATAACTAAAGCTAAGAGACTGATTAGACGTCATACCAAGAAATTCCGCTCAAGAGGACAAAAGGGTTCGTCTTCGCAACAGACACTTCCGGAATCTCCAAGGGAGCTCACACCATATGGGAGTGATTCTTCAAGTGGATCTGATCCATTTGAGGAATTTAACGAGATAGGGATCACTTCAGGAATGTCTAAGGGGGACGACGATCAAGTCATTTGAAAGAAAGAAGGTGATCATCTCAATGAGTTAAGGGAGCAGGCTCTAATTTTATCTAGGGTTGTTCAATGAAGGGTTCTGGGCCCAGGTGAGTCGCTGTGTCGAGGCAAGATATAATTTACTGGGTTATGGTGGCTGGCTTGGGTGAGACTTGGAAGATTTTAAATTTTTTCGAGTTTCTTCAAATAAAAGCATGAAAATGAAGTTCTTAGTGGCGATCTACGGAGGAGGATTTGGGTGGAACCCTACACGTTTCCTCTGTAAGCATTGCGTTTATTATTTTTGGCTAAAGGAAGGAGCTACGTTCTACTTCAAATGCATTGTTTGTTTTTGACTTTCTGGGTAGAAGGAATATACGGGCTCCCTCAAAATTTTATAGTTTTGTACATAGGAACGTTCAAATGCCTTTTGGGCTACACAATAAAAGCAAGAAATTCTCTCTCTCAAAAAAAAAAAAAAAAAAAAAAAAAAAAAGT

>transcript_2 full_length_coverage=2;length=8449

GGGACAGGGTGTAAACACCTCTTTACTATATAGCACCATCCGCCCCTAGCTGTATCGCTTCTTCTCCCTTTTTTATTTCATTCTACTTCGACCCAGTCCCTTCTCCATTACACTTCAATTCGATCCCAAAATCAACCTTTGGCACCGACATTCGAGGTCGATTACATAACTCGTAATAGTACCCTTCGTTTCAACCCCAATTTTAAAGAATATTCAATCTAACCAGATTAGGTTTCGTATACATAACACATATATACATATAATTCTGTATCGGATTGTTTTGTTGTTATTTTATTTATCTTGTGTCTCAGATGATGAAAGCTTGAGGTTTTACAATCTGGGTTTGATTCGGAATTGATCCTTGGTGGTGAAAGTTTGAGAATTCATTGTGGAGAAGGAATACGCAAACTACCGTAAGAGCTGGGGGCGAATCAGTCTCGACAAAGCCCGGCAGCGGCTCAAGCCGGCTTAGGATTCTGGTTTTTTCGCCCTAATGCTCCTCGAACCCACACACTCGCATACCTCCCTCAAATGGACTTCGTGAACCGTCACTCAGCACCGGATCACCGCCCTTCACCGTCTTCCGCTGCCGACACTTCTTCATCTTCTTCATCGTCTACAAATTCTGCGCCTCACCCCCAACTTCCTCATGCTCCCGAAGAACCTGAGTATTTGGCGCGGTACATGGTTGTTAAGCACTCATGGCGCGGCCGGTACAAGAGAATTTTGTGCATATCGAATTTCTCTATTACTACTTTGGATCCATCTACACTAGCAGTGACCAATTCGTATGATACGGCCACTGATTTCGAAGGGGCGGCTCCAATTCTAGGTCGGGATGAGAATTCTAATGAGTTCAATGTCAGTGTGAGAACGGATGGGCGTGGAAAGTTCAAGGGGATTAAGCTTTCATCGAGATACAGGGCAAGTATATTGACTGAGTTGCATAGGATCAGAGGGACTAGGATTGCGGCTGTTGCCGAGTTTCCAGTACTTCATCTGCGGCGACGGAATATGGAGTGGGTTCCTTTTAAATTGAAAATTACTTCTGTTGGAGTTGAACTAATTGAATCAAAATCTGGAGACCTACGCTGGTGCTTGGATTTTAGAGACATGGATTCTCCTGCTATCATTCTTCTCTCTGATGCTTATGGGAAGAAAAATATGGATCATGGAGGTTTTGTTCTCTGCCCTTTATATGGAAGAAAATCTAAAGCTTTCCAAGCTGCTACTGGGGCTTCAAACACAGCTGTTTTATCAAATTTGACTAAAACTGCGAAAACTATGGTGGGGTTGTCACTATCAGTGGACAGTTCTCAATCACTCAGTGCGGCAGAGTTCATAAAGCGAAGGGCCAAAGAGGCAGTTGGTGCTGATGAAACCCCTTGTGGGGGTTGGTCTGTGACAAGGTTGCGGTCTGCTGCTCATGGAACTCTAAATGCACCAGCTTTGAGTTTGGGAGTTGGGCCAAGAGGAGGACTTGGGGAACATGGCGATGCTGTCTCCCGTCAGCTTATTCTTACAAAGATTTCACTTGTAGAAAGGCGCCCAGATAACTATGAAGCTGTTATCGTTCGCCCTTTATCTACAGTAAGCTGTCTTGTTCGATTTGCTGAGGAGCCCCAGATGTTTGCAATTGAATTTAATGACGGATGCCCTATTCATGTTTATGCAAGCACTTCTCGTGATAGCTTACTTGCAGCAGTTCGAGATGTATTGCAAACAGAAGGTCAGTGCCCAGTACCTGTTCTGCCAAGGCTAACAATGCCTGGTCACCGTATTGATCCACCTTGTGGGAGAGTTCATTTACAAATTCAAAAATTACCACTTGGACAGCAACGCTCTGTTGCTGATATGGAAAGTACTTCCATGCATTTGAAACACTTGGCAGCAGCTGCCAAAGATGCTGTTGCTGAGGGGGGCTCGATTCCTGGCTCAAGAGCTAAACTATGGCGCAGAATAAGAGAGATTAATGCATGTGTTCCATATAGTGGAGTGCCCCCCAACATTGAAGTGCCTGAGGTGACTTTAATGGCCTTGATTACAATGCTTCCAGCTACTCCAAATCTTCCTCCAGAGTCTCCTCCTTTGCCACCTCCTTCACCTAAAGCAGCTGCAACAGTAATGGGCTTCATTGCATGTCTGCGCAGATTATTGGCTTCAAGAAGTGCAGCTTCACATGTTTTGTCTTTTCCTGCTGCTGTTGGAAGGATAATGGGGTTACTTAGAAATGGTTCAGAGGGTGTAGCAGCTGAAGCCGCGGGGCTTGTTGCGGTACTTGTTGGTGGTGGTCCTGGGGATGCAAATATACTAACGGATTCTAAAGGAGAGCGGCATGCCGTAATTATGCATGCCAAGTCTGTATTGTTTGCTCATCATAGTTATGCTATTATCCTCGTCAATAGATTGAAGCCTATGTCTGTATCACCCTTATTGTCAATGTCTGTTGTGGAAGTTCTTGAGGCTATGATATGTGAACCACATGGTGAAACCACCCAATATCCTGTTTTTGTTGAATTATTACGTCAAGTAGCCGGCTTGCGGCGTCGCTTGTTTGCATTGTTTGGACATCCTGCTGAGAGTGTTAGAGAAACAGTTGCTGTGATTATGCGTACAATTGCTGAAGAGGATGCGATTGCAGCAGAGTCCATGCGTGATGCTGCTTTGCGTGATGGTGCTTTGTTGAGGCATTTGTTGCATGCATTTTTCCTTCCTGCTGGTGAGCGGCGTGAAGTTAGTCGACAACTTGTGGCTCTTTGGGCAGACTCTTATCAACCAGCTCTTGATTTGTTGTCTAGAGTTTTGCCTCCTGGGCTTGTTGCTTATTTGCACACACGTCCTGACGGAGATGCATCTGAAGACCCCCAAGAAGGGTCATTGATCAGTAAAAGACAAAGACGTTTACTTCAGCAGAGAAAAGGTCATACAGGCAGAGGAATAATAACTCAGGAGCATTCCTTATCTTCTGCCAATAATTTTGAAGTTAGTGATTCAGCGAGACAGTCAAATGCTGTTCCTTCTAAAGGGTTAGATAACCACCAAAGACCTGCTCTAGATCCAAGTTTTGGACAGGTTCCAGCCATTCCCTCATCTGTTGTTCACACGGGTGAAAACTTTACCAGTGAATCGTCTCCTACAGGGGTTCCACCAATGGATAATTCAGTTATTTCTTCTGATGCTCCATCATTAAATATAAATGAGGTATTGGAGTCAAATGGTTCAATTTCTGTTGATCCTGATGCCAATGTAGTTGGTTCCCAGAACACTGGCGTTCCTGCTCCTGCTCAGGTTGTTATGGAGAGTGCACCTGTGGGATCTGGCAGGCTGCTATGTAACTGGCCTGAATTTTGGCGAGCATTTAATCTTGATCACAATCGTGCAGACTTGATTTGGAATGAGCGTACCAGGCAAGAGTTAAGGGAGGCTTTGCAAGCTGAGGTTCACAAATTGGACGTTGAGAAGGAACGTACTGAAGATATAGCTCCGGGAGGTGCAACAGTAGAGATAATGACTGGGCAAGATAGTGTGCCTCAGATATCATGGAATTATACGGAGTTTTCTGTTAGTTATTCTAGCCTATCAAAAGAAGTTTGTGTAGGTCAATATTATCTGCGTTTGCTTCTTGAGAGTGGAAGCAGTGGCAGAGCACAGGATTTCCCATTGCGTGACCCGGTTGCTTTCTTTAGAGCACTATATCATCGATTCTTATGTGATGCTGATATAGGACTTACTGTAGATGGTGCGGTGCCTGATGAATTAGGCGCATCTGATGATTGGTGTGATATGGGAAGACTAGATGGTTTTGGAGGAGGAGGGGGTTCTTCTGTTAGAGAGCTTTGTGCAAGAGCAATGGCAATTGTATATGATCAACATTATAAAACTATAGGCCCTTTTGAGGGTACCGCTCACATTACAGTTCTTTTGGACAGGACTGATGATAGAGCTTTGAGGCACCGTATTCTTCTTCTTTTAAAGGTTCTAATGAAGGTTTTATCAAATATCGAGGCTTGTGTGTTGGTTGGAGGGTGTGTGTTAGCTGTTGATATGTTGACAGTAATCCATGAAGCTTCAGAAAGAACTGCTATTCCCCTGCAATCAAATTTGATAGCTGCTACGGCTTTCATGGAACCACTCAAGGAATGGATGTATATTGACAAAGATGGTGCTCAAGTTGGGCCTATGGAGAAGGATGCTATCAGAAGGTTCTGGTCAAAGAAAGATATTGATTGGACAACAAGGTGTTGGGCTTCTGGAATGCTGGACTGGAAGAGGTTACGTGATATTCGTGAGCTTCGTTGGGCCCTGGCTGTTCGGGTTCCCGTGCTTACTCCAATTCAGGTAGGGGAGGCTGCATTGTCCATATTACACAGCATGGTATCTGCACATTCAGATTTAGACGATGCTGGAGAAATAGTTACCCCGACACCCAGAGTAAAACGGATCTTGTCAAGTCCTCGTTGTCTTCCACATATTGCACAGGCCATGCTTTCTGGGGAACCAGGTATTGTAGAGGCTGCTGCTGCTTTGATGAAGGCCATTGTTACTAGAAATCCCAAGGCAATGATACGGCTATACAGCACTGGAGCATTTTATTTTGCCCTAGCATACCCTGGATCCAATCTTCTTTCGATTGCCCAACTCTTTTCAGTGACTCATGTACATCAAGCATTTCACGGTGGCGAAGAAGCCGCACTTTCATCTTCACTCCCATTGGCAAAACGAAGTGTATTGGGTGGGCTTATCCCTGAATCATTGCTTTATGTATTGGAGCGTAGCGGTCCAGCTGCATTTGCAGCTGCAATGGTTTCTGATTCTGATACTCCAGAAATTATATGGACACACAAAATGCGAGCAGAAAATCTGATCCGCCAGGTTTTGCAGCATCTTGGTGATTTTCCCCAGAAATTATCACAGCATTGCCATTCATTATACGATTATGCGCCAATGCCGCCAGTGACATATCCAGAGCTAAGAGATGAAATGTGGTGTCATCGTTATTACCTTCGAAACTTATGTGATGAGATCCGGTTTCCAAACTGGCCAATTGTTGAACATGTTGAGTTTCTACAGTCATTGCTGGTAATGTGGCGTGAAGAGTTGACACGAAGACCTATGGATCTTTCTGAAGAAGAAGCTTGCAAAATACTAGAGATATCATTGGAAGATGTATCAAGTGATGACACTAATAATAAAAATTCTTTTGAGATGTCTGAGGAGATTGCTAGCATATCTAAGCAGATTGAGAACATTGATGAAGAAAAGCTCAAGCGGCAATATAGGAAACTTGCAATGAAATACCATCCTGACAAAAATCCTGAAGGGAGGGAGAAGTTTCTTGCTGTACAGAAAGCTTATGAGCGACTACAGGCCACCATGCAAGGCTTGCAAGGACCCCAACTTTGGAGGTTGTTGCTTTTGTTGAAGGGACAGTGTATCTTATACAGGCGATATGGGGGAGTATTGGAGCCATTTAAATATGCAGGTTATCCCATGTTGCTCAATGCAGTCACTGTGGACAAGGAAGACCACAATTTTCTTTCCTCTGATAGAGCACCTCTTCTTGTTGCAGCTTCAGAGCTCATTTGGCTGACGTGTGCATCTTCTTCACTGAATGGTGAAGAGCTTGTGAGGGATGGCGGGATACACCTTCTCTCGACTCTTCTTTCCCGTTGCATGTGTGTGGTCCAGCCAACTACTCCAGCGAGTGAACCATCAGCCATCATTGTTACAAATGTGATGAGAACTTTTGCTGCTTTGAGTCAGTTTGAGAATGCCAGAATTGAGGTGCTTGAATGTTCTGGACTAGTTGATGACATTGTGCATTGCACTGAACTTGAGCTTGTACCTGCGGCTGTTGATGCTTCCCTCCAGACTATAGCACATGTTTCCGTGTCCTCTGAATTACAAGATACCTTATTAAGATCTGGAGTTCTATGGTACCTTTTACCACTGTTGCTTCAGTATGACTCAACTGCAGAGGAGTCTGACACGACGGAGGCACACGGTGTTGGTGCTAGTGTTCAAATTGCCAAAAACTTACATTCCATTCGAGCATCCCAAGCTCTGTCAATGCTTAGTGGTTTGAGTTCTGGCGAAATTTCAACACCCTATAATCAGGCCGCAGCTGATGCTCTCAGAGCTTTGCTGACACCTAAACTCGCAAGTATGTTAAAAGATAAATTACCGAAAGACCTCCTGTCCAGATTAAACACAAACTTGGAGTCACCCGAGATTATTTGGAATTCTTCAACCCGAGCAGAACTATTGAAATTTGTGGACCAGCAGCGCGCAAGCCAGGGTCCTGATGGTTCATATGATCTGAAAGATTCACACGTCTTTGTGTATAAGGCCCTTTCAAAAGAGCTGTATGTTGGAAATGTGTACTTGAGGGTTTATAATGATCAACCAGACTTTGAGATCAGTGAGCCGAAAACTTTCTGTGTTGCCCTTGTTGACTTTATATCATGTCTAGTGCACAATCAAAGTGTTACAGATACTGATATTCAAAACGAAGTTAATCCCAGTGGCTCATCCATCAAAGCATCTGAGCCTGAAACTGGATCAACTAATGAACAAAGTGTTTCTGACAATTCTTCGGCGGTAACTGATGAAAAAGAGATGGGTAAAGATGATTTTGATCTGGTTAAGAAGCTTAAGTTTGGGTTGACCTCTCTTAAGAATTTACTGACAAGCAATCCAGATTTGGCGTCTATCTTTTCAAGTAAAGAACAGCTTTTACCTCTTTTCGAATGTTTTTCTGTTCCTGGTGTATCAGAATGTAACATCCCTCAACTTTGCTTGAGTGTGCTGTCACAATTGACTGCATATGCTCCATGCTTGGAGGCAATGGTCGCTGATGGATCTGGTCTTCTCCTTTTATTACAAATGCTTCACTCAGCCCCTAGTTGTCGTGAAGGTGTTCTTCATGTTCTTTATGCCTTGGCAAGTACACCAGAACTTGCATGGGCAGCTGCCAAACATGGCGGAGTGGTGTACATACTTGAACTTCTATTACCCTTGCAAGGAGAAGTTCCTCTACAGCAAAGAGCAGCAGCAGCTTCTTTATTGGGAAAGCTTGTTGGGCAGCCAATGCATGGACCTAGAGTGGCAATAACATTAGCAAGGTTTCTTCCAGATGGGTTAGTATCAGTTATTAGGGACGGTCCTGGTGAAGCTGTTATCTCTGCCCTTGAGCAGACTACGGAGACACCAGAACTTGTATGGACACCAGCAATGGCAACTTCTTTGTCTGCACAAATTGCAACCATGGCATCAGATCTGTATCGCGAACAAATGAAAGGTCGTGTTGTTGATTGGGATGTACCTGAGCAAGCATCTGGCCAGCAGGAAATGAGAGACGAGCCGCAGGTTGGAGGAATCTATGTTAGGCTGTTCTTGAAAGATCCCAAGTTTCCTCTACGAAATCCAAAGAGATTCTTGGAAGGACTATTAGACCAGTACCTATCATCCATTGCTGCGACACACTATGACATGCAAGCAGTTGACCCTGAGCTCCCTCTACTATTATCTGCTGCTTTGGTTTCATTATTGCGTGTACACCCTGCACTTGCGGATCATGTTGGATATCTCGGATATGTCCCCAAACTTGTGGCTGCAGTAGCTTACGAAGGAAGACGAGAAACAATGGCATCAGGGGAGGTGAAGAATGGTAATGCAGATGAAACGGAGGATGGTTCAAGCCAACCTTGTCCACAAACTCCACAAGAACGCGTGCGTCTCAGTTGTTTACGCGTCTTACATCAACTTGCAGCTAGTACTACTTGTGCTGAAGCTATGGCAGCAACTAGTGTAGGAACTCCTCAGGTTGTGCCACTTCTAATGAAAGCTATTGGGTGGCAAGGTGGAAGCATACTGGCCCTGGAGACACTTAAACGTGTTGTGGTTGCCGGAAATCGAGCTAGAGATGCCCTTGTTGCACAAGGACTTAAGGTTGGTCTAGTCGAAGTTCTACTTGGTCTCCTTGATTGGCGAGCTGGAGGAAGAAATGGTCTTTGCTCTCAGATGAAGTGGAATGAATCCGAAGCATCCATTGGCCGCGTGCTTGCCGTTGAGGTTCTACATGCCTTTGCCACAGAAGGTGCTCATTGTACTAAAGTGCGTGACATATTGAATGCTTCTGATGTTTGGAGTGCATATAAAGACCAAAAGCATGATCTTTTCCTGCCATCAAACGCTCAATCAGCTGCTGCAGGGGTTGCCGGTCTTATAGAAAGCTCGTCATCTAGACTGACTTATGCCCTTACAGCTCCCCCTCCACAATCTAATTCGTCAAGACCGCCAGACACCAATGGCAAGCAGGATAGTTTATATTAACATCCATGAACTCACCAAAATATACTCTACTCATTTGTATAGAACACTGTACAGTCTCTTTTTTGTTCATTTTAAATTTTCTCAAAAAAAAAAAAAAAAAAAAAAAAAAAAAAGT

>transcript_3 full_length_coverage=2;length=8018

GGGGTCATAACTCTGCTCTCTTCTCTCTCTCTCTCCTCCGGTTAGTGTAGGAATTCTCTGACGCTCTCGAATAAACATCCCTACACCGCAACCACTTCTATCATCTAACGCACGCCCACAGAGGGTTGGAGCAAATTCTAGGGTTTTTGATATTAGATCATTGCATACTGTAAGATCTGTGAGGGGATTTTGTAAAGATCTGTTTTTTTTTGGCGAAGAAGATACTTAGGGTTAGGGTTTTGATGCTTTGGCGGAGATTCAATCCGCCAAACGGGAAAGGGCTTTGGTTCGGACCGTGCAATGGCCAATCCCGGAGTCGGGGCTAAATTTGTGTCTGTGAATTTGAACAAATCTTATGGGCAACAATCTCATCCCAGCTCTTACGGATCGAGTCGAGTTCGACCTGGTAGTCATGTTGGGGGCAGCGGGGGAATGGTGGTTTTGTCAAGACCTCGGAGTTCGCAGAAGGTTGGGTCGAAGCTTTCTGTTCCACCCCCCTTGAATCTGCCTTCGTTGAGGAAGGAGCATGAACGCTTTGACTCGTCAGGATCGGGTAGTGGAGCGGCTGGTGGGGGGGTTGCAGGCAGTGGGCCAAGGCCGAATTCATCTGGTATGAGCTGGACGAAACCAGGGAATATTGCTTTGCAAGAGAAAGACGAGAGTGGTGAAACTCAGATGGATGCTATGGACCAGGGTTTGCAAAGTGTTAATTCGATTAACCGAGGTGGAAGTGTTTATACCCCACCTTCCGCTCGGTTGAATGCTGGTGGGCCTACTGTGTCTGCTTCAGCTCAAGCTTATCGACCTGTAGAGAAATCCACAGTCTTGAGGGGTGAGGATTTTCCTTCTTTGAAGGCTGCCTTGCCCACCACTTCTGGACCTTCACAGAAACAAAAGGATAGTTCGAATCAGAAACAGAAGCATGGAGTGGGTGAGGCCTCTAACGAGGAGAGGGATAATTCCCATTTCAGCTCACAAGTTCACATGCGCCCCCAGGTGCAATCCTCTCATAATATTGTCCGCAATGGGTTCAATGAGAATGGTGATGATGGCCATGTGTTGGGTGGTTCTGGCATGTCAGAAAAACAACCTAGGAAGCAGGAAGATTATTTTTCGGGTCCACTGCCGCTAGTTAGATTGAATCCAAGATCTGATTGGGCTGATGATGAGCGTGATACTGGTCATGGTTTCATAGACCGGGGAAGAGATCATGGGTTTTCAAAAAGCGAAGCATATTGGGATAGAGATTTCGATATGCCCAGAGTTAGTGTTTTGCCACACAAACCAGTACAAAAGTTTATTGATAGGCGGGGTCAGCGTGACGATGAAACTGGAAAAGTGTCTTCTAGTGAAGTCCTTAAAGTGGACTCATATGACAGAGATGTAAGAACACCCAGTAGGGAGGGTCGTCAAGGGAACACGTGGAAAAGTTTGGATCCTTATAGCAGAGATGTAAGAACACCCAGTAGAGAAGGTCAGGAAGCGAGCTCGTGGAGAAGTTCCCTTGCTCCAAAGGATGGCTTTAATTCCCAGGTGGTTGTTAATGATAACAACAATATTGGGGCAAGGCCATATAGTCTGAATCGAGAAGCAATCAAAGATAACAAGTTTGTCCCGCCAGCTTTTCGAGACAATGCTCAGGATGATTTCAATGGTGGAGTTGCTGGAAATCGAGATTCTTCTTTTGGAAGGAGGGATATGGGGTATGGACAAGGAGGGAGGCAGTACTGGAACAACACGGTAGAATCGTTTAGTAACCGAGGGGCTAATACTCACGATCGGTATGGCAGTGAACAATACAATAGACACAGAACTGGTGCTTTTCAGAATAACTCAGTGCCCAAATCTTCATTTTCTTCGGGTGGCAAAGGGCTTCCTATGAATGATCCAATACTAACTCTTGGAAGGGATAAACGTTCATTCTCTAAGAGTGAAAAACCTTATCAGGAGGACCCATTCCTAAAAGACTTTGGTGCTACTGGTTTTGATGGGGCGTGATCCCTTTTCTGGGGGACTTGTCGGCGTGGTTAAGAGGAAGAAAGATGTGCCTAAACCAGCTGACTTCCATGATCCTGTGAGGGAATCATTCGAGGCCGAACTTGAGAGAGTTCAGAAGATGCAAGAACAGGAACGACAGCGGGTTATTGAAGAACAAGAAAGAGCTTTGGAGTTGGCTCGAAGAGAAGAAGAGGAGAGAGTGCGACTAGCTAGAGAAAAAGAAGAACGACAGAGAAGACTTGAAGAAGAAGCCAGAGAAGCTGCTTGGAGAGCAGAACAGGAGCGACTAGAAGCCATACAAAGAGCTGAAGAGCAAAAGATAGCTAGAGAAGAGGAGAGACGCAGGATTGTTATGGAGGAAGAAAGGAGGAAACAAGCTGCCAAGCAAAAACTCTTAGAATTGGAAGAAAGGATTGCCAAGAGGCAGGCTGAATCAGCAAAGGGTGATAATTTTTCTGCTGTTACTGACGAGAAACTCTCAGGCATGCTGAAAGAAAAAGAAGCCATGAAGGAAGCAGAAATGGGTGATTGGGAAGATGGGGAAAGAATGGTGGAGAATATAACAACTTCATCATCATCTGATTTTTCTAATATGAATAGATCTTTTGAAATGGGTTCCAGGACTGAGTCATCTAGAGATGGTTCTGCTTTTCAAGACAGGGGAAAACCTGTTAATTCATGGAGAAGGGACATGTTTGAGAATAGAAACTATCTTTTCCAAGACCAGGAGAACGGGCACAACAGTCCCAGGCAAGATTCATCTATTGGTGCCAAAGCATTTTCTAGGAAAGAATACTATGGAGGATCTGGGTACATGTCTCCAAGGACATATTTTAAAGGCGGGGTTCAAGAGCCTCACATGGATGATTTTAATCATGTAAAAGGGCAGAGGTGGAATTTTCCCGGGGATGTAGATCATTATAGCAGAAATGCAGAGATGGATTCAGAATTTCATGAGAACTTCAGTGAAAAGTTTGGAGATGGTGGGTGGGGGCAGGGCCGTTCTCGGGGCAATCCTCATCAGTCTTACCCAGAACGGCTGTATCAGAATCCCGATGTGGATGAACTTTATTCCTTTGGGAGGTCAAGGTATTCCATGAGGCAACCTCGCGTTCTCCCTCCTCCACTTGCTTTCATGAACAAAATTTCCAATAGAGGCGAGAATGAACGTCCCGAGCCTTCCAGTTTTCTAGATAATGAGATGCAGTATAACCACACCACAAGAAGTGAATCTCCTATACAGATAGGGTATGATGTTGGTAGTCAGGAGAAGGTGGGACAACCTGAAATGATCAACGTTCAACAAGAGAATACAACGACCCAGGAGCATAAACTGGATAGGAAAGTCACATCGAGGTGCGACTCACAGTCTTCCCTTTCAGTTTCAAGCCCCCCAAATTCTCCAACTCATCTCTCTCATGATGACTTGGATGAATCTGAGGATAGTATGGTGATATCAGCTGCCGCAGAAGGTAAACATCCTTCTTTATCTGGGAATGAACCTGTTTCTGTAGCCGGGAACGAGAATATAATGACTGCCTCAAGTTCAATCTCAACAGGTGATGATGAAGAATGGGCTATTGAGAACAATCAACAGTTGCAGGAGCAAGAAGAATATGAGGATGAAGAGGGTTTCCAGGAAGAAGATGAAGTGCATGAAGGAGATGATATGAATATTGACTTGACCGAGGAGTTTGAAGATATCCATTTAGAGGAGAAAGGATCACCCTGCATGATGGATAACCTGATCCTAGGCTTTAATGAGGGCGTTGAAGTTAAAATGCCAAATGACGAATATGAAAGAAGTTCAAGGAATGAAGACATTTCATTTGCGATGCCGCAGGTTTCTATTGGAACTGTCGAAGAACAGGGATCTTTCGGTGGGATGCACAGTGATGAATCAGCCGTTCAACACGTTGATAGTTCTACTCAGGTAAGTATTGAGAAGGCAGTGCAAGATTTGACTATTGAGCCCAATACTTCAGCAGCATCTGATCTTTTAAATAATGGGGATGCTTCTAGTTGTTCTGGTCTACCTTCTCAGCATCAGATGCAGTCTTTGCATTCTTCTTCGGGTCAGTCTGTCATGTCTACGGCCTCTGCTGGACCGAGTCAGTCAGAGATACCCATTAAGCTCCAGTTTGGGCTATTTTCTGGTCCTTCTTTGATACCATCTCCAGTTCCAGCCATACAGATTGGTTCCATACAGATGCCTCTCCATCTACATCCTCAAGTTGGCCCTCCTATAACCCATATGCACCCATCACAGCCTCCTCTCTTCCAGTTTGGACAACTAAGATATACATCTCCAATCTCACAGGGGATCCTGCCATTGGCTCCTCAACCAATGTCTTTTGTTCAACCCAATATTCCCTCCCAATTTCCTGTTAATCAGAAGCCAGGAGGTCCTTTGCCCGTTCAAGCAGGCCAAGACAATTCTATTTGTGATTCTTTTAAAGGCAATGTTCCGTCTCTTTCCATGGATAATCAGCCAGGCCTGGTTCCTAGGCACTTGGATCTATCTCAGCAGAATGTATCGAAAGAGTAAATTCATTGCCAGTAAGAAAAAATGGAGAAAACAACGAACTGCCGTGTCAAGATCGAGGGGAAACTGCCAATATTGGTGAAAGTGATAATAGGTATGAATCAGGTACCCAAGAAGAACGCCGGGGGCAGCATGTTAAAGCTGTCAAGAGCTACACATCCTTTCAATCAGAAGGCCAGCCAGGAACTATGTCATCTCAATCCATTTCAAGAGAAAGAGATATAAGTGGATCAAAGGCTCCTGGCCCAGTATCTGGAAGTAAGGGGAAACAAATTGTCTTTACTGGCAGGAATTCTGGTTTGAGATCATCTTTTGCAACTCCTGTGACTTCTCGTGCGGAATCCAGTGGCTTTCAGAGAAGACCTCGACGTAATGTATCACGTACAGAGTTTCGGGTTTGGGAAAATGCTGATAGGGGGCGATCAGGCTTTGTTTCATCTAACCACTCAGGGCTAGATGATAAGTTAAATTTCAGTGGAAGGGCGACAGGAGTTCCTTCAAGAAATGGGTCTAAAAAGGGTGTTGCATTGAATAAACCATTGAAACAGACAGATCCTATTGGTTCGCAAGAGATTGATTCTGGAGGCAGAGCTGAAAAGGGAACTGGAAAAGAATCATCAACGAAGAGTCAGTACATCTCACGCTCTGGAGAGGGAAACCTGAAACGAAACATCTGTTCTGAAGAGGATATCGACGTTCCTTTGCAAAGCGGCATTGTGCGCGTATATGAACAACCTGGCATAGAAGCCCCTAGTGATGAAGATGACTTCATTGAGGTGAGATCGAAGAGGCAAATGCTGAATGATCGGCGTGAACAGAGAGAAAAAGAATTTAAGGCGAGGTCAAGGCTCATTAAGATGCCACGGAAACCTCGTCCTACTTTGCAAACTACTATGGTGTCAAGTAGCTCTAATAAAACATCTACACCATTTGGTGGAGAAGTAGCTAACCCCATCCACTCTGATTTTATTGGCACGGAGGGGCGAGGATTGGCAACCAAAGAAGTATCAGTGGGATTTAATGCCACGACAGCATCCCAACCATTGGCTCCAATTGGCACCCCTGCTGTGAATACTGATGTCCAGGCTGATATAAGATCCCAAACTATTATGTCTTCACATACAAGCTCCCTTCCTGTCATATCTAGTGGTGGAAAAAACCTTGGGCCGGGCTTAATATTTGATACCAAGAGTAAGGTTCTGGATAATGTTCAAACATCACTGGGCTCTTGGGGCAGTGCACGGACTAATCAACAGGTTATGAATCTAACACAGAGCCAACTTGACGAGGCCATGAAGCCTGCACGATTTGATACTCATGTTGTTTCTATCGGAGATCATACTAGCTCAATTGGCGAGCCAACCTTACCGTCTTCATCAATCTTGACAAAGGACAAATCATTTTCTTCCACAGCGAGCCCAGTTAACTCCCTGCTTGCTGGGGAGAAAATTCAATTTGGTGCGGTTACTTCTCCAACAATTCTCCCTTCAAGCAGTTGTGCTGTTTCACTTGCGATTGGCCCTCCGGGTTCAAGTAGGCCAGAGCTTCAGATGTCTCACAATCTTGCTGCAGCCGAGAATGATTGCTCTCTGTTCTTTGAAAAAGAGAAGCACCCCAACGAGTCAGGTGCTCGTTTACAAGATTGTGAGGCTGAGGCTGAAGCTGAAGCAGCTGCTTCAGCTGTTGCTGTTGCAGCTATCAGTAGTGACGAGATTGTTAGGAATGGGTTGAGTACATGCTCTGTCTCTGTTTCAGATACCAAAACATTTGGAGGGGCAGATATTGATGTGATAACCACAGGGGTGAATGATGAGCAGCAATTAGCAAGTCAATCAAGAGGTGAAGAGTCTCTGACAGTAGCACTCCCAGCTGATCTATCTGTTGAAACTCCCCCAATATCACTATGGCCACCGTTACAAAGTCCAAAAAATTATTCAAACCAGATGCTTTCACATTTCCCTGGAGGCCCACCTTCTCACTTTCCATTTTACGAGATGAACCCTATGTTGGGTGGCCCCATCTTTGCTTTTGGGCCCCATGATGAATCTGGTGGTACCCAATCACAATCCCAGAAGAGTAGCACAACAGGTTCAGGACCACATGGAACATGGCAACAATGTCATTCTGGTGTAGACTCATTCTATGGTCCTTCAGCAGGATTCACTGGTCCTTTTATTAGTCCATCTGGAGGGATCCGGGTGTTCAAGGTCCACCACACATGGTTGTATATAACCATTTTGCACCGGTCGGACAATTTGGACAAGTTGGGTTGAGTTTCATGGGTACAACTTACATACCATCTGGAAAGCAACCTGATTGGAAGCACAACCCTGCATCATCTGCCATGGGAATTAGTGAAACAGACATGAACAATATGAATATGGTTTCTGCACAGAGAAATCCTCCTAACATGCCGCCTCCTATTCAACACCTTGCTCCAGGGTCACCACTTCTTCCCATGGCTTCTCCTTTGGCTATGTTTGATGTATCTCCTTTCCAGTCATCTCCTGACATGTCGGTCCAAGCTCGATGGTCTCATGTTCCTGCTTCACCTCTTCATTCAATTCCTCTATCAATGCCAATGCAGCAACAAACAGAAATCGGTCTGCCTTTACAATTCAGCCATGGACAACCTGTTGATCAATCATTAACTTCCAACAGGTTCCCTGAGTCAAGAACTTCGACATCCTTGGACACAACTGGTGCACAGTTTCCAGATGAATTAGGTTTAGTCGACTCATCTAGTGGTGGAGCTTCAGCCCAGGGTGTTGTTACCAAGGTAGAAGCAGGAATGACGGAATCTGTTCAGAATGGCGTGAATAGCAGTAGCAGTAACAACGGTCAGAATCAGAATATGGTTTCTGGTTTCAAGTCTTCCCAGCAGAAGAATATGACAGGGTATGGTAATTATAACAGGGTGCCTCAGAAGAATGAATGGCCTCACCGGAGAATAGGATTTCAGGGTAGAAACAACCACCCTGTGGGTGGTGAAAAGAGCTTTCCTTCAAAGATGAAGCAAATTTATGTGGCGAAAACGAATACAAATGGGACAACATCTACAGCATTATGAAGGGTTTGACATCTGTCTCCCAAATTGAATAGTTTGGTTCGTTGTGGCAAAGCATATATTGCATTTGGTGGGGAAAGGGGAGATCGTATTTTTTTTTTCTTTTTTCTTTTCACGCGTGTTTACCAGCACTGGTCTTTGAACTGGTTTTGGGATGGAAATTTTGTGTAATTCTAAAAAGTGTTCTGTTGAGGAAGGGTGTGGATTGGGTTTAGCATATGGTATTAGATGCAGTGATTAAAAAAGTGGGGTTGTGTAAAAGTCAGGCGGGGTCGTGTCGGTTCGTCGGATCACTTAAATTGTGTTCTTTTTTGTGCCTAGAAACTAAATTATTTGACTTTGGGGTTGATATATTAAAAATTATTTCAGATCCTCCTGAAAAAAAAAAAAAAAAAAAAAAAAAAAAGT

>transcript_4 full_length_coverage=2;length=7792

GGGTCCGCCTATCGATTGAATTGCACAGAGCACTCTTCCCCTGCTTTGCTTTCTTTCCCTTCGTCTTCCTTCCCCTGCTTCTATAAATTTGTTTGCTTTTGTGTTCGTTTCGTTTCGTTTCCCCTGTTGTAAATTTGAGTCTCGAAGAATCCGCTGGGATTTTTCACCGGCGATAAGGATTTTCTTCGTTGATTCTCAAACCAAACCTCGTGATTTTCAAAGAATTTTCTCACAATTTCCCTGTCAATTTTCTGACTTTCCATCTGTGGAAGGAAATTTTTAGTCAAATAGATCTGTCGTTAGACGCCCGGTTGTGGATTAAGGATATCTCTAATCTCTATCGTTAGCTATCACTAATGTCGGCTTCTTCAGCTTCTGTCTTTAATCTCCAAAAGAACCCGTGTGTTGTTCCTTCGCTTAACCGTCCGTCTTTTAACCATCAACTCAATGTTTGTTCTGTAAGGCGCCATGGAAACAGAACAAACAGAGCCTCCTGTTACGCTAGCCGATGGAATGTTTTGGAGAATAGGTTTTTTGGAACGAGATTGCGGGAGTCTGGATCAGAAAGACTCCATCTTTGGCAATCGGACGTTTCCAGAAAGTCCTTAAAGCAGAGGGTCATGGTGCGATCCGCATTGTCCTTGGTTCCGGAGAAGCCCCTCGGTCTTTACGATCCTTCCATGGATAAGGATTCTTGTGGGGTTGGGTTCGTTGCGGAATTATCAGGCGAAGGCAGCCGGAAAACGGTGACTGATGCTTTGGAGATGTTGGTACGCATGTCACATAGAGGTGCTTGTGGCTGTGAAACAAATACCGGTGATGGAGCGGGTATTCTCGTGGCTCTTCCTCACGATTTCTTCAAGGTTGATATTGAGTTTGAGCTACCACCTCCAGGGGAGTATGCTGTCGGCATGTTCTTCTTGCCCACATCCGAGAGCCGAAGAGAACAGAGCAAAATTGTTTTTACTAAGGTTGCCGAGTCTCTTGGGCATACAGTTCTTGGGTGGCGGTCTGTCCCAACTGATAACTCAGGATTGGGCAAGTCTGCTTTGCAGACAGAACCCGTGATTGAACAAGTGTTCCTTACTTCCACTCCTAGGTCAAAAGCTGACTTGGAGCAACAGATGTACATTTTAAGAAGGGTTTCTATGGTTGCCATCCGAGCTGCTCTAAACCTTCAACATGGTGGTGTTAAGGACTTCTATATATGTTCTCTTTCCTCGAGGACTGTGGTTTACAAAGGTCAGCTAAAGCCCGACCAGTTGAAGCAATACTATTATGCAGATCTTGGCAATGAAAGGTTTACGAGCTACATGGCCCTGGTAAATATTTTATCCGTTTAACTTTTTTTCCTTTTCCACTGACTCCATACTTCACAAACAGCATGGGAACCACCGGTAACCGAAGAAGAGATGCAAGGTTAAGGAATGACTTCTGAGGCATTGAAGTTTGAATGATCTCTTAAGTTCTTCCCCTCCCTTGATTACTCTTTGAATTTGGATGCTGGATTGGATGCCTTGAGTGAGTTGGTAAGAAACTTCGTTATCAAGTAAATCATGTTTGAGTGCAGTGCTCTCTGTGATTGGATTTGAGGATGAGACCTCCACTTTGGTGGTTATCAAGTAAATTTTGTTGGGTCAAGATACTTCAGGGTTTTCCTTGATCCGTTCTGACACTTTGAAGGGCACTATTTTATGCTGGAAATATTTAATCATTGTCTCTAGTGTTTAATGGATTAATTATTTGTATAGGTACACTCTCGGTTCTCCACGAACACATTTCCTAGCTGGGATCGAGCTCAACCAATGCGTGTCTTGGGACATAATGGAGAAATTAACACACTCAGGGGCAATGTAAACTGGATGAAGGCTCGTGAGGGTCTATTGAAGTGCAAGGAACTTGGTCTTTCAAAAAATGAGATGAAGAAGCTTCTGCCTATTGTGGATGCCAGCTCATCTGATTCCGGAGCTTTTGATGGCGTTCTTGAGCTTTTGATTCGAGCTGGCAGAAGCCTCCCCGAGGCTATTATGATGATGATTCCTGAAGCATGGCAAAATGACAAGAACATTCATCCTGAACGGAAAGCCTTGTATGAATACTTCTCAGCTCTTATGGAGCCATGGGATGGACCGGCTCTTATATCATTTACTGATGGTCGCTATCTTGGAGCAACATTGGATCGAAATGGTTTACGCCCAGGTCGTTTTTACGTCACACATAGTGGTCGAGTAATAATGGCTAGTGAGGTTGGTGTGGTAGACATTGAACCCGAAGATGTGTGTAGGAAAGGAAGACTTAACCCAGGCATGATGCTTCTAGTGGATTTTGATAAACATATTGTTGTAGATGATGAGGCCTTGAAACAACAATATTCACTAGCAAGGCCTTATGGGGAGTGGCTTAAGAGGCAGAAAATAGAACTTAAGGACATTGTTGAATCTGTTCATGAATCTGATCGGATTTCTCCAACTATTGCTGGAGTGGTGCAGGCATCTAGCAATGACGATGACATGGAAAATATGGGAATTCATGGGTTATTGGCTCCATTAAAGGCGTTTGGTTACACAGTCGAAGCCTTGGAGATGTTGTTACTTCCTATGGCAAAAGATGGTAGTGAGGCCCTAGGTTCAATGGGAAATGATGCTCCTCTAGCGGTAATGTCTAACAGAGAGAAGCTGACGTTTGAATATTTCAAACAAATGTTCGCACAAGTCACAAACCCCCCTATTGATCCAATCCGGGAGAAGATTGTTACTTCCATGGAATGCATGATTGGCCCAGAAGGTGATCTCACAGAAACTACTGAAGAACAATGTCGTCGTCTTTCATTAAAAGGACCTCTTTTATCCGTTGAAGAAATGGAAGCAATTAAAAAGATGAATTACAGGGGTTGGAGAAGCAAAGTTCTTGATATCACTTATTCCAAAGACAGTGGTAAGAAAGGATTAGAGGAGACCCTAAATAGGATCTGTTATGAAGCGCGGGAGGCAATTAAAGAAGGTTACACATTACTGGTGCTTTCTGACAGAGCCTTTTCTTCAAAGCGTGTTGCTGTGAGCTCCCTTTTGGCTGTTGGGGCTGTTCATCATCATTTGGTTAAAAAGCTAGAGCGCACTCGAATTGGTTTAATAGTTGAATCTGCTGAACCACGTGAAGTTCATCATTTCTGTACACTGGTTGGATTTGGTGCAGATGCTATATGCCCATATTTGGCTATAGAAGCGATTTGGAGGCTACAGGTTGATGGAAAGATTCCACCTAAAGCAAGTGGTGAGTTTTATTTGAAGGATGAGTTGGTTAAGAAGTACTTTAAAGCAAGCAACTATGGAATGATGAAGGTTCTTGCTAAAATGGGGATTTCAACTTTGGCCTCTTACAAGGGTGCTCAGATTTTTGAAGCTCTGGGTCTTTCTTCAGAAGTGGTTGAAAGATGTTTTGCTGGAACTCCAAGTCGAGTTGAGGGTGCAACATTTGAAATGCTTGCTCGTGATGCACTTCATTTGCATGAAATGGCATTTCCCAAACGTTCTTTGCCTATTGGAAGTGCTGAAGCTGTAGCGTTACCCAATCCCGGGGACTATCACTGGAGGAAAGGTGGCGAGATTCACCTGAATGATCCTCTTGCCATAGCCTCTCTTCAAGAGGCTGCTAGAACTAATAGTGTTTCTGCCTATAAAGAATACTCCAAGCGCGTTCAGGAACTGAATAAAAGCTGCAATTTGCGTGGGCTTCTGAAGTTCAAAGAGTCTGCAGTGAAGGTTCCCTTGGATGAGGTGGAATCAGCCAGTGAGATTGTGAAAAGGTTTTGTACTGGAGCCATGAGTTATGGTTCGATATCATTGGAGGCGCACACCACTCTGGCTATTGCTATGAATAAAATTGGAGGGAAGTCAAACACAGGTGAGGGAGGTGAGAATCCGTCCAGATTGGTGCCCCTTCCAGATGGTTCAATGAATCCGAAGAGGAGTGCAATTAAGCAGGTTGCGAGTGGAAGATTTGGAGTTTCAAGCTATTACCTTACAAATGCCGATGAACTGCAGATAAAAATGGCCCAGGGGGCCAAGCCTGGTGAAGGAGGTGAACTTCCTGGCCACAAGGTTATTGGAGATATTGCTGTCACTAGGAATTCCACTGCTGGTGTTGGACTAATCAGCCCACCTCCTCATCACGATATCTATTCCATTGAAGACCTTGCCCAATTAATTCATGATCTTAAGAACTCCAATCCAGGGGCTCGAATCAGTGTAAAGTTGGTATCAGAAGCTGGTGTTGGAGTAATTGCTAGTGGAGTTGTCAAGGGGCATGCTGATCATGTCTTGATCTCAGGTCATGATGGAGGTACAGGGGCTTCTCGATGGACTGGTATTAAGAGTGCTGGGCTTCCCTGGGAACTTGGTCTTGCTGAGACGCATCAAACTCTTGTTGCTAATGACCTTCGTGGTCGAACAACTCTCCAAACAGATGGCCAACTGAAAACAGGAAGAGATGTGGCCATTGCTGCACTTCTCGGTGCGGAGGAGTTTGGCTTCAGCACAGCACCCCTCATTACACTTGGCTGCATAATGATGAGGAAGTGCCACAAAAACACCTGCCCGGTTGGCATTGCAACCCAAGATCCAGTTCTCCGAGAGAAGTTTGCTGGAGAACCCGAGCATGTCATAAACTTTTTCTTTATGCTAGCAGAAGAGCTGAGGGAGATTATGTCTCAACTTGGATTCCGCACAATCACTGAGATGATTGGCCGTTCTGATATGCTGGAGGTTGATAAAGAAGTGACAAAGAACAATGAGAAGCTAGAGAATATTGATCTCTCCCTATTACTTAGACCTGCTGCTGACATTCGGCCTGAAGCTGCTCAATATTGTGTCCAGAAACAAGATCATGGCTTGGACATGGCTTTAGACCAAAAACTTATAGCTCTTTCCAGTGCTGCTTTAGAAAAAGGTCTTCCTGTATACATTGAAACACCAATATGCAATGTGAACCGTGCAGTTGGGACAATGCTTAGCCATGAAGTGACTAAACGCTATGAGTTGGCAGGGCTTCCTGCAGACACCATCCATATCAAACTCAATGGAAGTGCAGGCCAGAGTCTTGGGGCTTTTCTCTGCCCTGGCATCATGCTGGAACTCGAAGGTGATAGCAATGACTATGTTGGTAAAGGACTATCAGGTGGCAAAATAGTAGTTTATCCTCCAAGAGCAAGCCAGTTTGATCCAAAGGAAAACATTGTTATAGGTAATGTGGCTCTGTATGGGGCAACTAGTGGTGAAGCATATTTTAATGGGATGGCAGCAGAAAGATTTTGTGTTCGTAATTCAGGGGTGAGAGCAGTTGTAGAAGGTATAGGTGACCATGGGTGCGAATACATGACAGGAGGGACAGTGATTGTTCTTGGAAAAACAGGAAGGAATTTTGCTGCAGGTATGAGTGGTGGTGTTGCTTATGTTCTTGACGTGGATGGGAAGTTCCGATCTCGATGCAATACCGAGCTGGTAGATCTTGATAAAGTTGAAGATGAAGAGGATATTCTGACTCTCAGGATGATGATACAACAACATCAGCGTCACACAGGCAGCCAGCTAGCCAGAGAAGTGCTCGCAGACTTTGAAAATCTTCTGCCAAAATTTATCAAGGTCTTCCCCAGGGATTATAAACGTGTTCTTGCAAGCATGAAGTCTGAGAAAGCCTCTGAAGAAGTGTCTGAGAAGGCTGCTAAAGAAACTGAGGATCAAGATGAGGCAGAGTTAAAGGAGAAAGATGCTTTTGAAGAACTTAAGAAGCTCGCAGCTGCATCAGTGAATGAGAAACCCAGTCAGAAGGTAGAGGAACCTAAGAGGCCTAGTCGTGTTAAGGATGCTGTCAAACATCGAGGTTTTGTTGCCTATGAGCGTGAGGGTGTTTCCTACAGAGATCCTCATATTCGGATGAACGACTGGGATGAGGTTATGGAACAGTCTAAACCTCCCCTGCTGTTGAAGACCCAATCAGCCCGTTGCATGGATTGTGGCACTCCCTTCTGTCATCAGGAGAAGTCTGGTTGTCCTCTTGGGAACAAGATACCTGAATTCAATGAGTTGGTGTATCAAAATAGATGGCGTGAAGCATTAGACCGGCTCCTAGAGACAAATAATTTCCCAGAGTTCACTGGTCGAGTGTGCCCTGCACCATGTGAAGGCTCTTGTGTTCTTGGTATTATTGAGAATCCCGTGTCCATCAAAAGCATAGAGTGTGCCATTATAGACAAGGCTTTTGAGGAGGGTTGGATGGTGCCACGGCCTCCACTTGAGAGAACAGGGAAAAGAGTTGCTATTGTTGGAAGTGGACCATCTGGCTTGGCTGCTGCTGATCAGCTGAACAAAATGGGCCATTTTGTTACTGTCTTTGAGCGTGCCGATAGAATTGGAGGGCTTATGATGTATGGAGTTCCCAATATGAAGGCTGGCAAGTTGGATATTGTTCAACGACGGGTTAACCTTATGGCCGAGGAAGGCGTCAATTTTGTGGTTAATGCTAATATTGGAGTAGACCCCTTATACTCTGTTGATCGGCTTAGGGAGGAGAATGATGCCATTGTTTTGGCAGTAGGAGCAACAAAACCAAGGGACCTTCCCGTTCCAGGACGGGAATTCTCAGGAATCCACTTTGCCATGGAGTTCCTTCATGCAAACACCAAGAGCTTGCTTGACAGCAATCTCGAAGATGGTAACTATATTTCTGCGAAGGGCAAGAAGGTAGTGGTCATTGGTGGAGGTGACACTGGTACAGATTGCATAGGGACATCAATTCGTCATGGCTGCAGTAGCATCATAAATCTAGAGCTTCTCCCTGTGCCACCACAAAGCAGGGCTCCTGGAAACCCTTGGCCCCAGTGGCCTCGCATATTCCGTGTAGATTATGGACACCAAGAAGCTGCTACAAAGTTTGGTAAAGACCCGAGATCTTATGAGGTACTGACCAAGAAGTTCGTTGGAGATGAAAATGGGGTTGTGAAGGGACTTGTAGTGGTAAAAGTTCAGTGGGAGAAGGATGCAAGTGGGAGGTTTCAGTTCAAGGAAGTAGAAGGCTCCGAGGAAATGATTGAAGCGGACCTTGTCCTGCTAGCCATGGGTTTCCTTGGACCCGAGTCGAACGTAGCTGAGAAGCTGGGCTTGGAGTGTGACAATCGGTCGAACTTCAAAGCAGAGTATGGCCGCTTTGCAACCAACGTTGATGGGATCTTTGCTGCTGGGGATTGCCGGCGGGGCCAGTCTCTGGTTGTATGGGCTATCTCAGAAGGTCGACAAGCTGCTTCACAAGTAGACAAGTATCTTGTAAGAGAAGAAGAGGACCTCACTATTAGTGAAGAGGACTTCAAGAAGCAGCAGCAAGGCAGCAGCAAATACACATTAATGACGTAGATGATCTCTCTTTTTTCCTTATTTTTTCTTTTACATAGTGCTGAAGAAAACAAAGACCAATACCATCAACAAAAGTGGTTCACAATCCCGAGCACAGCTGTTGAATTATTTGTGTATGACAATTCATGGGTTCGGAGAGCTCTTCTGGATTTCTACCGAGGGGTTGAGAAGAAGCAGAGCTAAGAGTGGGTTTCTTGTTGGTTGAGGTGAAGTGTGTGAGTAGGTTTAATTTTCTAAATATATTTGTTTATAAGTTTCACACCCTTGTTTGAGGTGTTAAGCTCCTGTGGTGGTTAAGAATAATGTATAGTTTTGTTTCCCAATCTTATGATTGATAGTAATATATGGGGAATTTGTGGTAAAAAAAAAAAAAAAAAAAAAAAAAAAAAAGT

>transcript_5 full_length_coverage=2;length=7585

GGGACTGCTCTTCCCTTCTCTCTCCGTCTCCGCCTATCGATTGAATTGCACAGAGCACTCTTCCCCTGCTTTGCTTTCTTTCCCTTCGTCTTCCTTCCCTGCTTCTATAAATTTGTTTGCTTTTGTGTTCGTTTCGTTTCGTTTCGTTTCCCCTGTTGTAAATTTGAGTCTCGAAGAATCCGCTGGGATTTTTCACCGGCGATAAGGATTTTCTTCGTTGATTCTCAAACCAAACCTCGTGATTTTCAAAGAATTTTCTCACAATTTCCCTGTCAATTTTCTGACTTTCCATCTGTGGAAGGAAATTTTTAGTCAAATAGATCTGTCGTTAGACGCCCGGTTGTGGATTAAGGATATCTCTAATCTCTATCGTTAGCTATCACTAATGTCGGCTTCTTCAGCTTCTGTCTTTAATCTCCAAAAGAACCCGTGTGTTGTTCCTTCGCTTAACCGTCCGTCTTTTAAACCATCAACTCAATGTTTGTTCTGTAAGGCGCCATGGAAACAGAACAAACAGAGCCTCCTGTTACGCTAGCCGATGGAATGTTTTGGAGAATAGGTTTTTTGGAACGAGATTGCGGGAGTCTGGATCAGAAAGACTCATCTTTGGCAATCGGACGTTTCCAGAAAGTCCTTAAAGCAGAGGGTCATGGTGCGATCCGCATTGTCCTTGGTTCCGGAGAAGCCCCTCGGTCTTTACGATCCTTCCATGGATAAGGATTCTTGTGGGGTTGGGTTCGTTGCGGAATTATCAGGCGAAGGCAGCCGGAAAACGGTGACTGATGCTTTGGAGATGTTGGTACGCATGTCACATAGAGGTGCTTGTGGCTGTGAAACAAATACCGGTGATGGAGCGGGTATTCTCGTGGCTCTTCCTCACGACTTCTTCAAGGTTGATATTGAGTTTGAGCTACCACCGTCCAGGGGAGTATGCTGTCGGCATGTTCTTCTTGCCCACATCCGAGAGCCGAAGAGAACAGAGCAAAATTGTTTTTACTAAGGTTGCCGAGTCTCTTGGGCATACAGTTCTTGGGTGGCGGTCTGTCCCAACTGATAACTCAGGATTGGGCAAGTCTGCTTTGCAGACAGAACCCGTGATTGAACAAGTGTTCCTTACTTCCACTCCTAGGTCAAAAGCTGACTTGGAGCAACAGATGTACATTTTAAGAAGGGTTTCTATGGTTGCCATCCGAGCTGCTCTAAACCTTCAACATGGTGGTGTTAAGGACTTCTATATATGTTCTCTTTCCTCGAGGACTGTGGTTTACAAAGGTCAGCTAAAGCCCGACCAGTTGAAGCAATACTATTATGCAGATCTTGGCAATGAAAGCATGGGAACCACCGGTAACCGAAGAAGAGATGCAAGGTTAAGGAATGACTTCTGAGGCATTGAAGTTTGAATGATCTCTTAAGTTCTTCCCCTCCTTGATTACTCTTTGAATTTGGATGCTGGATTGGATGCCTTGAGTGAGTTGGTACACTCTCGGTTCTCCACGAACACATTTCCTAGCTGGGATCGAGCTCAACCAATGCGTGTCTTGGGACATAATGGAGAAATTAACACACTCAGGGGCAATGTAAACTGGATGAAGGCTCGTGAGGGTCTATTGAAGTGCAAGGAACTTGGTCTTTCAAAAAATGAGATGAAGAAGCTTCTGCCTATTGTGGATGCCAGCTCATCTGATTCCGGAGCTTTTGATGGCGTTCTTGAGCTTTTGATTCGAGCTGGCAGAAGCCTCCCCGAGGCTATTATGATGATGATTCCTGAAGCATGGCAAAATGACAAGAACATTCATCCTGAACGGAAAGCCTTGTATGAATACTTCTCAGCTCTTATGGAGCCATGGGATGGACCGGCTCTTATATCATTTACTGATGGTCGCTATCTTGGAGCAACATTGGATCGAAATGGTTTACGCCCAGGTCGTTTTTACGTCACACATAGTGGTCGAGTAATAATGGCTAGTGAGGTTGGTGTGGTAGACATTGAACCCGAAGATGTGTGTAGGAAAGGAAGACTTAACCCAGGCATGATGCTTCTAGTGGATTTTGATAAACATATTGTTGTAGATGATGAGGCCTTGAAACAACAATATTCACTAGCAAGGCCTTATGGGGAGTGGCTTAAGAGGCAGAAAATAGAACTTAAGGACATTGTTGAATCTGTTCATGAATCTGATCGGATTTCTCCAACTATTGCTGGAGTGGTGCAGGCATCTAGCAATGACGATGACATGGAAAACATGGGAATTCATGGGTTATTGGCTCCATTAAAGGCGTTTGGTTACACAGTTGAAGCCTTGGAGATGTTGTTACTTCCTATGGCAAAAGATGGTAGTGAGGCCCTAGGTTCAATGGGAAATGATGCTCCTCTAGCGGTAATGTCTAACAGAGAGAAGCTGACGTTTGAATATTTCAAACAAATGTTCGCACAAGTCACAAACCCCCCTATTGATCCAATCCGGGAGAAGATTGTTACTTCCATGGAATGCATGATTGGCCCAGAAGGTGATCTCACAGAAACTACTGAAGAACAATGTCGTCGTCTTTCATTAAAAGGACCTCTTTTATCCGTTGAAGAAATGGAAGCAATTAAAAAGATGAATTACAGGGGTTGGAGAAGCAAAGTTCTTGATATCACTTATTCCAAAGACAGTGGTAAGAAAGGATTAGAGGAGACCCTAAATAGGATCTGTTATGAAGCGCGGGAGGCAATTAAAGAAGGTTACACATTACTGGTGCTTTCTGACAGAGCCTTTTCTTCAAAGCGTGTTGCTGTGAGCTCCCTTTTGGCTGTTGGGGCTGTTCATCATCATTTGGTTAAAAAGCTAGAGCGCACTCGAATTGGTTTAATAGTTGAATCTGCTGAACCACGTGAAGTTCATCATTTCTGTACACTGGTTGGATTTGGTGCAGATGCTATATGCCCATATTTGGCTATAGAAGCGATTTGGAGGCTACAGGTTGATGGAAAGATTCCACCTAAAGCAAGTGGTGAGTTTTATTTGAAGGATGAGTTGGTTAAGAAGTACTTTAAAGCAAGTAACTATGGAATGATGAAGGTTCTTGCTAAAATGGGGATTTCAACTTTGGCCTCTTACAAGGGTGCTCAGATTTTTGAAGCTCTGGGTCTTTCTTCAGAAGTGGTTGAAAAGATGTTTTGCTGGAACTCCAAGTCGAGTTGAGGGTGCAACATTTGAAATGCTTGCTCGTGATGCACTTCATTTGCATGAAATGGCATTTCCCAAACGTTCTTTGCCTATTGGAAGTGCTGAAGCTGTAGCGTTACCCAATCCCGGGGACTATCACTGGAGGAAAGGTGGCGAGATTCACCTGAATGATCCTCTTGCCATAGCCTCTCTTCAAGAGGCTGCTAGAACTAATAGTGTTTCTGCCTATAAAGAATACTCCAAGCGCGTTCAGGAACTAAATAAAAGCTGCAATTTGCGTGGGCTTCTGAAGTTCAAAGAGTCTGCAGTGAAGGTTCCCTTGGATGAGGTGGAATCAGCCAGTGAGATTGTGAAAAGGTTTTGTACTGGAGCCATGAGTTATGGTTCGATATCATTGGAGGCGCACACCACTCTGGCTATTGCTATGAATAAAATTGGAGGGAAGTCAAACACAGGTGAGGGAGGTGAGAATCCGTCCAGATTGGTGCCCCTTCCAGATGGTTCAATGAATCCGAAGAGGAGTGCAATTAAGCAGGTTGCGAGTGGAAGATTTGGAGTTTCAAGCTATTACCTTACAAATGCCGATGAACTGCAGATAAAAATGGCCCAGATGATGCCGTGTCTGCAAAGCACCTCTTGGTGTCTTTGCATCTATAACTGATTCCACATTTGGCTTTTGGGGGCCAAGCCTGGTGAAGGAGGTGAACTTCCTGGCCACAAGGTTATTGGAGATATTGCTGTCACTAGGAATTCCACTGCTGGTGTTGGACTAATCAGCCCACCTCCTCATCACGATATCTATTCCATTGAAGACCTTGCCCAATTAATTCATGATCTTAAGAACTCCAATCCAGGGGCTCGAATCAGTGTAAAGTTGGTATCAGAAGCTGGTGTTGGAGTAATTGCTAGTGGAGTTGTCAAGGGGCATGCTGATCATGTCTTGATCTCAGGTCATGATGGAGGTACAGGGGCTTCTCGATGGACTGGTATTAAGAGTGCTGGGCTTCCCTGGGAACTTGGTCTTGCTGAGACGCATCAAACTCTTGTTGCTAATGACCTTCGTGGTCGAACAACTCTCCAAACAGACGGCCAACTGAAAACAGGAAGAGATGTGGCCATTGCTGCACTTCTCGGTGCGGAGGAGTTTGGCTTCAGCACAGCACCCCTCATTACACTTGGCTGCATAATGATGAGGAAGTGCCACAAAAACACCTGCCCGGTTGGCATTGCAACCCAAGATCCAGTTCTCCGAGAGAAGTTTGCTGGAGAACCCGAGCATGTCATAAACTTTTTCTTTATGCTAGCAGAAGAGCTGAGGGAGATTATGTCTCAACTTGGATTCCGCACAATCACTGAGATGATTGGCCGTTCTGATATGCTGGAGGTTGATAAAGAAGTGACAAAGAACAATGAGAAGCTAGAGAATATTGATCTCTCCCTATTACTTAGACCTGCTGCTGACATTCGGCCTGAAGCTGCTCAATATTGTGTCCAGAAACAAGATCATGGCTTGGACATGGCTTTAGACCAAAAACTTATAGCTCTTTCCAGTGCTGCTTTAGAAAAAGGTCTTCCTGTATACATTGAAACACCAATATGCAATGTGAACCGTGCAGTTGGGACAATGCTTAGCCATGAAGTGACTAAACGCTATGAGTTGGCAGGGCTTCCTGCAGACACCATCCATATCAAACTCAATGGAAGTGCAGGCCAGAGTCTTGGGGCTTTTCTCTGCCCTGGCATCATGCTGGAACTCGAAGGTGATAGCAATGACTATGTTGGTAAAGGACTATCAGGTGGCAAAATAGTAGTTTATCCTCCAAGAGCAAGCCAGTTTGATCCAAAGGAAAACATTGTTATAGGTAATGTGGCTCTGTATGGGGCAACTAGTGGTGAAGCATATTTTAATGGGATGGCAGCAGAAAGATTTTGTGTTCGTAATTCAGGGGTGAGAGCAGTTGTAGAAGGAATAGGTGACCATGGGTGCGAATACATGACAGGAGGGACAGTGATTGTTCTTGGAAAAACAGGAAGGAATTTTGCTGCAGGTATGAGTGGTGGTGTTGCTTATGTTCTTGACGTGGATGGGAAGTTCCGATCTCGATGCAATACCGAGCTGGTAGATCTTGATAAAGTTGAAGATGAAGAGGATATTCTGACTCTCAGGATGATGATACAACAACATCAGCGTCACACAGGCAGCCAGCTAGCCAGAGAAGTGCTCGCAGACTTTGAAAATCTTCTGCCAAAATTTATCAAGGTCTTCCCCAGGGATTATAAACGTGTTCTTGCAAGCATGAAGTCTGAGAAAGCCTCTGAAGAAGTGTCTGAGAAGGCTGCTAAAGAAACTGAGGATCAAGATGAGGCAGAGTTAAAGGAGAAAGATGCTTTTGAAGAACTTAAGAAGCTCGCAGCTGCATCAGTGAATGAGAAACCCAGTCAGAAGGTAGAGGAACCTAAGAGGCCTAGTCGTGTTAAGGATGCTGTCAAACATCGAGGTTTTGTTGCCTATGAGCGTGAGGGTGTTTCCTACAGAGATCCTCATATTCGGATGAACGACTGGGATGAGGTTATGGAACAGTCAAAACCTCCCCTGCTGTTGAAGACCCAATCAGCCCGTTGCATGGATTGTGGCACTCCCTTCTGTCATCAGGAGAAGTCTGGTTGTCCTCTTGGGAACAAGATACCTGAATTCAATGAGTTGGTGTATCAAAATAGATGGCGTGAAGCATTAGACCGGCTCCTAGAGACAAATAATTTCCCAGAGTTCACTGGTCGAGTGTGCCCTGCACCATGTGAAGGCTCTTGTGTTCTTGGTATTATTGAGAATCCCGTGTCCATCAAAAGCATAGAGTGTGCCATTATAGACAAGGCTTTTGAGGAGGGTTGGATGGTGCCACGGCCTCCACTTGAGAGAACAGGGAAAAGAGTTGCTATTGTTGGAAGTGGACCATCTGGCTTGGCTGCTGCTGATCAGCTGAACAAAATGGGCCATTTTGTTACTGTCTTTGAGCGTGCCGATAGAATTGGAGGGCTTATGATGTATGGAGTTCCCAATATGAAGGCTGGCAAGTTGGATATTGTTCAACGACGGGTTAACCTTATGGCCGAGGAAGGTGTCAATTTTGTGGTTAATGCTAATATTGGAGTAGACCCCTTATACTCTGTTGATCGGCTTAGGGAGGAGAATGATGCCATTGTTTTGGCAGTAGGAGCAACAAAACCAAGGGACCTTCCCGTTCCAGGACGGGAATTCTCAGGAATCCACTTTGCCATGGAGTTCCTTCATGCAAACACCAAGAGCTTGCTTGACAGCAATCTCGAAGATGGTAACTATATTTCTGCGAAGGGCAAGAAGGTAGTGGTCATTGGTGGAGGTGACACTGGTACAGATTGCATAGGGACATCAATTCGTCATGGCTGCAGTAGCATCATAAATCTAGAGCTTCTCCCTGTGCCACCACAAAGCAGGGCTCCTGGAAACCCTTGGCCCCAGTGGCCTCGCATATTCCGTGTAGATTATGGACACCAAGAAGCTGCTACAAAGTTTGGTAAAGACCCGAGATCTTATGAGGTACTGACCAAGAAGTTCGTTGGAGATGAAAATGGGGTTGTGAAGGGACTTGTAGTGGTAAAAGTTCAGTGGGAGAAGGATGCAAGTGGGAGGTTTCAGTTCAAGGAAGTAGAAGGCTCCGAGGAAATGATTGAAGCGGACCTTGTCCTGCTAGCCATGGGTTTCCTTGGACCCGAGTCGAACGTAGCTGAGAAGCTGGGCTTGGAGTGTGACAATCGGTCGAACTTCAAAGCAGAGTATGGCCGCTTTGCAACCAACGTTGATGGGATCTTTGCTGCTGGGGATTGCCGGCGGGGCCAGTCTCTGGTTGTATGGGCTATCTCAGAAGGTCGACAAGCTGCTTCACAAGTAGACAAGTATCTTGTAAGAGAAGAAGAGGACCTCACTATTAGTGAAGAGGACTTCAAGAAGCAGCAGCAAGGCAGCAGCAAATACACATTAATGACGTAGATGATCTCTCTTTTTTCCTTATTTTTTCTTTTACATAGTGCTGAAGAAAACAAAGACCAATACCATCAACAAAAGTGGTTCACAATCCCGAGCACAGCTGTTGAATTATTTGTGTATGACAATTCATGGGTTCGGAGAGCTCTTCTGGATTTCTACCGAGGGGTTGAGAAGAAGCAGAGCTAAGAGTGGGTTTCTTGTTGGTTGAGGTGAAGTGTGTGAGTAGGTTTAATTTTCTAAATATATTTGTTTATAAGTTTCACACCCTTGTTTGAGGTGTTAAGCTCCTGTGGTGGTTAAGAATAATGTATAGTTTTGTTTCCCAATCTTATGATTGATAGTAATATATGGGGAATTTGTGGTAAAAAAAAAAAAAAAAAAAAAAAAAAAAAGT

>transcript_6 full_length_coverage=2;length=7604

GGGCCGAATCCGTTTAAAAAAATTAACGTGCCTTTTTCCTTGAAATTATAAAGCGTCTCGCTTTCGAAGACCAACCCTAATCTTTGATATATTTCAGTTCTTTGCAACGATGTTGTAGCATTTGTCATACCTGAATCTCTTCAGTGTTCGATTAAAAAGGTTTTAATGAAGAAATCGGCACTGGGGGATTTGGATGAGTTCTGTGCAATTAGACCAGAATGCCAAGGCGACGTTCCAAAGACCCGTTTTAAGCCCAGGGTAGGAAGAACTCTTAGTGCAAGAAGATGGAATGCTGCATTCTCTCAAGATGGTCTTTTGGATATAGCGAAAGTGCTTAGACGAATTCAGAGAGGGGGTGTTCATCCATCAATTAAACAAGAAGTCTGGGAGTTCTTGTTAGGTTGTTTTGATCCCAATAGCACCTTTGACGAACGGAATCGGCTCAGGCAACATCGGAGAGAGCAGTATGACTCATGGAAATCTGAATGTCAGAAGATGGTACCTATTATTGGTAGTGGGAAAATCATTACAGCAAATATCATCCCTGAAGATGGTCAAGCTATACAGAATCCAACCAATGGGGATTCTCAGGATAACAATGGAGCTATGCCGGCATCCTCTCTCACTTCCTCTTTGGATCACAGGGAACATGTAAATGATGCCGTTTTAAACAAGAAAGTGACTCAGTGGAAGCTTATTTTGTCTCAAATTGGTTTGGATGTTGATCGAACTGACCGAACGCTTGTCTTTTATGAAAATGAAGTCAATCGAGCAAAACTCTGGAATATTCTTGCAGTTTATGCTTGGGTGGATAACGATATTGGCTACTGTCAAGGTATGAATGACATTTGCTCTCCAATGGTAATTCTTTTTGAAAATGAAGCAGATGCCTTTTGGTGTTTTGAGCGCGCAATGCGCCGGCTGGTATGCATCATTTTCCTTATTAAAGGTGACACTATTGTTTTAGTAACAGATGTCATGCAGCTTTGTTTAGACTTTTAGTATTTCTTTCCCATGGATTGATTCTATTTTAGTCTTACTCAGTCAATAATTTGGTTTTCCTTTAAGGGCTAATAAATATAACGAGATGGATTTACAGTCCATTACTTAACACTTTCATTTTCATTAATTCAATTTTAGTAGACTTGTTCTTTGTACACCTTCACAGACTAAATTACTTTTGAAGCGAGCATTTGTTTTTTCTTTTACAGTATCTAGCTTGAAGATTAATAAAGCCGTATACAAGCAGTTTAGTCTGTGCTGGTGGAAAGCGTTAGTGAATTAAGAAGGCTTTCAGAAATAAAATTATCTGCACCACTTGTTTTCCATATTTCGTCATGTTTTGGTGTTTGGAAAATCTACCGCCTTTTGTGACAATCAGGAATGAAGGTTGATGATTGAAATTTGCCTTGTAATATCAAGCTTTAAACCATTTACTTGCACAAGTGCTAATGTCGTCATACGGATTTTAAGAAGTCAACTTCTCTATAATGATCAAGTATCACAAATTTTTTCCTTATACATACCTAATTTGTGCCCTCATGTACCTAGTGAGCACAGTACAATTTACTTTTTGAAATAAATTAATAATAATAACAATTGTACTTGAAATAAATAAAAAAAGGATGTGCAAACCCCATGTAGTATACAAACATAAACAAAACAGAAAGGAGCTTCAAATTTAAATTTCTAAAGACCCATTGAATTCTTAAACGGCAATCTTAAACTATTGATTTTAAAGCACGAGCTTTTTGGGGAATTGAATCTTTAATATATATATATATATGTACGCACACACAGAGACAGGAATTCTTCCTAAAATACTCCTCACACTTCTTCCTAAAATACTCCTCACACTTCTAATTATATATAATGGCGTTCTTTGCAAAACCCAAAGAATACCAAAGCAAGAAGATAAAAGATCACAACTCCAAGTTAACCTCACATAGCAAAAAGGTGCCACCATTTTGCACATGGCGCATTTATCTGGGATGGCTCAACTTCTCCTAGTCATTACATCAGCTGTTTAGGATTTGTCCAAAACCCACCCAAACAAAGAACACTTATTTTAAGGGACTGATGGAACTCAGTCTCCTTAAATGGGAAAAAAACCCTCTACTCTTGGTAGCCAAGATGTCAAATAATGATATAGTCTTCCTGTTACAGGACAAAATTAGACAAAATTATAAAGTTCTTAATTTTGAGTCCTTAGTGTTTGTAGTTTCTTTTATTAGTTTCCAATTTTATGTTATTTCTATTCTTCATTCCTTATTAGATTAGGATTAGTAATATTTCTATTTCTATGATTGACTTGAGTGGCAAACTAAGTCTTATGAGAAAATTATCCAAAAAAATTCTTATTAAAATTGATGATATTTGAAATTCTAGTTTCATATTATGTTTTACTTTTCGAGCAAGCATTATGAATTACTTTACCTATATATAGGCCGGGTTGTAGCCTTTAGAATTCAGAGTACTATTTAATAAATTTGAAATTTCTTTGAGAGCAATTTCTTTGAGTTTTTATATGTGAGGAGTCCTTGCCATTATTCTTGTGAGGAATCCAAGGATTTTGCAGATTTATTCCTTGTGGAATCAAGGTCCTTTAAGGATGGGTTATATCTTAGTTTTTGAAGATTTTTAGGGGGAATCTCTTTCTTTTTCTCTTTATTTTGTGAGTTGAGAATTGTCCTATGATACTTGCGCCAAAGCATAGTTACACGGAACACGGTTTTCTCCGGTACTTGGTAAGGGGATGTATATGTGGTAAGCTATTCTGTATGGTAGGCACTTTGTTAAAGGTGGGCTTCTTTTGTATCTTAGTACTTTATTTTGGTATTATACTTACCCCAAAATATATTTTCTCTCGAAAAAACACACAACTAGTGTCCTACCAAAGTTCAACCTATAATATAAAAACACAACACCCAATTAAATGAGGGAAATTTCGTATGTTCATAACAGCGTAAAAAGAATTTGGTATTAACTTGTCTTGTCCAAATTGGAGCAAGACGATTTATTTATACTCACAAATAATTACAAGAGAGACTAAAATACCCCTGGGTTGAATCCAGGGCAATAGAAATGAAATAAGCACACCAAAAGAAATAAAATAAGGCCCAATTAACTACGTATTCCAACACCCCCCCTCAAGCTGGTGAGTAGATATCTATAAGGCCAAGCTTGCTAATAGAAGTTCTTAACAAGGAAGGACCCACTGGCTTAGTCAACATATCTGCCATCTGATTCTTGGAGGGAATATAGAATGGAGAGACAATACCATTGCGGATCTTTTCTCGAATGAAATGAACATCAACCTCAATGTGCTTAGTTCGTTCATGGAGTACAGAGTCAGATGCAAGCTTGATAGCTGATTGATTGTCACAAAATAGTTTGGATGGTGACTTCTCTGGGAATCCTAGCTCATTCAAAATAGATCGCAGTCACATAGTCTCACTAGTTCCATTGGCCAAAGCACGATACTCTGATTCGGCTGATGAGCGGGAAACTACATCCTGTGTTTTACTCTTCCAGTAAATTAAGTGATCCCCATAAAAAGTGCAAAAGCCGGAGGTTGACCTTCTATCAGACTGGGTTCCTGCATAATCTGCATTAGTGTAACATGGTAACCCATTTTGAGGACCCGCAGCAAAGAAGAGTCCAAGTCCAGGACTGGATTTGAGGTATCTCAGGATCCGATACACAACTTCCATATGAAGTGTCCGAGGAGCATGCATGAATTGACTCACAACACTAACCGCAAATGACAAATCGGGCCGTGTATTAGTCAAATATATAAGTCTTCTTACTAACCGTTGGTACTGTGATGAGTCTGATAAGGGTTGCCCGGATTCACTCGACAGTTTCTGATTTGGATCCATAGGAGATGAGGCTGGTTTACATCCTATCATGCCAGTGTCATGCAACAAGTCTAGCGTATACTTACGTTGTGACATAGAGATTCCTTTTCGAGAACGGGCCACCTCAATTCCTAAGAAATAACGAAGGTTACCAAGATCCTTGACTTCAAAAACCTTTCCCAACTGCTGCTTGACTTGAGAAATGCCATTAAGATCATCACCAGTAAGAATTATGTCATCAACATAAACAAGAAGGATGCCAAGTTGTCCAGTGGGACTTCGCCTTATGAAGCATGTATGGTCTGAATGGCAGCGTGTGAATCCCAAGGAGAGTACTGCGGTGCTGAATCTGCCAAACCAAGCTCTCGGTGACTGCTTCAAACCATAGAGGGATTTTCTAAGGCGATATACTTTACCATGATACTCCCCCTGAGCCTGAAAACCTGGTGGAGTACACATATAAATGACTCCATCAAGATCACCATTAAGAAAAAGTATTCTTGACATCAAGTTGGTAAAGTGGCCAAGAATGAGATGCAGCAAGTGATACAAGTAACCGGACAGTATTCAGCTTAGCAACTGGAGCAAAAGTGATTGAAAAATCCTTCCCCATAATTTGTGTAAAGCCTTTAGCCACAAGACGAGCCTTATATCTCTCAACAGTACCATCAGCTCGATACTTGACTGTGAACACCCATTTGCAGCCTACTGCCTTCTGTTCTGGAGGTAGAGAAACAAGATCCCAAGTATGATTGTGTTCCAAAGTCTGCATTTCCTCCATCATAGCAGCTGTCCATTTGGGATCCTGTAGGGCTTCAGAAGCAGGCCTCGGAATAGAGATGGAATCAACTGCCCCCAAAAAGGCTCTGTACGAATCTGAAATACCCTGGTAAGAAACATGATTCTCAATAGAATGATTAGTACAAGAAGAGAAACCATATCGAGAAGGAGGTCGATGCTCACGGACAGGGTAGCGGGTAGTATGATCAGAAATGACAGTAGAAGGGTGAACAGTACCTGAGGAGGATTCGGATTCTGGCATAATAGGCTGTGCATCCTGAGTGCGAGGGCGACGAGCATAAAATCTCTCAAAGAAGTAAGAGGGAGGAGCAAATTCTGCCACGGGAAGATCAAGAACAGAAACAGGAAGTGCCGGTGGAGAAGTAGACACAGGAGGTGTCACAAAAACCTATACATGGTTAGTAACAAAATAAGGAGTATCCTCAAAGAAGGTAACATCCATGGAGTAATAAGTATGACGAGTAACTGGATCATAACACCTATATCCTTTGGAAATAGATGAATAACCAAGAAAAATGCAATGAATTGCCTTGTCATCCAATTTTGTGCGAGATGGACTCCTATTCTGGACAAAACAGGTGCACCCAAAAACACGAGGTAAAATGGGAAAAAGGGTAGTATCGAGATGGAGGACATGAAGTGGAGCCTTGCCTGACAAGATCCGCGAAGGAGTACGGTTAACCAGATATGTAGTAGTAAGGACTGCCAAGTGCCAAAAAGATTTGGGAACATTCATACCTCGAAGGATACACCGAACAACACACATAAGTTGACGATTTTTACGTTCGACAACACCATTTTGTTCAGGTGGGTGGGCACATGTGAGTTGCTGTAAAATACCCTTCAGGTTGAGTTGTGAGCAAAAATCATTAGAAATATATTCCCCCCCATTATCAGATCGAATACTATGAACAATGACATCATGTTGGGTGTTTGTCATTTGCAAAAATTGGGTGAATGTGGCAAAGACTTCAGATTTCCTTTTAAAAAGATAAAGCCAAGTGCATCGGGTGTGATCATCAATAAATGTGACATAATATCTATGACCGGACAAAGCAATAGTGGGAGAGGGACCCCAAACATCAGAATGAATAAGATCAAATGGAGCAGGTGCTCGATGCATGCGAGAAATATAACTAGTTCGAACAACGCATTTAAATTTATGAAATTTGCAAGCTTTTATTAAATCTGGAAAAAATAAACACAAATATTGAAAGTTAACATGTCCAACTCAAGCGTGTCACAATTCAAGATTATTTAAAGAAAAAACATAAGAACCCGAAGCGGGCAACACAGAAGCTTGCAAACTGGAACTAGGTGGAGATAAAGGTGAAGGTCCCCAAAGTAGTACAACCCATCGCGCTCATGGCCCATACCAAAAATCCTCTTCGAACTCAGGTCCGGTAAAAAACAAGCAGAAGCCAAAAAGATTACTGCACAATTTAAGGACTTGGCAAGACGACTGACAAAAAGTAAATTAAAAGCAAGATGTGGGACATGGCAAACATTGCGGAGTGTAATATCAGATTGAGACAAACATCACCCTTACTAAGAATAGTAGTAAAGGAACCATCAGCCAAACAGACCGATTGATTAACAGGACTAACATTAGAGGTAAATACTGAAGACTCACCAGCAGTCATATGATCATTTGCCCCGGAGTCCAAGATCCATGAGACGGGACTCCGGTGCGAGAGGAGAGCATGAGGGGTACCTTTAGCTGCCATTGCAGTGGATGAAGAAGAGGCAGAGGTAGTGGGTTGTGGAGAATTTTGCTGCATAAGTGAGCCAAGAGCCTGCTGCATCATTTGTATCTGATTCTGAAGCTGCGAGAGATCAAGAGAGGATGATGTAGGTGGTGCATCAGCTACTGCAACATTGCAGGAGGGAGAAGACGAGGATCCACGATTCACTGGATTGTTATGAGGACGAAGCTCAGGATGGAGTCTGTAACACCTATCAGCTGTATGTCCCTTGCGGTTACTGTGACTGCAATAATGCCTACCAGATGAGGGCTTAAAACGAGCAGCAAATGCCAACTGATCAGACAAGGTTGGTGAAGCAACAGATGGAGCGGTGTTTGGAGAAACATATTCAGTGGGAAGAAGTCTACGTTTCTCATGACAGTCAACCATAGCGTATGCATCAAATAGTGAAGGAAGCAAAAATGTGTTAATAATCATGTTGCAAGTGCTCTCAAATTCCGGTTTCAGTCCCATGAGAAATTGATAAGTGTGGTGTTTCTCCGAACGATCCATGACTAGCTTTGCATGTTTCTCGTCAAAGTCTTCCAGAGTCTCATAATGAGCTAGTTCCTCCCATCGACACTGAAGGTATGCAAAATAGTCCGTCACTGATAATCCTAGCGAAACCTGAGTAGCATGTGAGATTTCCCTGTGCAGCTCATAGATACGAGCATCATTCCTGGAATGAGCATACATTTGTGTCAAACGTGTCCAAAGGCCAGCAACATCTTTATGATACATAAACATAGTGTATAGCCGAGGTTCCATGGCATTTAGGAGCCCACCCAAGATCATACAATTTTCCTGATTCCATCTCTCAAATACTAGTAAGCCACCCTGTCATACTGCGTGTTCCCAAGAAAAGCTTGACAGAATGAGACCAGGAGGCGAAATTCTTCCCATCAAGCAGAGTAGTTGTAATTCTTTGAGTGGGAGCATAGGTGAAAAATAATTGGGGAACACCAATATTATCCTTGGAGGATTCCGACATAGTGAAATATGGAGCTTGGGTCACAAAAAAGGGGATAATAATAGAACTGTGGTGCAGAAATCGGCCTCTACCACAGTATTTTGGACCCAAGTAAATATAAGAGGACTGCTCAGCAATGACACACAGCCCCAAAAGTTATTTTCCAATTTTTTTTTGAATTAATTCATCTGGTTTTGAAAAAAAAAAAAAAAAAAAAAAAAAAAAAGT

>transcript_7 full_length_coverage=2;length=7513

GGTGGAAGTTGCAATCTGCAAAGATGAGGGTATTGAGCAATTTGAAGAACTTGAAATGGGCCCTTTTCTGCGTCATCCGCTTGTTGTGCATTATTTTTCTGTGAATTCTGAAGTCACCTGCGTGTACAAAATAACCAGTGAAGAGATAATTTATTTCCTCATCTTGTTCAAGGATACCCTCAAGAAAAAAAAACACATTGAAGCTGAGGAATTCCTGGATTTTATTGCCATGAAGCGTGAGGTTGTGGGCAGGGAAAAGCTTTGTGTGCGTATTCAAAGCATAGGGATGCATGCCACTTTTATCCGGGAAGCAAGGGCGTCAGAACATTCCACATTAAAGGAATTTCTTAAGAAATGCAGGAAACGTACTTTTGATTCCACAGAGAAAAAGCACGTGGATGATTTTGGTGGCAAGAACACAATTGTCTTCTCATCAATTTCAGAGGATGATAGAGAAGCGCAAAAATCTTTAGATGATGATCATGCTCGCCCTTACCTGAAGGGTGAGGCGGATGCTGACGTTTCTCAAGCTAGCAGCAACTTTTGCGACAATGAGAGTGGCCAACCAGTTGAAAAGATAAAAAAATCAAAAAATTTAAGGTGCGCCACATCTGCACCCGACAAATTGTTTAAAAATGGCAAGGTAAGTCTTGATCATTCGATTGATAACCTCATTGCTGAAAATTATATGAGTATTTTCATCACAATGTTGAAGGATGCATGTCAGGGCCAAACTATAGGCGAGGTTCTTGAGAGGATACTTCAATTTTATGTGTTTTCAGCTAAACGAGTCAGGAGAGCGAAGACGATGTTTTCATCAGCCCCACATGCTGGATTACTAATTATTGCCGTGGCATGTTTCAAATATGGAATATGGGATAGCATATATGCTACTTTTCAAGCCAAGAGTCAACCTTCTGATATTTATTCTAAATTTGAAAGCATAGATGTTAATCCAAGTGGAAAGAATTCACTCACGATTGAAGACCACATTTCTGAGCATATACACGCTTTACCTGTTGAAGATATTATTAAGAAAGTTGCCACTTATTTTGAGCATGATCGTGAAATTCCGAGAAATGGAAAATCTCTAGAGAAGACATTCGACTTTTTAAAGAAGCTGTGCAGTTGCGAGGCTTGGCTGACAGAGCAATTTTCTGTTAAGGAATTCAAGTATCTTGGTAATGGAGAGTTTCTCACATTCTTAGAACGTAATGCCTCTCTACTACCCAATGAGCTACTCAAGTGCTTAACTGGTGAGACATGTGAGAAGTCTCCTTTTGCTATTTGCATGCTCCAACATCAATTGGTTGTGTTACTGTCACAAGCTTCAAATAGCTTATGGGAGAATGAATCAATAACCAAGCAGAAAATTTCTTCACTACTTATGAGGCAATTTCCATTGATCAGTTTTAATATTATTGAAAATGGTTCCTCGAAAGACTTGTTAGATTTCACGACGGAGCAAAAAAGCAGTGTAATTTCGAATTGTGTCCTATTTTCAATTACATTATTTGGGACATGTTATATTGAAGAACCAGCAGCTCAGAATGACAATGATGTGTATGAAATTGCTGCAGTGAGAACTGACACAAGTAATAAAGAAGGAACCCTTGGCCCTGTTACATCCAATGAGGCAGTTGAAGTTCTTCTTCGGGCACCAATGTTGTCAGATCTGAATATATGGTCTCATTGGGACCTGCTTTTTGCTCCATCACTTGGTTCTCTTGTTGGATGGCTGTTGAATGAAGTCAATACGAAAGATCTTATGTGTTTGGTGACAAAAGATGGAAAAGTACTCCGTATTAATCCTTTAGCTACCCCTGGTTCATTCTTGGAAGCTTCACTTCAAGGATCATCTTTCCAAACTGCTGTGGAATTGATCTTTATTTTCATTACTTGGAGGGAAGCGACATGTTCCTTTGGCTCTTCTTAAATGCCACGCACGCCAAGCGTTTGAAGCGATTTTCGAGAATCCCGTGGAAACTATGGAAGTAAATGACAGGCAGAACTTTCTCATGCACGGGATAGCATCATATGGATGGCAAAAACTTCATCGAGATGACACTTCTAATTTGGATAGTGAGTTACAAAAAAGCATAGGAAGAACAAGTAAGGCATTGTCTGTTGCATCTAGGTTTATCATTGATTGTCTTGGTTATCTACCTTCAGAATTCCGTAGCTTTGCTGCAGAAATACTTCTTAATGGTTTTCGTCATGTTAATAAAAAAGCTCCTTCAGCCATTTTGTGTGAATGCACCAAGACAGAACAGCGTGTCATGCTTCATGAAGTAGGGTTGTCTCTTGGTATAGTGGAATGGATTGAAGATTACCATGCATTTTGCTCCACTGCTGCTATTGACTTGTTGAATCCCTCTAGCGCTTCACAGTTGAAAGCCACAAGTTCTGGAATCAGCACAACCTCAAATTATGTGCAAAATGCTTTGGATAATCTTTCTCCTCCTGACGGTGAAATGCAAATCTCTACTTTCGAAGCCTATGACCATAATGGAAAAAGTTCAGATTCAGAAGTTTGCCATCCTATTGACTGCATGGAAGTTGCCGGTAATAGATTCAACAATGATTGCATGCAACATGCATTGCAAATTAAAGAACACGAGGATGCGGTCCTAGTTATTGAATCTATAAGGCAAGAAGAATTTGGTCTTGGTCCTAGTCTTTCATCCGCGGAGAGTTGTATATTGAAAAAGCAGCATGCTCGCTTGGGAAGAGCCCTTCATTGTCTTTCACAAGAACTATATTCTCAGGATTCACATTTTCTTCTTGAACTGGTCCAAAATGCTGATGATAATATCTACCCCGAAAATGTGGAACCGACTCTAACATTCATTCTTCAGAAGGCATGCATTATTCTTTTAAATAATGAACAAGGCTTTTCTGCTCAGAACATCAGAGCCCTTTGTGATGTTGGAAATTCGACAAAAAAAGGATCCAGTTCTGGATATATTGGGAAGAAAGGCATTGGTTTCAAATCTGTATTTCGGGTCACAGATGCACCGGAGATTCATTCTAATGGGTTTCATGTCAAGTTTGATATAAGTGAGGGTCAGATTGGTTTTGTTTTGCCAACAGTTGTGCCTCCTTGCGATATTGATTCATTTTGTAGGCTAGCATCTGGTGACACCGACGAAATTCATGCTACTTGCTGGAACACTTGTATTATGCTTCCTTTTGGATCAAAAGTATCAGAAGGAACTTCAATGAACAACATACTGTCTATGTTTTCAGATCTCCATCCATCTTTATTACTTTTTCTTCATCGCGTCAAATGTATCAAGTTCAGAAATATGCTCAATAATTCCTTCATTGTGATGAAAAAAGAAATTGTCGGCAATGGTATCATAAAGGTTTCCCATGGGAAAGAGGAAATGACTTGGTTAGTAGTATCTCAGAAGTTGCAAGCTGATATCGTCCGCCATGATGTGCAAACAACCGAAATTGCCATAGCTTATACGCTGCAAGAATTAGATAAAGGGGAATACTCTCCCCATCTGGACCGGCAGCCTGTTTTTGCTTTTCTTCCCCTAAGAACATATGGTCTGAAATTTATACTTCAAGGTGATTTTGTTCTTCCGTCATCTAGAGAGGAAGTTGATGGAGACAGTCCCTGGAACCAGTGGTTATTGTCTGAGTTTCCTGGTTTGTTTGTCAGTGCAGAAAGCTCTTTCTGTGAGCTTCCTTGTTTTAGGGAGAATCCAGGAAAAGGTGTTACAGCTTATCTGAGCTTTGTTCCACTTGTTGGGGAAGTCCATGGATTTTTTTCTAGTCTTCCCCGGATGATTATTTCCAAGTTACGCATGTCAAACTGCTTGCTTCTGGAGGGAAACACTAACACGTGGGTTCCTCCGTGTAAGGTGCTAAGAAATTGGAACGAACAGGCTTGCGTTCTTTTACCCAATAGCTTACTTCTTGCGCACCTTGGACTTGGATTCCTGGATAAAGATATAATTATATCTGATCCACTGGCAAAGGCCCTGGGTATTGAGGAGTATGGACCCAAAATCTTACTTCAACTTATTACCTCTTTATCTTGTACAAAAAATGGCCTCAAATCATTGGGTTTGGGTTGGTTGTTTTGTTTCTTAAACGAAGTTTATACTATGTCATTTCATTATTCTGGGAAGAGATCTATAAATCATGGCATGGAATCAGATCTCTTAGATAACCTCAGAAAAATTTCATTTGTTCCACTTTCAGATGGTACTTATGGCTCCTTGGATGATGGCGCAATTTGGTTACATTATGATGCCTTGAGCACTGGGTTTGATGGTGAGCACGAATTGGAAGCGTTTCCAAAATTATATTCTAAACTGCGGACTGTAAGTCCTGCCCTTCTTTCTGCAGCAGCTGTTAAATTCTCGTGCACAGAAATGAGTTTTGTTGACAACGTTGTTAGGATGCTCCATAGAATTGGTGTCCAGCAACTGTCCACGCATGAAATTGTAAAGGTTCACATCTTACCAGCTATAACTGATGATAGAATCATAAATCAAGACAAGAATTTGATGACAGATTACCTCTCCTTTCTAATGCTCCATCTACAATCCAGTTGTCCTAATTGTCGTGTTGAAAGGCAATCCATTATTTCTGAGTTACATAAGAAAATTTTTATTTTAACCAATCATGGCTACAAACGACCATTCGAGGTATCAATTCACTTTGGTAAAGAATATGGGAATCCTATTGATATGAATAAGCTTATTGATGCTTTAGATTTTAATTGGGTTGAGGTTGATAGCAATTATTTGAAGCATCCAATCACCGAGTCACTTTCTGATGGACTGATAAAGTGGAGGACTTTTTTCCTAGAATTAGGCATCACAGATTTTGTACAAACAGTTCAAGTGGAGAAGAGTGTGATTGATATATCTCATATCCTTCTAAAAAATATGATGTGGGACGGGGAACTAATTTCTCGCGGGTCAGTTGCCAAAGATTGGGAATCCAGCGAGTTGGTCCAATTGTTGTCCTTGTTGTCCATGAACGGCAGTGCAGAAATGTGTAGATATCTCTTAGAGATCCTCGATACATTGTGGGATGACTGCTTTAGTGACAAAGTCACAGGTTACTGCAATTCTTTATCCAGTGGAGAAAGCAAACCCTTCAGATCTTCATTTTTAAACAGCATTTGTGATGTTCGGTGGACGGTATCAAGTATGGATGATGAGCTTCACTATCCTAAAGATCTATTCTATGACTGTGAGGCAGTGCGTTCTGTTCTCGGTGCTTCTACGCCATATGCTGTTCCAAAGGTGAAAAGTGGAAAGTTGTTAAGTGATATTGGATTTAAGACTCAAGTTACGCTCGATGGCGTTTTGGCAATTCTTCAAGTGTGGAGAAGATTAGAAATCCCTTTCAGGGCCAGCATAGCACAAATGTCCAGATTTTATACATTCATTTGGCATCACATGGCTACTTCAAAGCAGAAAATTGTGGAAGAGTTACACTCAGGACCTTCCATATTTGTTCCATCAACATCTGGTAGTTCGAACCATGAGGACCTTGTACCCGGTGTATTTTTGTCTCATGAGCAAGTATATTGGCATGATTTATCTAGCTCCATGGACCAAGTAAAGACAACCAATCCTGAGTTGGACTCAACAAGTGACACACATGGTCCATTAAGCAAAACATTGTGCAATGTTTATCCAGGCCTTCGTGACTTTTTTGTGAATGAATGTGGAGTAAACGAGATCCCCCCCTTCCGTAGTTGCCTTGATCATTTGCTGCATTTATCTAGTGTTTCTTCGCCTTCACAATCTGCCAATGCAGTTTTCCAAGTTGTTCTTAAGTGGGCTGATGGACTGAAATGTGGACTTGTGACTTCTCAAGATATTGTTTACTTGAAAGGATGTCTTCTTAAATTGGAATTTACAGTGCTTCCCACTGTACAAGACAAGTGGGTTTCCCTACACCCATCCTTTGGCCTTGTATGTTGGTGTGATGATGAGAAATTGAGTAAAGAATTTAGACATTCAGATAGTATAAATTTTCTATTCTTTGGTGAACTTAGTAACGACGACAAGGAAGCACTCAAAGTGAAGATGCATGCCCTCATGCAAACCCTGGGAATTCCTGCCATTTCAGAGGTCGTAACTCGTGAAGCAATATATTATGGTCCAGCAGATTCTAGTTTCAGAGAATCATTGGTGAACTGGGCTCTTCCTTATGCTCAGCGATACATCTATAATATACATCGCGATAAATATTGTCAACTTAAGCAGTCAGGATTTGAAAATCTGAATCGTCTTCGAGTTGTAGTTGTTGAAAAGCTGTTTTACAAATATGTCATACAGAGAATTAATACTTCATCAAAAAAGCGAATTGAATGTGATAGTGTTCTTCAGGGCAGCATCTTGTACACAACTGAAGAATCAGATTCTCACTCGGTGTTCATGGAGCTTTCTCGTTTATTATATAATGGAAGTCCTGAATTACCCTTGGCAAATTTTCTTCATATGATCACAACCATGGTCGAAGCAGGTTCCACCGAGAACCAACTGGAGTCTTTCATGTTGAATAGCCAAAAGATACAAGAGCTTCCCAATGAAGAATCTATTTGGTCCCTTTCTTTTGAAGACCTTGAAACAAGTGCTCCAGCGATAATCAACGAGCCAACCCCTTCAAAGTTGAAGAAGAAATCAAAATTCAACTCGAGCTGGCCGCCTGTGGATTGGCAGACGGCTCCAGGTTCCACTGTTAAGTCCATAACACCAGGTGAACTTCCACATTTATCCGGAACCTCTCAAAACATCGAAATCCTTCGTATGGGTCCAGTCGTCGAGATCAGTGCTAATCGGACTGTTGACAAGGATTCAGCTATAATAGCAACAGGTCGGCTTGACATTGCCCAATCGGATGACGACCCAGTCGTCGAGATCAGCGCTGATCGAACTGTTGACAAGGATTCAGCGATAATAACAACAGGTCGGCTTGACATTGCCCAACCGGATGAAACACAAGCTTTGGTAACCGGGAGATTGGGAGAACGTGTGGCATGGAAATACTTTGTGGAGAAAGCTGGGGAAAAGGTGGTTGAGTGGGTTAATGAAGATAGCGAAACTGGGTTACCTTATGATATAGTGATAAAGCAAGAGGGAAAGAGCACGGAGTACATAGAAGTTAAAACCACCAAATCGTTGAACAAAGACTGGTTTATTATATCAACGAGAGAATGGGAAGTTGCAGTTGATAAAGGTGAATATTTCAGTATTGCACATGTTTCGTTGGATAATAATAAGGCAAGGATGGTTATATTTAAAAATCTGGCAAGGTTGTGTCAGTTGGGGAAGTTGCAGTTAGCTGTTACCATGCCAAAGCAGCAGGCGGAATTTTCCATGGTCTAATAGATGTTTGATTCTTGTCACAAAACTAAAAACGGTGGCTTTTGTATGTAAATGGTATTGTAAATTAAAATATGTATGTCAACTTTATTATATGGTTATGATAAGTAATTCGAAAAAAAAAAAAAAAAAAAAAAAAAAAAAAAAGT

>transcript_8 full_length_coverage=3;length=7531

GGGGTACATCTTCAGCAGAGGGACCACGATGCAAGAGAGAAGCAGGAACATCAAGACAGATCACTACAAATCGATCTCCTCCGCGGTTCTCTCTAATCATTTACCCCTCCACCTTTTCAGATCTGAGACGATCCCTCCCGCTCCGATCCAAGGTGCCCTTGATTTCTTGCCGAACTTCGCTGGGTACTCATGGGTCGGGTACGCAGCTTCTTCCCTCCTCGTGATTTCTCACTTCCCGTCACCTCTCTCCCCCGAGGAAAGCCTAATTGGCCCCATCTTCCGGCAAGTTCTCGAACTATCCAGCGACGATTCCACCACTATAACTGCCGTTTCCTGGTCTCCGTTTACGCCTTCGACAGGAGATATCGCCGCGGCAGCGGCAAATTGTATTTACGTGCTTTCGCCTGATTCGGGGAGCCTGCACGGTTCTTTTTGTTGGAGCCAGACTGCATTACTTGTACAAACTACTAAGGTGGAGGCGCTCAAATGGACGGGTTCGGGGGATGGCATAGTTGCTGGTGGAATCGAGGTGGTCTTGTGGAAAAATAAGAGCATGTCTTGGGAGATAGCTTGGAAGTTCAAAGCAGAACTGCCTCAGTCTCTTGTTTCCGCGACCTGGTCTATCAAAGGACCTTCGGCAACTGCGGCAGCATGTGGTGATGGATCGTCCCTCATCAACGAGATTGGTAAGAGGGTGTGGGTATGCCAGAATGGTGGGAAATCTGAATACATGAAAGCCGACCTTTGGCATCCTCATCCCGTCTCTGTGATTCAGTGGAGACCACTAGGAAGACAATTAAACAGTAATGCGAAGCATTCACCAAGGCATGTGCTGCTAACTTGCTGCTTAGATGGGACTGTAAGGTTATGGAGCGATATTAATTACGGAAGGGTCAAAAAGATTGGCAAGGACGGTATTGATCACATGACTGCTAGGCCATCTTTTAGTGTTGCAGCTATAATTGAGATAAACCAGACATTGAATGGAACTCTGGGCACGGATGTATTTATGGCATGGGCAACAGAAGTTGGAGGTATAATTAATACCAGTGAAGGAATTGACCAATTTTTTCCCACAGAAGGTCATGAAAGTGATGACCCAGGCAGGTGTGAGTGGTTAGTTGGATTTGGTCCCGGAATGTTGCTTACTTTCTGGGCCATCCACTGCCTTGATGATATCTCTCCAATGAGGTTCCCACGAGTGAGTTTATGGAAGAGACAAGAATTACATGGTCTTGAAATGGGGAATCTACAAAGGAATGGGAATTTTAATTATAAAAACCAGTCTATTCTTAATAAAGTTGTTATTTCAAGGAATCAATTATTTGGTCCTCCAAATACATGTTCTTTAATTCAGTTATTTCCTTGTAATTCCTTGAGTTGGTCACTATTATATAATAAAACTCCGGTTAAGATAGAAGATGAATCTCCGAGTAGATCCAGGACAGAGAACATGTTGTCATGTAATGCAGGTGGATCTTTAAATATAGATGGTCATTTTGGGGAAATTTTACAAGTTGCAGTGCATCCTTTTAACCCTGAAGTTGAACTTGCTGTTTCTCTGGATTCCAATGGTTGTCTTCTATTTTGGTCACTTTCTACCATTTCCAATTGCATTTTGGGCCTGCCGACATTGAATCCTACATGGAAGCTTTGTGGAAAATTAGTATTCCAAGATTCATGTCCTAAATATACAAGCTTGAGGTGGGCTCCTTTGACATTAGATGAAGGGTGGGTTCTTCTTATGGGGCATGTTGGAGGAATTGATTGTTTTATAGTCAATACTTCTCAAAGTGAAGAAGAAAAAATAGTATGCTACAACTTATGCACTATACCATTTACTGGTCATGGTCCTCTTGTGGATAGTCCCACAAATATATTCTCAATTCCTTTGCCAACTGCTTGTTATGAAACCTTTAATACCAAGAGATTTATGCTTTTTGGGGTATGGATGAAGAGCTTTAAAGCTCTATCATGGGAAATCACCTTGCACTCTAATGATTTATTGGGAAGCTGCAGTAAATGTAGTTTTGACACTAGAAATCCTGCTGAATGCGAAGGTTGGAGATTTGAAAATACTTTTGCTGGTAAGAGATACTGTCTTGTTGTTGTTCTTTGTTCATCACATTTGCCAGATCCTTACATCCATGATCAAGTTACAAGTTTTGCGGTAGTCTCCCCAGGTAATTTGATTACCTCTGTACAGGAAAAGTGGGTTTCTGGTAATTCTTTGTGCAGGGCTGTTTCTGCATATCACATGGCTACAGGCTACTCTGATGGTACTGTGAAACTATGGAGAAGTAATCTTGCTAAACCATCAACTTCCCAGTTACTGTGGGAGTTGGTTGGTATGTTTGTTGCACATGAGGACCCAATTACTGCAATATCTTTGACTGATTGTGGTCGAAAGATTGCAACCATAAGCACAGCGGGTCATTCAAAAACAGTTAGCTCTCTCCATATATGGGATTCTGTACATCTTACAGGAACAGGGACTTTTATATTAGAAGATATGTTGTCCTTGGATGGAGATGTCATTGCTTTTAATTGGTTAAGTACTGGAAATGGTCAATTGTTACTTGGAGTTTGCATGGAAAATGAGCTGAAGGTATATGCCCAGAGGCGTTGTGGGGGCCAGACTTTGTTGAACTCTGGTAAATCTTTGGAAATGCACATCTGGTTTTGCATTGCATATGCTCATACTCTCTCCCCTATACATGACTTCTTTTGGGGGCCTGGGGCCTCAGCTGTGGTTGTCCATGGTAAATACTTCTGTCTATTTGCGCAATGGTTGTTCTTTATGGATAAGAAACATCCAGAAGAGACCGAGGATATTCCTCTTAATTGTCATGGTGGTGTCCATGATGACACTCTTCCTGCAACTTTCACCCACGACAACATTTGTAATTATGGAGAATTATCAATGGAAGACAGTATCAAGAATGATCACCAATCTGGCTGTTTGTTTCTAGGAAGTGCTCAAGTGAGACATGGTTCAGGTACCAGGCATGGTTTCTGGAGCATGCTAGAAATAGCTGAGACATTACTGGGCTCCTTGCCTATTTATCATCCGGAGGCACTCCTGATGAATATTTGTTCAGGCAATTGGAAACGTGCATATCTATCTGTTCGGCATCTTGTTGAGTTTCTTACCTCTTTTTATGCATCTGAAAAAAGATTTAGCTCTGCGAAGTCCAGCCATATCGTTCCGCAGATCCACTTCTTAGATTATTTTGAAGGACATCTCTCAGAATGTTCAACAGATAAAGGATTTGAATGGAGTGGAGATGCTACTCTATTCACATTGTCATCTCAATATCAGAGGGGCTTGACCCAATTTGCCTACAATTCAAAATCTGATGCTTCCGATATGTTTATTCCATCTTCGGAAAAATCTGAACTCAGTGGATTTGTTGAGCCACTTGAAAAACTATATGACTTAGCGGCCATAACCAACACAGAGAAGACGCAAATTCTTGCAATTATTGATCTTCTCAGCGAAGTCAGGAGTACACATTCTGTTTCTGCCTATGAAAGTCTTGATACACCTGGGCGGAGATTTTGGGTTGCAGTAAGGTTTCAACAACTATATTTTCATCGAAGGTTTGGTAGATCAGCTTCCACTGAACAGTTGGTTGTTGACTCTGGGCTAATTGGATGGGCCTTCCAGTCTGATTGTCAAGAAATTTTGTTTGGTTCCGTTCTTTCTAATGAATCATCTTGGCAAGAAATGCGAGCCATGGGTGTTGGATTTTGGTTTACTAATACAACACAATTGCGTACAAAGATGGAGAAACTGGCAAGATCACAATATTTAAGGAACAAAGATCCTAAGGCTTGTGCACTTTTATACATAGCATTGAATAGACTTCAAGTTTTGGCTGGCCTCTTTAAAATCAGCAAGGATGAGAAGGACAAGCCTTTGGTGGGATTTCTTTCACGTAATTTCCAGGAAGAGAAGAATAAGGCAGCTGCTTTAAAAAATGCTTACGTTTTGATGGGAAGACATCAGCTGGAGCTAGCTGTTTCTTTTTTTCTGCTTGGAGGTGATGCATCTTCTGCAATTACCGTTTGTGCAAAAAACCTTGGGGATGAGCAGCTTGCACTTGTAATTTGCCGGCTTGTGGAGGGGCATGGTGGACCATTAGAGAGTCAGCTAATATCAAAGTATATACTGCCATCTGCTATTGAGAAGGGAGATTACTGGCTTGCAAGCCTTCTAGAGTGGGAATTAGGGAACTACTATCAATCTTTTTTGATCATGCTTGGCTCCAAAAAGGATTCCATGATCAACAAGTCTGCTCTTTCGTCCAATCATACTTCTTTTTTGGATCCAAGTATTGGACAGTATTGTCTAACACTAACAGCCAAAAATTGCATGAGGAATGCTGTAGGAGAGCAAAATGCTGGAATTCTAGCCAGGTGGGCGAGTTTGATGGCGGCTACTGCCTATAATAGATGTGGCCTTCCTATTGAAGCTTTGGAATGCCTTTCATCTTCTCTGAGCATCCCTGGGGGTAAAGATCAAGTAAGCGTATCGGATATTGGAAATTCTGAAATTCTACCTGGAATACTAAATCCATCCTCAAGTAAATTATCAAATTGGCTGTCAGGTGATGTAGCTATTTGTTTGGAGTCCCATGCTAAATTAGATATAGCAATGAAGTACTTCTCAAAATTGATGATGGAGCACCCAAGCTGGCCATATTCTTATATAGCATGTAGTGGAGCTAATCCTTGCTCCAAAGAATACGAACAGAGTCAGTATGACATACTGCTTAAAAAATTTCGACAGAAGTTACACATAGCGATTGCAAATTTTGAGCTAAAGTTTTCGATAAATCCTGCTTTTCTAATCAACATGATTTTAGTCTTGTGCAATAATGGGTTATTGTTTATTGGATATGATGCATTACATGGGTATGCTTCTCAAAAGGACTTACAAGATATAAGCTATGCAGATGATAGTTTTCTGTTACACCCTATCCTGCCTAGGCTAATTTTAAAGGCTACGAAAGAAATCTCCTGTTTGATGTCACGATTTATTGCCATCTGTAGTATAACTTGTTCTCAACCAAAATCATGTACCGGTGAGAATGACGTGTCTGGTGATATTAGAACAGACTGGTCATATGCTTTGGGGCATTACGTTCAGGCTCTCATACTGTCATTATGGTGTTTAAGACACTCTTTGAAGATATTTTCCAACTCCAAGGATCTTAACAATGAATCCTTTATCGCTCTTGATTTATTTGAATATTGCATATATTTTGCATACACTTGGCTTCAAAGGAATTTCAAAGGTCTTATTATGATGGTGCAACCCCTCATGGTCACATATACCAATGGGCGTACTTATGAAATTGACATTGCAAATTTGAAGGTACTCCTTCACCAGATCGCAGAGTTGGTGAATCATAATGTCGGCGGTGACCTTCAAGTTGCTAGCTGGACACAAGATGGGCAAGGTGGAGATGGAATATCTTCTATACCAGCAGATGAAAGATGGCAAATCATAGGGAGTTGTTTGTGGCATTACGTTTCGAGCTTTGTGAAGCATAATCTGAATTCGATTTCTGATAAACTTGATGGTAGAGCTTCCTTGGCATCTGCTTCTGCATGTTCTGAACCTAAAGAAAATATTATTACCAACCAAATTAGAATGGTCTCAGTGATCTTGACCAAGTTACTTAGGACCACACTTGCACATATTTCTTCTTATCATGCAAAACAACTTGCTTCATTACTTAGGCAGAAAGTGGAGAATGGGTTATGTGTACCAACTCTTGTGTGGTTAGAAAAATCAAATCAGTCTCAGCCTGGATCCCTCAAGCATTTGAGCGTGCTTACTTTGGACATGATGAACAAACAAGATGAATTATGGGTTTTAAATACTTTATGGGATATCTGTGCTAATGCTAAAATAATATCTGAAGTTTTTTCTCAAGAAAATATAAATTGCTCACACTTTATTAGCCAGAAGCCTTCGAAGAGATGGTCTGACATTTATAAAGGCATTTTGGAGGACCATGAAATTGAGGAAACATACAATCAAGAAGGTAAGCTTAGCAGTAACTCTGCCGGTGGAGGACCTGGATTGCCTGTGAGGGCTCTGTTTAGGAGCAGCCACACTTTTCTAGGTTCTCGGCAGAAAGATACAGCCCCTACTAAGGAGGACATACCTTTCCAAAACCCTAAGGAACTTTACAAGAGAAATGGGGAGCTTTTTGAGGCATTGTGTATCAATTCCATTGATCAAGGGCAAGCTGCACTTGCTAGCAACAGGAAGGGGATACATTTCTTTCACTGGGAAGAGGAAGTAGGCTTGAGAGATAAATCAGATTATATATGGTCAGAGGCTGATTGGCCACATAATGGGTGGGCTGGAAGTGAATCAACCCCAGTTCCTACATGTGTTTCACCAGGTGTTGGTCTTGGGATGAAGAAGGGGGCACATCTTGGATTGGGTGGTGCAACGGTTGGGATTCCTGGTTACGGTGGCAGCATGGGTGCCTCTGGCTTAGGTTGGGAGATTCAAGAGGATTTTGAGGAGTTTGTTGATCCACCGGCTACTGTTGACAAGATAAGTACAAGGGCTTTCTCTAGTCACCCCTCCAGGCCTATGTTCTTGGTTGGTTCTAGCAATACGCACATTTACTTGTGGGAGTTTGGTAAGGAGAAAGCTACAGCAACTTATGGGGTCATGGCTGCTGCAAATGTGCCTCCACCATATGCTCTTGCATCAATATCAGCTTTGCAGTTTGATTGCTACGGACACCGATTTGCAACTGCTGCATTAGATGGAACCATATGCACGTGGCAGCTTGAAGTGGGAGGAAGAAGCAACATCCGTCCCACCGAATCATCTCTTTGCTTTAACAGCTACTCATCGGATTTGAGTTATGTTACTTCAAGTGGATCGATTATTGCTGCAGCTGGATATAACACAGATGGTGTTAATGTGGTGATATGGGATACGTTGGCTCCACCTACAACATCACGAGCTTCGATTACGTGTCATGAAGGTGGTGCACGGTCCCTTTCTGTGTTTGATAATGACATAGGAAGTGGTTCTATTTCTCCTTTAATTGTGACGGGTGGGAAAGGTGGTGACGTTGGGCTTCACGACTTCCGTTACATAGCAACGGGAAGGACCAAACGTCATGGGCATTCTGAACAAAATGGGATGCTTTGGTACTTACCAAAGGCTCACTTAGGGAGTGTGACCAAAATAAGTACCATACCAAATACCAGTTTGTTCTTAACGGGAAGTAAAGATGGAGATGTTAAACTTTGGGATGCCAAAAGAGCCAAATTAGTACATCACTGGTCGAAATTGCACGAAAGGCATACATTTCTTCAACCTAGTTCCCGGGGATTTGGTGGAGTTGTTCAGGCGGCTGTTACAGATATACAAGTGATTCCTAATGGATTCCTTACATGTGGTGGAGATGGTTCTGTAAAGTTGCTTCAGCTCAAACATCATTCACATGGATTTTAAAAAAAAAAAAAAAAAAAAAAAAAAAAAAGT

>transcript_9 full_length_coverage=2;length=7475

GGGAAGAACCAACAGAGGCATCATCGTCATCATCAGCAAAGCTAAAGAGTCAAAGCCTTCTCCGCAAGACCCTTCTCCTCGCGATTCGTTCTCTGTTCCAAATCTCTTTTAAGATAAGAGAGTGCTTCCCATGCAACACGTGGCACATTTGTGATTTCACAAGTTGAATTTAGAGCTAAAGACAACTGAAGTTATCTACTCATATTTCTATAGCAACTGACATAAGATTTCTACTTTAGAGTCCCATGGTTCAGCTTAGCTGGCACATCCTCTTATAGTAGTTTGATGCCGGAGGCAGTATGCCGGTTATAGTTGGGAAAACATGCAAAGGTATCACGCTACCAGCTGCACTAGTGCAGTGAATAATACTGCAATTGGTGGGCCATCAGCTAGGGATACTGTCCGTGCCGATTCATCTTCACTCTCAAATAACTTCCCCTCAAATTCAAGGCGGTCTACTCAGCTAACAGCATACAAGTTGAAGTGTGATAAAGAATCTTTGAATTCACGACTTGGGCCACCTGATTATCATCCTCAAACTCCGAATTGTGCAGAAGAAACTCTTACTAGGGAATATGTGCAATCTGGTTATAAGGAGACTGTTGAGGGACTTGAGGAGGCTAAGGAGATCTCACAGACTCAAGTTCAACATTTTAGTAAGCCTGTTGTTGTAAAGTGCAAAGAGGCAATCAGAAAACGTCTGAGGGCCATTAATGAATCTCGTGCTCAAAAGCGGAAGGCGGGTCAAGTTTATGGAGTGCCTCTTTCTGGTTCTCTAGTGACTAAGCCTGGTATCTTTCCAGAACAAAAGCCATGTACTGAAGACTTCCGTAAGAAATGGATCGAGGCCCTATCCCAGCACCATAAACGACTGCGGTCTTTGGCCGATCAAATTCCTCATGGTTACAGAAAGAAATCCCTTTTTGAAGTCTTAATCAGGAATAATGTTCCGTTATTAAGAGCATCTTGGTTTATAAAAGTAACTTACCTTAATCAGGTTCGGCCTGGCTCTGCCAGCATATCTTCTGGGGCACCTGACAAAACTCAGCTGTCTCGCACAGAACTCTGGACAAAAGACATTATTGATTACTTGCAGTACCTCCTAGATGAGCTTTTTTCAAGAAATAGTCACCCTGCTCCACACACCAGGGACCGGTCTCCACAAATGCTTTATGCTGGGTCAATACAGCACAAGAATGATCAAGCATCAACAGTTGTTGATGGTGAGGAGCCTTCCCTACATTTTAAATGGTGGTATGTGGTGCGGATCTTGCAATGGCACCATTCAGAAGGATTGCTTCTCCCTTCTCCCATTATCGATTGGGTTCTTAATCAGCTACAGGAGAAGGAGTTGTTCGAGATTTTGCAGTTGTTATTACCTATTATATATGGCGTCATAGAAACTGTTGTTCAGTCCCAGAAGTATGTACGTACTCTTGTGGGTGTAGCTGTCCGTTTCATCCGCGAACCTTCTCCAGGTGGTTCTGACCTAGTAGATAATTCCCGGAGAGAATATACTACTTCTGCTCTGGTTGAGATGCTTCGGTATTTGATTCTTGCTGTACCTGATACATTTGTTGCTTTGGATTGCTTTCCATTACCACCCTGCGTGGTGTCACAGACAGTAAATGATGGGAGTTTTTTATCAAAGATATCTAGGGATGCGGGAAAGATAAAAAATGATCCATCTGAAGTTCCTTGTGTTTTCAGAGATAAAAGGCTTGATACCCAGTATCACTCGTCCTTAAATCATGTCATTTCATCTATTCAAACTCGTGCAGATAATCTTGCAAAGGCTGCAAGCCCTGGCTATCCAGGTCACAGTGTGGCCAAAGCTGTACAGGCCTTGGACAAAGCTCTTGTACAGGGGGATGTGAGAGTGGCATATAAGTTTTTGTTTGAAGATATTTGTGATGGGGCCTCTGATGAAGGTTGGATTGCAGAAGTCAGCCCATGCTTGAGGTCATCATTGAAGTGGATTGGGACTGTAAACTTGTCATTTGTTTGCTCTGTGTTTTTCCTCTGTGAATGGGCGACCTGTGATTTCCGGGATTTTCGAACTGCACGCACTCATGATTTGAACTTCACTGGAAGGAAAGATTATTCTCAAGTGTACATTGCAATTCAGCTTTTGAAGCTAAATAAAAGACATATGCAAAGTCCTGTTCGAAGCAAAAATGACAGCGCCCTTGGGGTAAAAAACCATCCGAAAGGTGCCGGTCAGCAGAATAATGGTTATAACAGGACCTCCGTGGGAAATGTTCAGGAAATAAAATATAGAGGAAGCGTTGACTCATCAGATATTTTTCAAAGCCCAGGTCCTTTACATGATATTTTAGTGTGTTGGATTGATCAACATGAAACAAATAAAGGAGAAGGTTTCAAGCGAGTTCAACTTTTACTTATGGAACTCACACGATCTGGCTTATTTTACCCACAGGCGTATGTGAGGCAGCTGATAGTTAGTGGATATATGGATAGGAATGGACCTCTAGTTGACCTGGACAGACGGAAGAGACATTATCGAATCTTGAAACAGCTGCCAGCACCATTTTTGCGTGATGCTTTGGAGGAAGCACGGATTGCTGAAATGCCACGGCTTTTGGAGGCAATGCATGTTTACTCGAATGAACGCCGGCTTGTGCTTCGTGGGCTTCTATCTGATCAATGTAAAAATGCAATTAGTGCAAATGTACCATCCAAGAAACAAAAGCTCAATACGGTCTTTGTATGGGATGGTGCTTCTCCAACTCTTCAGTCGACATCCATTTCTTTGCATGGTAAAAATGTCAAGAGTGCAACTGGCATGGATGAATTGAAGACTGCCATAGCAGTATTGTTACAACTCCCAAATTCCTCTTCTGCATCTGTGGATACTGGACTTGATGAATCTCAGGGGAGTGTTAAGAGGCCTGTTGGGTTAATTTTCAACAAGATGGATTTGGTGGAAGGAACGCCTGGGTGTGAGGAATGTAGAAAGGTTAAGAGACAAAAGTTAAGTGAGGAAAGGAGTCCATACCTTCAAGCACATTCACCAAATCCCTTTGATGATGACGATACTTGGTGGGTAAGGAAGGGGTCTAAATCCTTAGAGTCTGTTAAAGTTGATCCACCACTTAAGTCAACCAAGCAGGCATCGAGGGGCAGGCAAAAAGTTGTGCGTAAAACTCAAAGTCTTGCTCAATTAGCAGCTGCTAGGATTGAGGGAAGCCAGGGAGCATCTACAAGTCATGCATGTGATAACAGGATAAACTGCCCTCATCACAGAACTGGTATGGAAGGAGAAACTCCCAAGTCAATGGATGGGGCAAGAACAACACATTATGGAGATATTGTTTCAATTGGAAAAGCTTTAAAGGAGCTGCGTTTTGTAGAGAAGCGGACCATTACAGTTTGGTTGATGAATACAGTTAGACAGTTTGTTGAGGAGACTGAAAAAACTGCTGCTAAGGTAGCCCAAATCGGTAGGCCATTCCCTGTGGATGAGAGGAGCTCTATACGGTGGAAGCTTGGTGAAGATGAGTTATCTTCCATTCTGTATTTGATGGATGTTTCTAATGATTTAGTTTCAGCAGCTCGATTTCTCCTTTGGTTGTTGCCAAAGGCTCTCACTAACCCAAATTCTACAATTCACGGTGGGAGAAGTGTTCTTATGCTGCCGAAGAATGTGGAAGGCCATGGATGTGAAGTGGGGGAGGCGTTTCTGTTATCATCTATCCGAAGGTATGAGAACATACTTGTTGCAACTGATCTTGTGCCTGAAGCCTTGTCGGCCACAATGCACCGTGCTTCAGGAGTTATTTCCTCGAATGGAAGGGTTTTGGGTTCAGGGGTCCTAGTTTTTACTCGTTACTTGTTGAAGAAATATGGCAATGTGGTTAGTGTCATTGACTGGGAAAAGAACTTCAGGGCAACATGTGACAAGAGACTTCTTTCTGAATTTGAATCTGGACGATCGTTAGATGGAGAGTTTGCATTTCCGCTTGGAGTTCCCGCAGGAGTTGAAGATCTTGATGACTTTCTTCGGCTAAAGATAAGTGGCGGACGGATATCCCGAGTTGGTGCGAACATGAATAGCGTAGTGCAAAAACACATGGATGAAGTTTTTCTCTCTTTCTTAGGCAAAGACAGGAAGCTTTATGCCACTGGTACTCCAAAAGAAAAATGGGATGATGGATATCAAGTAGCTCAACAAATTGTTATGGGACTAATGGAGTGCATGAGGCAGACTGGCGGTGCCGCTCAAGAAGGAGACCCTTCCTTAGTGTCCTCTGCTGTTTCTGCGATTGTTTCTTATATCGGACCGGGAATAGCAAAAATTTCAGACTTAACTGCAGGCAGTAATTATTCAAGTTTTCCGTCTGCTACCGGTTCATTAAATTTTGCTCGCCGCATTTTGCGCATTCATATTACTTGCCTATCCCTACTTAAGGAATATCTTGGTGAGCGCCAAAGTCGTGTATTTGATATAGCTCTTGCACAAGAGGCTTCTTCTGCTCTTGCTGGAGTTTTTGCTCCTGGAAAGGCTTCTCGTGCACAATTTCAGCCATCTCCTGAACCCCATGATTCCAGTTCAAGTATGGCAAGTGAACTCTTGAATAATTCATCCAAGGTAGTTCTTAGCAGGGCTACAAAAATTACAGCTGCTGTATCTGCACTTGTCATTGGGGCAGTTGTTCATGGAGTTACTAACCTGGAGAGATTGACGACTGTATTCAGGTTAAAGGAAGGGTTAGATGTACTTCAATTTGTAAGGAGTACAAAATCCAATTCAAATGGGAATGCTCGTTCAATTGGGGCTTTTAAAGTGGATAACTTAATTGAAGTGTATGTGCACTGGTTTAGACTACTTGTTGGGAACTGCAGAACCCTCTGTGATGGATTAATTGTGGAACTCCTTGGCGAATCATCCGTTGTTGCTCTTTCTAGGATGCAGCGAACACTACCTGTTAACTTGGTCTTTCCACCTGCCTATTCAATATTTGCATTTGTTATATGGCGACCGTTCATTCTTAACAGCAATATTGCTACTCGTGATGACATCCACCAATTATATCAATATTTAACATTGGCCATAGGGGATGCCATAAAACACCTTCCTTTTCGTGATGCATGCCTGAGAAATACTCGTGGTTTCTATGAACTTGTAGCTGCAGATTCTAGTGATGCTGAGTTTGCTTCCATGCTAGAGTTGAGTGGTCCAGACAGGCATTTGAAAACCATGGCTTTTGTTCCTCTCCGTGCTAGACTTTTCCTTGATGCGCTCATTGACTGTAAAATGCCACACACTGATTTTACACAGGATGAGGGGCATCGGGTCTCTGCACCTGAAAAGGAGATGAAACTTCTGGATAAGATTGTACATGTTTTGGACACCTTACAACCTGCAAAATTTCATTGGCAGTGGATCGAGCTTAGACTTCTGTTGAATGAACAAGCCCTTGTTGAGAAACTTGATACCCATGATATATCCTTGGCAGATGCTATACGCTCCCTCTCACATAATTCTGAAAAAAATGCTGCTTCTGAAAACGAGAACAATTTCATTGAGATCATCCTCACAAGGTTACTGGTCAGACCTGACGCTGCTCCTCTCTTTTCAGAACTGGTGCATCTTTTTGGGAGGTCACTTGAGGATTCCATGTTGTTGCAAGCTAAATGGTTTCTTGGAGGCCCTGATGTTCTTTTTGGACGCAAGTCCATTAGGCAGCGGCTTATCAATATTGCCGAGAGTAAGGGTCTTTCAACCAAGGCCCAGTTTTGGAAGCCATGGGGGTGGGTTAAATCTAGTTCCGAATTTGCACCAAACAGGGGAGAAAAGAGGAAGTTCGAAGTCGCTTCTCTTGAAGAAGGTGAAGTTGTTGAAGAAGGCATGGATGTAAAAAAAATCGGAAAAGGCTCCACTCAAATGTTTGACATTGAAGGTTTCATTATCAGTCAGCAGCACGTAACTGAAAGAGCTCTCATAGAATTAGTTCTTCCTTGCATCGATCAAAGCTCTGATGACTCGCGCAACATATTTGCAAGTGATTTAATAAAGCAGATGAATAATATTGAGCAACAAATAAATGCAGTTACTCGTGGAACAAATAAGCTGGCAGGCACAGTTGCTTCTGGAATTGATGGTCCTGTAAATAAAGGCAATAACCGGAAAGGTATTCGAGGTGGCAGTCCTGGATTGGCTAGACGATCAACAGTAATAGTTGATTCCACTGCACCTCCATCATCTGCTGCATTGCGAGCATCAATGTCACTGCGGCTTCATTTCATCTTAAGATTGCTTCCTATCATTTGTGCAGATGGGGAGCCATCTGGCCGGAACATGAGACATATGCTTGCCTCGGTAATACTTCGCCTTCTTGGAAGCAGAGTTGTGCATGAGGATGCAGACCTACCCTTCTATCCTACACACAGTTCTATATCAAAAAGAGAGGTGGAGACAATGATGGAAGCATCTCCTGTTGCTGCTTCGGTTTATTTATCTAGTGAAAGTTTATTTGATCGGTTATTGTTGGTACTTCATGGGTTGTTGAGTAGTTCCAAGCCAAGCTGGCTGAAGTCGAAACCAGCTTCGAAGTCTAGCATCGAATCCACTAAAGACTTCTCTCCGTTTGATCGTGAAGTCGCAGAAAGTTTACAGAATGATTTGGATCGAATGCAACTTCCTGACGCGATACGGTGGCGCATCCAAGCTGCAATGCCGATACTTTTCCCATCCATCAAGTGCTCCATCTCCTGCCAACCACCATCTGTATCCTCAACTACCCTTATTGCTTCACTTCAGCCCATCATTTCAGTTCCTGGATTTCAAAATCCAAACCCACCACCACCACAAAGGAATTCGGTACGAAACTCTGCCAACACACAAACCAAGTCTTCGAAATCATTACCTTTACAACAAGATCATGATACCGATATTGATCCATGGACCCTCTTGGAAGACGGTGCGGGTTCCGGCCCATCTTCGAGTAACACAGTTGTGATTGGTACCAGTGACCTTGCTAACCTTCGAGCGTCCAGCTGTCTTAAAGGAGCCATACGAGTGAGACGAACCGACCTCACATATATTGGTGCTGTTGATGATGATAGCTGATTTATGTTTTTTTGTCCATTCTTGAGTTAAAGAGAAAATCCCCCACCATCCTGCTTTCTTTTGCATTGGAGGAACATTTTTTTGGGCAGCGCGGGTTCTAACGAAGATGTTACTCATTGCCGCCCCATCAAAACGGGTCTCGGGGTTGCCAGGCCCTCACTAACCTTTTGACAACTACCACCATCGCCCTTTGCCCCCCGTTACTGGGCTAGGTGAGATTAGAATGCTTTGTTGTATATATTTAGGTTATTTAACTTGCCAATTTGAGCCGAGTAAAAAATGTCATGAATGATGTGTAGTTGTTTCATGCTCATTGATCTCTTCGTTGGAAGTCTTTTTTACACAAAAAAAAAAAAAAAAAAAAAAAAAAAAAAAAAAAAGT

>transcript_10 full_length_coverage=2;length=7442

GGGGTCTCCGCCTATCGATTGAATTGCACAGAGCACTCTTCCCCTGCTTTGCTTTCTTTCCCTTCGTCTTCCTTCCCCTGCTTCTATAAATTTGTTTGCTTTTGTGTTCGTTTCGTTTCGTTTCCCCTGTTGTAAATTTGAGTCTCGAAGAATCCGCTGGGATTTTTCACCGGCGATAAGGATTTTCTTCGTTGATTCTCAAACCAAACCTCGTGATTTTCAAAGAATTTTCTCACAATTTCCCTGTCAATTTCTGACTTTCCATCTGTGGAAGGAAATTTTTAGTCAAATAGATCTGTCGTTAGACGCCCGGTTGTGGATTAAGGATATCTCTAATCTCTATCGTTAGCTATCACTAATGTCGGCTTCTTCAGCTTCTGTCTTTAATCTCCAAAAGAACCCGTGTGTTGTTCCTTCGCTTAACCGTCCGTCTTTTAACCATCAACTCAATGTTTGTTCTGTAAGGCGCCATGGAAACAGAACAAACAGAGCCTCCTGTTACGCTAGCCGATGGAATGTTTTGGAGAATAGGTTTTTTTGGAACGAGATTGCGGGAGTCTGGATCAGAAAGACTCCATCTTTGGCAATCGGACGTTTCCAGAAAGTCCTTAAAGCAGAGGGTCATGGTGCGATCCGCATTGTCCTTGGTTCCGGAGAAGCCCCTCGGTCTTTACGATCCTTCCATGGATAAGGATTCTTGTGGGGTTGGGTTCGTTGCGGAATTATCAGGCGAAGGCAGCCGGAAAACGGTGACTGATGCTTTGGAGATGTTGGTACGCATGTCACATAGAGGTGCTTGTGGCTGTGAAACAAATACCGGTGATGGAGCGGGTATTCTCGTGGCTCTTCCTCACGATTTCTTCAAGGTTGATATTGAGTTTGAGCTACCACCTCCAGGGGAGTATGCTGTCGGCATGTTCTTCTTGCCCACATCCGAGAGCCGAAGAGAACAGAGCAAAATTGTTTTTACTAAGGTTGCCGAGTCTCTTGGGCATACAGTTCTTGGGTGGCGGTCTGTCCCAACTGATAACTCAGGATTGGGCAAGTCTGCTTTGCAGACAGAACCCGTGATTGAACAAGTGTTCCTTACTTCCACTCCTAGGTCAAAAGCTGACTTGGAGCAACAGATGTACATTTTAAGAAGGGTTTCTATGGTTGCCATCCGAGCTGCTCTAAACCTTCAACATGGTGGTGTTAAGGACTTCTATATATGTTCTCTTTCCTCGAGGACTGTGGTTTTACAAAAGGTCAGCTAAAGCCCGACCAGTTGAAGCAATACTATTATGCAGATCTTGGCAATGAAAGGTTTACGAGCTACATGGCCCTGGTACACTCTCGGTTCTCCACGAACACATTTCCTAGCTGGGATCGAGCTCAACCAATGCGTGTCTTGGGACATAATGGAGAAATTAACACACTCAGGGGCAATGTAAACTGGATGAAGGCTCGTGAGGGTCTATTGAAGTGCAAGGAACTTGGTCTTTCAAAAAATGAGATGAAGAAGCTTCTGCCTATTGTGGATGCCAGCTCATCTGATTCCGGAGCTTTTGATGGCGTTCTTGAGCTTTTGATTCGAGCTGGCAGAAGCCTCCCCGAGGCTATTATGATGATGATTCCTGAAGCATGGCAAAATGACAAGAACATTCATCCTGAACGGAAAGCCTTGTATGAATACTTCTCAGCTCTTATGGAGCCATGGGATGGACCGGCTCTTATATCATTTACTGATGGTCGCTATCTTGGAGCAACATTGGATCGAAATGGTTTACGCCCAGGTCGTTTTTACGTCACACATAGTGGTCGAGTAATAATGGCTAGTGAGGTTGGTGTGGTAGACATTGAACCCGAAGATGTGTGTAGGAAAGGAAGACTTAACCCAGGCATGATGCTTCTAGTGGATTTTGATAAACATATTGTTGTAGATGATGAGGCCTTGAAACAACAATATTCACTAGCAAGGCCTTATGGGGAGTGGCTTAAGAGGCAGAAAATAGAACTTAAGGACATTGTTGAATCTGTTCATGAATCTGATCGGATTTCTCCAACTATTGCTGGAGTGGTGCAGGCATCTAGCAATGACGATGACATGGAAAATATGGGAATTCATGGGTTATTGGCTCCATTAAAGGCGTTTGGTTACACAGTCGAAGCCTTGGAGATGTTGTTACTTCCTATGGCAAAAGATGGTAGTGAGGCCCTAGGTTCAATGGGAAATGATGCTCCTCTAGCGGTAATGTCTAACAGAGAGAAGCTGACGTTTGAATATTTCAAACAAATGTTCGCACAAGTCACAAACCCCCCTATTGATCCAATCCGGGAGAAGATTGTTACTTCCATGGAATGCATGATTGGCCCAGAAGGTGATCTCACAGAAACTACTGAAGAACAATGTCGTCGTCTTTCATTAAAAGGACCTCTTTTATCCGTTGAAGAAATGGAAGCAATTAAAAAGATGAATTACAGGGGTTGGAGAAGCAAAGTTCTTGATATCACTTATTCCAAAGACAGTGGTAAGAAAGGATTAGAGGAGACCCTAAATAGGATCTGTTATGAAGCGCGGGAGGCAATTAAAGAAGGTTACACATTACTGGTGCTTTCTGACAGAGCCTTTTCTTCAAAGCGTGTTGCTGTGAGCTCCCTTTTGGCTGTTGGGGCTGTTCATCATCATTTGGTTAAAAAGCTAGAGCGCACTCGAATTGGTTTAATAGTTGAATCTGCTGAACCACGTGAAGTTCATCATTTCTGTACACTGGTTGGATTTGGTGCAGATGCTATATGCCCATATTTGGCTATAGAAGCGATTTGGAGGCTACAGGTTGATGGAAAGATTCCACCTAAAGCAAGTGGTGAGTTTTATTTGAAGGATGAGTTGGTTAAGAAGTACTTTAAAGCAAGCAACTATGGAATGATGAAGGTTCTTGCTAAAATGGGGATTTCAACTTTGGCCTCTTACAAGGGTGCTCAGATTTTTGAAGCTCTGGGTCTTTCTTCAGAAGTGGTTGAAAGATGTTTTGCTGGAACTCCAAGTCGAGTTGAGGGTGCAACATTTGAAATGCTTGCTCGTGACGCACTTCATTTGCATGAAATGGCATTTCCCAAACGTTCTTTGCCTATTGGAAGTGCTGAAGCTGTAGCGTTACCCAATCCCGGGGACTATCACTGGAGGAAAGGTGGCGAGATTCACCTGAATGATCCTCTTGCCATAGCCTCTCTTCAAGAGGCTGCTAGAACTAATAGTGTTTCTGCCTATAAAGAATACTCCAAGCGCGTTCAGGAACTGAATAAAAGCTGCAATTTGCGTGGGCTTCTGAAGTTCAAAGAGTCTGCAGTGAAGGTTCCCTTGGATGAGGTGGAATCAGCCAGTGAGATTGTGAAAAGGTTTTGTACTGGAGCCATGAGTTATGGTTCGATATCATTGGAGGCGCACACCACTCTGGCTATTGCTATGAATAAAATTGGAGGGAAGTCAAACACAGGTGAGGGAGGTGAGAATCCGTCCAGATTGGTGCCCCTTCCAGATGGTTCAATGAATCCGAAGAGGAGTGCAATTAAGCAGGTTGCGAGTGGAAGATTTGGAGTTTCAAGCTATTACCTTACAAATGCCGATGAACTGCAGATAAAAATGGCCCAGATGATGCCGTGTCTGCAAAGCACCTCTTGGTGTCTTTGCATCTATAACTGATTCCACATTTGGCTTTTGGGGGCCAAGCCTGGTGAAGGAGGTGAACTTCCTGGCCACAAGGTTATTGGAGATATTGCTGTCACTAGGAATTCCACTGCTGGTGTTGGACTAATCAGCCCACCTCCTCATCACGATATCTATTCCATTGAAGACCTTGCCCAATTAATTCATGATCTTAAGAACTCCAATCCAGGGGCTCGAATCAGTGTAAAGTTGGTATCAGAAGCTGGTGTTGGAGTAATTGCTAGTGGAGTTGTCAAGGGGCATGCTGATCATGTCTTGATCTCAGGTCATGATGGAGGTACAGGGGCTTCTCGATGGACTGGTATTAAGAGTGCTGGGCTTCCCTGGGAACTTGGTCTTGCTGAGACGCATCAAACTCTTGTTGCTAATGACCTTCGTGGTCGAACAACTCTCCAAACAGATGGCCAACTGAAAACAGGAAGAGATGTGGCCATTGCTGCACTTCTCGGTGCGGAGGAGTTTGGCTTCAGCACAGCACCCCTCATTACACTTGGCTGCATAATGATGAGGAAGTGCCACAAAAACACCTGCCCGGTTGGCATTGCAACCCAAGATCCAGTTCTCGAGAGAAGTTTGCTGGAGAACCCGAGCATGTCATAAACTTTTTCTTTATGCTAGCAGAAGAGCTGAGGGAGATTATGTCTCAACTTGGATTCCGCACAATCACTGAGATGATTGGCCGTTCTGATATGCTGGAGGTTGATAAAGAAGTGACAAAGAACAATGAGAAGCTAGAGAATATTGATCTCTCCCTATTACTTAGACCTGCTGCTGACATTCGGCCTGAAGCTGCTCAATATTGTGTCCAGAAACAAGATCATGGCTTGGACATGGCTTTAGACCAAAAACTTATAGCTCTTTCCAGTGCTGCTTTAGAAAAAGGTCTTCCTGTATACATTGAAACACCAATATGCAATGTGAACCGTGCAGTTGGGACAATGCTTAGCCATGAAGTGACTAAACGCTATGAGTTGGCAGGGCTTCCTGCAGACACCATCCATATCAAACTCAATGGAAGTGCAGGCCAGAGTCTTGGGGCTTTTCTCTGCCCTGGCATCATGCTGGAACTCGAAGGTGATAGCAATGACTATGTTGGTAAAGGACTATCAGGTGGCAAAATAGTAGTTTATCCTCCAAGAGCAAGCCAGTTTGATCCAAAGGAAAACATTGTTATAGGTAATGTGGCTCTGTATGGGGCAACTAGTGGTGAAGCATATTTTAATGGGATGGCAGCAGAAAGATTTTGTGTTCGTAATTCAGGGGTGAGAGCAGTTGTAGAAGGTATAGGTGACCATGGGTGCGAATACATGACAGGAGGGACAGTGATTGTTCTTGGAAAAACAGGAAGGAATTTTGCTGCAGGTATGAGTGGTGGTGTTGCTTATGTTCTTGACGTGGATGGGAAGTTCCGATCTCGATGCAATACCGAGCTGGTAGATCTTGATAAAGTTGAAGATGAAGAGGATATTCTGACTCTCAGGATGATGATACAACAACATCAGCGTCACACAGGCAGCCAGCTAGCCAGAGAAGTGCTCGCAGACTTTGAAAATCTTCTGCCAAAATTTATCAAGGTCTTCCCAGGGATTATAAACGTGTTCTTGCAAGCATGAAGTCTGAGAAAGCCTCTGAAGAAGTGTCTGAGAAGGCTGCTAAAGAAACTGAGGATCAAGATGAGGCAGAGTTAAAGGAGAAAGATGCTTTTGAAGAACTTAAGAAGCTCGCAGCTGCATCAGTGAATGAGAAACCCAGTCAGAAGGTAGAGGAACCTAAGAGGCCTAGTCGTGTTAAGGATGCTGTCAAACATCGAGGTTTTGTTGCCTATGAGCGTGAGGGTGTTTCCTACAGAGATCCTCATATTCGGATGAACGACTGGGATGAGGTTATGGAACAGTCTAAACCTCCCCTGCTGTTGAAGACCCAATCAGCCCGTTGCATGGATTGTGGCACTCCCTTCTGTCATCAGGAGAAGTCTGGTTGTCCTCTTGGGAACAAGATACCTGAATTCAATGAGTTGGTGTATCAAAATAGATGGCGTGAAGCATTAGACCGGCTCCTAGAGACAAATAATTTCCCAGAGTTCACTGGTCGAGTGTGCCCTGCACCATGTGAAGGCTCTTGTGTTCTTGGTATTATTGAGAATCCCGTGTCCATCAAAAGCATAGAGTGTGCCATTATAGACAAGGCTTTTGAGGAGGGTTGGATGGTGCCACGGCCTCCACTTGAGAGAACAGGGAAAAGAGTTGCTATTGTTGGAAGTGGACCATCTGGCTTGGCTGCTGCTGATCAGCTGAACAAAATGGGCCATTTTGTTACTGTCTTTGAGCGTGCCGATAGAATTGGAGGGCTTATGATGTATGGAGTTCCCAATATGAAGGCTGGCAAGTTGGATATTGTTCAACGACGGGTTAACCTTATGGCCGAGGAAGGCGTCAATTTTGTGGTTAATGCTAATATTGGAGTAGACCCCTTATACTCTGTTGATCGGCTTAGGGAGGAGAATGATGCCATTGTTTTGGCAGTAGGAGCAACAAAACCAAGGGACCTTCCCGTTCCAGGACGGGAATTCTCAGGAATCCACTTTGCCATGGAGTTCCTTCATGCAAACACCAAGAGCTTGCTTGACAGCAATCTCGAAGATGGTAACTATATTTCTGCGAAGGGCAAGAAGGTAGTGGTCATTGGTGGAGGTGACACTGGTACAGATTGCATAGGGACATCAATTCGTCATGGCTGCAGTAGCATCATAAATCTAGAGCTTCTCCCTGTGCCACCACAAAGCAGGGCTCCTGGAAACCCTTGGCCCCAGTGGCCTCGCATATTCCGTGTAGATTATGGACACCAAGAAGCTGCTACAAAGTTTGGTAAAGACCCGAGATCTTATGAGGTACTGACCAAGAAGTTCGTTGGAGATGAAAATGGGGTTGTGAAGGGACTTGTAGTGGTAAAAGTTCAGTGGGAGAAGGATGCAAGTGGGAGGTTTCAGTTCAAGGAAGTAGAAGGCTCCGAGGAAATGATTGAAGCGGACCTTGTCCTGCTAGCCATGGGTTTCCTTGGACCCGAGTCGAACGTAGCTGAGAAGCTGGGCTTGGAGTGTGACAATCGGGTCGAACTTCAAAGCAGAGTATGGCCGCTTTGCAACCAACGTTGATGGGATCTTTGCTGCTGGGGATTGCCGGCGGGGCCAGTCTCTGGTTGTATGGGCTATCTCAGAAGGTCGACAAGCTGCTTCACAAGTAGACAAGTATCTTGTAAGAGAAGAAGAGGACCTCACTATTAGTGAAGAGGACTTCAAGAAGCAGCAGCAAGGCAGCAGCAAATACACATTAATGACGTAGATGATCTCTCTTTTTTCCTTATTTTTTCTTTTACATAGTGCTGAAGAAAACAAAGACCAATACCATCAACAAAAGTGGTTCACAATCCCGAGCACAGCTGTTGAATTATTTGTGTATGACAATTCATGGGTTCGGAGAGCTCTTCTGGATTTCTACCGAGGGGTTGAGAAGAAGCAGAGCTAAGAGTGGGTTTCTTGTTGGTTGAGGTGAAGTGTGTGAGTAGGTTTAATTTTCTAAATATATTTGTTTATAAGTTTCACACCCTTGTTTGAGGTGTTAAGCTCCTGTGGTGGTTAAGAATAATGTATAGTTTTGTTTCCCAATCTTATGATTGATAGTAATATATGGGGAATTTGTGGTAGTACCAAAAAAAAAAAAAAAAAAAAAAAAAAAAAAGT

>transcript_11 full_length_coverage=4;length=7380

AAAATAGAAAATAAAAAGCAAAACACAAAAAACAAAAACAAATCCTAAAAATACACACCCGTATTAGGGTTTGACTCAAAAAATGCAAATCGCGTGAACCCCCCCAGTCTGAATCACCCGATTCTATCGGCAATTTCCTCCGGCGAAGCGCGCAGTAACCGATCTCTCATTGGTCGAGATTTTTTCGCGAAAGAACCTTCTTCTTTGGTTTTAGTGAGAGAAACAGAAAGTAGAAAGAGAGAGAAAACATATATAGATATACATACATATATAAGAGAGAGAGGGGAGGCGGGGTTTTTGTAACGTTGGGATCGGAGGGAAAAAGGGGTTTTGAGGGATTTTTTATTTGATTTTAATTTTGTGTCCTGGATTGGATATAAATTTTTTTGCATGCAGTCTGGTGGTGGGACCGGTGGCAGGGCGCCGGTGGGGCCACCGGGACGGGCAACTTCGACATCGTCGGCGTCGCCTTCTTCTTCTTCGTCCGCTGTGTCGACGCCGCACTTAGGGTTTGATTCGATACAGCAGCAACAGCAGCAGCAACAACTGCAGCAGCAGCAGCAGAGACAGACATTCCAACAACAATTACTTAGAAAATCTGAAGGAAATGGAGCTCTTTTAGGGTATCAAGCTGGTGGCCTCCATGGAGTTATGGGAGGGGCAAATTTTGCCTCATCTTCTGGTTCTATGCAACTGCCCCAACAACCAAGGAAGCTAAATGACGCTGCTCAACAGCATGGTTCTCCCCATGTTCGAGAGGAGGGCCAGAATAAAGGTCAAGGCATTGAGCAACAAATGCTAAATCCTATTCATCATGCTTATCTCCAATATGCTTTCCAGGCCCAGCAGAAGTCAGCATTGGGTATGCAATCACAGCAGCAAGCTAAAATGGCTATGGTTGGGACTCCATCTGGGAAGGACCAAGAGACTTGGATGGGACATATGAAAATGCAGGAACTCATGTCTTTTCAGGCTGCTAACCAGGCTCAGGCATCATCATCATCTAAGAAGCCATTTGAACATTTTGCTCGTGGAGAGAGGCATATGGAGCAAGGACAGCAGCCTGGTACTGATCAGAGGGGTGAACCAAAGTCTTCCACCCAGCCGACAACAATTGGACAAATAGTGCCTCCAAATATTGTAAGGCCTATGCAGGTGCCACAAACTCAGCAGGGCATTCCAAACATGGCAAATAATCAGCTAGCGATGGACGCCCAGTTGAAACAATTGCATGCTTGGGCACTTGAACGTAAGATTGATCTGTCATTACCCGCAAATGCCAATTTGATGGCACAGCTCATTCCACTGATGCAATCAAGAATTGTTCACCACCAAAAACCTAATGAAAGCAATATGGTTTCACAGGCATCTCCTGCTCCAATGTTAAAGCAGCAGGTTACTTCTCCACCAGTTGCAAGTGAGAATTCTCCCCATGGTAATTCGTCAAGTGATGTGTCTGGACAGTCTGGCTCTGCAAAAGTTCGACAGACGGTTCCTCCCTCTCCCTTTGCCTCAACTTCAAATTCTGCAGTAATCAACAACACTAACAACATGGCAATGCAGCAGTTTGCTACTCACAGCCGAGAGAACCAGGTGCCTCATAGACAGTCGTCAATGTCTGGAAATGGAATTACTGCCATGCATCCCCCACAAACATCTATGAACACGAACCAGGGTGTGGATCAATCTTTACAAGCAAAGAATACAGTATATGGCCCAGAGGCTCTGCAAATGCAGTACCTTAGACAGTTGAACCGATCTTCGCCACAACCTGCGGCCAATGACGGGGCCTTGGGAAACCAAGTACCATCACAGGGTGGACCAGTGAATCAGGTTTCACAACAACGTTCTGGATTCACCAAGCAGCAACTGCATGTTCTCAAAGCTCAAATACTTGCATTTAGGCGTCTCAAGAAAGGAGATGGTACTCTCCCGCAAGAACTTCTTCAAGCTATTGCACCACCTCCACTAGAATTGCCGCCGCAACAGGTATTTCTTCCTGTAGGAACAAGTAGTCAGGATAGATCGGCTAGTAAGATTGTAGAAGACCAGGCAAGGCATATGGAGTCCAATGAGAAGGATACACAGGTTGTGACGCCAACTATTGGACGGAATAATTTTAAAGAAGAAGTCTTTGTAGGGGATGAGAAAGCAACACCATCAACATTGCATATGCATGGTATGCCGATTGTGGTGAAGGAACCTATACCAGTGGTATCTGCTGGAAAGGAAGAGCAGCAAAATAATGTATTTGCTATTAAATCAGAGCCGGATGTTGAACGTGGTAGTCAGAAAACTCCTGTTAGAAGTGATTTTACAGTTGATAAAGGGAAGGCAGTTGCGCCACAGGTTGCTGTATCTGATGCCGGACAAGTTAAGAAACCTGTTCAAGCTGGTAGTACACCGCAGCCGAAAGATGTTGTCTCTACCAGAAAGTATCATGGACCACTATTTGATTTCCCCTTTTTCACCAGGAAACATGACTCCTTTGGGTCAGCAATAATGGTTAACAACACTAACAATCTAATGTTGGCATATGATGTTAAAGATCTTCTTTTTGAGGAAGGAATGGAAGTGCTTAGCAAGAAAAGGACAGAAAATTTAAAGAAGATTGGTGGTTTACTGGCTGTAAATTTGGAGAGAAAAAGGATTAGGCCAGATCTTGTTTTGCGGTTGCAAATTGAAGAAAAGAAGCTGAAACTTCTGGATCTTCAGGCACGTTTAAGGGAGGAAGTCGATCAACAGCAACAGGAGATAATGGCAATGCCTGACAGGCCATACCGTAAGTTTGTTCGGCTATGTGAGCGCCAACGGATGGAACTTTCCAGGCAAGTTCAGGTCTCTCAGAAAGCTACAAGAGAGAAGCAGTTGAAATCCATTTTTCAGTGGCGTAAGAAGCTTCTTGAGGCTCACTGGGCCATCCGCGATGCACGGACTGCCCGCAACAGGGGAGTTGCAAAATATCATGAGAGAATGTTGAGAGAATTTTCTAAGCGAAAGGATGATCATCGGAATAGAAGAATGGAAGCATTGAAGAATAATGATGTCGAAAGGTACAGGGAAATGTTGATGGAGCAGCAAACTAGCATTCCAGGTGATGCTGCTGAACGATATGCTGTTCTTTCGTCATTTTTAACTCAGACAGAAGAATATCTTCATAGACTGGGGAGTAAGATAACAGCTGCCAAGAATCAGCAGGAGGTGGAAGAAGCTGCAAATGTTGCTGCTGTTGCTGCACGATCACATGGTCTGTCTGAAGAAGAAGTTAGGGCAGCAGCAGCTTGTGCCAGAGAGGAAGTAATGATAAGGAATCGCTTCTCTGAAATGAATGCACCTAAGGATAATTCATCTGTCAACAAGTATTATAATCTTGCACATGCTGTGAATGAAAGGGTCACCCGGCAGCCCTCAATTTTACGTGCTGGAACCTTACGAGATTATCAGCTTGTTGGGTTGCAGTGGATGCTCTCTTTGTATAACAACAAATTAAATGGAATATTGGCAGATGAGATGGGTCTTGGGAAGACTGTACAGGTAATGGCATTGATAGCTTATCTGATGGAGTATAAAGGGAACTATGGTCCACATCTTATTATTGTTCCAAATGCTGTTCTAGTAAACTGGAAGAGTGAGCTCCATACTTGGCTGCCATCTGTGTCCTGCATTTTTTATGTTGGGTCAAAGGAACAGCGGTCAAAATTATTTTCTCACGAGGTTTGTGCTCTGAAATTTAATGTCCTCGTGACAACTTACGAGTTCATCATGTATGATCGTTCCAAACTTTCTAAAGTTGATTGGAAGTATATTGTAATTGATGAAGCACAACGAATGAAAGATCGGGAATCAGTCTTAGCTCGGGATCTTGATAGATATCGCTGTCAGAGGCGTTTACTTCTCACAGGCACTCCGTTACAGAATGATCTGAAAGAGCTTTGGTCACTTTTGAATCTTCTTCTTCCCGAAGTGTTTGATAACCGGAAAGCTTTTCATGACTGGTTCTCAAAACCTTTTCAAAAGGAAGGTCCTACACATAATGCGGAGGATGACTGGCTTGAGACTGAAAAGAAGGTCATTATCATCCATCGACTTCATCAAATTTTGGAGCCTTTCATGCTCAGGCGTCGCGTTGAAGACGTGGAAGGTTCACTTCCACCAAAGGTCTCTATTGTTTTAAGATGCAGAATGTCATCTATTCAGAGTGCTATTTATGATTGGATCAAATCCACTGGCACTCTTCGAGTTGATCCTGAAGATGAACTGCGCAAGGTTCAAAAAAGTTCAATCTACACTGCTAAAATATACAGAACTTTAAATAACAGATGTATGGAGCTCAGGAAAGCTTGCAATCATCCTTTGCTTAATTATCCATATTTCAATGACTTTTCTAAAGATTTCCTTGTGAGATCATGTGGGAAATTGTGGATTTTGGATAGGATCCTCATTAAACTTCAGAGGACTGGGCACCGAGTACTACTTTTTAGTACTATGACAAAGCTCCTTGATATATTGGAGGAATATCTGCAATGGCGGCGACTTGTCTACAGACGAATTGATGGAACGACTAGCTTAGAAGACCGTGAATCAGCTATAGTGGACTTTAATGGTCTTGATTCTGACTGCTTTATCTTCTTGCTCAGCATTCGGGCTGCTGGACGAGGTTTGAATCTGCAGTCGGCTGACACAGTTGTGATATATGATCCCGATCCGAATCCTAAAAACGAGGAACAGGCAGTTGCTAGAGCCCATCGAATTGGACAGAAGAGAGAAGTGAAAGTCATTTATATGGAAGCAGTCGTGGACAAAATCTCTAGCCATCTGAAAGAGGATGAATTCTGCAGTGGTGGTACCGTTGATTTAGAGGATGACCTTGTGGGTAAGGACCGGTATATGGGATCTATTGAAAGCCTCATACGGAATAACATTCAGCAATATAAGATTGACATGGCAGACGAGGTTATCAATGCTGGGCGTTTTGACCAGAGAACAACACATGAAGAAAGGCGGCTGACTCTAGAAACATTGTTACATGACGAGGAACGATATCAAGAAACTCTCCATGATGTTCCCTCACTGCAAGAGGTAAATCGCATGATTGCTAGGAGTGAAGAAGAGGTTGAACTGTTTGATCAGATGGATGAAGAACTTGATTGGACTGAAGAGATGACAAGCTACGATGAGGTTCCTAAGTGGCTTCGAGCCAGTTCTAGAGAAGTCAATTCTACCATTGCAAATTTATCAAAGAAACCATTAAAAAAAAGTTCATTAGCTGGAAATATTGCCGTGCAACCCAGTGAAATAGTTCCCGATTTATCTCCAAAAACCGATAGAAGAAGGGGGCGGCCTAAGGGGAAAAAGTTCCCCAATTACTCAGAGTTGGATGATGAAAATGGAGAGTATTCTGAAGCAAGTTCTGGAGAGAGGAATGAGTACTCAGTGCATGAAGAAGAAGGCGACATTGGAGAATTTGAAGATGAAGAATTTAGTGGTGCTGTTGGGGCACCACCAATTCAAAAAGACCAATCAGAAGAAGACGGTCCAGTTTGTGATGGTGGGTATGATTATCCTCAAGTCATGGAAAACAGTAGAAACAATAACATAGTTGAAGAAGCCGGCTCCTCAGGATCATCATCAGAGAGTCGAAGATTGATTCGGACAGCGTCACCTTCCATATCTTCTCAGAAATTTGGGTCTTTGTCTGCATTAGACGCCAGGCCAAGTTCTATTTCAAAGAGGCCTTCAGATGAACTAGAGGAAGGGGAAATTGCTGTATCTGGAGATTCTCACATGGACCGCCAACAGTCAGGTAGTTGGATTCATGACCGTGACGAAGGTGAAGATGAACAAGTTTTGCAACCAAAAATGAAACAAATAAAACGGAAACGTAGTATGCGGGTTCGGCCCCGTCATATTGTGGAAAGGCCAGAAGAGAAGTTTAGCAATGGGAAGTCATCTCTACAACGTAGAGATCCATCTCCTTTGCCATTCCAAGTGGACCATAAAAATGAAGTGCAGATAAAGTCTGAACCAAAAATGTTTGGCGAAACCAATGCTTTCAAGCATGATCAAAATGATTCATCCTTGAAAAGGCGTAACTTGCCATCAAGGAGGGTTGCAAATACGTCAAAGTCGCATCTTTCCCCAAAATCTAGCAGATCGAATGGAATTTCCGCTCCTGCAGAAGATGCTGTTGAACATTCTCGAGAAAGTTGGGATGGTAAAATTACGAACACTAGTGGGACTTCACATTTTGGCACTAGAATGTCTGACATCATCCAAAGAAGGTGCAAAAACGTGATCAGCAAGCTTCAACGAAGGATAAACAAAGAAGGTCATCAAATTGTTCCGTTGCTAATCGATCTATGGAAGAGAATTGAAATTCATGGTTATAACACGGGCGGATCCGGAAATAATCTATTTGATTTAAAGAGAGTTGATCAACGAATTGACAGATTCGAGTATAACAGCGTGATGGAACTTGTGGTTGATGTGCAGACTATGTTAAAAAGTGCAGTCCATTATTATGGTTGTTCGCTTGAGGTGAGAACTGAAGCGAGGAAAGTACACGATTTGTTTTTCGATATCCTAAGGATTGCATTTCCCGACACAGATTTTCGAGAAGCTCGAAATGCAGTTTCTTTCTCTGGCTTGGGAGCTGCTTCTGCTTCGGCTCCAGCTCTGGTTCCAAGACAAGGCACAGTTGGCCAAAACAAGAGACACAAGCTGATAAACGAGGTGGAACCTGAACCTGGCCCTAGTCTTCCAAAACCATTACCACGTGCATCTTTACTTGACGACTCAAGGGCCAGAAGCCATATCATCCCCCAGAAGGAATCAAGACAGGACGAGTCTCTTTCACATTCTCCATTACTTACTCATCCAGGAGAGTTGGTTATTTGCAAGAAGAAGAGGAAAGATAGGGAAAAATCAGTAGTAAAGACCAGGACAGGGTCAAGTGGACCAGTTGGACCAGTTTCGTCACCTCCCAGTATAAATCGGAGTATTAGAAGTCCCGGACCGGGTTCAATTCCAAAGGAATCGAGGTTAAATCAGCAGAGTAATCAGCAAGGGTGGGCTAACCCCAACCAGGCTGCTGGTCAACAGGTAAATGGTGGTGGGATTGTGGGTTGGGCAAATCCGGTGAAAAGGACCAGAACTGATGCTGGGAAAAGGAGGCCTAGTCATTTATGAATAATAATTTTTTTTAGAAAAGGTTTGTGTGATTGGATGGAAAGATCTGTTGGTATTGATGTCGGGTGGGACCGGCGGTGACTGTATTAGTCGATCGAAGAGGCTTGTACGCATTGTACCCAATATTGTTAATATAGTATAAGCATGTTCTTCCATTGTAAAAAAAAAAAAAAAAAAAAAAAAAAAAAAAGT

>transcript_12 full_length_coverage=17;length=7367

GGGGTCTCCGCCTATCGATTGAATTGCACAGAGCACTCTTCCCCTGCTTTGCTTTCTTTCCCTTCGTCTTCCTTCCCCTGCTTCTATAAATTTGTTTGCTTTTGTGTTCGTTTCGTTTCGTTTCCCCTGTTGTAAATTTGAGTCTCGAAGAATCCGCTGGGATTTTTCACCGGCGATAAGGATTTTCTTCGTTGATTCTCAAACCAAACCTCGTGATTTTCAAAGAATTTTCTCACAATTTCCCTGTCAATTTTCTGACTTTCCATCTGTGGAAGGAAATTTTTAGTCAAATAGATCTGTCGTTAGACGCCCGGTTGTGGATTAAGGATATCTCTAATCTCTATCGTTAGCTATCACTAATGTCGGCTTCTTCAGCTTCTGTCTTTAATCTCCAAAAGAACCCGTGTGTTTTTCCTTCGCTTAACCGTCCGTCTTTTAACCATCAACTCAATGTTTGTTCTGTAAGGCGCCATGGAAACAGAACAAACAGAGCCTCCTGTTACGCTAGCCGATGGAATGTTTTGGAGAATAGGTTTTTTGGAACGAGATTGCGGGAGTCTGGATCAGAAAGACTCCATCTTTGGCAATCGGACGTTTCCAGAAAGTCCTTAAAGCAGAGGGTCATGGTGCGATCCGCATTGTCCTTGGTTCCGGAGAAGCCCCTCGGTCTTTACGATCCTTCCATGGATAAGGATTCTTGTGGGGTTGGGTTCGTTGCGGAATTATCAGGCGAAGGCAGCCGGAAAACGGTGACTGATGCTTTGGAGATGTTGGTACGCATGTCACATAGAGGTGCTTGTGGCTGTGAAACAAATACCGGTGATGGAGCGGGTATTCTCGTGGCTCTTCCTCACGACTTCTTCAAGGTTGATATTGAGTTTGAGCTACCACCTCCAGGGGAGTATGCTGTCGGCATGTTCTTCTTGCCCACATCCGAGAGCCGAAGAGAACAGAGCAAAATTGTTTTTACTAAGGTTGCCGAGTCTCTTGGGCATACAGTTCTTGGGTGGCGGTCTGTCCCAACTGATAACTCAGGATTGGGCAAGTCTGCTTTGCAGACAGAACCCGTGATTGAACAAGTGTTCCTTACTTCCACTCCTAGGTCAAAAGCTGACTTGGAGCAACAGATGTACATTTTAAGAAGGGTTTCTATGGTTGCCATCCGAGCTGCTCTAAACCTTCAACATGGTGGTGTTAAGGACTTCTATATATGTTCTCTTTCCTCGAGGACTGTGGTTTACAAAGGTCAGCTAAAGCCCGACCAGTTGAAGCAATACTATTATGCAGATCTTGGCAATGAAAGGTTTACGAGCTACATGGCCCTGGTACACTCTCGGTTCTCCACGAACACATTTCCTAGCTGGGATCGAGCTCAACCAATGCGTGTCTTGGGACATAATGGAGAAATTAACACACTCAGGGGCAATGTAAACTGGATGAAGGCTCGTGAGGGTCTATTGAAGTGCAAGGAACTTGGTCTTTCAAAAAATGAGATGAAGAAGCTTCTGCCTATTGTGGATGCCAGCTCATCTGATTCCGGAGCTTTTGATGGCGTTCTTGAGCTTTTGATTCGAGCTGGCAGAAGCCTCCCCGAGGCTATTATGATGATGATTCCTGAAGCATGGCAAAATGACAAGAACATTCATCCTGAACGGAAAGCCTTGTATGAATACTTCTCAGCTCTTATGGAGCCATGGGATGGACCGGCTCTTATATCATTTACTGATGGTCGCTATCTTGGAGCAACATTGGATCGAAATGGTTTACGCCCAGGTCGTTTTTACGTCACACATAGTGGTCGAGTAATAATGGCTAGTGAGGTTGGTGTGGTAGACATTGAACCCGAAGATGTGTGTAGGAAAGGAAGACTTAACCCAGGCATGATGCTTCTAGTGGATTTTGATAAACATATTGTTGTAGATGATGAGGCCTTGAAACAACAATATTCACTAGCAAGGCCTTATGGGGAGTGGCTTAAGAGGCAGAAAATAGAACTTAAGGACATTGTTGAATCTGTTCATGAATCTGATCGGATTTCTCCAACTATTGCTGGAGTGGTGCAGGCATCTAGCAATGACGATGACATGGAAAATATGGGAATTCATGGGTTATTGGCTCCATTAAAGGCGTTTGGTTACACAGTCGAAGCCTTGGAGATGTTGTTACTTCCTATGGCAAAAGATGGTAGTGAGGCCCTAGGTTCAATGGGAAATGATGCTCCTCTAGCGGTAATGTCTAACAGAGAGAAGCTGACGTTTGAATATTTCAAACAAATGTTCGCACAAGTCACAAACCCCCCTATTGATCCAATCCGGGAGAAGATTGTTACTTCCATGGAATGCATGATTGGCCCAGAAGGTGATCTCACAGAAACTACTGAAGAACAATGTCGTCGTCTTTCATTAAAAGGACCTCTTTTATCCGTTGAAGAAATGGAAGCAATTAAAAAGATGAATTACAGGGGTTGGAGAAGCAAAGTTCTTGATATCACTTATTCCAAAGACAGTGGTAAGAAAGGATTAGAGGAGACCCTAAATAGGATCTGTTATGAAGCGCGGGAGGCAATTAAAGAAGGTTACACATTACTGGTGCTTTCTGACAGAGCCTTTTCTTCAAAGCGTGTTGCTGTGAGCTCCCTTTTGGCTGTTGGGGCTGTTCATCATCATTTGGTTAAAAAGCTAGAGCGCACTCGAATTGGTTTAATAGTTGAATCTGCTGAACCACGTGAAGTTCATCATTTCTGTACACTGGTTGGATTTGGTGCAGATGCTATATGCCCATATTTGGCTATAGAAGCGATTTGGAGGCTACAGGTTGATGGAAAGATTCCACCTAAAGCAAGTGGTGAGTTTTATTTGAAGGATGAGTTGGTTAAGAAGTACTTTAAAGCAAGCAACTATGGAATGATGAAGGTTCTTGCTAAAATGGGGATTTCAACTTTGGCCTCTTACAAGGGTGCTCAGATTTTTGAAGCTCTGGGTCTTTCTTCAGAAGTGGTTGAAAGATGTTTTGCTGGAACTCCAAGTCGAGTTGAGGGTGCAACATTTGAAATGCTTGCTCGTGATGCACTTCATTTGCATGAAATGGCATTTCCCAAACGTTCTTTGCCTATTGGAAGTGCTGAAGCTGTAGCGTTACCCAATCCCGGGGACTATCACTGGAGGAAAGGTGGCGAGATTCACCTGAATGATCCTCTTGCCATAGCCTCTCTTCAAGAGGCTGCTAGAACTAATAGTGTTTCTGCCTATAAAGAATACTCCAAGCGCGTTCAGGAACTGAATAAAAGCTGCAATTTGCGTGGGCTTCTGAAGTTCAAAGAGTCTGCAGTGAAGGTTCCCTTGGATGAGGTGGAATCAGCCAGTGAGATTGTGAAAAGGTTTTGTACTGGAGCCATGAGTTATGGTTCGATATCATTGGAGGCGCACACCACTCTGGCTATTGCTATGAATAAAATTGGAGGGAAGTCAAACACAGGTGAGGGAGGTGAGAATCCGTCCAGATTGGTGCCCCTTCCAGATGGTTCAATGAATCCGAAGAGGAGTGCAATTAAGCAGGTTGCGAGTGGAAGATTTGGAGTTTCAAGCTATTACCTTACAAATGCCGATGAACTGCAGATAAAAATGGCCCAGGGGGCCAAGCCTGGTGAAGGAGGTGAACTTCCTGGCCACAAGGTTATTGGAGATATTGCTGTCACTAGGAATTCCACTGCTGGTGTTGGACTAATCAGCCCACCTCCTCATCACGATATCTATTCCATTGAAGACCTTGCCCAATTAATTCATGATCTTAAGAACTCCAATCCAGGGGCTCGAATCAGTGTAAAGTTGGTATCAGAAGCTGGTGTTGGAGTAATTGCTAGTGGAGTTGTCAAGGGGCATGCTGATCATGTCTTGATCTCAGGTCATGATGGAGGTACAGGGGCTTCTCGATGGACTGGTATTAAGAGTGCTGGGCTTCCCTGGGAACTTGGTCTTGCTGAGACGCATCAAACTCTTGTTGCTAATGACCTTCGTGGTCGAACAACTCTCCAAACAGATGGCCAACTGAAAACAGGAAGAGATGTGGCCATTGCTGCACTTCTCGGTGCGGAGGAGTTTGGCTTCAGCACAGCACCCCTCATTACACTTGGCTGCATAATGATGAGGAAGTGCCACAAAAACACCTGCCCGGTTGGCATTGCAACCCAAGATCCAGTTCTCCGAGAGAAGTTTGCTGGAGAACCCGAGCATGTCATAAACTTTTTCTTTATGCTAGCAGAAGAGCTGAGGGAGATTATGTCTCAACTTGGATTCCGCACAATCACTGAGATGATTGGCCGTTCTGATATGCTGGAGGTTGATAAAGAAGTGACAAAGAACAATGAGAAGCTAGAGAATATTGATCTCTCCCTATTACTTAGACCTGCTGCTGACATTCGGCCTGAAGCTGCTCAATATTGTGTCCAGAAACAAGATCATGGCTTGGACATGGCTTTAGACCAAAAACTTATAGCTCTTTCCAGTGCTGCTTTAGAAAAAGGTCTTCCTGTATACATTGAAACACCAATATGCAATGTGAACCGTGCAGTTGGGACAATGCTTAGCCATGAAGTGACTAAACGCTATGAGTTGGCAGGGCTTCCTGCAGACACCATCCATATCAAACTCAATGGAAGTGCAGGCCAGAGTCTTGGGGCTTTTCTCTGCCCTGGCATCATGCTGGAACTCGAAGGTGATAGCAATGACTATGTTGGTAAAGGACTATCAGGTGGCAAAATAGTAGTTTATCCTCCAAGAGCAAGCCAGTTTGATCCAAAGGAAAACATTGTTATAGGTAATGTGGCTCTGTATGGGGCAACTAGTGGTGAAGCATATTTTAATGGGATGGCAGCAGAAAGATTTTGTGTTCGTAATTCAGGGGTGAGAGCAGTTGTAGAAGGTATAGGTGACCATGGGTGCGAATACATGACAGGAGGGACAGTGATTGTTCTTGGAAAAACAGGAAGGAATTTTGCTGCAGGTATGAGTGGTGGTGTTGCTTATGTTCTTGACGTGGATGGGAAGTTCCAATCTCGATGCAATACCGAGCTGGTAGATCTTGATAAAGTTGAAGATGAAGAGGATATTCTGACTCTCAGGATGATGATACAACAACATCAGCGTCACACAGGCAGCCAGCTAGCCAGAGAAGTGCTCGCAGACTTTGAAAATCTTCTGCCAAAATTTATCAAGGTCTTCCCCAGGGATTATAAACGTGTTCTTGCAAGCATGAAGTCTGAGAAAGCCTCTGAAGAAGTGTCTGAGAAGGCTGCTAAAGAAACTGAGGATCAAGATGAGGCAGAGTTAAAGGAGAAAGATGCTTTTGAAGAACTTAAGAAGCTCGCAGCTGCATCAGTGAATGAGAAACCCAGTCAGAAGGTAGAGGAACCTAAGAGGCCTAGTCGTGTTAAGGATGCTGTCAAACATCGAGGTTTTGTTGCCTATGAGCGTGAGGGTGTTTCCTACAGAGATCCTCATATTCGGATGAACGACTGGGATGAGGTTATGGAACAGTCTAAACCTCCCCTGCTGTTGAAGACCCAATCAGCCCGTTGCATGGATTGTGGCACTCCCTTCTGTCATCAGGAGAAGTCTGGTTGTCCTCTTGGGAACAAGATACCTGAATTCAATGAGTTGGTGTATCAAAATAGATGGCGTGAAGCATTAGACCGGCTCCTAGAGACAAATAATTTCCCAGAGTTCACTGGTCGAGTGTGCCCTGCACCATGTGAAGGCTCTTGTGTTCTTGGTATTATTGAGAATCCCGTGTCCATCAAAAGCATAGAGTGTGCCATTATAGACAAGGCTTTTGAGGAGGGTTGGATGGTGCCACGGCCTCCACTTGAGAGAACAGGGAAAAGAGTTGCTATTGTTGGAAGTGGACCATCTGGCTTGGCTGCTGCTGATCAGCTGAACAAAATGGGCCATTTTGTTACTGTCTTTGAGCGTGCCGATAGAATTGGAGGGCTTATGATGTATGGAGTTCCCAATATGAAGGCTGGCAAGTTGGATATTGTTCAACGACGGGTTAACCTTATGGCCGAGGAAGGCGTCAATTTTGTGGTTAATGCTAATATTGGAGTAGACCCCTTATACTCTGTTGATCGGCTTAGGGAGGAGAATGATGCCATTGTTTTGGCAGTAGGAGCAACAAAACCAAGGGACCTTCCCGTTCCAGGACGGGAATTCTCAGGAATCCACTTTGCCATGGAGTTCCTTCATGCAAACACCAAGAGCTTGCTTGACAGCAATCTCGAAGATGGTAACTATATTTCTGCGAAGGGCAAGAAGGTAGTGGTCATTGGTGGAGGTGACACTGGTACAGATTGCATAGGGACATCAATTCGTCATGGCTGCAGTAGCATCATAAATCTAGAGCTTCTCCCTGTGCCACCACAAAGCAGGGCTCCTGGAAACCCTTGGCCCCAGTGGCCTCGCATATTCCGTGTAGATTATGGACACCAAGAAGCTGCTACAAAGTTTGGTAAAGACCCGAGATCTTATGAGGTACTGACCAAGAAGTTCGTTGGAGAAGAAAATGGGGTTGTGAAGGGACTTGTAGTGGTAAAAGTTCAGTGGGAGAAGGATGCAAGTGGGAGGTTTCAGTTCAAGGAAGTAGAAGGCTCCGAGGAAATGATTGAAGCGGACCTTGTCCTGCTAGCCATGGGTTTCCTTGGACCCGAGTCGAACGTAGCTGAGAAGCTGGGCTTGGAGTGTGACAATCGGTCGAACTTCAAAGCAGAGTATGGCCGCTTTGCAACCAACGTTGATGGGATCTTTGCTGCTGGGGATTGCCGGCGGGGCCAGTCTCTGGTTGTATGGGCTATCTCAGAAGGTCGACAAGCTGCTTCACAAGTAGACAAGTATCTTGTAAGAGAAGAAGAGGACCTCACTATTAGTGAAGAGGACTTCAAGAAGCAGCAGCAAGGCAGCAGCAAATACACATTAATGACGTAGATGATCTCTCTTTTTTCCTTATTTTTTCTTTTACATAGTGCTGAAGAAAACAAAGACCAATACCATCAACAAAAGTGGTTCACAATCCCGAGCACAGCTGTTGAATTATTTGTGTATGACAATTCATGGGTTCGGAGAGCTCTTCTGGATTTCTACCGAGGGGTTGAGAAGAAGCAGAGCTAAGAGTGGGTTTCTTGTTGGTTGAGGTGAAGTGTGTGAGTAGGTTTAATTTTCTAAATATATTTGTTTATAAGTTTCACACCCTTGTTTGAGGTGTTAAGCTCCTGTGGTGGTTAAGAATAATGTATAGTTTTGTTTCCCAATCTTATGATTGATAGTAATATATGGGGAATTTGTGGTAGTAAAAAAAAAAAAAAAAAAAAAAAAAAAAAA

>transcript_13 full_length_coverage=2;length=7314

GGGGAGGAACCCTAAAAACTGAACAGAGAGAACACGCAAACTCTCAAATATCTTTCGATTTGCCATTTTCATGGTAGTTTAGGGTTTGTTAGATAAATCAAACATGTGGAATGGTCAGCTTGCGCCTCCGGGCACCGGTGGCTCGTCAATTCCGCCACCTCCAGCGGCACAACCGTCGTACACGGTGTTGCCTTCTCCGGCGGAGGCCGAGGCTCGGCTCGAAGAGAAGGCACGAAAATGGCAGCAGCTTAATACAAAGAGGTACAGCGATAAGAGGAAGTTCGGGTTTGTCGAGGCGCAGAAGGAGAATATGCCTCAAGAGCACGTTAGGAAAATTATAAGAGACCATGGGGACATGTCGTCCAAAAATATCGTCATGATAAACGTGTGTATCTTGGAGCCCTTAAATTTATTCCACATGCAGTCTATAAGCTTCTTGAGAACATGCCAATGCCGTGGGAACAAGTTCGCGAAGTGACAGTGTTGTACCATACTACTGGAGCGATCACATTTGTTAATGAAATTCCATGGGTTGTAGAACCTATCTATTTAGCTCAATGGGGCACAATGTGGATCATGATGCGAAGAGAGAAGAGAGATCGTCGGCATTTCAAGAGAATGCGGTTTCCGCCATTTGATGATGAGGAACCTCCGTTGGACTATGCTGATAATCTGTTAGATGTGGATCCTCTGGAGCCAATTCAACTGGAGTTGGATGAAGAAGAAGATTCTGCTGTATACACTTGGTTTTATGACCATAAGCCTTTAGTGAAAACTAAACTTATCAATGGTCCAAGTTATCGGAAATGGCATCTCTCGCTCCCAATCATGGCGACTCTTCATCGGCTTGCAGGACAGCTGCTTTCTGATTTAATTGACCGCAATTATTTTTACTTGTTTGATATGGAGTCATTCTTTACTGCCAAGGCGTTGAACATGTGCATACCTGGTGGCCCCAAGTTTGAGCCCTTGTATCGTGACATGGAGAAAGGGGATGAGGACTGGAATGAATTCAATGACATCAATAAACTTATAATCCGGTCACCTCTTAGGACCGAGTATAGAATTGCTTTCCCCCATCTTTATAACAATCGGCCCAGGAAAGTAAAGTTAAGTATATATCATACTCCCATGATCATGTACATAAAGACCGAGGATCCCGATTTACCGGCATTTTATTACGATCCCTTGATACACCCTATATCTAACAAGGAGAGAAAGAAAGTTCATGAAGAAGATGATGACGAGGAGTTCGTGTTGGCTGAAGGGGTTGAACCCTTGCTGCAAGACACTCAACTTTATACTGACACTACGGCTGCAGGTATTTCGCTGCTGTTTGCTCCACGCCCTTTCAACATGAGGTCTGGTAGGATGAGACGGGCTGAGGATATACCACTTGTTTCAGAGTGGTACAAGGAGCACTGTCCTCCATCATACCCGGTTAAAGTCAGGGTCAGCTACCAGAAGCTATTAAAATGTTTTGTGTTGAATGAGCTGCATCACAGACCACCCAAGGCTCAGAAGAAGAAACATTTATTCCGCTCCCTCCAAGCCACCAAGTTTTTCCAATCAACAGAGCTTGATTGGGCCGAAGCAGGACTCCAAGTTTGTAAACAAGGGTACAATATGCTTAACCTCTTGATCCATCGGAAAAATCTCAATTACCTTCACCTCGATTACAATTTCAATCTAAAGCCCGTGAAGACTCTCACTACCAAGGAACGCAAGAAATCACGGTTTGGTAATGCCTTCCATCTGTGCCGTGAAATCCTGCGTTTGACGAAGCTTGTAGTTGATGCCAATATTCAGTTTCGCTTAGGAAATGTGGATGCTTTTCAATTGGCTGACGGCTTGCAGTATACCTTTTCACACGTTGGGCAGTTGACTGGCATGTATCGTTACAAGTACCGGCTTATGCGTCAGATCAGAATGTGCAAAGACTTGAAGCACTTGATTTATTACCGCTTTAATACTGGGCCAGTGGGGAAGGGACCAGGATGTGGCTTCTGGGCGCCAATGTGGAGAGTGTGGCTGTTCTTTTTGCGTGGAATAGTGCCCCTGCTGGAACGGTGGTTGGGCAATTTATTGGCACGTCAATTTGAAGGGCGCCATTCAAAAGGAGTGGCCAAGACTGTCACTAAGCAACGTGTTGAAAGCCATTTTGATTTGGAGCTTCGAGCTGCTGTCATGCATGATGTTCTTGATGCCATGCCAGAGGGCATTAAACAGAATAAGGCAAGAACTATCTTGCAGCATCTCAGTGAAGCATGGCGCTGCTGGAAAGCTAACATCCCTTGGAAGGTGCCTGGTCTGCCGGTTCCAATTGAGAACATGATTCTTCGTTATGTGAAGTCGAAGGCGGATTGGTGGACAAATGTTGCCCATTACAATCGTGAACGTATAAGAAGAGGTGCAACAGTTGACAAGACAGTTTGTCGAAAGAATCTGGGACGTTTGACTCGTCTTTGGCTAAAGGCAGAGCAGGAAAGGCAACATAATTACTTGAAAGATGGTCCATATGTCACTCCAGAAGAAGCGGTTGCTATTTACACAACTACTGTACATTGGTTAGAATCAAGGAAGTTCTCACCCATTCCTTTCCCTCCATTGTCTTACAAACACGATACCAAGCTTCTTATCCTTGCTCTGGAGAGGTTGAAGGAATCATATAGTGTCGCTGTGAGACTGAATCAACTACAAAGAGAAGAGTTGGGTCTCATTGAACAAGCTTATGATAATCCTCACGAGGCTTTGTCTCGGATTAAGCGCCACTTGCTTACACAGCGCGCCTTCAAAGAAGTTGGCATTGAGTTCATGGATTTGTACAGTTATCTGATTCCAGTTTATGAGATTGAACCTCTGGAGAAGATCACAGATGCATACCTTGATCAGTATTTATGGTATGAAGGTGACAAGCGCCATCTATTCCCGAATTGGATTAAGCCGGCAGATTCTGAGCCACCCCCGCTTTTAGTCTATAAATGGTGTCAGGGTATAAACAACTTACAGGGGATATGGGACACAAGTGATGGGCAATGTGTTGTGATGCTTCAGACAAAGTTCGAGAAGTTCTTTGAGAAGATCGACTTGACTATGTTAAACAGGCTTCTCCGTCTGGTTCTTGATCACAACATTGCTGATTATGTTACTGCAAAGAACAATGTTGTGTTGTCTTACAAGGATATGAGTCACACAAACTCATATGGTCTCATACGTGGTCTTCAGTTTGCTTCTTTTGTTGTTCAATATTATGGACTTGTGCTGGATCTCTTGCTTCTTGGATTGACTAGAGCCACTGAAATTGCTGGTCCAGCACAGATGCCGAATGAGTTCATTACTTATTGGGATACTAAAGTTGAAACACGACATCCGATTAGATTGTATTCTCGATACATTGACAAAGTGCATATATTGTTTCGCTTCACCCATGAAGAAGCCCGAGATCTGATCCAGCGTTATTTGACTGAGCATCCTGATCCTAACAATGAAAATATGGTTGGATATAACAATAAGAAGTGTTGGCCCAGAGATGCGAGAATGAGGCTCATGAAACATGATGTCAATCTTGGGAGGAGTGTTTTCTGGGACATGAAGAACCGTCTTCCTCGAAGCATCACAACACTTGAATGGGAGAACAGCTTTGTTTCTGTTTACAGCAAGGACAACCCAAACCTACTTTTCAGCATGTGTGGATTTGAAGTTCGTATACTGCCCAAGCTTAGAATGACTCAAGAGGCATTCAGCAATACTAGAGATGGAGTTTGGAATCTGCAGAATGAACAGACCAAAGAACGCACTGCAGTTGCTTTCTTAAGGGTTGATGATGAGCACATGAAAGTATTTGAGAATCGTGTGAGGCAGATTCTCATGTCTTCAGGGTCAACAACGTTTACCAAAATTGTTAACAAGTGGAATACGGCTCTCATAGGTCTCATGACATATTTCCGAGAAGCAACTGTACATACTCAAGAACTATTGGATTTGTTGGTTAAATGTGAAAATAAAATCCAAACCCGTATCAAGATCGGATTGAATTCAAAAATGCCTAGCAGATTCCCTCCTGTCATTTTCTACACGCCAAAGGAAATTGGAGGTCTTGGCATGTTATCGATGGGTCACATATTGATTCCACAGAGCGATCTGCGATACAGCCAGCAGACAGATGTTGGTGTAACTCATTTTAGGAGTGGTATGAGTCACGAGGAAGACCAGCTGATTCCAAATCTTTATCGTTACATACAGCCATGGGAGAGTGAATTCATAGATTCACAGCGTGTTTGGGCGGAGTATGCGCTGAAGAGGCAAGAGGCTCAGTCACAAAACAGGCGTTTAACACTTGAAGATTTGGAAGACTCATGGGACAGGGGCATACCTCGAATCAATACTTTGTTTCAGAAGGACAGGCATACTTTGGCTTACGACAAAGGGTGGAGAGTGCGCACTGATTTCAAACAGTACCAAGTCTTGAAGCAGAATCCCTTCTGGTGGACGCATCAAAGGCATGATGGAAAATTGTGGAATTTGAACAATTATCGAACTGATGTCATCCAAGCACTTGGAGGAGTTGAAGGAATTCTTGAGCACACATTGTTTAAAGGAACATACTTTCCAACTTGGGAAGGTCTCTTTTGGGAGAAAGCATCTGGTTTCGAGGAGTCGATGAAGTACAAGAAGCTGACCAATGCACAGAGATCTGGTCTCAACCAAATTCCCAACCGTAGATTTACCCTCTGGTGGTCGCCTACAATAAATCGTGCCAACGTTTACGTGGGTTTCCAAGTGCAGTTAGATTTAACTGGAATATTCATGCACGGAAAAATTCCAACCTTGAAGATATCATTGATCCAGATATTCCGTGCTCATTTGTGGCAAAAGATTCACGAGAGTGTTGTCATGGATCTCTGTCAAGTCTTGGATCAAGAGTTGGATGCATTGGAAATCGAGACTGTGCAAAAGGAAACGATCCATCCAAGAAAGAGTTACAAAATGAACAGCTCTTGTGCCGACATCCTTCTATTTGCTGCTCATCGATGGCCCATGTCAAAACCTAGCCTGGTGGCGGAGTCAAAGGAGGTATTTGATCAAAAAGCAAGCAATAAATACTGGATAGATGTGCAACTTCGTTGGGGAGATTATGATTCTCACGATATTGAGCGTTACACCAGGGCGAAGTTTATGGATTATACAACAGATAACATGTCCATCTATCCATCTCCAACTGGGGTGATGATTGGGCTCGATTTGGCTTATAATTTGCATTCTGCATTTGGTAACTGGTTTCCCGGTTCAAAACCGCTGATCCAACAGGCTATGAACAAAATTATGAAGTCAAATCCAGCTCTATATGTTTTAAGGGAGCGTATAAGGAAAGGTTTGCAGCTGTATTCTTCTGAGCCTACAGAGCCGTATCTCTCTTCTCAAAATTATGGTGAGATTTTCAGCAACCAGATTATATGGTTTGTTGACGACACTAACGTTTATCGTGTCACAATCCATAAAACATTCGAAGGAAATCTCACAACGAAACCCATCAACGGTGCTATATTCATTTTCAATCCAAGGACCGGGCAATTGTTTTTGAAGGTGATTCATACTAGTGTATGGGCAGGACAAAAGCGTCTCGGTCAGTTAGCTAAGTGGAAAACTGCAGAAGAAGTGGCTGCTTTAGTTCGTTCTTTGCCCGTTGAAGAACAGCCGAAGCAAATAATCGTGACTCGTAAAGGAATGCTCGACCCCTTGGAAGTTCACTTGCTCGATTTTCCTAACATTGTCATTAAAGGAAGTGAGCTACAACTCCCATTCCAAGCGTGCTTGAAGATTGAGAAATTTGGTGACCTGATTCTGAAAGCTACCGAGCCACAGATGGTTTTGTTCAACATTTACGATGACTGGCTGAAATCGATTTCATCTTACACTGCTTTCTCTCGTCTCATATTGATTCTTCGTGCGCTTCATGTCAACAATGAGAAGGCAAAGATGTTGCTCAAGCCTGACAAAACAATCACCACTGAGCCACACCACATCTGGCCTTCTCTCAATGACGATCAGTGGATGAAGGTTGAAGTTGCATTGAGAGATCTAATACTTTCAGACTATGCGAAGAAGAATAACGTCAACACTTCAGCTCTTACACAGTCGGAGATTCGCGATATCATACTTGGAGCTGAGATTACTCCACCTTCGCAACAAAGACAACAGATTGCGGAGATTGAGAAGCAGGCGAAAGAAGCGAGTCAACTCACAGCTGTTACGACAAGGACAACAAATGTGCATGGTGACGAACTTATTGTCACTACTACGAGTCCGTATGAGCAGAATGCTTTTGGGTCGAAGACTGATTGGCGTGTGAGGGCAATATCAGCCACGAATCTCTATCTCCGAGTTAATCATATATATGTCAACTCAGAGGACATAAAGGAGACTGGCTATACCTACATTATGCCCAAGAATATTTTGAAGAAGTTCATTTGCATAGCGGATCTTCGAACCCAGATTGCAGGGTACTTGTACGGTCGGAGTCCTCCAGATAATCCTCAGGTGAAAGAGATTCGATGTATCGCCATGCCACCTCAATGGGGTACGCACCAGCAAGTGCATCTCCCAACTAACCTACCTGAACATGAATTTTTGAATGACATGGAGCCATTGGGATGGATGCATACCCAACCGAACGAGCTACCTCAGCTATCACCACAGGATTTGACAGCTCATGCTCGGATTTTAGAGAGTAACAAGCAATGGGATGGTGAGAAGTGTATTATCTTGACTTGTAGTTTTACTCCTGGTTCATGCTCGTTGACTGCTTATAAGCTGACCCCGACTGGATATGAGTGGGGACGTGTGAATAAGGATACTGGAAGTAATCCTCATGGGTACTTGCCGACACATTATGAGAAGGTCCAGATGCTTCTGAGTGATCGGTTCTTTGGTTTCTATATGATTCCGGATAATGGTCCATGGAATTACAACTTCATGGGAGTGAAGCATACCCCAAGCATGAAGTATGGTACCAAGCTTGGAATACCTCGAGAGTACTACAATGTGGATCACAGGCCGACTCATTTTCTGGAATTTGGTAAGATGGAGGATGGTGACATGGCTGAAGGAGACCGTGATGATACATTTACTTGAGGTGGTTCTCTCACCGAGCATGAGTGCTGATGAAAAATGACCATATAATTGACTTTTTGTTGTAAGCCATGTAACAATCTTGATTTGAGGTGTTTGTAAATACAAAAACTGAGTTTATTCTTTTTGCTATTATAGTCCTAAACATAGTAAAAAAAAAAAAAAAAAAAAAAAAAAAAAAAAAGT

>transcript_14 full_length_coverage=2;length=7319

GGGGCAACCAACGGAGTAGCCATTACTTGTGCTCTCCTCATCTGGACACTCGGTTGTCCACATTCATTTCGGTTCTTAATCTTTTTGATTTTATTTCTCTCGGTTTATCATTTGTTCTATCGCGCTGGCGGATAAGATGATCCGAAAAATCTGGATCTGGTTCTCCTCTGGTTTTGTCCGTTGCTCCAGACAAGATCCAAAATTGTTTTTGCCCGTTGCTCTGTTTTTTAGCAGAGCTGAGGATAAGGTGACCGTTGGTGATGGTCTTTTAAATAGCTTGCTCTGGAAAATTTATGTTTCGCCGATTCTTATTAAAAAATTCAGAGGGCCATTTACTCTATAAACTGACCTATTGCTCTCAATCTGACCGAGAGGATGGAGACGTGGTTTTAAATCACGGGAGCTCCGGCGTGTTCACATTTCGTGGGTCTTGATTCTCTCAACCATAGGCGCCATTTAGAATTGGAACTGGGAGGAGATCCACGGCCTTAGAGGATTCAGATCGTGACCCATGGAGGATCACTGACAGAGCACCACACATCTTGAACTGAGAAGGGAGAAGATCGCTGGGTAAGGTTTCAGGGACACCGTGCGTCGACTTATCTTCAGGCAAGCAACTTTCTCTGTAACGGGCCTGATACAGAGGAGAAAGAAAGTTTGAAGTTCGGACTCTCTGTGGTGATTTTTAGATGGAAAAGAGGAATGATAGATTGTCAAGTTGAGATTTTCCAGCGAGAAGGATAGATTGCGTCTATTGGAAGCTGCGTCAGTTAGTCAAACTGAAACTGTAGGAAGGTTCCGTGAATGAACTTGGTTCTCTTTTGGAACTACTCGAGAGGTTGGAGACGCGTTTTTTAAAAGAATAAAAATCTGGAGCTCCGATGTGCTTATTTTTCAGTAAAGGAACAGAGTTCCATGAAAATATTCCTTTTACACGTGGTTCCTTTCTGGCGTGGCTGACGAAGCTTATCAGAGAGAGACTCTCTCCTGTAGCTTAAAGAGGACGCGAGCTAGCATTCTGTTTTTCCCGTGAGCTTATTCTCTGAAACAGAGCGGACTGTTACTTTTCCTTCCCAGCGAGCTTAACTGAAGAGAAGTGGCAGGCAAAGCTTATCGTGCGAAGAAGCTTAATGGGTCCTTTATGGATGGTGACCATTCGAGTTCATATATGGCATAATGTTGGAGGGCTTGTGAGTGGATCCGAATTGGGAAAAGGCCTAAAATCTTAACAAAAGAAAAAAAAAAAAACTCTTTCAGATAGTGTGTTGGTCAGTAGCTCCTTCATTTATCCTTCTCCATATATTTGTATTTCATCACTTTATATCTGGCCTGTGAGTTGTTGTCATCACTTAGCTTACATGTTGCATATTTTGCATTTGCATATTTTGTTCATGCCATTATATATGCATGTCATTTGTGTCTTGCATTCATAATTCTTGAGTTCTGATCATAGGGCATGCATATACTTTTAGGTGCTCCATTACATCTATGTGAACATGTTCATGAACACTTATTCTGATTGCATTTCTATTACTTGGCATATAGTGTTTGTGCATTCGTCATATCATTATTGCATATTTCATATGTGTTCATCACTCTACAAATTGTGAGAATTTCATGATCATTGAGTTCTGAGAAATTGGACTCAAGACTTGGGTGAGTAAGCCCATTGGTACCCTAGAAATTTATTGACCATATTTTTGTGGGTATGTGATTTATATTTTGTGCTCAAAATTTGTAATCAATTTTGTTATTGGATTCGGTTGTTGGTGAACCTCTTAAAACCATCATTTGGTTTGGAACGGTTATTGCTCATCCATAAAAGCATACCTTAGGTTCAAGTTTGTCCTCAATCCAAAAGCTTGTAGGTTGTGTGTTTGGTAAGAGTTCATAGGTTGAACTCAAACCAATTTGAGTCCACACTTTAGGTTACTAGCTTGTCCTAAAATTAAAAGCTAGTAGGTTGTGTGTTAGCCTAGGAAACATTCAAGTTCTTGTTGATCTTAGAAAACATGTTTAGGATTTTCTTTGCAGGTTTGTCCTTGTGATTTAGTCACTAAACAAAATGTGTAATTAGGTTCTTTGTGAGCCTTGTAAACTTTCAAGTTTAAGTTTAGCTTAGAAAAACTTTGTAAAGGTTTTTGGTCAACCTAGAAAACCAGTTTTTATTATAGTGGAAGAAACCTTAACGGGCCACATAGGCCTTGTTAGGATGAGTGGATGTAGGCTAAGTCAAGGAGGCTTAGTTGAACCACTATATATGCTTGTGTTGATTTTTCTTGCATGCTTTACTTTATTCGTTGCTTATCTATAGTCTTCATAACAATTCAACTCAAGCTATTGATTCTTCAAAGGAAAATCAAACTCTCAATTTTCACTCAACTTGGTTTAACTTAAAATTGGCAAATGATTTTCAAAAAGGTTCAAAAGATCCTATTCACCCCCCTCTAGGATCCATAGCTGCACTTTCAATTGGTATCAAAGCAGGTCACTCAATATAGGTTTAAACACCTGAGTGAGATCCTTTAGGGTTTCGTAGTCTTCTTTTCAGCCATGATGTCTTCCTTCGATACTTCGATCGATAAAGCTCCCATACTTGATAGTACTAATTATGATACTTGGAAATTTAGAATGAAAATCTTCCTTAAATCTTTAGACTATGGAGTTTGGTTTATTGTTGAAAAAGGTTGGTCTGAACCTGTGAAAATTAATCCTAAAAACAAAAACCGTACTATTCCTAAACCTCCCATAGAGTGGTCTCAAAAGGAGAAGAGTGACAGTGAATTGAATGCTAAGGCTTTAGAGATGATACTTAGTGCTGTCACCCAAAATGAGCCTAATCTTCTAACCACTTGTACTTCCGCCAAACATGCTTGGGATATCCTTAGGATGAAGTTTGAAGATGGTGTTGATCCTCCTTTAATGACTCTTACGAGTACTACAAATGACACCTCTTCTAAAGAGTGTCAAGAATTAGAGTCTGAAGACGAAAGTTCTGAATCGGAAGATGATCAAGATAATGACGAGGAAACCATTGAAGAATCCTATGAGAACCTATATAAAGATTTTGTGAGGATTGGTCTTGAGAATCAGAATTTGAAATGTCTCAATAAAAGTCTTCTTTCTAAGATCACTGATCTTAAGTCTTCAAAAATTTCTAAACTTGAGGATGATGTTGAGAGTTTAAAGTGTAAGAATCTTGAGCTAAAAGAAAGAAATGTCAACATTGAGTCTGAACTTGAAATTGCCTTGAATACTATTCGCACTGTAGAAGATAAGAATGTGATCCTTGAAAAGGGAATGGTTGATCTCAAGGACAAAATCTATGACCTAGAATCACAAAAGAAATTCGTTGAAATTTCTTGCTCCAAGGATGAGAAAGTTGAGCTGCAAATTAAAACTCTTGAATCTCAAATTTGTGATCTAAATGATTTGATGTTTAATCTCAAAAGCAAAAATGTTAAATTAGAAGATAAAGCTCACCAATATGACAAATTGAAGAATGAGTGTGACACTGCTTTAAATGAGATTCTGAACGTGGAAGAAAAGCTTAAAGTGTTTGAAAAGTCAAATAAGGAACTAATTGCAAAGATTGTTGATTTGGAAAACATAAACTCGAAGTTCCTTAGTGGTAGGAAGAAACTTGATGAGATGTTAAGAGCTGGTAAGAGTTATTATGATAGGACTGGACTTGGGTACCTCTCCGAGACAGCGAACAATTTAAATCCCAAGATAAAGTCCATTAAAGTGACTCAAAAACCTCTTGCTCGATACAACACTCAAGGGTACACATACCCTAAACCGAATACGAAAAACTTCCATTGCTATTACTGTGGTATTCGTGGTCACAAACAGTTTGAGTGTAGACTTAAAAGGATTAAGAAGCAAGAATCTTGGAGATCCACTCACACTAAGAAAGTCTATCGGATTGTGAAAATTCATGATGAACCTTGGTGGCACCGCACTTCAAAAGTCTCTACATCTCAAAAGATGAAAGGAAAGATGCATGAGCAATGCTCAGTTGCCTTAACCGCATGTCAAACTGGAAAAGACACTGCTTGGTACATGGACAGTGCTTGCTCTAAGCTTATGACAGGTGACAAAGATAAATTTTGGTTTCTAGAAGAGTTTGATGGTGGAGATGTTACATTTGGAGATGGATCAAAAGCAATGATCATTGGTAAAGGGACTGTGCATGTGTTTGACACAACTAAGATCGAGAATGTTCTTCTTGTAAAAGGCTTAAAGAAAAATCTCTTAAGTGTTGCTCAACTGTGTAAGGATGAGGACGTCGATATTATCTTCAACAAATCAAAATATGAAGTGTTTGACAAAGATCGTAATGTTTACATGATTGGAAGAAAATCTTCAGAAAATTGTTACACCTTGGATGTTGAGAATTGTGAAGACAACCGTGTTGGAATTTCATTTAACGTTGTCATGGAGCAGACCAACCACTTTGGCAAGGATCAGCAAGTGAATGCTACTAATGACACCATTGCTAGAGAGCCGGATGTTAGAGTTATCACCAGAAGACGATCTCAGGATGTTGAAAGACAAGTGTGCCTTACTTCTAACATGGACTATTATCCCACTAAGCATCAATTGTCGAACATCCTTACTAATTCTCTAGAAACCACTGGCTTTGAGTTTCTTCGCACTTCCATTGGAGTGTGTCAAGTAACTTAATCTTTCCTCACCGCATTTCATTTTCTTTTCTTTTTGTGTGTGTGAGTTTTAGTGTTTATGTTTGAGTCGATCTAAGTTTTGCATTGAGTCATTTTTGGTGTCTTGCGTTTTGCATCGAGTTCATACATTTCATTTTTGGTATCATTCGTGCATGTGTCTCATTCTTGCATTTTCCCCTACCATGCATTATTCTTCATTATTATATATGGCATTTGTTATGACAAAAAGGGGGAGAAAAGGTATTCTCTTGTTTGCTTTTGCAGGTATGGAGACAGGGGGAGCAATACAAAAGCATGAATGGAATTATAAATCTCAGGGGGAGCAAAGCTCGATTTCTTGAAAATGAAGAAAGTTCGTTTATTGAAGTTGAAGAATGCTTGATTCTTCAAGACGAAGAAAATACATATCTTGAAGTTGAAGAAAGCGCGTTGCTTAAAATTCAAGAAAGCTCAAGTGTGCAAGAATCGCTCCTGCAATCAACCAAGACGAAGAAGATGTTTTGTCATAAAATTGCCATTGGGGGAGATTGAAACGGCACCACATCGCACCGTTTTGTGAATGTCATTTTTATGACAAACAGGTTTGTGAAACTAGGTGTCCTTCCTCCCGGTTGCACCAAAGTTAGTGCAGAGTGGGAATTTGAGTCTCATGCATTTAGATTGTCATTGCATTGCATTCATGCATGTGTGTGTGTGTTGAGTGGTTGATTGCATGGTTTAGTGGACATTAATCCAAAATTATTTTCGTGTGTGTTGATCCGAACATGATGGGTTGAGGTGATTGATTTCAAATTCGTTTTAACCGAAAATCATTAAACAATTTTCTTTAAGAAAATCTTGCTCTTTATAAGTTGCTTCTACTTAATGATATTTATCCTCTCAAAAATATTTTCTAAAATTTCCCTTAAAATTTCTTAAGGTTTTTAATTTTACTTTAACACGATCAAAGAAATTTGAGTGTTAAATCTTTGATTAAAAATATATCTTTATTTTGTTAAAACAAAAGAAAGGGAAGATATTTTTTATTGGATTCTTGGTTTGACCCGGTCCGCTCACAAAATGCTGGAGAACAAGATTTTGAAAAATATGAGACAGGCAAAATGGGTATCGCTGAGGATGGTATATGCCTCGCATTTTCCAGTTGTGGAAATTACTAATTCGTCCTCCTTCATACGCGCGTGAGGTATTCGTGGCTGTCATGAATTGTGCGTGAGCTTTCGTGCTCTCAGGCTTTGGGTGAAGAAACAGATTCTCTTTGTGACCGTTGGTCACATGTTGTCGAGCTGGCGGATAACGTTTCTTATCCTCGCCTGTTGCTATTGGTTATTCCATTTCATATTATAACCGTTGAAGGGATAGGGTTGTAGCGCAACCAACGGAGTAGCCATTACTTGTGCTCTCCTCATCTGGACACTCGGTTGTCCACATTCATTTCGGTTCTTAATCTTTTTGATTTTATTTCTCTCGGTTTATCATTTGTTCTATCGCGCTGGCGGATAAGATGATCCGAAAAATCTGGATCTGGTTCTCCTCTGGTTTTGTCCGTTGCTCCAGACAAGATCCAAAATTGTTTTTGCCCGTTGCTCTGTTTTTTAGCAGAGCTGAGGATAAGGTGACCGTTGGTGATGGTCTTTTAAATAGCTTGCTCTGGAAAATTTATGTTTCGCCGATTCTTATTAAAAAATTCAGAGGGCCATTTACTCTATAAACTGACCTATTGCTCTCAATCTGACCGAGAGGATGGAGACGTGGTTTTAAATCACGGGAGCTCCGGCGTGTTCACATTTCGTGGGTCTTGATTCTCTCAACCATAGGCGCCATTTAGAATTGGAACTGGGAGGAGATCCACGGCCTTAGAGGATTCAGATCGTGACCCATGGAGGATCACTGACAGAGCACCACACATCTTGAACTGAGAAGGGAGAAGATCGCTGGGTAAGGTTTCAGGGACACCGTGCGTCGACTTATCTTCAGGCAAGCAACTTTCTCTGTAACGGGCCTGATACAGAGGAGAAAGAAAGTTTGAAGTTCGGACTCTCTGTGGTGATTTTTAGATGGAAAAGAGGAATGATAGATTGTCAAGTTGAGATTTTCCAGCGAGAAGGATAGATTGCGTCTATTGGAAGCTGCGTCAGTTAGTCAAACTGAAACTGTAGGAAGGTTCCGTGAATGAACTTGGTTCTCTTTTGGAACTACTCGAGAGGTTGGAGACGCGTTTTTTAAAAGAATAAAAATCTGGAGCTCCGATGTGCTTATTTTTCAGTAAAGGAACAGAGTTCCATGAAAATATTCCTTTTACACGTGGTTCCTTTCTGGCGTGGCTGACGAAGCTTATCAGAGAGAGACTCTCTCCTGTAGCTTAAAGAGGACGCGAGCTAGCATTCTGTTTTTCCCGTGAGCTTATTCTCTGAAACAGAGCGGACTGTTACTTTTCCTTCCCAGCGAGCTTAACTGAAGAGAAGTGGCAGGCAAAGCTTATCGTGCGAAGAAGCTTAATGGGTCCTTTATGGATGGTGACCATTCGAGTTCATATATGGCATAATGTTGGAGGGCTTGTGAGTGGATCCGAATTGGGAAAAGGCCTAAAATCTTAACAAAAGAAAAAAAAAAAAAAAAAAAAAAAAAAAAAGT

>transcript_15 full_length_coverage=2;length=7227

GGGATCCCTAACAATTTTCTGTTCACCGGTCTCTTTTCTCTCATACTCAGATCTGTGATTTTCCATATTGGGATGTTTGTGATTTTGCAGATTATGATGTTTGTTGTGCCGCTGGTGGGATGAATACCGATGTTTGTTTTGCCGGAGAAGAGAAACTAATAGGACCTCATGTGCTTGCTTCACCGGCGGTTATGGTGAATGAAGATGCTTGCGGCCGGGGAAAAAGATGAGATTCCCGTTTGTTTGTGACCGTGACAGCGAAGAATTGCCTAGAGCCCTGAAAATGCCAGGGCCGGCCCTGCCTCTCTAGATCCACCTCCAGCAAGAACAGCTTACTCTTCTTCCTCTGAACCTCAAGCTATCCACTGAACTCTATCAGAGTCCAACCAATGTACAGTGGAAACAGAAAAGGAGATCGGAGAGACCCACGAAACTTCAAGAAGCAGAAGCTCATTCGCACCGCCGAAGAAGAAATCGAATCCAAGCTAGGCTTTGATCTCTTCTCAGAAGGAGAAAAGAGGCTTGGATGGCTGCTGACGTTCGCTTCTTCAGCTTGGGAAGATAAAGACTCCTACAAGGCATATAGTTGTGTTGATCTTTATTTCGTTTCTCAGGATGGTTCTACTTTCAAATCAAAGTACAAGTTCCGTCCTTATTTCTACGCAGCCACAAAGGAGAAAATGGAAATTGACGTCGAATCATATTTAAAACGACGCTATGAAACTCAAATTGCTGATATCGAAATTGTGGAGAAAGAAGACCTTGATCTAAAAAACCATTTGTCTGGACTGCACAAGTCCTATTTAAAAATATATTTTTATACCGTTCAACAATTAATGAACGTGAAGAGCGATCTATTGCATGTCGTTGAAAGAAATCAGGCAAAATTAAATGCTGCAGAAGCTTACGAGTCGATCTTAAATGGGAAAAGCGAACAAAGACCACAAGATTTTATTGATTGTATTGTGGATCTCCGTGAGTATGATGTTCCTTATCATGTTCGCTTTGCCATTGACAATGATATCAGATGCGGGCAGTGGTACGATGTTAGTGTATCCAGTTCTGGTGTTATGCTAGAGAAGAGGGCTGATCTCTTGCAACGTGCCGAAGTTCATGTTTGTGCTTTTGATATTGAAACTACAAAGCTTCCTTTGAAATTTCCAGATGCTGAATATGATATGATAATGATGATTTCATACATGGTTGATGGACAAGGATACCTGATTATTAACAGAGAGTGCGTTGGAGAAGATATAGAGGATTTGGAGTACACTCCAAAACCTGAATTTGAAGGGTATTTCAAAGTGACAAATGTTGAAAATGAGGAAATGCTTCTTAAAAAGTGGTTTCTCCATATGCAAGAAGTGAAGCCCGGCATATATGTCACGTACAATGGTGATTTTTTTGACTGGCCATTCTTGGAAAGAAGAGCAGCCCATCATGGGTTTAAAATGGGTGATGAGTTAGGGTTCCAGTGTGACAAGATTCAAGGGGAATGTCGTGCTAAATTTGCTTGCCATCTGGATTGTTACGCTTGGGTCAAACGTGATAGTTATCTTCCTCAAGGAAGCCAAGGCCTAAAGGCTGTTACAAAGGCCAAGTTGGGCTATGATCCACTGGAGGTCAAACCCGAGGAAATGGTTCGCTTTGCAATGGAAAAGCCGCAGATGATGGCCTCCTATTCTGTTTCCGATGCTGTTTCAACTTACTACTTGTATATGACCTATGTTCACCCCTTCATTTTCTCTCTTGCAACTATAATACCCATGCCACCGGACGAGGTTTTGCGCAAAGGAAGTGGAACCCTCTGCGAAATGCTTCTTATGGTTGAGGCATACAAGGCAAATGTTATCTGTCCTAATAAACACCAATCTGATCCAGAAAAGTTCTACAATAATCACCTTTTGGAGAGTGAGACATATATAGGTGGCCATGTGGAATGCCTTGAAAGTGGTGTTTTCAGATCTGACATTCCGACTAGCTTTAAGCTTGATTCATCCGCCTTTGAGCAACTAATCATTAACCTTGATCGAGACCTGCAATATGCTATACGGGTAGAGGGTAAGATGGACTTGGAATCAGTCTCCAACTACGATGAAGTGAAGAATTCTATCATGGCGAAGCTTGCGAGATTGCGAGATGAACCTATACATGAAGATTGCCCCCTTATTTATCATCTTGACGTTGCTGCAATGTATCCGAACATTATTTTGACAAATAGGCTTCAGCCACCATCGATAGTTTCAGACGATATCTGTACTGCATGTGATTTTAATCGTCCGGGAAAAACTTGTCTTCGCAATCTTGACTGGAAGTGGCGTGGGGAGACATTCACGGCAAACAAAAGTGATTATTATCATTTGAAGAGGCAACTTGAGTCTGAGCTTGTTGATGGTGTCGATGGGCGGTTGTCAAGATCTTTTCTTGACCTTCCTAAGGCAGAGCAACAAATAAAACTCAAAGAGCGACTAAAGAAATACTGTCAAAAGGCATATAAACGGGTACTTGACAAGCCAGTCACTGAACTTCGTAAAGCTGGGATCTGTATGAGGGAAAATCCTTTTTATGTTGACACTGTCAGAAGCTTTCGTGATAGGAGGTATGAATACAAAGGACTTAATAAAGTTTGGAAGGGGAGGTTATCTGAAGCTAAATCTAGTGGAAATTCCATAAAGATCCAAGAAGCCCAGGATATGGTAGTTCTTTTTGATTCATTACAACTTGCTCACAAGTGCATCCTTAATTCCTTCTATGGATACGTCATGCGCAAGGGTGCAAGATGGTATTCAATGGAAATGGCTGGAGTAGTTACCTATACTGGAGCAAAAATTATTCAGAATGCTCGCTTACTAGTTGAAAAGATTGGAAAACCACTAGAGTTAGATACAGATGGTATCTGGTGTGCACTACCTGGATCTTTCCCTGAGAACTTCACCTTCAAAACAAAAGACTCGAAGAAGCTGACAATCTCATATCCTTGTGTTATGCTTAACGTTGACGTGGCTATAAACAATACAAACGATCAGTACCAGACACTCAAAGATCCTATCAATAAAACATATGCAACGCACGATGAATGCTCAATTGAATTTGAAGTAGATGGGCCGTATAAGGCAATGATTATTCCCGCTTCCAAGGAGGAAGGTATTTTAATCAAGAAGCGATATGCTGTTTTTAATGATGATGGAACCCTGGCGGAGCTTAAAGGTTTTGAGATCAAGCGCAGAGGTGAGCTGAAGCTGATCAAAGTTTTCCAGGCTGAGCTTTTTGACAAGTTCCTCCACGGGTCAACCTTAGAGGAATGCTATTCAGTTGTCTCGTCTGTTGCAAACCGTTGGCTTGATCTTCTTGATAATCAAGGAATGGATATTGCGGATAGTGAATTGCTTGACTACATTTCAGAATCAAGCACCATGAGCAAGTCCTTGGCAGAGTATGGTGAACAAAAGTCATGTGCAGTGACCACTGCAAGGCGTCTTGCTGATTTTCTTGGTGAGGCAATGGTTAAAGACAAAGGATTGCGCTGCGAGTATATAGTGGCATGTGAACCAAGAGGAACACCTGTAAGTGAGCGTGCTGTTCCTGTTGCAATATTTGAAACCGATGCTGAAGTTATGAAGTTTTATGTACGGAAATGGTGCAAAGTTTCGTCAGATGTTGGCATTCGATCCATTATTGACTGGTCCTATTACAAGCAACGGCTTAGTTCATCAATTCAAAAAATTATTACCATTCCTGCTGCAATGCAAAAGGTTGCAAATCCTGTTCCTAGGGTCATTCATCCTGATTGGCTCCATAAAAAGGTTCGCGAGAAGGAGGACAAATTTCGCCAGCGAAAATTAGATGATATGTTTAGCTCAATGATCAAGCACGATACAGTAAAAATGAAAAATGATGCTGAAGAGAATGGTGAAGATTTGGAGGACTTTGGAAATAAAACCCGAACTTCCATTGTTGGACCTAGGCCCATTGTCCGTTGCTATGCGGTTAATAACAAACCAAGCTGCCCACTAAATTCCTTGCAACAGCCAACTGATCACAGGGAAAGTGTGCAGCAGCTGATGTCACCATTGCAACAAAATGCTGCTTGTGTTGAAAATATTGATAGAAATGTAGATTATCAAGGATGGCTAGAAATAAAGAAGAGGAAGTGGAAAGATACTCGGGACAAAAGGAAGAGGCAAAGGTTGGGCAATTCAAGAACATCTCACCAAGATAATAATGTTTCTGAACTGCCTGGTGGTGTGATAAATCATAGACAAGCTCCGGAGAGAACTGGCGTGACTTCATATTTCAAGAGACACGAACTTGCCTTGTACGATGCCACTGGCAGGTAATACAACTTCTTCCGGGGTCACAGCATGGCCAGTTTTTAGCTTGGGTGATTGTAGATGGAATCATGCTCAAGATTCCCATTACTGTTCCCAGAGTTTTTTACCTGAACTCTAAAGCACCCATAACAGAAGAATTTCCTGGAAGGCATGTCAATAAGACTCTTCCTCATGGACGTCACAGCTATAATCTGATCGAGGTAGTAATTGATGAAGATCAGTTTCGGGCAGAAAATAAGAAACTTGCAGCTCATCTTGCAGATCCAGAAGTTGAGGGGAATATATGAAACAAGGGTGCCATTAGAGTTTAGTGCTATTCTCCAGATTGGCTGTGTCTGTAAAGTGGATAAAACAGCAAAGAAAAGAAATGCCCAAGATAGTTGGGATTTTATCAGAACTGCATATGAAGACCACAACAGAATGTTCTTATCTAGAACAATCTTTCACTTACTTTTACTTGTATCATAGCATCTCTGAGGGACGAGCTATATACGTTGGGTATTTCCCTGCATCTAAAGTGATATCTGTTGTGGTGGTTAATCCTTTCCAGAATAAAGAATTATCACCATCTATCGTTGAAAAACAATTTCGTGAAGCTTGTCACACAATTTCTATTGAACCTTCACCACCTAAGAGTGGTATCACTTTCAAGGTTGTGTACGTTGGATATGTCAAAGATGCAGAAAAGGTTCTGCAGAGAACAATTAATGAGCTCAGACATCAACATCATGGACCTGCAATTGCCGTTATTGAATGCCCTGATGTTCATTTAATTAAGTCAGGAATACAAGCTCTGGATGATTTCCCATGTGTAACCATCCCTTCCAATGCTCGTGATAGTCACTATCAGGCTCTTGGATGGCAAATTTTGGCTGCAAAAATTGCAATGCAACGATGTGCTGCATCATCCCAGTGGTTAAATGAAAGGATCTCTCTTTCAAAATATGCACATGTGCCGTTGGGGAATTTTGAAGTTGATTGGCTTATATTTATTGCAGATATGTTCTTTTCAAGAGCATTACATGATCAGCAGCAGGTACTTTGGATATCTGATGATGGTGTTCCAGATTTAGGAGGCCTTAATGAAGAAGACACATGTTTTGCTGATGAGGTCCAGCAACCCGTTCTTACATATCCCGGAGCATATAGAAAAGTTACCGTGGAGCTAAAGATTCATCACCTGGCTGTTAATGCTCTTTTGAAAAGCAACCAAGTGAATGAAATGGAGGGAGGTGCTTTATTTGGATTTGATCACGATGTGAATTCTGGGTCCCATAGTCTGGATGAACAGTCTGGGTTTGATGAAGCTACCTCATGTGCACCTGCATTTCGTGTCTTGAAGCAATTGATCCAAAGATGCCTTGCTGATGCAGTAACATCTGGAAATGTATTTGCTGATGCAATCTTGCAACATCTGTATCGATGGCTTTGCAGCCCGCAATCAAAACTTCATGACCCAGCTCTTCACCGTGTACTTCACAAGGTCATGCAAAAGGTGTTTGGATTATTCTTAGCCGAGTTTCGCAAATTGGGTGCCACAATCATATTTGCAAACTTCTCAAAGATTATTATAGACACAGAAAGCCAGATTTAACAGCAGCCAAAGCTTACTGTGACAGTGTACTCAAAACTCTGCAAACCAGAGACCTATTCGAGTGGATTGAGCTTGAAACCGTTCACTTTTGGCATTCCTTGCTTTTTATGGATCAGTATAACTACGGTGGAATCCAGGCCAGAGCCCAGGGTGGACCATCGGTAGGTAGTTCAAATTCAACAAATGACTCCATACACGATGAATCTCAAGTGGAAATTGTATCAAGCTGGAATATTTCTGAGCACTTACCTAAGGCAACTCAGGATCATTTTGTTTTAATCGTTTCCGAATTTCTATACAATCCATGGAGACATGCACAAGAACAAGCTGCAATGAGAGCATTTTCGCAGAATGGTACCACGTGTACTCCAATCACTGGTGCAGCTGCTGAGAAGTTTGAATCACTCATGACAGATTATTTAAAAGAGCAGATCACCTCTTACTTCACAGACAAACTTCTTAGGACTGTTCGCGATATTTCTCTACATATGAAGGGTATGAACAAATCTGACAATACCCAACCTATCCCACAACTTGCCGGTGGTACCCATACCGGGGATTCAGCTCTGGAGTTTATTAAGCACGTTTGTGCTGTTCTCGCTCTTGACCAAAATGTTCAACATGATATTCTGGTTATGAGGAGGAATCTGCTAAAATTTGTGAGTGTCAGGGAGTTTGCTCCAGAAGCAGAGTTTCGAGATCCTTGCATGTCATTTGTCTTACCCAATGTCATTTGCAGCTATTGCAATGACTGCAGAGACTTAGACTTGTGTCGCGACTCAGCATTATTAGCCCAGGAGTGGCGTTGTGCGGTACCACAATGTGGACAGCCTTATGACCGGGAGGTGATGGAGAACGCTCTCCTTCAAATTGTGCGTCAGAGAGATCGTCTCTACCATGTACAAGACTTGGTGTGCCAAAAGTGCAACCAAGTTAAAGCAGTACATTTAGCCGAGCAATGTTCTTGCGGTGGTTCATTCAGGTGCAAGGAAGATGCGCCTGAATTCCGAAGCAAAATGCAGGTTTTCTTGTACATAGCTGCGCATCAGAAGTTTCAACTTCTTAAAGAATGCACCGAGTGGATTTTGGAGTACGATAAAATTGTAATTAACCTAACATTATAAGAGTGGAGATAACAGCAGCGTAGTGTATCTTACATCTTGTTGTGATTAATTCTGATTTATATATTTATAAATTGTATTTAACTTTTTAATCATTGTAACTTGAGGATGAATGTAGCTTCATTATTAGTATAATTAGTTATTTGATTAAAAAAAAAAAAAAAAAAAAAAAAAAAAAAGT

>transcript_17 full_length_coverage=2;length=7184

GGGATCTTTCAGTGTAAAGCGATTTTTACTTTCTCACACAGTAGGATTATAATTACTTCATGTCTCTCTATCTTCACCTGAAACAATTTGAGGAACACAAAAGGAAGATAAAAACCAAACCCTAACTCATCTCTCTCTTCTGACCTCTACACAAAGCCCTAATTTCAGTTCACATTCACTTTATTCCGAGCCATATTAAGCTTCAAAACCCAAGTTTCAGAGTTCAATCAAACGACAAAATGGCTCATTTGAGGAATTCACGACTATACTATTGGCAGTTGAAGCCGCTGCTGTAAGTAGGGATTTGATTTGTTGAAGGTGTTGTTGGTTACATGCATATGGTGTAGTTGAGGGGAGGGGAAAGGGCGAGGAGAAGCCATGGAAGGAGACGACCGTCGGCTTATGGTGGCTTGCGTTATTTCAGGCACCCTTTTCTCCGTTTTGGGTTCGGCTTCTTTTTCTATACTCTGGGCAGTAAATTGGAGGCCGTGGAGGATCTATAGTTGGATCATTGCCAGAAAATGGCCAGATATCTTACAGGGGCCTCAGCTGGGTATATTATGCAGTTTTCTGTCTTTATCCGCATGGATAGTCGTTATTTCTCCAATCGCCGTGCTCATTGCGTGGGGGTGCTGGTTGATTGTGATATTGGGTCGGGATATAATTGGTTTGGCTGTCATAATGGCTGGCATTGCTCTTCTATTGGCATTTTATTCAATCATGCTTTGGTGGAGAACACAATGGCAAAGCTCAAGGGCTGTTGCTGTCCTCCTCCTTCTAGCTGTTGCCCTACTCTGTGCATACGAACTTTGTGCCGTGTATGTTACAGCAGGTTCAAGTGCATCTCAGCGATACTCACCTTCTGGTTTCTTTTTTGGTGTATCAGCGATTGCCTTGGCGATTAATATGCTATTTATTTGCAGAATGGTTTTTAATGGGAATGGCTTAGATGTGGATGAGTATGTGCGGAGGGCTTACAAATTTGCTTATTCTGATTGTATTGAAGTGGGTCCTGTGGCTTGTTTACCAGAACCACCAGATCCCAATGAGTTATATCCTCGGCAATCCAGTAGGGCTTCACATCTTGGGCTTCTTTACCTTGGTTCGCTTGTAGTACTTTTTGTGTACTCCCTCTTATATGGCCTGACAGCTAAGGAGGCACACTGGCTCGGGGCAATTACTTCAGCTGCTATTATCATTCTCGATTGGAACATGGGGGCATGCTTGTATGGGTTTCAGCTCCTTGAAAGTCGTGTTGCAGCACTTTTTGTTGCTGGCGCATCTCGGGTTTTCCTCATTTGCTTTGGAGTGCATTACTGGTATTTAGGACACTGCATTAGTTATGCAGTTGTAGCATCTGTGCTATTAGGTGCTGCTGTTTCTCGTCATCTATCAGTTACAAACCCATTAACTGCAAGGAGAGATGCCTTACAGAGCACAGTGATTCGCCTGAGAGAGGGATTCCGCAGAAAAGAACAGAGTAGTTCATCTAGCTCATCTGAAGGTTGTGGCTCAAGTGTGAAACGTAGCAGTAGTGTTGAAGCGGGTAACCTTGGTAATGTTATTGAAGCTAATAGCAGAAGTACAGCTCATTGCACAGGTGATGCTAATAACTGGAATAATGTAGTACTGTGTAGAACAGCTAGTTCTCATGAGGGGATTAACAGCGATAAGAGCATAGATAGTGGAAGGCCAAGTTTAGCAATGCGTAGCAGTTCTTGTCGCTCAGTGGTACAAGAGCCTGATGTAGGAACGTCCGTTGCTGATAAAAATTATGATAACAACTGCTCCTTGGTGGTTTGTTCTAGTAGTGGTCTTGAAAGCCAAGGTTGGGAGTCGAGCACATCTACTTCTAATCAACAAACATTGGATCTGAATTTAGCTCGTGCATTTCAGGAAAAGTTAAATGACCCCAGAGTTACATCTATGTTGAAGAGAAGAGCAAGGCCAGGTGACCTAGAATTAGTTAGTTTATTGGAAGATAAAGGATTGGATCCTAATTTTGCCATGATGTTGAAAGAGAAGAGTTTGGACCCAACTATCTTGGCATTACTTCAGAGAAGTAGTTTGGATGCAGATAGAGATCATCGTGACAATACTGATATAACCATCATCGATTCAAATGGAGTTGACAATGCTTTGCCCAATCAAATTTCATTGTCAGAAGAACTGAGACTTCGGGGTTTAGAGAAGTGGCTTCAATTCTCCAGACTTGTTTTGCACCACATAGCAGGTACTCCAGAGCGAGCATGGGTTCTCTTTAGTTTTATCTTCATCCTTGAAACAGTCGTTGTGGCCATCGTCCGCCCAGAGACAATTAAGGTTATAAATGCTGCCCATCAGCAGTTCGAATTCGGTTTTGCTGTGCTGCTTTTGTCCCCTGTTATCTGTTCGATCGTTGCTTTTCTTTGCTCACTTAAAGCAGAAGAAATGACCATGACATCAAAGCCACGCAAGTACGGTTTTATAGCTTGGCTGCTGAGCACGTCTGTTGGTTTGCTGCTTTCCTTTTTGAGTAAATCGTCAGTCCTTCTTGGATTGTCCTTGACTGTCCCACTCATGCTGGCATGCCTCTCTGTTGCACTTCCTATATGGATCCGTAATGGCTATCAGTTTTGGGTTTCACGAGTAGAATGCGTAGGTCATGCTGGAAACCATCAAACTCCAAGGAAAACGGAGGGGCTTGTTCTGGTCATCTGTATATCAGTATTTGCTGGATCTGTATTAGCTTTGGGTGCGATAGTGTCTGCAAAGCCTTTAGATGATTTGGGATACAAGGGATGGACTGGTGAGCAGAGAAGTTTCACCTCTCCTTATGCATCATCTCTGTACATCGGCTGGGCGATGGCATCTTCAATTGCTTTAATAGTTACTGGTGTTTTGCCAATCGTTTCATGGTTTGCGACATATAGGTTCTCGCCCTCTTCGGCTATCTGTGTTGGAATATTTGCAGTTGTGCTTGTGACATTTTGTGGTGTCTCTTATTTGGAGGTTGTGAGTTCTAGGGATGACAAAGCTCCAACAAAGGGTGATTTCCTTGCTGCTTTACTTCCTTTAGTATTTATTCCGGCACTGCTCTTACTTTCCTCTGGTTTGCATAAATGGAGAGATGACAATTGGAAACTTTCTCGTGGTGTTTATGTATTTGTTATCATCGGTCTTCTTCTTCTGCTTGGCGCTATATCAGCCATTATAGTTATAGTTGATCCTTGGACGATTGGGGTCGCGTTTCTTTTAGTACTTCTCTTGATCGTACTAGCTATTGGCGTTATCAATTATTGGGCCTCAAACAATTTTTATTTGACCAGGACGCAGATGTTCTTTGTTTGTTTCCTTGCTTTTCTTTTGGCTTTGGCAGCATTTCTCGTTGGATGGTTTGAAGATAAACCCTTCGTTGGAGCATCTGTTGGTTTTTTTACCTTTCTCTTTCTTCTTGCCGGAAGAGCATTGACTGTGCTTCTTTCACCTCCTATTGTAGTTTATTCACCAAGAGTTCTGCCAGTATACGTTTATGATGCTCATGCAGATTGTGGGAAAAATGTCAGTGCTGCATTTCTTATGCTTTATGGAATTGGTTTGGCGATTGAGGGCTGGGGTGTTGTTGCAAGTTTGAAAATTTATCCACCGTTTGCTGGTGCTGCAGTATCGGCAATTACTCTTGTTGTAGCCTTCGGTTTTGCTGTATCTCGTCCATGCTTGACTCTTAAGACAATGGAAGACGCGGTTCACTTTCTCAGTAAGGAAACTGTGATACAAGCAATTGCTCGATCTGCCACTAAGACAAGGAATGCTCTATCTGGAACTTATTCGGCTCCCCAGAGATCTGCTAGTTCAGCTGCTCTTTTGGTTGGAGACCCTACGATCATGCGTGATAGGGCTGGAAATTTTGTGCTTCCTAGAGCAGATGTCATGAAATTGAGAGATCGTCTTAGAAATGAGGAATCAGCGGCAGGATCATTTCTCTGTCGCATGAGAAAAGGAAGGACATTTCGGCATGAAGTAACCAGTGAAGTAGGCCACAGAAGAGAAATGTGCGCGCATGCCCGAATATTGGCTTTGGAAGAGGCAATTGATACTGAGTGGGTATACATGTGGGATAAATTTGGTGGTTATTTACTTCTTTTGCTTGGTTTGACTGCTAAGGCGGAGCGGGTACAGGATGAGGTCCGCTTGAGACTTTTCCTTGATAGCATAGGGTTTTCCGACTTAAGTGCCAAAAAAATAAAAAAATGGATGCCAGAGGACCGTAGACAATTTGAGATTATTCAGGAAAGTTATATAAGGGAAAAGGAGATGGAGGAGGAAATTTTAATGCAGAGACGTGAAGAGGAGGGGAGGGGTAAAGAAAGAAGGAAGGTTCTCTTGGAGAAGGAAGAACGCAAATGGAAGGAGATAGAAGCTTCTCTCATTTCCTCTATTCCAAATGCTGGAAGCAGGGAGGCAGCAGCCATGGCAGCTGCAGTGCGTGCAGTAGGATGTGATTCTGTTCTCGATGATTCTTTTGCACGAGAGAGGGTATCAAGTATTGCACGTAGGATACGCACAGCCCAGTTAGCTCGACGCGCACTTCAGACGGGAGTAACAGGTGCTGTATGTGTTCTCGATGATGAACCAACAACAAGTGGCAGACATTGTGGTCAAATTGATTTCAATATATGTCAAAGTCAAAAGGTTAGCTTCTCTATTGCTGTAATGATCCAGCCCGAGTCTGGGCCGGTCTGTCTTTTAGGCACTGAATTCCAGAAGCAAGTGTGCTGGGAAATCCTGGTAGCTGGTTCAGAACAAGGCATAGAGGCTGGCCAAGTTGGCCTTAGACTGATCACTAAAGGTGATAGACAAACGACAGTAGCTAAGGAGTGGAGTATCAGTGCTACGAGTATTGCTGATGGAAGGTGGCATATTGTGACAATGACCATTGATGCTGATTTAGGTGAGGCAACTTGCTATATAGATGGTGGTTTTGATGGCTACCAGACTGGATTACCATTGCGTGTGGGAAACTACATTTGGGAACAAAGTACGGAAGTATGGGTTGGTGTTAGACCGCCTATAGATATGGATGCTTTTGGGAGGTCAGATAGTGAAGGCGCGGAGTCTAAGATGCATATAATGGACGTTTTCCTTTGGGGAAGGTGCTTGACTGAAGATGAGATTGCCGCTGTTCATGCTTCCATGGGATCATCTGAGTACAGTATGGTAGATTTTCCTGAAGATAATTGGCAATGGGCAGATTCACCTTCCAGAGTTGATGAGTGGGATAGTGATCCTGCAGACGTAGATCTATATGACAGGGATGAGGTTGACTGGGATGGGCAATATTCAAGTGGTAGGAAACGAAGGTCAGAACGCGAGGGGGTAGTGGTCGATATGGATTCTTTTGCCAGAAAGTTGAGGAAACCTAGGATGGAAACACGTGAAGAAATCAACCAACGAATGCTTTCAGTTGAATTGGCTGTCAAAGAAGCCCTATCTGCAAGGGGAGAAGCACATTTTACTGATCAGGAGTTCCCTCCTAATGATCAGTCATTATTTGTAGATACAGGAAACCCCCCGTCAAAATTGCAGGTTGTTTCTCAATGGATGAGGCCAACTGAGATGGTGAAAGAGAGCCATCAAGATTCTTGTCCGTGTTTGTTTTCTGGGGTTGCAAATCCTTCTGATGTTTGTCAGGGACGGCTGGGTGATTGTTGGTTTTTGAGTGCCGTTGCTGTTTTAACTGAGGTTTCTCGAATATCCGAAGTGATCATTACACCAGAATATAACGAGGAAGGAATTTACACTGTCCGCTTTTGTATTCAGGGTGAGTGGGTTCCTGTTGTTGTTGATGATTGGATCCCATGCGAATCACCCGGTAAACCAGCATTTGCTACCAGTAGGAAGGGGAACGAACTCTGGGTTTCTATATTGGAGAAGGCTTATGCTAAATTGCATGGATCTTACGAGGCATTAGAAGGTGGGCTCGTCCAAGATGCTCTTGTCGACCTCACTGGAGGAGCTGGTGAAGAGATCGACATGAGAAGTGCCCAGGCCCAGATTGATCTCGCAAGTGGTAGATTATGGTCTCAATTGTTGCGATTTAAGCAAGAAGGATTCTTACTCGGCGCTGGGAGTCCTTCTGGTTCTGATGTACATGTTTCTTCCAGTGGCATTGTCCAAGGGCATGCTTATTCAATATTGCAGGTAAGGGATATAGATGGGCACAGGCTTGTTCAGATTAGAAATCCATGGGCAAATGAAGTTGAATGGAATGGTCCTTGGTCTGACTCTTCACCTGAATGGACTGACAGAATGAAGCACAAGCTCAAACATGTTGCACAGTCAAAAGATGGTATATTCTGGATGTCTTGGCAAGATTTCCAAATCCACTTCCGTTCAATATACGTTTGTCGGATTTACCCGCCTGAGATGTGTTATTCCGTCCATGGCCAATGGCGGGGTTTCAGTGCTGGTGGTTGCCAAGATTATGACACATGGCATCAAAATCCACAGTTTCGATTGAGAGCCACCGGACCTGATGCATCATGTCCAATTCACGTCTTTATTACCTTAACTCAGGGTGTGAGTTTCTCGAGAACAACAGCTGGTTTCCGAAATTATCAATCGAGTCATGATTCGATGATGTTTTATATCGGGATGAGGATACTCAAAACATGTGGTCGTCGTGCTGCTTATAACATTTACATGCATGAATCGGTTGGAGGGACGGATTATGTGAATTCTCGGGAAATATCGTGTGAAATGGTTCTCGAGCCTGATCCAAAGGGTTATACAATTGTTCCTACAACTATTCATCCTGGGGAAGAAGCACCTTTTGTTCTCTCTGTTTTCACCAAGGCACCTATAGCCCTTGAAGCTTTATAGCATCATGCAATGGTTTTGTTTCGTCATTTGGTACCCAATTTTTGGACCGCTTGGGTTGTCCATCGAGTTATTGAACCGCGAATCGGTGCACATAGTGGCTTTGGTGGTTCTGATTGAGCTGGCCTTGGGTTTTTTATCAACTCAATGGATGGCCAAAGGTCAAAGGAAATGATGATCATGAGCTGTTTGGTATTTTGACCAAAAGGGGGTTCTCGGAGGGTGAAGTGTACATGAGTAGGTAATTGTAACCATATAGGCGTGTGGATCAATGTAAATGTGTGGGGGAAAAAAAAAAAAAAAAAAAAAAAAAAAAAAAAAAAAGT

>transcript_18 full_length_coverage=2;length=7123

GGGGAGTTTCTCTCGCCTCTCTCTTAAAACACATATACACACTCTCTCTCTAAAAGCGTCAACTCCGTCGGATCTGCAGTCTGTACGAGTTATTTCGAAGCAGATCAACTGAATTTCAACCCACTGTGATCTAGGGTTCCAAAAGACGTCGTCTTTCATGAAGAATTGATGCTTTCACTTAGCTCCAAATGTTCAAGACTTCCTCACATGACTTTGTTCTTCTGGTCTACCAAACACCAATTTTCGCTCTTTATACTCGCATCAGCTTAAGGGTTTGGTCAATTTCATAAACCATTAAACCCTTTGGGGTTTCTGGTTTTGTATGCAGGAGCCAAGTGATTAAATCTAGGGTTTGCTGCCATAGAGTAAAATTGGGGTTATATTAAGCTTTAGTTATAGGTTTGCTGGTATAGAGTAAATTTGGGGTTATATTGAGCTTTAGTTTATATAGTTTTGGGAGGTAGAATTGTAAAGCTAGGTTTTTTTGGAGAGCTTTTGTTGAGATTTTGGATCAAGTAAGGTAGAAGATAGAGAAACATGTTTTATTCTCAATTTATATTGGCAAAAAAAGGGCCACTAGGGACGATATGGATAGCTGCACATTTGGAGAGAAAGCTTCGCAAGAATCAGGTTGCAGATACCGATATTGGCGTGTCCGTAGATTCAATTCTTTCCCCTGAAGTACCCATTGCACTCCGGTTGTCCAGTCATCTTCTGCTTGGTGTAGTCAGGATATATTCTAGAAAGGTTAATTACCTTTTTGATGACTGCAGTGAGGCTTTGCTTAAGGTGAAGCAAGCTTTTCGCTCCACTGCAGTTGACTTACCTCCAGAAGAATCCACTGCACCATATCACTCTATCACCTTGCCTGAGACTTTTGATCTTGATGATTTTGAGCTGCCAGATAATGAAAATTTTCAGGGCAACTATGTTGATCACCACATCAGTACAAAGGAGCAGATTACGCTCCAAGATACTATGGAGGGTGTTGTTTTTTCAACATCTCAGTTTGGACTGGATGAGCAATTTGGCGATGGTGACGCTTCTCAAATCAGTTTCGACCTTGATGAGGACTTGTTTCTTGACAAGGTTGCAGCTCCAAGACATACCGGAGTTATGTTGGATTCAAAGGATGATCCCCAGGCATCTGTACAGCCAATGACATCCCTTGACAATTATGTAAATAACGCGTGGACGATTGATACCTCAGAAGCCATGCCAGTGAATGGTCGTGGAGAACAGCATAAAGGCCTAGTTGCAAATAATGATATCATCGAGTCTGCCCAGGCCCCATCTACTCCTGGATTAATTGAAGAACCAAATTTATCCAATATGCAAATGGGCCTAGCTTGCGATGATCATATGGAGTTAGAAAACTACAATTCAATGGAATTTGAAGCGAGAGAAGCCATAGGAAATTCTTTCAGTAAATCAGATCATCACGGGGATAAAAAATGTGTGGATGGGCCTTTGTCCAGTATCGATATGAATATTGATTGCATGCCTGTTCAGGAAAATGATCATGTTATGGGCGAGCCCGTGATCTCACAAGCCAACCCACAAGGAGATTTACCGTCTACGTCTGAAAATGCGGGGAAAATTGAAGTGCAAAACTTCTCCAATATACAAATGACTATGGCTTGCAATGATCTTATTGAGTCAGAAGATCATAATTCAATAGAACTCGAAGTGAGAGAGACCGCAGGAAGTTCTTCAAGTAAATTAGATCATCACGGGCATAAAAATTCTGCAGAGTGGGCTTCGTTCAATATAGATATGAATCTTGATTGCATGCCTGTTCAGGAGAATGATCATGTTATGGGTGAGCAGGAGATCTTACAAGCCGAGCCACAAGTAGATATACCATCTACTTCTGATACTATGGAGAACATGTTGGATAATCCTCTTTTAGCATCTTTACCATCTCCGGTTCTAACTGAGAAAGAAAATTCTGTTTCTGTAGTGTCAGAGTTTCAGGGTAGGATTACTGCACCTGATGACATGGACAGTGTGGGGGATTTACAAAATGGAGTTCTGAGCAAAACCGAACAAAGTACAACGTTTACGGGTCAAGACCATTTGGAATGCACGGAATCCTTAGGAGTTAGATTGGAGGAAACTGTAGCCTCTCCTAGTTTTTCCCTTGGGGCCTCTGTTTTGGAGGATGCTCGTCATAAACCAGGTTCTGGTGTTGGACCAATTTCTGAGAAGTCCAGTTTAACCAATACCTATCCTCTAGTACCTGGAGGTATTTCTGGGAGTGATTCAGCATTGCTTCAGCCAGAGATTTCTGATAGTGTAGAGATAGCTGGAAACATGGAAACATGCCCCCCTAATGCACTGGTATCTGTCTGTTCTTTGAAGTCTCCTACTGGACCCGGGCTTGAGAATGTCGAGGCTTCGGCTGCTCATGAACCAAAACATTCTGAGACTTCGAATCCCAACCAAGAAGGGCCATCTACTTGCATCCATGTACTTCAGCAAGCATGCAACTCACTTGAAAACGAACCTGTTAAATCATTTTTTGGAGGTAACAATTCTATAGTTGTTCCTGACATACCCTCTGAAGTTTTATTGGAGGGTCAGGCTGGTCATAAACCAAAAGATCCTGAGATTATGAACCCTGATGAGGAAGGGCCATCTACTCGCATCCATGTACTTCAGGCTTGCAATTCAATTCAAAACCAACCTGTTAAATCATTTCTTGGAGGTTACAGTTCTGTAGTTCCTGACTTACCTGCTGAGACATCTGAATGGGAGGCAACCCATATTTTGGGAGCTTCAACTGAGGTGCAAGCAGCGAATTTTGATGGTACACCTGGGCACCAGCCAGCACCAGGGCAAGCTGCGCTACCAGCCAGTGGCCATCAGCATGAGGCCAGCACCGCAAGGGTTCAGCAGCAGTTGCCACAGGAGCCCATAGCCACCGTCAGCTGGCGCCCTACAGCAAATTTTGATGGTACCCCTGGGCACCAGCCAGCACCAGGGCAAGCTGTGCTACCAACCAGTGATCAGCACCACGAGGCCAGCACCACAAGGCTCCCGCAGCAGTTGCCACAGGAGCCCATAGCCGCCGCCAGCTGGCACCCTACAGAGAATTTTTTTGACGGGAGCACTGGCCATCAGTCAGCACCATGGCAAGCTGCGGAACCAGCCAGCAACCAGCAACACCAGGTGGGCAGCACAGGGCACCAGCAACAGCAGGACACCAGTAGCACCAGGTTGCAGGTGCCAACAGCCACCACCAGCTCCCCCACAACAGATGTAGATAATTTATTTAAGGGGACCACTGGCCATCAGTCAGCACCATGGAAAGCTGCGGTACCAGTCAGCGATCAGCAACACCAGGATGACAACACAAGGCACCCCCAGCAGCAGCTGTTGCAGGACATCAGTAGCACCAGGTCACAGGCGCCAACAGCCACCACCAGCTCCCCCACAACAGATTTTTTTGATAGCACTACCGAGCACCAACCAGTATCATGGCAAGCCGTGGCATCACCCAGTGACCACCAACACCAGGTGGACACTGCAAGGCACCTGCACCAGGGCAGCACCAGGCTGCAGGCGGCCACAATCACCACCAGCTGTCCCCCGGCAGTGACTTTTTTTGACGGTTCCACTGGGCACCACATGGCACCATGCCAAGCTGCGGTACTAGCTGGCAACCAGCAACACCAGGACGACACTGCAAGGCAGCTGGGACAGAGGTCGCAGGACACCAGCAGCACAAGGCCTCAGGCGCCCTCAACCACCACCACCAGCTGCTCCCCAACAGCAATCTTTTTTAATGGCTCCACTGATCACCAACCGACACCATGGAAAGCCGCAGCACCAGCCAGCGACCAGCAACACCAGGCTGACACCGCAAGCCACCTGCACCAGCAGTCACTGGACACCATCAGCACCAGGCTGCAGGTGCCCACGACCACCACCAGTTGGCCCCCAACAGTGAATTTTTTTGATGGCACCACTGGGCACCAACTGGCCCCATGGCAAGCTGCGGTACCAGCCAGCGACCAGCAGCACCAGGCCGACACCCCAATGCACCTGCAGCCGCAGTCGCAGGACACCGGCAGCACCGGTTTGCAAGCTCGCACAATCACCACCAACTGCCCCCCAACAGCAAATATTTTTGATGCCACTGCTGGGAACCAGCCAGCACCATGGCAAGCTGTGGTACAAGCCAGTGACCAGCAGTGCCAGACCGACTCTCCAAAGTACCAGCTGCAGACTCCTGAGGACACCAGCAGCACCAGGCCGCAGGTGCCCACAACTACCACCAGCTGGCGCCAAACAGGAGAAGGCTACCGTGCAACTGATATGGGCCAACCGTCCTTAGACGTGAACCAGTTATTGGAACCAAATTCTTGTCAGTATGTTGACGCAGGTCGCAGGAAGTTGGATGAGCAACTAGCGAATGCCAAAAATACTGTTGAGAATTATTTTGGTTATGCCGATTTGCCTGCACCTGAAAAAATGCTTTCTGTTCCAGAAGGGCGTAGCCATCTAGGGAATAATTTTTTGTTGGAGTCTACTCCGGGCAAAGAAGCCACAATGCATGGCGAAGGAAATGGCACTGGGGTCCAAAACATTACTGGAAATAAGCGTAGTTATATGGAAAGTATTCTGACTACGCAGAGCCTGAATTCGGTTGAACCATTTGAGAAGTCCCGATCTGAAAGAACTACGGGATCTGTTCCTGATGATGATGATTTGTTATCTTCCATCCTAGTTGGAAGAAGGTCTTCAATTTTGAAAATGAAGCCCACTCCACCTCCATCTGAAACAGTGTCTGTGAAACGCCGTCGAACTGCACCCCGGGCCAGTGCATCTAAGAGGAAGGTGTTTATGGATGATACAATGGTCTTGCATGGCGATATAATACGACAACATTTGACAAATACTGAAGACATACGTAGAGTACGGAAGAAGGCTCCTTGCACTCGACCTGAAATTTGGATGATTCAGAATCAATTCTTGGAGGATGAAATTTTCGGTGATCCCATATTTAGTGGTATGTCGTCAGAATTGATTTGTTCACTCGGTCAAAGATATGATCCGAGTATAATTAGGGTTTCTCAGAATGATGAAAGTATAGCTTTGTTTGGAGCAGCAAAGAATGGAGAGCTTTCTATGACACCAAATGTTATCAATGAAACTGAACCAGAAAGGATCATTGAATCTGAAATAACCAGAAATGACGGGGAAGTTCAATCTGCCGAGACTGTTGTCCAAACTGAGGTTCAGCATCTTGATAATGATACTCAAATGCAATTGAATGCTAATACTACGATGCCTGAGCTTAGGACTTTGCACCCGGAAACAGATGGGATCACTGAACCTATAATAGTCAGAAATGACGGGGAAGCCCAATCTGCCATGGCTTTAGTTCAGACCAAGGACCAGCATAATTATATTGCTACTCAAATACAAATCAATTCTAATACTATAATACCTGAGCTCAAGTCTTCACACCCTGAAACGGAAGGGATCACTGAACCTATAATAGCCAGAAATGACGGTGAAGCCCAACCTGCTAATACTTTTGTCCAGACGGCAGAGCTGCATAATTACAATGATACTCAAATGCAAATGAATGCTAATACTGTGATACCTGAACTTGGCACTTTGCAACCCGAACATATGGGCAAGATAGCTGAAATGGAATTTGCTAGAGGTGGTTCTGCAGTTGCTAATGCAGAGGAGACTTCTGTCGCTCTTGGGATTGGATTATCATCCCCAGTTGATTTAGTTTCTGTAGATATTTGTAACTTGTCAACTGATCCTGTGGAGAAACCCAGTGGTTCTCCACAGATCGCTGTGGCTTGCTTGGTTCCCGACCAGAAGTTGGATTCTCAATCTGTTGAGAAGGATGCTTCTGTAGTGGATTTATGCATTGAGAAAGCAGTTGAGCCTGTTGAAGTTTCTGAAATTCAAGTCGTAGTTGGTACTGAATCTTGTGCAAGTTTTGAGAAAGATGCTTCTGCAGTAAATCTTGGGCAAGGAGTTGAGTTTATTGAAGTTCCAGAACTTCATGACGGCGATATTATTGGAGCTGGGACTGAATCTGGTGCGACAGGTGAGTTTCTTTTGGAAGAAAACAGAGGCGGTCTTTCAGTTGAAAACAGAACAGATAATCCTACCGACAGTGTAGATCTTCCCCTTGAAACCGTCTCTCCAGCAATTGACTTGGTTGTTGTGACTGAAGACCAAGCTGAGGAGGAAACTGCTAAAATCGAAGAAGGGGTTGTAAATGAGGCTACTGAAAAGGGGTATGATGGAAGGGATCGAACCTCTTACGGTGTATATAGCCAAGAACCTAAAACTGATTATTCCCTGAGTGACGGACAAGATGCATCCTTGAATAACGTTGAAAAATCTGGTGGCCAGGAGGTTGTACCACAAGATGTAAGAGATGCCGAAACTGCTTCCTTCGACCATGCTGATGTTGAAATCCATGATGATGTGGAAAATGAAACAGTTGAGCATGATACTGAGTTCCTCAACTTTGATAACGACGGTGATGATGATGGCGGAGCTGACGATGACTATGATGACTGTATGCCTTGTCCAGAAGAAACCCGCATTCTTGAGAACAGCGGATGGTCTTCCCGTACCAGGGCTGTTGCCAAGTACCTCCAAACCGTATTCGACAAAGAAGCTGACCACGGTAAAAAAGTTATTCCATTGGACAACCTTTTGAGTGGCAAGACTCGAAAGGAAGCGTCGAGGATGTTTTTTGAAAGTTTGGTTCTTAAAACAAGGGATTATATACATGTAGAACAGGAAAACCCTTTTGATAACATTATTATAAAGCCTCGAGTAAAGCTCTTGAAATCGGAGTTCTGATATATCCTACATAATTATCCGCCCGAGTTTGGGTATTCTTCTTCATTTTGGTGTGTATTTTTGTTTATTGTTTTGTTTAGTTGTTGTAACTATTAGTTAAAAGTGGGGTCATGTTTTATCCCCAGTTGTTGTAACAGTGTTGTAACATTAGGCTGCATTTATATGTGTACATATATGTAACTGTAATAATGTGATTCACCATTTGCAATTAAAGATGCTAATCAATTTATTTGTTAAAAAAAAAAAAAAAAAAAAAAAAAAAAAAAAAAGT

>transcript_19 full_length_coverage=18;length=7086

GGGGAGTTTCTCTCGCCTCTCTCTTAAAACACATATACACACTCTCTCTCTAAAAGCGTCAACTCCGTCGGATCTGCAGTCTGTACGAGTTATTTCGAAGCAGATCAACTGAATTTCAACCCACTGTGATCTAGGGTTCCAAAAGACGTCGTCTTTCATGAAGAATTGATGCTTTCACTTAGCTCCAAATGTTCAAGACTTCCTCACATGACTTTGTTCTTCTGGTCTACCAAACACCAATTTTCGCTCTTTATACTCGCATCAGCTTAAGGGTTTGGTCAATTTCATAAACCATTAAACCCTTTGGGGTTTCTGGTTTTGTATGCAGGAGCCAAGTGATTAAATCTAGGGTTTGCTGCCATAGAGTAAAATTGGGGTTATATTAAGCTTTAGTTATAGGTTTGCTGGTATAGAGTAAATTTGGGGTTATATTGAGCTTTAGTTTATATAGTTTTGGGAGGTAGAATTGTAAAGCTAGGTTTTTTTGGAGAGCTTTTGTTGAGATTTTGGATCAAGTAAGGTAGAAGATAGAGAAACATGTTTTATTCTCAATTTATATTGGCAAAAAAAGGGCCACTAGGGACGATATGGATAGCTGCACATTTGGAGAGAAAGCTTCGCAAGAATCAGGTTGCAGATACCGATATTGGCGTGTCCGTAGATTCAATTCTTTCCCCTGAAGTACCCATTGCACTCCGGTTGTCCAGTCATCTTCTGCTTGGTGTAGTCAGGATATATTCTAGAAAGGTTAATTACCTTTTTGATGACTGCAGTGAGGCTTTGCTTAAGGTGAAGCAAGCTTTTCGCTCCACTGCAGTTGACTTACCTCCAGAAGAATCCACTGCACCATATCACTCTATCACCTTGCCTGAGACTTTTGATCTTGATGATTTTGAGCTGCCAGATAATGAAAATTTTCAGGGCAACTATGTTGATCACCACATCAGTACAAAGGAGCAGATTACGCTCCAAGATACTATGGAGGGTGTTGTTTTTTCAACATCTCAGTTTGGACTGGATGAGCAATTTGGCGATGGTGACGCTTCTCAAATCAGTTTCGACCTTGATGAGGACTTGTTTCTTGACAAGGTTGCAGCTCCAAGACATACCGGAGTTATGTTGGATTCAAAGGATGATCCCCAGGCATCTGTACAGCCAATGACATCCCTTGACAATTATGTAAATAACGCGTGGACGATTGATACCTCAGAAGCCATGCCAGTGAATGGTCGTGGAGAACAGCATAAAGGCCTAGTTGCAAATAATGATATCATCGAGTCTGCCCAGGCCCCATCTACTCCTGGATTAATTGAAGAGCCAAATTTATCCAATATGCAAATGGGCCTAGCTTGCGATGATCATATGGAGTTAGAAAACTACAATTCAATGGAATTTGAAGCGAGAGAAGCCATAGGAAATTCTTTCAGTAAATCAGATCATCACGGGGATAAAAAATGTGTGGATGGGCCTTTGTCCAGTATCGATATGAATAATGATTGCATGCCTGTTCAGGAAAATGATCATGTTATGGGCGAGCCCGTGATCTCACAAGCCAACCCACAAGGAGATTTACCGTCTACGTCTGAAAATGCGGGGAAAATTGAAGTGCAAAACTTCTCCAATATACAAATGACTATGGCTTGCAATGATCTTATTGAGTCAGAAGATCATAATTCAATAGAACTCGAAGTGAGAGAGACCGCAGGAAGTTCTTCAAGTAAATTAGATCATCACGGGCATAAAAATTCTGCAGAGTGGGCTTCGTTCAATATAGATATGAATCTTGATTGCATGCCTGTTCAGGAGAATGATCATGTTATGGGTGAGCAGGAGATCTTACAAGCCGAGCCACAAGTAGATATACCATCTACTTCTGATACTATGGAGAACATGTTGGATAATCCTCTTTTAGCATCTTTACCATCTCCGGTTCTAACTGAGAAAGAAAATTCTGTTTCTGTAGTGTCAGAGTTTCAGGGTAGGATTACTGCACCTGATGACATGGACAGTGTGGGGGATTTACAAAATGGAGTTCTGAGCAAAACCGAACAAAGTACAACGTTTACGGGTCAAGACCATTTGGAATGCACGGAATCCTTAGGAGTTAGATTGGAGGAAACTGTAGCCTCTCCTAGTTTTTCCCTTGGGACCTCTGTTTTGGAGGATGCTCGTCATAAACCAGGTTCTGGTGTTGGACCAATTTCTGAGAAGTCCAGTTTAACCAATACCTATCCTCTAGTACCTGGAGGTATTTCTGGGAGTGATTCAGCATTGCTTCAGCCAGAGATTTCTGATAGTGTAGAGATAGCTGGAAACATGGAAACATGCCCCCCTAATGCACTGGTATCTGTCTGTTCTTTGAAGTCTCCTACTGGACCCGGGCTTGAGAATGTCGAGGCTTCGGCTGCTCATGAACCAAAACATTCTGAGACTTCGAATCCCAACCAAGAAGGGCCATCTACTTGCATCCATGTACTTCAGCAAGCATGCAACTCACTTGAAAACGAACCTGTTAAATCATTTTTTGGAGGTAACAATTCTATAGTTGTTCCTGACATACCCTCTGAAGTTTTATTGGAGGGTCAGGCTGGTCATAAACCAAAAGATCCTGAGATTATGAACCCTGATGAGGAAGGGCCATCTACTCGCATCCATGTACTTCAGGCTTGCAATTCAATTCAAAACCAACCTGTTAAATCATTTCTTGGAGGTTACAGTTCTGTAGTTCCTGACTTACCTGCTGAGACATCTGAATGGGAGGCAACCCATATTTTGGGAGCTTCAACTGAGGTGCAAGCAGCGAATTTTGATGGTACACCTGGGCACCAGCCAGCACCAGGGCAAGCTGCGCTACCAGCCAGTGGCCATCAGCATGAGGCCAGCACCGCAAGGGTTCAGCAGCAGTTGCCACAGGAGCCCATAGCCACCGTCAGCTGGCGCCCTACAGCAAATTTTGATGGTACCCCTGGGCACCAGCCAGCACCAGGGCAAGCTGTGCTACCAGCCAGTGATCAGCACCACGAGGCCAGCACCACAAGGCTCCCGCAGCAGTTGCCACAGGAGCCCATAGCCGCCGCCAGCTGGCACCCTACAGAGAATTTTTTTGACGGGAGCACTGGCCATCAGTCAGCACCATGGCAAGCTGCGGAACCAGCCAGCAACCAGCAACACCAGGACACCAGTAGCACCAGGTTGCAGGTGCCAACAGCCACCACCAGCTCCCCCACAACAGATGTAGATAATTTATTTAAGGGGACCACTGGCCATCAGTCAGCACCATGGAAAGCTGCGGTACCAGTCAGCGATCAGCAACACCAGGATGACAACACAAGGCACCCCCAGCAGCAGCTGTTGCAGGACATCAGTAGCACCAGGTCACAGGCGCCAACAGCCACCACCAGCTCCCCCACAACAGATTTTTTTGATAGCACTACCGAGCACCAACCAGTATCATGGCAAGCCGTGGCATCACCCAGTGACCACCAACACCAGGTGGACACTGCAAGGCACCTGCACCAGGGCAGCACCAGGCTGCAGGCGGCCACAATCACCACCAGCTGTCCCCCGGCAGTGACTTTTTTTGACGGTTCCACTGGGCACCACATGGCACCATGCCAAGCTGCGGTACTAGCTGGCAACCAGCAACACCAGGACGACACTGCAAGGCAGCTGGGACAGAGGTCGCAGGACACCAGCAGCACAAGGCCTCAGGCGCCCTCAACCACCACCACCAGCTGCTCCCCAACAGCAATCTTTTTTAATGGCTCCACTGATCACCAACCGACACCATGGAAAGCCGCAGCACCAGCCAGCGACCAGCAACACCAGGCTGACACCGCAAGCCACCTGCACCAGCAGTCACTGGACACCATCAGCACCAGGCTGCAGGTGCCCACGACCACCACCAGTTGGCCCCCAACAGTGAATTTTTTTGATGGCACCACTGGGCACCAACTGGCCCCATGGCAAGCTGCGGTACCAGCCAGCGACCAGCAGCACCAGGCCGACACCCCAATGCACCTGCAGTCGCAGTCGCAGGACACCGGCAGCACCGGTTTGCAAGCTCGCACAATCACCACCAACTGCCCCCCAACAGCAAATATTTTTGATGCCACTGCTGGGAACCAGCCAGCACCATGGCAAGCTGTGGTACAAGCCAGTGACCAGCAGTGCCAGACCGGCTCTCCAAAGTACCAGCTGCAGACTCCTGAGGACACCAGCAGCACCAGGCCGCAGGTGCCCACAACTACCACCAGCTGGCGCCAAACAGGAGAAGGCTACCGTGCAACTGATATGGGCCAACCGTCCTTAGACGTGAACCAGTTATTGGAACCAAATTCTTGTCAGTATGTTGACGCAGGTCGCAGGAAGTTGGATGAGCAACTAGCGAATGCCAAAAATACTGTTGAGAATTATTTTGGTTATGCCGATTTGCCTGCACCTGAAAAAATGCTTTCTGTTCCAGAAGGGCGTAGCCATCTAGGGAATAATTTTTTGTTGGAGTCTACTCCGGGCAAAGAAGCCACAATGCATGGCGAAGGAAATGGCACTGGGGTCCAAAACATTACTGGAAATAAGCGTAGTTATATGGAAAGTATTCTGACTACGCAGAGCCTGAATTCGGTTGAACCATTTGAGAAGTCCCGATCTGAAAGATCTACGGGATCTGTTCCTGATGATGATGATTTGTTATCTTCCATCCTAGTTGGAAGAAGGTCTTCAATTTTGAAAATGAAGCCCACTCCACCTCCATCTGAAACAGTGTCTGTGAAACGCCGTCGAACTGCACCCCGGGCCAGTGCATCTAAGAGGAAGGTGTTTATGGATGATACAATGGTCTTGCATGGCGATATAATACGACAACATTTGACAAATACTGAAGACATACGTAGAGTACGGAAGAAGGCTCCTTGCACTCGACCTGAAATTTGGATGATTCAGAATCAATTCTTGGAGGATGAAATTTTCGGTGATCCCATATTTAGTGGTATGTCGTCAGAATTGATTTGTTCACTCGGTCAAAGATATGATCCGAGTATAATTAGGGTTTCTCAGAATGATGAAAGTATAGCTTTGTTTGGAGCAGCAAAGAATGGAGAGCTTTCTATGACACCAAATGTTATCAATGAAACTGAACCAGAAAGGATCATTGAATCTGAAATAACCAGAAATGACGGGGAAGTTCAATCTGCCGAGACTGTTGTCCAAACTGAGGTTCAGCATCTTGATAATGATACTCAAATGCAATTGAATGCTAATACTACGATGCCTGAGCTTAGGACTTTGCACCCGGAAACAGATGGGATCACTGAACCTATAATAGTCAGAAATGACGGGGAAGCCCAACCTGCCATGGCTTTAGTTCAGACCAAGGACCAGCATAATTATATTGCTACTCAAATACAAATCAATTCTAATACTATAATACCTGAGCTCAAGTCTTCACACCCTGAAACGGAAGGGATCACTGAACCTATAATAGTCAGAAATGACGGTGAAGCCCAACCTGCTAATACTTTTGTCCAGACGGCAGAGCTGCATAATTACAATGATACTCAAATGCAAATGAATGCTAATACTGTGATACCTGAACTTGGCACTTTGCAACCCGAACATATGGGCAAGATAGCTGAAATGGAATTTGCTAGAGGTGGTTCTGCAGTTGCTAATGCAGAGGAGACTTCTGTCGCTCTTGGGATTGGATTATCATCCCCAGTTGATTTAGTTTCTGTAGATATTTGTAACTTGTCAACTGATCCTGTGGAGAAACCCAGTGGTTCTCCACAGATCGCTGTGGCTTGCTTGGTTCCTGACCAGAAGTTGGATTCTCAATCTGTTGAGAAGGATGCTTCTGTAGTGGATTTATGCATTGAGAAAGGAGTTGAGCCTGTTGAAGTTTCTGAAATTCAAGTCGTAGTTGGTACTGAATCTTGTGCAAGTTTTGAGAAAGATGCTTCTGCAGTAAATCTTGGGCAAGGAGTTGAGTTTATTGAAGTTCCAGAACTTCATGACGGCGATATTATTGGAGCTGGGACTGAATCTGGTGCGACAGGTGAGTTTCTTTTGGAAGAAAACAGAGGCGGTCTTTCAGTTGAAAACAGAACAGATAATCCTACCGACAGTGTAGATCTTCCCCTTGAAACCGTCTCTCCAGCAATTGACTTGGTTGTTGTGACTGAAGACCAAGCTGAGGAGGAAACTGCTAAAATCGAAGAAGGGGTTGTAAATGAGGCTACTGAAAAGGGGTATGATGGCAGGGATCGAACCTCTTACGGTGTATATAGCCAAGAACCTAAAACTGATTATTCCCTGAGTGACGGACAAGATGCATCCTTGAATAACGTTGAAAAATCTGGTGGCCAGGAGGTTGTACCACAAGATGTAAGAGATGCCGAAACTGCTTCCTTCGACCATGCTGATGTTGAAATCCATGATGATGTGGAAAATGAAACAGTTGAGCATGATACTGAGTTCCTCAACTTTGATAACGACGGTGATGATGATGGCGGAGCTGACGATGACTATGATGACTGTATGCCTTGTCCAGAAGAAACCCGCATTCTTGAGAACAGCGGATGGTCTTCCCGTACCAGGGCTGTTGCCAAGTACCTCCAAACCGTATTCGACAAAGAAGCTGACCACGGTAAAAAAGTTATTCCATTGGACAACCTTTTGAGTGGCAAGACTCGAAAGGAAGCGTCGAGGATGTTTTTTGAAAGTTTGGTTCTTAAAACAAGGGATTATATACATGTAGAACAGGAAAACCCTTTTGATAACATTATTATAAAGCCTCGAGTAAAGCTCTTGAAATCGGAGTTCTGATATATCCTACATAATTATCCGCCCGAGTTTGGGTATTCTTCTTCATTTTGGTGTGTATTTTTGTTTATTGTTTTGTTTAGTTGTTGTAACTATTAGTTAAAAGTGGGGTCATGTTTTATCCCCAGTTGTTGTAACAGTGTTGTAACATTAGGCTGCATTTATATGTGTACATATATGTAACTGTAATAATGTGATTCACCATTTGCAATTAAAGATGCTAATCAATTTATTTGTTAAAAAAAAAAAAAAAAAAAAAAAAAAAAA

>transcript_20 full_length_coverage=2;length=7035

GGATGCTGCGAAACTGGCACCAGCCTGGTAGTGATGGTTCTACATCAAAATCAAGTGGAACTATAGACACACATGATAAAAGTGTTGTCCACATTCCATCCTCCATTTCACTTGCTGCTTCATCTAACTTGAATGATCAAGAGAAAAATGACTTTGCATCCCAGCTGCTTCGAGCTTGCAGTTCTCTTAGACAGCAAGCTTTTGTCAATTATCTGATGGATATCCTGCAGCAATTAGTGCATGTGTTTAAATCCCCATCTGCTAATTTTGAAACTTCACATGGTTTGAACCCTGGATCTGGATGTGGAGCTTTACTAACTATACGAAGAGAGCTACCTGCGGGTAATTTCTCTCCATTCTTTTCAGATTCATATGCGAAGTCTCACCGTGCAGATATATTCATGGACTACCATAGGCTACTGTTGGAGAATGCTTTCCGACTGGTTTACAGTTTGGTTCGACCAGAAAAGCAAGACAAGAGTGGGGAGAAGGAGAAGGAGAAGGAGAAGGCTCATAAAATTTCTTCTGGAAAGGATTTGAAGTTAGATGGGTATCAAGATGTTCTATGCAGTTACATCAACAATCCTCACACTACATTTGTAAGAAGGTATGCGAGGAGGCTTTTTCTGCATCTCTGCGGTAGCAAAACCCAATATTATAGTGTTCGAGACTCCTGGCAGTTCTCAAGTGAAGTTAAGAAACTTTACAAACATGTGAACAAGTCTGGTGCCTTCCAGAATCCTGCCTCGTATGAGAGAAGCGTTAAGATAGTGAAGAGCCTGACTACTATGGCTGAAGTTGCTGCTGCACGGCCTCGGAACTGGCAAAAGTACTGTTTGAGGCATGGAGATGTTTTGCCCTTTTTAATGAATGGAGTATTCCGTCTTGGGGAAGAATCTGTTATTCAGACTCTTAAGCTTTTAAATTTGGCCTTCTATACAGGAAAGGATATGAGCCACTCTTTACAGAAAGCTGAAGCAGGTGAGACTGGAACAAGTTCAATTAAATTGGGCACACATTCACTGGATTCAAAGAAGAAGAAGAAAGGCGAGGATGGAACTGAATCTGGTTTGGAGAAGCCCTATTTGGATATGGAGCCAGTGGTAGATATCTTCACTGACAAGGGTGGTGATGTTTTGAGGCAATTCATTGATTGTTTTTTATTGGAATGGAATTCAAGCTCTGTTCGGGTGGAGGCTAAGTGTGTTCTTTATGGTGTCTGGCATCACGCAAAGCTGCCATTCAAAGATACCATGTTTACGACTCTCCTGCAGAAAGTGAAATGTCTTCCAATGTATGGTCAAAATATTATTGAGTACACTGAACTTCTGATTTGGTTGTTAGGGAAGGCCCCAGATAGTAGTTCAAAGCAGCAGAGCACTGAAATTGTGGATCGATGCTTGACCCCTGATGTGATTAAGTGCTTTTTTGAGACACTTCACTCACAAAATGAGCTTTTAGCTAACCATCCCAATTCTCGCATATACAATACTTTAAGTGGCCTTGTGGAATTTGATGGTTACTATTTGGAAAGTGAGCCTTGTGTTGCCTGTAGCTCTCCCGAGGTACCCTACAGCAGGATGAAGCTAGAAAGTCTAAAATCGGAGACTAAGTTCACTGACAATCGCATCATTGTGAAATGCACTGGGAGCTATACCATCCAGACTGTGACCATGAATGTTCATGATGCTCGGAAGTCCAAATCAGTGAAAGTTTTGAACCTTTATTACAACAATAGGCCCGTGGCTGACTTGTCAGAGCTGAAAAATAATTGGTCCTTGTGGAAGCGTGCAAAGAGTTGTCATCTTGCTTTTAATCAGACTGAGCTAAAAGTAGAGTTTCCTATTCCCATTACTGCATGCAACTTCATGATTGAGCTGGATTCTTTTTATGAAAATCTACAGGCTTTATCCCTTGAACCATTGCAATGTCCACGTTGCAGTCGACCTGTCACCGATAAGCATGGGATTTGTGGCAATTGCCATGAAAATGCATATCAATGCCGGCAATGCCGCAACATTAATTATGAGAATCTGGACTCTTTTCTTTGCAATGAGTGTGGGTATAGCAAATATGGTCGTTTTGAATTTAACTTCATGGCAAGGCCAAGTTTTACATTCGATAATATGGAGAATGAAGATGATATGAAAAGGGGATTGGCGGCTATAGAATCTGAATCCGAAAATGCTCATAGAAGATATCAGCAACTTCTGGGCTTCAAGAAGCCCTTGCTCAAGATTGTATCCAGCGTTGGTGAGAATGAAATGGATTCACAACAGAAGGATTCTGTCCAGCAAATGATGGTCTCTTTGCCTGGACCTTCGTGTAAGATAAATCGTAAAATTGCTCTTCTTGGCGTGCTATATGGTGAGAAATGTAAAGCAGCTTTTGATTCCGTCAGCAAGAGTGTTCAGACGCTGCAAGGGCTTCGTCGTGTGTTGATGAATTACTTGCACCAGAAACAGTCGGGTAATGCAGTGGTGTCATCAAGATTTGTAGTTTCCAGGACACCAAATAGTTGCTACGGCTGTGCCACTATTTTCGTAACACAATGTCTTGAGATATTACAGGTGCTATCAAGGAACCCAAATTCTAAGAAGCAACTTGTTGCTGCCGGTATTTTATCTGAGTTGTTTGAAAATAATATTCATCAAGGTCCAAAAACTGCTCGTGTACAGGCTAGGGCAGTTCTTTGTGCTTTCTCTGAAGGTGACATGAGTGCAGTGGCTGAGTTGAACAGTTTAATACAGAAAAAGGTTATGTACTGCCTTGAACATCACCGTTCCATGGACATAGCTCTGGCCACTCGAGAAGAGTTGTCATTGCTTTCAGAAGTGTGTTCTTTGGCAGATGAATTCTGGGAGTTAAGATTACGTGTTGTTTTTCAATTGTTATTTTCTTCCATCAAATTGGGTGCTAAACATCCTGCCATATCAGAGCATGTTATTCTTCCTTGTCTGCGAATTATATCTCAGGCGTGTACTCCTCCGAAACCTGATATGGTCGAGAAAGAGGAAGGGATGGGAAAATCTGCTCCTGCTTCGCAGCTAAAGGATGAGAATAACTCAAGTCCATCTGGATCTTTGGGTGGCCTTGTCAGTGGAAGTAAGTCTGCACTTGAGTCGGGGGAGAAAAATTGGGATGATTCTCATAAGACCCAGGATATTCAGTTACTTAGCTATTCAGAGTGGGAGAAGGGAGCATCATATCTTGATTTTGTTAGGAGGCAGTATAAAGTCTCTCAGTCAGTCAAAGGTGCTGGCCAAAAGTCTCGTTCTCAGAGATTTGATTACCTAGCTCTCAAGTATGCACTTAGATGGAAGCGACGTGTTTCCAAGACAACTAAGAGTGAATTGTCAACATTTGAGCTGGGGTCATGGGTTACTGAACTTGTGTTGAGTGCTTGTTCTCAATCCATCCGGTCTGAGATGTGCATGTTGATTAGTTTGCTTTGTGCCCAAAGTTCGTCAAGACAATTTCGGTTATTGAACTTACTGATGACTTTGTTACCGGCTACTCTAGCTGCTGGTGAATGTGCTGCTGAATATTTTGAATTGCTTTTTAAGATGATTGATTCAGAAGATGCTCGCTTGTTCTTGACTGTACGAGGATGTTTAACTACAATATGTAAGTTAATTACCCAAGAAGTGGGCAACATTGATTCTCTCGAGAGGAGCCTACATATTGACATTTCACAGGGATTTATCCTTCACAAGCTGATAGAACTGCTTGGAAAATTTTTAGAGGTTCCCAATATTAGATCCAGATTCATGCAAGACGACTTGCTGTCCGAAGTCCTTGAGGCTCTTATTGTCATTCGAGGTTTGATAGTGCAGAAGACAAAGCTAATAAGTGACTGCAACCGGCTTTTGAAAGATCTCCTAGACAGTCTTCTGCTTGAGAGCAGTGAAAATAAACGTCAATTCATTCGAGCTTGTATCAGTGGTCTTCAAGTCCATGGAGAGGAGAGAAAAGGACGGACTTCTTTGTTTATCTTGGAGCAGCTCTGCAATTTGATCTCCCCATCGAAACCGGAGTCTGTATATCTCTTGGTGTTGAACAAAGCACACACACAAGAAGAATTTATCAGGGGATCAATGACGAAGAATCCATACTCTAGTGCTGAGATTGGCCCTTTGATGCGTGACGTTAAAAACAAAATTTGCCATCAGTTGGATCTTTTAGGTCTCGTTGAAGATGACTATGGCATGGAGTTGTTAGTAGCTGGCAATATCATTTCTCTTGATTTGAGCATTGCTCAAGTCTATGAGCAAGTCTGGAAAAAGTCAAGTAGCCAATCATCAAACTCGATTGCTAATTCTTCACTGCTATCTCCTAATGCACTCACGTCTGGCACTGGCAGAGAGTGTCCACCTATGACGGTTACTTATCGGCTTCAGGGATTGGATGGTGAAGCTACTGAGCCCATGATTAAAGAATTGGAAGAAGATAGAGAGGAATCACAAGATCCCGAGGTTGAGTTTGCGATAGCAGGTGCTGTTCGTGAGTATGGTGGGCTAGAAATTATTTTGGGCATGGTCCAGCGTTTACGAGATGATCTAAAGTCCAACCAAGAGCAATTGGTTGCAGTTCTTAATCTCCTAATGCACTGCTGCAAGATAAGAGAGAACAGACGAGTCTTGTTAAGGTTAGGAGCTTTAGGTTTACTTCTCGAAACAGCAAGGCGTGCGTTCTCCGTGGATGCCATGGAGCCAGCCGAAGGCATTCTTTTAATCGTGGAGAGTTTGACGTTGGAGGCTAATGAAAGCGATAATATTAACATCACACAAAGTGCTCTTACAGTCACTAGCGAAGAAACAGGAGCTGGCGAACAAGCCAAGAAAATCGTCCTCATGTTCTTGGAAAGATTGTGCAACACTTCAGGGCTTAAGAAATCAAACAAACAGCAGAGGAACACTGAGATGGTTGCAAGAATCTTGCCGTACTTGACCTATGGTGAACCTTCTGCAATGGGAGCTTTGATACTACATTTCAACCCTTACTTGCAAGACTGGAGTGAATTTGACCGGTTACAGAAGCAGAATCAAGACAATCCAAAAGATGAAGCCACTGCTCAGCAAGCTGCCAAACAGAGGTTTGTTTTGGAGAACTTTGTTCGAGTTTCAGAGTCTCTCAAGACAAGTTCATGCGGGGAGAGACTGAAGGACATCATATTAGAAAAGGGTATTACTAGCGTTGCAGTTAAACATCTGAAGGAGAGTTTTGGGCAGGCTGGGTTTAAATCAAGTGCAGAATGGGCTTCAGGTTTGAAACTGCCTTCTGTTCCACTTATTTTGTCCATGCTTAGAGGATTGTCAATGGGTCATTTAGCCACTCAAAACTGTATTGAGGAGGGAGGGATTTTACCTTTACTTCATGCTTTGGAGGGTGTTCCTGGAGAAAATGAAATTGGGGCGAGGGCTGAGAATTTGTTGGACACACTATCTGATAAAGAAGGAAAGGGGGATGGGTTTTTGGAGGAGAAAGTAAGCACATTACGGCATGCCACTAGAGATGAAATGAGGCGCCGCGCTTTAAGGAAGAGAGAGGAGATGCTTCAGGGACTTGGAATGCGACGAGAACTATCATCTGATGGTGGTGAGCGAATTGTTGTTTCTCGACCAATCCTCGAAGGTTTCGAAGACGTTGAGGAAGAAGAAGATGGGTTGGCATGTATGGTATGCCGCGAAGGCTACAGTTTGAGACCTACTGATTTATTAGGAGTCTACTCTTACAGCAAGCGTGTGAACTTAGGTGTTGGAACTTCAGGAAGTGCACGGGGTGAATGTGTTTACACAACGGTTAGTCATTTCAATATCATACACTTCCAGTGTCATCAAGAAGCTAAAAGAGCAGACGCGGCTTTGAGGAATCCAAAGAAAGAGTGGGAAGGGGCTACACTTAGAAACAATGAAACTCTTTGCAATTCGTTATTTCCAGTGCGAGGTCCATCTGTTCCGATAGCACAGTATGTTCGGTATGTTGACCAGTATTGGGATAATCTGAATGCGCTTGGACGTGCTGATGGGAGCCGGTTAAGGTTGCTAACTTATGACATTGTTCTGATGCTCGCCCGATTTGCCACAGGCGCATCGTTTAGTGTCGATTGCAAAGGTGGAGGAAGAGAAAGCAACTCACGTTTCCTTCCATTCATGATTCAAATGGCCCGCCACCTTCTTGATCAAGGAAACAATTCTTCTCAACGCAAAACTATGGCCAAATCTGTATCGACTTACTTGGCAGATTCTCGACCTTCTCCTGGAACCCAACCCTCTGCTGGAACAGAAGAAACTGTACAATTCATGATGGTCAACTCACTTCTCTCCGAATCATATGATTCGTGGTTACAACACCGTCCTGCGTTTTTACAACGTGGAATCTATCACGCGTACATGCAACACACGCACGGCAAGTCAACTACTGGAACTGAGGATGAAAATCTACTGGCTATTGTTCAACCGATGCTTGTTTATACTGGGTTGATTGAACAGCTTCAGTGTTTTTTCAAGGTGAAAAAGGTGGTAAGAGAAGAAGGAACAGGAACGAGTGGTGGAGGAGAAGGAGATGGTGAGAGTTTGGAGAGTTGGGAAGTGGGGATGAAGGAGAAGTTGTTGAATGTGAAAGAGATGGTGGGATTTTCGAAGGAGTTGCTGACGTGGCTTGAGGATATTACTTCAGTTACTGATTTGCAGGAGGCGTTTGATGTGATTGGGGTTTTAGCGGATGTATTGTCGGGTGGGTTTGCAAGGTGTGAGGATTTCGTGCATGGTGCGATTAATGCGGGGAAAAGTTGAGATTTTTGAATATGTGAGGAGCATGATGTGTTGTAATTAGTGTTGGTTGTGTGTTTAAAGTTTGTAATTAAGATGTAATTATCAAATTAGTATTGTTGAGTGATGCTTGTGCCCATTCTTATGGATAAAGGGTGCACTCAACTTTCTCCATATTGTTGTCTGTTGAACACATAGCTCCTTGGTAATGGAAATTTTCCCCCAAATTCTAGCAAAAAAAAAAAAAAAAAAAAAAAAAAAAAGT

>transcript_21 full_length_coverage=11;length=7048

GGGAGAAATAAATCTGGCAAACCAAAAACTCTTCCCCCATCTCTACTCTACTCATCTCTCTCTCTCTGCACTTTCTCTTTCCCCCTTTTATCTTCTTCTCGATCTCCCCCTTCTCTTCTCTTCCACGACATCCATAGATGCATTGAGGAGCTTCTTCAGCCCACAGTCTAAAGGAGGCTTAAAGTCCTTTTTTTGACTGTGTGTTCAAGGTAGATCTGGTGTGAGAAAACTGAAAAGGATCTGGGTTAATTTATTTGTGAATCTTCAAAGCTGGCGGCAACACTGGCTTGGAGGTACACTGCCACTAATGGCAGTGGCCTTGCTGCTAACGACCTGGAAAGAAATTCAGATGCCAAGCATCAAGATCTTGAGCCTCCTACTCCACACTCGCTCATAAAAATGGGTTCCAGGGAGCGGAGTAGCATGGAGGATCCAGATGGGACATTAGCAAGTGTTGCCCAATGCATTGAGCAGTTGCGGCAGAATTCTTCTTCTTTACAGGAAAAAGAACGTTCTTTGAAGCAATTGCTAGAACTTATTGATACGCGTGAGAATGCTTTCAGCGCTGTTGGATCTCACTCTCAGGCAGTTCCGGTTCTTGTTTCTCTTCTCCGATCGGGATCCCTTGGAGTGAAAATGCAGGCAGCGACTGTTTTAGGCTCGCTGTGTAAGGAGAACGAATTAAGGGTAAAAGTGTTGCTTGGGGGCTGCATTCCACCATTGCTTGGTCTTCTCAAATCCAGCTCGGCAGAAGGTCAAATTGCAGCTGCAAAAACTATTTATTCTGTTTCTCAAGGTGGTGCTAGGGATTATGTCGGATCCAAAATTTTTTCAACTGAAGGTGTTGTGCCTGTGCTCTGGGAGCTGCTACAAAATGGGGTTAAGGCTGGAAACTTGGTTGATAACTTACTTACCGGTGCTTTAAGAAACCTTTCCAGCAGCACCGAAGGCTTTTGGTCCGCAACTTTAAAAGCTGGAGGAGTAGATACACTTGTGAAGTTGCTTACAACTGGGAAACCAAGTACTCAAGCGAATGTGTGCTTTCTTCTTGCGTGTGTGATGATGGAAGATGCATCTGTTTGTTCTAGGGTGTTGGCTGCAAAAGCGACGAAAGAATTACTTAAGCTATTAGGACCTGGAAATGATGCTTCTGTCAGGGCAGAAGCTGCAGGTGCTCTCAAGTCTCTCTCTGCCCAGTGTAAAGAAGCTAGGCAGGAGATAGCCAGTTCCAATGGTATTCCTGTTTTAATAAATGCTACAATAGCTCCTTCAAAAGAATTTATGCAAGGTGAGTACGCCCAAGCATTGCAGGAGAGTGCCATGTGTGCACTCGCAAACATTTCTGGTGGTTTATCATTTGTTATCTCAAGCCTTGGTCAAAGCCTTGAATCGTGCACTTCACCAGCACAGATTGCTGACACTTTAGGGGCTTTAGCTTCGGCACTTATGATATATGATAGCAAAGCAGAATCTACTCGAGCATCAGATCCTCTGATTATCGAGCAAACGTTGATTAAGCAGTTCAAGCCTCGAGTACAGTTTCTTGTGCAGGAGCGTACCATTGAAGCCCTGGCCAGTTTGTATGGGAATGCCGTATTCTCAGATAAACTTACGAACTCTGATGCTAAACACCTGTTGGTTGGTTTGATCACAATGGCCACTAATGAAGTTCAGGATGAGCTGATAAGGTCACTTCTTGTTCTTTGCAATGATGAAGGGAGCCTGTGGCGTGCACTTCAAGGTCGTGAGGGGGTTCAGCTTTTGATTTCTCTCCTTGGGCTTTCGTCAGAGCAGCAGCAGGAATGTGCTGTTGCATTGCTTTGCCTTCTATCCAATGAGAATGATGAAAGTAAATGGGCTATTACTGCTGCTGGTGGTATACCTCCGCTTGTTCAAATTCTAGAGACTGGGTCTGTAAAAGCCAAGGAAGATTCTGCAACAATCCTTGGGAACCTCTGTAATCACAGTGAAGACATACGGGCGTGCGTTGAAAGTGCTGATGCTGTCCCTGCTTTATTGTGGCTATTGAAGAACGGAAGTTCAAATGGGAAAGAGATTGCAGCAAAGACATTGAACCATTTGATCCATAAATCAGATACAGCAACTATCAGCCAGCTCACTGCATTGTTAACTAGCGATCTACCAGAATCTAAAGTTTATGTCTTGGATGCCCTAAGAAGTATGCTTTCTGTTGCTCCCCTCAGTGATATTTTACATGAAGGCAGTGCTGCAAATGATGCGATTGAGACAATGATAAAAATATTGGGCTCCACCAAAGAAGAGACTCAAGCCAAGTCTGCATCGTCTCTAGCTGGAATCTTTGATCTCAGAAAGGACTTGCGTGAAAGTAGTGTTGCTGTTAAGACTCTTTGGTCGGTCATGAAGCTACTAAAAGTTGAATCTGAGAAGATCCTAGTAGAGTCCTCGTGTTGCCTTTCTGCAATATTTCTTTCTATAAAAGAGAACCGAGATGTGGCTGCTATTGCTAGAGATGCATTGTCTCCGTTGGTATTGCTTGCTAACTCTTCTGTTCTGGAAGTTGCAGAGCAGGCGACTTGCGCTTTGGCGAATCTTCTTTTGGATAATGAAGTTTCTGAGAAAGCTATTCCTGAGGAAATTATCTTGCCCGCTACTAGGGTTTTGCGTGAAGGCACAGTTGGTGGGAAGACCCATGCAGCGGCAGCAATTGCCCGTTTGCTCTGTTCACGTCAAATTGATTATGCCTTAACTGATTGTGTGAATCGCGCCGGAACTGTTCTTGCACTAGTTTCTTTTCTGGAATCTACAAATAGTGGATCTTTTGCCACATCACATGCACTAGATGCACTTGCTTTTCTGTCAAGGTCAGAGGGAGCCAGCGGGCATGTCAAACCTGCGTGGGCTGTTTTAGCTGAATTCCCAAATAGCATAGCCCCAATAGTTTGGTCTATTGCGGATTCAACACCCTTGTTGCAGGATAAAGCTATTGAAATTTTGTCACGAATTTGTCGGGACCAGCCTGTTGTTCTAGGAAATACAATTGCTGGTGCTTCTGGATGTATTTCATCAATTGCTAGAAGGGTAATTGGTTCCACAAAAACAAAGGTCAAAATTGGGGGAACAGCACTTCTCATTTGTGCTGCTCGAGTTGATCATCAAAGAGTGGTCGAAGATCTGAATCAATCGAACTCACTTGATCAGCTTGCTCAATCTCTTGTTGTAATGCTTAGTTCCTTGCCATCTTCTATTTTGGGAAACCAGGGTGACTATGACATGGAGGCCATAAGTATTTACAGGCATTCTAAAGAAGAAGCCAGTGATGGCGAAACCAATAAAAGCACAGCAGTCATCAATGGTGCTAATATGACTATATGGCTACTCTCCGTTCTTGCCTGTCATGATAACAAAAGTAAAACTGTGATAATGGAGGCCGGAGCAATTGAAGTACTCACTGAAAAGATATCTCAGTACTCGTCGCAATATGCCCAGATTGATTTTAAAGAAGATAGTAGCATTTGGGTTTGTGCTTTACTGCTGGCAATTTTATTTCAAGATAGGGATATTATACGAGCACATGCAACCATGAAATCTATACCAGTACTCTCAAATTTGTTAAAGTCCGAAGATTCGGCAAACAGATATTTTGCTGCACAGGCAACTGCTAGTCTAGTCTGTAATGGTAGCAGGGGGACTCTTCTGTCTGTTGCAAATTCTGGGGCAGCTGGTGGGCTTATTTCCTTACTTGGCTGTGCTGATGCTGATATATATGATCTTCTAGAATTGTCAGAGGAATTTGCTTTAGTGCGCTACCCAGAGCAAGTTGCTCTCGAGAGGTTGTTTAGAGTTGATGACATTAGGGTTGGTGCTACTTCACGGAAAGCAATACCCGCACTTGTTGATCTACTTAAGCCTATACCGGATCGCCCAGGGGCACCTTTCCTAGCACTTGGGATTTTGACTCAGCTTGCAAAAGACTGTCCTTCGAATAAGATTGTAATGGTTGAATCAGGAGTTTTAGAGGCACTGACCAAATATCTTTCTCTTGGGCCTCAAGATGCAACTGAGGAAGCCGCTACTGATCTACTTGGAATCCTGTTTGGCAGCGCTGAAATACGGAGACATGAATCTTCATTGGGTGCTGTCAGACAGCTTGTAGCTGTGTTACGATTAGGTGGAAGAGGAGCAAGATTCAGTGCTGCTAAAGCACTGGAAAGCCTATTTTCTTCTGACCATATTAGGAATGCAGAAACTGCCCGACAAGCAGTTCAGCCCTTGGTGGAAGTTCTTAATACTGGTTTGGAAAAGGAGCAGCATGCTGCCATATCTGCATTGGTTAGGTTATTGAGTGAGAACCCATCGAAAGCCCTTGCTGTTGCAGACGTGGAAATGAATGCCGTGGATGTTCTGTGCAGGATACTTTCATCAAATTGTTCAATGGAGTTGAAGGGTGATGCTGCTGAGTTGTGTTATGTTCTTTTTGGGAATACTAGAATCAGATCTACAATGGCTGCGGCTCGATGTGTTGAGCCTTTGGTTGCTCTCCTCGTATCGGAGTTCAGTCCTGCACAGCATTCAGTTGTTCGTGCATTGGATAAGCTCGTGGAGGAGGAGCAACTCGCTGAACTAGTTGCTGCACATGGTGCAGTTATTCCTCTTGTTGGCCTGCTCTATGGTAGGAACTACATGCTTCATGAGGCCATTTCTAGAGCTCTTGTGAAGTTGGGAAAGGACAGGCCTTCTTGTAAGATGGACATGGTGAAGGCTGGCGTAATCGAGAGCCTGCTTGACATCCTCCAAGAAGCACCAGATTTTCTCTGTTCTGCTTTTGCTGAATTGCTCCGCATATTGACTAATAATGCTACCATTGCCAAGGGTCCGTCTGCAGCTAAAGTGGTCGAACCTCTCTTTTTGTTGCTAACAAGACCAGAATTCGGGCCTGACGGACAGCATAGTGCATTACAGGTTCTTGTTAATATTTTAGAGCATCCACAGTGCCGTTCTGATTACACCCTGACATCTCACCAAGCTATTGAACCACTTATCCCTTTGCTAGATTCTCCAGCTTCAGCAGTGCAACAGTTGGCAGCTGAGCTTCTATCCCATCTACTCTTGGAAGAGAATCTCCAGAAGGATCCAGTGACTCAGCAAGCAATTGGTCCCCTGATACGAGTTCTTGGTTCTGGTATACCCATTTTGCAACAGAGAGCTATAAAGGCTCTTGTTAGTATTGCACTAATTTGGCCAAATCAAATTGCAAAAGAGGGAGGTGTCAGTGAGCTGTCCAAAGTAATATTGCAAGCCGATCCTTCAGTCCCTCATGCCTCATGGGAATCTGGTGCTTCTGTTTTATCCAGTATTCTGCAATTTAGTTCAGAGTTCTATTTGGAAGTACCTGTTGCTGTGTTGGTAAAGTTACTTCGGTCCGGATCAGAAAGTACAGTAATTGGTGCATTGAATGCTCTTTTGGTGTTGGAGAGTGATGATGCAACCAGTGCCGAAGCAATGGCTGAAAGTGGAGCTATAGAGGCTCTTTTGGAACTTCTAAGATGTCATCAGTGTGAGGAACCCGCTGCTAGACTGCTTGAAGCGTTGCTGAACAATGTAAAAATCAGAGAATCCAAGTCTACTAAATCTGCAATCTTGCCATTATCTCAGTACCTCTTGGATCCACAGACTCAAACTCAACAAGCAAGGTTGCTTGCAACTCTAGCTCTTGGCGATCTTTTTGTGAATGAGGGCCTTGCTCGAACTAGTGATGCTGTTTCAGCTTGTCGTGCTTTGGTGAATGTTCTTGAAGACCAGCCTACAGAAGAAATGAAAGTGATAGCTATATGTGCATTGCAAAACCTTGTTATGTACAGTCGGTCCAATAAAAGGGCAGTTGCAGAAGCTGGTGGTGTTCAGGTTCTACTGGATCTGATCAGTTCTAGCGATCCAGATACATCAGTTCAAGCTGCAATGTTTATTAAGCTTCTCTTTTCTAATCATACCATTCAAGAATATGCTTCAAGCGAAACCGTCAGAGCTATAACTGCTGCTATTGAAAAGGATTTATGGGCAAGTGGAACTGTAAATGAGGAGTATCTAAAAGCTTTAAATGCACTGTTTAGCAACTTCCCACGGTTAAGAGCGACAGAGCCTGCAACACTTAGCATTCCTCATTTGGTGACATCCTTGAAGACTGGTACAGAGGCTACTCAAGAAGCTGCCTTGGACTCACTTTTTCTTCTCAGGCAAGCTTGGTCTGCATGCCCAGCTGAAGTGTCGAGAGCTCAATCGGTTGCTGCTGCAGATGCAATTCCATTGCTGCAATACCTGATCCAATCTGGGCCGCCTCGATTTCAAGAGAAAGCGGAATTTCTGTTGCAGTGTTTGCCAGGGACGTTAGTTGTGGTTATCAAGCGTGGGAATAACATGAAACAGTCGGTTGGAAATCCGAGTGTGTATTGCAAAATTACCCTTGGCAACACTCCACCTAAGCAAACTAAGGTTGTATCAACAGGACCCACTCCGGAGTGGGATGAGAGCTTTGCGTGGTCCTTTGAGAGCCCTCCGAAAGGGCAGAAGCTTCACATATCTTGCAAGAACAAGAGCAAAATGGGGAAGAGTTCATTTGGAAAAGTAACAATCCAGATTGACCGGGTAGTTATGCTGGGAACAGTTGCTGGGGAGTACACTTTGATACCCGAAAGCAAAAGCGGGCCGTCACGAAATCTAGAAATAGAATTCCAGTGGTCTAACAAGTAATAACCATTACTCAAATATTCGTTTGTTACATTGCTGGTACAAAAAAAATTCAATTAGAGACAAAAAGTGTTCAGAAAGGCTCTTTTTTTTTTTTGAAATTATTTATAATATATGTCATTGTATATATAGTCTGCTGGAGATTTTGATTATTGTAGTTCTGTGTAGTCCTAAAGTAGTCATTTGTTCATGTATTCTTTTGTAACCTGATCTTGGACAACTTTATTTTCTTTGTATTGAATTTTGTGTGCTCTGTAAAATTAGAGGAAAATAATATGGGTTGTTCCAGTTGTAAAAAAAAAAAAAAAAAAAAAAAAAAAAAAGT

>transcript_22 full_length_coverage=2;length=7048

GGGGACCCAGACTCTCCTCTTTCTCTTTCTCTCTCTCCAAAACTCTCTCTTTCAATCCTCTCCGTGACAGGCCTCTCATTTTGCACCTTCTCTCAGGCACTAAATTTCAGGGTGTTTGATTTGAATTGTGAGGGAAGCTTACTGTGGTGAGCTTGCCCTACATCATTGGTTCATTTCAAACAGTTGTGGTGGGGCATTTATGCGTGTCTCTATGCATGTGTGCGTGGGTTTTGATTGAATGCTGAATAATTGAGGTGGTGGTGATTCTTGGGCGGAGGAGGTGGTGGAGTTTGAGTTAAACAAAAGGAGGGAGTTCTAAGGCATTTGCATTATCGCCGAGGTGACCAAGGAGAAAGCTAAAGTGGAGTACTGTTGCAAATGTCAAAGTCTCCCTCCCCTGAACCACAAGTATCTACTTCTCGTCGTAGGGAATCAAATGGAGCAACGGGAACGAGCGACCCGGAAAGTACAGTGGCTACAGTTGCTCGTTTTATTGAGCAGCTACATACCAAAGTGTCCTCGCCACATGAGAAAGAACTTGTTACGGCCCGTTTACTACGTATCGCTCGAGCAAGAAAGGATGCCCGGACACTTATTGGTTCGCATTCCCAAGCAATGCCATTGTTCATATCTATTCTCAGGAGTGGCACCCCTGCTGCAAAAGTGAATGTGGCTGCAACTCTTAGTATTCTGTGCAAAGATGAAGACTTACGGCTAAAGGTGCTTCTAGGTGGATGCATCCCGCCACTACTTTCGCTATTGAAGTCTGGATCAACTGAAGCTAGGAAGGTTTCAGCAGAAGCTATATATGAAGTTTCTTCTGGTAGGCTATCAGATGATCACATAGGTATGAAAATCTTTATTACGGAAGGTGTAGTACCCACTTTATGGGATCAGCTAAATCCAAAGAATAAGCAGGACAGAGTAGTAGAAGGGTTTGTTACCGGGGCTTTGAGAAATCTTTGTGGAGACAAGGATGGGTATTGGAGAGCAACACTCGAGGCTGGAGGAGTGGATATTATTGTCGGGCTTCTTTCTTCCGACAATGCTGCTTCTCAATCTAATGCAGCCTCTCTCTTGGCTCGCCTGATGTTGGCTTTCGGTGATAGCATCCCTAAAGTAATAGATTCTGGAGCAGTCAAAGCTTTGCTTCGACTTGTTGGTAAAGAAAATGATATTTCTGTTCGTGCCAGTGCTGCCGATGCTTTGGAGGTCCTTTCATCAAAGTCAACCAAAGCTAAGAAAACTATTGTGGATGCAGAAGGTGTTCCAGTTCTCATTGGGGCAGTTGTTGCTCCTTCTAAAGAGTGTATGCAAGGGGAGTTTGGCCAGGCTCTACAGGAGCATGCAACACGTGCTTTATCAAACATATGCGGTGGGATGTCTGCTTTAATACTATATCTTGGAGAACTTTCACGATCTCCTCGCTTAGCTGCACCAGTTGCTGATATAATCGGAGCACTTGCCTACTCTTTGATGATCTTTGAGCAGGAAGAAACCTTTGATGCCACACAGATAGAAGATATTTTAGTATTGTTACTAAAGCCCCGGGATAATAAGCTTGTTCAGGAGCGTGTCCTTGAGGCCATGGCCAGCTTGTATGGAAACATCTATCTCTCAACGTGGTTCAAACATGCAGAAGCAAAGAAAGTACTCATTGGACTCATAACAATGGCAGCTACCGACGTGCAAGAGTATTTAATACCGTCATTGACAAGCTTATGCTGTGAAGGGGTGGGCATCTGGGAGGCCATTGGGAAAAGGGAAGGAATCCAGCTATTGATATCATTGCTGGGCTTATCTAGTGAGCAGCATCAAGAGTATGCCATTCAGTTGCTAGCTATCTTGACTGACCAAGTTGATGACAGCAAGTGGGCTATTACTGCTGCTGGTGGAATTCCTCCACTAGTGCAGTTATTAGAGATGGGATCTCAGGAGGCAAGGGAGGATGCCGCGCATGTTCTGTGGAACTTGTGCTGCCACAGTGAAGACATCCGTGCCTGTGTTGAAAGTGCGGAAGCTGTCCCAGCATTTTTGTGGCTTCTAAAAAGCGGAGGACCAAAAGGGCAAGAAGCATCAGCTATGGCACTTACAAAGCTTGTCCGAACAGCTGATTCTGCCACCATTAATCAGTTAGTAGCTTTGCTCCTGGGAGACTCTCCTAACTCAAAGACTCACATAATTAGAGTTTTGGGTCATGTGCTAACCATGGCCTCTCACAAGGAGCTTGTGCACAAGGGAACTGCAGCTAATACAGGGGTGACATCTCTTGTCCAGATTCTCAACTCCTCAAATGAAGAAACCCAAGAGTATGCAGCTTCTGTCTTGGCAGATCTATTCAGCAACAGACAAGATATATGTGATAGTCTTGCTATCGATGAGATTGTGCATCCTTGCATGAAGCTTTTGACCAGCAAATCACAAGTTATTGCAACACAGTCAGCTCGAGCCTTGGGTGCTCTGTCCCGTCCAACCAAGAATAAGGTTACAAAAAAGATGTCTTATTTTGCAGAAGGGGATGTCATGCCCCTGATCAGGTTGGCTAAAAATTCTTCCATTGATGCGGCCGAGACTGCAGTTGCTGCGCTGGCCAATCTTCTCTCTGATCCTCAGATAGCCGCAGAAGCCCTTGCAGAAGATCTTGTTTCAGCTTTGACTAGAGTATTGGGAGAAGGCACTTCAGAAGGTAAGAAGAATGCATCACGCGCGCTTTATCATTTACTTCAGCATTTTCCCGTAGGTGATGTACTCACTGGAAATGCTCAGTGTCGTTTTGCTGTGCTTGCGCTTGCTGATTCCTTAAAAGAAATGGATATGGATGGGACTGATGCTTATGATGCCTTAGAAGTTATTGCACTATTGGCTAGGACGAAACAGAGCGTGAACTTCACATACCCCCCATGGTCTGCCCTTGCCGAAGTTCCCTCAAGCTTAGAGCCTCTTGTGCGCTGCTTGGCTGAGGGGCCTCCTCCGCTGCAAGATAAGGCTATCGAGATTTTATCTAGGCTTTGTGGGGATCAACCAGTTGTCCTGGGTGATCTGTTGGTTGCAAAATCGGCATCCATTGGCTCACTAGCCAACAAAATAATGAACGCTTCCAGTTTAGAAGTGAGAGTTGGAGGGACTGCACTACTCATTTGTGCTGCAAAAGAACACAAACAACAGTCGATGGAAGCACTTGATGCGTCAGGATATTTAAAACCACTAATATATGCTTTAGTTGAAATGATGAAACGAAATTCTAGCTGCTGCTCTCTAGAAATTGAAGTCAGAACTCCTAGAGGTTACACGGAAAGAACTGTGTTTCAGGATGGCGATGAGTTTGAGATTCCTGATCCAGCCACTGTTTTGGGCGGTACCGTTGCCTTGTGGTTGCTGTCAATAGTATCTTCGTTTCATGCAAAAAACAAAGTTACCGTTATGGAAGCCGGTGGAGTTGAAGCCCTTTCGGACAAGCTTGCAAGTTTTACTTCCAATCCACAGGCAGAGTTTGAGGATACAGAAGGTATTTGGATTAGTGCGCTGCTTATGGCTATTTTGTTCCAAGATGAAAATGTTGTTTTGTCTCCTGCAACTATGCGCATCATACCCTCACTTGCTCTCCTGCTGAAATCTGATGAAGTTATTGATAGATTCTTTGCTGCCCAGGCAATGGCTAGTCTTGTTTGTAACAATAGCAAGGGGATTAATCTTGCCATTGCGAACTCAGGTGCAGTTGCTGGGTTAATTACCTCAATTGGTTACCTAGAATCAGATTTACCAAACCTTGTTGCTTTATCAGAGGAATTTTCCCTGGTACGAAACCCTGACCAAATTGTGCTTGAACACCTTTTTGAAATTGAAGATGTAAGAGTTGGTTCCACTGCACGGAAAACTATACCTCTGTTGGTCGATCAGTTGAGACCAATACCAGATAGGCCAGGTGCCCCACCAATTGCTGTTCGAATCTTAACTCGTATTGCTGATGGAAGTGATACAAACAAATTAAGCATGGCTGAAGCTGGTGCTCTGGATGCTCTAACAAGGTATCTATCTTTGAGCCCTCAAGACTCAACAGAGGCTGCTATATCTGAACTTCTGAGAATATTATTTAGCAATCCTGACATCCTTCGATACGAAGCGTCAGCTAGTTCTTTGAATCAACTTATAGCTGTTCTGCGTTTAGGATCAAAAAGTGCTAGATTCAGTGCAGCTGGGGCTCTGCACGAACTTTTTAATGCTGAGAACATCAGAGATTCTGAACTAGCCATGCAGGCTGTTCAGCCCTTGGTTGACATGCTTAATGCTGCATCAGAGAGTGAGCAAGATGCTGCCCTTGTTACATTGATCAAATTGACTTCGGGGAAGTCTTCAAAAGCTGGTATGTTGGCTGAGGTGGATGGAAACCCCCTTGAAAGTCTATACCGAATATTATCGTCCACTTCATCCTTGGAACTTAAGAGAAATGCTGCACAACTTTGTATTGTTTTGTTTGATAATGCAAAAGTTAGAGCAAATCCCACTGCCTCTGAGTGCATACAGCCCCTTATATCGCTGATGCAATCTGATACAAGTACAGCAGTGGAATCTGGTGTTTGTGCATTTGAGAGATTGTTGGATGATGAACAACAAGTGGAGTTTGCGGCAGCTTATGATATTGTTGATCTCCTTGTTGGCTTGGTTTCTGGATCAAATCATCGACTTATTGAGGCTAGCATCTCATCTCTCATAAAGTTGGGCAAAGACCGGACTCCCCGCAAACTGGATATGGTCAAAGCTGGCATTATTGATAATTGTCTTGAGTTGCTCCCTGTTGCACCCAGTTCGTTATGTTCTTCAATTGCAGAACTGTTCCGCATTTTAACAAATAGTAGTGCAATTGCTAAAAGTTCAGCTGCTGCAAAAATTGTAGAACCCCTTTTTTTGGTTTTGCTTCGTCCAGATTTCAGTATGTGGGGACAGCACAGTGCATTGCAAGCACTAGTTAACATTTTGGAGAAACCACAAAGCCTTGCCACTTTGAACCTCACTCCCAGCCAAGTTATTGAGCCTCTGATTTCATTTCTAGAATCTCCATCTCATGCAATTCAACAACTCGGTACAGAATTGCTATCTCATCTTCTTGCTCAGGAGCATTTTCAGCATGATATTACAACAAAAAATGCAGTTGTGCCCCTTGTCCAGCTTGCAGGGATTGGAATAGTGAACTTACAGCAAACAGCAATAAAGGCATTAGAAAATATCTCTACTAGCTGGCCAAAGGCAGTTGCTGATGCTGGGGGTATTTTCGAGCTCGCAAAAGTTATTGTTCAAGAAGATCCTCAACCACCTCTTGCATTATGGGAATCAGCTGCATTGGTTATCTCTAATGTGCTGCATTCCAATGCTGAATACTATTTCAAAGTTCCTGTGGCGGTTCTTGTGAAAATGTTGCACTCTACACTGGAGAGCACTATTACTGTGGCTCTTAATGCTTTAATTGTTCACGAGAGGAGTGATGCTTCATGTGCCGAACAGCTGACTGAAGCTAATGCTATAGATGCTTTGTTAGACTTACTAAGATCTCATCAATGTGAAGAAGCATCTGGAAGATTACTTGAATCTTTGTTTAACAATGTTAGGGTACGAGAGATGAAGGTTTCCAAATATGCAATAGCGCCATTGTCGCAGTATCTGTTAGATCCGCAAACCAAATCACAATCTGGCCGGCTTCTTGCTGCTCTAGCTCTTGGCGATCTCTCCCAGCATGAAGGACTCGCTAGAGCTAGTGATTCTGTCTCCGCGTGTCGTGCACTGGTGAGCTTGCTTGAAGATCAGCCAACAGAAGAAATGAAAATGGTGGCTATTTGTGCATTGCAAAACTTTGTTATGCACAGTAGAACAAACAGGCGAGCTGTTGCAGAAGCAGGGGGTATATTGGTTATTCAGGAACTACTTATGTCTCCAGATCCAGAACTCGTTGGTCAAGCTGCCTTGCTGATCAAATTTTTATTCTCTAATCACACGCTTCAAGAGTATGTATCAAACGAGCTCATCAGATCTTTGACGGCTGCATTAGAGAAAGAATTATGGTCCACAGAAACTATTAATGAAGATGTCTTGGGAACCATACATGTGATATTTACCAACTTCCCTAAGCTTCACATTTCTGAAGCTGCCACTCTATCTATATCTCATTTGGTAGGAGCTCTTAAATCTGGGAGTGAAGCTGCTCAGGAGTCTGTATTAGATACCCTATGCTTATTAAAAGATTCTTGGTCATCCATGCCAATCGATATTGCAAAATCTCAAGCAATAATTGCAGCTGAAGCTATTCCTATTCTTCAGATGTTGATGAAAACCTGTCCACCGGGTTTCCATGAGAGAGCAGATAGCCTGTTACATTGCCTGCCTGGGTGTTTAACCGTTACGATTAGGCGTGGGAATAACCTAAAGCAAGCCATGGGTAGCACAAATGCTTTTTGTCGGTTAACAATAGGCAATGGTCCTCCACGGCAAACCAAGGTAGTGAACCATGACACATCTCCAGAATGGGAAGAAGGATTTACCTGGGCATTTGATGTACCACCGAAGGGGCAAAAACTCCACATTGTTTGCAAAAGCAAAAATACTTTCGGAAAGACCACTCTTGGGAGAGTAACGATTCAAATCGACAAAGTCGTGACAGAAGGCGTCTATAGTGGATTGTTTAGCCTTAACCATGACAATAATAAAGACGGAGCTTCTAGAACACTCGAAATCGAGATTATCTGGTCAAACAGGTCATCGAACGAGAGAGAATCTGAAGAATAAAATGCAGTTAAATCAGGTGTAGTCCGTGTTTGCTTGTAAGTAATTATGAATGTATTAAAAGGTCCAGTTTTGTAGTTCTGAAAGAATATGTATGCCTCCTGAAGTGTTGATTAGTTTGGAGAAGTGGGTGAAAAAAAGGGGTTCCCACGTGTAGGGGAATATTAGAAGTTTGTTGTACAGGGTCAGTGTCTTTGTAGGTACTAGCAATTTAAACATGTTTAGAGAAGGAATCGAATTGTGACAAAAAAAAAAAAAAAAAAAAAAAAAAAAAAAAAGT

>transcript_23 full_length_coverage=2;length=7076

GGTGGATCCAAATGATCCGGATCCAAATACTGATGGTGAAACGAGTGGCTTACATGTGCAGGTGAATGATGCTGGTGATGAAAATGCTGATACTTGTAGTAAAGACATTTGTGATGGAGGGGAGGAACCCCATGTTATGAGTAACGAGGTTCACATTAACAATCAAAATATGGAGAGGCATGCATTTGAAAGCGGTGCCTATTATTTGGATCCAAATGCTGATGGTGAAAAAAGTGGTATACCCGTGAAAGTGAATGATGCCAGTGATGAAAATGTAAATAGTCCAGGTAAACACATTGCTGACAGAGGGGAGGAGCACCATGCTTTGATTAAAGAGTTTCACATGAGTGACCAAAATATGGGGAGACATCCAGTTGAAAGCAGTGCCTATAATTTGGATCCAAATGCTGATGGTGAAATAAGTGGCTTACCTATGCAGGTGAATGGTGCCAGTGATGGCAATGCAGATAGTTCAGGTAAAGACATTGGTGACGGAGGGGACCATGTTCTGAGCGAAGATGTTCACATTAATGATCAAAATATGGAGAGGCTTCCATTTGAGAGTGGTGCCTATAATTTGGATCCAAATGCTGATGGTGAAAATATTGGCATGCCTGTACAGGTGAATGATGTCAGTGATGAAAATGCTGATAATCTAGGTAAAGACATTGCTGACAGAGGGGAGGAACGCCATAGTTCGAGTAAAGAGGTTCTCATGAACGATCAAAATGTGGAGAGAGATGCAGTTGAAAGCATTGTCTATAGTTTGGATCCAAATTGTTCAGATCAAAATGTTGATGGTGAAAAAGGTGGCTTATCTGTGACCGAATGCGATGTTGCAAATCAGATGGAAGTTGATATGACATCTGATGTATCTTTGGATAACAGGGCACAAGAGAATTCTTCTGCTTCTAGGATGCAAGTTGATCATCTAGTCACTTCTGCACAGGATATCGGTACAAGCATTATGCAGTTGAACAATCAAGAGGCCGTGCAGGTAAATGATTCTAGACCTTCTAGCGATGAGAACGAAGATGGTTTACTTAAAGGCAACGGTGAAGGAGGGGGGGTTCAACCTGTTCTGTGCGAAGTTGTTCAAATGAATGATCAGAATGAGGAACATGCAGTTGAAAATGGTGCTTATAATTTGGAGACCATTCCCAGTCTGGCCTCCAGGGTTAAATCTACCGGAGAACGACCTGCACTTGAAACTAGAATCAGTAATGCGGAGGAACCTTCTAGTATGATAATGAAGGGGGATCCTGCCTCACAGATTGTGGAAGGATGCAGTGAGGATACATGTGCATCGATGCCTGTTCAGGTCAGCAAATGTGAAGTAGTGACTTCGTGCAGAGACACAGAGTCGTGTGATCAGTTTAAACAAATCATGCATGAGAAATCACCCATAGCTTTGGGAGGTGATAACAGTTATAAGGGGAATGCCGTTGAGTTCAGCAACACGATATTGGGGATATGTGCTAGTCCAGGTCTGAAGAAGGATACTCTTGCAGAGATGTCATATGGGCAGGGTTCTGTTGAGAAAAGAGAGGATTTGTTAGAAAGAGGAAATGAAAAGGATAATGTTCCAGTCCGCAATTCTGAAGCATCTCTGTTATCTGTAAAAGGTGGCAAACCTTCTAAAGATCAATGTGATGGAAGCAGTTATCGTCAAGTGGGAGATATATCTAGTGTAAAGGTGGTTTTTTCTTCAGCTGAATTGCTTACGGAAACACATGCAACTAAACATTGTAAAGGTGTTCTTGATCCTTTTGGAGTTCGCAGAGAGGACTCAAATAGTGAAGACCTTGTCTCATCTTCTACTGTGGCAAAATCCATGGTATATGAAGATGATGTAGTCTCCCGGCAAGGTGTTGATGGTAACTTTGACCGAGATGTTTCTGTTATCGAAAAGGAGAATTTACAGTTGCCCACGGATTCTAGTGATGTGGGTTGTGAGATCGCCAGTTCCCTTATCATTCATAATAAGGCTGGATCCTCGTCTCCAGGTGACGAAACCCTAAATGTAAATGATAAAGAGGCGTCTCTAAAAGTTGCAGAGTCGATTCTAAACAAAGGCGATATGCTCACACAGCCTGTGCCGCCTTTAGAGGACTCATCCGATTTTGGCCAAATAGGTCAGAAAGATAGTGAAGGTATATTGGAACCTGCTGATAATAATTGCGGGCTGATTCCTATCCCAAGCAGTGAAGGTAATGCTTCATGTGCCCATGATGGTTCTTTGAGTTCTGTTTGTATGTCCGAATGTGAAGATAAGGTTCGGGTGCTGGAGGGTGGAAGTGTTTATGCAGATTCTGACAAACCTAATTGTGGATCTCCCACTGTTATTAGTTGCACTGAACTTTGTAAAGAAAAAGAAAATCAGCAGGGAGTCAAGGGGCCTTTGGATCAGATTGCCCCACATTCTAATATCAATGATGGTTCTGCCAAAAAAGTGTGCTCTACTTCCCAGGATATGAAAGAAGATGATGCCTTAGACGAGAAGAGTTTTTCTTTTGAAGTAAATTCATTGGCTGACTTGCCTGGAAGAGAGTCTGGCAAGGGTTGGCAGCCATTTACCACTATTCAAGATCCCAAAGTATCCATGATTGTAGAGGGATCTCCTACGATATCTGGTTCAGGCCAAATTGATTATAAGTTGGCACAAGAAGTTCCCCATGCAAGTCCTCGAGCGTCTAATGGAGTGAATATAGTTGGTGGTTCCAGAGCTACTCCTGAGCGTAAAACAAAACGAGGGTCTGGTAAGGCAGCAGGGAAAGAAAGTGCTAAGAAAGGAAATCGTACAAAAGAATTAACTCCCATGAAACAGCAACTAGACAGAGTTGAAAAATCATGTGTTGTGTTGCCGAGCCCACCTGGGACTTGCCTGCTTGTGCAATCCAAAGAGATGCAGCACTTTGGGATTATGGAACGCAATAACAAAAAACCATCTAATGCTTTTACTGCGACATCTGGTCTGCCAGATTTAAATACTTCAGCTTCTCTACCGGCAGTGTTTCAACAGCCTTTTACAGACATGCAACAAGTGCAACTGCGTGCTCAGATCTTTGTTTATGGATCTTTGATCCAAGGAGCAACACCTGACGAGGCATGTATGGCATCTGCTTTTGGGGGACTGGATGGTGGAAGGAACATTTGGGAGAACGCATGGCGCACATCTATAGAAAGGCTTCACGGTCAAAAATTTCATCCCAGTACCCCTGAAACCCCTTTGCAGCAACGTTCAGGTGTTAGAGCTTCAGATCAAGCAGGTAAACAGGGCACACTTCAAAATAAAGTCATCTCTTCACCTGTTGGTCGAGCTAGCAGCAAGTGTACTCCGCCACCAGTTGCAAATCCTATGATACCTCTTTCATCACCACTTTGGAGTATTTCCACCCCATCATTTGATGGTATGCAATCTAGTGGCATGCCAAGAGGTTCAGTTATGGAACATAACCATGCAATTTCACCATTGCATCCTTTTAAGATCACACCTGTAAGGAATTTTGTTGGGCATAATGCTTCTTGGTCATCTCAGGCCCCTTCTCCTTGTTCTCGGGTTGCTTCTCCACAAACATCGGCCAGTTCCCGTTTTTCTGCATTGCCTATCACAGAAACAGTTAAATTAACTCCTGTTAGGGAATCATCCGTACCACTTGCCTCTGCTTTAAAGCTCGTATCTCCTAGTACTATTGTTCATAGTGGGGATCCTACTACTGTTTTTCCAGGGACTGCTTCCTTGCTTGATGTGAAAAAGGCAACATCATCACCTAGCTCAAATTCTGCAGATCCAAAGCCAAGGAAGAGAAAAAAAGTCCCCAGTTCCTGAAAATCCTGGTCTTATCTCTTTGGCACCTCAAACTCGAATAGAACCAGTTTCTTTGACACCTCAACCTCGAACAGAACCAGTCTCTTTGACACCTCAACCTCGAACAGAACCAGTCTCTTTAACAGCTCGAACCCGTACAGAACCAGTCTCTTTGACCCCTCGAACTCGAAGTGAACCAGTTTTTTTGACCCCTCGAACTCGAGTAGAACCAGTCTCTTTGACACCTTGGACACGAACAGAACCAGCTTCTTTGCTGCCTCGAGCCCGGACAGAATCAGTTTCTACTCCAGATGCCTGCAATAGTCATTTATTTACAACTGTTACTGTCACAACTCCTAGTTGCACAGTGTCTCAAAGTAAATCTGGGAAATTTTGTACAGCATCATCATCTCCAGCATCTTCCACTGATTGTCAGAAAATTGGGGATGTGGGTGTAGATCAGAGGGTTATATTATCAGAAGAATCCATTAATAATGTTAAAGAAGCTCAGTTGCAGGCGGAAAATGCTGCTGATCTTGCTGCTGCTGCAGTTAAGCACTGCCAAGGTATATGGAGTCAGCTGGATAAGCAGAAGATTTCTGGATTGATACCGGAGGATGAAGCTAAACTTTCTTATGCAGCTGTTGCAATTGCTGCCGCTGCTTCTGTTGCAAAGGCAGCAGCAGCTGTTGCTAAGGTTTCATTAGATGCTGCATTGCAGGAACAACTGATGGCTGAAGAAGTACTAGCTTCGAGAGGAAATGAAAATTCTTGTCAAAGTAACACGGTTCCTCTTTCTGATGGCGTGAATATTCTGGGGAAGGCTACATCTGCATCCATCTTAAAGGGTGAAGGTGCAACCAGCTGTTCCAGTTCAGTTATTGTTGCTGCCAGGGAGGCTGTTAGGAGGAAGGTTGAAGCTGCTTCGGCTGCTTCAAAGCGAGCTGAAAATATGGATGCTATAGTAAAAGCTGCAGAGCTGGCAGCGGAAGCGGCAACTCAGGCTGGAAAAATTGTTGCTATGAAAGATCCCTTGACTTTGACTGAATTAGTAAAAGCCGGTCCAGAGGGTTACTGGAAAATACCCCAAGTATCTTCTGAGCTGGTCCTGACGTCAAATAACACGAATAAAGGGCAATCTAACTTAGATAGCGTTGAAGATGATCGCGATGGTTCTGCTCAATGTTTGAAGGGTGAAACATTAAATGAAAAAGGAACACAGACCACATATGGGAAGCCATCGACTCAAAGTGATCTGTCCATGGAATCCATGGAGGGCCACACAAAATTGGTTGATGGCATTTCGAGTTCTGTAACAAACAGGGAAAAGGTTTTCAGAGCACAAAGGGGGCATAAAGTTTCTGACATGGCGAAAACCGTTGGTGTTGTTCCAGAATCTGAGATTGGATCAAAATCTACTTCCATCACTGTTCAAAATGAGTATGAAAAAGCGGGGGAGATTTCAAAAGAGAGCAACATCAAGGAGGGTTCACTTGTAGAGGTTTTTAAAGATGGGGATGGTATTAAAGCGGCGTGGTTCTCAGCCAAGGTGTTGAGCTTGAAGAATGGAGGGGCGTATGTATGTTATCCTGAAATACCATCAGAAGGCCAGGTAAAGGAGTGGGTGGCACTGAAAGGCGAAGGAGATCAGGCACCCAGAATACGAATTGCCCATCCTATGACCAGCATACCATTTGAGGGAACAAGGAAGAGACGCAGAGAAGCTGTGGGGGATTATGCTTGGTCTGTTGGAGATAGAGTTGATGCCAAGATCCGAGATTGTTGGTGGGAAGGAGTCATCACTGGAAAGAATGACGAAATCACACTCACTGTTCATTTTCCAGCCCAAGGAGAAACATCTTCTGTTAAACCTTGGCAGCTTCGGCCTTCTCTCATATGGAAGGATGGGGAATGGATCGAATGGTCCGGTTCAAGAGAAACCAAACGCTCTTCTCATGAGGGTATCGACGTGCCACAAGAAAAGCGGCTCAAGTTAGGCAGTCCTCTGGTAGATCCAAAAGGGAAGGACAAGATACCTGAACCTAGTATCTCTGAAGAGTTAAAACTAACAGGCTTATCATCAAAAGATAAAATATTCAATGTTGGCAAGAACGCTAGAGATGAAAACCAGCCTGACGGAGGAGGTAGAACAATGCGAACTGGTTTGCAAAAAGAAGGATCAAAAGTGATATTTGGTATTCCCAAGCCTGGAAAGAAGAGGAAATTCATGGATGTTAGCAAACATTACGTAGCAGAGAGGAGCACTAAACCTAAGCCGCCAGAAGAAGAAAATGATTCGATCAAGGTTAGTAAATATTTAATGCCACAAGGATCCAGCTCGCGTGGATGGAAAAATAATTCCAAAGTTGATTTGCGGGAGAAACGAGTTGCTGATTCAAAACCGAGTCGGGTTCTTAAATCCGGGAAACCACAAACCATTTCGACTTCATCCAGATCTTTACCTCAGAAGCAGCCTAATTTTACAGAGATCAAGGATTCTGGTCGGCATGGTGAGACTACATCAGGAAAGTCGAATGCGACCCCGTTTCGATCTTTTTCTAGTACCGAGGGTGCACCAGCTGGTCCGATGGTGTTTACTTCTACAGGTCTTATATCAGATAACCCTCCTTCCTCATCTAAGAAAGAAACCATGTCAAATTCAAAACGCCTGAATAAAGGAAGACTTGCACCTCCAAGTGGAAGGTCGGCTAGAACTGAACAGGAGAAAGTACATAATGGCAATTCTGTAAAATCGACTCCGGAAGTTGTTGAACCTCGTAGATCTTGTCGGAAAATTCAACCGACATCACGGTTATTAGAAGGGCTTCAGAGTTCATTGATCATCTCAAAAATACCTGCTGTGTCTCATGACAAAAGTCATAAAAACCAAAGCAGGAGTGGTTCTTCTAGAGGGAATAATAATCATGGTTGAGAGGAAATGACCGGGGCAATTTAAGGTTAATGACAGACAGAGGCACAACATCTCCCGTGTTGTTTTTGTGTATACTTGTGATAGAGAAAACCAGGAACGAAAATTCTCGCTTTCCTAAGGGTTTTAGTTGTAAAATGTGTTGAGTAGATGTTTATATATTTAATTTAGTTTTTGGTCTCGTCACAATTTATGATGATTTTAGTATTTCGAATGGGTTGGATGTATCTGGTAACTCTCATCTGGTAACTCATTGTAATTTGAGCTTATAATTGCAGGAAAATTTTAATCCATCCTTATGTTTTAGTTAAAAAAAAAAAAAAAAAAAAAAAAAAAAGT

>transcript_24 full_length_coverage=14;length=7018

GGGGCCGATTCTTATTAAAAAATTCAGAGGGCCATTTACTCTATAAACTGACCTATTGCTCTCAATCTGACCGAGAGGATGGAGACGTGGTTTTAAATCACGGGAGCTCCGGCGTGTTCACATTTCGTGGGTCTTGATTCTCTCAACCATAGGCGCCATTTAGAATTGGAACTGGGAGGAGATCCACGGCCTTAGAGGATTCAGATCGTGACCCATGGAGGATCACTGACAGAGCACCACACATCTTGAACTGAGAAGGGAGAAGATCGCTGGGTAAGGTTTCAGGGACACCGTGCGTCGACTTATCTTCAGGCAAGCAACTTTCTCTGTAACGGGCCTGATACAGAGGAGAAAGAAAGTTTGAAGTTCGGACTCTCTGTGGTGATTTTTAGATGGAAAAGAGGAATGATAGATTGTCAAGTTGAGATTTTCCAGCGAGAAGGATAGATTGCGTCTATTGGAAGCTGCGTCAGTTAGTCAAACTGAAACTGTAGGAAGGTTCCGTGAATGAACTTGGTTCTCTTTTGGAACTACTCGAGAGGTTGGAGACGCGTTTTTTAAAAGAATAAAAATCTGGAGCTCCGATGTGCTTATTTTTCAGTAAAGGAACAGAGTTCCATGAAAATATTCCTTTTACACGTGGTTCCTTTCTGGCGTGGCTGACGAAGCTTATCAGAGAGAGACTCTCTCCTGTAGCTTAAAGAGGACGCGAGCTAGCATTCTGTTTTTCCCGTGAGCTTATTCTCTGAAACAGAGCGGACTGTTACTTTTCCTTCCCAGCGAGCTTAACTGAAGAGAAGTGGCAGGCAAAGCTTATCGTGCGAAGAAGCTTAATGGGTCCTTTATGGATGGTGACCATTCGAGTTCATATATGGCATAATGTTGGAGGGCTTGTGAGTGGATCCGAATTGGGAAAAGGCCTAAAATCTTAACAAAAGAAAAAAAAAAAAACTCTTTCAGATAGTGTGTTGGTCAGTAGCTCCTTCATTTATCCTTCTCCATATATTTGTATTTCATCACTTTATATCTGGCCTGTGAGTTGTTGTCATCACTTAGCTTACATGTTGCATATTTTGCATTTGCATATTTTGTTCATGCCATTATATATGCATGTCATTTGTGTCTTGCATTCATAATTCTTGAGTTCTGATCATAGGGCATGCATATACTTTTAGGTGCTCCATTACATCTATGTGAACATGTTCATGAACACTTATTCTGATTGCATTTCTATTACTTGGCATATAGTGTTTGTGCATTCGTCATATCATTATTGCATATTTCATATGTGTTCATCACTCTACAAATTGTGAGAATTTCATGATCATTGAGTTCTGAGAAATTGGACTCAAGACTTGGGTGAGTAAGCCCATTGGTACCCTAGAAATTTATTGACCATATTTTTGTGGGTATGTGATTTATATTTTGTGCTCAAAATTTGTAATCAATTTTGTTATTGGATTCGGTTGTTGGTGAACCTCTTAAAACCATCATTTGGTTTGGAACGGTTATTGCTCATCCATAAAAGCATACCTTAGGTTCAAGTTTGTCCTCAATCCAAAAGCTTGTAGGTTGTGTGTTTGGTAAGAGTTCATAGGTTGAACTCAAACCAATTTGAGTCCACACTTTAGGTTACTAGCTTGTCCTAAAATTAAAAGCTAGTAGGTTGTGTGTTAGCCTAGGAAACATTCAAGTTCTTGTTGATCTTAGAAAACATGTTTAGGATTTTCTTTGCAGGTTTGTCCTTGTGATTTAGTCACTAAACAAAATGTGTAATTAGGTTCTTTGTGAGCCTTGTAAACTTTCAAGTTTAAGTTTAGCTTAGAAAAACTTTGTAAAGGTTTTTGGTCAACCTAGAAAACCAGTTTTTATTATAGTGGAAGAAACCTTAACGGGCCACATAGGCCTTGTTAGGATGAGTGGATGTAGGCTAAGTCAAGGAGGCTTAGTTGAACCACTATATATGCTTGTGTTGATTTTTCTTGCATGCTTTACTTTATTCGTTGCTTATCTATAGTCTTCATAACAATTCAACTCAAGCTATTGATTCTTCAAAGGAAAATCAAACTCTCAATTTTCACTCAACTTGGTTTAACTTAAAATTGGCAAATGATTTTCAAAAAGGTTCAAAAGATCCTATTCACCCCCCTCTAGGATCCATAGCTGCACTTTCAATTGGTATCAAAGCAGGTCACTCAATATAGGTTTAAACACCTGAGTGAGATCCTTTAGGGTTTCGTAGTCTTCTTTTCAGCCATGATGTCTTCCTTCGATACTTCGATCGATAAAGCTCCCATACTTGATAGTACTAATTATGATACTTGGAAATTTAGAATGAAAATCTTCCTTAAATCTTTAGACTATGGAGTTTGGTTTATTGTTGAAAAAGGTTGGTCTGAACCTGTGAAAATTAATCCTAAAAACAAAAACCGTACTATTCCTAAACCTCCCATAGAGTGGTCTCAAAAGGAGAAGAGTGACAGTGAATTGAATGCTAAGGCTTTAGAGATGATACTTAGTGCTGTCACCCAAAATGAGCCTAATCTTCTAACCACTTGTACTTCCGCCAAACATGCTTGGGATATCCTTAGGATGAAGTTTGAAGATGGTGTTGATCCTCCTTTAATGACTCTTACGAGTACTACAAATGACACCTCTTCTAAAGAGTGTCAAGAATTAGAGTCTGAAGACGAAAGTTCTGAATCGGAAGATGATCAAGATAATGACGAGGAAACCATTGAAGAATCCTATGAGAACCTATATAAAGATTTTGTGAGGATTGGTCTTGAGAATCAGAATTTGAAATGTCTCAATAAAAGTCTTCTTTCTAAGATCACTGATCTTAAGTCTTCAAAAATTTCTAAACTTGAGGATGATGTTGAGAGTTTAAAGTGTAAGAATCTTGAGCTAAAAGAAAGAAATGTCAACATTGAGTCTGAACTTGAAATTGCCTTGAATACTATTCGCACTGTAGAAGATAAGAATGTGATCCTTGAAAAGGGAATGGTTGATCTCAAGGACAAAATCTATGACCTAGAATCACAAAAGAAATTCGTTGAAATTTCTTGCTCCAAGGATGAGAAAGTTGAGCTGCAAATTAAAACTCTTGAATCTCAAATTTGTGATCTAAATGATTTGATGTTTAATCTCAAAAGCAAAAATGTTAAATTAGAAGATAAAGCTCACCAATATGACAAATTGAAGAATGAGTGTGACACTGCTTTAAATGAGATTCTGAACGTGGAAGAAAAGCTTAAAGTGTTTGAAAAGTCAAATAAGGAACTAATTGCAAAGATTGTTGATTTGGAAAACATAAACTCGAAGTTCCTTAGTGGTAGGAAGAAACTTGATGAGATGTTAAGAGCTGGTAAGAGTTATTATGATAGGACTGGACTTGGGTACCTCTCCGAGACAGCGAACAATTTAAATCCCAAGATAAAGTCCATTAAAGTGACTCAAAAACCTCTTGCTCGATACAACACTCAAGGGTACACATACCCTAAACCGAATACGAAAAACTTCCATTGCTATTACTGTGGTATTCGTGGTCACAAACAGTTTGAGTGTAGACTTAAAAGGATTAAGAAGCAAGAATCTTGGAGATCCACTCACACTAAGAAAGTCTATCGGATTGTGAAAATTCATGATGAACCTTGGTGGCACCGCACTTCAAAAGTCTCTACATCTCAAAAGATGAAAGGAAAGATGCATGAGCAATGCTCAGTTGCCTTAACCGCATGTCAAACTGGAAAAGACACTGCTTGGTACATGGACAGTGCTTGCTCTAAGCTTATGACAGGTGACAAAGATAAATTTTGGTTTCTAGAAGAGTTTGATGGTGGAGATGTTACATTTGGAGATGGATCAAAAGCAATGATCATTGGTAAAGGGACTGTGCATGTGTTTGACACAACTAAGATCGAGAATGTTCTTCTTGTAAAAGGCTTAAAGAAAAATCTCTTAAGTGTTGCTCAACTGTGTAAGGATGAGGACGTCGATATTATCTTCAACAAATCAAAATATGAAGTGTTTGACAAAGATCGTAATGTTTACATGATTGGAAGAAAATCTTCAGAAAATTGTTACACCTTGGATGTTGAGAATTGTGAAGACAACCGTGTTGGAATTTCATTTAACGTTGTCATGGAGCAGACCAACCACTTTGGCAAGGATCAGCAAGTGAATGCTACTAATGACACCATTGCTAGAGAGCCGGATGTTAGAGTTATCACCAGAAGACGATCTCAGGATGTTGAAAGACAAGTGTGCCTTACTTCTAACATGGACTATTATCCCACTAAGCATCAATTGTCGAACATCCTTACTAATTCTCTAGAAACCACTGGCTTTGAGTTTCTTCGCACTTCCATTGGAGTGTGTCAAGTAACTTAATCTTTCCTCACCGCATTTCATTTTCTTTTCTTTTTGTGTGTGTGAGTTTTAGTGTTTATGTTTGAGTCGATCTAAGTTTTGCATTGAGTCATTTTTGGTGTCTTGCGTTTTGCATCGAGTTCATACATTTCATTTTTGGTATCATTCGTGCATGTGTCTCATTCTTGCATTTTCCCCTACCATGCATTATTCTTCATTATTATATATGGCATTTGTTATGACAAAAAGGGGGAGAAAAGGTATTCTCTTGTTTGCTTTTGCAGGTATGGAGACAGGGGGAGCAATACAAAAGCATGAATGGAATTATAAATCTCAGGGGGAGCAAAGCTCGATTTCTTGAAAATGAAGAAAGTTCGTTTATTGAAGTTGAAGAATGCTTGATTCTTCAAGACGAAGAAAATACATATCTTGAAGTTGAAGAAAGCGCGTTGCTTAAAATTCAAGAAAGCTCAAGTGTGCAAGAATCGCTCCTGCAATCAACCAAGACGAAGAAGATGTTTTGTCATAAAATTGCCATTGGGGGAGATTGAAACGGCACCACATCGCACCGTTTTGTGAATGTCATTTTTATGACAAACAGGTTTGTGAAACTAGGTGTCCTTCCTCCCGGTTGCACCAAAGTTAGTGCAGAGTGGGAATTTGAGTCTCATGCATTTAGATTGTCATTGCATTGCATTCATGCATGTGTGTGTGTGTTGAGTGGTTGATTGCATGGTTTAGTGGACATTAATCCAAAATTATTTTCGTGTGTGTTGATCCGAACATGATGGGTTGAGGTGATTGATTTCAAATTCGTTTTAACCGAAAATCATTAAACAATTTTCTTTAAGAAAATCTTGCTCTTTATAAGTTGCTTCTACTTAATGATATTTATCCTCTCAAAAATATTTTCTAAAATTTCCCTTAAAATTTCTTAAGGTTTTTAATTTTACTTTAACACGATCAAAGAAATTTGAGTGTTAAATCTTTGATTAAAAATATATCTTTATTTTGTTAAAACAAAAGAAAGGGAAGATATTTTTTATTGGATTCTTGGTTTGACCCGGTCCGCTCACAAAATGCTGGAGAACAAGATTTTGAAAAATATGAGACAGGCAAAATGGGTATCGCTGAGGATGGTATATGCCTCGCATTTTCCAGTTGTGGAAATTACTAATTCGTCCTCCTTCATACGCGCGTGAGGTATTCGTGGCTGTCATGAATTGTGCGTGAGCTTTCGTGCTCTCAGGCTTTGGGTGAAGAAACAGATTCTCTTTGTGACCGTTGGTCACATGTTGTCGAGCTGGCGGATAACGTTTCTTATCCTCGCCTGTTGCTATTGGTTATTCCATTTCATATTATAACCGTTGAAGGGATAGGGTTGTAGCGCAACCAACGGAGTAGCCATTACTTGTGCTCTCCTCATCTGGACACTCGGTTGTCCACATTCATTTCGGTTCTTAATCTTTTTGATTTTATTTCTCTCGGTTTATCATTTGTTCTATCGCGCTGGCGGATAAGATGATCCGAAAAATCTGGATCTGGTTCTCCTCTGGTTTTGTCCGTTGCTCCAGACAAGATCCAAAATTGTTTTTGCCCGTTGCTCTGTTTTTTAGCAGAGCTGAGGATAAGGTGACCGTTGGTGATGGTCTTTTAAATAGCTTGCTCTGGAAAATTTATGTTTCGCCGATTCTTATTAAAAAATTCAGAGGGCCATTTACTCTATAAACTGACCTATTGCTCTCAATCTGACCGAGAGGATGGAGACGTGGTTTTAAATCACGGGAGCTCCGGCGTGTTCACATTTCGTGGGTCTTGATTCTCTCAACCATAGGCGCCATTTAGAATTGGAACTGGGAGGAGATCCACGGCCTTAGAGGATTCAGATCGTGACCCATGGAGGATCACTGACAGAGCACCACACATCTTGAACTGAGAAGGGAGAAGATCGCTGGGTAAGGTTTCAGGGACACCGTGCGTCGACTTATCTTCAGGCAAGCAACTTTCTCTGTAACGGGCCTGATACAGAGGAGAAAGAAAGTTTGAAGTTCGGACTCTCTGTGGTGATTTTTAGATGGAAAAGAGGAATGATAGATTGTCAAGTTGAGATTTTCCAGCGAGAAGGATAGATTGCGTCTATTGGAAGCTGCGTCAGTTAGTCAAACTGAAACTGTAGGAAGGTTCCGTGAATGAACTTGGTTCTCTTTTGGAACTACTCGAGAGGTTGGAGACGCGTTTTTTAAAAGAATAAAAATCTGGAGCTCCGATGTGCTTATTTTTCAGTAAAGGAACAGAGTTCCATGAAAATATTCCTTTTACACGTGGTTCCTTTCTGGCGTGGCTGACGAAGCTTATCAGAGAGAGACTCTCTCCTGTAGCTTAAAGAGGACGCGAGCTAGCATTCTGTTTTTCCCGTGAGCTTATTCTCTGAAACAGAGCGGACTGTTACTTTTCCTTCCCAGCGAGCTTAACTGAAGAGAAGTGGCAGGCAAAGCTTATCGTGCGAAGAAGCTTAATGGGTCCTTTATGGATGGTGACCATTCGAGTTCATATATGGCATAATGTTGGAGGGCTTGTGAGTGGATCCGAATTGGGAAAAGGCCTAAAATCTTAACAAAAAAAAAAAAAAAAAAAAAAAAAAAAAAAA

>transcript_25 full_length_coverage=2;length=7059

GGGGTATCTCATTTACATGCTTTCATGCCCTAATCTCTCTCTTCACCTCAGTGTGTAACCGATTGATCTGTTACTCTTCCTTTCTTCAACCATATATATATATATATACACACACCCCTCATTAGTGCCTATTTAGTGTTGTTTGATCCATCAATAAGATTGTGGTTGTTCCCTCTGCCTTCAATAATATTGAGTTGTAGGAACAGAAACTCGAGCATTTCCAGCAGCTACATTTCTGTCCCCAATCTTTGATGCCTGAACTTCATCATTGACTCATGACACGTTTCCTGTTCATGTTAGTCATAAATGTAAGACCCAGGATCCAAAGATGAAGGAAACTGTTTTGACAGCTAGTAAGGTGATCAACAGAAATTGGGTCCTGAAGCGCAAACGGAGAAAGCTTCCATGTGGACCAGATCTATCCAATGGTAAGGAAGGTAAAGCTGTAGCCTGTGAATCCCTTAGGAATACTTCTTTGGCTAAATGCAGGGTGGAAAGCGTAATGAGTTCTGATCAGTCTCCATGTAAGAAGAAAGGGAATGATGGGTATTACTTTGAATGTGTGATCTGTGATCTTGGTGGCAACTTGCTGTGTTGTGATAGCTGTCCCCGAACTTATCATCTTCAGTGCCTTAATCCACCTCTTAAGCGCATTCCAACGGGAAAGTGGCAATGTTCAAGTTGCTGTCAAAAAAGTGATATGCGTGAATCTATTACTCATCTAAATTCCATTTCAAAACGAGCCAGAACAAAGAATACTACTACAAAATCCAAATCTGGAATCAAGATATCTGCCGCAGACAAAGCATCCTGCTTTTTTGGAAGTTCCATTCTTGGAAAGAAAAGATCCTCTGCCAAAGTAAAATTTGCCTCATCTCACAGAGTCCAATCCAGTGAGAATAAACTGGATTCCTTCCAAATAGATGATTCTTATAGCACCAAGCCATGCCATCCATCCATCCTTGATTCTGCATATGTGACTGTTGATAATGAAAAGAAACTTGAATTATTTTCAACACACACACCCATGGAGGGGGAATCAATCTCTCTTGCTAATGAAGTCTTGCTCTTATCTAGGACTTCAGATTTGGAGTCTAAAGGTGAAGCTTTTGAGAAGAAGGCCGACTTATCCTGTAATAACGGGTCTCCAGGAAGTAAATATGCTTTGGGTGCTGTCAGTAAGAATGCTAGAAAAAGAAAAATCAAAGTTGTTGATGATGACATTCAGAAGAAGTCTAGGACTGATAAGGGTAAATCTGTGGTAGATCTATCTAAAAAAGGTGGATCCAGAGCAAAGTCTGTATGCCACGGAACTAGTAAACTGCACCGTAAACGTAGAACTATTGACAACAGAGTTCTCAAGTCTCTTTCAAAAGGGGATGTTGGAACAAAGAGGTCAGATGTCCAGCAGGAAGACGAGAGGCCTTGCGAGGAAGCAGCTCATTCATCGCATGAGCTAGATGAAGCAGGAGGTATGGTGGATGAATTAGTGACACATGAACATAGTGTTACGGGTGAACTTCGACAGGTTGATCGGGTTCTGGGATGCCGAGTGCAAGGTCATAAAACAAACACTTCAAGTCACATCTCCGTGGCTGTTGCAAATGACGTGCCTTCTGATGATTTACTCTTTCCAAAAAATCAAAACAGATTATCAGAGGAAAATCGCAGTTCTGATATGGAAATAGATGGAGAATCTGATGAAAATCTTACCAAGGGTTGCCAGACTATTATTAATAGTGAAACAAGGATTAAGAATGATATCCGAGTAGACAGTGTACATGTTTACAAAAGATTGGCGACCAAAGAATGTAAAGTCAATGCCCTGGATTTAGTGAGAAAAGGCATCAAGGAATTGGGTTCTACTGTTACTAATGGTAAAGATCAAAATGATTCGGCTATACCTACCAGAGATTCTGGAAGAACTATTGAAAAGGCGGCAACAGAAGCAAGTGCAGATGCTAGCATGAAAAGTCAGTTTTCTAATGAAATTCCGAAAAAAAGTGATCAACCTGCCCCTCATGAAACCAAAGATATAGAACCTGATATAGCTAAGATAATGAGTAGTAGTGCTGAAAACAAAGTTCAGGACCTCACTCTGGCTGAATCTGCATTGTATGATGGAGGGACAGTCTTGTATGAATTTTTAGTGAAGTGGGCAGGGAAATCTCACATTCATAACAGTTGGATTTCTGAGGCTCAGCTGAAAGTTCTCGCAAAGAGAAAACTAGATAATTACAAGGCGAAGTATGGAATTGCAGTTATTAATATCTGTGAGGAACGGTGGAAGCAGCCTCAGCGGTTATTTCTATCCGTGCTTTGAAATATGGTAGGAGTGAAGCATTTGTAAAATGGAGCGGTCTTCCTTATGATGAATGCACTTGGGAAAGATCTAATGAACCCGTGGTTGAAAAGTCTTCACATCTGGTGGATCTGTTTAATCTATTTGAACAGAAAACAGTGGAAAATGATGCTACTAAGGATGATGCACCAAAGATGAAAGGTGGTTGCCACCAAGGTGAGATAATTACTCTGACAGAGCAACCTAAGGAGCTGAAAGGAGGTTCTTTGTTTCCGCATCAGCTAGAAGCACTGAATTGGTTGCGTAAATGCTGGCATAAATCCAAAAATGTGATACTTGCTGATGAGATGGGGCTTGGAAAAACAGTATCAGCTTGTGCTTTTATTTCATCATTATATTTTGAGTTCAAAGCAAAGCTGCCTTGTTTAGTCTTGGTTCCTCTCTCAACGATGCCTAACTGGCTCTCTGAGTTTTCATTATGGGCTCCCAACTTGAATGTCGTGGAGTATCATGGGTGTTCAAAAGCAAGAGCCATAATTCGCCAATATGAATGGCATGCTACAGATCCGAATGACTTAAATAAGAAAACACTTTCCTATAAGTTTAATGTTCTTTTAACTACATATGAAATGGTTCTTGCTGATTCTTCTCATCTGCGTGGAGTTCCTTGGGAAGTTCTTGTGGTTGATGAAGGGCATCGTCTGAAAAACTCGGGAAGTAAGCTTTTCAGCTTGCTCAATTCATTTTCTTTTCAACACCGTGTCCTGTTGACTGGTACCCCACTTCAAAATAACTTGGGAGAGATGTATAACTTGCTAAACTTCTTGCAGCCGGCTTCATTTCCTTCTTTGTCTTCATTTGAGGAGAAATTTAATGACCTTACAACTGCGGAAAAAGTGGAGGAGCTAAAGAAACTAGTTGCTCCACATATGCTTCGAAGGCTTAAAAAAGATGCCATGCAGAATATTCCCCCTAAGACCGAACGGATGGTTCCTGTTGAGTTGTCATCCATCCAAGCTGAATATTACCGTGCGATGCTAACAAAGAATTATCAGATTTTACGTAATATAGGAAAAGGAGTTGCTCAACAATCAATGCTAAACATAGTAATGCAGTTAAGAAAGGTTTGCAATCATCCATATCTCATACCGGGTACTGAACCAGAGTGTGGTTCAGTAGAGTTCCTTCATGAAATGCGGATAAAAGCTTCGGCTAAGCTGACACTGCTGCATTCTATGCTTAAGGTGTTATATAAGGAAGGTCATAGAGTTCTTATATTTTCACAGATGACGAAGCTTCTTGATATTCTGGAGGATTATTTGACTTTTGAATTTGGGCCGAAAACATTTGAGAGAGTGGATGGCTCAGTTTCAGTGGCAGATCGTCAATCTGCAATCACACGCTTTAACCAAGATAAAAGTCGCTTTGTATTCCTGTTATCTACACGCTCTTGTGGTCTTGGGATCAATTTGGCATCTGCTGACACTGTGATTATCTATGATTCTGATTTCAATCCTCATGCTGATATCCAAGCAATGAACAGAGCACACCGAATTGGGCAATCAAAAAGACTTTTGGTGTACAGGCTTGTTGTTCGAGCCAGCGTTGAAGAGCGCATCTTGCAGCTTGCAAAGAAGAAACTAATGCTTGATCAGCTTTTGTAAACAAGTCTGGTTCTCAAAAGGAAGTGGAAGATATCCTACGATGGGGAACAGAGGAACTCTTTAGAGATTCTTCTGGCATGACTGAGAAAGATACTATAGAAAATCTTAAAAGCAAAGATGAAATATCAACAGATATGGAATATAAGCACAAGAGGAGATGTGGTGGTCTGGGAGATGTTTACAAAGACAAATGTACAGATGGAAGCACTGGGATTGTGTGGGATGAAAGTGCGATTGTGAAATTGCTTGACCGTACAAGCCTCCAGTTTGGTTCACCTGAGATTGCTGAAGGGGATTTGGAGAATGATATGCTTGGCTCAGTAAAGTCCGTGGAATGGAATGACGAATCAACAGAAGAACCGGTGGCAACTGAATCACCTCCTGGTGATGATGTTTGTGCTCAAAATTCTGAAAGAAAAGAGGATAATTTGGTTATTGGTACTGAAGAAAATGAATGGGACAGACTTTTGCGATTGAGATGGGAGAGGTATCAAAATGAGGAAGAAGCAGTACTTGGTCGAGGAAAGCGCCAAAGGAAAGCTGTTTCTTATAGAGATGCATTTTCCATACACCAGAGTGAAACTTTGAGTGAGAGTGGTAATGAACTGGAACCAGATCCAGAACCAGAGCTCGAGCGGGAATATACCCCAGCGGGTCGTGCGCTAAAAGTCAAGTATGCTAAACTCCGTGCCAGACAAAAAGAACGTCTTGCTCAGAGGAATGTAATAGAAGGATCTTGCAGTATCGTGGAGAGATTTGAACTGGAATCACTGCCTCAATATCCTTGTACAAATGTCCAAAACAGGGATCCTATGACTAAATTAAATCAATCAGTCCGAGAGAAGGTTCCAGCAAATGGCCTGGAGGATAAGAAATCCAGTCAGGCATTGACTGCAGCGATGAGCAAGAATGCGTTAATCTCAAGGTTGGGTGGAGTATCAAAGCATAAAAGTCATCTGGATCTTTCTGTCAGACCCCCTGGCCATCCTTCACCAAGTCATAATTTCCAGGAAACAAGCTACTCACATTCGGCGCACACCGGCAACTTGTTGCCTGTTCTAGGGCTATGTGCTCCCAATGCTTATCAACGAGAGTCAGCTGAGTTGAACTTTTCAAGATCAAATGGAAAACAGAGAAGGTTGGAAATTGGATCTGATTTTCCATTCTCTCTTGCCCTGGTTCTGGAACTGCAGTCGAGATGGAGATAAAATGTATGAAGACTACCCGAGACAAATTAATACAACCTGATGCATCCGCAGAAATTATGTTGCAGCGGATGAAGACTGATAATCTTCTGCCATTTAGTCTGTATCATCCCACTACCCTACAAGAAAATGGGTGTGCAAGTTTCAGTGGGAATTTCTTCGATTTTCAGGAAAAGATGGCTCTGCCCAACTTAGCCCATGATGATAAGTTGTCCAGATTCCCAACTGTATCTAAAAGCATGTCCAGTCCACATTCTGATTATTTACCTAGTTTATCGTTGGGTAGTGGAGTTGACCGTTCAAATGACTCTATGCATGGGCTTCCCACAATGCCGCTACTGCCCAATTTTAAGTTCCTCCAATTAGATGCACCCAGATACAATGAAAAAGAGGAGTTGCATCCCATGTTGGCTTTGGGTCAGATCCCGACAACATTTTCATCGTTGCCTAAGAACCATAGGAAGGTTCTTGAAAACATTATGATGAGGACTGGGTGTGGACCGAGTAACTTGTTCAAAAAGAAATCTAAAGTAGATGGCTGGTCTGAAGACGAACTAGATTGCCTCTGGATTGGTGTTCGTAGGCATGGACGGGGAAATTGGGATGCCATCCTTAGAGACCCTAGGTTGAAATTTTCGAAGTACAAAACTTTAGAGGATTTGTCGGCCAGGTGGGAGGAGGAACAGGTAAAGATGTTGGATGGAGGGTCAGATTTTGCAGTGCCAAAATCAGTCAAGCCTAGGAAATCTTGCATATTTCCTGGAATCACTGATGGAATGATGGCACGGGCATTGCATGGAAGCAGACTCGGAGGAGTGCCGCTCAAGTTTCAGACCCACTTGACAGACATGAAATTAGGGTTTGAACCATCTGATCAGTTTGTTTTGCAAGACAAGCAGCATTTTGCACCAATCCCAACTTGGAATCCTGAGAAAATTCGGCCATGTTTTTTTGACATTCCCGATCCTGGTATTTCGAACCCTTATGTCCCCCCCAATCCTTTTGGAACTAGTAGCAGTTTTGATCTAGAGCAGCAAAAGGAGGATGATGTTTTGGACAGATCGTACGGTGGGTGTTCAAAGGGGAAGGAAGTAGCTGGTCCAGAGAACAAGTTGCCCCATTGGCTTCGGGAAGCCGTAAAACCACCCCCACCAGATCCCGACCTGCCACCCACGGTATCGGCAATAGCTGAGTCAGTTCGGCTGCTGTATGGGAAAGAGAAACCGACCATTCCTCCGTTTGTGGTCCCAGGCCCTCCTCCTCCCCAGCCGAAGGATCCACGTCCGAGTCTGGGCATTAAGAAGAAGAAAAGGAAACGCAATAAGATGAGGAGCCAGCCAGAAATTGGCCGGGATATCGATGTTGCTTCAAGCTCAATGACACCATTTTTATCCCAGGACCATGTGAACTTGAAAACATGTGTTGGATTGTCCCCTTCTCCACAAGTGCTTCCATTGGTGGCGTCTTGTATGACTCCTGGCCCATCGTGCTTGGAGAAATCTGTCAACCCAGGAGGAGTGGAAAATGAAAGTGGGGATTCAAGCAAAACTCAGTCTGATCCTCCTTTAGAGGAAATATCATCTGAAGGAACCGTGTCAGATCATCCTGCAACTGACCACCAACCATAGCCGCCCTTATTTTTAAAAGTTTCTTGGCCGGTCCTTAAATGTGTTGATTTTTATAGCCATGATGATGATGATGATGCTTTGCAGCGAGTGTTGAACATAGGACCAACCATTTAGAATATTTCTTGTGTAAAGCTTCTGTAACTGGAAATGGAATATATGTACATATGTATATGATGTTGCCCCTCGTTTCATATTTGCTTCTAAAAAAAAAAAAAAAAAAAAAAAAAAAAAAAAAAAAAAAAAAAAAAAAAAAAAAAAGT

>transcript_26 full_length_coverage=2;length=7032

GGGAGTATTAACACTAATATTATTATTTACTCCATATTACCACCACCACCACCACCAAACCAATCTCAAAAACCCAAAACAAACTCCTCCTCGATCTCTGCTCTCACCCTGTCAGTCGTCAGAACCTTCCTTGGCAAGATTCCCTGCTCTATCTTCCACCTTAAGCACCTGAACACCCGCCAGCTTTGACTGGATTTGGATCCGACAAGTTTCTGGTCAAGTCTGCGTCTGATTCAATAAGTTTCTTGACTCAGCATCTAGGTGGATATTAGATACATTCCCTGCCGCAAGATGGAATGATAGAAAGGCTTTAGACATGTACTGAATGAATGGAGCAGGAGCATGGCAGTAGAGTTTTGATGGTTTGCAAAGATTGACATATAAGTGATGTCGAGGTTATCTAAGTGGAAGCTTGAGAAGACAAAAGTAAAAGTGGTCTTTCGGCTGCAATTCCATGCTACCCATATTCCTCAAACAGGATGGGATAAATTGTTTATATCTCTCATCCCTGCTGATTCTGGAAAGGCAACTGCAAAGACAACCAAAGCTAGTGTAAGAAATGGGACCTGCAAATGGGGAGATCCTATATATGAAACTACTAGGCTTCTACAAGATACTAAAACTAAGCAATACGATGAAAAGCTCTATAAACTTGTTGTGACAATGGGTTCTTCACGATCTAGCATTCTTGGTGAGGCTAGCATTAACCTTGCTGATTATGCTGATACATCAAAGCCTTCTGCTGTTGCACTGACTCTTCATGGATGTGACTCAGGGACTATTTTACATGTTACTGTACAGCTGCTAACTTCAAAAACTGGATTCAGAGAGTTTGAGCAGCAGAGGGAACTCAGAGACAGGGGGTTGCGGACCACTGACCAAAATAGTAATGATGAATCTGGTGGTGGTGAAGTATCATCTTCTGAAGTGACTGTCAGTGATCAGATGGATAAGGTCAGTGCAAGAGTTGGATTCAAACCAGAATCTAAAGAGCTTCCTGCACTCCGAGAAGGTGCGGGGTTGAATGAAGAATATACAGACTCAGCTGTTGGATGTGATGGCTCGTCCAATACTTCAGAGAGTTTATATGCCGAAAAGCATGATGCATCGAGCACACATGAAATTGACAGCCTTAAGAGTACAATATCTGGTGATCTAGGTGGGCATTCCCTTAATCAAAGCCCTCGCTTGGAGAAAGGAGATCCACCTGATCATCGTTTTTTGGGACAGGGTAATAATGACTGGGTTCATGGCTGGGGTTCAGACTATTCAGTGGATAATGACTTGGCGATTGCTAATGAAGAAAATAGTAGACTCAGGGGAAGCTTGGAAGTGGCTGAATCGTCCATTTATGAACTTAAGTTGGAAGTAAACTCTTTACAAATTCATGCTGATGAAATTGGTCTTGAAACACAAAAGTTTGCCCACCATCTTTCTCTCGAGGTTGCTTCAGGAGAAGAATTGGCAAAAGAAGTTTCTGTACTTAAATCGGAGTGTTCAAAGTTGAAAGGTGATCTTGAACGGCTTAAAAGTTTCAAATTAAGCTCTCTATTTTCCAGCACGGAAACTATTGAAACAGACCAAAATCAGTTATTCCAAAATTTACAGCTCAAGTGGCTCAAGGGGTTGATGGTTATGGAGGATAAAGTACTAGAGCTTCAAAACAAGGCATGCCTTGAATTCCATGAAACAGACTTGAGGTTCCTTCACCCAGATTTAGAGGTATTGCTTGGTGTGGTGCAGAATCTCAAACAAGGAACTGGACAAGCTTTTTCTGTACTCAACAAAGTGAAATCAACAACAGAAAATGTAAAGGAGATTGGGGAAATGAATATACACAAAAGTGAACAATTTGGTTCGGGAATCGGGTTTGATTCAGACTTGTACCAACCTGACAGTATGCTTCACTGTTTAAACATACCTGGGTTGGCATCTCAAGAACCTGATTCTGTTGATGCTACCGATGCAATGAAAGGGAAAATCTTTGAACTTCTAAGGGAGTTGGATGAGGCCAAAGCTGAACGGGAAAGCCATGCAAGAAAAATGAACCAGATGGAGTGCTACTATGAAGCTCTCATTCAGGAGCTTGAGGAAAACCAGAAGCAGATGCTGGGAGAGTTGCACAATCTCAGAAATGAGCATTCTACTTGCCTTTACACACTGTCATCCTCTAAAGATCAGATGGAAGCAATGCACCAAGAAATGAATGAGCAAATCTTAAGATTTGCCGAGGACAGACGGAACTTGGATGCCCTTAACAAGGAGCTTGAAAAAAGGGCTATTACTTCAGAAGCAGCGCTTAAAAGAGCACGCTTAAATTATTCCATCGCAGTAGATCATTTACAGAAGGACCTTGAACTGCTTTCTTTCCAGGTTTTGTCTATGTTCGAAACTAATGAGAACCTCATTAGGGAAGCTTTTACAGAAACTTCACTACCATGTTCTCAAGTGTACCTGGAAACGGTGCAGAAGCAGAACCAGCATTTTCAGGGTACAGATGCTTCCAAACTCTTGCATTGTCAGAATCGAAGTCCTGCTGCAAAGAAAGAACTTTTGGGGGGAGATGTGCTTTTGGAGGACATGAAAAGATCACTCCACTTGCAGAAGGAACTTTACCAGAAGATTGAAGATGAACTCTTTGAAATGTATTTTTTGAATTTGCACTTGGATGTGTTCTCAAAGACACTACAAGAAAGTTTGCAAGAAGCAAGTGCCAATGTTCGAAGTACGAAAGACAAAATGGGAGAGCTTACACAGAAGCTAGAGCATTCAATCCAGTCAAAGGAGTTGTTGACACGAAGGCTACGTGCTGCTATGGATGATGTTGATGCACTGAATGAGTACAAGGCTAGTTGCGTTTCGAAGGGCAATGACATGGCTCTGCATAATAAAATTTTGGAAGAAAATTTAAGAAGTGTTGCTAGTGAAAATTGTTTTCTTAACCAGAAGATCACAGAATGGGAACCCTTAGTGATGGAATGCAGAAATTTTGAAAGAAAATATGAGGGTTGTGCTGCTGAGAAAAAAAAGATGGAAAATTTATTACAACAGGAAGCTTTGGAGAAAAAAAGTCTTCGAGATGAAATTTTTGCTTTGCGGGAACAGTTAAAAAGTGTTAAAACTGAAAGCGATGAACTGGCGTCTGTGAAGGACCATCTGCAGAGAAGTGTCAACCTTTTGCAAGATAAGTTGGGTACTCTATTGGCAAATTATGACAGACAGTTCAAAGGATTGTCTCTCTGTAGTAAACCTCTGTGCTCAGGTTTGGATTTCAAGGACCTCATGGGTGTTGTGTCCCAAATGGAAGAATATCAACATATCGCATGCGGAACGATTCTTCACCTCATGGAAGAGAAGAAAGATCTGGAGAATCAAAGAGATACTGCTCAATCAGAAATTCTGGTCATGAAAGAAAAGTTTAAAGACGTACAAGATACAGTGAATAAAGTAGATGTGTCTAATGTCCTGGTGCAAAAGCTTCAGTTGAATCTTGATGCTTTCGCTAACAGACTTCAGATTAGCTCTGAAACTGAAGAAAAATATGCACACCAGAAGAATGAGGTTTTTTCTGTTCTTGAGCACTTGGAAGTTGAGCTGCAACAACTTACATCTAAGAACAGGGACCTTGCTGAAGAAATCCAAGCATTGACGACTGTAACTGAGGAACTTGAACGGAGTAAGTTAACCATTTCAGAATTTGCGCAAAAAAACCAAGCTTTGATGTTGTCTTTACAGGAAAAAACTGATGAGTCTGTCAATCTTGCTTCCGAGCTTACTTGTTTAAAAGAAAGTTTGAGAAGTCTGCATGATGAGTTACGTGTTGAAAGGGGCACCAGAGATAAAATAGAGGACTCAGTTACAGAACTCACGTTGCAGTTGGGGACTGTAAGTGAGGAACTTGGAAGGAGTAAGTTAACTATATCAGAATGTGTGCAGAATAATCAAGCTCTGACCATCTCTTTACAGGAAAAGACTGAAGAGTCTGTCAATCTTGCATCGGAGCTTACTTGTTTAAAAGAAAATTTGAGAAGTCTGCATGATGAGTTACGTGTTGAAAGGGGCACCAGAGATAAATTAGAGGACACAGTTACGGAACTCACACCTCAGTTGGGGACTTTAAGTGAGGAACTTGGAAGGTGCAAGTTAATTATATCAGAATTTAAGCAGAATAATCAAGCTTTGACCATCTCTTTACAGGAAAAGACTGATGAGTCTGTCACTCTTGCATCAGAGCTTAATAGTTTAAAAGAAAGTTTGGGATGTCTGCATGATGAGTTGCATGTTGAAAGAGGCTCCAAAGATAAATTTGAGGACATGGTTACAGAACTTATGTCCCAGTTGGGGACGATAACTGAGGAACTTGGACAAAGTAAGTTGATCATAGCAGAATTTGCGCAAAAAAACCAAGCTTTGATGACAAGATTACATGAAAAAACTGATGAGTCTGTCAATCTTGCAACGGAGCTGGATAGTTTAAAAGAAAATTTGAGATGTCTTCACGATGAGTTGTCTGTAGAAAGATGCACCAGAGACAAATTAGAGGGCAGGGTTACCGAACTTACATCTCAGTTAAATGAAAAAAATAATCTGTTGCTCCATTTTGACCTGCAGAAGGCTGAACTGGTTCATTTCAAGCAGCAAGTTGCAGGTCTAGAATCAGAAAAATCACAATTAGATCTTCGTTTGCTGCACTCTGAGGCATGTCTGGAGAAGGTTTATGAAGAATCTTTTTCAGCTACTGGTCTGGAATCTCAGTTATCTGAAATGCATGAACTTTTAATAGCTGGGGATGTTAAGATTGTTTTCACAAAAACTCAGTACGAGGGATGGATTGAGGTATTTCTTCAGCAACTTGAATCCTCAGCTAGGCACCTTGAGGAGTTTCACCAGAAGAATCTTAATGTAGAGACAGCACTAAACCGTTGCCTTGCAAGTGAAGCACATTATGTTGAAGAAAATGCAAGATTGATGACTACTATCGAATCCCTTAGATCTGACTTGGAAGCTGCCGTAGCCCAAAATAATGTCCTCTTGGATTCAAACAGTGCTATAACAACTGACCTCGAGGAATATAAGAAGAGGGCTGCAATTTTGGAGGGTAGTTATTTGAAAGACACAAAAAAGCACACTCTTGAGGTTCAGCAGCTGAAGCACACACTGGCATGTTCTGAAGAAGAGATTGAGAACCTGATACTCTTCAAGGAAGAACTGGAAATCAAAGTTGTAGTACTGACTTCCAAATTAGAAGGATGTATTGATGAACTGTTGACGCTGCGAAAGCAGTGTAATGAGCTTTCTAACAGGCTCTCTGAACAAATGTTGAAGACAGAAGAATTCCGAAATTTGTCCGTCCATTTTAAGGAGCTTAAAGACAAGGCTGATGCTGAGTGTCTCCAGGCTCGTCAAAAAAGAGACCCTGAGGTACCATCAGTTGCCGTGCAGGAGTCCTTGAGAATTGCCTTTATCAAAGAACAATACGAAACCAAGTTGCAAGAGTTGAGACACCAGCTTTCTATTTCCAAAAAGCACAATGAGGAGATGCTATGGAAATTACAAGATGCCATTGATGAAGTTGAGAACAGAAAGAAATCCGAAGCTTCTCATTTAAAGAATAATGAAGAGCTGTTGTCGAAGAAATTGGAATTGGAGGCTGAGTTACAGGCAGTAATTTCTGACAAACGTGAAAGAATCATGACTTATGACCAAATTCAAGCTGAACTGGAGTGCTCATTAATAAGCCTTGAGTGCTGCAAGGAAGAAAAACAAAAGCTTGAGATTTCCTTGCAAGAGTGCAACGAGGAAAAGGCTAAAATTGCAGATGAACTTATCTTGATGAAGGAGCAGCTGCAGAAATGGAACTCTACAATAGATACTCAAAAGGAAGGCCTTAATGGCTCACATAAAGTGGGGAGCATTTCTGGTAACCCAGTTATAGAAAAGACTCGAGAGGAAGTTTCAGTTGCAGGTACTTGGAATGGTAATGACTTGGTCCAAGATGGCTCAACGACTCGTGAAATAGAAGATTTATGCTCATTTCCCATTGATGTGAAACCCAAGCAGGATGTTTTTGTATCTAGAGGGATAGATGGAATCCAAGGACATTCATTTGTAGATCAAGAGAACTTGCTTGATGGTGATACAAAGCAATTAGCACTCATCAATGATCACTTCAAAACTAAAAGTTTGAGGTCTAGCTTGGACCACCTACATAATGAGTTGGAAAGGATGAAAAATGAAAACTCGCTTCTTCCACACGATGATGATCATTTTGATCCATGTTTTCATGGTTTACAAAGCGAATTGATGCAATTACATAAGGCAAATGAAGAATTGAGATGCGTATTTCCTTCTTTTAACGAGCTTTCAGATAGCGGGAATGCATTAGAAAGAGTACTTGCTTTGGAAATTGAGCTTGCTGAAGCATTGCAGGCAAAGAAGAAATCAAGCATACATTTTCAGAGTTCATTCTTGAAACAACACAATGACGAGGAAGCAGTATTCAAGAGCTTTAAAGACATAAACGAGCTGATCAAAGACATGTTAGAGGTAAAGGGAAAGTATGCAGTTGTGGAGACGGAACTAAAAGAGATGCACGACCGTTACTCTCAACTGAGTCTCCAGTTTGCAGAAGTAGAAGGAGAGAGGCAGAAACTCATGATGACACTCAAGAATGTTCGCGCGTCAAAAAAATTACTGCAGTTAAATCTATTCTCTTCGGCTACTCTCGGGGAAAATTCATCATAATAGTCCCTTTTTACCTGCTAAAGATGAAATTATTACGAAAAAAATTGGCCTGCAAGTCCAATGACAAAAAAATTTCAAGCTTTAGTAAAAGATGTCATGTTTTGTGTAAATAAAAAATGCGTTTACCGATTATTATCGGTTTTCAATATTAGAATGTAGATCCATGTAATTTTTAGGGTGGTGTAATGTAATTCCTTCTGCTGTATACTGTATAGCCAATGAAATAATTTAAAATTCCCATTTTGGGTTTTGTTCAAAAAAAAAAAAAAAAAAAAAAAAAAAAAAAGT

>transcript_27 full_length_coverage=2;length=6954

GGGGAGTTTCTCTCGCCTCTCTCTTAAAACACATATACACACTCTCTCTCTAAAAGCGTCAACTCCGTCGGATCTGCAGTCTGTACGAGTTATTTCGAAGCAGATCAACTGAATTTCAACCCACTGTGATCTAGGGTTCCAAAAGACGTCGTCTTTCATGAAGAATTGATGCTTTCACTTAGCTCCAAATGTTCAAGACTTCCTCACATGACTTTGTTCTTCTGGTCTACCAAACACCAATTTTCGCTCTTTATACTCGCATCAGCTTAAGGGTTTGGTCAATTTCATAAACCATTAAACCCTTTGGGGTTTCTGGTTTTGTATGCAGGAGCCAAGTGATTAAATCTAGGGTTTGCTGCCATAGAGTAAAATTGGGGTTATATTAAGCTTTAGTTATAGGTTTGCTGGTATAGAGTAAATTTGGGGTTATATTGAGCTTTAGTTTATATAGTTTTGGGAGGTAGAATTGTAAAGCTAGGTTTTTTTGGAGAGCTTTTGTTGAGATTTTGGATCAAGTAAGGTAGAAGATAGAGAAACATGTTTTATTCTCAATTTATATTGGCAAAAAAAGGGCCACTAGGGACGATATGGATAGCTGCACATTTGGAGAGAAAGCTTCGCAAGAATCAGGTTGCAGATACCGATATTGGCGTGTCCGTAGATTCAATTCTTTCCCCTGAAGTACCCATTGCACTCCGGTTGTCCAGTCATCTTCTGCTTGGTGTAGTCAGGATATATTCTAGAAAGGTTAATTACCTTTTTGATGACTGCAGTGAGGCTTTGCTTAAGGTGAAGCAAGCTTTTCGCTCCACTGCAGTTGACTTACCTCCAGAAGAATCCACTGCACCATATCACTCTATCACCTTGCCTGAGACTTTTGATCTTGATGATTTTGAGCTGCCAGATAATGAAAATTTTCAGGGCAACTATGTTGATCACCACATCAGTACAAAGGAGCAGATTACGCTCCAAGATACTATGGAGGGTGTTGTTTTTTTCAACATCTCAGTTTGGACTGGATGAGCAATTTGGCGATGGTGACGCTTCTCAAATCAGTTTCGACCTTGATGAGGACTTGTTTCTTGACAAGGTTGCAGCTCCAAGACATACCGGAGTTATGTTGGATTCAAAGGATGATCCCCAGGCATCTGTACAGCCAATGACATCCCTTGACAATTATGTAAATAACGCGTGGACGATTGATACCTCAGAAGCCATGCCAGTGAATGGTCGTGGAGAACAGCATAAAGGCCTAGTTGCAAATAATGATATCATCGAGTCTGCCCAGGCCCCATCTACTCCTGGATTAATTGAAGAGCCAAATTTATCCAATATGCAAATGGGCCTAGCTTGCGATGATCATATGGAGTTAGAAAACTACAATTCAATGGAATTTGAAGCAAGAGAAGCCATAGGAAATTCTTTCAGTAAATCAGATCATCACGGGGATAAAAAATGTGTGGATGGGCCTTTGTCCAGTATCGATATGAATATTGATTGCATGCCTGTTCAGGAAAATGATCATGTTATGGGCGAGCCCGTGATCTCACAAGCCAACCCACAAGGAGATTTACCGTCTACGTCTGAAAATGCGGGGAAAATTGAAGTGCAAAACTTCTCCAATATACAAATGACTATGGCTTGCAATGATCTTATTGAGTCAGAAGATCATAATTCAATAGAACTCGAAGTGAGAGAGACCGCAGGAAGTTCTTCAAGTAAATTAGATCATCACGGGCATAAAAATTCTGCAGAGTGGGCTTCGTTCAATATAGATATGAATCTTGATTGCATGCCTGTTCAGGAGAATGATCATGTTATGGGTGAGCAGGAGATCTTACAAGCCGAGCCACAAGTAGATATACCATCTACTTCTGATACTATGGAGAACATGTTGGATAATCCTCTTTTAGCATCTTTACCATCTCCGGTTCTAACTGAGAAAGAAAATTCTGTTTCTGTAGTGTCAGAGTTTCAGGGTAGGATTACTGCACCTGATGACATGGACAGTGTGGGGGATTTACAAAATGGAGTTCTGAGCAAAACCGAACAAAGTACAACGTTTACGGGTCAAGACCATTTGGAATGCACGGAATCCTTAGGAGTTAGATTGGAGGAAACTGTAGCCTCTCCTAGTTTTTTCCCTTGGGACCTCTGTTTTGGAGGATGCTCGTCATAAACCAGGTTCTGGTGTTGGACCAATTTCTGAGAAGTCCAGTTTAACCAATACCTATCCTCTAGTACCTGGAGGTATTTCTGGGAGTGATTCAGCATTGCTTCAGCCAGAGATTTCTGATAGTGTAGAGATAGCTGGAAACATGGAAACATGCCCCCCTAATGCACTGGTATCTGTCTGTTCTTTGAAGTCTCCTACTGGACCCGGGCTTGAGAATGTCGAGGCTTCGGCTGCTCATGAACCAAAACATTCTGAGACTTCGAATCCCAACCAAGAAGGGCCATCTACTTGCATCCATGTACTTCAGCAAGCATGCAACTCACTTGAAAACGAACCTGTTAAATCATTTTTTGGAGGTAACAATTCTATAGTTGTTCCTGACATACCCTCTGAAGTTTTATTGGAGGGTCAGGCTGGTCATAAACCAAAAGATCCTGAGATTATGAACCCTGATGAGGAAGGGCCATCTACTCGCATCCATGTACTTCAGGCTTGCAATTCAATTCAAAACCAACCTGTTAAATCATTTCTTGGAGGTTACAGTTCTGTAGTTCCTGACTTACCTGCTGAGACATCTGAATGGGAGGCAACCCATATTTTGGGAGCTTCAACTGAGGTGCAAGCAAATTTTGATGGTACCCCTGGGCACCAGCCAGCACCAGGGCAAGCTGTGCTACCAGCCAGTGATCAGCACCACGAGGCCAGCACCACAAGGCTCCCGCAGCAGTTGCCACAGGAGCCCATAGCCGCCGCCAGCTGGCACCCTACAGAGAATTTTTTTGACGGGAGCACTGGCCATCAGTCAGCACCATGGCAAGCTGCGGAACCAGCCAGCAACCAGCAACACCAGGACACCAGTAGCACCAGGTTGCAGGTGCCAACAGCCACCACCAGCTCCCCCACAACAGATGTAGATAATTTATTTAAGGGGACCACTGGCCATCAGTCAGCACCATGGAAAGCTGCGGTACCAGTCAGCGATCAGCAACACCAGGATGACAACACAAGGCACCCCCAGCAGCAGCTGTTGCAGGACATCAGTAGCACCAGGTCACAGGCGCCAACAGCCACCACCAGCTCCCCCACAACAGATTTTTTTGATAGCACTACCGAGCACCAACCAGTATCATGGCAAGCCGTGGCATCACCCAGTGACCACCAACACCAGGTGGACACTGCAAGGCACCTGCACCAGGGCAGCACCAGGCTGCAGGCGGCCACAATCACCACCAGCTGTCCCCCGGCAGTGACTTTTTTTGACGGTTCCACTGGGCACCACATGGCACCATGCCAAGCTGCGGTACTAGCTGGCAACCAGCAACACCAGGACGACACTGCAAGGCAGCTGGGACAGAGGTCGCAGGACACCAGCAGCACAAGGCCTCAGGCGCCCTCAACCACCACCACCAGCTGCTCCCCAACAGCAATCTTTTTTAATGGCTCCACTGATCACCAACCGACACCATGGAAAGCCGCAGCACCAGCCAGCGACCAGCAACACCAGGCTGACACCGCAAGCCACCTGCACCAGCAGTCACTGGACACCATCAGCACCAGGCTGCAGGTGCCCACGACCACCACCAGTTGGCCCCCAACAGTGAATTTTTTTGATGGCACCACTGGGCACCAACTGGCCCCATGGCAAGCTGCGGTACCAGCCAGCGACCAGCAGCACCAGGCCGACACCCCAATGCACCTGCAGTCGCAGTCGCAGGACACCGGCAGCACCGGTTTGCAAGCTCGCACAGTCACCACCAACTGCCCCCCAACAGCAAATATTTTTGATGCCACTGCTGGGAACCAGCCAGCACCATGGCAAGCTGTGGTACAAGCCAGTGACCAGCAGTGCCAGACCGGCTCTCCAAAGTACCAGCTGCAGACTCCTGAGGACACCAGCAGCACCAGGCCGCAGGTGCCCACAACTACCACCAGCTGGCGCCAAACAGGAGAAGGCTACCGTGCAACTGATATGGGCCAACCGTCCTTAGACGTGAACCAGTTATTGGAACCAAATTCTTGTCAGTATGTTGACGCAGGTCGCAGGAAGTTGGATGAGCAACTAGCGAATGCCAAAAATACTGTTGAGAATTATTTTGGTTATGCCGATTTGCCTGCACCTGAAAAAATGCTTTCTGTTCCAGAAGGGCGTAGCCATCTAGGGAATAATTTTTTGTTGGAGTCTACTCCGGGCAAAGAAGCCACAATGCATGGCGAAGGAAATGGCACTGGGGTCCAAAACATTACTGGAAATAAGCGTAGTTATATGGAAAGTATTCTGACTACGCAGAGCCTGAATTCGGTTGAACCATTTGAGAAGTCCCGATCTGAAAGAACTACGGGATCTGTTCCTGATGATGATGATTTGTTATCTTCCATCCTAGTTGGAAGAAGGTCTTCAATTTTGAAAATGAAGCCCACTCCACCTCCATCTGAAACAGTGTCTGTGAAACGCCGTCGAACTGCACCCCGGGCCAGTGCATCTAAGAGGAAGGTGTTTATGGATGATACAATGGTCTTGCATGGCGATATAATACGACAACATTTGACAAATACTGAAGACATACGTAGAGTACGGAAGAAGGCTCCTTGCACTCGACCTGAAATTTGGATGATTCAGAATCAATTCTTGGAGGATGAAATTTTCGGTGATCCCATATTTAGTGGTATGTCGTCAGAATTGATTTGTTCACTCGGTCAAAGATATGATCCGAGTATAATTAGGGTTTCTCAGAATGATGAAAGTATAGCTTTGTTTGGAGCAGCAAAGAATGGAGAGCTTTCTATGACACCAAATGTTATCAATGAAACTGAACCAGAAAGGATCATTGAATCTGAAATAACCAGAAATGACGGGGAAGTTCAATCTGCCGAGACTGTTGTCCAAACTGAGGTTCAGCATCTTGATAATGATACTCAAATGCAATTGAATGCTAATACTACGATGCCTGAGCTTAGGACTTTGCACCCGGAAACAGATGGGATCACTGAACCTATAATAGTCAGAAATGACGGGGAAGCCCAACCTGCCATGGCTTTAGTTCAGACCAAGGACCAGCATAATTATATTGCTACTCAAATACAAATCAATTCTAATACTATAATACCTGAGCTCAAGTCTTCACACCCTGAAACGGAAGGGATCACTGAACCTATAATAGCTAGAAATGACGGTGAAGCCCAACCTGCTAATACTTTTGTCCAGACGGCAGAGCTGCATAATTACAATGATACTCAAATGCAAATGAATGCTAATACTGTGATACCTGAACTTGGCACTTTGCAACCCGAACATATGGGCAAGATAGCTGAAATGGAATTTGCTAGAGGTGGTTCTGCAGTTGCTAATGCAGAGGAGACTTCTGTCGCTCTTGGGATTGGATTATCATCCCCAGTTGATTTAGTTTCTGTAGATATTTGTAACTTGTCAACTGATCCTGTGGAGAAACCCAGTGGTTCTCCACAGATCGCTGTGGCTTGCTTGGTTCCTGACCAGAAGTTGGATTCTCAATCTGTTGAGAAGGATGCTTCTGTAGTGGATTTATGCATTGAGAAAGGAGTTGAGCCTGTTGAAGTTTCTGAAATTCAAGTCGTAGTTGGTACTGAATCTTGTGCAAGTTTTGAGAAAGATGCTTCTGCAGTAAATCTTGGGCAAGGAGTTGAGTTTATTGAAGTTCCAGAACTTCATGACGGCGATATTATTGGAGCTGGGACTGAATCTGGTGCGACAGGTGAGTTTCTTTTGGAAGAAAACAGAGGCGGTCTTTCAGTTGAAAACAGAACAGATAATCCTACCGACAGTGTAGATCTTCCCCTTGAAACCGTCTCTCCAGCAATTGACTTGGTTGTTGTGACTGAAGACCAAGCTGAGGAGGAAACTGCTAAAATCGAAGAAGGGGTTGTAAATGAGGCTACTGAAAAGGGGTATGATGGCAGGGATCGAACCTCTTACGGTGTATATAGCCAAGAACCTAAAACTGATTATTCCCTGAGTGACGGACAAGATGCATCCTTGAATAACGTTGAAAAATCTGGTGGCCAGGAGGTTGTACCACAAGATGTAAGAGATGCCGAAACTGCTTCCTTCGACCATGCTGATGTTGAAATCCATGATGATGTGGAAAATGAAACAGTTGAGCATGATACTGAGTTCCTCAACTTTGATAACGACGGTGATGATGATGGCGGAGCTGACGATGACTATGATGACTGTATGCCTTGTCCAGAAGAAACCCGCATTCTTGAGAACAGCGGATGGTCTTCCCGTACCAGGGCTGTTGCCAAGTACCTCCAAACCGTATTCGACAAAGAAGCTGACCACGGTAAAAAAGTTATTCCATTGGACAACCTTTTGAGTGGCAAGACTCGAAAGGAAGCGTCGAGGATGTTTTTTGAAAGTTTGGTTCTTAAAACAAGGGATTATATACATGTAGAACAGGAAAACCCTTTTGATAACATTATTATAAAGCCTCGAGTAAAGCTCTTGAAATCGGAGTTCTGATATATCCTACATAATTATCCGCCCGAGTTTGGGTATTCTTCTTCATTTTGGTGTGTATTTTTGTTTATTGTTTTGTTTAGTTGTTGTAACTATTAGTTAAAAGTGGGGTCATGTTTTATCCCCAGTTGTTGTAACAGTGTTGTAACATTAGGCTGCATTTATATGTGTACATATATGTAACTGTAATAATGTGATTCACCATTTGCAATTAAAGATGCTAATCAATTTATTTGTTAACCTTTGTGCAAGAAAAAAAAAAAAAAAAAAAAAAAAAAAAAGT

>transcript_28 full_length_coverage=6;length=6931

GGGGAGTTTCTCTCGCCTCTCTCTTAAAACACATATACACACTCTCTCTCTAAAAGCGTCAACTCCGTCGGATCTGCAGTCTGTACGAGTTATTTCGAAGCAGATCAACTGAATTTCAACCCACTGTGATCTAGGGTTCCAAAAGACGTCGTCTTTCATGAAGAATTGATGCTTTCACTTAGCTCCAAATGTTCAAGACTTCCTCACATGACTTTGTTCTTCTGGTCTACCAAACACCAATTTTCGCTCTTTATACTCGCATCAGCTTAAGGGTTTGGTCAATTTCATAAACCATTAAACCCTTTGGGGTTTCTGGTTTTGTATGCAGGAGCCAAGTGATTAAATCTAGGGTTTGCTGCCATAGAGTAAAATTGGGGTTATATTAAGCTTTAGTTATAGGTTTGCTGGTATAGAGTAAATTTGGGGTTATATTGAGCTTTAGTTTATATAGTTTTGGGAGGTAGAATTGTAAAGCTAGGTTTTTTTGGAGAGCTTTTGTTGAGATTTTGGATCAAGTAAGGTAGAAGATAGAGAAACATGTTTTATTCTCAATTTATATTGGCAAAAAAAGGGCCACTAGGGACGATATGGATAGCTGCACATTTGGAGAGAAAGCTTCGCAAGAATCAGGTTGCAGATACCGATATTGGCGTGTCCGTAGATTCAATTCTTTCCCCTGAAGTACCCATTGCACTCCGGTTGTCCAGTCATCTTCTGCTTGGTGTAGTCAGGATATATTCTAGAAAGGTTAATTACCTTTTTGATGACTGCAGTGAGGCTTTGCTTAAGGTGAAGCAAGCTTTTCGCTCCACTGCAGTTGACTTACCTCCAGAAGAATCCACTGCACCATATCACTCTATCACCTTGCCTGAGACTTTTGATCTTGATGATTTTGAGCTGCCAGATAATGAAAATTTTCAGGGCAACTATGTTGATCACCACATCAGTACAAAGGAGCAGATTACGCTCCAAGATACTATGGAGGGTGTTGTTTTTTCAACATCTCAGTTTGGACTGGATGAGCAATTTGGCGATGGTGACGCTTCTCAAATCAGTTTCGACCTTGATGAGGACTTGTTTCTTGACAAGGTTGCAGCTCCAAGACATACCGGAGTTATGTTGGATTCAAAGGATGATCCCCAGGCATCTGTACAGCCAATGACATCCCTTGACAATTATGTAAATAACGCGTGGACGATTGATACCTCAGAAGCCATGCCAGTGAATGGTCGTGGAGAACAGCATAAAGGCCTAGTTGCAAATAATGATATCATCGAGTCTGCCCAGGCCCCATCTACTCCTGGATTAATTGAAGAGCCAAATTTATCCAATATGCAAATGGGCCTAGCTTGCGATGATCATATGGAGTTAGAAAACTACAATTCAATGGAATTTGAAGCGAGAGAAGCCATAGGAAATTCTTTCAGTAAATCAGATCATCACGGGGATAAAAAATGTGTGGATGGGCCTTTGTCCAGTATCGATATGAATAATGATTGCATGCCTGTTCAGGAAAATGATCATGTTATGGGCGAGCCCGTGATCTCACAAGCCAACCCACAAGGAGATTTACCGTCTACGTCTGAAAATGCGGGGAAAATTGAAGTGCAAAACTTCTCCAATATACAAATGACTATGGCTTGCAATGATCTTATTGAGTCAGAAGATCATAATTCAATAGAACTCGAAGTGAGAGAGACCGCAGGAAGTTCTTCAAGTAAATTAGATCATCACGGGCATAAAAATTCTGCAGAGTGGGCTTCGTTCAATATAGATATGAATCTTGATTGCATGCCTGTTCAGGAGAATGATCATGTTATGGGTGAGCAGGAGATCTTACAAGCCGAGCCACAAGTAGATATACCATCTACTTCTGATACTATGGAGAACATGTTGGATAATCCTCTTTTAGCATCTTTACCATCTCCGGTTCTAACTGAGAAAGAAAATTCTGTTTCTGTAGTGTCAGAGTTTCAGGGTAGGATTACTGCACCTGATGACATGGACAGTGTGGGGGATTTACAAAATGGAGTTCTGAGCAAAACCGAACAAAGTACAACGTTTACGGGTCAAGACCATTTGGAATGCACGGAATCCTTAGGAGTTAGATTGGAGGAAACTGTAGCCTCTCCTAGTTTTTCCCTTGGGACCTCTGTTTTGGAGGATGCTCGTCATAAACCAGGTTCTGGTGTTGGACCAATTTCTGAGAAGTCCAGTTTAACCAATACCTATCCTCTAGTACCTGGAGGTATTTCTGGGAGTGATTCAGCATTGCTTCAGCCAGAGATTTCTGATAGTGTAGAGATAGCTGGAAACATGGAAACATGCCCCCCTAATGCACTGGTATCTGTCTGTTCTTTGAAGTCTCCTACTGGACCCGGGCTTGAGAATGTCGAGGCTTCGGCTGCTCATGAACCAAAACATTCTGAGACTTCGAATCCCAACCAAGAAGGGCCATCTACTTGCATCCATGTACTTCAGCAAGCATGCAACTCACTTGAAAACGAACCTGTTAAATCATTTTTTGGAGGTAACAATTCTATAGTTGTTCCTGACATACCCTCTGAAGTTTTATTGGAGGGTCAGGCTGGTCATAAACCAAAAGATCCTGAGATTATGAACCCTGATGAGGAAGGGCCATCTACTCGCATCCATGTACTTCAGGCTTGCAATTCAATTCAAAACCAACCTGTTAAATCATTTCTTGGAGGTTACAGTTCTGTAGTTCCTGACTTACCTGCTGAGACATCTGAATGGGAGGCAACCCATATTTTGGGAGCTTCAACTGAGGTGCAAGCAGCGAATTTTGATGGTACACCTGGGCACCAGCCAGCACCAGGGCAAGCTGCGCTACCAGCCAGTGGCCATCAGCATGAGGCCAGCACCGCAAGGGTTCAGCAGCAGTTGCCACAGGAGCCCATAGCCACCGTCAGCTGGCGCCCTACAGCAAATTTTGATGGTACCCCTGGGCACCAGCCAGCACCAGGGCAAGCTGTGCTACCAGCCAGTGATCAGCACCACGAGGCCAGCACCACAAGGCTCCCGCAGCAGTTGCCACAGGAGCCCATAGCCGCCGCCAGCTGGCACCCTACAGAGAATTTTTTTGACGGGAGCACTGGCCATCAGTCAGCACCATGGCAAGCTGCGGAACCAGCCAGCAACCAGCAACACCAGGACACCAGTAGCACCAGGTTGCAGGTGCCAACAGCCACCACCAGCTCCCCCACAACAGATGTAGATAATTTATTTAAGGGGACCACTGGCCATCAGTCAGCACCATGGAAAGCTGCGGTACCAGTCAGCGATCAGCAACACCAGGATGACAACACAAGGCACCCCCAGCAGCAGCTGTTGCAGGACATCAGTAGCACCAGGTCACAGGCGCCAACAGCCACCACCAGCTCCCCCACAACAGTGACTTTTTTTGACGGTTCCACTGGGCACCACATGGCACCATGCCAAGCTGCGGTACTAGCTGGCAACCAGCAACACCAGGACGACACTGCAAGGCAGCTGGGACAGAGGTCGCAGGACACCAGCAGCACAAGGCCTCAGGCGCCCTCAACCACCACCACCAGCTGCTCCCCAACAGCAATCTTTTTTAATGGCTCCACTGATCACCAACCGACACCATGGAAAGCCGCAGCACCAGCCAGCGACCAGCAACACCAGGCTGACACCGCAAGCCACCTGCACCAGCAGTCACTGGACACCATCAGCACCAGGCTGCAGGTGCCCACGACCACCACCAGTTGGCCCCCAACAGTGAATTTTTTTGATGGCACCACTGGGCACCAACTGGCCCCATGGCAAGCTGCGGTACCAGCCAGCGACCAGCAGCACCAGGCCGACACCCCAATGCACCTGCAGTCGCAGTCGCAGGACACCGGCAGCACCGGTTTGCAAGCTCGCACAATCACCACCAACTGCCCCCCAACAGCAAATATTTTTGATGCCACTGCTGGGAACCAGCCAGCACCATGGCAAGCTGTGGTACAAGCCAGTGACCAGCAGTGCCAGACCGGCTCTCCAAAGTACCAGCTGCAGACTCCTGAGGACACCAGCAGCACCAGGCCGCAGGTGCCCACAACTACCACCAGCTGGCGCCAAACAGGAGAAGGCTACCGTGCAACTGATATGGGCCAACCGTCCTTAGACGTGAACCAGTTATTGGAACCAAATTCTTGTCAGTATGTTGACGCAGGTCGCAGGAAGTTGGATGAGCAACTAGCGAATGCCAAAAATACTGTTGAGAATTATTTTGGTTATGCCGATTTGCCTGCACCTGAAAAAATGCTTTCTGTTCCAGAAGGGCGTAGCCATCTAGGGAATAATTTTTTGTTGGAGTCTACTCCGGGCAAAGAAGCCACAATGCATGGCGAAGGAAATGGCACTGGGGTCCAAAACATTACTGGAAATAAGCGTAGTTATATGGAAAGTATTCTGACTACGCAGAGCCTGAATTCGGTTGAACCATTTGAGAAGTCCCGATCTGAAAGATCTACGGGATCTGTTCCTGATGATGATGATTTGTTATCTTCCATCCTAGTTGGAAGAAGGTCTTCAATTTTGAAAATGAAGCCCACTCCACCTCCATCTGAAACAGTGTCTGTGAAACGCCGTCGAACTGCACCCCGGGCCAGTGCATCTAAGAGGAAGGTGTTTATGGATGATACAATGGTCTTGCATGGCGATATAATACGACAACATTTGACAAATACTGAAGACATACGTAGAGTACGGAAGAAGGCTCCTTGCACTCGACCTGAAATTTGGATGATTCAGAATCAATTCTTGGAGGATGAAATTTTCGGTGATCCCATATTTAGTGGTATGTCGTCAGAATTGATTTGTTCACTCGGTCAAAGATATGATCCGAGTATAATTAGGGTTTCTCAGAATGATGAAAGTATAGCTTTGTTTGGAGCAGCAAAGAATGGAGAGCTTTCTATGACACCAAATGTTATCAATGAAACTGAACCAGAAAGGATCATTGAATCTGAAATAACCAGAAATGACGGGGAAGTTCAATCTGCCGAGACTGTTGTCCAAACTGAGGTTCAGCATCTTGATAATGATACTCAAATGCAATTGAATGCTAATACTACGATGCCTGAGCTTAGGACTTTGCACCCGGAAACAGATGGGATCACTGAACCTATAATAGTCAGAAATGACGGGGAAGCCCAACCTGCCATGGCTTTAGTTCAGACCAAGGACCAGCATAATTATATTGCTACTCAAATACAAATCAATTCTAATACTATAATACCTGAGCTCAAGTCTTCACACCCTGAAACGGAAGGGATCACTGAACCTATAATAGTCAGAAATGACGGTGAAGCCCAACCTGCTAATACTTTTGTCCAGACGGCAGAGCTGCATAATTACAATGATACTCAAATGCAAATGAATGCTAATACTGTGATACCTGAACTTGGCACTTTGCAACCCGAACATATGGGCAAGATAGCTGAAATGGAATTTGCTAGAGGTGGTTCTGCAGTTGCTAATGCAGAGGAGACTTCTGTCGCTCTTGGGATTGGATTATCATCCCCAGTTGATTTAGTTTCTGTAGATATTTGTAACTTGTCAACTGATCCTGTGGAGAAACCCAGTGGTTCTCCACAGATCGCTGTGGCTTGCTTGGTTCCTGACCAGAAGTTGGATTCTCAATCTGTTGAGAAGGATGCTTCTGTAGTGGATTTATGCATTGAGAAAGGAGTTGAGCCTGTTGAAGTTTCTGAAATTCAAGTCGTAGTTGGTACTGAATCTTGTGCAAGTTTTGAGAAAGATGCTTCTGCAGTAAATCTTGGGCAAGGAGTTGAGTTTATTGAAGTTCCAGAACTTCATGACGGCGATATTATTGGAGCTGGGACTGAATCTGGTGCGACAGGTGAGTTTCTTTTGGAAGAAAACAGAGGCGGTCTTTCAGTTGAAAACAGAACAGATAATCCTACCGACAGTGTAGATCTTCCCCTTGAAACCGTCTCTCCAGCAATTGACTTGGTTGTTGTGACTGAAGACCAAGCTGAGGAGGAAACTGCTAAAATCGAAGAAGGGGTTGTAAATGAGGCTACTGAAAAGGGGTATGATGGCAGGGATCGAACCTCTTACGGTGTATATAGCCAAGAACCTAAAACTGATTATTCCCTGAGTGACGGACAAGATGCATCCTTGAATAACGTTGAAAAATCTGGTGGCCAGGAGGTTGTACCACAAGATGTAAGAGATGCCGAAACTGCTTCCTTCGACCATGCTGATGTTGAAATCCATGATGATGTGGAAAATGAAACAGTTGAGCATGATACTGAGTTCCTCAACTTTGATAACGACGGTGATGATGATGGCGGAGCTGACGATGACTATGATGACTGTATGCCTTGTCCAGAAGAAACCCGCATTCTTGAGAACAGCGGATGGTCTTCCCGTACCAGGGCTGTTGCCAAGTACCTCCAAACCGTATTCGACAAAGAAGCTGACCACGGTAAAAAAGTTATTCCATTGGACAACCTTTTGAGTGGCAAGACTCGAAAGGAAGCGTCGAGGATGTTTTTTGAAAGTTTGGTTCTTAAAACAAGGGATTATATACATGTAGAACAGGAAAACCCTTTTGATAACATTATTATAAAGCCTCGAGTAAAGCTCTTGAAATCGGAGTTCTGATATATCCTACATAATTATCCGCCCGAGTTTGGGTATTCTTCTTCATTTTGGTGTGTATTTTTGTTTATTGTTTTGTTTAGTTGTTGTAACTATTAGTTAAAAGTGGGGTCATGTTTTATCCCCAGTTGTTGTAACAGTGTTGTAACATTAGGCTGCATTTATATGTGTACATATATGTAACTGTAATAATGTGATTCACCATTTGCAATTAAAGATGCTAATCAATTTATTTGTTAAAAAAAAAAAAAAAAAAAAAAAAAAAAAA

>transcript_29 full_length_coverage=2;length=6892

GGGGTCACTTCCATTGCAACCAAAATTGGAGAGTACCTGGTTGCTCCAATCGGTCGTCAAATTGGTTATGTTCTTTTCTACAAGAGCAACGTTGGAGATTTCAAGAATCAAGTTAAGGAATTGGAAAATAAAAGAGTGGAGGTCCAATGGCGAGCTGCTGAAGCCGAAAGGAATGGAGATGTTATTCTACCAAGTGTTAAGAGCTGGTTAGTAACTGTGGAAAAGATCAAACAAGAGTCGAGTGAACTTGATGGTGAATTCCAAGTAACTAAACGGTGCTTCAACGTCCCTTATCCCAGTTTGCAGTCACGTTACCAAGGAAGCAAGAAAGCTAAGAAGTCCACAGCCGTTGCTCGTGAGCTCGAACAAAGTGGCAACTTTGAAAGAGTATCTCTTTCTAGTCATCCATCAGCAACACGGTTTAAATCAACTGAAGTGGCAGAAAATTTTGAGTCTAGAAGGCTGACTTTTAAAGAAATTAAGGAAGCAATCGAAGATGCCCAGATCAGTATGATTGGGATATATGGCATGGGTGGAGTTGGTAAAACTGTACTAGTGAAAGAAGTTGTCAAACAAGCTCAAGATGGTTTTCAATTTGAGGAAGTAGTTATGGCAGTGGTTTCACAAGCCGTAAACTTGGAAAGAATTCAGCAACAAATTGCAGAAATGCTTGGCGTGAAATTTGAGAGGGAGAGCGAAATGGGAAGAGCAGAACGGTTGAATGCAAGACTTAAGCAAAAGAAGAAGGTGTTGGTAATATTGGATGATATATGGGAACGGGTTAACTTGGAGGCTATAGGAATTCCATTTGGAGCGGAACACGAAGGTTGCAAAATCGTCTTAACATCTAGACAGGAAAATGTATGCAATCAGATGCGCACTCAAAGGAATATCCAGGTTGGAGTCTTACTGAAACAAGAAGCATGGGATCTGTTCCAGAAGATGGCAGGTGATTCTGTACATGCTCCTCAAATAAAACCTTTAGCATCTAAGATTGTCAAAAGATGTTCTGGTGTACCACTTGCTATTGTGACAATCGCAAGAGCACTAAATGATAAAAGCTTGTCATTTTGGAAGGATGCTGCCATTCGAAGACGCATAAGCAAGGAAGTTTTTTCAGCTCTCCAATTGAGTTATGATTATTTAGAAAGTGAGGAAGCCAAATTAATTTTCCTTCTTTGTTGTATATATGGTGAGGATCATAATATACCAATAGAAGATTTGTGGAAATATGGGTTCAGTTTGGGCTTGTTCCAAGAAGTTGATACAGAGGAAGCAGCAAGAGACAGAGCATTTGCACTGGCTGAGATCTTAAAATATTCTTGTTTATTACAAGAGGGTAGGGATGGAGATTGTGTCAAAATGCATGATGTTGTGCGAGACTTTGCCATTTCAGTTGCATCCAAAGATGAATATGGGTTTCTTGTAAGATCTGGTCCTGGATTTAACAACTTCCCAGAAAGCATTGAATTCCAACGGTACACTAGGATCTCAATAATATCTGATGCTATTCATATTTTTCCTGATAGATTGGAATGTCCGAAACTTCAATTTTTATATTTAGAATGCAGTCATCCTTCGTTGAAAGTCTCTGACGATTTCTTTTCAGGGATGAAATTACTCAAAGTTTTAGTCATGTCTGGTATGAATTTTTTGTCACTACCACCATCTCTTGTGGCTTTGGAAAACCTTCAGACATTGTGTTTAGACAATTGCAGTCTGAAAGATGTATCTATCATTGGGACACTAAAGGAACTAAAGTTTCTTAGCTTTATTGGTTCCAAATTGGAGGAGTTACCCAAAGAAATTGGAGAGCTGATTCATCTAAAGCTACTAGATTTGAGTCAGTGTGGAGAGCTCAAAATATTTCCCCATGATGTCATTCTGCGACTATCTCAGTTAGAAGGGTTGTATATGGGAGCTAGCTCTGATACATATGGCTTTAGAGCGTGGGAGGTAGAAAGACGAGACAAAGAAAGAAGAAACGTCAGCCTCAGGGATCTGAAACACATGTCTCGCCTAACCAGTATACACATTACTGTACCAGATGAAGACTGTTTGCCTGATGACGGGGATTCACTTTTTAATAACTTGTTGAGATTTCAGATATGTATAGGAACATCATTCGAAGCAAGGGGCCACTTTTTGTACTCAAGAGCATTAACACTTGAGTCCTTGAGGATTCCGTTAAAGTTGTTAAGCATTCCTCTCAAGACAGCTGAATATCTGCACTTGGACGAAATATTGGGGTTGGAAAACATACTTTATGACTTGGACACAGAGGGGGTTCTAAACTTGAAGTGTCTTACTGTGAAGAAAACTCGTAAGGTGGAATACATCTTTAATGCAAAGGCATCGGTTTCACCTGACACCTCTTTTAGCTTGGTGAAATTGAAGCTTTCTGATCTTCGAGACTTGAAAGCGATATACCACGGCCATCTTCCTAGTGAATTCTTCGTTAAGCTGCAAGAGTTAAAGCTAAGTGAACTACCTGCACTAATAAATATATGGAAGGAGCCTGCTACACATGCATGCCTCCGCAACCTACGAGTTTTGATAGTGGAAGAATGTAATGGACTGAAAAACCTCTTTCCACATTTCACAGCCGAAGATCTTTCAGAACTTGTGGAATTAAGGGTAGAATCTTGCGAGATAATGGAAGTAATATTTCAAAATGAAGTAGAAGAAGCAGCAGCAACGATGAATAACATAGAGTTCCCTCATTTGCAAATTTTGGAGCTCAAATCCTTGCCAAACTTTATAAATCTTTACTCTAAAAGGAAGTGTTCCAATACCGACACAAAAGAACCTCTCTTCTCCAAAAAGGTTGCATTCCCAACATTGGAGAAATTGATAATTTGGGAAGTTGGTGATGTGAAAGAGATTTGGCACCATCAAATTTCAGATAAGTCCTTTTTTCAACTGAAAACCCTTCAAGTAGATTGTTGTCACCAATTGCAAAATGTCGTTCCATTCCATATGCTAACAAGGTTACATAATTTAGAGCAACTTTCTATACAAAATTGTGATTCAGTGAAAGAAGTAGTTGAACTTGAAGGACTTGTTGTTGACGAAGGAGAAGTAAATGGAAGACTGTCTCAGTTAAGAGAATTATGGTTACGGAAGCTTCCTGAATTAAAGCACATCATGTGGTGGGACAAGTATCCACATGCAACGCTCAGCTTCAAAAATTTGGGTTCTATACACATTTTTGCATGTAACTGTCTGAAAAGCATATTCCCACCTTCTATTGCCAGCAGTCTTGTGCAACTCAGAAGGGTAGAAATAAATTCATGCGAGACAGTGAAAGCTATAATTTTAAACGAAGGAGGTGACAAAGAGAACTGCTTTGAGATCGTTGAGTTTCCACGGCTACACACTCTTACATTGGAAGCTCTGCCAAGTCTTGATTGTTTCTACCAAATCCCATGTCCTGCCAAAGAGGCCAACAGCCTTCATGATCTCAGACAACCTCTCCCCATGTTTGATGAAAAGGTTGCATTCCCTGTTCTGGAGAAATTGGTAATTAGAGAAGCCAACAATTTGAAAGAGATTTGGCATCCACAAGTTGCATTTAAATCTTTTTCTCAAGTGAAAGTCCTGGAATTGAAAAGCCGTCACAAGGTGTTAAACGTTATTCCCTTTTACATGCTTCAAAGGTTACAGAGTTTGGAGGAACTTTATGTGGAGGACTGTGATTCCGTGGAAAAGGTTGTTGAACATGAGGGGCTTGCTGTGGTGGAAAATGTAAATGGAACACTACCTCAATTACGAATTTTGAAGTTACGGCTACTACCCAAGTTAAAAAGTGTATTCCCACCATCTATAGCTTGTAGTCTTGTGCAACTCCAAGAAGTAGAAATATCATCATGCAAGATGATGGAAACAATTATCGTAAAGGAAAAAGAACAAAAAGGAAACTGCATAGGGACTATTGTGTTTCCGGAATTACACACTTTCAAACTTAAAGATCTTCCAAGCCTCGAGAGTTTTATGGGGTCTCCTAACAAAGAGGGCAACCTTGACCATGACCTCATACAACCTCTATTCGACAAAAAGGTTATATTTCCTGTTTTGAAGAAATTGGTTATTCATGGTGTCCATAATTTGAAAGAGATGTGGCACCCACAAATTCCGGCTAACTCCTTTTCTCAGCTGAAAGTCCTGGAAGTGCAAAGCTGTCATCAAATGTTAAATGTTGTTCCGTTTCATGTGCTAAGGATGGCACAGAGTTTGGAGGAAATTATAGTGGAAGACTGCGACTCAATGGAAGAGATAGTAGTTGAACCAGAAGGTCTTGTTGTCACGGAACGACATGTAAATGGAATTCTATTCACTCGGTTAAGAAGATTGAGGTTACGCAAACTACCCAAGTTAAAGCATATTATGTATTGGGACCAGTATCCACAGGGAACACTTGGCTTCCAGAGTGTGATTTCAATAGATATCTCTGGGTGCAACAGTCTGAAAAGTCTATTCCCACCTTCCATAGCCAACAGTCTTGTTCAACTCCAATATGTATATATTGATTCATGTGATCTGCTGGAGACAATAATTGAAAAGGAAAGAGACGGAGAAGAAAACTTGTTAGGGATCATTATGTTCCCACAGCTGCGCTCTCTCATCCTGAGTAAACTACCAAACATCATGAGTTTCCACACAGAGTTAAATTGGTCATCCCTAGAAAATTTGCAGATAGTGGGCTGTCCAGCCATGAAGACATTTATAACTAGCGTTCCATGCTCACAAAAGCTGAAGAAAGGAGTTGACGAATTAAAAGAGGCACCTAAGAAGGGCAACCTTTGCAGCTCCCAACGTCCTTTCTTTGACAACAAGGTCGCATTAACAAGTTTGACTAAATTGAGGATTGAGGACATGGACAACATGCAGCAGATATTTGATAACCAACATCCTGATGAATTCCTTTCTCAACTAGGAGGGCTGAGAGTGGTCAATTGTGACAAATTATTGAGGGTGGTCCCATCCTGCTTACTTCCAGGGTTAAGGAATTTATGTCAACTTGAAATAAGAAAATGCAAGTCAGTGGAAGAAATTTTGGAAGGAGTAGTTGGTGAAATACAGGCTACTGCTATATCGTTCACTAAATTAGAAGAATTGGAGTTAAATGATCTGCCTAACCTCAGGATTTTTTGCTCGGGGAATTATACTCTAAACTGCTCATTCTTGAATTCGGTGAAAGTGGAAAACTGTCCCAGAATGCAGACATTTTCTTCTGGATTAATCAGTGCACCAGAGCTAGAAAGAGTTGAACTAGAAGATGATAAATTTATATGGGAAGGTGACCTCAATACCACCATAAAACGCATTTTCCATGAAAAGAATATGTCGGGTATAGGAAGTGTCAGTGATGAGTCATCTCACAAGCTGGACTCTGAGCAGGGTAATGATGCCAATGGCGATGAGTTAGCCATCCAGGGGAGGATTTCTAGTGAGAAGAAAGATGCCGAGGAAAGTGAGACAGTAAGTCACTTGTACAGTCGTGAGAGTACATCAATCACCGTGTCAGCAACTAAAGAGAATATTGTCACAAACATTGATCAGGAGCACGATATGTTGGCTATAGGGGATTTTAGTGGCAGTGAGCCACCTCGCAAGCTGGCTTCCAAGGAGGAGGTCATCTCTAACAAGGGAAAGGGAAAGGCTGTGGCAGTTGTTGAAGGGATTGATGAGAGACTTCCTTCAAGTTCTAGGACACCGGTATCAAGTCACCAACCATGTTCGATTAGTGGTGTATCTATTGTGGAGGACACTCGGACGTTGTTAGATGCAGGTGTCTTCCCTGAGGACATTGCTAGTGGAGCTATTGATCGTCAGTACCTGCGTCAAAACATGGACAAGAATATGCCGAGTGTAGGGAGTGTCAGTGATGACTCATCTCACAAGCTCCATATTGATATGGGTCAGAATGTGCCAGGTGTAGGGGATGCCAATGGCGATGAGTTAGCCATCCAGGGGAGGATTTCCAATGAGAAGAAGAACGCTGAGGAAAGTGAGACAGTAAGTCACTTGTACAGTCGTGAGAGTACATCAATCACCGTGTCATCAACTAAAGAGAACATTGTCACAAACATTGATCAGGAACACGATATGTTGGCTATAGGGGATTTTAGTGGCAGTGAGCCACCTCGCAAGCTTGCTTCCAAAGAGGAGGTCATCTCTAATGAGGGGAAGGGAAAGGCTGTGGCAGTTGTTGAAGGGATTGATGAGAGACTTCCTTCTAGTTCTAGAACACCGGGGAAGGGAAAGGCTGTTTCAGTTGTGGAAGGGATTGATGAGAAACTTCCTTCTAGTTCTAGGACACCGGTATCAAGTCACCAACCATGTTCGATTAGTGGTGTATCTATGGAGGACACTCGGACGTTGATAGATGCAGGTGTCTTCCTTGAGGACATTGCTAGTGGAGCTCTTGATCGTCAGAACGTGCGTCGAAACATGGACAAGCTTCGTCCAGATCAGATGGTGGTCTCCCGTGATGAGTTTGAATTCCTCCAGAGGGATCGAGATTGGCTGAGACGGAGGGTGGAGGATTTGTTGGTTAGTGGGTATCGTGGTAGTAGAACTCCGGGTGTGAGTACGACTCTTGACGTTGGTTGGGCTGCTTGGAGTTTTTTGCTTATTTTTAGTCTTCTTTTTTTTCTTGCTTTTTGCATTTTGCTTTTTGGTGGTTTGTGTTATGCTTATATAAACAAGGTCTGATAAGAATGGAAAGATCTTTTTCCCTGCTTCATTTTAAAATAGGACCTTAGGTCTAGACCAATCTTTGGAATTTTGGGGCGGCCCTAACGGAATGCTCTAATATTTTCTTGTCATTTTTTGGTATGTTTTCAATTTGTTGTTCAAAAAAAAAAAAAAAAAAAAAAAAAAAAAAAGT

>transcript_30 full_length_coverage=2;length=6843

GGGGATCTGATTCGCAAAACGGAGAGGGGAATTTTTCTTTCATCAGAAGGCGAAGAACCACGACTGACTACAACTGGTTTTTGAATCTAATTTGAAATCTCGATATGGCCCATCTTGGCGGTGGTGCCGAGGCACACGCTCGATTCAAGCAGTACGAGTATCGAGCCAATTCGAGTCTTGTTCTGACCACCGATTCACGTCCGCGGGATACACATGAACCTACTGGCGAACCTGAGTCCCTATACGGAAAGATTGATCCTAAAACATTCGGTGACAGGGCTTATAGAGGGAGGCCTGTTGAGTTGGATGAAAAACTCAAGAAATCGAAGAAGAAAAAGGAACGTGAACCAATGGCTGAACCAGTGCCGAGTCGACAGAGCAAGAGACGACGTATTCAAGAGGAAAGTGTTCTGACTTCCACGGAGGAAGGCGTTTATCAGCCCAAGACTAAGGAAACTAGAGCTGCTTACGAGGCTATGCTTAGTGTGATTCAGACACAGTTGGGTGGGCAGCCGTTGAATATAGTAAGTGGTGCCGCTGATGAGATTCTGGCTGTCTTAAAGAATGATACGTTTAAGAATCCAGAAAAGAAAAAAGAGATTGAGAAACTGTTGAACCCTATTCCAAACAGTATATTTGATCAGTTGGTCTCCATTGGGAGGCTTATTACTGATTTCCAAGATGGGAGTGATGTAGCAGGGGTTACAGTGACAAATACTGATGATTCTCTTGATAATGATATGGGTGTTGCAGTTGAATTTGAAGAGAACGAGGACGAGGAGGATGAAAGCGATCTTGATATTGTACAAGATGATGAAGATGAGGATGAAGACTTAACTGAACCCCATGGTTCAGGTGCTATGCAAATGGGCCGCGGGATTGATGATGATGATATGCAGGAAGCAAATGAGGGTATGACTCTAAATGTGCAAGATATTGATGCTTATTGGCTTCAGAGGAAGATCTCACAGGCATTTGACCAACAGATTGATCCACAACAGTGCCAAAAGCTTGCAGAAGAGGTACTCAAAATACTTGCTGAAGGTAATGAAGGAGAAATTGAATCTAAGCTGTTGGCCCATTTACAGTTTGAGAAGTTCAGCCTGGTTAAGTTTTTGATGCGCAATCGCCTGAAGATTGTCTGGTGTACTCGTTTGACAAGGGCTGAAGACCAAGAAGAGAGGAAAAAAATTGAAGAAGAAATGGTAGGTCTGGGTCCAGATTTAACCGCAATAGTGGATCAGTTGCATGCCACAAGGGCAACTGCCAAAGAGAGACAAAAGAACTTGGAGAAGAGTATTAGAGAGGAGGCTCGCCGGCTGAAGGATGAGAGTGGTGGGGATGGGGACAGAGCTGGCAGGTTCGTTGACAGAGAGTCAGATGGTGGTTGGTTGAAGGGTCAGCGCCAGTTGCTTGATCTTGACAGCATTGCATTTCAGCAAGGGGGTCTTTTAATGGCGAATAAGAAGTGTGAGCTTCCACTGGGGTCTTACAGAAATCAAGGCAAGGGTTATGAAGAAGTTCACGTGCCCTATTTGAAGCCTAAACCAATGGCTGCTGATGAACATCTTATAAAGATAACAAGCATGCCAGAATGGGCACAACCCGCGTTCAAAGGAATGACCCACTTAAACAGGGTTCAGAGTAGGGTTTATGAGCGTGCCCTTTTTAGGGCAGATAACCTTCTTTTATGTGCTCCTACAGGGGCAGGGAAAACTAATGTTGCAATGCTCACCATACTACAACAGATGGCTTTGAATAGGAATGATGACGGGTCTTTCAACCACAGTGATTATAAGATTGTCTATGTGGCGCCAATGAAAGCTCTTGTAGCTGAAGTGGTTGGTAATTTATCTAATCGTCTGCAGGACTACGGTGTTACAGTGAAGGAACTTAGTGGTGACCAGTCGTTGACTCGCCAACAGATAGACGAAACTCAGATAATCGTCACTACTCCTGAGAAGTGGGATATTATTACCAGAAAGTCAGGAGACCGTACCTATACACAGCTTGTCAAACTTCTCATCATTGATGAGATTCATCTTCTTCATGATAATAGAGGACCTGTGCTGGAAAGTATTGTCGCGAGAACTGTTAGGCAAATTGAAACCACCAAAGAGCATATTCGTTTAGTTGGGTTATCGGCTACCCTTCCTAATTATGAAGATGTTGCATTGTTTTTAAGGGTTGATGTGAATAAGGGACTCTATCATTTTGATAATAGTTACAGGCCTTGTCCTCTTGCTCAACGATTTATTGGAATCACAGTGAAGAAGCCACTACAGAGGTTCCAGTTGATGAATGATCTCTGTTATGAGAAGGTAATGGATGGAGCAGGAAAGCACCAGGTTCTTATTTTTGTCCACTCGAGGAAAGAAACAACCAAAACGGCCCGTGCAATCCGAGATACTGCACTTGCTAATGATACTCTTGGTAGGTTCTTGAAAGAAGAGGGTGCGAGCCGTGAGATCCTCAATAGTCACACAGAGCTGGTCAAGAGCAGTGATCTAAAGGACTTGTTGCCATATGGGTTTGCTATTCATCATGCTGGGATGGCAAGGACTGATCGGCAACTTGTTGAAGACCTCTTTGCTGACGGGCATGTACAAGTACTGGTTTCCACAGCAACCCTTGCTTGGGGTGTGAATTTGCCTGCTCATACTGTGATAATTAAAGGGACCCAGATTTACAGTCCTGAAAAGGGAGCATGGACTGAACTTAGTTCTTTAGATGTTATGCAGATGCTGGGTCGTGCAGGAAGGCCTCAATTTGATTCTTACGGAGAAGGCATAATCATTACTGGCCATAGTGAACTACAATACTATCTTTCATTGATGAACCAACAGCTTCCTATTGAAAGTCAGTTCATCTCCAAACTAGCTGACCAGCTGAATGCTGAAATTGTTCTTGGAACTGTTCAAAATGCTAGAGAAGCCTGCAATTGGATTGGGTATACATACCTATATGTTCGCATGTTACGAAATCCTACGCTCTATGGTTTAGCACCTGATGTGCTGACAAGAGATATAACACTGGAGGAGAGGAGGGCTGATTTGGTTCATTCTGCTGCAACTGTTTTGGACAAGAGTAACCTGGTGAAGTATGATAGGAAAAGTGGATATTTCCAGGTCACGGACTTGGGTCGTATTGCTAGCTATTACTATATAACTCACGGGACAATTTCGACATATAATGAGCATTTGAAGCCAACTATGGGAGATATTGAGCTTTATCGGCTGTTCTCTCTCAGTGAAGAATTCAAATATGTTACAGTGAGACAAGATGAGAAGATGGAACTAGCAAAGCTTTTAGATCGTGTTCCTATACCCATCAAAGAAAGCCTTGAAGAACCTAGTGCCAAGATCAATGTTTTGCTTCAAGCATATATTTCACAGCTGAAGCTTGATGGGCTTTCAGTGACGTCTGATATGGTGTACATAACTCAGAGTGCTGGCCGTCTTCTGCGAGCTCTTTTCGAGATTGTTCTAAAAAGAGGATGGGCGCAATTAGCTGAGAAGGCTTTAAACCTCTGTAAAGAGGTTACCAAGAGGATGTGGAGTGTCCAAACGCCCCTTCGCCAATTCACTGGAATTCCAAGTGAAATTTTGACAAAGATGGAGAGGAAAGATATGGCCTGGGAAAGATATTACGACCTCAGCCCACAGGAGATAGGAGAGCTTGTCCATTACCAAAAGATGGGTAAAACACTTCACAGGTTGATTCACCAGTTCCCAAAGTTAATCCTTGCAGCTCAGGTTCAGCCAATTACTCGCACTATTTTGAAGGTTGAACTGACAATAACACCAGACTTCCAATGGGAGGACAAGTATCATGGATATGTAGAGCCATTTTGGGTAATTGTGGAGGATAATGACGGGGAACAAATTCTTCACCATGAATACTTTATGTTGAAGAAACAGTATATCAATGAGGACCACACGTTGAGTTTCACGGTGCCAATCTATGAACCATTGCCACCCCAGTACTTCATTCGTGTTGTTGCAGATAAATGGCTTGGATCACAGTCTGTTTTGCCCGTTTCCTTCAGGCATCTTATTTTACCAGAAAAGTACCCTCCACCCACTGAGTTACTGGATTTGCAAGCTCTGCCCGTGACAGCATTGAGGAACCCATCATACGAAGCACTTTACCAAGAATTCAAGCATTTCAATCCTGTCCAAACTCAGGTTTTTACTGTTCTATACAACACCGATGACAATGTTTTAGTTGCAGCACCGACGGGGAGCGGGAAGACAATATGTGCAGAGTTTGCTTTATTGAGGAATCATCAAAAAGGACCTGGAAGTGTCATGCGTGCTGTGTACATCGCACCCATCGAAGCTCTTGCTAAGGAACGTTATCGTGACTGGGAAAAGAAATTCGGAAGGGGTCTTGGAATGCGTGTTGTTGAATTAACCGGAGAAACCACGACCGATTTGAAACTTCTGGAGAAAGGCCAAGTAATTATCAGCACCCCAGAGAAATGGGACGCTTTGTCCCGCCGCTGGAGACAGAGGAAACATGTTCAACAGGTTAGCCTCTTTATCATCGACGAGCTCCATTTAATTGGGGGGGATCATGGCGGGCCTGTATTGGAAGTCATTGTTTCCAGAATGAGATACATTGCAAGTCAGGTTGAGAACAAGATACGCATTGTAGCCCTTTCTACTTCTCTTGCTAATGCCAAGGATATTGGAGAATGGATAGGGGCAACTTCTCATGGCCTTTTCAATTTCCCTCCCGGTGTCCGTCCTCTGCCACTGGAGATACACATTCAAGGAGTGGATATAGCAAATTTTGAAGCAAGGATGCAGGCAATGACTAAGCCAACATACACTGCTATTATGCAACACGCTAAGAATGAAAAGCCTGCTATTGTCTTTGTTTCTACAAGGAAGCACGTCCGACAAGCTGCTATCGATCTGATGACCTACTCAATTGCAGACACGGGGGAAAAATCAACATTCCTGTTACGTTCCCTTGAAGAACTAGAGCCGTTTGTTGAGAAAATTGAGGAAGAAACTTTAAAAGCCACCCTACGTCACGGTGTGGGTTACTTGCATGAGGGTTTAACCAGTATGGATCAAGAAGTTGTGTCACAGCTATTTGAAGCGGGGTGGATTAGGGTTTGTGTGATGATTAATTCAATGTGTTGGGGGCTGCCTTTGTCAGCGCATTTAGTTGTTGTGATGGGAACACAGTACTACGATGGGCGGGAAAATGCTCACACTGATTACCCAGTTACCGATCTGTTACAGATGATGGGTCACGCGAGTCGACCGCTTCTCGACAATTCCGGAAAGTGTGTCATTCTTTGCCACGCGCCAAGAAAAGAGTACTACAAGAAGTTTTTGTACGAAGCGTTTCCAGTCGAGAGCCATTTGCAACACTTCTTGCACGATAACTTCAACGCAGAAGTCGTTGTTGGAGTGATTGAAAATAAGCAGGCTGCGGTTGATTACTTGACGTGGACGTTCATGTACAGAAGATTAACACAGAATCCAAATTACTACAATCTTCAAGGAGTGAGCCACAGACATCTTTCAGATCACCTATCCGAGCTGGTAGAAAACACATTAAGTGAATTGGAAACGAGCAAGTGTTTGTCGATCGAGGATGATATGGACCTTTCTCCATTAAATTTGGGGATGATTGCTTCATACTATTACATCAGTTATAGAACGATTGAGCGGTTTAGTTCTTTATTAACTATGAAAACAAAGATGAAGGGTCTTTTGGAGATTCTGGCTTCGGCTTCTGAGTATGCCCAGCTTCCTATGCGTCCCGGGGAGGAAGATTTGATTCGAAGGTTAAATAATCATCAGAGATTCTCAGTGGAAAGTGCAAAGTGGAGTGATCCACATGGCAAGGCAAATGTTCTTCTACAAGCGCACTTTTCAAGACAAACAGTGAGTGGGAATTTGAAGTTAGACCAGCGAGAGGTGCTGCTTTCTGCTAGCAGATTGCTTCAGGCGATGGTTGATGTTATATCGAGCAATGGTTGGTTGAGTCTGGCCATTGTTGCAATGGAAGTGAGTCAAATGGTGACACAAGGGATGTGGGAGCGGGACTCGATGCTGTTACAGCTTCCTCATTTTACAAAGGAGTTGGCGAAGAAGTGTTTGGAGAAGGGTGTTGAGACGGTGTTTGACTTGGTTGACATGGCAGATGATGAGCGGCGCGAGCTTTTACAGATGTCAGACGCGCAGTTGTTGGACATTGTTAAATTCTGCAACCGATTCCCCAACATTGACTTGACGTACGACGTTCTGGAGAGTGATAACGTGAGAGCTGAAGAAAATGTCACGTTACAAGTCAACCTGGAGCGCGATATGGAAGGGCGGGCAGAAGTGGGTCCAGTTGATGCTGCAAGGTATCCGAAAGCTAAAGAGGAAGGGTGGTGGCTTGTGGTTGGGGATAGCAAGACAAACCAGTTGCTTGCAATCAAGAGGGTGTCTCTGCAGAGGAAGGCTAAGGTGAAGCTTGAGTTTTCTGCTCCGGCGGAAGTGGGGAAGAAGGCGTATACATTGTATTTCATGTGTGATTCGTATTTAGGTTGTGATCAGGAGTATAACTTCAGTCTTGACGTTAAAGAATCAGGTGCCATGGAAGAAGGTAATGAATGATATAAATTAATTCTATTTGGTTAGCTTTTGTTAAGTTTGAACGGGTTGTATACCAATCTGCTTATCGAGTAGAATTTTTGTATTTTTTTTTTCCTCTTCTGTTCTCTCTGTAGCAATTCAAGTTTATGAACTGAACAATAGATTGGGCCTTTCTGGTCTGGGGTTAATTTATCATCATCTTTTTCAAAAAAAAAAAAAAAAAAAAAAAAAAAAAAGT

>transcript_31 full_length_coverage=2;length=6819

GGGGTGAGGGCTTTTTCTCTTCCCCCCCTCTTCGAAGCCTTCCTGAGTTCGTACTTTATACTTCTTAAACCCTGAACCCAGAAGCAGGGAGAGCGGCAAAGATAGTGAACACTGCGTATATACGAAGAGAGATGGAGGTTGAGTTGGAGCCGAGAGTGAAACCCCTGCCCTACAAAATAAAAAGCATGTCTCGTGAATCCCCCTCTCAAAAGGCTACTCATGTCCTTGAAACCGACCTTCGCGCTCACTGGTCTACTGGTACCAACACCAAGGAATGGATCTTGCTCGAACTTGACGAACCTTGCTTACTATCACATATACGGATTTATAATAAGTCTGTTCTTGAGTGGGAAATTGCAGTTGGTTTGCGTTACAAGCCAGAGACATTTGTAAAAGTTCGCCCACGCTGTGAAGCGCCTAGACGTGAGATGATGTACTCTATGAACTACACTCCGTGCCGCTTTGTGCGAATATCATGTTTGCGGGGTAACCCTATAGCTATTTTCTTCATTCAGCTGATTGGGATTTCTGTGATTGGTCTTGAACCGGAGTTTCATCCAGTTGTTAATTACTTGTTGCCACACATTATATCACACAAACTAGATGCTCATGATATGCATCTCCAGTTGCTTCAAGCCATGGCAAATAGGTTGCTGGTATTCCTTCCCCAACTTGAGGCAGATCTCACCACCTTTTCAGATGCTGCTGAGACTAACATTCGTTTTCTTGCTATGCTTGCTGGTCCATTCTATCCAATTCTTCATGTGGTAATTGAAAGAGAAGCTTTGAGGTCTTCGGGCAATATTGCAGACTCTGAAGCTTCTAGGAATAGTCTGCTATCATCAGCGCCGACTGTTTCTTCCAATTTTGAGCCAAGGAGATCGCGGTGCACTTCACCTTTTCTCTTACATACATCCAGTGCCCCAGTGTTCCGTCCAGATGCAATTATTGTGCTACTGAGAAAGGCATGTAAAGATTCCAATCTGGGAACTGTTTGCAGGCTGGCTTCTAGAATTCTGCAGAAGTTCATAGAGCCAGCTACAACGCATGAAGAATCAGTGCTTTCTGGTGCAGTTGCAGCTTCTGTTACGGATGACGTGTCAAAATCTGAAGTACCTACTCCCGTTTGTTTCGTTGATTATTCAAACTTGTTTGGAGAAGAATTCCAGATACCTTATGATAAGTGGGACTCCAGCTATCTTAATGTCTTGGATGTAGGGGCAGTGGAAGAAGGGATTTTACATGTTCTTTATGCTTGTGCATCAGAGCCTTTAATGTGCAGCAAGTTGGCAGATACCACCTCTGACTTCTGGTCTGCGTTACCACTTGTACAAGCGTTGCTCCCAGCGCTCCGCCCTTCTGTGAGCAGTTCTCTCGAGCATGTTGATGATAATTTTTTTCAATGGAAATTACCTTTTGTGCAACGAGCCCTATCCCAGATTGTAGCGACATCGTCTTCATCATTGTATCGTCCACTTCTTCATGCCTGTGCTGGCTATTTATCGTCATTCTCACCATCACATGCTAAGGCTGCATGTGTTCTGATTGATTTGTGTTCTTCTGTGTTGGCACCATGGATGTCTCAGGTGATTGCAAAGGTTGATCTAACCGTGGAGCTTTTGGAGGACCTTCTCGGTGTAATTCAGGGTGCTCACTATTTCTTGCCTCGTGCTCGTGCTGCCTTGAAATATATCATGCTGGCCCTATCTGGGCATATGGATGATATACTATCAAATTATAAGGAAGTTAAGCACAGAATTCTCTTCCTTGTCGAGATGCTGGAGCCTTTTCTGGATCCTGCCATATTCTCAATGAAGAGTACAATTGCATTTGGGGACGTATCCTCCATTTGTCTGGAAAAGCAGGAAAGTACTTGTATTATTGCCCTCAGTGTCATCCGCATGGCAGTACGAAAGCCTGCCGTTCTTCCTTCACTGGAATCTGAATGGAGGCGTGGGTCAGTTGCACCTAGTGTGCTCCTCTCAGTATTAGAACCTCATATGCAGTTGCCACCTGAGATTGATCTTTGCAAGTCTCCTGTTTTTAAAACATCCGAACAAGAACCTTCAAATGTTTCATCATCTTCTTCTGTTCTTCACCATGGAGCAGTCTCCTTGAAATCCAGTAGTGCAGATGACACTAATGGGAAGGCAGATGGTTCTGACACAACATTACAGATGGACATTTTAGAAGATTTAAGTTTTTTTTTTTGCCCCGCCAGAACTGAGGAGCATAGCACTGGCAAATACTTTAAGCGGTGGGTTAGAGTCTGCCTATGGCAATGTCAATACAAAAGAGAAACTTGTAGTGGATAAAATTTTGGCTAACCAGTCTCCAAATGCTGTAGTGTTGGATGCTGGTTTTGCTGTTGAATACTTTAACTTGCACACAGACTATTTGCAGCTTACAAATTTTCGAGATTGTGAGTTGAGAGCTTCCGAGTTTCAACGTCTGGCTTTAGACTTGCACTCCCAAACTGGTATTACCCCCGAGGGTCATGATGCCGCTATAGATGCTTTGCTGTTGGCGGCAGAGTGCTATGTCAATCCCTTTTTTATGTCATTCAGAGCCAGTCAAAATGTTGTCAACCAGATGAAGAATGACGGGACCAAAATTCCACGAAACTTCGACATTTTAGAGCTCAGAAACACTAGGGAAAAGAACAATAGTGATTTGAAAAAAGTAGCTCATCTTGAAAGGAAGAGAGACAGAATTGTTCTTCAGTTACTGCTCGAAGCTGCCAATTTGGATAGAGAGTACCGGGAAAAGGTGTCTGAGGGAGAACACTGTTCATACCATATTGAAGCTTATCATGAACAAGTCGTACATATGTCTCCACTTGATATAGAATCTGCAGATGCAATCACCTTGGTTCGGCAGAATCAAGATTTATTGTGCAACTTTCTCATTCAGCGGTTGCAAAGAGAGCAGCATTCAGTGCATGAAATTCTTATGCAAAGTCTCCTGTTTTTGTTGCATTCAGCTACTAAGCTATCCTGTGCTCCTGAACACGTGATTGATATCATTTTGGGTTCTGCTGAATATTTTAATGGGATGCTAACCTCTTTATATTATCAGTTCAAAGAAGACAACTTGCAATTGAGTCCAGAAAAGGTTCATGAGGTACAACGTCGTTGGATGCTTCTTCAAAAATTGTTGATAGCTTCAACCGGTTTTGATGAAGGGCCAGATGTAGTCATTAATATCAACAAGGGTTTTTGGTATGGGAATTTGATTCCACCTTCGGCATGGATAAAGAGAATACCTACATTCTCTAACTCTGCTTCCCCCTTGGTTCGCTTTCTTGGTTGGATGGCAGTATCTCGTAATGCCAAACAGTATTTAAAAGAGCGTTTTTTCCTTGCTTCAGATCTATCACAGTTAACGTATTTACTATCCATTTTTGCAGATGAGCTTGCACAAGTAGATCATATTCTCAATAAAAAGGATCAAGCTTTAAATGTTGAAGAATCAGGAGATAAACAAGATCCTTGTGTATATGACAACCAATCCTTTGGTGTCGTTTACCCTGATCTCAGCAAGTTCTTTCCAAATATAAGAAAACAATTTGTAGGTTTTGGAGAAACTGTTTTGGAGGCTGTTGGAATGCAGTTGAGATCTCTTTCTTCTAGAGTTGTGCCTGATGTACTGTGTTGGTTTTCTGATCTATGTTCATGGCCGTTTCTCCAAATGGGCCATCCTTCTAGTTATTTGAAAGGTTACGGTGCTAAAAATGCCAGAGCAATCATTCTGTACATTCTTGAAGCAATTGTAGTTGAGCACATGGAAGCGATGGTGCCTGAAATACCTAGAGTCGTGCAAGTGTTGGTATCCCTATGTAGAAACTCATATTGTAATGTGTCATTTCTCGACTCTATTTTGCGTCTGTTGAAGCCAATTATCTCTTATTCTTTACGTAGGGTGTCTGATGAGGAAAAAATGTTGATTGATGATTCATGCCTTAACTTTGAGTCTTTATGCTTTGATGAACTTTTCAGTAATGTCAGACACAAAAATGAGAATCAAGATGGTTCTGAAGAAAAAGTATACAGCAGAGCGCTAACAATTTTTATTTTGGCTTCCGTATTTCTTGATTTATCTTTCCAACGTCGTAGGGAATTTTTACAGTCATTGATCTCGTGGGCTGAATTTGCTACTTTTGAGCCAACTTCTTCTTTCCATGATTACCTTTGCGCATTTCAATGCGTCTTGGAAAGTTGCAAACTTTTGTTCATTCAGACGTTAAGAGTATTTGGTATCATCCCATTTCAAATGCCCCACTTTTCTGACGTCAACTCTGGTGCACTCCGTGAGGATAGCTTAGAGTCATATTCATGGTTTCTGATTGATGTGTGCCCCAACTCTTCCCTAGCAAAAGTTTCCGAGAAGGTTGGAACTAACAATTCTGATGCTGAAAAATGTGAAAGGGTTCATCATTTATCTACTGAAGAAATAGAATTTTTTTGTAGAGACTTAGAGTCTCTCATTGACAAACTTATTCCAACTATTGAGCTTTGTTGGAAGCTCCATCATCTACTAGCAAAGAATTTGACTATCACATCAGCAGAGTGTTTCTTTTACTCGAGATGCTTATCTTCGATTGCAAAGAAAGTTGTTAATGCTGTAGAGGATAACAGCAGAAATCTTTCTCCATCTAAGTCAGTTGACCAGTTTTCATTTGATTGGAGGATTGGTCTTGAAGGACTTTCTGAAATCATTATGATGCTCCAAGAAAGCCATTGCTGGGAAGTTGCATCTATGCTGCTTGATTGTCTACTAGGAGTGCCCCAGTGTTTTCGTCTGGATAACGTGCTTGGTTGTATTTGTTCTGCAATGGATAATTTTTCTTCTAAAGCACCAAAAATTGCTTGGCGTTTACAGATTGATAAATGGTTGTCAATTTTACTTGCAAGAAGTGTCCATAGCTTTAATGAAGGTCAGGCTCATCTTGTTGGTCTGTTCTGTAAGATGCTAGGTCACCCCGAACCTGAGCAACGGTTTATTGTACTCCAGCACTTAGGAAAACTCGTTGGCCAAGATGTGAACGGTGGAACAGCAATACTTCACAACATGCCTGTTTCACCTAATCTAGCTATTCCGGTTTCTGAGTCAATTTTATCTTTGCTTGTATCAAATACATGGGATCAAGTGGTTGTGCTGGCATCATCTGATACTTCCTTACTTTTGAGGACGCGTGCAACGACACTTCTTGCAAGTTATATCCCATTTGCGAATCGGCACAAGTTACAATCTTTTCTTGCTGCAGCTGATAGTGTTCTTCCTTGTTTGGGAGAGCTTGCACATCCAACATGCGAGGGCCCATTACTACAACTTTCATTAGAACTCATTGCCAGTGCATGCTTACACTCTCCAAGTGAAGATATTTTTTTGATCCCAGAAAATGTTTTGAGGAACATTGAGACTTTAGGAATGTCAAAAAATGGCAGGCTTGGGGATCTCGAGAAACAGGCTTGCCAAGCTTTGTGTAGGCTGAGAAAGGAGGGGGGTGAGGTTAAAGAGGTCCTGAAAGAAGTGCCATCTTCACGCTCTTTGAAACAAGTTGACCCCGATTTTCTAAGCACTCGAGAATCAATTCTTCAGGTCTTAGCCAATCTAACTTCTGTTCAGTCGTACATTGATATCTTCTCAAACAAAATTGAGGAAGATGTTATGGAACTAGAGGAAGCTGAAATAGAGTTGGACATTCTTGAGAAAGATGAGACATTACGAGAGTCATCTATAGATATTAAAGAAGGGCAAAATCTTTCTTGTATAGCCACTTACGTGAAGGATGATAGCCGTCTTCAGCAAATTAAGGATTGCATTCATTCCATCGAGAAATCCAAAATCAGAGAAGACATAAAATCCCGCAGGCAAAATAAGCTTTTTGCAAGACGTGCACGTCATAAATATTTAGAAGAGGCAGCTTTACGAGAAGCAGAACTTGTTCAAGAACTTGATAGGGAGAAGGCGAGTGAAGCGGAGAGGGAAATTGAGAGACAGCATTTGTTGGAACTTGAGCGCGCAAAAACGAGAGAGCTGCGTCACAATCTTGATATGGAGAAGGAAAGGCAAGCACAGAGAGACTTCAGCGTGAACTAGAGCAAGCCGAATCTGGTGTGCGGCCATCCCGACGTGAATTTCCTTCTTCCAATCATAACAGTCGGCCTCGGGACAGGTATAGAGAAAGGGAAAATGGGAGACCGGGCAATGAAGGAAACTATAGAACAAGTGGCGGCGGTGAAACTTTGATGACCAGCACCAGCCCGTCTGTTGTTATGTCAGGGGGGTCTCGACCATATCCAACAATTTTACAATCGCGGGATCGTCTGGATGAATGTGGGAGTAGTTATGAAGAGAATTTTGATGGAAGTAAGGATTCGGGTGATACAGGTAGTGTTGGAGATCTGGATTTGGTGTCGTCATTTGATGGGCAGAGTGGTGGATATGGGTCGGGTCAAAGGCATGGAGCCAGAGGGAGCAGCAAGTCGTCGAGGCAGATGATGGAGCGTCGGGAGCGAGGAGATGGAAGACGGGAAGGCAAGTGGGAGAGAAAACATTAAGGATGAAAAAATGCGACGGACTTGGGACATCATGTCTTGTACTCTTGTAACAATACAATGTATGTATAATATTAGACAAAGCTTATTTTGTTTTATTTTTTTTATCTTCGAGGAGATATGTAGAAGCATTTGATCAGGTGACACGTGGAATCATGGCAAAGACAGCGGCTGATGAAACCCAAAGTTGAATATGTAACGAGTGATTATATTGGTTGCTTGCCCTAAAAAAAAAAAAAAAAAAAAAAAAAAAAAAAGT

>transcript_32 full_length_coverage=2;length=6821

GGGGGAGTTACAGAGCCTTCTTAACTTATACTTTTCTCCACAATTGCCATCTTTTCCCCTCTCTCGCACCGTCTTGCTCTGTAGCTCGCACCCTCCACAACTGCCGTCTTTTCCCCTCTCTCAATCCGTCTCCCCCTTTTCTGCCACAGCATGGCTACAACAATAGGGTCACAGTTACAAGCAGTTAAGTCCATCATACAAGCCAACCCGGAACCTATTAAACGACCCTTCACTCGGCCATCCGTCATTTTTGATCCCAAAGAAGCTGCAGACACTGACATCGACACCATAAGAGAGATTGCACTCCCAGGTTTAGAAGTTCTTATCCTTATAGATGAACGTTTTAGAACCTATAAAAAGAACTTATTTAGTCACCAGAGTAGAGAATTAGACCGAGAGTTGATGGGCATAGAAGAGAATAACAATATCGATGCATCCATTAATTCTTACCTGCAGTTACTCTCGGGATATCTTGAACTACCTTCTGCGCTTAAGACACTGGAGTACTTGATACGTAGATACAAGATACATGTCTACAACATGGAGGAATTGCTTCTATGTGCATTGCCTTACCATGACACCCATGCTTTTGTTCGAATTGTGCAGTTACTTAAATTGGGAAATGGTAAATGGAAATTTCTCGAAGCTGTTCAAGTTTCAGGGGCACCACCACCTAGAAAGGTCATTGTGCAGCAGTGCATCCGTGATATGGGGGTTTTGGAGGCTTTATCCAACTATGCATCTCCTACAAAGAAGTTCCAACATTTGAGGCCTGTAATCAGCTTTTGCACAGCAGTTATTGTCGAGGCTTTGGGTTCTGTAATGACCGTTGACAGTGACATCGTGAAGAGAATTCTTCCATTTGTAGTTTCTGGACTTCAACTTGGTGCAAAAGGAGGTCAAGATCACAAGGCTGGTGCTTTGATGATTGTTGGTTTGCTAGCAAGTAAAGTGAAATTGTCTCCTAAACTTGTCAAAAGTTTGATCCGGTCAATTGCCAACATAGCTCAAGAGGATGCAAAAGAGTCAACTGACCTGCAGTGGTTTCGTTTGTCACTAATGTCATTAATTAACGTTGTTCAGTTGCAATCCGTCGATATAATCCCGAAGAAAGCTTTGGATATTCTGAAGGAAATCAGGGATCTTGCTGGGGTTCTTTTAGAACTGTCCAAGGAGTTCAATATTGATAAATTTCTTGCCGTGCTTTTGGATTCTCTGATTGCATACAGTTCAGATGATTCTTGCCATCTTGCTTTGATATCAATAATCAAAACAATTCCTGTGAAGGATTTTGTTGACCAGCTAGTTTCCAAGGTTCTATTTTCCTGCATAAGACTATCAGAGAAACTGTGTGATTCAACATCATCCGAATCAGGAAACCGGGCTAAGCAAATTTTGCTTCTTGTTAACAAGTCCTATCCCTTGGAACTACGTGGAGCAGTTCGCAAGTTCTTGGAGGATACCAAAGTACAACCCAAAGGAGAAGGTTCGGTATTTGAGATCTTGTCTAGAATGTTAGATGGGAATATAGATTTACCACTGGCAGTCCCAGATTTAAAAATTTGGTTTACTTTGGATCATCCTAAGGCTGAGGTCCGACGTGCGGCACTTTTTGGTTTGGACACATCTGCCATTCTGAAATCCAAAGCTACTGATTCACAGAGGTTTTTAACTATTCAAGATGCAATATTGAGCCAACTTCGGGATGATGACCTAACTGTTATTCAGGCAGTTTTATCTCTTGATAAACTGTCTGAAATAGTAAATTCTCCTGGATTTCTCAAAGCATTGCATGGTGTGCTCCAGAGATGTATTAGCATATTAATGTTAAGTACATCCAATAGTACTGCTTTAGCTTGTGATGTTGCTCTTTCATGCCTGAAGCATGCTGTTTCAAACTTCCGTAACCAGATTGACTACTCAAAAAATCTTGCTTCTATGATGCTTCCTTTAGTTCTAATTTTACCCGAGACACAGAAGTTGAACCTGAAAGCTTTGGAATTAGCCAAGGAAGTAAATTGGTCGTTGTATCAGAATCTTGGTGGTACCTTCAGCACAGAAGCAAAATTTGAACCTGAAAGCTTATCTACAATTAATGTGGATACTATTAGCTGTTTGGCAAAAACGTTTTCAGTGCATCCGGAAGAGTACATCCCTTGGTTTGTTGAATGTTTCAATGATTTTGAGCTGTCAAAGACGACAATCTTCTTGGTTATGCTGCAGTCACTGATGATGCAAAAGCATAAAGTTTGTCAATTTTCGGCATGGTTTGAAGCTTGCTTTCCTGTTCTGAAGAATGAGTGGAATATTCTTCTAACTGCAGGACACCTTTTTTCAATGGAAGAGTTCAACTCAGAAATGCTGAATTGGGATTGCGTACGATTAGCAGATCAGCTCTTTGACTCCAATCTTAATCTTAGAGATTTAAATGCAAATATTCTAATTTGCATATTTTGGAGACTAGTAGATGTCTTTATCTCAACAGTTCCTGCAGATGTCTTATTGGATAATAATGGACTATGGACATGCAGACTTCGGGATATTTTTGTCTTTTTTGCGTCCTCTCGGTTAAAGCACGTATATAGGAAACATCTTCACGAACTTATTACGAAATGCAACATTTCTCCCGTTCCTTTCTTATCCAAGTTTTTTACAGAAGAAGGTGTTCCAATTGCAGTTCAAGTAGAGAGTCTTAGTTGTTTTTCATTTCTTTGTTCTCGTTCAGATGATGAATTATCTTCTCAACTCTTGATCGAATTTCCATCACTCCTTGTTCCCTTGTCCGTTGAAAACCTGGATATAAATATGGCTGCCGTGGAATGTGTTGACGAATTATACTCATTATGGTCTTCCAATTTTTCTAGCAAGAAAAATGGGAACAATGCGATTTGGAGTCATTTTCTTGGGGATCTTTTGAGGTTACTGGTTCAGCAAAAGAGACTTATTGTATCGGATAGACACTTCCTCCCATCCCTTTTGACAGCTTTGCTCAGCTCTTCCAGTCATAGTCTACTGGTGCCACACGATATTGAACAAAGGTTTGACCAATCTATGAAAAAAGACATTCTGGCCTTCATTTTGGGTTCTGCTCTAAAATTTTCGGCGTATGGGAAGCTGAGGATTCTGACTTTACTCAAAGGAATTGGCAGTGCAATTGTGCTTTCTAAAGATGTTGAGGAATTGCTATCTGAGCTTTTAAGAAGACGCAGCCAATACTATTTGAAGCTGGATAAATCATGCCAGAATTTGTCTGCTAATGAAGTTGAGATCCTGTGTGTTTTACTTGAGAGCTGTGCCGTCCCTACTCCACTTTGTGGACATGTTTATGAAGATCATTTGTTGAAGGCATTGCAATTGAAAGGTATGCCTACGGACGACCCTGTTGCTGTTCAACCTTGTATCACAGTTCTGCTAAAGCTCAGTGCTTCCCTGTATAGTAGTTTGAAGACTGAGACACAGGATCGCTTGTTCCATGACATCGTCTTTCTCTTTCGGAATGCTAATGGTGCTATACAAAATGCAACTAGAGAGGCCTTGCTGCGCTTAAATATTACTTGTTTCACCATAGGCCAGACGCTTGATCGCATTAATAAACAAGAAAGGTGCATGGTTGCTTTATCATCTGGGAAGAAAAAAAAGAAACCAATGGAGCAACATAACCTTGATCTTTATCGTGAAGGCGAAAATACAATTTTTTTTCTTGGCTCCCTCCTTGACATTTTGCTATTAAAGAAGGACATAGAGAACAGGAAATCTCTTTTAGGACCCTTATTTAAGCTTCTTGAAAAGATACTTTCAGATGAGTGGGTTTCTGGGGCTGTTGATCATGATGATGAGTGGGTCAAGGCTTCATCTGGCATTTCTCAGACAATTTCCAGCACGATATGTGACATTCAACGAAGGCTTCTTTCACTCCTGAAAGATATTAGTTCTTCACTGTTACCAGCAGAGGATGACATATTAAATAAAACCAACATTAAGCTGTTAGTTGAAGGTGCTCGTACTGCAAAAGATGGAGACACCCGCAACCGTGTATTTTCACTTTTATCCTCTATTGCAAAGATCAGCCCAGTCAAAGTTTTTGACCATATACCCGACATTTTCAAGGTTATTGGAGAGTCTGCAGTGACACAGGTTGACAGCCACTCTCAACAGGTGTGTGAGGATCTTCTTTCTTCAATAGTTCCATGTTGGTTATCTGTAACAGACGGCACGGATAAATTGCTTCAGATTTTTGTAGATGTATTGCCTGAAGTTGCCGAGCATAGAAGACTTCCAATTATTGTATACCTATTAAGGACTTTGGGAGAATCTAATAGCTTGGCATCACTGCTTGTCCTCCTTTTCCGGCCATTGGTGTTGAAAGGGTCAACTTCAAATATACACAGAGAACAGGAGTTCTTATTTGCAGTGCGTGTATATGAGCAATATTCAAGCATCACATGGCTTCCGTCTCTAGTTATGCTGCTTCAGAAAATAGGTGGTGGTAATCCATGCCAAGAACTGTTTGTGAAATTGCTAATTTCAATGCAATTTATTTTACACAAGTTGCAAGATTCTGAGTTTATTTTCAAGCTTGAATCAAGGGAAGATTCAGATAACATTCAGAGAACACTTGGAGAAATTATGGAGCAGGTGGTTTCACTTTTACAACTCACTGACTCAAGAAGAGAGCAAATAGATGTTCCTGTTTCCATTAGGAAAGAAGTAAAGGAGTGTATGCATACTGTCTTGAGAACTATTACAAATGTCATGATCCCCTCGGCATACTTCAGATGTATCATAAAATTGCTTGGCCATGCAGATACAAATGCGAGGAAGAAGGCTCTGGGGCTTCTATTTGAAACAATGAAGGAACACGACACAACTAAAATGAAGCTTAAGCAGAAGAGGGAGACAAAATCTGATCGTTGGCTTCATATGGATGAGAGTGCTTTAGAATCCTTTGACAAGATGTGTTTGGAAATTGTTGAGTTAGTTAATGATTCTATTGATGATTCAGCCACACCCTTAAAATTGGCTGCCATTTCAGCACTTGAAGTTCTGGCCAACAAGTTCTCTTCTAATTATTCCATTTTCAGCAAGTGCCTTGCATCTATTGCGAAAAACATTGGTTCAGAGAACTCGGCTGTTGCTTCCAGCTGCCTTCGAACATCTGGTGCCTTGATCAATGTGCTTGGGCTAAGGGCACTGTCTGAGCTTCCTCACATTATGGAGACTATGCTAAAGAGGTCTAGTGATAGCACATCCATTGGCTTGTCAAGTTCTAGGGAATCACTTATGCTCTCTATTCTCGTCACTTTGGAGGCAGTTATAGACAAACTCGGTGGTTTTCTGAACCCGTATTTAGGAGAAATAATAGGACTTGTGGTGCTCAAACCTGAATATGTTTCTGAATCAAATTTAAAGCTGAAATCGAAAGCTGATGTTGTGCGGAGTCTCCTCACCGACAAAGTTCATATTCGACTCGCTCTTCCATCCATGCTGAAGCTATACTCCGAAGCCGTTAAGTCTGGGGATTCAAGCTTGGCCATCTTTTTCGAAATGCTCGCAAACTTGGTAGTTGCAATGGACCGGTCCTCTGTCAATGGTTACCATGTAAAGATATTTGACCTGTGCTTGTCAGCCCTTGATCTTCGCCGTCAACACCAAATCTCAATTACCAACATTAATACTGTAGAGAAAAGTGTCATCAATGCAATGATTACTTTGACAATGAAACTTACTGAGACAATGTTTAAGCCTCTTTTTATTAAGAGCATTGAATGGGCAGAGTTAGACGTGGAAGGAAGCACAAATATAGACAGGGCTATTTCTTTCTATAGCTTAGTAAGTAAAATCGCCGAGAGCCATCGATCTTTATTTGTACCATATTTCAAGTACTTGCTCGAGAGTTGTATACGATATCTTACTGATGGTGCTGAAGATGCGAAAAGTAATGGTTTGATTCGAAAGAAAAAGAAGGCCAAACTTGTGGAAGCAAATAAAGAGGGATACGAAGGTGTATTATCGCTTGGAAAATGGCATATTAGAGCTTTGGTCTTATCATCTTTACAAAAATGCTTCCTTTACGATACTGGGAGCTTGAAGTTTCTGGACTCATCAAATTTCCAGGCTTTGTTAAAACCCATTGTTTCACAACTTCTTGTGGAACCACCAGATTGTTTAGAACAGCATTCAGAAATACCTTCAATTAATGAAATTGATGACTTGTTGGTTTCTTGTGTTGGACAAATGGCTGTTACTGCTGGAACTGATACTCTATGGAAACCTTTGAATCACGAGGTGTTGATGCAAACACGGAGTGAAAAGGTTCGTAGCCGAGTTTTGGGTTTGAGAATTGTGAAGAATTTTGTGGAGCGGCTGAGAGAAGAATTTCTGGTATTACTTCCCGAAACGATACCCTTTCTTGGTGAATTGCTCGAAGATGTGGAGCTACCTGTTAAATCACTTGCACAAGAGATCCTTAAAGAAATGGAGACAATGAGCGGTGAAAACCTTGAAGAATACCTCAACTAAAATCCCCGAGCGAGTTATGTATATTTACAAAAGTGAAGTTGTAAAAGGCTTTTTAACTGGATGTTTATTGTACAAGTTATTGATGGGCTGACAAGTTGAGTTACTGAGAGGGGGTACAAATGGCAGTTTTGTTGTACGAGGTTTTTTTTTTCTCTTCTTCTTGGTAGCCATAGAAATATTCTAGAACCAAGCTATTGTTGCCTCCCTCGTGTGGGTGTACAGTGTACTTATGGATGCATAGTGAAGGCTAAATTTTTGGGGATTTTGATTCAAAAAAAAAAAAAAAAAAAAAAAAAAAAAGT

>transcript_33 full_length_coverage=2;length=6796

GGGGCTCCTCTTCTCTCACTGCAACTTCCATGGCGAAGAGATTCGTAAGGGAGAACGTACCTCTATCAAGATTCGGAGTGTTGGTGGCACAGCTGGAGTCAATCGTTGCCTCCGCAGCTCAACAGCCTCCGGACGCCCTTCTTTGCTTCGATCTCCTTTCTGATCTTCTATCCTCCATTGATGAAGAACCAAAGGAATCAATTTTGCTATGGCAGAGGAAATGTGAGGATGCACTTTATTCCCTGCTTATTCTTGGGGCTCGGAGGCCAGTGCGACATTTGGCATCCGTCGCAATGGCAAGGATAATAGCAAAGGGGGATGGAATTTCAATATACTCGAGAGCGAGCAGCCTCCAAGGATTTCTTTCTGATGGAAAGAGAAGCGAGCCTCTGCGAGTTGCTGGTGCTGCACAATGCTTGGGAGAACTATATCACCTTTTTGGGAGAAGGATCACTTCTGGTTTACTTGAAACGACAATTATTGCAACAAAATTAATGAAGTTTCATGAGGACTTCGTAAGACAAGAAGCTCTTAATATGCTTAAGAATGCTTTGGAAGGCTCTGCTAGTAGTGGTGCTTCTTCAGCATATACTGAGGCATTCCGTCTAATCATGCGGTTTGGTGTTGGGGATAAATCATTTGTTGTTAGAATAGCTGCTGCAAGATGTCTGAAGGCATTTGCCAACATTGGGGGGCCGGGTTTAGGAGTTGGAGAACTTGACAATGCAGCTTCTTATTGTGTTAAGGCTCTTGAGGATCCCGTTTCATCTGTCCGGGATGCCTTTGCTGAAGCTTTGGGCATGTTGCTTGCTCTTGGAATGAATCCTGAGGCGCAGGTGCAACCAAGGGGAAAAGTTCATTCCACTCCACCAAAGAAACTTGAAGGTGGTTTGCAGAGGCATTTTTCATTGCCCTTCATTAAAGCAAGCGGAGTTCGGTCGAAGGATTTGCGAATTGGCCTGACCTTGTCATGGGTATTCTTTTTACAGGCCACTCGTCTAAAGTATCTTCATCCGGATAGTGAGCTTCAAAACTATGCATTGCAGATTATGGGCATGCTTGGGGTGGATACTTCTGTTGATGCCCATGCTCTGGCATGTGTTCTCTACATTCTTCGGGTTGGCCTAATTGATCAGATGACAGAACCTACTCAAAGGAATTTTTTAGCTTTGTTGGGAGAGCAGCTTCTGTCTCCTGATGTTAGTCCTTCCGTGGAAGTTTCGGTTTTACGTACCTTGTCGTATACTCTTAAAACACTGGGAGAGGTTCCACTTGAATTAAAAGAAGTTCTTGATAACACAGTTGTTGCAGCAGTATCTCATTCTTCACAACTTGTTCGTATCGAGGCTGCTTTGACGTTACGTGCACTGGCCGAGGTTGATCCCACTTGTGTTGGTGCTTTGGTTTCATATGGGGTGACTACACTAAGTGCTTTAAGAGAGAATATTTCATTTGAAAAGGGGACCAATTTAAAAATTGAGCTTGATTCTTTGCATGGGCAAGCTACAGTTTTAGCAGCGTTGGTATCCATATCACCGAAGTTACCACTTGGCTATCCAGCCCGATTACCCAGATCAGTGTTTGAAGTTTCAAAGAAAATGCTGACAGAATCCAGTCGAAATCCAGTTGCTGCTACGGTTGAAACAGAAGCTGGATGGTTACTTTTATCATCACTATTGGCTTCTATGCCAAAGGAGGAGCTTGAAGACCAGGTCTTTGATATTCTGTCCTTATGGGCGTCGCTGTTTAGTGGGAATGCAGAATATCAAATTAAGCAAAATGAAAATTTAACATCTAGAGTGTATGTGTGGTCTGCTGCTGTTAATGCACTCATGGCATTCGTAAAATGCTTTCTTTCTTCTAATTCTGTGGAGATTGGGATTTTACTTCAGCCTGTGCTAGTTTATCTTAGTGGGGCTTTGTCGTACATATCTTTGATTGCTTCCAAGGAACTGCCAAATATGAACCCTGCTATGGAAATATTCATCATTAGAACATTAATAGCTTATCAATCCCTGCCAGATCCTATGGCGTATAAAAGTGATCATCCCCAGATCATTCATTTATGCACAACTCCTTTTAGGGATGCTTTTAGTTGTGAGGAAAGCTCATGCCTGAGGTTGTTATTAGACAAAAGAGATGCATGGTTGGGTCCTTGGATTCCTGGAAGGGACTGGTTTGAGGATGAATTTCGTGCTTTTCATGGTGGAAAAGATGGACTTATGCCTTGTGTATGGAGAATGAAACCTCTAGTTTTCCTCAGCCGGAGACTATAAGCAAGACGCTAGTCAATCAGATGCTCCTTTGCTTTGGAATTATGTTTGCTTCTCAAGATAATAGTGGGATGCTGTCCCTTCTTGGCATGATTGATCAATGTCTGAAATCTGGTAAGAAGCAGCCATGGCATGCAGCCAATATGACCAATATATGCGCGGGATTACTTTCCGGACTTAAGGCTTTGCTTACTTTACGCCCGCAACCGCTGGTTTGGAGGTGTTAAGTTTAGCACAAACTATATTTCAGAGCATTCTGTCAGAGGGTGGAATTTGTGCATCACAGCGTAGAGCATCATCAGAGGGTCTTGGTCTTTTAGCTCGCCTAGGAAATGATATCTTTACTGCAAGAATGACAAGGTTGTTGCTTGGTGAGCTTAGCGGGGCAACCGATTCAAACTATGCTGGATCAATTGCTTTTGCACTTGGCTGTATTCATCGCAGTGCAGGTGGAATGGCATTGTCAACTTTAGTACCTGCTACGGTGAGCTCACTTTCTTCACTGGCTAAAAGTTCAGTGGCTGATCTACAGATCTGGTCTTTGCACGGACTTCTTTTGACCATTGAAGCAGCTGGACCATCCTACGTATCTCAAGTTCAGGCGACACTTGGCCTTGCATTGGATATTCTTTTGTCTGAGGAGAATGGACGTGTGGACCTTCAGCAGGGAGTAGGCCGCCTTATAAATGCTATTGTTGCTGTTCTTGGTCCTGAGCTTGCTCCTGGCAGCATCTTTTTCTCACGATGCAAGTCTGTTGTTGCCGAGATAAGCTCCTGGCAAGAAACAGCAACACTTCTCGAAAGCGTCAGATTTACACAACAACTTGTCCTTTTTGCCCCACAAGCTGTTTCAGTGCATTCCCATGTGCAAACTCTTCTCCGAACTCTATCCTCAAGACAGCCGACACTTAGGCATCTGGCAATCTCCACTCTGCGGCATCTCATAGAAAAGGACCCAGTTTCCATTATCAATGAGCAAATTGAAGACAACTTATTCCATATGGTAGATGAAGAAACAGATTCTGAGATTGGAAATTTGGTGCGGTCCACAATAATGCGATTGCTTTATGCATCATGCCCTTCACGACCGTCTCATTGGATATCTATATGTCATAATATGGTTCTTGCTACCTCAACTAGAAGAAATGCTGGAATGAATAACATAGAATATGATCCTTCCGATGGTGACACAAGGTTGAATTCTGGAGAAGATGATGAAAACATGGTTTCTAGCTCTCAAGGCATACCAATGCAAGGTTACGGATTTGATGCTTCCGGTATTAATGCTAATAGAGAAAAGCACCTCAGATACAGAACCAAAGTTTTTGCTGCAGAGTGTTTGAGTAATCTACCTACAGCTGTTGGAAGAAATCCTGCTCATTTCGACCTCGCATTGGCAAGGAGACAACCTGCCGATGGACCATCTTCCGATTGGCTAGTTTTCCATATACAAGAGCTTATATCACTTGCTTATCAGATAAGCACAATCCAGGTTGAAAACATTCAGCCAATTGGTGTGGGACTCCTGAGTACCATTGTGGACAAGTTCGAAATGATATCAGACCCTGAGCTTCCAGGGCACCTTTTATTGGAACAGTATCAGGCTCAACTAGTGTCTGCAGTTCGCACTGCCTTAGATTCATCATCTGGCCCTATTCTTTTGGAAGCAGGCTTGCAGCTGGCTACTAAGATACTGACGAGTGGAATAATTAGTGGGGATCAAGTTGCAGTTAAGCGTATCTTTTCATTGATTTCACGCCCATTAAACGAATTTGAGGACCTTTATTATCCCTCATTTGCCGAATGGGTTTCATGCAAGATAAAAATAAGGCTTTTAGCTGCTCATGCCTCTCTCAAATGCTATACATATGCATTCTTAAGGAGACACCAAAGTGGTGTTCCAAATGAGTATTTGGCACTATTACCATTGTTTTCAAAAAGCTCGACTACTCTGGGGAAGTATTGGATCTGGATTCTGAAGGACTATAGTTATATTTGCTTCCGCATGTATCTTAACAGAAATTGGAAACCATTCCTGGATGGAATTCAATCACCACTGGTTTCATCAAAATTACGTCCATGTTTAGAAGAAGCCTGGCCAGTAATTTTGCAAGCGCTTTCACTTGATGCAATTCCTGTGAATTGTGATATGAATGGATCTTCCGAATCAACTTCTGAGAGTATATCAAATAATACTTTGATATCTGGATACAGCATGGTTGAACTGGAGTTGGAAGAATTTCGGTTTCTATGGGGATTCTCCCTACTTGTTTTATTTCAAAGACAACATCCAGCCCTTGGTGTACACATAATACCATTGTCTTCTGCTAAAGCTAAATTTGGTGGAGACTCACCAGTTGAAACAAATTCTCCAAGTTTAAAGTTACACGAAATTGTGCTACCGGTGTTCCAGTTTCTCTCAACAGAATTGTTTTTCAGTATGGGGTTTCTTACTATTGATTTATGTCGAGAACTGCTGCAGGTTTTCTCATATTCGGTTTCCATGGAAGATTCATGGGATAGTCTTGCAATATCAGTTTTATCACAGGTTGTGCGGAACTGCCCTGGGGATTTTGTTGGAGAAGAAAGCTTTGTTTGTCCTGCGATGGAACTTTGTTTGGCCTACATTTTCAAAGTCTATCATATTGCTGCTGCAGTTATGCCAGAACACCCAAACTGGGAGGATGCGATTTCTCCCTTATTTACTACAGCGAAGACACTACTGAGTCGTTTTGAGCCTGAAAAGCAAATAAAATCGTTGTTGGCGTTTCTGTTAATTGGTTACAAATGCATTAGAGGAGCTTCAACTGAGTTATCTTTTTCAAAAGTTAATGATTTTGTCCAATATACCAGTTCCTTATTGAAGAAACATATTGAAGATAAATCTAAACTTGATGATGATGCTATTCTCAATTTGAGAACAATCCTTAGTGCTTGTTTAAATGCAATTGTTAGTTTGACTAATGATTGCATCGAGGGCATTCGTTTACTGGAGAATAAGAGGACTGATTTACGGAAACTGCTGCAATTGAAGCTTGCTTTTTCTCTTGAACAAGCTATTTTGTTTGCCACACTGTTTCATGAAATTGAATGTCATGGAGAGAGGAAAGAGAGTAACCCAGACCTTTTTACTATGTTCAAGCATTCCACATTATGTATCCAAGCTGTTCTTATTAATTCAGATATACAGGTTCAAGCAATTGGATTACAAATGCTAAAAAGCATTGTACAAAAGAGTACCAATGTAGAAAACAACACTTTTCTTGTGTTCTTCATTGGGGAATTTTTCAGAGATATTTTCGCCATAATCGTAACGAGGCTGAAGAAGCCCATAACAAGGGAATCTGTGGCTGTTGTGGGCGAGTGCTTGAGAATCTTGGTGCTTGTACAAACACTGGCTAAAGCTAGTGAATGTCAGAGAGGTCTCATGAATCTGCTTCTAGACTCCATTGTCATGGTTTTCTTGGCAACCGAAGATGATGGTTCTCAGGAAGTATATGATATAAGAAGCACAGCTGCAAGGCTTGTTTCTCATCTTGCTCAAATTCCTTCTTCAGCAGTTCACTTCAAGGATGTTTTGGTAGCAATGCCTGTGGCACACAGGCAGCAGATTCAGGGAATTATCCGTGCTTCTGTGGCACAAGATCAAAGTCCAGCACAAACAACAACATCCGCTCCTTCCCCGGCTTTAGTAATAAAGCTGCCTTCACAAACAGAACAAAAGAATTCTCCTTCAGCTCAGGCGAATGTGAATTTTACAAAAGAGGAAGATGAAGATGAAAAGGAAGAGGAGGAAGAAGATGATTGGGATGCTTTCCAGTCTTTTCCTGCCTCTACAGATCCAACTTCTACAAACTCAAAATTCGAAAGTGCTTCTGATGCTGAAGAGCCTACCTTATCTGAGAACCCTTCCATTTCAGATTCTATCCAAGTAAAAGATATCGATATCACCAATGAAGACCATCAAAAGGCTGTTGAAGAGAATGTGGTATCTGAATCAGTAAATAACCCTGAATCAGAAAATAACTTGAATGAGATCAAACAACAGGATGATTCCCATAGTAATCGAGAGAAAGAAGATGCAGCCGTGGCAAGTCAAGAAACAAATCAAGTTTCATCTGATTTACAACCTGTTGAAAATGCAGAAGGATCAATTAAAGTGGAGTTAGTGGAGGGGGGAAATCAAGGAGGTACTAAGTGATAAAAGTGGCCCTCAAGTGTCCTCCGATCCTTCATCTTTTAAACAGGCTTCTGATAATGAAACAGATTAGTGGTGTAGAAATCAAAGAAGAAATGCTGGAGACGTTTTGACTGTTGAAAATATAGCACTTTGTTTTGAAATACAAATTGATAGCTCGTTACTTTCGTGAGAGACTAGTGGGCTCGTGGCTAAAGAGTCAAGTCTCTGGACTCCCCTAGCTGTACTTTTTAACTACATGGCTAGTTATTTTGGTCACGTATAATTTTTCAACGTATCTAAAACTATCATTAAAAAAAAAAAAAAAAAAAAAAAAAAAAAAAGT

>transcript_34 full_length_coverage=3;length=6793

GGGGTGTTCCCTTTTTTGAAAACTTATAACAATACACGAGCGGAAGCCACTCATCTTCATCTTCAACTTCAGAAAAGCGGCTACCTCCATTGACCATTGAATAGACGAACACAATCGCCGGAGGCACGGGGCGCTGCAAAAATACCATATTTATTTGGGACACACCAACGAGCACACACCAACTAGCACAGACGGAAAGGCAGAAACCACAATTGGAAACTAGGGTTTTGTCAAATTTGGGAATTTTGCAAAATCAGGAAACAGCTTTACTACAGGGTCTGTAAATAAGGAGTGTTACTATATTCTCTGCCCTCTTTTCCTCCATCTTTCTTTCTCTATATATATCAGGCCCGGATTCATTGTGAAGAAGATCTGTTAAAGCATGGGTCGTCCTGAGCCGTGTGTGTTGTTTGCTCAGACATTCGTACACCCTCAACTCGATGAGTACGTCGATGAGGTAATATTTGCTGAACCTATTGTCATCACTGCTTGCGAGTTGCTTGAACAGAGCGCTTCATCGGCATCTACAGCTGTGACACTTCTTGGGGCTACTTCGCCTCCTTCATTTGCTTTGGAGGTTTTTGTTCAATGTGAAGGAGAGACAAGGTTTAGGCGCCTCTGTCAACCATTTCTGTATTCTCATTCTTCATCAAATGTTCTAGAAGTCGAGGCAATAGTGACTAGTCATCTGGTTGTAAGGGGTAGCTATCGCAGCCTCAGCTTGATCATCTATGGGAACACAGCAGAAGATTTGGGGCAATTCAAAATAGGTGTTGATTTAGATAGTAGTTTGACTAATCTTGTTTGTTCTACTGAGGGAAAGCTTGATGACCTCCCTCCCGCATTACGTTCAAGTAATCTTGCAATTGAAGATTCCATATCCTCTCTCAAGGCATTAACTCTACGAGTTGCTCCATTGGATATATCTGTTGAAACGAAGCAATTTTTACAGTTGCTGATTAAGGTTTTGGAGTTGACAAACTTAGGGGATGCAATCTATAACGTTGTTAGTACTGTTGTGTCGTCAGCTTCTTACGTCAGCCGTGACCTGCCATGCACAACAATTCATCAGAAACAGTTCACACGTGGAAGATCCAGTTTTTATGAAGAGTCTCAGCATGGCATTATTGTGGCCAGGAAAGAGCTTCTTGATCTATATAGAGATTTTAATTACAATTCAGATAATATATCCTCGGAATCATTGGCAGAGTGTACTTTTCTTGAATCTGAGGCTGATTTGGCTACTTCGAAACAGTTGATGGATTTGTTAAACCAGTACTTTCATTTTGAAAGTAACTTCTTAAATGTTGGACCAAATAAGTTATCACAGAATAAAAACATCATTCTTGGGTTGAGTGTGGCTCTTTTGTTGTGCTCTGGTAGGGAGGGCTGCTTTCACTTTGTTAATGGTGGTGGAATGGAACAGCTTGCACGTGTTTTTAGTCATGACTTGCAAAATTCTACTGCTGTTACATTAATGCTACTTGGAGTTGTTGAGCAGGCTACTCGGCATTCAATTGGGTGTGAGGGATTTTTAGGTTGGTGGCCTCGAGAAGATGAAAATGTCCCCTCTGGTATTAGTGAAGGTTACAGTCAATTATTGAAGTTACTTATGCAAAAACAGCGTCATGATGTTGCTGCTATTGCAACTTACGTCCTTCATCGTCTGCGTTTATATGAAGTTGCTACCAGATATGAGTGTGCAGTATTGTCTGTTTTGGGAGGTCTTTCTGCTGTTGGTAAGGTTACAAGTGTTACTTTGGACATGCTCATCAGTGCCAAGTCACAACTAAAATTCTTCTTGAAATTGATAAATGCACGTGGTCCGATTGAAGATCCTTCTCCAGTAGCTTGTGCAAGCAGATCATTAATTCTTGGTCATACGGAAGGATTGTTATCTTATAAAGCAACCAGTGGCTTGATTGCTTCATCAAATTGTTGTTTTTCAACTTCAGATATAGACTCACATTTACTATCACTTCTCAAGGAGAGGGGATTCCTTCCTTTGTCAGCTGCACTGCTGTCATCTACTATTTTGCGTTCCGAAGTGGGTCATTCTTTGGACATATTTGTTGATATTGCATCATTGGTTGAGGGCATAATTCTATCACTTCTCTTTTGTCGCCCAGGCTTAATCTTCTTACTGAATCACTCTGAGCTTTCTGCCACCCTAATTCTTGCCCTGAGGGGTGTTGATGATATGAACAAGGAGGAGTGTGTCCCACTCCGATATGCATCTGTCCTAATATCCAAGGGTTTCTTTTGTCGCCCTAGTGAAGTTGGAATGATTGTTGAGATGCATTTGAGAGTGGTTTATGCAATAGACCGTTTGCTTACATCATCTTCACAGTCCGAAGAATTTTTATGGGTGCTATGGGAGCTTTGTGGACTTTCAAGGTCTGATTGTGGACGTCAAGCTGTGTTGGCTATAGGATATTTTCCGGAGGCTATTTCTGTTTTGATGGAAGCATTGCATTCTGTCAAGGAATTGGAGCCTGCCGCCATGACCAGTGGAACCTCACCGTTAAACATCGCGATCTTTCATTCAGTTGCTGAAATTTTTGAAGTCCTTGTTACTGATTCTACTGCGTCTTCTCTGGCTTCTTGGATTGAACATGCTATGGAAATTTATAGGGCTTTACATTCGTCTTCCCCTGGGTCCAATAGAAAGGACGCCCCCACGAGGCTGTTGGAATGGATAGATGCTGGTGTAGTTTACCACAAAAATGGGGCCGTTGGTCTTCTTCAGTATATTGCTATATTAGCTTTTGATGGGGATGCCCACTTAGGTTTGACGAGCGTTCTAGTATCAGATCCAATGGACGTTGAGAATACAGTTGGTGACTCTTCCTCCGGTTCTGATGTTCATGTTATGGAGAACCTTGCAAAATTAATTTCGGAGAAGTCATTTGATGGTCAATCCCTTCGAGACTCTTCAGTGACACAGTTAACGACAGCATTTCGTATTTTGGCTTTCATTTCGGAAAATCCGGCTGTTGCTGCAACTCTGTATGATGAAGGGGCCATAACTGTTGTCTGTGCGGTTCTTCTCAATTGTGGATTCATGCTTGAAAGGTCCTCCAACAACTATGATTACCTTGTTGACGAGGGTACAGAGTGCAATTCTACTTCAGAATTACTCTCGGAACGTAATCGCGAACAGAGCTTAGTTGATCTATTGATTCCTTCTTTGGTACTCTTGATCACCCTTTTGCAGAAACTACGGGAAGCTAAGGAGGAGCATAGAAATACAAAATTAATGAATGCTTTGTTGCGGCTGCATCGTGAAGTAAGCCCAAAGTTAGCTGCATGTGCAGCAGATTTATCCTCCCCTTGTCCTGATTCTGCCCTCGGTTTTGGAGCTGTCTGCCATCTTCTTGTGTCTGCACTTGCTTGTTGGCCAACATATGGTTGGACTCCTGGTCTTTTCCATTCTCTCCTTGCCAATGTTCAAGCTACTTCATTGCTGGCATTTGGTCCAAAGGAAACCTGCAGTTTGCTTTGTCTTCTGAATGACTTATTTCCTGAAGAAGGCATTTGGCTTTGGAAGAATGGGATGCCTCTGTTAAGTGCTTTGAGAACATTGGCTGTTGGGACTTTATTAGGGCCTCAGAAGGAGAAGCAGGTTGATTGGTACCTGCAGCCTGGATATCTTAAAATACTGCTTAATCAGTTGACCCCACAGCTTGAGAAAATTGCACAAATAATTTTACATTATTCTGTTAGTGCATTGGTGGTTATACAAGACATGCTTCGAGTTTTCATAATCCGCATTGCTTGCCAAAAAGCTGATCACGCTTCTGTTCTTCTGCGACCCATATTTTTATGGATTCATGATTGCCTTTCTTATTCGTCTTCTTTGTCAGACACAGATACTTACAAGGTTTACAGATTGCTCAATTTTCTAGCTAGCTTGTTGGAGCATCCCCGTGCCAAGCCGCTATTATTGAAGGAGGGTGCCGTTCAGATGCTTATTAAAGTTCTAGAGAGGTGCAGTGGTACTGTTATTTCAGATGAAAAACAACTTCCAGACAGTAGAAATTCATCTACATGCGGGTTCACTCAACTTAGTTGGTGCCTCCCTGTATTCAAGTCTTTCTTATTAATTTTTGATTCTCGGACATCTCTTCATTATCCTGGGAGATCTAATAAGCTCAATTTTGAAAGTTTTATCGAGGATTGTACGTTGGTCTTTCCTCATCTTTTTAGGTTATGCCAGGTCCTGCCAGTTGGAAAAGAATTACTAGCTTGTCTAACGACTTTTAAAGAACTGGGTTCTCGCAGTGTGGGTCAAAGTGCTCTGATGGTCATTTTTTCACGTGTCTGCTCCTCTACCAGTGAAGAACTAGAGTCGGAGAGAGGGTACGATAGGGATGGCAATTGTAATCTTTCTACCAAATTTGAATGGAGAAATCGTCCTCCTTTGCTCTGTTGCTGGAAGAAGTTATTGAGATCAGTTGATTCAAAGGATGTCATATCAAGCTATGCAATTGAGGCTGTTGGTGCATTATCGTTAGGTGCTTTGCTCTTTTCAATAGATGACAATAAATTGAACTTGAACAGGGTTGCTGCATTAAAATGCCTTTTTGGGCTTCCTTATGACATGGGTCTGACGGATGGTTTTCCTGAAGAAAACATAAAGTTCATAATTGAAATGACAACTCTTTTAACTTCAATGATCAGAGATGATGATTATTCAACTACCTCTAATATGAAGAGCACTTTGTATCAGGTTTTGGAGTATACAAAATCATTCTTGTCATTGATGGAAAACCCCATCGGTTCAGTTTTAGTGAAGGATATCATTTCTCCCGAGGTTCTAGTTTCTTCAAAGCTACATCAGACAACAGATGGCAGTGTTGAAAAGTTTGATGAATTTTTACAGCTAGGACTTCGGGACAAGTTTCTGTGGGAATGTCCAGAAACTTTACCCGATAGATTAACTCAAACCACTGGTTCCTTGAAAAGGAAAACACCAACACTAGAAGGAGGACCAAGCAGGCGTGCCAGAGGAGACAATCCTCCTGCTGAGATCATGGTTCAGAACACATTTTCACGGGGACCAGGACTGCCCATTACCCCTTCTGCCCCTACTCGTAGGGATACCTTTCGGCAACGCAAGCCAAATACCAGCAGGCCTCCTTCTATGCACGTGGATGATTACGTTGCTAGAGAAAGAAGTGTAGATGGTAATGTTAGCTCTAACGTAATTGCTGTCCAACGAGTTGGACCGAGTGGAGGAAGAGCTCCGTCAATTCATGTGGACGAATTCATTGCCAGACAAAAGGAACGTCAGAATTCCATAGCATCCGTATCGGGTGAAAATACAGCTCAGGAGAAGAACGTGACTCCCGAGAATGACAGTGTTACCGAAAAATCCAACAAATCTAGTCGGTTAAAAGCAGATCTTGACGATGACCTTCAGGGAATTGATATCGTCTTTGATGGCGAGGAGTCTGAATCCGATGACAAATTACCCTTTCCTCAACCAGACGATACTCTACAACAACCCGCCCCAGTCATAATGGATCAAAATTCTCCCCACTCCATCGTTGAAGAAACCGAGAGCGACGTGAATGAAAGTAGTCAGTTTTCCCATTTATCTACACCATTAGCATCTAACCTGGACGAAAATTCTCAAAGTGAGTTTTCATCTTCACGTCCTTTGGTTCGAGAACAAAGTGTCAACTCAGAGAAGAAGTACGATCCATCGGTTAAAACATCTAGCGGGTTTGATCCTTCTGTATCAGCAAGTAGCTCTGGATTTCCTGCTTCGCTCTATAACAACAAGCTTTCGGCAACATCTTCCATGCCTCCCCCTAATTTCTACTCAAAACAATCTTCGGTTCAACCACCTTTACCCCCGATGCCACCTCCACAAGCAGTAATATCTCAAGGTCCAGATCATGTCCCAACTCAATCATCTCCATATCTCTCCGGATTTAACAACACTTCTGTTTCCATGTCACGAACTTCTATTACTTCTCCAAGTGGGTCTGTCCGACCTTCACCGCCACTTCCTTCAACACCGCCACCTTTTATCAAACCTTCGACTTCCCAACCTTACAATCAACCATCAGGGTCAAGACTTAATAGTTATCCACTACCTCCAATGATGCCAAACATGGCTTTCAATAGGCCACCGGGTTCAATTCAGCTGCAACCACTTCAGCCACCACAGGTTCCACGACCTGCTCCTCAGCCACCTCAGCATATTAGGCCGCCGAATCAAGAACAAGGGGTGTCTGTTCAACTTCAGCAAGTACAGATATTACAGCAGCCACAACATGTATATTATCAAACTGTACAAGAGAGCTATAGTACACCACATGTTCAGCAACAGCAACAGCAACAGCAACAGCAACAACAAGAAGTTGAAGTCATGAATCAGCAAGAGCAACCACAAGATCCTTCCGGAATGTCGTTGCAACACTTTTTTAGTTCTCCAGAAGCTATTCAGTCTTTATTGGGTGATCGGGATAAGCTTTGCCAGCTTTTGGAGCAGCATCCAAAGTTAATGCAGATGCTTCAGGAAAGGTTGGGTCATCTATAGAGACTGTATGCGGATCTTCTAGCATTCAGATGTGTACACTAGTGATTACTCCATGTATATAAAGTCATGTGGGCTTCTTGATGTGTTGTTAAATGTGACATTTTTTGTTGTAACAATGTAATATAGATTTATATTTGGTTTATTTGTTGTTAAAATTGGGAGTTTCCCAATGCCACTTTATATCAATTTCTTTTATTTCTTGCAAGAAAAAAAAAAAAAAAAAAAAAAAAAAAAAAAAGT

>transcript_35 full_length_coverage=2;length=6783

GGATCGCGCACAGAAAGAATCTAGAGAGAGAAACTCATTTATATACAAATACACGACGCTGTGGAGAGAAATAGTGAGGGATGGGTGAGAGCTCAACTGGGGCTCTTGTGCCCACCGTAAAAACAGAGCCGCCACCATCACCATCACCATCACCACTTTCGCCACCACAACCGACGATGACGACGGAAGAAGTAGTAATCGCAGATACAGAGAATGGGGAGCCAGAGCTTCTAGACAAGGATTTGTTGTGTCCCATATGCATGCAGATAATCAAAGACGCGTTTCTCACATCTTGTGGACATAGCTTTTGTTATATGTGCATTGTCACTCATCTACGGAACAAGAACGATTGTCCTTGCTGTGGCCATTACCTCACCAGCAACCAACTATTCCCTAATTTTCTCCTCAATAAGTTATTGAAGAAAACCTCGGCTCGTCAAATAGCAAAATCGGCATCTCCTTTTGAACATTTACGACATGCATTACAACAGACAGGGTTGTGAATTGTCAGTTAAGGATCTAGACAGCCTGTTGTCTCTTCTTTCAGAGAAGAAAAGAAAGATGCAGCAAGAAGAAGCGGAGACAAACATGCAAGTTCTGCTTGACTTCTTGCATTGTTTAAGGAAGCAAAAGCTTGAAGAGCTCAATAAGATACAAACTGATCTTCAGTATATTAAGGAGGACATAAATGCTGTGGAGAGGAATAGAATAGAGTTGTATCGTGCAAGGGAGATGTACTCCATAAAACTGAGGATGCATGATAATCCCACTGCAACAAAAGCGTGGCCTTCATTGATCGATAAGCATAGCAATGGTATTATATCTAGATCCCACAGTGTACAAGGCTGGATGGGTTCTGGAGATCCACAGAACAATAAAGCTGATGTAAAGGCTCAACTTATCTCTCAGGGACTGCAAATAAAAGATGCTTACAGTGGGTCTGATTCACAACATGTTTCTCAATCAGGACTAGCTTCAGCAAGAAAAAGACAAGTCCATGCACAGTTCAATGATCTACAAGAGTGTTACTTGCAAAAGCGGCGAAACTGGACCCGTCAATCTCACAAACAGGAAGAAAGGGACACAAATGTTATGACAAGAGAAGGTTATCATGCAGGTCTAGAGGATTTTCAGTCTGTGCTAACTAGTTTTACACGATACAGCCGGTTGAGGGTCATTGCGGAACTTAAACATGTGGATCTCTTTCACTCAGCAAATATCGTGTCAAGGTGAGTTAAACTGCATTTTATAACAGACATTTGTGTTCCGGTTTAACTCCCTAGGTTGGAAAATAATCTGAAAAAGCTCTCTGATTCTATTCTGTCAGTGGATGCTGAAAAAGGAAATTCCATGGTCATAAATACTAAATACATAATACCATGGCCATTTCTGTTATTGGCAAAATGTTATCCACCACACTCTGCTATATATTGCTTGTAAGAGCATGATTTTTCTAATTACCAAGAGGAAAAATAATTCAAATTTTCTGCTAAGGAGTGAATATATGATATGTTATTATTCAATATGTTGTAAAATTAGAAATTTTGGGGGGTACCAATATTTGCTCAGGTACTAAATCTATGGAGAATTTCTTGCTCTTTTGAGTACTAATATTAGATTTTAAGCAAATGAGAAGGGAATGATGTGACTCTGTTTATCATTTAGAACGTGTGTACATACATATAGTGATAAAAGCCTAAATCAATTCCTACCTAAATCCCATGAATATAGGAAGCCTATTACAATAATATCCAATTCCTTAAATTAACCAACCGGAATTTATATGACCAGGATGATCGAATGATCCATCCATGTAGGCTCTTGGTACCAGTGAGTTCACATCAACCACTTCTGTAAATGCAATAGCCGATAAAATGTGTCTGGATAAACAAAGGGTTGTATTCGTTTGATCATACTCACAATCTCCTCTAATGTGACCATCCTAGTATGTTGAGCATTTTCTTCTCGAGTGAATTCTCTATGTCCCTTCATCATGTAGTCTCCTATATCAATATGGTTCTGACACTTAAATCTCATTTTGAAATGTTAATGAACAAGTTTGCTATCCTCATGACCTTCCAACCATATGCATATTTTATCCTTAGTGTGCCAAATCCTATTCATCCATCTTCATGGAGTTAATGAAGTTGTTAAATCAATTAATTCTACAATTTGCAACTTTAGTGCTACAAATTTTCTCTCTTTTATTACTTAATTTATTGAAATCATCCGTAATTACTAAAGTTTTGGAAAGGAGGTTGGGTTTATAAGCAATTTCAAAGAAGTAAGGCCAAACCTTGTTTAGATTCGGATTGGGGTACCCATACAAGCCAACACAGTCCCAAGAGTAATAGTATCAGTCATTAACGTTGAGCACACCAATTCTTGGATCACACCATTCAATTTTGAAGTTACTGGATGCACTAAATCATGGTCAAGTTTAACCTTTCCTTCAAAATCAATGGGTCCCGTACTTGCCAAGTCCCCCAAGTCACTGCAACTCTGATTTCCACGGGAGCAAGTTGCACTCTCTGTCTTTCCCTACTCTTTCTCTTTCTAGTCATATATTCCCTTCCTTATATTCTCCCCCCTAAAAAAAGCATAGGATTTCTTTCTTACAGTTTGAGATGAGGAAAAATAGGTGCTGGAACGGAAGGATGAATTATATAGCAGTGCATATCTGGGAGTTGTTTTTGCTGGAAATGTGGTGTGGAGACTCTCACCCTTAGTTACATTGGAGGACGAACTTTAAATAAAATAATATAAGAAAGAAATTTGGCCAGACCGCCAGATGAACAGCTTCTGGTGACCCTCCAGAAAGGCAGAATAACTCGGAGAAAAGCCTATTTCGGCACTCGGAACCTGGTGTTGGAAATAGAAAAGGAAATAATACGATTTATTTGTTTAATTTGTAAAACAAATGAATCTGATAGAATTAGAATATGTGTACCTTGCATTACTGATGTTGGAAGGGACGTCCCTGTGTTGCCATAGGTAATTAAATTGGATGCAACCTTCCTTCCCTTCTCTACCCCTTTTCTCTGTATCTCTATATTTTCTAAAAGAGTTTATATTCACGCGTGTCTGATGGAAAAACACATATGATTATATAGAGAGGGAAGGGGACTATCCGAATTTCATAAATTAACCAGGTTCATCCATCATGAACTTGAATTAGGTCCATATTAAAACTGAGTCATAACTCATCTAAATTAAGACACATGTTAATGTTAGACGAACAGAAGCTGACCAAGTCAGTTCCAATCCTGCGCAACAAAGTGATAAGAATAGTTAGCATTATCTTGTACACATGTCCCTATTATTTAGGAATAGATAAACTCTGTTGTAACAGATTGAGATGTATAAACTCTGTTGTAACAGATTGAGATGTATGTATATACTGTGGATGATCAATGAATAAAATACGAGAGTTTACATTCTGTTTAGCATTTCTTTCATGGTATCAGAGCTTAGGAAATAAATCTAGTGAGTCATGTCAAGTCCACTGTCTCCATTGTCTTCCTCTCCTTCGTCCGTTGAAAATCTGTCAAACATGTCAAGTCAAATGTCACAAAATAGCGTTATTCCCCTCTCGGGTGTCATCACAGAAAAGTTAGGTCGTTCCAACTATGTTCTTTGGCTAACCCAAATCCTTCCTGTGTTAAGAGGAAGAGGTTTAATGGGTTTGTCACCGGGAACAAACCCTGTCCACCAGAGTATATCCTAGATAACAAAGGCGAACCCACTTCCATTATGAATCCAGAACATAATATGTGGATCCAGCTCGACCAAACTGTTCTAGGAATCATAATGAATTCTGTCACTTCACCTATTCTAGGAACAACAGCCAGGAAACATACATCAGCAGATGCCTGGAAAGCGTTAGAGAAACGCTTTGCCTGTTCCTCCCAACATCGGGTCCAACAATTAATGGCTACCTTGTATCAAACCACTAGGGGAGAGTCCTCAATCACTGAGTTTCTTGAGCGTATAAACCAGGTAGCATATTTACTCGTTGTTATCGATAACCCAATTCCTGAGTCCCAACTTGTGATGATTGTTATGCAGAATGTTGGTCCCAGATTTGAGATGACCATCTCAGCTGCTCAAGCTCGTGAGACTCTCATAGGGTATGATGACCTGGCTGCGTTACTTCTTAACGCTGAGCATAGAATGAGTCAAGCTGAAACAGAACCTGTTTCACCCTCGCCTGTTGCCCTGTTTGGGTCTAAGCCACATCCAATCACTATCGCAGTTCCTCCAACCGTGGGAGAGGCCGTGGATTTTCTTTAAATAAAGGATCCACATTCAATCGTGGGAAAGGATCATCCAAACCTGCAAATTTCAACAACAACAGCCAAAACCGACCAACGTGTCAAATCTGTGAGAGAGTTGGTCACACCGCTATCGATTGTTATGATAGAATGAACACTGCATATGAAGGGAGAGAACCTACTAAAAAGCTGATGGCTATGGTCGTTGCGCCTACAAATAGTACCAATGCATCCACTTGGTATACTGATTTCGACGCCTCGCATCATGTAACCACTGATCTCGCAAACCTGGTCATACAAGAAGAGAACACAGGATCAGATAAAATAGCAGTTGGTAATGGTTCGGGTTTATCAATTCACAACTCAGGTTCAAGTTCTGTCCTTTCTCAAAATAAATTTAGCTTAAACAAATATCTTCATTGTCCTCAAGCTATCACAAATCTGTTCTCAGTACAAAGTTTGCATCTGACAATCATTGTTATTTTGTGTTTCTTCCCTCTTGTTTTTTTATCAAGGATCTCAAAACTCAGAAGACGCTTTTTAAGGGTAAATGCGTGGATGGACTTTACCCGGTTCGTTTTGATGGCTCCTGTCATTCTGCATTTCCCTCTCCTCCTCCTCTTGCACTTGTTGGAATAAAAACTGATCTCTCCATCTGGCATGCACGACTTGGTCATCCGTCACTCCCGGTCATGGCCCATCTGCAAGCAAATAAGAATTTACAAATTGTTAGTTTCAAATCTATGTCGTTTTGTAATTCTTGCCAACTTGGTAAGAGAAAGAAACTTCCTTTTTCAAATCGTGTGTCAATAAGTACAAGCCCATTAGAACTTGTACATTCTGATGTATGGCCCAGCCCTTATCTTTCTCATTTTGGAATGAAGTATTATGTGCTTTTTGTCGATGATTACTCTAGGTACTGCTGGATATTCCCTATGAAATATAAACACGAAGTACGTAATATTTTCAAAGATTTCAAAAATCTAGTTGAAAATAGATTCTCCACCAAAATCAAATCGTTTCAATCCGACGGTGGCGGTGAATATACAAAAAAGGACTTACAAAAATATTTGCTTGACAATGGCATCCATTTTCGTTCCTCCTGCCCATCTCACCCCGAACAAAATGGGTTAGCCGAACGAAAACATAGACAGGTCGTCGATACTGGCTTAACTATGCTTGCCCATTCATCCATGCCAATAAAATATTGGGATTCTGCTTTTCAAACAGCCATGTACCTCATTAACAGACTACCAACAAAAACCTTGAAATTAGCTATTCCATACACAAAATTATTTAATCTAAAACCTAAATTCGAGTTCCTACGTACCTACGGTTGTGCATGTTTTCCCTGCTTGAGATCGTACAACTCCAACAAATTACAATTTCGCTCCAAGCTCTGTGTCTTCTTGGGGTACTCTTTGCATCACCATGGTTACCGGTGTCTGGATCCTTCGACTGGTCAAATTTATCTGTCGAAACACGTGGTCTTCGACGAGCAACAATTCCCTTTTAAGAACAATTAAACGTCTTCCCGCTCCATCCCTTCCTTTAAGGAATTTGTTCTTGACAGGAACTCACCAAACGAGCCGCTAGCATCTTCGCAAGAATTTTCCCCCGTTGATCGCGTTTCTTCCTCCTTGAATCAATCTTCCACATCAGCTATTGCGGATTCTCCGCTCTTTATCTCCACACCAATTCCTGAAAACTCAAAACACGAAAGTTGTCCGGAATCGAATTTCTTATAAAAGTTGTTCTTATGACTTTTCCAAATTCTAACTTCTAATAAGCTGAAACTGTAGATTTTCAGATCTGCATCTTTCAGTCCGACCGCTCCAAATTAATCTTTTAACGGTGTTTTAAATTTGTAAACTCTTAGACATTTATTTTGTGGTCTTGTCTAATGATGTGCACACCTTCTCTAGGTTGTTCAACCGTTACCTCTTGGAAATAATTCATCCAAGTTTTGAAACCAAAACAATTATCTTATTTATATAAAGAAATACTTCCTATTCCATGGAACTCAACCATCCAAGGTACTAATAATCCCATACGCATGATGTGAAGGCCGTCGTTTTGTGGCAAACAAAATAAATGAGGTTACCTAAGTCTTGGAAATAAGCCATCCAAGTCTAGAGAAATTATATCTTGAACTCCTTAAATTGACTGGAATTAAGCCATCCAGTAGTTTTAAAAGCACGGGTACAACTGAGGGAAGCGTAAAAAAAGGTTTTACATCATTTTAATATTTTAATATTTTTCTTTGGTGTTTGAAGTGTTTTTTAAGTTTGTGGACAGCAAGTATTTAATTTCTTGAGAGCACATTAGACACTCTCATAACATCCCGGTTTACTAATCAGGTTGTTTAAACTTTAGCCCAAAGACCCGGAACCATACGGGTTCCTAAATTTTCAAAGTGTGAAAATTTTCTAGAAATTTGGACACCATTTTGGCTGTATTTTTGATATCCAAAAAAAAAAAAAAAAAAAAAAAAAAAAAAAAAAAAAGT

>transcript_36 full_length_coverage=3;length=6606

GGGGTGGAATTAAATTTACGGGACAAATTCACATTTCACACTAAACACGGAAACACAATCAAATCCAAACTCGAAGTCGTCTATTTTCAATAATCCCAAATATATCCACTCCTCTCATAACTGCAATCTTCCCCTCCCCCCTCTGTATCTGCTCTATATGGAGGTGGTGCGAGCTTCTATCGTGAGCAATTTATCTAAAAACCCAGTTCTATTAGTTTCTTTTGGCCTGACTATAATCACAATTCGAAAACCCTAATTCTTCTCGTTTCGAAGCATCGAAGATGTTTCCCTGGAACATCGCAAAGTCCGCGGAGGCGATGCTCTCCCGCTGGGCGATAAAGCGGCTGTGTAAGTTTTTGTTAAAGAAGAAATTAGGGCAATTTATTTTGGGGGATATCGATCTTGATCAGCTCGAAGTGCAGCTCAGTGCCGGCACCATCCAGCTTTCTGATCTTGCTCTTAACGTTGATTATCTCAACCAACAGTTCAGCGCAGTAGCAGCAGTGACTGTGAAAGAAGGATCAATTGGCTCCCTACTAGTTAAAATGCCTTGGAAAGGAAATGGTTGTCAGATTGAGATTGATGAACTTGAGCTTTTGCTTGCTCCTTCTGTGGACAAAAATTCTCGAGCTGGTGAGGAATCTTGTATTAACAGTCAATATAGTAACCAAAGTACCAACCATGAGGTAGGAAAGCTTGATTGTGAAATGTTGGAGAATGCTATGACATCTGGTTCCGTGGATGTCCATGAAGGTGTTAAAACAATTGCCAAGATGGTTAAATGGCTGCTTACAAGTTTTAATGTAAAAGTTAGAAAACTGATTGTTGCATTTGATCCCTGTTCCGAGAAAGATGAAAAGAAAACAGGGCAATACTCAACCTTGGTTCTTCGAATTTCTGAAGCAGAATGTGGGACATGCATTTCTGAAGATGCTGCTTCAAACAGTGATGCAAGAGCTGAGAGCTTTCTTGGATTAAGTCGACTAATGAACTTTGTAAAATTTCAAGGTGTAATTCTTGAATTGCTTCATATGGATGACGTGGATAGGTTTTCTTGTGCTCCAGGAACAACATTTGATGAATGTTTTGCATGTTGCTCCCTATCAAATGCTACAACGCCAATCATGACTGGGGAGAAAGGAGGATTTTCAGGGACACTAAAGCTAAGTATACCTTGGAAGAATGGTTCCTTAGATATCCACAAAGTGGATGCAAATGTTTCTATTGATCCTGTAAAACTAAGAATTCAACCCAGCTCAATAAAATGGTTTCTAATTTTATTAGAATCTATTAGGAGTATGGATAAAGATGGCAGGGGTCATATGCATCAGAAGTCAATGGACTCTGTTTATTTTAACATGGCATCTCACTTTTCATCATCCACTGGTTCTGCTGGTCTTGCTACTGATAAATCGACACCAATCTGTGAAAGCTTTTCTACTGAAATTTGTTCTCCAATTGAGCAAGAATTGGTAACAGATGGTCTGCTACAGGGGTCTCATCTTATATCAGATTGGGTGCCATTTTCTATCAACAAAAATCAGAAATATGGAACTGAAGAAGATTTAGATTTTGGAGAAAGCATGGATCAATTTTTTGAATGTTTTGATGGCATGAGAAGTTCTCAATCAGCTTTGGGGAGTAGTGGGATGTGGAACTGGACTTGTTCCATCTTTAGTGCAATAACGGCTGCGTCCAGCCTTGCTTCTGGATCTTTGCATGTTCCGAACGAACAGCAGCATGTTGAAACCAATTTTAAGATACTTGCTACCGGAATTTCCATTGTTTTTTCTTTCTATGATGAAGATCAGAAACACTCGTGTGATCTGAAGGGAGATCACGCTGACCCAGATTTTCATTATCTGGTTGCAGAATGCAGAGACACGAGTCTTGTTTTACAGGTATGTCCTCGAGATATGAAGGTTGAAGCAACGGTGCAGCATATTGAGCTTGCCGATTACTACTGTAATGGAAATAATGATAGGAAATTTGGCTTGCACAGTCAGACTCATTTCATGCAACATCTGCAAGATGAAGTTGAAGCTGCTCTCCCTCGATTTGCCTTGTCTTCCAGCATTCACGATGTACAAAGACCATGTCGGGTAGCTGCCACAATTTTCCCATTTATCAATTCTACAAGTATTAATGAAGACAATGTAGTTAAGGTCACATTGCTTCAGACTTCCGGTGCTTCTCATTGCCAACTTACTATAAATTCAAGTTCTGTGGATGGCACTTTAACAGGGCCAACATCATTTTCGTTGAAGCTTCCACCATTTGTGTTCTGGGTGAACTTTCATCTGATTAATATCTTATACGATTTTGTGAAGGAAGTTGGAAATTGTGTTGAAAAGAGTATCACAGGAAATGTTATTCCATCTGATGCATTGAAAGAGAATCATAAATTTCCCAAGGGGGACGTGAAAAGAGGTTCTCATCCTTGTGTTACAACATTGTCTTCAAAAGAAAGTTTACGTGGTAACATATTCCTCCTTGATGCTAGAGTAGTTTTATGCTTCCCTATTAAAAATGGTGAAGATTGTGGAGGGTACTCAATCTTGGATCAATTTATCTCTCTTGATTTTTCTTCACCATCGACTTTGAGCAAGGGAATAATTCAAGATGCTAATCAAATTTCACATGCAAGTTCCCAGAAAAGGTATTCTTCGGCTACCACTCGTGCCTTTCATTTGAATGTTGGCAACCTTGACGTTCACTTAGTCACTTCTTCTTGTAAAGATGATGTTGACATCAGCTCCTGTAATATGCAGAGGCAGAAATTTTCTGCCCAACACATATTGTCTGTTACCAGCAGTAAAAATTGTCCTTCTGTCATTAGTATGATTTGGCAGGAGGGTACGACTACTGGTCCTTGGATAGCAAAGAAAGCTAAGTCTTTAGCTAATTTAGAGGATTCGAGAAGAAGAAATAAATTCATGGGAACGGGTTATGAGTTTGCCTCTGTAACTGCAGTGAAAGATCTGGAAGACCTATATTCTCGTACAAGGCAAGAAATCATTTTGAGCTCTGCTCTATTTCTGCATGTCCATCTATCTCCCGTATCAGTTAATCTAGACAGTTCACAATATAAAGGTGTACATCTTCTTTTAGATCAGGTGATAAAAGAGTTTTCACGCTTAACCTGTGATCCAGTTGCCATTGGAGAACAATCTCCTGTGTCTCAAACAACATTTCTTGTGGAATGTGATTCTGTTGAAATTTCAATTAGCCCCGAAACAATAGGTAGTACTAATGAATCGATGCAGACTGAACTTCCTGGCTCTTGGCATTATTTGAAACTGAAAATTCAGAAGTTCAATGTGCTATCAGTCTCAGACATTGGAGGAATCAGGGGTGCCAATTTTCTTTGGGTATCCCATCGCGAAGGCAAATTGTGGGGTTCCAGTACTGGTGTTCCAGACGAAATGTTCCTTTTGATCTCGTGTTGCAACTCTACAGTGAAACGTGGTGATGGAGAAGGTTCAAATGCATTATCTTCCCGTTTTGCTGGTTCTGATATTATATACCTGTGGGAGCCGGAGAATTCCCACAGTTCTACTTCTGTAAGTGTCAGATGTGGCACAATTGTTGCAGTTGGTGGTCGCTTGGATTGGTTGGATGCAATATTCTCCTTTTTCAGTTTGCCAACTTCTGAAACTGAACAAACAGGTGAGAATAGTTTGCAGAAGGGGAATTCAAGTGTGTCTTCTGGAGCTTCTTTTGTTCTTGACTTGGTCGATATTGGGTTGAGTTATGAGCCCCACTTACAAAATTTGGGGGTCAGTGGTGAAGTTTTCGATTTTGAGTCTTCTTCTGGTAATGCCAATGAAGAAACGAGCGAATCTGTTGCTTGTTTGTTAGCTGCATCTTCCTTGAGTCTTTCTAATACAACAGTGGCAGGTTCTGTTGACAATGAGTATACAATTAGAGTACAGGATTTGGGGCTTCTTATTTGTGCAGTGTCTGGGCCCGAGAATGTTGGTGGCACTTATAGTGTACAACATCTTCACAAGGTTGGCTATGTTAAAGTTGCTGGGGAGGCACTTCTTGAAGCGATTTTAAGAACCAACTGTAAAAATGGGCTCCTCTGGGAAGTAGAATGTTCTGAATCTCACATTGATGTTGATACTTGCCATGACACCACTTCTGGCCTGATTCATTTGGCTGCTCAACTGCAACAACTTTTTGCTCCTGATATTGAGGAATCAGTTGTGCATTTGCAGACTAGGTGGAACAATGTTCAGCAGGCACAAGCGAAGAATTATTCCAGTGATGAAAGAATGATCTATAATGATGAATCTGCCCCATCAGCTTCTCAACTGCACACTTGTAGTCCGGATAGAAGCAATACACTTGGGATGGTTGGTTTGATGGATGAGATATGCGAGGATGCATTTCAATTAGATGGAAATAGGACTTTCGAATATGATTCTTGTGAATCACCACTTCATAATTCAATGGATGGGGGTTTTCTTGGAGATGCACACAACTTAACAAGATTGCAAAGTAGTCAGACCTTAATTCCTGGATATATAGAAGGCTACTGCTTATCTGAGTTACGGCATCTGTCAGAATTATCTGTATGTAGCCAGTCATCTAACGAATTTCGTAAATGCAAATCCAGAAATATGGGAAATGGAGATCTTGGAAAAGGAACTAGTGGATGGTATGGGGATAGCGCTCTAAAAATTGTTGAAAATCATATTGCAGAAGTAAGTGAGGGAATTGGCGTGAAACACTTTGTGGAAGGCAGGCTTCCTTTTATGCATCGTACAAGATCTGATGGTTCTGAAAAGGCAAAGGGACGTCTACTTCTCAAGAACATTAATGTTAGATGGAGAATGTATGCTGGTTCCGACTGGCTTGACTCTAGGAAGAGTGGCCATCATATACGTGGAAGGAATACAGCTGGCTGTTTAGAACTTGCATTATCCAGGATGGATTTTCAGTATGATCTTTTTCATGATGGTGAGGCACTTGTATCTAAGCTCTCTCTTTCTGTTCAGGATTTTCATTTTTATGACAACAGCAGAGGTGCACCATGGAAACTGGTGTTGGGATATTATGATTCAAAAAAACATCCCAGAGAATCGTTTTCGAAAGCATTCAAGCTGGACTTAGAAGCCGTCAGACCAGATCCTTCAACCCCTCTTGAGGAGTATCGGTTACATATTGCACTTCTTCCTATGCTTTTGCATCTCCATCAGAGCCAACTTGATTTTCTTATCAGCTTTTTTGGAGAGAGCACGTCAGGCGACCAGTCTCCAAGTCATCCACAGGATTTGGGTAGGTCGAAATCATTACCGGCAGAGTATGGTAATTTGGGGAGGCATACCATTGCAGTGGAGGCATTGCTGCCTTATTTCCAGAAGTTTGATATACGGCCCGTTCTTGTTCGGGTTGACTACAGTCCCTGCCGTGTTGATCTCACAGCATTAAGAGGTGGAAAGTATGTAGAACTTGTCAACCTTGTTCCATGGAAGGGAGTTGAACTACAGCTCAAACATGTTAATGGTGCCGGTGTCTTTGGTTGGAGCAGTGTGTGCGAGACAATAATAGGAGAGTGGTTGGAAGATATTTCTCAAAACCAGGTTCATAAGCTGTTAAAGGGTCTTCCTCCAATCCGATCATTGGTTGCTGTTGGTTCTGGTGCCGCTAAGCTAGTGTCTTTACCCATTAAAAACTACAAGAAGGATCAAAGATTGCTTAAGGGAATGCAAAGAGGTACAATTGCATTTCTAAGAAGTATATCACTTGAAGCTGTTGGATTGGGGGTGCATTTAGCAGCAGGAGCTCACGACATTCTACTCCAAGCAGAGTATATTCTCACAAGCGTTCCACCTTCAGTTCCATGGCCTGCGCAAAATAGAACAGGGTCAAATATAAGATCAAATCAACCTAAAGATGCCAAACAAGGAATTCAGCAGGCTTATGCGAGTCTCAGTGATGGTTTGGGGAAATCTGCTTCTGCTTTAATTCGGAAGCCATTGAAAAAATACCAGCGTGGAAGTGGTGCGGGATCTGCTTTGGCAACTGCTGTTCGAGGAGCTCCGGCAGCTGCTATAGCTCCGGCTTCAGCTGCTTTTGGTGCTGTACGTTGTGCACTTCTTGGTGTTAGAAACAGCCTTGATCCTGAACATAAGAAAGAGTCCATGGAGAAATATCACGGTCCGACACAGCCTCAGGAACGCACTTAATGATGGCAAATATTGTGAAGATGTAAGTTTTAATTTTGAAAGGTGGAGGATTGGAACCATCAAGAAGTTGTTCATGTGGTCTTTTGCTTCGGTCACGAAAGGAAGGATCATAGAGCTGCTCTTTTGTTGCTACGGTGGAATCCTCTGTTATCATGTGTACAGTTGATGAAACTAGCATACAAATTCTTTTCCGCCGAGAGGGCAAGGCGGTGATTTTTGTACAAAATGTAAATATCAAATGGGGTGCTGCTTAGTTTTTTCTTTGTTCAAGAGTAGAGTAGCATTGTAGTGGTTGTAATAGCGTGTTGATGGATAGTACGATCACTATTCTTGTTCTTCCAAAGGTTTGAATATAATTTATAAATATATGTTGCTTCAAAAAAAAAAAAAAAAAAAAAAAAAAAAAAGT

>transcript_37 full_length_coverage=2;length=6763

GGGATATATTTTTTTAAAGGGTTTCGTTCACCTTCAACCTGTTGTTGGCGCAGAAAAGCCCTAACCCCGTTTTTCTCATCCTCCTCAATTTTAACCTCAAATACTTATTCTCTAAAAGGGTATCTTAACACATCAACAGAGTTTGTGAATTTTTGGTGCCGAAGGTTAAGGCGAATGCGAGTTCAGAAACTGAGATCGTAGCCTGTGTTTGCGCCTGTGTATGCTGGGGACGAAGTGTTCGAATGAGAGAAGCTTTCACTAGGGCTTTATGGCTCAGCAGTCCTCTCGTCTTCATCGTCTGCTCACTCTATTGGACACTGGCTCGACCCAAGCAACGAGATTTACTGCTGCTCGTCAGATTGGGGATATTGCCAAATCACATCCTCAAGACTTAAACTCTCTTCTAAGGAAGGTTTGTCAATACCTCCGTAGTAAAAATTGGGATACAAGGGTTGCTGCTGCTCATGCCATTGGGGCTATTGCTGAGAATGTTAAGCAAACATCTTTGTCTGAACTATTTGCTAGTGTTCAAACAAAAATGAATGTAGCTGGGATTTCCGGTGTTGTTGAAGATGTGATGGCATGGCCTAACTTTCACATATCAGGCGTTACATTTAGAAGCTTTGAGATTAACAAGGTATTGGAGTTTGGTGCTTTATTGGCATCTGGGGGACAGGAGTACGATATTGCAAGCGATAATACCAAGAACCCTAGGGAGCGATTGGCTCGACAGAAGCAAAATCTCCGACGCCGTTTGGGTTTGGATGTTTGTGAGCAGTTCATGGATGTCAATGATATGATAAGAGATGAAGACCTTATTGTTCACAAATTTAACTCCCATGCAAATGGAGTAGATAATAGGTTCTACAAACCACAGCCCGTACATAATATACAGCAACTAGTTGCAAATTACGTTCCTAATTTTAAATCCAAAAGGCCCAGTGCTAGGGAATTGAATCTCTTGAAACGAAAAGCAAAAATTAACTCGAAAGACCAAACGAAAAATTGGTCTGAGGATGGAGATACCGACTTATCATCTTCTCCGAATGTGATGACACCAAAGGGGTTTTGTCCGGATTCATCAACTTTTAGTAAGGTGCCCGTGGATGACATTCTTGATGAAGACAGCTTTGAGCATGATGGAGATGATCGGTGGCCTTTCCGTAGTTTTGTTGAACAACTCATCCTCGACATGTTTGATCCCGTTTGGGAGGTTCGCCATGGTAGTGTCATGGCTTTACGAGAGATTTTAACTCACCAAGGTGCTTCTGCTGGAGTATTCACGCCTGACCTGAGTTCTGATAGTGCATCTTTTGTTGATCTAAAAGATAAGTGTAACCTAAATATATTGAAGAGGGAAAGAGAAATTGATTTGAATATGCAAGTTTCAGCTGACAAGTGTGAGCCAAACCTAAAAAGGCCAAAGTATGAAGATGCATCGTTTCAATTGATGCATACAATTGGCAAGGATGCCAACTCTAGTATTCCCATAAAAGTTGAAGATGGTGGAGGTTATTTACCTACTGGGCATGTTAACGGTCAACTGTGTGATAGTTCTGTTAAGGTGGAGCCTGAATCTCATGTTGACGGTGCATTGTCGTTTTGTAAAGAAGCTGTTGATATGGCAGAGCGAAAGAGTCACGACGAAGATAAGATTTTCAATGGGATGGCAGATATGCTGACTAGTATTCCTGAAAATTGCGAGCTTATGAATTTGGTTAACCTGGCTAGGCATTGTTGGCTTAGAAATTCTGAATTTCTTCAAGATTGTGCCATTCGTTTCTTGTGTGTACTGTCGCTTGATCGTTTTGGAGATTATGTATCTGATCAGGTGGTTGCCCCAGTACGGGAGACTTGTGCACAAGCATTAGGTGTTGTGCTTAAGTATATGCATCCCTCGTTAGTTCATGAAACATTGAAGATCTTGCTGCAAATGCAGGGTAGACCAGAATGGGAAATTCGTCACGGGTGCCTTTTGGGGATCAAGTATTTGGTCGCTGTCCGGCAGGAGATGCTTCCGGATTTGCTTGGCTGTGTTCTCCCGGCTTGTAAATCTGGGTTGGAAGACCCTGACGACGATGTCCGTGCAGTTGCTGCAGATGCTTTAATACCCACTGCACCTGCTCTAGTTTCTCTTAAGGGTCAGGCATTGCAGTCCATTATCATGCTTCTTTGGGATATTTTACTTGATTTAGATGATCTAAGTCCATCTACTAGCAGTGTAATGAACCTGTTGGCGGAAATCTATTCTCAGGAAGAGATGATCCCGAAGATGTTTGGGGCATTGACATTGAAAGAGAAACCAGAATTTGATTTAAATGAAGTAGTTTGTCTTGATGATGTTGGAGAAGGAATAAATACACAAGAAAATCCTTACATGTTATCAACTATGGCACCACGATTATGGCCATTTATGCGGCATAGTATCACTTCAGTCCGTTATTCAGCCATTCGCACACTGGAACGGCTGCTTGAAGCTGGATACAAGAAAAATGCCTCAGTGCCCTCTACTAGTTCTTTTTGGCCTTCTTTTATTTTGGGTGATACACTGAGGATTGTTTTTCAGAATTTACTGTTGGAATCAAATGAGGAAATCTTGCGATGTTCAGAAAGTGTTTGGAGGCTCCTTGTTCAGTGCCCTGTTGAAGACTTGGAAACTGCTGCGGGGTCATATATATCTTCTTGGATAGAGCTTGCAACTACTCCGTATGGTTCATCTCTAGATGCTTCCAAAATGTTTTGGCCAGTGGCCCTTCCTCGAAAAAGTCACTTTAGAGCAGCAGCTAAGATGAGGGCTGTGAAGCTTGAGAATGACTCTTGTAGGAGCATTGGTCTGGATTCTGCTAGCGGAACAAATACACTGGAAAGAAATGGAGATGTCTCCACTAATGCTGTGAAGATAATTGTTGGTGCTGACCTGGAAATGTCGGTAACTCATACCCGAGTGATTACTGCAGAAGCAATTGGAATATTTGCTTCTAAGTTGCATGAGGGATCTTTACATTATATAAATGATCCGATATGGAAGGCCCTTGCCTCTATCTCTGGTGTACAGCGGCAGGTGGCTTCCATGGTTCTTATTTCTTGGTTCAAAGAGATAAAAAGCAGGGACCACTCGGGAGCTCATGGAGTTACGCCTGGTTCCTCTGATGATTTTCGAAGATGGTTATTGGATTTGTTAGCATGCACTGACCCTGCATTCCCTACAAAAGATTCACTTCTTCCTTATGCTGAGCTTTCAAGAACATACGCTAAGATGCGTAATGAGGCGAGTCAACTTTTACATGCAGTGGAGTCATCTGGCATGTTTAAAAACATGACATCAACTACCAACATTGATTTGGGAAGATTGGGTGCAGATGATGCAATTAATTTTGCTTCAAAACTATCGCCTCTCAGGATTGATGCTACTGGTAAAGAATCTAAGGAAAGGCAAATTATTGAGGATTTAGACTCATTAAAACAAAGACTTTTGACAACTTCAGGCTATTTAAAATGTGTTCAGAGCAATTTGCATGTTACCGTCTCTGCCTTAGTTGCTGGTGCAGTTGTCTGGATGTCAGAGCTTCCTTCACGTCTTAATCCAATCATTTTGCCTTTAATGGCTTCTATAAGAAGAGAGCAGGAGGAAATACTGCAACAAAAGGCTGCTGAGGCACTTGCAGAGCTTATTTATCACTGTGTTCCACGCAGGCCTGGTCCCAATGATAAGTTAATTAAGAATCTTTGTAGTTTAACATGCATGGATCCTTGCGAGACACCTCAAGCTGGAGTCGTAAGTTCCATGGATGTTATTGAAGATCAAGATCTTCTTTCCTTTGGGTGCCGTACAGGTAATCAGAAAGCAAAGGTTCATATGCCAGCTGCCGGTGAAGATCGGTCAAGAGTTGAGGGCTTTATTAGCAGAAGAGGGTCTGAACTTGCATTGATACAATTGTGTAAGAGGTTTGGTGCTTCATTGTTTGATAAGCTTCCTAAGCTGTGGGATTGCTTAACTGAAGTTCTTAGGCCTGCTAATCTGGAGGGGATGACTCCTGAAGACAAAAGGCAAATAATGATAGCTATTGAGTCCATTAAAGATCCTCAAATTTTGATAAACAATATCCAGGTGGTACGTTCTATTACTCCCATGCTGGATGATACATTGAAACTAAAACTGATCACACTTCTCCCATGGATTTTCAAATGTGTTTGCCATTCCCATGTTGCTGTTAGATTAGCTGCTTCCAGATGTATTACTTCGATGGCCAAGTCAATGACGGTAATTGTCATGGGATTGGTACTTGAGAATGCCATTCCTATGTTAGGCGATATGGCATCTATTCATGCCAGACAAGGTGCTGCCATGCTTGTTGCTCTGCTTGTTCAGGGACTTGGTGTGGAGCTGGTCCCTTATGCCCCTTTGTTAGTTGTCCCTCTTCTGAGATGTATGAGCGACTGTGATCATTCCGTAAGACAGAGTGTGACACACAGTTTTGCTGCACTAGTGCCTCTTCTTCCATTGGCACGAGGCGTTCTCCCACCCAGTGGACTGAGTGAAGGATTATCTAGGAATACAGAAGATGCGCAATTTTTAGAACAACTCCTTGACAATTCCCATATTGATGATTACAAGCTCTGCACTGAATTAAAAGTGACACTAAGGAGGTATCAGCAGGAAGGAATAAATTGGTTGGCCTTTTTAAGACGTTTCAAGCTTCATGGAATTTTATGTGATGATATGGGGCTTGGTAAAACCCTTCAGGCCTCAGCAATTGTGGCATCTGACATAGCAGAGCAGAGGGCTTTAAGTAGTAGTGTGGTTCCTTTACCATCCCTCATTATTTGCCCATCGACGCTCGTTGGACACTGGGCCTATGAGATAGAGAAGTATATTGATGCTTCTGTAATAACCACACTCCAATATGTTGGTTCTGCTCAAGAGCGCATTTCTCTTCGTGGTCATTTTGCTAAGCATAATGTCATTATAACATCATATGACGTGGTCCGTAAAGATATTGATAGTCTTGGACAGCTTCTCTGGAATTATTGTATTTTAGATGAAGGACATATCATCAAGAACTCAAAGTCCAAAATTACGAGTGCGGTTAAACAGTTGAAAGCACAGCACCGTCTTATTCTGAGTGGAACCCCAATTCAGAATAATGTCTTGGATTTGTGGTCCCTTTTTGACTTTCTAATGCCAGGGTTTCTTGGGACAGAGAGACAATTTCAAGCTACATATGGAAAACCTCTAGTGGCAGCTAGAGATTTCAAATGTTCTGCCAAGGATGCTGAAGCAGGGGTATTAGCTATGGAAGCATTACACAAGCAGGTCATGCCTTTCCTTCTCCGTCGAACAAAAAGTGAAGTCTTGTCTGATTTACCAGAGAAAATTATTCAGGACAGGCATTGTGACCTGAGTTGCGTCCAAATAAAACTGTACGAACAATTTTCTGGTTCACATGTTAGGGAAGAAATATCAAGTATGGTCAAATTGGATGAGTCAGCAGAAAGAGAAGCAAATCGTGCTTCACCCAAAGCATCCTCTCATGTTTTCCAGGCACTTCAGTACCTGCTAAAACTTTGTAGTCATCCATTACTGGTTGTGGGTGAAAAGATTCCAGATTCACTCTCAAATGTTTTGTCTGAGATGTTCCCTCACTGTAATGACATAATTTCAGAACTTCATAAACTCCACCACTCTCCCAAATTAGTTGCTCTTCAAGAAATTCTGGAAGAGTGTGGTATAGGAGGAGGTATGGATGGTTCGGTTAATGAGGGTAGTAATATTAGTGTTGGACAACATAGAGTATTGATATTTGCTCAACATAAGGCTTTGTTGGATATAATAGAGAGAGACTTGTTTCACACCCATATGAGAAATGTGACATACTTGCGGCTAGATGGATCGGTTGAAGCAGAAAAACGATTTGACATTGTGAAAGCATTCAATTCTGACCCTACCATTGATGTTTTGCTTCTTACAACACATGTTGGTGGACTGGGTTTAAACCTGACATCAGCTGACACTCTTGTTTTTATGGAGCACGACTGGAATCCAATGCGAGATCATCAGGCAATGGACAGGGCACACAGATTAGGGCAGAAAAAAGTTGTGAATGTACATCGTTTAATCATGCGTGGAACACTGGAAGAAAAAGTGATGAGCCTCCAGAGGTTCAAATTGTCTGTTGCTAATGCTGTTATAAATGCGGAGAATGCTAGTCTCAAAACAATGAATACAGACCAGTTGCTTGATCTTTTCACATCTGCACAAACCAGTAAAAAGGGTGCTTTAAAGCCGCTTTCAGGCGGTGACATTGAAGAAGATCCCAAGTTAGTTGGTACCAAGAAGGGGTTAAAAGCAATTCTAGGTGGACTGGAAGAGCTCTGGGACCAATCACAGTACACTGAAGAATACAATCTCAGCCAGTTTTTAGCAAAGCTTAACGGCTAAAATTCTGCGAAGTGTATATTTGGGTTCAGATTTTGACAGTTTTAGTTGTTTTTGTTTTTACTTTTCCTTTTATTCCTTTGTCTTCCCTTCTGTTATGCATTATTAGCAGGAGGCCGCCTTGGTATGACTAGTTAGTGCGGTGAGTGACCTCGGCCCGGCCAGGAGGGTATTTGGGAAAGGAAGTAATTGTAAATCTTTTGATATTCAATATACATCACCACCTGTTTTTATGTTTTTAAATCAATTGTACATTCACCTTCTATTTTTATGTTTCAGGGATTATTGATAAGGAAAAAAAAAAAAAAAAAAAAAAAAAAAAAAAGT

>transcript_38 full_length_coverage=2;length=6757

GGCATCGTCGTCTGGTCACCACACCGCGGAGCATCAGGTTTTAATGTAAAGTAAGGTTTGGGGGTTGATGGTAAAAAGTGAAATTAGAATTACAAGGTGAGTGCTCTTACCCTGAAGAAGAAAAGAAAATGAAAGCCGGAAGTGCGGCAAAGTTGATTGTCGACGCTCTGCTGCAGCGGTTCCTTCCCCTTGCCAGGCGTCGGATTGAAACAGCTCAAGCACAGGATGGACAGTACCTTCGGCCATCAGATCCAGCCTATGAGCAAGTATTGGATTCTTTAGCTATGGTAGCGCGGCATACACCTGTGCCTCTCCTAGAAGCTCTTCTCAGATGGAGAGAAAGTGAATCCCCAAAGGGTGCACATGATGCTTCTACATTCCAGAGAAAGCTTGCAGTGGAGTGCATTTTTTGTTCTGCATGTATTCGCTTTGTGGAGTGTTGTCCCCAGGAGGGAATTACAGAGAAGCTTTGGTCTGGACTTGAAAACTTCGTCTTTGATTGGCTCATAAATGCTGATAGGGTTGTTAGCCAGATAGAATATCCCTCACTGGTTGATTTGCGGGGGCTGCTCCTGGACCTAGTTGCACAACTACTAGGTGCTTTATCTCGGATAAGATTTAGTTCTGTTACCGAGCGCTTTTTCATGGAACTCAATACCCGTCGAATTGACACTAGTGTTGCCCGGAGTGAAACACTTAGTATCATTAATGGGATGCGTTACTTGAAACTTGGGGTTAAGACCGAGGGTGCACTAAATGCATCAGCCTCCTTTGTGGCAAAAGCAAACCCTCTTAACCGTGCTCCGCATAAACGAAAAAGTGAACTTCACCATGCACTCTGTAATATGCTATCAAACATCCTAGCACCACTTGCAGATGGTGGGAAAAGTCAGTGGCCTCCTTCAGGTGTTGAACCTGCACTCACTCTGTGGTACGATGCAGTTACACGAATTAGAGGGCAGCTTATGCATTGGATGGACAAACAGAGCAAACATATAGCTGTTGGTTATCCTCTGGTGACACTTCTTCTCTGTCTTGGTGATCATCATACCTTCCACAGTAGCTTTAGCACTCACAAGGAACAACTCTACAAGCTTCTTAGAGACAAGAACCATCGTTTCATGGCCCTGGATTGTCTTCACCGAGTTTTGAGGTTTTTTTTGAGTGTTCACGCACATTCTGAGCCCCAAAATCGTGTTTGGGATTGTCTTGATAGCGTGAATCAGCAGCTGATAACCATTTTGAGGAAAGGAATACTTACTCAAGATGTCCAACATGATAAACTTGTTGAGTTTTGTGTGACTATAGCAGAACATAATCTCGACTTTGCCATGAATCATATGATATTAGAATTATTGAAGCAAGATAATTCAAGTGAAGCAAAAGTTATTGGCCTCCGTGCTTTGCTTGCTATTGTCATATCCCCTTCAAGCAAGCATGTTGGTTTGGAAATTTTTCAAGGTCATGATATTGGCCACTATATTCCTAAAGTGAAGGCTGCAATTGAGTCAATTTTGAGGTCTTGCCATAGAATCTATAGTCAGGCTCTTCTAATTTCTTCAAGGACCACCATAGATGCCGTGACCAAAGAAAAGTCTCAAGGATATCTCTTTCGGTCAGTGCTGAAGTGCATACCATATCTGATAGAAGAAGTTGGCCGAAGTGATAAGATAACCGAAATAATACCTCAGCATGGCATAAGTATTGATCCCGGTGTTCGAGAAGAAGCGGTACAGGTACTGAATCGGATTGTAAGGTTTCTTCCCCATCGGCGCTTTGCAGTTATGAGAGGGATGGCCAACTTCATTCTACGGCTTCCTGATGAGTTCCCTCTTCTCATTCAGACGTCATTGGGACGCCTGCTAGACCTCATGCGATTTTGGAGAGCTTGTCTATCTACTGATCGTCTAGAATGTGATGCTCAAGCTGCAAAGCCCGTGGGGGTGGGAAGTGAGGGATTGAAGAAGCCTTCCATTCACCATTCAGGAGGAGCAATAGAGTTTTATGCTTCAGAGATAGATGCAGTTGGGCTTATCTTTCTTAGTTCTGTTGATAGTCAAATTCGGCATACAGCACTGGAGTTATTGCGTTGTGTTCGTGCTTTGAGGAATGATATAAGAGACCTTTCATTACATGAGCCATCAGATTATAATTTAAAAGATGAAGCCGAACCAATATTTATAATTGATGTCCTTGAAGAAAATGGGGATGACATAGTTCAGAGTTGCTATTGGGATTCTGGGCGTCCATTTGATTTGAGACGGGAATCTGATTCAATTCCTCCTGATGTGACACTTCAGTCTATTCTATTTGAAAGCCCTGATAAGAACCGTTGGGCACGGTGTCTTAGTGAGCTTGTTAAATATGCTGCTCAGCTATGTCCAAGCTCTGTTCAGGAAGCCAAGTTGGAGGTTATGCAGCGTCTTTCCCATATCACACCTGCCGAGTTAGGTGGAAAGGCCCATCAGTCACAGGATGCAGAGAACAAACTGGATCAGTGGCTAATGTATTCAATGTTTGCATGTTCCTGTCCACCTGATAGTAGAGTTGGTGGTATAGCAGCAACCAAAGAGCTTTATCCTCTAATTTTCCCCTCACTAAAATCTGGATCTGAAGCTCACATACATGCAGCTACCATGGCTCTTGGCCACTCACATTTGGAAGTATGTGAAATTATGTTTGGCGAGCTTACGTCTTTTGTTGACGAGGTGTCCCTTGAAACAGAAGGAAAGCCAAAGTGGAAGAGTCAAAAGGCTCGCCGTGAAGAACTCCGTATCCACATTGCAAACATATATCGCAATGTTGCTGAGAATATCTGGCCCGGCATGCTTAGCCGCAAACCAGTTTTCCGTCTTCATTATCTGAAGTTCATTGAGGAAACAACCAGACATATTCTAACAGCGCCTGCTGAGAATTTTCAGGAAATGCAACCCCTTCGTTTTTCTCTTGCTTCTGTTCTGAGATCATTAGCCCCTGAATTTGTTGAATCAAAGTCTGAAAAGTTCGATCTTAGAACTAGAAAGCGACTGTTTGATCTTCTTCTCTCCTGGTGTGATGACACAGGAAGTACATGGGGTCAAGATGGTGTTAGCGACTACAGACGTGAAGTCGAACGTTACAAGTCGTCTCAACACACTCGATCAAAAGATTCCGTTGACAAACTTTCATTTGATAAAGAAGTGAGTGAACAAGTTGAGGCAATCCAGTGGGCCTCAATGAATGCTATGGCTTCATTGTTATATGGGCCTTGCTTTGATGACAATGCAAGAAAAATGAGTGGCCGTGTAATATCTTGGATCAACAGTTTATTCATTGAGCCTGCACCTAGGGCTCCATTTGGTTATTCGCCAGTGGATCCAAGAACTCCATCTTATTCTAAATACACAGGGGAAGGCGGGCGTGGAGGTGCTGGGCGTGACAGACACAGGGGTGGCCATCTTCGCGTTGCCTTGGCAAAAATGGCTTTAAAGAATCTTCTTCTTACGAATTTGGACCTGTTTCCTGCTTGCATTGATCAGTGCTACTACTCTGATGCTGCCATAGCTGATGGATACTTCAGTGTACTGGCTGAAGTCTACATGCGCCAAGAGATACCAAAGTGTGAAGTTCAGAGACTGTTGAGCCTGATCCTCTATAAGGTAGTGGACCCATCTAGACAAATCCGTGATGATGCCCTCCAGATGCTTGAAACACTCTCTGTCCGTGAGTGGGCTGAGGATGGGATCGAAGGCTCAGGAAGCTATCGAGCTGCTGTTGTGGGTAACCTCCCCGATTCCTACCAACAGTTCCAGTACAAACTCTCCTGCAAGCTTGCCAAAGATCACCCGGAGCTTAGTCAGCTCCTTTGTGAAGAGATCATGCAGAGGCAACTTGACGCCGTTGATATAATTGCACAACATCAGGTCTTAACGTGCATGGCTCCATGGATTGAGAATCTCAATTTCTGGAAACTCAAAGATTCAGGTTGGAGTGAAAGATTGTTAAAAAGCCTTTACTATGTAACATGGCGGCATGGCGATCAGTTTCCTGATGAAATTGAGAAACTGTGGAGCACAATTGCTAGCAAGCCCAGGAATATAAGTCCGGTCCTAGACTTCTTGATCACCAAAGGAATTGAAGATTGCGATTCAAATGCATCAGCTGAAATTAGCGGTGCATTTGCTACATACTTCTCAGTTGCTAAAAGGGTAAGTTTATATTTAGCAAGGATATGCCCACAGCGCACGATAGATCACTTAGTTTACCAGTTGGCTCAGCGGATGCTTGAGGATAGCATTGAACCTATTAGACCAACTGCAAGTAAAGGAGATGCGAATGGAAATTTTGTGTTGGAATTCTCTCAAGGCCCTGCACCGATCCAAATTGCATCTGTTGTTGATAGCCAGCCACACATGTCCCCATTACTCGTCCGTGGTTCTCTCGACGGTCCACTAAGGAACACCAGTGGAAGCCTAAGCTGGCGAACAGCAGGTGTTCAAGGCCGAAGTGTTTCTGGTCCAATAAGTCCCATGCCTCCTGAGATGAACATTGTTCCTGTACCCACTGGTAGATCTGGTCAGCTCCTTCCATCTTTAGTTAACATGTCTGGTCCATTAATGGGTGTTCGAAGCTCAACAGGCAGTTTACGAAGCCGCCACGTGTCCCGCGACAGTGGAGATTACTTTATTGACACTCCGAACTCTGGTGAAGAAGGGGTACATTCTGGGGTTGGTACTCATGGTATTAGCGCGAAAGAACTTCAATCAGCTTTACAAGGACATCAGCAGCACTCACTCACTCATGCAGACATAGCCTTAATTCTACTTGCAGAAATTGCTTACGAGAACGATGAAGACTTTCGTGAGCACTTGCCTTTACTCTTCCATGTCACGTTTGTCTCTATGGATAGTTCAGAAGATATTGTGCTAGAACACTGCCAGCATTTACTTGTGAATTTACTATACTCACTAGCAGGCCGACACCTGGAGCTTTATGAAGTTGAAAATTGTGAAGGAGAAAACAAGCAACAAGTCGTCAGCTTAATCAAATACGTTCAATCAAAACGAGGAAGCATGATGTGGGAAAACGAAGACCCAACAGTTGTACGACCCGAACTTCCAAGTGCAGCACTCTTATCCGCCTTAGTTCAAAGCATGGTAGACGCTATATTCTTCCAAGGAGATCTCCGTGAAACTTGGGGTGCCGAGGCACTTAAATGGGCAATGGAATGCACATCGCGACATCTCAGCTGCCGATCACACCAGATTTACCGCGCCTTACGACCTAGTGTAACGAGTGATACATGTGTATTACTCCTCCGTTGCCTCCACCGTTGTTTAGGAAATCCAGTACCTCCCGTTTTGGGTTGCATTATGGAAATTCTGTTAACTTTACAAGTAATGGTGGAGAATATGGAACCCGAAAAGGTTATACTCTATCCACAGCTGTTTTGGGGATGTGTAGCCATGATGCATACAGATTTTGTCCATGTGTACTGTCAAGTGCTCGAGCTTTTTTCTCGTGTGATTGATCGGTTATCTTTCCGTGAAAGAACGACGGAGAATGTTCTTCTTTCGAGCATGCCGAGGGATGAACTTGATACGAGTGTTTGTGATCCAGATTTTCAACGACTAGAGTCTAGGCATTTTTGTGAGCCAAGTGGGAAAGTACCGGCTTTTGAAGGTGTTCAACCTCTCGTTCTTAAAGGACTCATGTCAGCCGTTAGTCATGGTGTTTCAATTGAAGTTTTATCACGAATCACTGTTCACTCATGCGACTCAATTTTTGGAGATGGAGAGACGCGACTCTTAATGCACATCACCGGTTTATTACCATGGCTATGTTTACAGCTCAGCAAGGACTCGATTATCGGACCGACTTCACCGCTCCAACAACAATCACAAAAAGCTTGCTCTGTCACGGCGAATATTGCTATCTGGTGTCGTGCAAAATCGCTGGACGAGCTCGCGACAGTATTTTTATCTTACTCGCGTGGCGAGATAAAAAGTATTTTTAACCTTCTCGGTTGCGTCTCACCATTGTTGTGCAACGAGTGGTTCCCAAAACATTCTGCACTCGCCTTCGGCCACTTGTTAAGGTTGTTGGAAAGAGGTCCGGTTGAGTATCAGCGCGTGATACTTTTGATACTTAAAGGGTTACTCCAACACACTCCGATGGACGCCGCACAGAGTCCGCACATGTATGCAATTGTTTCTCAATTAGTGGAGAGTACATTGTGTTGGGAAGCGTTGAGTGTTTTGGAAGCGCTATTGCAGAGTTGTAGCTCGTTGACGGGAGGGTCGTCACACGCCCATGAGAATGGGTTTAGTGGGGATGATGAGAAGATGCTTGCGCCTCAGACATCATTTAAGGCGAGGAGCGGTCCGTTACAGTTTGCAATGGGTGGGTCGGGTTTTGGAGTACCAATGGGTGGTGGTGGTGGTCAAGTGGGGAGTACAGAGTCTGCGAGAGAAGTGGCGTTGCAGAATACGAGGTTGATACTTGGGCGGGTGCTGGATATTTGTGCGCTTGGGAGGAGGAGGGATTATAAGAGATTGGTGCCTTTTGTGACGAATATGGGATGAGAAGTGATTGTACACACGTGTATCGTTAGTTTTGAACATGATTTTTGTAAATAGGCATAACAAGGTTGTTATTGGTAATTTAGTGAAGATGGTTTTGTAATTATACATATGGGATATGTTATGATTGAAATGTAATTGTATTTTTTCTAAACAAGATTTGTATGAAAAAAAAAAAAAAAAAAAAAAAAAAAAGT

>transcript_39 full_length_coverage=2;length=6700

GGAAAGCAGTGCCTATAATTTGGATCCAAATGCTGATGGTGAAATAAGTGGCTTACCTATGCAGGTGAATGGTGCCAGTGATGGCAATGCAGATAGTTCAGGTAAAGACATTGGTGACGGAGGGGACCATGTTCTGAGCGAAGATGTTCACATTAATGATCAAAATATGGAGAGGCTTCCATTTGAGAGTGGTGCCTATAATTTGGATCCAAATGCTGATGGTGAAAATATTGGCATGCCTGTACAGGTGAATGATGTCAGTGATGAAAATGCTGATAATCTAGGTAAAGACATTGCTGACAGAGGGGAGGAACGCCATAGTTCGAGTAAAGAGGTTCTCATGAACGATCAAAATGTGGAGAGAGATGCAGTTGAAAGCATTGTCTATAGTTTGGATCCAAATTGTTCAGATCAAAATGTTGATGGTGAAAAAGGTGGCTTATCTGTGACCGAATGCGATGTTGCAAATCAGATGGAAGTTGATATGACATCTGATGTATCTTTGGATAACAGGGCACAAGAGAATTCTTCTGCTTCTAGGATGCAAGTTGATCATCTAGTCACTTCTGCACAGGATATCGGTACAAGCATTATGCAGTTGAACAATCAAGAGGCCGTGCAGGTAAATGATTCTAGACCTTCTAGCGATGAGAACGAAGATGGTTTACTTAAAGGCAACGGTGAAGGAGGGGGGGTTCAACCTGTTCTGTGCGAAGTTGTTCAAATGAATGATCAGAATGAGGAACATGCAGTTGAAAATGGTGCTTATAATTTGGAGACCATTCCCAGTCTGGCCTCCAGGGTTAAATCTACCGGAGAACGACCTGCACTTGAAACTAGAATCAGTAATGCGGAGGAACCTTCTAGTATGATAATGAAGGGGGATCCTGCCTCACAGATTGTGGAAGGATGCAGTGAGGATACATGTGCATCGATGCCTGTTCAGGTCAGCAAATGTGAAGTAGTGACTTCGTGCAGAGACACAGAGTCGTGTGATCAGTTTAAACAAATCATGCATGAGAAATCACCCATAGCTTTGGGAGGTGATAACAGTTATAAGGGGAATGCCGTTGAGTTCAGCAACACGATATTGGGGATATGTGCTAGTCCAGGTCTGAAGAAGGATACTCTTGCAGAGATGTCATATGGGCAGGGTTCTGTTGAGAAAAGAGAGGATTTGTTAGAAAGAGGAAATGAAAAGGATAATGTTCCAGTCCGCAATTCTGAAGCATCTCTGTTATCTGTAAAAGGTGGCAAACCTTCTAAAGATCAATGTGATGGAAGCAGTTATCGTCAAGTGGGAGATATATCTAGTGTAAAGGTGGTTTTTTCTTCAGCTGAATTGCTTACGGAAACACATGCAACTAAACATTGTAAAGGTGTTCTTGATCCTTTTGGAGTTCGCAGAGAGGACTCAAATAGTGAAGACCTTGTCTCATCTTCTACTGTGGCAAAATCCATGGTATATGAAGATGATGTAGTCTCCCGGCAAGGTGTTGATGGTAACTTTGACCGAGATGTTTCTGTTATCGAAAAGGAGAATTTACAGTTGCCCACGGATTCTAGTGATGTGGGTTGTGAGATCGCCAGTTCCCTTATCATTCATAATAAGGCTGGATCCTCGTCTCCAGGTGACGAAACCCTAAATGTAAATGATAAAGAGGCGTCTCTAAAAGTTGCAGAGTCGATTCTAAACAAAGGCGATATGCTCACACAGCCTGTGCCGCCTTTAGAGGACTCATCCGATTTTGGCCAAATAGGTCAGAAAGATAGTGAAGGTATATTGGAACCTGCTGATAATAATTGCGGGCTGATTCCTATCCCAAGCAGTGAAGGTAATGCTTCATGTGCCCATGATGGTTCTTTGAGTTCTGTTTGTATGTCCGAATGTGAAGATAAGGTTCGGGTGCTGGAGGGTGGAAGTGTTTATGCAGATTCTGACAAACCTAATTGTGGATCTCCCACTGTTATTAGTTGCACTGAACTTTGTAAAGAAAAAGAAAATCAGCAGGGAGTCAAGGGGCCTTTGGATCAGATTGCCCCACATTCTAATATCAATGATGGTTCTGCCAAAAAAGTGTGCTCTACTTCCCAGGATATGAAAGAAGATGATGCCTTAGACGAGAAGAGTTTTTCTTTTGAAGTAAATTCATTGGCTGACTTGCCTGGAAGAGAGTCTGGCAAGGGTTGGCAGCCATTTACCACTATTCAAGATCCCAAAGTATCCATGATTGTAGAGGGATCTCCTACGATATCTGGTTCAGGCCAAATTGATTATAAGTTGGCACAAGAAGTTCCCCATGCAAGTCCTCGAGCGTCTAATGGAGTGAATATAGTTGGTGGTTCCAGAGCTACTCCTGAGCGTAAAACAAAACGAGGGTCTGGTAAGGCAGCAGGGAAAGAAAGTGCTAAGAAAGGAAATCGTACAAAAGAATTAACTCCCATGAAACAGCAACTAGACAGAGTTGAAAAATCATGTGTTGTGTTGCCGAGCCCACCTGGGACTTGCCTGCTTGTGCAATCCAAAGAGATGCAGCACTTTGGGATTATGGAACGCAATAACAAAAAACCATCTAATGCTTTTACTGCGACATCTGGTCTGCCAGATTTAAATACTTCAGCTTCTCTACCGGCAGTGTTTCAACAGCCTTTTACAGACATGCAACAAGTGCAACTGCGTGCTCAGATCTTTGTTTATGGATCTTTGATCCAAGGAGCAACACCTGACGAGGCATGTATGGCATCTGCTTTTGGGGGACTGGATGGTGGAAGGAACATTTGGGAGAACGCATGGCGCACATCTATAGAAAGGCTTCACGGTCAAAAATTTCATCCCAGTACCCCTGAAACCCCTTTGCAGCAACGTTCAGGTGTTAGAGCTTCAGATCAAGCAGGTAAACAGGGCACACTTCAAAATAAAGTCATCTCTTCACCTGTTGGTCGAGCTAGCAGCAAGTGTACTCCGCCACCAGTTGCAAATCCTATGATACCTCTTTCATCACCACTTTGGAGTATTTCCACCCCATCATTTGATGGTATGCAATCTAGTGGCATGCCAAGAGGTTCAGTTATGGAACATAACCATGCAATTTCACCATTGCATCCTTTTAAGATCACACCTGTAAGGAATTTTGTTGGGCATAATGCTTCTTGGTCATCTCAGGCCCCTTCTCCTTGTTCTCGGGTTGCTTCTCCACAAACATCGGCCAGTTCCCGTTTTTCTGCATTGCCTATCACAGAAACAGTTAAATTAACTCCTGTTAGGGAATCATCCGTACCACTTGCCTCTGCTTTAAAGCTCGTATCTCCTAGTACTATTGTTCATAGTGGGGATCCTACTACTGTTTTTCCAGGGACTGCTTCCTTGCTTGATGTGAAAAAGGCAACATCATCACCTAGCTCAAATTCTGCAGATCCAAAGCCAAGGAAGAGAAAAAAAGTCCCAGTTCCTGAAAATCCTGGTCTTATCTCTTTGGCACCTCAAACTCGAATAGAACCAGTTTCTTTGACACCTCAACCTCGAACAGAACCAGTCTCTTTGACACCTCAACCTCGAACAGAACCAGTCTCTTTAACAGCTCGAACCCGTACAGAACCAGTCTCTTTGACCCCTCGAACTCGAAGTGAACCAGTTTTTTTGACCCCTCGAACTCGAGTAGAACCAGTCTCTTTGACACCTTGGACACGAACAGAACCAGCTTCTTTGCTGCCTCGAGCCCGGACAGAATCAGTTTCTACTCCAGATGCCTGCAATAGTCATTTATTTACAACTGTTACTGTCACAACTCCTAGTTGCACAGTGTCTCAAAGTAAATCTGGGAAATTTTGTACAGCAGCATCATCTCCAGCATCTTCCACTGATTGTCAGAAAATTGGGGATGTGGGTGTAGATCAGAGGGTTATATTATCAGAAGAATCCATTAATAATGTTAAAGAAGCTCAGTTGCAGGCGGAAAATGCTGCTGATCTTGCTGCTGCTGCAGTTAAGCACTGCCAAGGTATATGGAGTCAGCTGGATAAGCAGAAGATTTCTGGATTGATACCGGAGGATGAAGCTAAACTTTCTTATGCAGCTGTTGCAATTGCTGCCGCTGCTTCTGTTGCAAAGGCAGCAGCAGCTGTTGCTAAGGTTTCATTAGATGCTGCATTGCAGGAACAACTGATGGCTGAAGAAGTACTAGCTTCGAGAGGAAATGAAAATTCTTGTCAAAGTAACACGGTTCCTCTTTCTGATGGCGTGAATATTCTGGGGAAGGCTACATCTGCATCCATCTTAAAGGGTGAAGGTGCAACCAGCTGTTCCAGTTCAGTTATTGTTGCTGCCAGGGAGGCTGTTAGGAGGAAGGTTGAAGCTGCTTCGGCTGCTTCAAAGCGAGCTGAAAATATGGATGCTATAGTAAAAGCTGCAGAGCTGGCAGCGGAAGCGGCAACTCAGGCTGGAAAAATTGTTGCTATGAAAGATCCCTTGACTTTGACTGAATTAGTAAAAGCCGGTCCAGAGGGTTACTGGAAAATACCCCAAGTATCTTCTGAGCTGGTCCTGACGTCAAATAACACGAATAAAGGGCAATCTAACTTAGATAGCGTTGAAGATGATCGCGATGGTTCTGCTCAATGTTTGAAGGGTGAAACATTAAATGAAAAAGGAACACAGACCACATATGGGAAGCCATCGACTCAAAGTGATCTGTCCATGGAATCCATGGAGGGCCACACAAAATTGGTTGATGGCATTTCGAGTTCTGTAACAAACAGGGAAAAGGTTTTCAGAGCACAAAGGGGGCATAAAGTTTCTGACATGGCGAAAACCGTTGGTGTTGTTCCAGAATCTGAGATTGGATCAAAATCTACTTCCATCACTGTTCAAAATGAGTATGAAAAAGCGGGGGAGATTTCAAAAGAGAGCAACATCAAGGAGGGTTCACTTGTAGAGGTTTTTAAAGATGGGGATGGTATTAAAGCGGCGTGGTTCTCAGCCAAGGTGTTGAGCTTGAAGAATGGAGGGGCGTATGTATGTTATCCTGAAATACCATCAGAAGGCCAGGTAAAGGAGTGGGTGGCACTGAAAGGCGAAGGAGATCAGGCACCCAGAATACGAATTGCCCATCCTATGACCAGCATACCATTTGAGGGAACAAGGAAGAGACGCAGAGAAGCTGTGGGGGATTATGCTTGGTCTGTTGGAGATAGAGTTGATGCCAAGATCCGAGATTGTTGGTGGGAAGGAGTCATCACTGGAAAGAATGACGAAATCACACTCACTGTTCATTTTCCAGCCAAGGAGAAACATCTTCTGTTAAACCTTGGCAGCTTCGGCCTTCTCTCATATGGAAGGATGGGGAATGGATCGAATGGTCCGGTTCAAGAGAAACCAAACGCTCTTCTCATGAGGGTATCGACGTGCCACAAGAAAAGCGGCTCAAGTTAGGCAGTCCTCTGGTAGATCCAAAAGGGAAGGACAAGATACCTGAACCTAGTATCTCTGAAGAGTTAAAACTAACAGGCTTATCATCAAAAGATAAAATATTCAATGTTGGCAAGAACGCTAGAGATGAAAACCAGCCTGACGGAGGAGGTAGAACAATGCGAATTGGTTTGCAAAAAGAAGGATCAAAAGTGATATTTGGTATTCCCAAGCCTGGAAAGAAGAGGAAATTCATGGATGTTAGCAAACATTACGTAGCAGAGAGGAGCACTAAACCTAAGCCGCCAGAAGAAGAAAATGATTCGATCAAGGTTAGTAAATATTTAATGCCACAAGGATCCAGCTCGCGTGGATGGAAAAATAATTCCAAAGTTGATTTGCGGGAGAAACGAGTTGCTGATTCAAAACCGAGTCGGGTTCTTAAATCCGGGAAACCACAAACCATTTCGACTTCATCCAGATCTTTACCTCAGAAGCAGCCTAATTTTACAGAGATCAAGGATTCTGGTCGGCATGGTGAGACTACATCAGGAAAGTCGAATGCGACCCCGTTTCGATCTTTTTCTAGTACCGAGGGTGCACCAGCTGGTCCGATGGTGTTTACTTCTACAGGTCTTATATCAGATAACCCTCCTTCCTCATCTAAGAAAGAAACCATGTCAAATTCAAAACGCCTGAATAAAGGAAGACTTGCACCTCCAAGTGGAAGGTCGGCTAGAACTGAACAGGAGAAAGTACATAATGGCAATTCTGTAAAATCGACTCCGGAAGTTGTTGAACCTCGTAGATCTTGTCGGAAAATTCAACCGACATCACGGTTATTAGAAGGGCTTCAGAGTTCATTGATCATCTCAAAAATACCTGCTGTGTCTCATGACAAAAGTCATAAAAACCAAAGCAGGAGTGGTTCTTCTAGAGGGAATAATAATCATGGTTGAGAGGAAATGACCGGGGCAATTTAAGGTTAATGACAGACAGAGGCACAACATCTCCCGTGTTGTTTTTGTGTATACTTGTGATAGAGAAAACCAGGAACGAAAATTCTCGCTTTCCTAAGGGTTTTAGTTGTAAAATGTGTTGAGTAGATGTTTATATATTTAATTTAGTTTTTGGTCTCGTCACAATTTATGATGATTTTAGTATTTCGAATGGGTTGGATGTATCTGGTAACTCTCATCTGGTAACTCATTGTAATTTGAGCTTATAATTGCAGGAAAATTTTAATCCATCCTTATGTTTTAGTGAAAAAAAAAAAAAAAAAAAAAAAAAAAAAGT

>transcript_40 full_length_coverage=10;length=6681

GGGGAACACATTATCTATTTTGCTGTGAAAGATTATCTAGAGAAGTAGACGGGAGAAAGTATAGCTAGAAAGAGAACATTCATGTTATATTGATAACTGATGAGGGACTCATTCTCCGGTTAGGGTTTGGAATCCTGTGTAGGAACTTGTGAACACTACTGTAACCCTGGAGAGCGAAAGCTCTCGATCCAGATACTGTTGTTGCACCGATTGGCTAGGTTATTTGTCTGGTATGGAGATCGATTCCCCTTCTCACTCCGATTTTCTATCGCCTCGTGAGCGAATCGTACAGAGGCTTGTTCAACAAGGAGTTCCCAAAGAGCTGCTTGATCGCCTCCAGCGTGGTTTGATTGCTTTTGTCAAACAGAACAGGCTTCGAATACCAGAGCTCGTATCTGCCATTTTACCAACTGATGAGGAGGTAGCGGAGGCTTTATCAAAAGCTAAAGCAGTGAGCAAAGCCGCCAAAGCTTTTGGTAGTCTAAGCCTGAAAAAAAGGTTCCGTGAAAGCATGGTTTGGTTACAGTGGTTGATGTTTGAGGGTGACCCAGGGGTTGCCCTGGATGCCCTTGCTAAAATGAGTGTTGGTCAGCGTGGTGTTTGTGGGGCTGTTTGGGGAAACAATGATATAGCGTACCGCTGCCGAACATGTGAGTACGACCCAACATGTGCAATCTGTGTTCCTTGCTTCAAGAATGGGAACCACAAGGACCATGATTATTCTATTATTTACACAGGTGGTGGTTGTTGTGATTGCGGGGATGTAACAGCATGGAAACGCGATGGCTTTTGTTCAAAGCATAAAGGTGCAGAGCAGATACAACCCCTGCCAGAGGAGTTTGCAAACTCCGTGGGGCCTGTCCTCGATTCCCTTTTGGTTTGTTGGAAAAACAAGCTATTGTCTGCAGAAGCTGTATATCTGGAAAACCCTAGAACAAGTGATCATGTTGCAGAACTCAGAAAGATTGCAAATGAACTGACATTTGTTGTGGTTGAGATGCTTCTAGAGTTATGCAAGCACAGTGAGAGTTTGCTCAGTTTTGTTGCTAAGAAGGTTATTTCTTTAAGTGGTTTATTGGATATTCTGGTGAGGGCAGAGAGGTTTTTAGGTGAAGTTGTGAGGAAGCTCCATGAATTGCTCCTAAAGCTTCTGGCAGAACCTGTGTTTAAGTATGAGTTTGCTAAATCATTCCTGAATTATTATCCGGTTGTTATAGAAGAAGCCATAAGAAAGAGTAGTGAAACTGTTTATAAGAAGTATCCACTCCTTTCCACATTCTCTGTGCAAATATTCACAGTGCCAACTCTAACCCCCCGTCTTGTCAGGGAGGTGAATCTATTGGCTATGCTCCTGGGGTGTCTTGGAGATATTTTCATTTCTTGTGCCGGAGAAGATGGTCGTTTACAGGTTACTAAGTGGAGCAATCTGTTTGAACCCACTGTCCGTGTGATTGAAGATATTCGATTTGTTATGAGTCATGTTCAAGTACCCAAATATGTAACTCAAGAACAGCGAGATATCTCAAGAACTTGGATAAGACTCCTGGCTTTTGTGCAAGGGATGAACCCTCAAAAAAGAGAGACAGGCCTCCATATAGAAGAAGAAAATGAGAATTTTCATTTTCCCTTTGCTTTGGGACACTCAATTACAAACATTAATGCTCTCTTGGTGACTGGAGCATGTTCTGTAGCTAATGCTGAAGAGACACAAGACGGGACTCTCTTGAACACAAAAATTGATGATTTGATGATGATGATGATGGCATACGACATGCAAAAGTTGGACGGATTTCCCAGGAATCCTCTGTGTGTAGTGCGGCAGGGAGGAATAATTCATTGGCTTGTGCCTCGAAAGTTTCTGAAGTAGAGTCACATAATTTAGTTCATGGTGTAATTCCTTCCTCTGTCACATGTTTAATATATGAGTGCTTGAGGGCAATTGAGAATTGTTTGGGTTTGGATAATACATCAGGGGCTCTTCCAACCACTTCTAATATCTCTGGAAGTAATTTTATGACATTCAAGAAAACACTATCTAAGATAAAAGAGGGGAAATATATCTTTAGTAAACATTCCAGTTCAAGTGAAGTTCTTGAAAGGCCACTTTCTTCACCTGTTTATAACCATTCAAGTTCTGACTTGATATTTAGAAGACAGGATAGTAAACCAATGATCACTTGTGAAGGCATGGATAATATGCATGATACTGCAGTCCTTGATGATAGTACCATTGAAGGAGAATGTGCCACAGAATTAGAAGCCTTACGTGTGTTGAGTTTGTCAGATTGGCCAGATATAATTTATGATGTTAGTTCGCAAGATATTTCTCTTCACATTCCCTTGCATCGATTACTTTCCCTGCTTTTACAGAAAGCATTGAGAAGATTTTATGATGAATCTGCAATGCCAAATGTGGTGAATGCAAGTTCTACTAATCCATGGTCGGCAATTTATTCTGACTTCTTTGGACATGTCCTAGGAGGCTGCCACCCGTATGGGTTTTCGGCTTTTGTTATGGAGCATCCTTTACGGATTAGGGCATTTTGTGCTGAGGTTCATGCTGGAATGTGGCGGAAGAATGGGGATGTTCCTCTATATTCTTGTGAGTGGTACAGTTCAGTACGCGGGGCTGAGCAGGGTTTAGAGCTTGATCTCTTCCTACTGCAGTGCTGTGCTGCGTTAGCTCCAGCAGACCTTTATGTTAATAGAATTATAGAACGCTTTGGGTTATCCAGCTACCTTACTCTGAATCTTGAACGGCCTAGTGAGTATGAACCAGTTTTGGTCGAGGAAATGCTCACTCTTATCATCCAAATAGTTAAAGAACGAAGGTTTTGTGGGCTAACAACAGCTGAAAGTTTAAAAAGGGAGTTGATTTATAGGTTAGCTGTTGGAGATGCGACTCACAGCCAATTGATGAAATCTCTCCCTCGTGACCTTTCCAAATTTGAGCATCTTCAGGAAATTTTGGATACTGTTGCAGTATATTCCAATCCATCGGGCTTAAATCAGGGTATGTACTCACTGCGATGGACGTTTTGGAAAGAACTGGATTTATATCACCCTCGTTGGAACCCAAAGGAGTTACAAGGTGCAGAAGAAAGATACATGCGTTTTTGTAGTGTTTCTGCATTGACCACTCAACTCCCTAGGTGGACTAAGATATATCATTCTCTCAACGGAGTAGCTAGAATTGCTACTTGCAAAATGGTTCTTCAAATTGTCCGTGCAGTGTTATTTTATGCTGTTTTCACTGATAAATCAACCACATCCCGTGCTCCTGATTGGGTGCTTCTCACCGCATTGCATTTGCTCTCATTAGCATTAGACATATGTTCTGTGCAAAAGGAATCTGGCACTCAGTTATGCTATGTTGGGGATTTGATTCCCATGCTAGCTTTTGCTGGTGAAGCTATTGGTGGGGGAAGAAATGACAGATGTGGTGAACAGAGCTTGTTATCTCTTCTTGTTTCATTAATGAGGATGCATAAGAAAGAAAATTTATACAATTCTATGGAAGCAGGCAATTGTGACATTTCTTCCTTGATTGAAAGTTTATTAAAAAAGTTTGCTGAGATTGACTCTGGATGCATGACCACACTTCAAAAACTTGCCCCTGAAGTGGTCAACCACTTATCACAATCCATTCTGAACAGTGATAGTATGTTGGGGTCATCTTCTGACAATGACAAACGGAAGGCAAAAGCTCGAGAGAGACAGGCTGCAATACTGGAAAAAATGAAAACCGAGCAGTCCAAATTTTTGGCAAGCATCACTCCCACTTCAGATGACGAGTTAAATGGTTCAAAACCTGTACAAGAAGTACACAGTTCTGATGTTGGAGAAGAGTCTGCACAAGAAGTTTGCTGTCTTTGCCACGACCCCAGTTCTAAAAATCCTTTATCTTTTTTAGTTCTTCTTCAGAAATCCAGGCTTGTGAGTTTTGTCAATAAAGGCCCCCCATCATGGGAACAAGTTTGCCAGTCAGGTAAAGAGCAATCAACTGCCTCAAGTAACACTTACTCAAGTGGTTCGGAAATGGTTTCAACTTCCCAATTGATGCAGTCTTCAGAAATAGTTTCATCTTCCCAATTGGTGCAGTTGGTTCAGAAAGCTGTTAATGAGTTCTCCTTTTATGCGCGATGCGGGGACGTCAATGCTTTTTTAGAATTCATTAAGACTCGCTTTTCTTCAGTGAGAAGTATTCAACTGCCTCACACAGCTGATGACACGTTGGACAAGGCAGTAAGTTCCTCTGAGATGTTGGAAGAAGATTTGTACAATTCCATCCTGAAAGAAACACATGATAGTTTGCCGCATCCAAAATTCTATTCAGACTCTCCCTTACTGGGGAAATATATAGCTGTTCTTTCAAGAGAGAGGGTAGACAATCCTTCAGCTTCTGAGAATGACCATTCACGTAATAGCAAGGGTCCATCAGAATCTATACCACAGCTTTCCACATACGATGGATTTAGCCCCTCAGACTGTGATGGGATTCATCTTTCGTCCTGTGGGCATGCTGTGCATCGGGGATGTCTTGAACGTTATTTATCTTCCCTGAAGGAGAGATATATTAGAAGAATTGCTTTTGAAGGAGGGCATATTGTGGATCCAGATCAGGGAGAGTTCCTCTGCCCTGTATGCCGCCGACTTTCGAATTCTGTCTTGCCTGCATTACCTGGACATACTCAAAAGGTCTTGGGGCAGCCAATGATTTCAACTGTTGATTTGCCACATGCTGAAGGCCCATCAACATCAAGCGAGAAAATTGATTTCCTTCACCTTCGTCATGCTCTGACTCTAATTGAAACTGCAGGAAGCATGGTTGTGAAGGGTGAAATCCTCAGAGCTTTACCCATGCGGAGAAATGGAAGAACAAGACCAAATCTTGAACCTCTCGTTGATATGCTTTCTGGAATGTATTTTCCAGGCAAAAAGGATAAGTTATCAGGATCTGCGAGGGTAAGTGATTCATTAATTATGTGGGACACTGTTAAGTACTCCCTCATATCAACAGAAATTGCTGCTCGTAGTGGAAGGACTTCTATGACTCCAAGATATGGCCTTGATTCTTTATACAAGGAGCTTAAATCTTCTGGTGGATTTATTTTATCCTTGCTGCTTAAAGTTATCCAAAGCATGCGGAGCAAAAATCCTCTTCATGTGCTTTTAAGATTTAGAGGCATTCAGCTCTTTGCAGAATCAATTTACCCTCGTGTTATTGGAGAAGAACTTATCAGCAGCACGTGCAGACAAGGAGGGTACATGTCAAGTATCTTGAAACATATAGAAACGGATATACCCTATCAAGACATTCAATTTTGGAAACGAGCTTCTGATCCTCTTCTTGCTCGCGACCCCTTTTCATCATTAATGTGGGTTCTATTTTGCCTTCCATCTCCATTCCTATCTTGCGATGAGTCATTTTTATGTCTTGTGCATCTCTTCTATCTAGTCTCGGTTACTCAGGCAATAATCACATATTGTGGAAAATTCCAATCAAGTGTTGGTGAATTAGGTTCACACGGTTGCCTGATTTCCGACATATCAAACTTCATGGGACAATCTGGATTTCCTATGGATTATTTTGTTTCCAACTATTTTACTGATTCTTGTTATAACTTAAACGACATGATTCGTAGCTTCACTTTCCCTTTCTTGAGAAGGTGTGCATTGCTCTCCAAACTATTGAGTTCTACCGCGTCAACACCTCATGGATTTGACGCATTGTGTCCTGATGACATTGATGATATGATGGATACTTGTAATGCTAATGACGCTGAGGTGGAGTTTATTGAGGTTGGGAAGCTGGAGAACATGTTTAATATTCCCCAACTGGCCGATGTTGTTAAGGACGAAATGACTCGAGGCGTAGTATTGAAATGGTTGCATCATTTTCGAAGTGGGTTTCAAGTTGCTGGCCGTGAATTTGTTTTGTATTCCACTCCAGCAGTTCCCTTTAAGTTGATGAATTTGCCTCATGTTTACCATGAGATCTTGCAGAGGCACATAAAACAACGTTGTCCTGAATGCAAACTTGTTATGGATGATCCCGCGTTATGTTTGTTGTGCGGTAGATTATGTTGTCCTACCTGGAAGTCGTGCTGCAGGGAATACAGTTGTCAAACTCATGCAACTGCATGTGGTGCTGGTGTAGGAGTATTTTTGTTAGTCAGGAAAACTACAATACTGCTTCAAAGATCTAAACGTCAGGCACTTTGGCCTTCTCCTTACTTGGATGCATTTGGGGAAGAGGATATTGAGATGTATAGGGGAAAACCACTGTATTTAAATGAGGAACGATATGCCGCGCTAAGTTACATGGTGGCTTCTCATGGTCTTGATCGGAGTACCAAAGTTCTCGGTAAAACAACAATTGGGACTTTCTTTATGATATAGAAATAGAATCTAAAGAGAGAGTTTTCCCCTCGTTATATATATTATTCATCATTTTGTTTTTTTTTCTTTTTTTTTTTCCCCCTTGGGTTGGCTGGGAAATAAGGTTGTACAGGAGTGTTTGTTTTAAAGAAGATATTATGTATAGCAGTTTTTCATTATGATGCAAATAATGCATTAAAATGCATAGTTGAATCAATCCAGCTTTTCTTCAATTTCTGAATTTTGATAGAATGAAATTAATTTTTATGAAAAAAAAAAAAAAAAAAAAAAAAAAAAAAA

>transcript_41 full_length_coverage=2;length=6686

GGGGCACTCAGACAGAGAGCGAGGCGGCACAAACCGCGAAGATATAGATAGAAAGGGTGTGAGAGAGAGAGATGGCGTTGATAGCCCGTGGAATCTTAGAATCACACAGTTGTTCCCACTCAACAATCTGCGTGCTTCGTACGGTCTATCCACGCTAATCTGCACCTCCACTCTTCTGTTAGGGTTCACATCCCACGTCAAACGATCATCGCGAATACTCTCTCTCTCTCACACTATTTAGTATCCATTCTTTGATTTAAAAAAATATACAAAATTACAATTTTATTCATTTGCATTAAAGATTTGATTTTCTTCTCGTTTTTATTTTGGTAAAAAGATTTGATATTTTTTGCTGGCCAGTGTTGCGATTAGTGTAATGGGGGACGACACTAGGGTTTTGCCAAGCGGCGACCTCCCTACAGATGATGTGAAGCCGTCGTCTTATTGGTTGGATGCCTGCGAGGATATCTCCTGCGACCTTATCGATTTTGATACCGCCGTTGTCCCAGAATCAGCAGTTGATGCTTCCTCCATTGACGCCGACGCCGATGCTGACGCCGATCCCCTCTTCTTCGGAGAAATGGATACCCTTTTCGACTCTATCAAGAACGGGTCTGGCCTCTCCCCGGTTGTTGATGATTGCTCCGTTCGACAAGGTTGTGTCGTGGGTGCTTCTCGGGTTTGCGATATTCATCAAGTTGACGACTTAATTCTCGAGGATACTTGCGCACAGCCGGATGCTACTTCTGCAGCTGCAGTTGCTAACGAGAAGGGTTGCAAGAAGAGGGAAGAAGAAGAAGGCGATAATGCTAAAGCAGAACGCAGATTGGTTCACTCGCTGCCGCCCAAGCCAATTGGGCTATATAGGCGTGATTATCGGGACCGAGATTTCAATAGTGACGAAAGATACCACCACAAAAGGCCCCGCCTTGGCAATAATTACAGCGGAGACAGACATTCTTCTAGCAGGGAGCAATATTTCCCTTCCCCTAGAGAAAGATGTTCAAGGAAGAGACCGCGCGAGTGGGTAGAGTTTGATAGGAGGGACAGGGATCAAGCTAAGAGAAGAGAGCATTATAATAATAATACCAGGACTAGGCGGGATAATAGGGACTGGAAAGAAGGAAGGGGTTATTGGGAGAGGGACCGGGATTCGGATGAGATGGTTTTTCAAAAAGGTCCTTGGGAAGCTGATCGTAAAAGAGAGGACAAGACTGCCGTGAAAAACCAGCAAGAGAATATTAAATCAGAGGTGCCTAAAAAAGTGCCGGAAGAGCAAGCCCGCGAGTACCAGTTGGAAGTTCTTGAACAGGCAAAAAAGACGAATACAATAGCTTTCCTCGAAACTGGGGCAGGGAAAACACTCATTGCCATCCTCCTCATGAAGAGTATTTCCAGCGACTTGCAAAGGCAAAACAAGAAAATGCTTGCACTATTTCTGGTACCCAAAGTTCCACTTGTTTATCAGCAAGCAGAAGTTATTCGCGAGCAAACAGGTTACCAAGTAGGTCATTACTGTGGTGAAATGGGTCAAGATTTTTGGGATTCTCGCAGGTGGCAGAGGGAGTTTGAAACAAAACAGGTTCTAGTGATGACCGCTCAAATTCTGTTGAACATTCTCAGGCACAGCATAATAAAAATGGAAGCAATCAATCTCCTTATTCTGGATGAGTGTCATCATGCTGTAAAGAAGCATCCTTATTCATTAGTGATGTCTGAGTTCTATCATACAATTCCAAAGGAGAAGAGACCATCTGTTTTTGGAATGACTGCTTCTCCTGTTAACTTAAAGGGTGTTTCTAGCCAAGTAGATTGCGCAATAAAAATACGTAATCTTGAAAGTAAACTAGATTCCATAGTTTGCACAATAAAAGACCGCAAGGAGCTGGAGAAACATGTACCGATGCCATCCGAACTTGTGGTGGAGTATGACAAAGCAGCCAGCTTATGGTCCCTTCATGAACGAATAAAACGAATGGAACTGACGGTTGAAGAAGCTGCACAATCAAGTTCTAGAAGAAGTAAATGGCAATTTATGGGAGCTAGAGATGCTGGGGCCAAGGAAGAGTTGCGCCAAGTTTATGGTGTCTCCGAAAGAACAGAAAGTGACGGGGCTGTTAATTTAATTCAAAAATTGAGAGCTATAAATTATGCACTCGGTGAACTGGGACAGTGGTGTGCCTACAAGGTAGCACAATCATTTTTGGCCGCTTTACAAAATGATGAAAGGGCAAACTACCAACTTGATGTTAAGTTTCAAGAATCATACTTGAATAAAGTTGTCTCTCTCTTACGATGCCAATTATCAGAGGGAGCTGTATCTTACAAGGATACTGAAGTCGCTGACAAAGAGAGTTGTGTTGCTCAAGACGGGAATGAACTTGATGATATTGAAGAGGGAGAGCTTCCAGATAGTCACATTGTCTCTGGTGGTGAGCATGTGGATGTGATAATAGGAGCTGCTGTAGCTGATGGAAAAGTGACCCCAAAAGTGCAGTCACTGGTTAAAATTCTTCTCAAGTATCAGCAGACAGAGGATTTCCGAGCAATTGTTTTTGTTGAGCGAGTTGTGACAGCCTTAGTTCTTCCCAAGGTTTTTGCAGAGCTTCCATCTCTGAGTTTTATCAAGTGTGCAAGCTTGATTGGGCACAACAATAGTCAGGAAATGCGGACAAGCCAGATGCAGGACACAATTGCCAAATTTCGAGATGGTCGTGTGACATTGTTAGTTGCCACTAGTGTCGCTGAGGAGGGACTTGATATTCGCCAATGCAACGTTGTCATTCGCTTTGACCTTGCTAAAACTGTTCTGGCATACATTCAGTCTAGGGGTCGTGCAAGAAAGCCTGGGTCAGATTACATATTGATGGTTGAGAGGGAAAATTTATCACATGGAACATTTCTGAGGAATGCTAGGAATAGCGAGGAAACCTTGCGGAAAGAAGCAATTGATAGAACTGACCTTAGTCATCTTAAACATTCTTCAAGGTTAATTTCAGTGGAAACAGCACCCGGTACAATATACCAGGTGGAGTCAACTGGTGCTGTTGTGAGCTTAAATTCAGCTGTCGGACTCATCCATTTTTATTGCTCTCAGCTACCAAGTGACAGATATTCCATTCTTCACCCTGAATTTGTTATGGAGCCCCATGAGAAGCCAGGGGGCCCAATTGAATATTCATGCAAGCTTCAACTCCCCTGCAATGCGCCATTTGAAAAACTTGACGGTCCTGTATGCAGTTCAATGCGCCTTGCACAACAGGCCGTTTGTTTGGCTGCTTGCAAGAAGCTCCATGAGATGGGGGCATTTACTGACATGCTATTGCCAGACAAGGGAAGTAGGGAAGAAGGGGAAAAGGTTGACCAGAATGATGAAGACAATCCACTCCCAGGAACTGCTAGGCACAGAGAGTTCTATCCTGAAGGCGTAGCTAATATTCTCAAGGGAGAGTGGATTCTATCAGGAAGGGATGGATGCGACGGCTCCAAATTACGTCTGTATATGTATGTTGTTAAATGTGTGAACATTGGCTCCTCAAAAGATACATTTTTAACTCAAGTTTCAGATTTTGCAGTACTGTTCGGCAAAGAGCTGGATGCAGAGGTGTTATCGATGTCAATGGATCTCTTTGTTGCTCGAACCATGAAAACAGAGGCATCTCTTGTGTTCCGAGGTTCAGTAGATATTACAGAAAGTCAGTTGGCATCTCTTAAGAGTTTTCATGTAAGGCTGATGAGCATTGTACTCGATGTGGATGTGGAACCTTCAACCACCCCGTGGGATCCGGCAAAGGCATATTTGTTCGTCCCTGTGGTTGGCCATGAGTCTGCAGACCTTACAAAAGAAATTGATTGGAATCTGGTTGTAAAAATAACTGAGACAGAGGAATGGAGCAATCCCTTCAGAGAGCTCGACCAGATGTTTACCTTGGCACAAATGAGCGAACGTTAGGCGGGGATCGAAGGGAATATGGTTTTGGGAAATTGCGTCATGGCATTGCTTTTGGACAGAAAGCCCATCCCACATATGGTATTAGAGGAGCTGTAGCACAGTTTGATGTTGTAAAAGCTTCTGGATTGATTCCAAACCGCAATGCTATTGAGATGCAAAAGAATTTGAACAAAGGTGAATTAATGATGGCTGATAGATGTGCAAGTGTAGAAGATTGGTGGGAAGAATTGTAACAGCTGCTCATTCTGGGAAGAGGTTTTATGTGGATTCTGTATGCTATGATATGACTGCAGAGAATTCATTTCCAAGGAAAGATGGTTATCTTGGTCCTTTAGAGTACAGCTCATATGCCGATTACTACAAGCAAAATTGTGCGTGAAGAAGAGAAAAGCTGATCTATGAAGGAAACTGTGAACTACTCTCACTCTGAAATATGTGAGCTTGGTTAAACCATGTTCCGTTTTTCCCAGTGACAGTAGTTTATGAATTCCAACAGATTGGAGAGGCTTTTGTTTCTTCTTGCACAAGAATGGTGCATTTGGCTATCCGGTCTTGTGTATATTACCAACAGGTATGGAGTTGAGTTGATCTATAAGCAACAACCTCTAATAAAAGGTCGTGGTGTTTCATATTGCAAAAATCTCTTATCACCTCGATTTGAACAGTCAGAAGGTGAATCTGAGGAGACTGTTGATAAAACATATTATGTGTTCCTCCCTCCGGAGCTGTGTTATGTACATCCACTTTCTGGATCACTTGTTCGAGGTGCCCAGAGATTGCCATCAATAATGAGAAGGGTTGAGAGCATGTTGCTTGCAGTTGAGCTCAAGGAAATAATTAATTATCCTGTTCCAACTTCAAAGATATTGGAAGCTTTGACTGCTGCTTCATGCCAGGAGACATTTTGTTATGAAAGAGCGGAGCTTTTGGGTGATGCATACCTGAAATGGGTTGTTAGTCGATTTCTTTTTCTTAAGTATCCCAGAAACATGAAGGGCAGCTCACTAGGATGAGACAACAAATGGTTAGTAACATAGTATTGTTTCAGTTTGCATTGAGTAGAGGACTTCAGTCATACATCCAAGCAGATCGTTTTGCTCCCATCTAGATGGGCTGCTCCTGGGGTTCTGCCCGTCTTTGACGAGAAGTTACCAAGGAAGCAGAATCTGGTTTATTTGATCAGGAAAGTTCGCCTGTAAATGGTGATGGGTATGAAGATCACGAAATGGAAGATGGGGAGCTTGAGAGTGATTTAAGTTGTTACAGAGTCCTCTCTAGCAAGACCCTGGCAGATGTTGTGGAAGCACTCATTGGAATCTTTTACGTGGAAGGCGGAAAGAAGGCTGCAAACCACATGATGAAATGGGTGGGAATAAAGGTAGATTTTGATCTGGAAGAGGAGATGGAGTGCACAACTAAGTCATGCAATGTTCCAGAAAGCATACTTAAGAGTGTGAACTTTGATTATTTGGAGAAAGCATTGAACATTAAGTTCAGAAATAAGGGGTTGCTGGTAGAAGCTATTACCCATGCTTCACGACCATCTTCTGGAGTTTCATGTTATCAGCGGTTGGAGTTTGTTGGTGATGCAGTCTTGGATCATCTCATTACAAAGCACTTGTTCTTCACTTATACAGATTTGCCCCCAGGCCGCTTGACTGACTTGCGGGCTGCTGCTGTAAACAATGAAAACTTTGCACGTGTTGCGGTGAAACACAATCTCCATGTACACCTTCGGCATGGGTCGAGTGCTCTTGAGAGACAGATACGAGAGTTTGTGAAGGAAGTTAAGGAGGAATTGTCAAAACCAGGGTTCAACTCGTTTGGTTTGGGGGATTGTAAAGCTCCAAAAGTTCTTGGCGACATTGTCGAATCCATCGCAGGGGCTATTTTTCTTGACAATGACCGTGAGACTTCAGTTGTGTGGAAGGTTTTCCAACCTTTGTTGCAGCCGATGGTGACTCCAGAAACGCTCCCAATGCATCCGGTACGGGAGCTACAAGAACGATGCCAACAGCAGGCTGAGGGTTTGGAGTACAAATCCAGTCGAAGTGGAAATTTGGCGACCGTGGAGGTGTATGTAGAAGGGGTTCAGGTTGGAGTAGCTCAGAACCCACAGAAAAAGATGGCACAGAAACTAGCTGCAAGGAATGCACTAGTAGTTTTAAAAGACAAGGAAATGGCCGAAGCAACTGCTACTAGCAAGCTGAAGGAGAGTGGTGGGAAGAAGAAGAATGGGAGCTTAACATTTGCTAGACAGAGCTTGAATGATATCTGCTTGCGGAGAAATTGGCCGATGCCAACATATCGGTGTGTGAACGAAGGTGGCCTGCGCATGCAAAGAGATTTACGTTTGCGGTTCGTGTGAATACGAGTGATAGGGGATGGACGGATGAATGTGTTGGAGAGCCTATGCCAAGCGTGAAGAAGGCGAAAGACACTGCTGCTGTTCTTCTCTTGGAACTACTCAATACGCGGTACCCCTAACCCTAATATTTGACCAAGCCTAATAGTTTAATTAGTGGTGGTGGTGGTGTAAGTTTATTGTAGGGTAGAGTTAGACATTTAGGCAGCGCCTCTGTTGTAACTTTATGCACTTTATGCTTTTAAATTAAACTTGGATTTTTATAAGCCAGCTCATCCGCTCAGCTGAGTCTGTCAATTGTTCAATATAAATGCAAGTAGAAATTGAATTTCGTACAAAAAAAAAAAAAAAAAAAAAAAAAAAAAAAGT

>transcript_42 full_length_coverage=2;length=6602

GGGGCACTTCTCTCTCTTGTTAAAGTTATCGTTTGCACCTCATCGTCCTGTCATTTGGGCGCTCTCCCTCTTTCCGTTGCCGGCACTAGGTTGAGAAAAAGGAGGAAAATCGAGGGGTTCATCTCTTTCTTCTTTGGGTCCGAAGATTTAAAAGAGACGATATATAGATCGGAATAGAAGATACTAATATAGGTATAGATATAATATATAACAATGTCGGAAGAGGAGAAGTTGTTGAAGGAGGCTAAGAAACTGCCATGGGAAGATCGGTTATTACACAAGAACTGGAAGGTTAGGAATGAAGGGAACATCGACTTAGCCGCCTTATGCGGTTCCATCACGGATCCCAAGGATCATCGCCTTCGTGAATTCGGACCGTTGTTTAGGAAGACGGTAGCGGATGCAAATGCACCTGTACAAGAGAAGGCTCTAGATGCTTTAATTGCCTACTTACGAGCTGTTGATGCTGATGCCGGAAGGTATGCAAAGGAAGTATGTGATGCAATTATAGCAAAATGTCTCACTGGTCGGCCCAAGACGGTGGAGAAGGCTCAAGCAGTATTCATGCTTTGGGTAGAACTGGAGGCTGTTGACGTTTTCCTGGATGCTATGGAGAAAGGGATAAAAGCCAAAGTTGCAAAAGCTGTTGTCCCTGCAATTGATGTGATGTTTCAAGCCTTAAGTGAATTTGGGTCAAAGGTTGTTCCCCCAAAAAGAATTCTAAAGATGCTTCCTGAACTCTTTGACCACCAAGACCAAAATGTCCGTGCATCCTCCAAAGGATTGACTCTTGAGCTTTGTCGTTGGATTGGAAAAGATCCAGTGAAATCAATATTGTTTGAGAAAATGCGGGATACAATGAAAAAAGAGCTAGAGGCTGAGCTTGCCAATGTTACGGGGTCAGCCAGGCCCACTCGCAAAATAAGATCTGAACAAGATAAGGAGCCAGAACAGGAAGCTGTTGCTGAAGTTGTTGGTCCTGGTCCATCTGAAGAATCTGCAGCTGATGTTTCTCAGGACATAGATGAGTATGATCTTGTAGATCCTGTTGATATATTAACTCCTTTGGAAAAGTCAGGGTTTTGGGATGGAGTGAAAGCCACCAAATGGTCAGAAAGAAAGGACGCTGTTTGCAGAGCTAACCAAGCTTGCTTCTACTAAAAGGATATTGCCTGGTGATTTCACGGAAGTTTGTAGGACACTTAAGAAGCTTATTACGGACAATAACATAGCTGTTGCAGCTGAAGCTATCCAAGCTATTGGGAATCTTGCCAGGGGCCTAAGATCCCATTTTTCTGGGAGTTCACGGTTCTTGTTGCCAGTTTTACTGGAAAAACTGAAAGAGAAAAAACCTACTATAACTGAGGCGCTTACTCAACTCTCCAAGCAATGCACAAGGGCTGGGTGCTTAAATCTTCCTGACATTGTGGAAGATGTTAAAGTGGCTGTTAAAAATAAAGTTCCTCTTGTGCGCTCCTTGACTTTGAATTGGTGACATATTGTATTGAGACTAGCAATAAGGCCGTTGTTCTCAAGTTGCACAAGGATTATGTTCCGATCTGTATGGAGAGCCTCAATGATGGGACACCAGATGTGAGGGACGCAGCCTTTTCGGTTTTGGCGGCAATCGCTAAGTCGGTTGGTATGAGGCCTTTGGAAAGATCACTGGAGAAACTTGATGATGTTCGAAAAAAGAAGCTTTCTGAAATGATTGGGAATTCAGTTGTTGGTCTGCCTACTGCCGCGAACTCAGCTGTCCAAACAGCGGGTGGGAGTGTATCATCTTCGCAGGCTTCAGAAGGACCATTTGTTAGAAATCGGCAGCAAGCATGCTTAGTGGGAAGAAACCTGTGCAGGCAGCTCCTTCTAATAAGAAAGGAGGGCCTGTAAAATCGGTTGCAAATAAGAAAGGAGATGGAGCTGGACAGTTGAAAACTTTAAAATCAGTTGAACCTCCTGAAGACATTGAGCCAGCAGAGATGAGCCTTGAAGAAATTGAAAGCAGATTAGGTTCTCTTATACCCGCGAATGCTGTTTCTCAGTTAAAGAGCACTGTATGGAAAGAAACGACTTGAAGCAATTGTCTCTTTAAAACAACAGGTGGAAGGCATTCAAGACCTTGACCAGTCTGCAGAGATGTTGATTCGTTTACTTTGTACTGTCCCCGGGTGGAATGAGAAAAACGTTCAGGTCCAGCAACAGGTCATTGAAGTTATTACTCACATAGCTTCTACTGTGAAGAAATTTCCCAAAAAATGCGTTGTGCTTTGCCTTCTAGGTATAAGTGAACGGATAGCAGATATCAAGACCCGTGTTCATGCCATGAAATGCCTTACTGCTTTTTCCGAAGCAGTAGGTCCTGGATTCATTTTTGAGAGACTTTACAAAATCATGAAAGAGCACAAGAATCCTAAGGTTCTAAGCGAGGGTATATCGTGGATGGTTTCAGCAGTTGATGACTTTGGCGTGTCACTTTTGAAACTGAAGGATTTAATTGATTTTTGTAAAGATACTGGACTGCAGTCCAGTACTGCTGCAACTAGAAATTCTACTATCAAGCTTATTGGCACTCTACATAAGTATGTCGGACCAGATATTAAAGGGTTTCTTGTTGATGTCAAACCTGCACTTCTTAGTGCACTTGATGCAGAGTATGAGAAAAACCCATTTGAGGGTGCCTCTGCAGTTCCCAAAAAAAAAACTGTAAGGGCAGCAGACTCTACAATGTCTATGGCTGCTGGTGGGCTAGATAGCTTGCCACGTGAAGATATTAGTGGAAAGATTACACCTGCTCTGTTAAAGGGATTGGAAAGTCCTGATTGGAAGGTCCGTTTGGAATCAATTGAAGCTGTAAATAAAATCTTGGAAGAGGCCAATAAGCGGATTCAACCTACTGGAACTGTGGAGTTATTGGTGCTCTTAGAGGGCGTCTCTTTGACAGCAATAAAAATTTAGTCATGGCAACTTTGAATACCGTTGGTGCTGTTGCGTCTGCGATGGGTCCAGCAGTTGAGAAGGCAAGCAAGGGTATTCTGTCAGATGTTTTGAAATGCCTGTGTGACAATAAAAAACATATGAGAGAATGTACTTTGACCACTTTAGATTCTTGGCTTGCCGCTGTTCACCTCGATAAAATGGTTCCTTACATTATGACGTCTTTAACGGATACCAAGATTGGTGCGGAAGGACGCAAGGATCTATTTGATTGGTTATCAAGGCAACTTTCTGGATTAGTTGATTTTTCTGATGCTGTACACCTGCTGAAACCTGCAGCAGCAGCTATGACGGATAAATCATCAGATGTTCGAAAAGCAGCAGAAGCATGCATTGCTGAGATTCTGAGAGTGTGTGGACAAGAAACAGTGACCAAGAGTTTAAAAGATTTACACGGTCCAGCTCTAACTCTTGTTCTTGAGCGGCTGAAACCACATGGATCTTTTCAAGAATCATTTGAACCAGTGAAAGCAGCTTCAATGGGGGGTAACATCTAAAATCATTTCCAAAGTTGGAAAATCTGGCACCAATGTTTTACCAAAGCATGGAAACAAAGCCATATCTTCGAGAGCTGTTGCGACTAAGGGTTCAAGGTCAGACTCAATAATGTCTGTTCACGATATTGCTGTCCAGTCACAAGCTTTGTTAAATGTCAAGGATTCAAACAAGGAGGATAGGAGAGAATGGTTGTTCGCAGGTTTAAATTTGAAGAGCTTCGAATAGAACAGATTCAAGATCTGGGAGAATGACCTTACAAAGTATTTTAGAGAGGATTTGCACAGGCGGCTTCTAAGCACCGACTTTAAGAAGCAAGTAGATGGCCTTGAGATGCTACAGAAGACACTTCCATCCGTTGGGAAAGAAATAATTGAAGTATTAGATATACTTCTGAGGTGGTCTGTTTTGCGGTTTTGTGAATCTAATACGACATGCCTGCTGAAGGTGCTTGAATTTCTTCCCGAACTGTTTGACTTCTTGAGGGGTGAAGCTTACATTTTGACTGAATCGGAAGCGGCGATATTTCTCCCCATGCTTGATAGAGAAGTCTGGGCATAACATTGAGAAAGTACGAGAAAAAATGCGGGAATTGACAAAACAAATCGTCAACATATACTCCGCAGCAAAATCTTTTCCCTATATTTTGGAGGGTTTGCGCTCTAAGAACAACCGAACTCGGATAGAATGTGCTGACCTTGTTGGATTCTTGATTGACCATCATGGAGCCGAGATTGGTGGTCAATTAAAATCCTTGCAAACTGTTGCAAGCTTGACAGCAGAGAGAGATGGTGAACTTAGGAAAGCTGCCCTGAACACCTTAGCTACTGGTTATAAGATTCTTGGTGATGACATATGGAAATACGTTGGGAAGCTAACTGATGCTCAAAAAAGCATGTTAGATGATAGATTTAAGTGGAAGGCCCGAGAAATGGAGAAAAGGAAGGAAGGAAAGCCAGGTGAAGCTAGAGCTGCTTTAAGACGTTCTGTTAGGGAGAATGGGTCTGATGTGGCAGAGCAAAGTGGAGAAGTTTCACGATCCGTCGCTGGCCCAATCTTTGCAAGTTCTAGGGAATCCTATGCTTATCAGGAGCCTCAAATGGATAGAAATTTGATGCCCAGAATGCTTCCTACTGCAACAGCTCCCACAGACTGGAATGAAGCTTTGGATATCATTTCATTCGGTTCTCCTGAGCAGTCTGTCGAAGGAATGAAAGTTGTGTGCCATGAATTGACCCAAGCCACCAATGATCCTGAAGGCAGTGCAATGGACGATCTCGTCAAAGACGCAGATAGACTCGTTTCGTGCTTAGCAAACAAGGTACCCAAGACTTTTGACTTCAGTCTGACCGGAGCTTCGTCGAGGTCTTGTAAATATGTTCTGAACACGCTCATGCAGACATTTCAAAATAAAAGACTTGCTCATGCTGTTAAGGAGAGTACTCTTGACAGCCTAATTACTGAGCTTCTCCTTTGGCTTTTGGATGAAAGGGTTTTCCGCATATGGACGATGGCAGCCAACTTTTGAAAGCCTTAAATGTTTTAATGCTCAAGATTCTGGACAATGCAGATCGGACATCTTCTTTTGTTGTCCTGATTAAACTCTTACGTCCGCTTCACCCTTCAAGATGGCCATCTCCTGCTTCAAATGAGACCTTTGCTGCTAGAAATCAGAAGTTCTCTGATTTGGTAGTCAAATGTCTTATTAAACTTACTAAGGTTCTTCAAAGTACGATATACGAAGTTGACCTTGACCGTACCTTCAAAGTATACATGTCTACTTACAAGAATTGGGGATGGAAGAAATCAGGAGAAGAGCTGGAGCTGATGACAAACCATTACGCATGGTGAAAACTGTTCTACACGAACTCGTTAAGCTTCGTGGTACAGCAATAAAGGGTCATCTTTCTATGGTTCCTATAGACATGGAACCTCAACCTATCATTCTTGCCTACATTGATCTTAACCTTCAAACTCTAGCCGCGGCTAGAATGTTGACTCCATCTGGACCCGTGGGTCAAACTCATTGGGGTGATTCAACAGCTAACAACCCATCTCCGGCCACTCATTCTGCGGATGCTCAATTAAAGCAAGAACTTGCTGCAATCTTCAAGAAAATCGGTGATAAGCAAACATGCACAATCGGTCTCTACGAGCTTTACCGCATAACTCAACTTTACCCTAAAGTTGATATATTTGCTCAACTCCAAAATGCCAGTGAGGCGTTTCGAACATACATAAGAGATGGTTTAACTCAAATGGAGAAAAAATGCAGCAGCTGGAAGAACACCTTCAAGTGTACCAATGCCAACTCCTCCCCAGCTTCCTTAACTTCCCCAAAATTCGCCCCACTTTCACCTGTACATACAAATTCTTTAAACGAGGGCAAAGTGGTAAATGCAAAAACCCGAACCGACAAACTTCAGTTTACCACCGTCATATACAGATGAAGATCATCATAATTATGGCGACCAAAGGAATGATAGATTTCCAACTGGAGTTACGAGCGGTACGCTGGATGCAATTAGAGAAAGGATGAAGAGTATTCAGTTAGCTGCGGCTGGAGGTGGGAACCATCCGGAAGCGGGGAATAAGCCGTAATGTATGTGAATGGGAATTTATCTCACGCATCGGGTGTAGCAGTGGAAGGTGTGCAGACTGCAGTACTTCCAATGGATGAGAAGGCGTTATCTGGGCTTCAGGCTCGGATGGAGAGGCTTAAGAGTGGTTCGATGGAACCTCTATAGGAGGAGAGGGGATAGAGATTTTGATTTTGTAGAGTGCAATGGAAGGGCAGTTGATGGTATAAAAAATATGTATACTTAGATGTTTTACGCTCTTTTGTATTTGGTATGGAAGCTTGTATTTATTCTCTTGAGATGTTGTCATGGAAGAGGTTAAGGCTAGGATGAGTATTATTTGGTATGTGCTAATGATTTGTTATGCACTGGAGTCGTGTGTCCAAAGACCCAGGCATTTTGTATATTTTAGTTGAGGCCTTTCGTTATTTTTGGCATGTAGCAATTGTTCACTGTTGAATGCAATATTTTTACATTATGACAATGTGTTATTTTTTTAAAAAAAAAAAAAAAAAAAAAAAAAAAAAAAAAGTA

>transcript_43 full_length_coverage=2;length=6673

GGGGTTTAAGATGGTCGAGCGAGTTGGTATAAAAGAAAGTTTAGGCGTGTTGAGCTGGGCGTGCCAATTATCACTGAAAGATACACCAGAGGGCACCGATTTTTCTTTCTCAGAGATTACGATCCAAGAAGAAAATATATACAACCCCTATAGAGGAGACTAAGATGGTGAGGTTCCATTCAAGGGAATCAAGGCCGTAAGGTACATGTCTTTAGTTCGTTTGCTCTTGGTTGAGATATTTTGAAATCAAGCAGTGAGAATAGACTAGAACGGAGAAAGGAAGAAAGAGAGGCCACTTTCATGATTCAAAGCTGATACAAAGAATCGAGGCTGGGGGACTGACAAGCAACTGCAAATCCAAATTTTATCAGAAAATTATTTATGAGCCGCTTTGAAAATAAACGATTTTGCTTCTTTTCTTGTTCTAGAAAGATACAGAGGAGAAGACCATGTCAAATCATATATTTTCGGTGATTCAGAAAAAAGGGATAGGGCCATCATCATGGGGAGCAATTTTCTCTTTTGGGTTTATTAATTGGCTTTGACAGTGTTTTGTTGGAAGCACTGAAAAGGTATTGAGAGATTTTTTCTCTCTCTGGTTTGGTGATGAAGTAAGAGGAATAATTTTAGTCTGGAAAAATTAAGAGTAAAATACAGATGAAAGAGGATCCCGTATGAAGAAGATCAAGACTAACCTATGTCATCTCATGGCTGGGCGCGCCAGTTTTATTTATTTACCGTTGGTCATCACCTTGGATCTGCTAGCTATTATTAGTTAGGGCCTCCTTCCAGTTATGACTGATAAATAGCCTTTCATCACGAGACTGTGCGGGATCCTACATGGTGTACGCTTAGGCAATATAGGCCCTATAGTGCAAATTAATGAAGGTGGGAGTCTAGTCAAATGTGCTTTATCTCAAAAGGGGCACAAACTCAGTAAAAGGTAAACCTTTGCCAGAATGTTGGTGAGTACTCTTCTCATTTGTATCTTTCCTCACAGATATCCTTAATGTTTCATTTTTCATAAGGCTCTAGACATACTTTCATCATTTAGCATATAATGTGCCTAGTTTTCTCTATTTGCATAATTTATCATCATTTCATCATACTTTAGTCAGTGTTGCTCATGTTATCGCATTACATATCATAAGTGTCTAAAATTCATACACCTTTGAGTCCTGATAAATTGGACTCGGAACTTGGGTGAGTAAGCCCACTTCGACCCTAGACTATTTTGGACCATATTATTCGTGGGTTGTTTTTTTATTTTTTGTGCTCAAATATTTGTAATCAAAATTGTATTTAATCTCGGTTGTTGGTGAACCACTTAAAAACCAACATTGTTTTAGAATGGTTATTGCTCATCCACAAAGCAAGGAATTCTAGGTTGTAGGTGAATCCTTTGAGAAATCTTCCCGACTTGGTTTGTGGTTATCCCTTAAGAAACCATAGGTTAGGTTTTTGGTAATCCCCTTAGAAACCATAGGTTGTTGGTCCGCCCTTGAGAAACCATCAGTTAGGTGTTTAGCTCAAAACCCACCGGTTGTAGGTTAGGCTTTGAGAAACCTTCTATAGAGTAGGTACGGTTTGTTGGTGATCACCCTCAAACCAATGGTTATTGCTTAACCAAAACGCATTCCATTGTACAGGATTTTTTGGTTTCCCGTTTTAAACCGAATGCATGTAGTGTGAGTTAATCTGAGTCCGGTAAGGCTTGGGAGTGGATGTAGGCATTGTCGAACCACTATAAATCCCGGTGTTCTTTTATGCTTTACTTTATTTTATTTACATGCTCAATCAATATCTTTCACAACGAATCAATCTTAAATAATTTAATTCTTAAAATACTATTCAACTCAAATTTGGATTCTCAAGTATAATCAAAATTCACTCAACTTGGTTAAAATTAAAATTGGAAAATGATTTTCAAAAAGGTTCAAAAGATTATATTCACCCCCCCTCTAGGATCCTTAGATGCACTTTCACTTGGTATCAGAGCAGGTTACTCAATATACGTTTAAATACCCGAGTGAGATCCTTTAGGTTTCATAGTCTTCTTTTCTTCCATAATGACTTTCGTCGATAGTTCCATTAACAAAGCACCTATCTTTGATGGTACGAACTATGCGTATTGGAAAACTCGAATGATGATCTTTCTTAAATCTGTTGATGAGATATTATGGATTTGTGTGCGTGAATGTTGGATATAACCCACTAAGATAGATCTTGATAACATTTCTAAGAATCTTCCTAAACCATACTGTGAGTGGACCAACGATGAGAAGAGCGACAATCAAATCAATAGCAAAGCTTTAAATGCCATTTTCTGTGCTGGCACTCCTGACGAGTATAAATGTATCCTCACTTGTACCTCAGCCAAACACGTCTGGGACATCCTAGAGATGACCCATGAAGGTACCTCACACGTTAAGAGCTCCAAACTCCAAATTTTGAAACATCAATTTGAAACTATGAAAATGGAGTCTGACGAGACCATTAATGAGTATTGCACTAGATTAAATGAGGTTGTCACTTCCAGTTAGGGATTAGGAGTCTCATATTCTGATTCTGATATCATCTCCAAGATACTTAGATCCTTGACCAACATATGGATATCAAAGGTTGATGCTATTGAGGAATCCAAATACATAACTCAAATGAAACTTGAAGATCTTATAGGAAACCTCCAGACTTATGAGATGAAGCATGAAGATCCCAAGAAGAAATTCGTTGCTCTAAAAGCTTCTAAGAAATTACTTGACACATTTGATGAGTCTTCCAAACATGCACTTGAAAGTCTTGAATTTATAGAAAGTCTTCAAAGGGAACTTGCATTAATTTCTAAGAAATATAAAAAGTTCTTCAAGAGAGGTTCAACCTCTAAAAATAAACATGATGTTGGCAAGTTTCACACTTCGAGAAAAATTGTGGACACATCGTTTAATCCTGAATGTTATGAGTGCCACGAATATGGTCACATATCTCCAAACTGCCATAACCGAAGGAAAGACAAGAAAAGGTTTTCGAAGACTAAGGCGATGGCGGCGATATGGAGTGAGAGTAGCGAAGAAGATAACGAAGATGGTGACTCTGGATCTGAAGATGAAGATAACTGGGTACTTGCTTTGATGGTCAAGAATACTATATCTGATGAATATGTGGAAGACACTGAGGATGAGGATGAACTTGAGACTGAAGATGGAGAGATTAAAAATGAGGAGACTCTCCAAGATTCTTTTAACAACCTGTACAAGGAATTCGTGAGAGTTGGTCTTAAAAACCAGAAATTGGAAGAACTGAACAAGAACTTACTTTCTGAAGTCCAAGCTTTGAAATCCATGGAATCTGCTCGTGATATTTCTTGCTCCAAATATGTTGAAACCGAAAAAATCATCACTACTCTTGAATCAAAAATTTGTGAGCTGAATGATCTGGTGGAAGAATTAAAAAGTAAAAATGACAAATTAAAAGATAGAGACCATCGGTATGAAAAAATGAAGATGGAGTTTGACACTGCTCCCGTAGAGATCATGAAATTGGAAGACAAGCTTGGGGTGTTAAAAAAATCCAACAAGGAACTAACTGCTAAAATTGCTGAGTTATAAAACATAAATTCTAAGTTCCTTAGTGGTCAGAAGAAATTGGATGAGATGTTAAAAGCTGGTAAGAGTTATTATGATAGGACTGGACTTGGGTATCTCTTCGAGACAATGAATAATTTAAATCCCAAGATAACTTCCATCAAGGTTACTCAAAGACCTCCAGCTCGATACAACACTCATGGGTACAACTATTATATATTGAACACGAAAAACCTCCATTGCTATTATTGTGGTATTCGGGGTCACAAACAGTTTGATTGTCGACTTAAGATGGTTAAGAAGCAAGCTAAGCAACAAACTTGGAAATCCACTCACATGAAGCAAGTTTACAGGATTGTGAAAATACACGATGAACCTTGGTGGCACCGCACTTCGAAGATCTCTGCATCTCAAAAGATGAAAGGAAAGATGCTTGAGAATTGCTTTATTGCCATGACCACACCTCCATTGGAGTGTGTTATGTGACCGTGTCACTTTTCTCACCGCATTCCTTTTTGTTTATTTTTGTTTATTTATTTTTGGTTGTGTGTGTGAGTCTTAGTGTCTTTTCATTGAGTTGTATTTTCTTGCATTTTGTGTTGAGTTCACACATTTCTTTTTAGTGTCATTCATGTGTCTCATGAGTGTCTCATACTTGCATTTTTCCCATTCATGCATTATTCTTGATTATTATTATATATGGCATTTATTATGACAAAAAGGGGGAGAAAATGTATTTCTCTTGTGTTGTTTTGCAGGTATGAAGACAAGGGGGGCAAGATATACGCCTGAATAATATTACAAATCTCATTGGGAGAAAAACTGAATTTCTTGAACTTTAAGAAATCTCGATTCTTAAAGACGAAGAAAGTATGATTCTTGAATATGAAGAAATCTCAATTCTTGATGATGAATAAAGTTCATTTATTGAAGATGAAGAAAGATCATTTCTTAAAGTTCAAGAAAGCGCATTTCTTAGAAATCTAGAAAGCTCAAGTGTGCAAGAATCGATCCTCAAATCAACCAAGATGGAAAAAGTGTTTTGTTATAAAATTATCATTGGGGGAGATTGAAACGGCACCGCATATCGAATGTCATTTTTATGATAAATGGGTTTGTTAGTTTAGGTTGCCACACCTCCCAGTTGCACCGAATTTTGTGCAGAGTGGGATTTGCATTTCATTGCATTGCATTTCATGCATGTGTGTGTTGAATGGTTGATTGTATGATCCTGAGGTTTGAACCAAAATCTTTTGTCATTTTATTTTAGTGTTATTTTCAAAAACTTGCTCTCTTTAAATCCGTTATTGAAAAAACTCTATTTAAAATCCTTTTCACCTTAAGAAAATCTTTCATTTCAAAAATATATAATATTAATAGTTTTTGTTGGGCCATTTTTTTTGTTGGACTTGGTTTGGGTTCTCTTGACATCATCCTTGCCCGGTTTGTGATAAGATCCTTAGATCTTACTAGTTTCTCTTCGCATGTGACATGTGTCTGGCTTGTGCGTGTCTTCCTTTGGTTCGCTAATTTCATATCTTATGTCCGTTGCTAATCATTGTAACATTTATGATTTACATTTTAGTGCGTGAGTCTCATAGCACATGAGCTTTTAAAGACGTGACCATTATTTTCCCTTATCCGTTGGCCATGCATCCAGGCTGTCCAGTAGGTGATCTACAGGTGGTGGATAAGCACGATAAGATCCTAATCTTCATTTTTCAATTTTTTTTTTGTTTTTTTCCCGTCTATTGCTTCCTATTCTCTGGCGATATGCCTTGGCATTTCAGACAACATCTTTCTTAAAAAACCTGCATCTTTTTGCTGCCTCTCCATCTTATCTCGTCCAGCTGTTGTGATTTGAATTTGGTTGTGCACTCTGTTCCTGGACAAACCAGTTTCTAAGTTCATTCTATTTTTTTTTTTTATATTCTTAGCGTTGGTCTTTGCTTCGCCAGCTGGTGTTTGATCATTGGCTTGAGTTTCTAACCGTAGGATCAGTTTTTTGGTTTATGCTATGTCATTTGGATAAGGGTCTCATCCCTTGTTTTGTTGATTAGGTCCTTCCTGGTTTTTTGCCGACTGTAAAGGTTTGAGGAGCTTGGATCCTTTGTGACCGTTGAAGATGGTCGGGAGAGTTGGTATAAAAAAGTTTAGGCGTGTTGAGCTGGGTGTGCCAATTATCACTGAAAGATACACTAGAGGGCACCAATTTTTCTTTATCAGAGATTACGATCCAAGAAGGAAATAAATACAACCCCTATAGAGGAGACTGAGATGGTGAGGTTCCGTTCAAGGCCGTAAGGTACAGGCCTTCAGTTCGTTTTCTCTTGGCTGAGATATATTGAAATCAAGCAGTGAGAAGAGACTAGAATGGAAAAAGGAAGAAACAGATGCCATTTTCATCAATCAAAGCTGACACAAAGAATCGAGGCTGGGGGACTGACAAGCAACTGTAGATCCAAATTTTATTAGAAAATTATTTCTGAGCTGCTTCGAAAAGAAATAATTTTGCTTCTTCTCTTGTTCCAGAAAGTTACAGAAGAGAAGACCCTGTCCGATCATATATTTTTGGTGAATCAGAAAAAAGGGATAGAGTTGTCATCATGGGGAGCAATTTTCTCTTTTGGGCTTCTTAATAGGCTTTGACAGTGTCTTGTTGGAAGCTCTTAAAAGGTATTGAGATACTTTTTCTCTCTCTCGCTTGGTGATGAAGTAAGAGGAATAATTTTAGTCTGGAAAAATTAAGAGTAAAATATAGATGAAAGAGGAGCCCGTATGAAGAAGATCAAGACTAACATATGTCACATCGTGACTGGGCGAGCCAGTTTTGTTTATTTACTGTTGGTCATCACCTTGGATCTGCTATCTATTATTAGTTAGGGCCTCCTTCCTGTTATGACTGAAAACTAGTCTTTCATCACGAGACTGTGTGGGATCCTTCATGGTGTACTCTTAGGCAATATAGGTCCTACAATGCAAATTAATGAAGATGGGAGTCTAGTCAAATGGGCTTTATCTTAAAAGGGGTACAAACTCAGTAAAAGGTAAACCTTTGTCAAAAAAAAAAAAAAAAAAAAAAAAAAAAAAGT

>transcript_44 full_length_coverage=2;length=6584

GGGGAACAAGACACAAACAATCACAATCTTTTTTATGCATTATGAAGCACATAAATCATCTCTCTGAATCACAACATTTCATTCTCTCTCGTCTTTTTTACTCTACATTTCTCTGTTTCTCCACCATGAAAGTTTCCTTGCTGTAAAGCATATCTCATACTTCTGTGTATATACATTTTTCTCTCTCTCGACCAGGCATTGCACCGCCATGGAAGCGCTGATCGAGCTCTGCGACCTGATCGCACAGAAGCCCACACAATTCGCCGATAAACTCGCATGGGATTTGCAGCAGATGCCCTCCTCCCGAGTTGCTCCTCTCTGGATCGCCTAGGGTTTCGAGGTCTCAGCTCAATGCTGTTCTCGTCATTGCCAGATTTCTTTCCAAATGCTCTGACGCGTCCGACTACCGCCCCAAAGCAGGCTGTACTCGAGTTTATCCGCTCAATTCCGGCGTCATTCAATCATTCTTTCTGGCCGCAATCGTACGGTACGGATTCCATTTCGTCCTTCTTCGTAGATTTCTTGGGGTACGTATCCAAGGCGACCGAGTTGTCGTCGGATTTTTCCACGGAGGTTTCCGGGTTCATGGGCGAGATCGTCATCTCTGCTATCAGTAATACCGGTGGGAATACCGTAATTTCTAGGGTTTTTCTGGCAGCCCTTTCGCAGAACTTTCCACCAGTTTTGCCTTCTGACGCAGATAAGTTGGTCAATTTTCTTTTAGATCAGTACGTAATTGCGGTCCCGAGCTCGCCGAGAGAGCCAGTGCCTGCGGGTGGCGACGTCAGAGGCGTCATCGGCGCAGAGTTCACCGCTTAACGTAAACCAAAACGAGTCTTCGAGTTCGGGAAATGAAGCTAGCAGTGCTTCCAAAATTGCGGATGATGCCACAAGTGCGTCTTCTGGGGCATCTGTGATGATGAATGGGGGGAAATTTGATGTGGAAGAGTGGCACGGGGGGAGCATTATGGTGTGAATTTCGGGGTACAATGATGGAGGGGGGAGGAAACGCAATGTTTTAGGCAACAAGTTGCTGCATTTGAGGCGGAGTCCGTGAGAGTTTGGGAGAAACAAGAGATCGCATTCAAATTGATTGGGCACATTTTGGATAAAGTCCACATTGATCCTAAGCTCTCAGAGCAGGTTAGACTTATTGCTAAGAAGCAGCTACAATCCATGTCGGCTTTTTTGAAGATAAGGAAGCGAGATTGGACAGAACAGGGCCCGCTGCTAAAAGTTCGAATCAATACAAAACTATCCGTTTACAAAGCTGCAGCTAGGTTGAAACTTAAGAGTCTAGCCTCTCTCGATTCAGATGGGAAATCATCCAAGAGGTTGCTCCTCGAGACTCTTGCCTTGTTGATAGACGCTGCTGAAGCATGTTTACTCTCTGTGTGGCGAAAGTTCAAAATTAGTGAGGAGCTATTTGCTTCCTTGCTTGCCGGAATTGCACAAATTGCTGTTACACGTGGAGGTCAGCTGTTGCGGGTTCTGCTCATTCGCCTCAAACCCCTTGTGCTTACTACATGTGCACAGGCTGATAACTGGGGTGCGATGTTCGAGAGTGTGATGAAGACTAGTTGTGAGATAATTGAATTTGGCTGGAGTAAGGATCGAGCTCCTGTAGACACTTTTATTATGGCATTGGCCACGAGTATTACGTGAACGCAATGATTATGAGGAAGAGGTTCAGGATGGAAAGGAGAAAGGCAGTTCTTAATGTGCAGCTTAACGTAATACGCTTGCTAGCTGATTTGAATGTTCTGCTAACAAGTCTGAAGTGGTTGACATGATATTGCCATTATTTATTGAAAGTTTAGAGGAGGGTGATGCTTCAACTCCTGGTCTATTGCGACTCCGACTTCTTGATGCTGTGTCTCGCATGGCAAGTTTAGGTTTTGAGAAGTCCTATCGTGAGACAGTTGTTCTGATGACAAGAAGCTACTTGGGTAAACTATCAAGCATAGGATCTGCTGAAAACAAAACATTGGATGAAGAAGCCACCAAGGAACGTATTGAGACTCTTCCTGCAGGGTTTCGTTTGATTGCTAGTGGGCTTACTGGTACAAAATTGCGGTCAGACTATCGTCATCGATTGCTATCTTTATGCTCAGATGTAGGATTGGCTGCCGAGTCAAAAAGTGGGAGGAGTGGAGCAGATTTTTCTGGGACCTTTACTTCCTGCTGTTGCGGAAATTTGTTCTGATTTTGATCCTACTATGGATGTGGAACCCTTCACTCTTAAAGTTATTTCGCAACTTGTGGTTCTATATTGCTCTTTTTGGTCTGGCACCTCCCATACAGAAAAATCAGGCTCCAGCAAAGTCTGTTTCTACTACATTGAATAGTGTAGGAAGCATGAGTTCTGTTGCTCTTCAAGCTGTGGGTGGACCATACATGTGGGATATTCAGTGGTCTTCTGCTGTTCAGCGCATTGCTCTAGGGACCCCACCCCCTTGTTGTAAGCTCTGTAAAATGGCTTGAAGATGAGTTGGAACTCAATGCTCTTCACAACCCTGACAGTCGTAGAGGGAGTGGCAACGAGAAAGCTGCTGTAACCCAAAGAGCTGCTCTTTTCTGCTGCATTAGGTGGGCGAGTTGAGGTTTCAGCAATGAGCACAATTTCAGGAGTTAAAAGCTACCTATCTCCTTGCGGTAGCTTTTCTGGAGATAATACGTTTCAGCAGTAACGGTGGCATCCTTAATGGTGGCCCTAATTTGACTGCTTCTAGAAGCTCCTTCAGCTGTGTTTTTGAATACCTCAAAACCCCTAGTCTCATGCCATCTGTCTTTCAGTGTTTAATGGCAATTGTTCACAGGGCATTCGAAACAGCAGTGTTGTGGCTGGATGATCGGACATCCGAAACAGGCAATGAAGGCGAAATTAGAGAGTCTACTCTTTCTGCCCATGCATGTTTTCTCATAAAAAGTATGTCCCAGAGGGAGGAACACATTCGGGATCTCTCTGTCAACTTGTTGACCCAACTTAGAGACAGGTTTCCACAGCTTTTATGGAACTCTTCTTGTGGGGATTCCCTGCTATTTTCAGTTCATAATGAGACTCCTACTCTTGTCACTGATCCTGCTTGGGTTGCGACCGTTCGTTCTTTGTACCAAAAGGTTGTTCGGGAATGGATTATTTGTTCACTTTCATATGCTCCATGCACCAGTCAGGGTCTTCTTCAGGAAAAGCTCTGTAAAGCAAACACATGGCAGAGAGCGCAGCACACAACTGATGTTGTTTCTCTTTTATCTGAGATTCGGATTGGCACGGGTAAGAATGATTGTTGGACAGGCACACGGACAGCAAATATTCCTGCAGTTATGGCTGCTGCGGCTGCGGCATCAGGTGCAAATCTGAAATTGATGGAAGCCTTCAACTTGGAGGTGCTGAGTACAGGAATAGTCAGTGCAACAGTAAAGTGCAACCATGCTGGAGAGATTGCTGGCATGAGAAGGTTGTACGAGAGCATGGGTGGATTCCCTGCTCAAACAGGCTTTGGGATTGGTCTTGGCCTTCAAAGGTTGAGATCCGGAGTGTCTCCTCAGCAACCACAGCCCGAGAATGATGCATTTAGTGGACTTTTGCTTACCAGATTTGTGAATCTACTGCAACAATTTGTTAACACCGCAGAGAAAGGTGGGGAAGTGGACAAAACATCCTTTCGCGAAACTTGTTCTCAGGCAGCTGCGCTACTTCTCTCAAATCTGGATTCTGATTCAAAATCAAATATAGAGGGATTCTCACAACTCTTACGTCTTCTTTGCTGGTGTCCTGCTTACATTTCCACCCCCGACGTGATGGAAACTGGTGTATTCATTTGGACTTGGTTAGTTTCTGCTGCACCTCAGCTGGGGGTCTCTTGTCCTTGCTGAGCCTTGTTGATGCATGGTTATGGACCATTGATACAAAAAGAGGTCTCTTTGCATCTGAAGTAAGGTATTCTGGACCTGCTGCAAAATTAAGGCCTCACCTTGTCCCAGGGGAGCCAGAAGTGCAGCCTGAAACAGATCCCGTTGAACAAATAATGGCTCACAGACTATGGCTTGGATTTTTCATTGATCGCTTTTGAGGTAGTCCGATACAACAGTGTTGAACAACTCTTGCTTCTTGGTAGGATGTTACAGGGGACAACGAGACTACCCTGGAACTTTTCACGCCATCCTGCAGCTTACTGGGACTTTTTTCACTGTCATGCTTCTTGGGCTCAAGTTTTGCTCTTGTCAGTCCCAGGGAATTTTGCATAGTTTTAGAGCAGGACTTCAATTGCTGGAAGACCGCATTTATCGGTCCTCTTTGGGTTGGTTTGCTTATGAGCCTGAGTGGTATGATCTAGACAATGCAAATTTTGCCCAGAGTGAGGCTCAATCTATATCCTTATTTGCTCATTATCTTTTGAACGAGCGAGGGGATGCTCATCAGACAGATTCAAAAGGACGTGGGCGTGAAAACGGGAGCCCTTTGGGTGATGTGATGGATCAATACCACCCTGTTTGGGGCGCAAATGGAGAACTATGCGGCTGGAAGAGAAAAACGAAAGCAGTTACTTTTGATGCTATGTCAGCACGAGGCTGATAGACTTGAAGTTTGGGCACAGCCCCACCAACTCAAAAGAAAGTACATCTTTTCGGCCTAAATATAGCTCAGAAAAATGGATTGAATATGCCCGGATTGCCTTCTCTGTGGATCCTCGGATTGCTTTATCCGTGGCCTCAAGGTTCCCGACAATCCCTTCTCTAAAGGCTGAAGTGACTCAGCTGGTCCAGTTGCATATATTGGAGATCCGCTGCATACCTGAAGCCTTGCCATATTTTGTGACTCCAAAGGCAGTTGATGAAAATTCATCACTTTTGCAACAATTGCCACACTGGGCTGCATGTTCAATCACACAGGCACTTGAATTTCTTACTCCTGCATACAAGGGGCATCCAACGTGTTATGGCTTATGTTCTTAGAGTCTTGGAGTCCTATCCTCCAGAACGAGTAACCTTTTTCATGCCACAGCTAGTTCAGGCTCTTCGATACGATGAAGAGAAGTTGGTAGAAGGATACTTGCTTAGAGCTGCTCAAAGAAGTGATATATTTGCCCATATTCTCATATGGCATTTGCAGGGGCGAAACATGTGTACCAGAAACGGGGAAAGATGCAGGCTCTGGAAAGAATAGCTCATTTCAAGCACTATTGCCAGTTGTCAGGCAGCGAATTGTTGATGAATTCACTCCTAAGGCCCTTGATTTGTTTAATAGAGAATTTGACTTCTTTGACAAAGTTACGTCTATTTCTGGTGTGCTCTTTCCACTTCCAAAGGAAGAACGCCGGGCTGGCATTCGGAGGGAGTTGGAGAAAATTGAGATGGATGGAGATGACCTTTATCTACCAACTGCTACTACTAAATTAGTCCGGGGTATTCAAGTGGATAGCGGAATACCCTTACAATCAGCAGCAAAAGTTCCAATCATGATCACTTTCAACGTCGTAGATCGCAATGGGGATCGAAACGATCTAAGACCCCAAGCTTGCATTTTTAAAGTTGGAGACGATTGTCGACAAGATGTTCTTGCTTTACAAGTGATTTCATTACTTAGGGACATTATTTGAAGCAGTTGGACTTAATCTTTATGTATTTCCTTATGGTGTTCTCCCAACTGGTCCAGAGCGAGGAATAATTGAGGTTGTGCCTAATACACGGAGCAGAAGTCAGATGGGGTGAAACCACTGATGGTGGTTTGTACGAGATTTTCCAACAGGATTACGGCCCAGTTGGATCTCCTAGTTTCGAAGCTGCTCGTGAGAATTTCATCATTAGTAGTGCTGGTTATGCAGTTGCCAGTCTTCTACTTCAACCAAAGGATAGACACAATGGGAATCTTCTCTTTGACAATGTAGGGAGGCTTGTACATATTGATTTCGGGTTCATCCTGGAAACTTCACCCGGTGGAAATATGCGATTTGAAAGTGCACACTTTAAGCTGAGTCATGAGATGACTCAATTACTTGACCCATCTGGTGTGATGAAAAGTGAAACGTGGTACCAGTTTGTAGGGTTGTGTGTGAAAGGGTACTTAGCGGCGAGACGCTATATGGATGGGATTATTAACACGGTGTTAATGATGGTAGACAGTGGGCTACCTTGTTTTAGTAGAGGAGACCCAATTGGAAATCTACGGAAGAGATTTCATCCAGAAATGACAGAACGCGAGGCGGCTAATTTTATGATCCGAACGTGTACAGATGCATATAACAAGTGGACTACGGCTGGTTATGACTTGATTCAGTATCTGCAGCAGGGAATCGAGAAGTAAATGAGATTTGTTTATTCAGAGGTTGTCCTCTTCAAATGTACATTTTATTTATTAGCTATATATATATTTTTTTTGTCCGAGGGATGTTTGCTTCCATGGTGGCAGTATTGAGATAGGGTTTGTTAAAACGGCAGATGAGTTTTTTTTTTTTTTTGGCAACCTAGTGGTTGGATCCCTCGTTATGTCTGTATAGTGAATAGACAAAAAGCAAGATTCTTTTATTTTTTCTTTCTTAATAGATTATTTCAAAAAAAAAAAAAAAAAAAAAAAAAAAAAAAGT

>transcript_45 full_length_coverage=4;length=6643

GGGGAACACATTATCTATTTTGCTGTGAAAGATTATCTAGAGAAGTAGACAGGAGAAAGTATAGCTAGAAAGAGAACATTCATGTTATATTGATAACTGATGAGGGACTCATTCTCCGGTTAGGGTTTGGAATCCTGTGTAGGAACTTGTGAACACTACTGTAACCCTGGAGAGCGAAAGCTCTCGATCCAGATACTGTTGTTGCACCGATTGGCTAGGTTATTTGTCTGGTATGGAGATCGATTCCCCTTCTCACTCCGATTTTCTATCGCCTCGTGAGCGAATCGTACAGAGGCTTGTTCAACAAGGAGTTCCCAAAGAGCTGCTTGATCGCCTCCAGCGTGGTTTGATTGCTTTTGTCAAACAGAACAGGCTTCGAATACCAGAGCTCGTATCTGCCATTTTACCAACTGATGAGGAGGTAGCGGAGGCTTTATCAAAAGCTAAAGCAGTGAGCAAAGCCGCCAAAGCTTTTGGTAGTCTAAGCCTGAAAAAAAGGTTCCGTGAAAGCATGGTTTGGTTACAGTGGTTGATGTTTGAGGGTGACCCAGGGGTTGCCCTGGATGCCCTTGCTAAAATGAGTGTTGGTCAGCGTGGTGTTTGTGGGGCTGTTTGGGGAAACAATGATATAGCGTACCGCTGCCGAACATGTGAGTACGACCCAACATGTGCAATCTGTGTTCCTTGCTTCAAGAATGGGAACCACAAGGACCATGATTATTCTATTATTTACACAGGTGGTGGTTGTTGTGATTGCGGGGATGTAACAGCATGGAAACGCGATGGCTTTTGTTCAAAGCATAAAGGTGCAGAGCAGATACAACCCCTGCCAGAGGAGTTTGCAAACTCCGTGGGGCCTGTCCTCGATTCCCTTTTGGTTTGTTGGAAAAACAAGCTATTGTCTGCAGAAGCTGTATATCTGGAAAACCCTAGAACAAGTGATCATGTTGCAGAACTCAGAAAGATTGCAAATGAACTGACATTTGTTGTGGTTGAGATGCTTCTAGAGTTATGCAAGCACAGTGAGAGTTTGCTCAGTTTTGTTGCTAAGAAGGTTATTTCTTTAAGTGGTTTATTGGATATTCTGGTGAGGGCAGAGAGGTTTTTAGGTGAAGTTGTGAGGAAGCTCCATGAATTGCTCCTAAAGCTTCTGGCAGAACCTGTGTTTAAGTATGAGTTTGCTAAATCATTCCTGAATTATTATCCGGTTGTTATAGAAGAAGCCATAAGAAAGAGTAGTGAAACTGTTTATAAGAAGTATCCACTCCTTTCCACATTCTCTGTGCAAATATTCACAGTGCCAACTCTAACCCCCCGTCTTGTCAGGGAGGTGAATCTATTGGCTATGCTCCTGGGGTGTCTTGGAGATATTTTCATTTCTTGTGCCGGAGAAGATGGTCGTTTACAGGTTACTAAGTGGAGCAATCTGTTTGAACCCACTGTCCGTGTGATTGAAGATATTCGATTTGTTATGAGTCATGTTCAAGTACCCAAATATGTAACTCAAGAACAGCGAGATATCTCAAGAACTTGGATAAGACTCCTGGCTTTTGTGCAAGGGATGAACCCTCAAAAAAGAGAGACAGGCCTCCATATAGAAGAAGAAAATGAGAATTTTCATTTTCCCTTTGCTTTGGGACACTCAATTACAAACATTAATGCTCTCTTGGTGACTGGAGCATGTTCTGTAGCTAATGCTGAAGAGACACAAGACGGGACTCTCTTGAACACAAAAATTGATGATTTGGATGATGATGATGGCATACGACATGCAAAAGTTGGACGGATTTCCCAGGAATCCTCTGTGTGTAGTGCGGCAGGGAGGAATAATTCATTGGCTTGTGCCTCGAAAGTTTCTGAAGTAGAGTCACATAATTTAGTTCATGGTGTAATTCCTTCCTCTGTCACATGTTTAATATATGAGTGCTTGAGGGCAATTGAGAATTGTTTGGGTTTGGATAATACATCAGGGGCTCTTCCAACCACTTCTAATATCTCTGGAAGTAATTTTATGACATTCAAGAAAACACTATCTAAGATAAAAGAGGGGAAATATATCTTTAGTAAACATTCCAGTTCAAGTGAAGTTCTTGAAAGGCCACTTTCTTCACCTGTTTATAACCATTCAAGTTCTGACTTGATATTTAGAAGACAGGATAGTAAACCAATGATCACTTGTGAAGGCATGGATAATATGCATGATACTGCAGTCCTTGATGATAGTACCATTGAAGGAGAATGTGCCACAGAATTAGAAGCCTTACGTGTGTTGAGTTTGTCAGATTGGCCAGATATAATTTATGATGTTAGTTCGCAAGATATTTCTCTTCACATTCCCTTGCATCGATTACTTTCCCTGCTTTTACAGAAAGCATTGAGAAGATTTTATGATGAATCTGCAATGCCAAATGTGGTGAATGCAAGTTCTACTAATCCATGGTCGGCAATTTATTCTGACTTCTTTGGACATGTCCTAGGAGGCTGCCACCCGTATGGGTTTTCGGCTTTTGTTATGGAGCATCCTTTACGGATTAGGGCATTTTGTGCTGAGGTTCATGCTGGAATGTGGCGGAAGAATGGGGATGTTCCTCTATATTCTTGTGAGTGGTACAGTTCAGTACGCGGGGCTGAGCAGGGTTTAGAGCTTGATCTCTTCCTACTGCAGTGCTGTGCTGCGTTAGCTCCAGCAGACCTTTATGTTAATAGAATTATAGAACGCTTTGGGTTATCCAGCTACCTTACTCTGAATCTTGAACGGCCTAGTGAGTATGAACCAGTTTTGGTCGAGGAAATGCTCACTCTTATCATCCAAATAGTTAAAGAACGAAGGTTTTGTGGGCTAACAACAGCTGAAAGTTTAAAAAGGGAGTTGATTTATAGGTTAGCTGTTGGAGATGCGACTCACAGCCAATTGATGAAATCTCTCCCTCGCGACCTTTCCAAATTTGAGCATCTTCAGGAAATTTTGGATACTGTTGCAGTATATTCCAATCCATCGGGCTTAAATCAGGGTATGTACTCACTGCGATGGACGTTTTGGAAAGAACTGGATTTATATCACCCTCGTTGGAACCCAAAGGAGTTACAAGGTGCAGAAGAAAGATACATGCGTTTTTGTAGTGTTTCTGCATTGACCACTCAACTCCCTAGGTGGACTAAGATATATCATTCTCTCAACGGAGTAGCTAGAATTGCTACTTGCAAAATGGTTCTTCAAATTGTCCGTGCAGTGTTATTTTATGCTGTTTTCACTGATAAATCAACCACATCCCGTGCTCCTGATTGGGTGCTTCTCACCGCATTGCATTTGCTCTCATTAGCATTAGACATATGTTCTGTGCAAAAGGAATCTGGCACTCAGTTATGCTATGTTGGGGATTTGATTCCCATGCTAGCTTTTGCTGGTGAAGCTATTGGTGGGGGAAGAAATGACAGATGTGGTGAACAGAGCTTGTTATCTCTTCTTGTTTCATTAATGAGGATGCATAAGAAAGAAAATTTATACAATTCTATGGAAGCAGGCAATTGTGACATTTCTTCCTTGATTGAAAGTTTATTAAAAAAGTTTGCTGAGATTGACTCTGGATGCATGACCACACTTCAAAAACTTGCCCCTGAAGTGGTCAACCACTTATCACAATCCATTCTGAACAGTGATAGTATGTTGGGGTCATCTTCTGACAATGACAAACGGAAGGCAAAAGCTCGAGAGAGACAGGCTGCAATACTGGAAAAAATGAAAACCGAGCAGTCCAAATTTTTGGCAAGCATCACTCCCACTTCAGATGACGAGTTAAATGGTTCAAAACCTGTACAAGAAGTACACAGTTCTGATGTTGGAGAAGAGTCTGCACAAGAAGTTTGCTGTCTTTGCCACGACCCCAGTTCTAAAAATCCTTTATCTTTTTTAGTTCTTCTTCAGAAATCCAGGCTTGTGAGTTTTGTCAATAAAGGCCCTCCATCATGGGAACAAGTTTGCCAGTCAGGTAAAGAGCAATCAACTGCCTCAAGTAACACTTACTCAAGTGGTTCGGAAATGGTTTCAACTTCCCAATTGATGCAGTCTTCAGAAATAGTTTCATCTTCCCAATTGGTGCAGTTGGTTCAGAAAGCTGTTAATGAGTTCTCCTTTTATGCGCGATGCGGGGACGTCAATGCTTTTTTAGAATTCATTAAGACTCGCTTTTCTTCAGTGAGAAGTATTCAACTGCCTCACACAACTGATGACACGTTGGACAAGGCAGTAAGTTCCTCTGAGATGTTGGAAGAAGATTTGTACAATTCCATCCTGAAAGAAACACATGATAGTTTGCCGCATCCAAAATTCTATTCAGACTCTCCCTTACTGGGGAAATATATAGCTGTTCTTTCAAGAGAGAGGGTAGACAATCCTTCAGCTTCTGAGAATGACCATTCACGTAATAGCAAGGGTCCATCAGAATCTATACCACAGCTTTCCACATACGATGGATTTAGCCCCTCAGACTGTGATGGGATTCATCTTTCGTCCTGTGGGCATGCTGTGCATCGGGGATGTCTTGAACGTTATTTATCTTCCCTGAAGGAGAGATATATTAGAAGAATTGCTTTTGAAGGAGGGCATATTGTGGATCCAGATCAGGGAGAGTTCCTCTGTCCTGTATGCCGCCGACTTTCGAATTCTGTCTTGCCTGCATTACCTGGACATACTCAAAAGGTCTTGGGGCAGCCAATGATTTCAACTGTTGATTTGCCACATGCTGAAGGCCCATCAACATCAAGCGAGAAAATTGATTTCCTTCACCTTCGTCATGCTCTGACTCTAATTGAAACTGCAGGAAGCATGGTTGTGAAGGGTGAAATCCTCAGAGCTTTACCCATGCGGAGAAATGGAAGAACAAGACCAAATCTTGAACCTCTCGTTGATATGCTTTCTGGAATGTATTTTTCAGGCAAAAAGGATAAGTTATCAGGATCTGCGAGGGTAAGTGATTCATTAATTATGTGGGACACTCTTAAGTACTCCCTCATATCAACAGAAATTGCTGCTCGTAGTGGAAGGACTTCTATGACTCCAAGATATGGCCTTGATTCTTTATACAAGGAGCTTAAATCTTCTGGTGGATTTATTTTATCCTTGCTGCTTAAAGTTATCCAAAGCATGCGGAGCAAAAATCCTCTTCATGTGCTTTTAAGATTTAGAGGCATTCAGCTCTTTGCAGAATCAATTTACCCTCGTGTTATTGGAGAAGAACTTATCAGCAGCACGTGCAGACAAGGAGGTTACATGTCAAGTATCTTGAAACATATAGAAACGGATATACCCTATCAAGACATTCAATTTTGGAAACGAGCTTCTGATCCTCTTCTTGCTCGCGACCCCTTTTCATCATTAATGTGGGTTCTATTTTGCCTTCCATCTCCATTCCTATCTTGCGATGAGTCATTTTTATGTCTTGTGCATCTCTTCTATCTAGTCTCGGTTACTCAGGCAATAATCACATATTGTGGAAAATTCCAATCAAGTGTTGGTGAATTAGGTTCACACGGTTGCCTGATTTCCGACATATCAAACTTCATGGGACAATCTGGATTTCCTATGGATTATTTTGTTTCCAACTATTTTACTGATTCTTGTTATAACTTAAACGACATGATTCGTAGCTTCACTTTCCCTTTCTTGAGAAGGTGTGCATTGCTCTCCAAACTATTGAGTTCTACCGCGTCAACACCTCATGGATTTGACGCATTGTGTCCTGATGACATTGATGATATGATGGATACTTGTAATGCTAATGACGCCGAGGTGGAGCTTATTGAGGTTGGGAAGCTGGAGAACATGTTTAATATTCCCCAACTGGCCGATGTTGTTAAGGACGAAATGACTCGAGGCGTAGTATTGAAATGGTTGCATCATTTTCGAAGTGGGTTTCAAGTTGCTGGCCGTGAATTTGTTTTGTATTCCACTCCAGCAGTTCCCTTTAAGTTGATGAATTTGCCTCATGTTTACCATGAGATCTTGCAGAGGCACATAAAACAACGTTGTCCTGAATGCAAACTTGTTATGGATGATCCCGCGTTATGTTTGTTGTGCGGTAGATTATGTTGTCCTACCTGGAAGTCGTGCTGCAGGGAATACAGTTGTCAAACTCATGCAACTGCATGTGGTGCTGGTGTAGGAGTATTTTTGTTAGTCAGGAAAACTACAATACTGCTTCAAAGATCTAAACGTCAGGCACTTTGGCCTTCTCCTTACTTGGATGCATTTGGGGAAGAGGATATTGAGATGTATAGGGGAAAACCACTGTATTTAAATGAGGAACGATATGCCGCGCTAAGTTACATGGTGGCTTCTCATGGTCTTGATCGGAGTACCAAAGTTCTCGGTAAAACAACAATTGGGACTTTCTTTATGATATAGAAATAGAATCTAAAGAGAGAGTTTTCCCCTCGTTATATATATTATTCATCATTTTGTTTTTTTTTCTTTTTTTTTTTCCCCCTTGGGTTGGCTGGGAAATAAGGTTGTACAGGAGTGTTTGTTTTAAAGAAGATATTATGTATAGCAGTTTTTCATTATGATGCAAATAATGCATTAAAATGCATAGTTGAATCAATCCAGCTTTTCTTCAAAAAAAAAAAAAAAAAAAAAAAAAAAAAAAAA

>transcript_46 full_length_coverage=4;length=6646

GGGGTGCCCCCACAAAACCAAAGACGAACTTTCTCCATAAAGAAAACAAAATGCTAAAAATCAGATGAACAAGACACAAACAATCACAATCTTTTTTATGCATTATGAAGCACATAAATCATCTCTCTGAATCACAACATTTCATTCTCTCTCGTCTTTTTTACTCTACATTTCTCTGTTTCTCCACCATGAAAGTTTCCTTGCTGTAAAGCATATCTCATACTTCTGTGTATATACATTTTTCTCTCTCTCGACCAGGCATTGCACCGCCATGGAAGCGCTGATCGAGCTCTGCGACCTGATCGCACAGAAGCCCACACAATTCGCCGATAAACTCGCATGGATTTGCAGCAGATGCCCTCCTCCCGAGTTGCTCCTCTCTGGATCGCCTAGGGTTTCGAGGTCTCAGCTCAATGCTGTTCTCGTCATTGCCAGATTTCTTTCCAAATGCTCTGACGCGTCCGACTACCGCCCCAAAGCAGCTGTACTCGAGTTTATCCGCTCAATTCCGGCGTCATTCAATCATTCTTTCTGGCCGCAATCGTACGGTACGGATTCCATTTCGTCCTTCTTCGTAGATTTCTTGGGGTACGTATCCAAGGCGACCGAGTTGTCGTCGGATTTTTCCACGGAGGTTTCCGGGTTCATGGGCGAGATCGTCATCTCTGCTATCAGTAATACCGGTGGGAATACCGTAATTTCTAGGGTTTTTCTGGCAGCCCTTTCGCAGAACTTTCCACCAGTTTTGCCTTCTGACGCAGATAAGTTGGTCAATTTTCTTTTAGATCAGTACGTAATTGCGGTCCCGAGCTCGCCGAGAGAGCCAGTGCCTGCGGTGGCGACGTCAGAGGCGTCATCGGCGCAGAGTTCACCGCTTAACGTAAACCAAAACGAGTCTTCGAGTTCGGGAAATGAAGCTAGCAGTGCTTCCAAAATTGCGGATGATGCCACAAGTGCGTCTTCTGGGGCATCTGTGATGATGAATGGGGGAAATTTGATGTGGAAGAGTGGCACGGGGGAGCATTATGGTGTGAATTTCGGGTACAATGATGGAGGGGGAGGAAACGCAATGTTTAGGCAACAAGTTGCTGCATTTGAGGCGGAGTCCGTGGAGAGTTTGGAGAAACAAGAGATCGCATTCAAATTGATTGGGCACATTTTGGATAAAGTCCACATTGATCCTAAGCTCTCAGAGCAGGTTAGACTTATTGCTAAGAAGCAGCTACAATCCATGTCGGCTTTTTTGAAGATAAGGAAGCGAGATTGGACAGAACAGGGCCCGCTGCTAAAAGTTCGAATCAATACAAAACTATCCGTTTACAAAGCTGCAGCTAGGTTGAAACTTAAGAGTCTAGCCTCTCTCGATTCAGATGGGAAATCATCCAAGAGGTTGCTCCTCGAGACTCTTGCCTTGTTGATAGACGCTGCTGAAGCATGTTTACTCTCTGTGTGGCGAAAGTTCAAAATTAGTGAGGAGCTATTTGCTTCCTTGCTTGCCGGAATTGCACAAATTGCTGTTACACGTGGAGGTCAGCTGTTGCGGGTTCTGCTCATTCGCCTCAAACCCCTTGTGCTTACTACATGTGCACAGGCTGATAACTGGGGTGCGATGTTCGAGAGTGTGATGAAGACTAGTTGTGAGATAATTGAATTTGGCTGGAGTAAGGATCGAGCTCCTGTAGACACTTTTATTATGGCATTGGCCACGAGTATACGTGAACGCAATGATTATGAGGAAGAGGTTCAGGATGGAAAGGAGAAAGCAGTTCTTAATGTGCAGCTTAACGTAATACGCTTGCTAGCTGATTTGAATGTTTCTGCTAACAAGTCTGAAGTGGTTGACATGATATTGCCATTATTTATTGAAAGTTTAGAGGAGGGTGATGCTTCAACTCCTGGTCTATTGCGACTCCGACTTCTTGATGCTGTGTCTCGCATGGCAAGTTTAGGTTTTGAGAAGTCCTATCGTGAGACAGTTGTTCTGATGACAAGAAGCTACTTGGGTAAACTATCAAGCATAGGATCTGCTGAAAACAAAACATTGGATGAAGAAGCCACCAAGGAACGTATTGAGACTCTTCCTGCAGGGTTTCGTTTGATTGCTAGTGGGCTTACTGGTACAAAATTGCGGTCAGACTATCGTCATCGATTGCTATCTTTATGCTCAGATGTAGGATTGGCTGCCGAGTCAAAAAGTGGGAGGAGTGGAGCAGATTTTCTGGGACCTTTACTTCCTGCTGTTGCGGAAATTTGTTCTGATTTTGATCCTACTATGGATGTGGAACCTTCACTCTTAAAGTTATTTCGCAACTTGTGGTTCTATATTGCTCTTTTTGGTCTGGCACCTCCCATACAGAAAAATCAGGCTCCAGCAAAGTCTGTTTCTACTACATTGAATAGTGTAGGAAGCATGAGTTCTGTTGCTCTTCAAGCTGTGGGTGGACCATACATGTGGGATATTCAGTGGTCTTCTGCTGTTCAGCGCATTGCTCTAGGGACCCCACCCCTTGTTGTAAGCTCTGTAAAATGGCTTGAAGATGAGTTGGAACTCAATGCTCTTCACAACCCTGACAGTCGTAGAGGGAGTGGCAACGAGAAAGCTGCTGTAACCCAAAGAGCTGCTCTTTCTGCTGCATTAGGTGGGCGAGTTGAGGTTTCAGCAATGAGCACAATTTCAGGAGTTAAAGCTACCTATCTCCTTGCGGTAGCTTTTCTGGAGATAATACGTTTCAGCAGTAACGGTGGCATCCTTAATGGTGGCCCTAATTTGACTGCTTCTAGAAGCTCCTTCAGCTGTGTTTTTGAATACCTCAAAACCCCTAGTCTCATGCCATCTGTCTTTCAGTGTTTAATGGCAATTGTTCACAGGGCATTCGAAACAGCAGTGTTGTGGCTGGATGATCGGACATCCGAAACAGGCAATGAAGGCGAAATTAGAGAGTCTACTCTTTCTGCCCATGCATGTTTTCTCATAAAAAGTATGTCCCAGAGGGAGGAACACATTCGGGATCTCTCTGTCAACTTGTTGACCCAACTTAGAGACAGGTTTCCACAGCTTTTATGGAACTCTTCTTGTGGGGATTCCCTGCTATTTTCAGTTCATAATGAGACTCCTACTCTTGTCACTGATCCTGCTTGGGTTGCGACCGTTCGTTCTTTGTACCAAAAGGTTGTTCGGGAATGGATTATTTGTTCACTTTCATATGCTCCATGCACCAGTCAGGGTCTTCTTCAGGAAAAGCTCTGTAAAGCAAACACATGGCAGAGAGCGCAGCACACAACTGATGTTGTTTCTCTTTTATCTGAGATTCGGATTGGCACGGGTAAGAATGATTGTTGGACAGGCACACGGACAGCAAATATTCCTGCAGTTATGGCTGCTGCGGCTGCGGCATCAGGTGCAAATCTGAAATTGATGGAAGCCTTCAACTTGGAGGTGCTGAGTACAGGAATAGTCAGTGCAACAGTAAAGTGCAACCATGCTGGAGAGATTGCTGGCATGAGAAGGTTGTACGAGAGCATGGGTGGATTCCCTGCTCAAACAGGCTTTGGGATTGGTCTTGGCCTTCAAAGGTTGAGATCCGGAGTGTCTCCTCAGCAACCACAGCCCGAGAATGATGCATTTAGTGGACTTTTGCTTACCAGATTTGTGAATCTACTGCAACAATTTGTTAACACCGCAGAGAAAGGTGGGGAAGTGGACAAAACATCCTTTCGCGAAACTTGTTCTCAGGCAGCTGCGCTACTTCTCTCAAATCTGGATTCTGATTCAAAATCAAATATAGAGGGATTCTCACAACTCTTACGTCTTCTTTGCTGGTGTCCTGCTTACATTTCCACCCCCGACGTGATGGAAACTGGTGTATTCATTTGGACTTGGTTAGTTTCTGCTGCACCTCAGCTGGGGTCTCTTGTCCTTGCTGAGCTTGTTGATGCATGGTTATGGACCATTGATACAAAAAGAGGTCTCTTTGCATCTGAAGTAAGGTATTCTGGACCTGCTGCAAAATTAAGGCCTCACCTTGTCCCAGGGGAGCCAGAAGTGCAGCCTGAAACAGATCCCGTTGAACAAATAATGGCTCACAGACTATGGCTTGGATTTTTCATTGATCGCTTTGAGGTAGTCCGATACAACAGTGTTGAACAACTCTTGCTTCTTGGTAGGATGTTACAGGGGACAACGAGACTACCCTGGAACTTTTCACGCCATCCTGCAGCTACTGGGACTTTTTTCACTGTCATGCTTCTTGGGCTCAAGTTTTGCTCTTGTCAGTCCCAGGGAATTTTGCATAGTTTTAGAGCAGGACTTCAATTGCTGGAAGACCGCATTTATCGGTCCTCTTTGGGTTGGTTTGCTTATGAGCCTGAGTGGTATGATCTAGACAATGCAAATTTTGCCCAGAGTGAGGCTCAATCTATATCCTTATTTGCTCATTATCTTTTGAACGAGCGAGGGGATGCTCATCAGACAGATTCAAAAGGACGTGGGCGTGAAAACGGGAGCCCTTTGGGTGATGTGCAGATGGATCAATACCACCCTGTTTGGGGCCAAATGGAGAACTATGCGGCTGGAAGAGAAAAACGAAAGCAGTTACTTTTGATGCTATGTCAGCACGAGGCTGATAGACTTGAAGTTTGGGCACAGCCCACCAACTCAAAAGAAAGTACATCTTTTCGGCCTAAATATAGCTCAGAAAAATGGATTGAATATGCCCGGATTGCCTTCTCTGTGGATCCTCGGATTGCTTTATCCGTGGCCTCAAGGTTCCCGACAATCCCTTCTCTAAAGGCTGAAGTGACTCAGCTGGTCCAGTTGCATATATTGGAGATCCGCTGCATACCTGAAGCCTTGCCATATTTTGTGACTCCAAAGGCAGTTGATGAAAATTCATCACTTTTGCAACAATTGCCACACTGGGCTGCATGTTCAATCACACAGGCACTTGAATTTCTTACTCCTGCATACAAGGGGCATCCACGTGTTATGGCTTATGTTCTTAGAGTCTTGGAGTCCTATCCTCCAGAACGAGTAACCTTTTTCATGCCACAGCTAGTTCAGGCTCTTCGATACGATGAAGAGAAGTTGGTAGAAGGATACTTGCTTAGAGCTGCTCAAAGAAGTGATATATTTGCCCATATTCTCATATGGCATTTGCAGGGCGAAACATGTGTACCAGAAACGGGGAAAGATGCAGGCTCTGGAAAGAATAGCTCATTTCAAGCACTATTGCCAGTTGTCAGGCAGCGAATTGTTGATGAATTCACTCCTAAGGCCCTTGATTTGTTTAATAGAGAATTTGACTTCTTTGACAAAGTTACGTCTATTTCTGGTGTGCTCTTTCCACTTCCAAAGGAAGAACGCCGGGCTGGCATTCGGAGGGAGTTGGAGAAAATTGAGATGGATGGAGATGACCTTTATCTACCAACTGCTACTACTAAATTAGTCCGGGGTATTCAAGTGGATAGCGGAATACCCTTACAATCAGCAGCAAAAGTTCCAATCATGATCACTTTCAACGTCGTAGATCGCAATGGGGATCGAAACGATCTAAGACCCCAAGCTTGCATTTTTAAAGTTGGAGACGATTGTCGACAAGATGTTCTTGCTTTACAAGTGATTTCATTACTTAGGGACATATTTGAAGCAGTTGGACTTAATCTTTATGTATTTCCTTATGGTGTTCTCCCAACTGGTCCAGAGCGAGGAATAATTGAGGTTGTGCCTAATACACGGAGCAGAAGTCAGATGGGTGAAACCACTGATGGTGGTTTGTACGAGATTTTCCAACAGGATTACGGCCCAGTTGGATCTCCTAGTTTCGAAGCTGCTCGTGAGAATTTCATCATTAGTAGTGCTGGTTATGCAGTTGCCAGTCTTCTACTTCAACCAAAGGATAGACACAATGGGAATCTTCTCTTTGACAATGTAGGGAGGCTTGTACATATTGATTTCGGGTTCATCCTGGAAACTTCACCCGGTGGAAATATGCGATTTGAAAGTGCACACTTTAAGCTGAGTCATGAGATGACTCAATTACTTGACCCATCTGGTGTGATGAAAAGTGAAACGTGGTACCAGTTTGTAGGGTTGTGTGTGAAAGGGTACTTAGCGGCGAGACGCTATATGGATGGGATTATTAACACGGTGTTAATGATGGTAGACAGTGGGCTACCTTGTTTTAGTAGAGGAGACCCAATTGGAAATCTACGGAAGAGATTTCATCCAGAAATGACAGAACGCGAGGCGGCTAATTTTATGATCCGAACGTGTACAGATGCATATAACAAGTGGACTACGGCTGGTTATGACTTGATTCAGTATCTGCAGCAGGGAATCGAGAAGTAAATGAGATTTGTTTATTCAGAGGTTGTCCTCTTCAAATGTACATTTTATTTATTAGCTATATATATATTTTTTTTGTCCGAGGGATGTTTGCTTCCATGGTGGCAGTATTGAGATAGGGTTTGTTAAAACGGCAGATGAGTTTTTTTTTTTTTGGCAACCTAGTGGTTGGATCCCTCGTTATGTCTGTATAGTGAATAGACAAAAGCAAGATTCTTTTATTTTTTCTTTCTTAATAGATTATTTCAATAAAGGACTGTTTTGATTGAAAAAAAAAAAAAAAAAAAAAAAAAAAAAAAAAGT

>transcript_47 full_length_coverage=2;length=6613

GGGAGAAAAGGAAAAGAAAAGAAAACGCGGCTGCTGTGCACCTAAACCAGACTCTCTCTCTATCTCTCTCTTCTCTCTGTGTGCTGATCGATTGTCAGCTATAGAGATCGCATACACGATCCAGACCACTAGGGTCTCGGAATCAAATGATTCCATTCAAGGATCTTTGGCGTTTATCACTTTGATAAGAATCGTTTAATTTTGGGTATTGGTGATATCGACGTGGGTTTGTAATCGTGTCCGATCCTGATCGTTCTGGGAAATTCCTTGGTGGGTTTGGGTGGGGTTTGGAAGATATCAAATGAGTCGAATTTTATTCGTGGTTTGGATGCGGGGTCGTGGAGAGAAGGTCTGGGTTTGAGGGTTTGGAAGCGGACCTTTTTATTTATTTATTTATGATTTCTGTGGGGAGTAGAGATGGAAGGGAGGGGTTAGGGTTAGGGTTTGGTCTTGGGAGAGAGATGGAAGACACTGAGCTTGAGGAAGGCGAGGCCTGCTCTCACCAGAATGATGATGCGAACATCGACCCTGATGTTTATCTCTCGTATCTCGATGAGAAACTTTATAATGTTTTGGGACATTTTCAAAAAGATTTTGAAGGTGGGGTTTCAGCAGAAAATTTGGGGGCAAAATTTGGTGGGTATGGTTCATTTTTACCTACCTATCAGAGGTCTCCTGCTTGGTCTCAGCCAAAGACACCACTAAAAATTCAGAACCATAACACACCGAGATCCCCAAATGGTTTGCAACTGGAGGGCGGTCATCGTAACTCTGCAGTGTCGGCATGTGCACCTCTATCTGTGAGACAGGGTTCTGCTTCTACCTGCGCCGCGGCACTGTCTGGATCGAAGGCATCATCTGTTCATGATTCAGTCAAACAGAATCTATGCAAGCCATCTATTCCTGTTGGGGAATTTACTCCCAGATCTGAAATTGCAAATGTGCCTTCTAATCTACCAGACCAGAAAACACTGAAGGTTCGAATCAAAATGGGCTCTGATAACTTGTCGACACAAAAAAATGCTGCAATATACAGTGGTCTTGGTCTTGACGTCTCACCATCTTCCTCTTTCGAAAACAGCCCCATAGAAAGCGAAGGGTTGTCCCATGAGCCTCGAGATTCTCCAGATGAATCTCCTACTACCATTCTTCGGACAATGACATTCTTTCCTTTGCATGGGGGTCTACTGTTATCGCCTCTTCCTAATGGTTTAATTCATTTGACCAAAAAGGAAAAGCTTCCGAGAGACAATAGATCTTGCTTGGTCCCTAAGGGTGGACAAGGAAGTTCGGTCATGTTAATGGATGGGTCTTACTCTGTAAAGGATGGGAATTTGTTGGGAGACAAAAGAACAAGGGCATCAGAGAAAAAATGTTCTTCAGTAGAATTGAAGAATGGCAATAGTAAAGATGCTCGGAGCAGCATTGGCGTCTTACCGAAGAAGGAATTAGAGGTTGACACTTTGGCTTGTGAAGATTCTGCAAGAGGTACTGTTATGGCATCTGATATGACCAGGGAAAATAAGAGTGTAGCGAAGAACAGATTCTTCTCTGATCTAGCAAAAGAGGAACTGCTGGAGCCTATATTTGCCCAAGAGGTTACCCGGGTCGAAAAGTCCAATGAAAAGGCTGTTTCTGCTGGAAAGATTTGGGAAGATAAAAAGGAAAGCTCCGATAGTGACGTTTCAGTTAAAACAAGGAAAGATAGAAATTGCAAAGGAGAACAAAGTTACGAATCAATTAAATCTGATTCAATCCACTGGAGGGAAAAGAAAGCTTCGAACACTGAACTCATAGATCCCATGAAACAGAACAGTGAGCATAAAGCAACATCCCATGAACAGCATGGCATGAGATTGCCTCCTGGGGAGGAGCATCCATCTTATGTAGGGAAAAAGAAGTCTAAGGGAACTCGAAGTCATGGCACTCCAGCTGCAGAGATACAAAAAGAAAGCTTGCGGGTGGGTTCTTCGTCGTTGACCAAGAATAAGAAGAGCACTCCTGCCAAAAGTTCTCTGTCCAATGGTGAATTGGAAAACCTCAAATTACAAAAAGATGTGAGGAAGGCCAGAGATGCTTACAGAGATTTATTTGGGGACAATACATTGGAACAGGAAGAGAATGAATTGGATTCATTAGATATGCGGTCTGAAGGCAAGTTGAAAGACCCCGAGGTGGTTGAAAAAAGTACAGTTTCATTTAATAACATATCAAAGGAGAGAATGAGTGGTAAGAAAAATAATAGGCCATCGACATCCGAGCCATATTCAAAAGTGGCTTCAAATGAAGCTCCTCACAGTGGAAATGGGTCCATTCCTAGTGCAGCTCCTAATGCTGTAGTCATAGAAGACAATTGGGTCCAGTGTGACAAATGTCATAAATGGCGTCTTCTTCCAATTGGTACAAAGCTTGAAAACCTTCCTGAGAAGTGGCTGTGTAGCATGCTTTATTGGCTGCCTGGAATGAATCGGTGTAGTATTAGTGAAGATGAAACAACTGGGGCTCTAATTGCATCTTATCAAATTCCTGCTCCCGTGGGCCAAAATAATATGCAGTGTCTTTCTAATGGATTTACATCAGGAGTAACCCCAGCTGATGTTCGGAACCCTGGCTGGAACCACCAAAATACTCATTTTCATGGGCCTAGTGGTGGAAAAAAGAAACATGGATCGAAAGAAATATCAGATGCCACCAACCAAGATGGTCCGGCTCAGTTTGGAAGCTCGATAAAGAACTTACAGCCATCAGTGAGAAGTAAAAGTTTAACCGATATGGATCAGCCACCTCTAACAGATGAACTTGATTTTCCGCATTCGAGGAAATCTAGTGATTTAGCTGTGGAGAAACATAGACATAAGAAGAAAGAGAAGCATAAGCTACTGGATCACTATCCTGATGGAGGTGATAGGAAAAAGTCGAAGATGAAAAGCAAAAGGGAGACTGATCAGGAATCTTTTAGAGTTTCTAAGAAAACTAAAACAGGTGTGCATTGTAGCATTGAAGATTTCCAGTCCGACCCTGGTGGGGGCATCAGAAAAGAAGGTCCTAGCTTGAGTAGTAGTCTTCCCTCTAACATGGCAGGGAAGTGTCGGCCTAAATTAGATGATGTAAATGACAGCTTACAAGTATCTGTTAAAAAACCAAAAGACCGAGTTCAGATTCCTTTGGATGAATCCTTGGATAATAGAAAACATGATAATGACATAGTTCCTCAGAAGAGAAAAGCGAAAGACTGTCAGGACTCTCAAATTCACTTGGGGTGCATTCCAGGCGAACATCTTCAGGATGGCAGGGTTTCTGTGGAGGAGTTTAGTGAGAATGACCACAGGAAGGAAAAAAAGGCAAAGCTGTCCAATTTTGAGGGGAAAGAGTATAGTTTATGTAAAAGCGATGGTGGAACAGATAAAAAAGGTGATACTGCGAAGGAGCAGCAGTTGGGGAAAGATTTAGGGAGTGTATTGTCTCAACGGAGCTCTGATGGTATGGATTTTTTGAAAAGGGATTTAGGGTCTCTACAGCCTTCGGTGCCAACCACTTCAAGCTCTTCTAAGGTCTCCAGTTCTTATAAAACCAGAACCTTCCAGGAAGTGAAAGGCTCTCCAGTGGAATCGGTGTCTTCGTCGCCCCTGAGAATTTTACATCCTGATAAGCTTTCATCAGCAGGAGGTAGCCTTGTGGGAAAAGATGGTTCACAAGATGGTGGTTTTCTTACACAAGGTAGTCCAAGAAGATGCTCAGATGGTGAAGATGATGGTGGGAGTGATCGATCTGCGACTGCAAGGAAGAATAAATCTTACATTGGCAATCATCGCGGGTCTCTTGAGTCGTCTGTGCTTAATTTTCAGGACACGGATTTGAGTCAATTGGTGGGTAGTAAAGCTAAAGAGCAGGCTGTTGCTTCTCGTGATTTTACAAATCACCCTTTTAATAATGGCAGTGCTGACACCTTAGCCCCAGGTACTCAATATCCTAGTGAACCACATGTTTCTGACCAGTGTCGCGAAGTAGAAAGAAGAAATGACAATCATTATCATGCTAATGGATTTCATCCCAGGAAATCTGGGAAGGGGTCCTCTTCACGATCGAAGGACAAGAATCGCAATTCTAAATCTGAATATGACAAGGGTAAGATTTCTCATTCTTATAATGAAACTCAAGAACATGAGCACCACGATCAAAAAGTGAGGGACAGAAAAAACAAGATGCAAGAGAAGTCTGGGGTTAACTCTGACACTGCAGAGAAGAAGTATGCTGATAAGAGAGAGTCTGCAGGAAAGTCGTCAAGTAAGAGTAGTAAAAGGGAAAGTCAGTCAAAATTTGGAGGGCGCGATGGTCCAGATCTTAAAGTCGATGTTATTAGCAATCACAACACAATGTCTACTCCAAAGCAAAAATTGCTGCAGGAGCATGATGGAGAACGATCTTCAAAAAGATTTATTTCTGAAAAAATTGACCGAGTGGAAATAGTTTCTGGAAGAGGTAAGTCTCTACCATTACCACCCTCTGGAGGAGGCCAAATTGAGCCCCTGCCTCGCACTCGAGTGGTTCCTGGATCTCATAAAGGTAATGGAGCAGTTGTTTTATCCGTTGGTTCTGCTGAAGGTGATGATGTGAAGGTTTCGAAACATATTAGGAAGGCCGATAACCAGAATGGAAGTCAGCATGTGAGTTCAAGACATTCCACACCCAATGGGCATAGGATTAGTGATCTTAATGCCCCGAGTCCTGTGAGAAGGGATTCTTCAAGTCTGGCTGCTACGAATGCTTTGAAAGAAGCCAAAAGTCTTAAACACATGGCTGATCGTCTTAAGAATTGTGAGTCGAATCTCGAGAGTATAGGGCTTTACTTTCAGGCAGCATTAAAGTTTCTTCACGGAGCCTCTCTTTTAGAATCTTGCAACAGTGAGAGTGCCAAACAAGGAGAGTTGATTCAATCAATGCAAATTTATAGTAGCACTGCAAAACTCTGCGAGTTTTGTGCGCATGAATACGAGAAATCCAAAGACATGGCTGCCGCTGCTCTGGCCTACAAATGTATGGAAGTTGCATACATGAGGGTAATATATTCCTCACATAACAATGCAAGTAGAGATCGGCATGAATTGCAGACGGCTCTGCAAATGGTTCCACCAGGTGAGTCTCCTTCCTCCTCTGCTTCGGATATAGATAATTTAAACAACACGGCTATGGTAGATAAGGTTGCCATAGCCAAGGGTGTCAGCTCTCCTCAAGTCGCTGGAAACCATGTCATAGCTGCTCGAAACCGTCCCAACTTTGTGCGGCTGCTGAACTTTGCGCAGGATGTAAATTTTGCAATGGAAGCCTCAAGAAAATCACGGATTGCTTTTGCAGCTGCTAATGTAGGCTTGGAGGAGGCTCGACACAGGGATGTTATATCTTCCATTAGAAGGGCTCTTGATTTTAATTTTCAGGATGTAGAGGGATTATTGCGTCTGGTGAAGCTTGCAATGGATGCCATCAGCCATTAGAAAGTCCCATGTTAATGGAGGTTACATCATCCGTGGGAAGATGTATTCTTTCTTTTACCTTGTTTCCTTGGCTGTTGAGCCTTTTAAGGAGCAAAAGAAGCGAGGCTGACCTGGATCTCTCTGCTGCACACTATGTAATTATTCTGTCAGGAGACGGGTTAGCGCCAGCTTAGTGCCCTCTGTATATGTTGTACAATACAGATTTTGATCAATTCTTTATTAGGCTTCATCTACTTTCTTTCATGGATAAGGGCCTGGCTTTTGAGTCAAAATCTCAAGTTTGGTGTTGAGGATGTGTTTTGTGGTCCGTCAGGATTGTATTTGCAGGAAAAAAGATGTATAGAGATTGAAAGCCCATTCATAAACTATTGGGGAAAGCTGGACTCCAATACAAATTTTTGGCTATTCACAAAGGGTGTTTATGTTGTTGGTGTCACTGTATTGGTTATTTTTTCATTTTAGTTTTATTTAGACAAAACTGAAGGGTTGGAAATTTTGAGGAAATGCATGAACTTGGTGACCTCACTTGTACTGGCAGTGACTGCACTTTCTTTTATGCTTTAAGCATCATTCGTCGAGGGTAAAAAGATGTCATTCTTTGGTTGTCTTTCAAAGCTTCTGTAATTGCAGTAGGAACAATTTGATAGAGATGAGTAGGAAGAAGTTAGCAAAAACTATATCTATTGAAAATAAAACACACATGTTAACCTTTTTGTAGTTCCCATTGGATGGGGGTTTCTTGAGTAATTATCCTTTCAAAGTGGCTATCTATGTACATATACAGACGGTTGAACTAAACATCAAGAACAGTTAAAATATCGTTCTTTTTTGGAATGTCATAATGCACCATCTGCTTGGAGGTTTGTGGATCTTACAGGTTGATTATCGAAGCAGTTGCATGGCATCTAGCTAATAACATCTCTCCGTGGAGTAGTTGCTGCTTTTGTTTGTTTTTGTTGTTTAATTGAAAACGGGATGCCATTTGAAAAGAGAGATTTTGACCCATCTAGATAATTCCTGCTTTCACTGATCCCTGAAATGTGTATAGCAGCATCAATATTTTTTTGTCTTTATAATAAAAATTTGAAACCCAAAAAAAAAAAAAAAAAAAAAAAAAAAAAAGT

>transcript_50 full_length_coverage=2;length=6607

GGGGTGACGTTTTTGGCGATGCCACACCAAACACCCCCCAACCCAAAAATCATCATCTCCCACCGTTCGTCCATTGGATTTGGCCGTATCAGCACAACAGTCCCCCATCTTTTCTTCTCTCCAGGGCTTCGGTCCGCTTCTCTTTATTCTCATCGGATCTGAGTCCGCCCCGCATAATTTGCATCATTAATTACTCTCATTCTATATCCGACCGGCCGATCCCTTCACTTTATCCGATCCTCACCCCATCGGATCTGAGTTCCAACCAAAAGCCCAACGTATAACTCGTTATAATATAAATTCTCAAAGTTGTAGGGCTGCGACTGATATAATTCGAAAGCCTTCATAAAGTAGTTGTAGTGGTGCGACTGATTCGAAACTGCCCGCTAGTATATACATTGCAACGGTGAGTTATTTTGATCTCATGAATGAGGCCAAATTCAAGTGAGCATGAAATATGGTCATATCTGCATTTGCTCAACGGTTCAACCGTTTTCCAAATCTCTCAGACGTTGGTATGTGGAATTGCAACATCAGAGAAGCTGTAAGCCAAGGCCACGCACAGAAGGCCCTCCTTCTCTTCCGCCAAATGAAACAAAAGGGAATTCTTAACATATCAATGAGTGTTTCTGTCTGTAATTGGTCTTGTTGGAAATTGGGTCTGGATATCATTGAGGAGACCTTGTTATCGAATTGCTGAATTTCGCAGATATCAGGCGTTCTTACCAATCGTCAGTGTCGTTGATGTTGACTATGTATATTCAAGCTGGAAAATCATTCCGATCATGATAGATGGACGAATCCAAATACTGAATTGGCATGATCCGTCTCTCAGATCGAAAAACTCAAAACACGAAGGTTGTAGCTCTCGTCGAGAGCTTTCCAACGATACCTAGATCGTCGAAATCCGACCACCGTAGCCACCAGAAAAGGCGGTCAAAGTTTGACGGGCGTTAAACTTCCGACGGCTTCGACGCCACTCTCCGGCCAGATTCCGGCCACCGGACCACCGCGGTTTTGAAGATCTCGTCAAGATCTTTCCAGGGAGTACTTGTACGCTCAAAACGGAGTTCGTATGCAACGGGAAATCGTGGTCAAAGTTTTTGGCCCGACGGTATGATTTCAAATCCGAATTTAATGTTATTTTGTTTTCTAATTTTGTTTTTCCGTGCTTCGCAGGCTTGTACCTTAAGGCGTACAACGCGCTCTAAAGCGCTAAAACTTCGGAAATTAATATAAAATCCAAAACAGATCAGAATTGAGCGCCGTTTTTTTTCATGGGTTCGTATCGACGAACCCTAACCAATTTTACAATAAAAACCCAGAAACCCCCAATCAATATTTTCTGGGATTTTCATAAACATAAAAGGGGTTTCTTTAATAATCAAAAGTCAACCGTTTTTGACGCAAAAATCATCTTTTTGAAATTCCTTCGATGTGGATGACTTATTTACTGTGTTTGATGATGTTTTGCATGGTGGATCTAACGTTTGACATGATTGATTGTTTTATTATGATTTATGGATTGTTATGCTTATTTTATTATGTGACGATGCATATCTCGATATTATTATATCTATATGGTTTTGCACATATATGTTAAAGCTGGTGACCTCATGAGGAAGACCCATTTAAGCTTTGCAATCCTTGAGGGATCGCCATGACCACAAGAAATTGGTCTTGCTCTATATGGCAAAGAATAAATGGATAAATTGCGTTTCCATTATTTTAAATCTGTATTATTTTCAAGACTAGCCCTATGTGGTGAGAAAATAATTGAGGAAGAATCTGTCTACCTTTAATCATATAGACAACGTGACCTCAAGAGTTATAATAAAATCCTAACTGCTCTGCTTGTTGGTGAGGAGAGGGATAAACTCCCTTCTCAAGATCCATAATTTTAGACCAACTGGGCTATAGTCCAAAAAGAGATAGAGGCAAGGGACGTGGATGTGGTTCCTCAAATAGAGGAAAGCACAATAGAAGTAATGATTACCCAAGAAGAGGTAACAACTCATCCCAAGGTTGAGAAGATCACTCTGATAGAGGTGATTACAAAGGTCCACAAAAGACTTAGGATTTGAAGGAAAAACATATTTTTGATTTGACAACCTCTATGTGTTACCGTTATGGTGAACCTGGACATTGGGCATGTGTTTGTCATGGTCCACAAAATAAGGTTGATAAATATCATAGCTCTAGAAAGACTAAAGCTTATGCAATGGAAACGAATATAATCACTAGAAAAGAAGATGTTAAGTATCTTGACCGAATGATCGATGAAACAGAAATTGCCATCGAGGTTATTGCTCGTCTGGACTGTTCTGATTTTTGTGATGATCCATATTATGATTTACTTAGCCATGGAGTTTAAGTCAATGCAGTAGTGAAGTTGATTATTTCGTCTACTCTCTTTTTCCTTTTATTTTAATCTGAATAATGTTCATATGAACCATGCCTAGTTCATTAATTCCCTTTAGTACTTTAATTTAAGTATGAATGAATAAAATCATTCTTGATTGAAAACCACTTACTTATATTCTATTCAAATTTTCAAAACATGATTTTTTAAATGAACATGAAAATTGATTTTTATGCAAATTCCACCAAATTGGATTTTGCTTGCCCAAAATATTTTTCATGAGATTGATTATCGTTTATGGTAATGATGTATATTTCTCATGGATGTCATGATTATGATTTATATTTCTCATGGATGACTTGTGGTTTAATATTTTGGCATGAATGTTATGGTTGATCATTATCGCATTAATGTTTGCATATTTTACATATTATTTTTGAGGTGGGATGGCCCATTCTCTATTTGTGATGTACAAAACTTGATTATTGCAAACAATATTTTAGTTTTTGGTGCATTTTATTATATTTGTATGAATGTATGTATTGTTATGTGATGCATGATTCAAATGAATGTACTTATTGAGGATGGTATATAGGGCAATAAACTTTTAAGATATCGATCATTGCGAGGTAAATGCATATAGAAGATTTGAAATGACATGGTTGTAAACATGGTCATTACATCCCATATTTAGACATAGCCTCTAAAATGTAACCACGTTTACATGGGTATGTACTTCTTCATTGTCTATCGATTGATGAGACACTAGAGAAAGGGTAAAGGAATGTGTTCTAGTGGAACAATTGCCTTATCAAAATTGCATAGAATTACCCGATATTTTGATGATGCAAACTAGCTAAGTATTTAGAAGCCTAAATTGATATCCTAATAACATAAGATCTTAGTGTTAAGGATATGGCTGTAAATGAACCTGTATATCTTAGCAATACAAAATACTGCTCCACAAAAATGGAGAGTATAATCATGCCCTTATGAAACTCAAGATGAGTTACTTACCTCATAAAGAACGTAAAAGTACATTGCTTAGAACTAGCATTAATGCACCATACTCATGCAATTCCTAGAGGGAATTTAATAAAGCATTGAGGATAATGATCATTTCTTTCCGCCTTGAGGGGGAGTAGAACATAACATTGCAACATTTCTTTCCGCCTTGAGGGGGAGTAGAACATAACATTGCAACATTTCTTTCCGCCTTGAGGGGGAAAAGAACACTAATCTAAGACACTCTTTCCGCCTTGAGGGGGAGAAGAAAAGAATGTGTAGAACATTCCATTTGTTTCAAACTATCATCTAATGTAGCTAATGCTGAAGCTAGAAGCTCACATTAAGGAAATCTCTTTAGAACAATGTGTCAGATCAATGGCACATAATTCCTAATGCTACAATAAATTGATTGAAAATGGGGAATCAAAATCCTTTAGAAGGATCTTGCTAGCCACTAAAATGATTCATAAATCATAAGTCTATCGCTTGGATTCCCAAAATGACAAATTAGAAAGTGTCTTAAATCGTGATTATGATTCCTTCGACGAGTGATTTGAAGATTGTTCTCCTTTTAGTGAAACAACATTCTCGTCGTTAGGGAGAGATAAATAAGGAAATCGTTTCAGTAGAACGACGTGAAACTGTTCCTAAAAATCGTCTCATCTAAACCCCCTATTACCTAAAGGGACTTTGAATCATAAATCAGATGATACAATAGAAGATGACTAAGGTCATTAAGCCAAATAAATGTCTTAGAAGAGACAAAGTCCCAACATGGCTTGAAAACAGTCTATGGCACATCGAGTATGTGATAGACCTATACTTTCTGAAAATCATGCCCTACACCCATGATGGGGTGAAGTATTACGCTCTTAAAGAGGTAATACATATTTCCAAATTCTCTAAACCTCTAGAGGAGGAAAAAGATGAGACATAGAAAACCTTGATTAAGGTTAATGGTACCAAAATTAAGAGAATTGTGGAAGATGATCATCAATGATATACACATATATATAGTAGCTACTAACTATCAAAAGGGCGATGATTAGTTATTCCACACATAGAATTGGCCAATAAACTTGAAGTGTGGAATACATAACCAAATGAGGTTATACATAGTTTGCACATATTATAGTCCTGAAGAATCATGAGAGGATATGAAATAGTGCAAGTATGGAATATGTGAGTAGATATGACTCAATAGTGCAAAGAAAATGACATATGAGAGCAAGTGACTCTATAGTGTTCATATATGAGCTCAATGATTATTACATCTATATGATGTAATTGTACTCAAAAGTACAAGTGAAAATGTGCAAGGTGCACAGAGGAGAATGTCCTCAAAGTATCATGATACTATAAATATAAAGGATCCCATATTGACGTCAAAACTCTTGAGGAGTTCATAGTTCTAGAACATGCATGTAAATAGACTATAGTAGTTTGTATTATTATGTTTTTCATACTCATGAATCTCATGCTGCATACATAATTATGTTTATGAATGATTTAACAATTTATCTTGATTGAATGATATTGTTTTTCTGAATGCAATGTAATTTCCTCATACTCATAAGATTGAGACAACCGGTAAGTTGTGCTAATTAAAGTAAATCATTGTGACAACCTATAGTTGTACAGTTGAAGATACTTTCCATATCTGTAAAATTGAGGAAACATTTGTAGACTATAAGCTCTACTAATTAATATGGAAGAATTAGAAGACCTGAAGTTCTTCTACCAGGAGCATTTGCTCTGGTTATGATCTTCTTCATATGACAAAGTTATTTTGGTCAGGTTTTGATAAAGAAGATCTTGGTATTAAAATACCACATCTTAGTGCTATTGGAGCATTGGTGCACTTAGCATAATATACATAACTAGTTCTTGACCCCACATGTCCATGTGTTTTCATCAAAAAGAACTAGTAATGGTTTCACCATTCTAACTGTTTGTGTAGATGGTATAAACCTAATAGGATCTCTTGAGGAGATCAATGAGGCTGCAAATTACCTAAAGTAGGAATTTGAAATGAAATATCTTGGTAAGATAAGATTTTGCCTCTGCCTTTAAAGTAAGAAGTTGTACTAGTGGGATTCTTTGTCCATCAATCAAAATACACTGAAAGTGTCTTGAAAAGATTCAATTTGGATAATGCACATCCACTGAGCACACTTATGGTAAATATTAAAAGAGTATATTTAAACCTAGAGAAGATGGTGAAGAGGTCCATGACCCTAAGATACCTTATTTGAGTGTAATTGGTGCTTCATCATACTTAGCACAATGTGCTTACTCTAACATTGTTTTCCAAATGAATCTGATAAGCTAGATATAGTTATGCACTAACTCATAGGCTTTGAGTTGAAATTAAGCATATCTTGAGATATCTTCGTAGAATTACTAATTTAGGCATATTTTACTTTTGTAAATTACCTAATGATTCCAAACTAGTTGATTATGCAGATTCAGGTTGCTTACCATTATCTGATAGTTCCAAACTAGTTGGTTATGCAGATTCATGTTATTTACCTAATCCACTCAAAGATTGATCACAAGTGATCTTTGCCTTTCATTTCATAGTGATCAATAAGACAACTTTGGCAATCTCTTATGAGATCTTAAACACTTCCTGAAACAATGTTGCTCATCAACAATGGATACATCAGAGGAGGCAAGCACATTCCCCGAAGTATTTCTTCACCCAAGAACTCCAAGGAAGGAAAGAAATCGTCATCCAACGAAAGCTCGTACTTGAGGTTGGTATATATTATATAAAGCTCATCTTCAAGGAGAGTTCGACATCAGGGGGAGAAACCAAGCATCAGAAGACTGTACTCTTTTTCCCTTAGACTAGGTTTTTCCCATTGGGTTTTTTACTAGCTAGGTTTTTAATGAGGCAGGTTTATCTTTGTAATTGGAATTCACAAAGTTGTACTCTTTTTCCTTGGCTTTGGTTTTGTCCCATCTAGGCCATGGGTTTTCCAAGCAAGGTTTTAACGAGGCAACGTGTATTGTGATTATCCAAGGGGGAGTGTTAAGAAATATTGTATTTTCTATTTATGGGAGTGGATATTCACAATCCACTATTCCCTTAATTGGTTTGATCTTCCAAGATCTTATATTGACTTGGAGATAAAGCCTACTAAGAATCTATAAATAAGAGAGGTTTTGGGGTGCCATAAACCCTACAATTTTCGGCCAAAAAAAAAAAAAAAAAAAAAAAAAAAAAAAAGT

>transcript_51 full_length_coverage=2;length=6569

GGTAGAGAAACATGTTTTATTCTCAATTTATATTGGCAAAAAAAGGGCCACTAGGGACGATATGGATAGCTGCACATTTGGAGAGAAAGCTTCGCAAGAATCAGGTTGCAGATACCGATATTGGCGTGTCCGTAGATTCAATTCTTTCCCCTGAAGTACCCATTGCACTCCGGTTGTCCAGTCATCTTCTGCTTGGTGTAGTCAGGATATATTCTAGAAAGGTTAATTACCTTTTTGATGACTGCAGTGAGGCTTTGCTTAAGGTGAAGCAAGCTTTTCGCTCCACTGCAGTTGACTTACCTCCAGAAGAATCCACTGCACCATATCACTCTATCACCTTGCCTGAGACTTTTGATCTTGATGATTTTGAGCTGCCAGATAATGAAAATTTTCAGGGCAACTATGTTGATCACCACATCAGTACAAAGGAGCAGATTACGCTCCAAGATACTATGGAGGGTGTTGTTTTTTCAACATCTCAGTTTGGACTGGATGAGCAATTTGGCGATGGTGACGCTTCTCAAATCAGTTTCGACCTTGATGAGGACTTGTTTCTTGACAAGGTTGCAGCTCCAAGACATACCGGAGTTATGTTGGATTCAAAGGATGATCCCCAGGCATCTGTACAGCCAATGACATCCCTTGACAATTATGTAAATAACGCGTGGACGATTGATACCTCAGAAGCCATGCCAGTGAATGGTCGTGGAGAACAGCATAAAGGCCTAGTTGCAAATAATGATATCATCGAGTCTGCCCAGGCCCCATCTACTCCTGGATTAATTGAAGAGCCAAATTTATCCAATATGCAAATGGGCCTAGCTTGCGATGATCATATGGAGTTAGAAAACTACAATTCAATGGAATTTGAAGCGAGAGAAGCCATAGGAAATTCTTTCAGTAAATCAGATCATCACGGGGATAAAAAATGTGTGGATGGGCCTTTGTCCAGTATCGATATGAATAATGATTGCATGCCTGTTCAGGAAAATGATCATGTTATGGGCGAGCCCGTGATCTCACAAGCCAACCCACAAGGAGATTTACCGTCTACGTCTGAAAATGCGGGGAAAATTGAAGTGCAAAACTTCTCCAATATACAAATGACTATGGCTTGCAATGACCTTATTGAGTCAGAAGATCATAATTCAATAGAACTCGAAGTGAGAGAGACCGCAGGAAGTTCTTCAAGTAAATTAGATCATCACGGGCATAAAAATTCTGCAGAGTGGGCTTCGTTCAATATAGATATGAATCTTGATTGCATGCCTGTTCAGGAGAATGATCATGTTATGGGTGAGCAGGAGATCTTACAAGCCGAGCCACAAGTAGATATACCATCTACTTCTGATACTATGGAGAACATGTTGGATAATCCTCTTTTAGCATCTTTACCATCTCCGGTTCTAACTGAGAAAGAAAATTCTGTTTCTGTAGTGTCAGAGTTTCAGGGTAGGATTACTGCACCTGATGACATGGACAGTGTGGGGGATTTACAAAATGGAGTTCTGAGCAAAACCGAACAAAGTACAACGTTTACGGGTCAAGACCATTTGGAATGCACGGAATCCTTAGGAGTTAGATTGGAGGAAACTGTAGCCTCTCCTAGTTTTTCCCTTGGGACCTCTGTTTTGGAGGATGCTCGTCATAAACCAGGTTCTGGTGTTGGACCAATTTCTGAGAAGTCCAGTTTAACCAATACCTATCCTCTAGTACCTGGAGGTATTTCTGGGAGTGATTCAGCATTGCTTCAGCCAGAGATTTCTGATAGTGTAGAGATAGCTGGAAACATGGAAACATGCCCCCCTAATGCACTGGTATCTGTCTGTTCTTTGAAGTCTCCTACTGGACCCGGGCTTGAGAATGTCGAGGCTTCGGCTGCTCATGAACCAAAACATTCTGAGACTTCGAATCCCAACCAAGAAGGGCCATCTACTTGCATCCATGTACTTCAGCAAGCATGCAACTCACTTGAAAACGAACCTGTTAAATCATTTTTTGGAGGTAACAATTCTATAGTTGTTCCTGACATACCCTCTGAAGTTTTATTGGAGGGTCAGGCTGGTCATAAACCAAAAGATCCTGAGATTATGAACCCTGATGAGGAAGGGCCATCTACTCGCATCCATGTACTTCAGGCTTGCAATTCAATTCAAAACCAACCTGTTAAATCATTTCTTGGAGGTTACAGTTCTGTAGTTCCTGACTTACCTGCTGAGACATCTGAATGGGAGGCAACCCATATTTTGGGAGCTTCAACTGAGGTGCAAGCAGCGAATTTTGATGGTACACCTGGGCACCAGCCAGCACCAGGGCAAGCTGCGCTACCAGCCAGTGGCCATCAGCATGAGGCCAGCACCGCAAGGGTTCAGCAGCAGTTGCCACAGGAGCCCATAGCCACCGTCAGCTGGCGCCCTACAGCAAATTTTGATGGTACCCCTGGGCACCAGCCAGCACCAGGGCAAGCTGTGCTACCAGCCAGTGATCAGCACCACGAGGCCAGCACCACAAGGCTCCCGCAGCAGTTGCCACAGGAGCCCATAGCCGCCGCCAGCTGGCACCCTACAGAGAATTTTTTTGACGGGAGCACTGGCCATCAGTCAGCACCATGGCAAGCTGCGGAACCAGCCAGCAACCAGCAACACCAGGACACCAGTAGCACCAGGTTGCAGGTGCCAACAGCCACCACCAGCTCCCCCACAACAGATGTAGATAATTTATTTAAGGGGACCACTGGCCATCAGTCAGCACCATGGAAAGCTGCGGTACCAGTCAGCGATCAGCAACACCAGGATGACAACACAAGGCACCCCCAGCAGCAGCTGTTGCAGGACATCAGTAGCACCAGGTCACAGGCGCCAACAGCCACCACCAGCTCCCCCACAACAGATTTTTTTGATAGCACTACCGAGCACCAACCAGTATCATGGCAAGCCGTGGCATCACCCAGTGACCACCAACACCAGGTGGACACTGCAAGGCACCTGCACCAGGGCAGCACCAGGCTGCAGGCGGCCACAATCACCACCAGCTGTCCCCCGGCAGTGACTTTTTTTGACGGTTCCACTGGGCACCACATGGCACCATGCCAAGCTGCGGTACTAGCTGGCAACCAGCAACACCAGGACGACACTGCAAGGCAGCTGGGACAGAGGTCGCAGGACACCAGCAGCACAAGGCCTCAGGCGCCCTCAACCACCACCACCAGCTGCTCCCCAACAGCAATCTTTTTTAATGGCTCCACTGATCACCAACCGACACCATGGAAAGCCGCAGCACCAGCCAGCGACCAGCAACACCAGGCTGACACCGCAAGCCACCTGCACCAGCAGTCACTGGACACCATCAGCACCAGGCTGCAGGTGCCCACGACCACCACCAGTTGGCCCCCAACAGTGAATTTTTTTGATGGCACCACTGGGCACCAACTGGCCCCATGGCAAGCTGCGGTACCAGCCAGCGACCAGCAGCACCAGGCCGACACCCCAATGCACCTGCAGTCGCAGTCGCAGGACACCGGCAGCACCGGTTTGCAAGCTCGCACAATCACCACCAACTGCCCCCCAACAGCAAATATTTTTGATGCCACTGCTGGGAACCAGCCAGCACCATGGCAAGCTGTGGTACAAGCCAGTGACCAGCAGTGCCAGACCGGCTCTCCAAAGTACCAGCTGCAGACTCCTGAGGACACCAGCAGCACCAGGCCGCAGGTGCCCACAACTACCACCAGCTGGCGCCAAACAGGAGAAGGCTACCGTGCAACTGATATGGGCCAACCGTCCTTAGACGTGAACCAGTTATTGGAACCAAATTCTTGTCAGTATGTTGACGCAGGTCGCAGGAAGTTGGATGAGCAACTAGCGAATGCCAAAAATACTGTTGAGAATTATTTTGGTTATGCCGATTTGCCTGCACCTGAAAAAATGCTTTCTGTTCCAGAAGGGCGTAGCCATCTAGGGAATAATTTTTTGTTGGAGTCTACTCCGGGCAAAGAAGCCACAATGCATGGCGAAGGAAATGGCACTGGGGTCCAAAACATTACTGGAAATAAGCGTAGTTATATGGAAAGTATTCTGACTACGCAGAGCCTGAATTCGGTTGAACCATTTGAGAAGTCCCGATCTGAAAGATCTACGGGATCTGTTCCTGATGATGATGATTTGTTATCTTCCATCCTAGTTGGAAGAAGGTCTTCAATTTTGAAAATGAAGCCCACTCCACCTCCATCTGAAACAGTGTCTGTGAAACGCCGTCGAACTGCACCCCGGGCCAGTGCATCTAAGAGGAAGGTGTTTATGGATGATACAATGGTCTTGCATGGCGATATAATACGACAACATTTGACAAATACTGAAGACATACGTAGAGTACGGAAGAAGGCTCCTTGCACTCGACCTGAAATTTGGATGATTCAGAATCAATTCTTGGAGGATGAAATTTTCGGTGATCCCATATTTAGTGGTATGTCGTCAGAATTGATTTGTTCACTCGGTCAAAGATATGATCCGAGTATAATTAGGGTTTCTCAGAATGATGAAAGTATAGCTTTGTTTGGAGCAGCAAAGAATGGAGAGCTTTCTATGACACCAAATGTTATCAATGAAACTGAACCAGAAAGGATCATTGAATCTGAAATAACCAGAAATGACGGGGAAGTTCAATCTGCCGAGACTGTTGTCCAAACTGAGGTTCAGCATCTTGATAATGATACTCAAATGCAATTGAATGCTAATACTACGATGCCTGAGCTTAGGACTTTGCACCCGGAAACAGATGGGATCACTGAACCTATAATAGTCAGAAATGACGGGGAAGCCCAACCTGCCATGGCTTTAGTTCAGACCAAGGACCAGCATAATTATATTGCTACTCAAATACAAATCAATTCTAATACTATAATACCTGAGCTCAAGTCTTCACACCCTGAAACGGAAGGGATCACTGAACCTATAATAGTCAGAAATGACGGTGAAGCCCAACCTGCTAATACTTTTGTCCAGACGGCAGAGCTGCATAATTACAATGATACTCAAATGCAAATGAATGCTAATACTGTGATACCTGAACTTGGCACTTTGCAACCCGAACATATGGGCAAGATAGCTGAAATGGAATTTGCTAGAGGTGGTTCTGCAGTTGCTAATGCAGAGGAGACTTCTGTCGCTCTTGGGATTGGATTATCATCCCCAGTTGATTTAGTTTCTGTAGATATTTGTAACTTGTCAACTGATCCTGTGGAGAAACCCAGTGGTTCTCCACAGATCGCTGTGGCTTGCTTGGTTCCTGACCAGAAGTTGGATTCTCAATCTGTTGAGAAGGATGCTTCTGTAGTGGATTTATGCATTGAGAAAGGAGTTGAGCCTGTTGAAGTTTCTGAAATTCAAGTCGTAGTTGGTACTGAATCTTGTGCAAGTTTTGAGAAAGATGCTTCTGCAGTAAATCTTGGGCAAGGAGTTGAGTTTATTGAAGTTCCAGAACTTCATGACGGCGATATTATTGGAGCTGGGACTGAATCTGGTGCGACAGGTGAGTTTCTTTTGGAAGAAAACAGAGGCGGTCTTTCAGTTGAAAACAGAACAGATAATCCTACCGACAGTGTAGATCTTCCCCTTGAAACCGTCTCTCCAGCAATTGACTTGGTTGTTGTGACTGAAGACCAAGCTGAGGAGGAAACTGCTAAAATCGAAGAAGGGGTTGTAAATGAGGCTACTGAAAAGGGGTATGATGGCAGGGATCGAACCTCTTACGGTGTATATAGCCAAGAACCTAAAACTGATTATTCCCTGAGTGACGGACAAGATGCATCCTTGAATAACGTTGAAAAATCTGGTGGCCAGGAGGTTGTACCACAAGATGTAAGAGATGCCGAAACTGCTTCCTTCGACCATGCTGATGTTGAAATCCATGATGATGTGGAAAATGAAACAGTTGAGCATGATACTGAGTTCCTCAACTTTGATAACGACGGTGATGATGATGGCGGAGCTGACGATGACTATGATGACTGTATGCCTTGTCCAGAAGAAACCCGCATTCTTGAGAACAGCGGATGGTCTTCCCGTACCAGGGCTGTTGCCAAGTACCTCCAAACCGTATTCGACAAAGAAGCTGACCACGGTAAAAAAGTTATTCCATTGGACAACCTTTTGAGTGGCAAGACTCGAAAGGAAGCGTCGAGGATGTTTTTTGAAAGTTTGGTTCTTAAAACAAGGGATTATATACATGTAGAACAGGAAAACCCTTTTGATAACATTATTATAAAGCCTCGAGTAAAGCTCTTGAAATCGGAGTTCTGATATATCCTACATAATTATCCGCCCGAGTTTGGGTATTCTTCTTCATTTTGGTGTGTATTTTTGTTTATTGTTTTGTTTAGTTGTTGTAACTATTAGTTAAAAGTGGGGTCATGTTTTATCCCCAGTTGTTGTAACAGTGTTGTAACATTAGGCTGCATTTATATGTGTACATATATGTAACTGTAATAATGTGATTCACCATTTGCAATTAAAGATGCTAATCAATTTATTTGTTAACCTTAAAAAAAAAAAAAAAAAAAAAAAAAAAAAAGT

>transcript_53 full_length_coverage=2;length=6479

GGGGCGCTCTCCCTCTTTCCGTTGCCGGCACTAGGTTGAGAAAAAGGAGGAAAAATCGAGGGGTTCATCTCTTTCTTCTTTGGGTCCGAAGATTTAAAAGAGACGATATATAGATCAGAATAGAAGATACTAATATAGGTATAGATATAATATATAACAATGTCGGAAGAGGAGAAGTTGTTGAAGGAGGCTAAGAAACTGCCATGGGAAGATCGGTTATTACACAAGAACTGGAAGGTTAGGAATGAAGGGAACATCGACTTAGCCGCCTTATGCGGTTCCATCACGGATCCCAAGGATCATCGCCTTCGTGAATTCGGACCGTTGTTTAGGAAGACGGTAGCGGATGCAAATGCACCTGTACAAGAGAAGGCTCTAGATGCTTTAATTGCCTACTTACGAGCTGTTGATGCTGATGCCGGAAGGTATGCAAAGGAAGTATGTGATGCAATTATAGCAAAATGTCTCACTGGTCGGCCCAAGACGGTGGAGAAGGCTCAAGCAGTATTCATGCTTTGGGTAGAACTGGAGGCTGTTGACGTTTTCCTGGATGCTATGGAGAAAGGGATAAAAGCCAAAGTTGCAAAAGCTGTTGTCCCTGCAATTGATGTGATGTTTCAAGCCTTAAGTGAATTTGGGTCAAAGGTTGTTCCCCCAAAAAGAATTCTAAAGATGCTTCCTGAACTCTTTGACCACCAAGACCAAAATGTCCGTGCATCCTCCAAAGGATTGACTCTTGAGCTTTGTCGTTGGATTGGAAAAGATCCAGTGAAATCAATATTGTTTGAGAAAATGCGGGATACAATGAAAAAAGAGCTAGAGGCTGAGCTTGCCAATGTTACGGGGTCAGCCAGGCCCACTCGCAAAATAAGATCTGAACAAGATAAGGAGCCAGAACAGGAAGCTGTTGCTGAAGTTGTTGGTCCTGGTCCATCTGAAGAATCTGCAGCTGATGTTTCTCAGGACATAGATGAGTATGATCTTGTAGATCCTGTTGATATATTAACTCCTTTGGAAAAGTCAGGGTTTTGGGATGGAGTGAAAGCCACCAAATGGTCAGAAAGAAAGGACGCTGTTGCAGAGCTAACCAAGCTTGCTTCTACTAAAAGGATATTGCCTGGTGATTTCACGGAAGTTTGTAGGACACTTAAGAAGCTTATTACGGACAATAACATAGCTGTTGCAGCTGAAGCTATCCAAGCTATTGGGAATCTTGCCAGGGGCCTAAGATCCCATTTTTCTGGGAGTTCACGGTTCTTGTTGCCAGTTTTACTGGAAAAACTGAAAGAGAAAAAACCTACTATAACTGAGGCGCTTACTCAAACTCTCCAAGCAATGCACAAGGCTGGGTGCTTAAATCTTCCTGACATTGTGGAAGATGTTAAAGTGGCTGTTAAAAATAAAGTTCCTCTTGTGCGCTCCTTGACTTTGAATTGGGTGACATATTGTATTGAGACTAGCAATAAGGCCGTTGTTCTCAAGTTGCACAAGGATTATGTTCCGATCTGTATGGAGAGCCTCAATGATGGGACACCAGATGTGAGGGACGCAGCCTTTTCGGTTTTGGCGGCAATCGCTAAGTCGGTTGGTATGAGGCCTTTGGAAAGATCACTGGAGAAACTTGATGATGTTCGAAAAAAGAAGCTTTCTGAAATGATTGGGAATTCAGTTGTTGGTCTGCCTACTGCCGCGAACTCAGCTGTCCAAACAGCGGGTGGGAGTGTATCATCTTCGCAGGCTTCAGAAGGACCATTTGTTAGAAAATCGGCAGCAAGCATGCTTAGTGGGAAGAAACCTGTGCAGGCAGCTCCTTCTAATAAGAAAGGAGGGCCTGTAAAATCGGTTGCAAATAAGAAAGGAGATGGAGCTGGACAGTTGAAAACTTTAAAATCAGTTGAACCTCCTGAAGACATTGAGCCAGCAGAGATGAGCCTTGAAGAAATTGAAAGCAGATTAGGTTCTCTTATACCCGCGAATGCTGTTTCTCAGTTAAAGAGCACTGTATGGAAAGAACGACTTGAAGCAATTGTCTCTTTAAAACAACAGGTGGAAGGCATTCAAGACCTTGACCAGTCTGCAGAGATGTTGATTCGTTTACTTTGTACTGTTCCCGGGTGGAATGAGAAAAACGTTCAGGTCCAGCAACAGGTCATTGAAGTTATTACTCACATAGCTTCTACTGTGAAGAAATTTCCCAAAAAATGCGTTGTGCTTTGCCTTCTAGGTATAAGTGAACGGATAGCAGATATCAAGACCCGTGTTCATGCCATGAAATGCCTTACTGCTTTTTCCGAAGCAGTAGGTCCTGGATTCATTTTTGAGAGACTTTACAAAATCATGAAAGAGCACAAGAATCCTAAGGTTCTAAGCGAGGGTATATCGTGGATGGTTTCAGCAGTTGATGACTTTGGCGTGTCACTTTTGAAACTGAAGGATTTAATTGATTTTTGTAAAGATACTGGACTGCAGTCCAGTACTGCTGCAACTAGAAATTCTACTATCAAGCTTATTGGCACTCTACATAAGTATGTCGGACCAGATATTAAAGGGTTTCTTGTTGATGTCAAACCTGCACTTCTTAGTGCACTTGATGCAGAGTATGAGAAAAACCCATTTGAGGGTGCCTCTGCAGTTCCCAAAAAAACTGTAAGGGCAGCAGACTCTACAATGTCTATGGCTGCTGGTGGGCTAGATAGCTTGCCACGTGAAGATATTAGTGGAAAGATTACACCTGCTCTGTTAAAGGGATTGGAAAGTCCTGATTGGAAGGTCCGTTTGGAATCAATTGAAGCTGTAAATAAAATCTTGGAAGAGGCCAATAAGCGGATTCAACCTACTGGAACTGTGGAGTTATTTGGTGCTCTTAGAGGGCGTCTCTTTGACAGCAATAAAAATTTAGTCATGGCAACTTTGAATACCGTTGGTGCTGTTGCGTCTGCGATGGGTCCAGCAGTTGAGAAGGCAAGCAAGGGTATTCTGTCAGATGTTTTGAAATGCCTGTGTGACAATAAAAAACATATGAGAGAATGTACTTTGACCACTTTAGATTCTTGGCTTGCCGCTGTTCACCTCGATAAAATGGTTCCTTACATTATGACGTCTTTAACGGATACCAAGATTGGTGCGGAAGGACGCAAGGATCTATTTGATTGGTTATCAAGGCAACTTTCTGGATTAGTTGATTTTTCTGATGCTGTACACCTGCTGAAACCTGCAGCAGCAGCTATGACGGATAAATCATCAGATGTTCGAAAAGCAGCAGAAGCATGCATTGCTGAGATTCTGAGAGTGTGTGGACAAGAAACAGTGACCAAGAGTTTAAAAGATTTACACGGTCCAGCTCTAACTCTTGTTCTTGAGCGGCTGAAACCACATGGATCTTTTCAAGAATCATTTGAACCAGTGAAAGCAGCTTCAATGGGGGTAACATCTAAAATCATTTCCAAAGTTGGAAAATCTGGCACCAATGTTTTACCAAAGCATGGAAACAAAGCCATATCTTCGAGAGCTGTTGCGACTAAGGGTTCAAGGTCAGACTCAATAATGTCTGTTCACGATATTGCTGTCCAGTCACAAGCTTTGTTAAATGTCAAGGATTCAAACAAGGAGGATAGGGAGAGAATGGTTGTTCGCAGGTTTAAATTTGAAGAGCTTCGAATAGAACAGATTCAAGATCTGGAGAATGACCTTACAAAGTATTTTAGAGAGGATTTGCACAGGCGGCTTCTAAGCACCGACTTTAAGAAGCAAGTAGATGGCCTTGAGATGCTACAGAAGACACTTCCATCCGTTGGGAAAGAAATAATTGAAGTATTAGATATACTTCTGAGGTGGTCTGTTTTGCGGTTTTGTGAATCTAATACGACATGCCTGCTGAAGGTGCTTGAATTTCTTCCCGAACTGTTTGACTTCTTGAGGGGTGAAGCTTACATTTTGACTGAATCGGAAGCGGCGATATTTCTCCCATGCTTGATAGAGAAGTCTGGGCATAACATTGAGAAAGTACGAGAAAAAATGCGGGAATTGACAAAACAAATCGTCAACATATACTCCGCAGCAAAATCTTTTCCCTATATTTTGGAGGGTTTGCGCTCTAAGAACAACCGAACTCGGATAGAATGTGCTGACCTTGTTGGATTCTTGATTGACCATCATGGAGCCGAGATTGGTGGTCAATTAAAATCCTTGCAAACTGTTGCAAGCTTGACAGCAGAGAGAGATGGTGAACTTAGGAAAGCTGCCCTGAACACCTTAGCTACTGGTTATAAGATTCTTGGTGATGACATATGGAAATACGTTGGGAAGCTAACTGATGCTCAAAAAAGCATGTTAGATGATAGATTTAAGTGGAAGGCCCGAGAAATGGAGAAAAGGAAGGAAGGAAAGCCAGGTGAAGCTAGAGCTGCTTTAAGACGTTCTGTTAGGGAGAATGGGTCTGATGTGGCAGAGCAAAGTGGAGAAGTTTCACGATCCGTCGCTGGCCCAATCTTTGCAAGTTCTAGGGAATCCTATGCTTATCAGGAGCCTCAAATGGATAGAAATTTGATGCCCAGAATGCTTCCTACTGCAACAGCTCCCACAGACTGGAATGAAGCTTTGGATATCATTTCATTCGGTTCTCCTGAGCAGTCTGTCGAAGGAATGAAAGTTGTGTGCCATGAATTGACCCAAGCCACCAATGATCCTGAAGGCAGTGCAATGGACGATCTCGTCAAAGACGCAGATAGACTCGTTTCGTGCTTAGCAAACAAGGTACCCAAGACTTTTGACTTCAGTCTGACCGGAGCTTCGTCGAGGTCTTGTAAATATGTTCTGAACACGCTCATGCAGACATTTCAAAATAAAAGACTTGCTCATGCTGTTAAGGAGAGTACTCTTGACAGCCTAATTACTGAGCTTCTCCTTTGGCTTTTGGATGAAAGGGTTCCGCATATGGACGATGGCAGCCAACTTTTGAAAGCCTTAAATGTTTTAATGCTCAAGATTCTGGACAATGCAGATCGGACATCTTCTTTTGTTGTCCTGATTAAACTCTTACGTCCGCTTCACCCTTCAAGATGGCCATCTCCTGCTTCAAATGAGACCTTTGCTGCTAGAAATCAGAAGTTCTCTGATTTGGTAGTCAAATGTCTTATTAAACTTACTAAGGTTCTTCAAAGTACGATATACGAAGTTGACCTTGACCGTATCCTTCAAAGTATACATGTCTACTTACAAGAATTGGGGATGGAAGAAATCAGGAGAAGAGCTGGAGCTGATGACAAACCATTACGCATGGTGAAAACTGTTCTACACGAACTCGTTAAGCTTCGTGGTACAGCAATAAAGGGTCATCTTTCTATGGTTCCTATAGACATGGAACCTCAACCTATCATTCTTGCCTACATTGATCTTAACCTTCAAACTCTAGCCGCGGCTAGAATGTTGACTCCATCTGGACCCGTGGGTCAAACTCATTGGGGTGATTCAACAGCTAACAACCCATCTCCGGCCACTCATTCTGCGGATGCTCAATTAAAGCAAGAACTTGCTGCAATCTTCAAGAAAATCGGTGATAAGCAAACATGCACAATCGGTCTCTACGAGCTTTACCGCATAACTCAACTTTACCCTAAAGTTGATATATTTGCTCAACTCCAAAATGCCAGTGAGGCGTTTCGAACATACATAAGAGATGGTTTAACTCAAATGGAGAAAAATGCAGCAGCTGGAAGAACACCTTCAAGTGTACCAATGCCAACTCCTCCCCCAGCTTCCTTAACTTCCCCAAAATTCGCCCCACTTTCACCTGTACATACAAATTCTTTAAACGAGGGCAAAGTGGTAAATGCAAAACCCGAACCGACAAACTTCAGTTTACCACCGTCATATACAGATGAAGATCATCATAATTATGGCGACCAAAGGAATGATAGATTTCCAACTGGAGTTACGAGCGGTACGCTGGATGCAATTAGAGAAAGGATGAAGAGTATTCAGTTAGCTGCGGCTGGAGGTGGGAACCATCCGGAAGCGGGGAATAAGCCGTTAATGTATGTGAATGGGAATTTATCTCACGCATCGGGTGTAGCAGTGGAAGGTGTGCAGACTGCAGTACTTCCAATGGATGAGAAGGCGTTATCTGGGCTTCAGGCTCGGATGGAGAGGCTTAAGAGTGGTTCGATGGAACCTCTATAGGAGGAGAGGGGATAGAGATTTTGATTTTGTAGAGTGCAATGGAAGGGCAGTTGATGGTATAAAAATATGTATACTTAGATGTTTTACGCTCTTTTGTATTTGGTATGGAAGCTTGTATTTATTCTCTTGAGATGTTGTCATGGAAGAGGTTAAGGCTAGGATGAGTATTATTTGGGTATGTGCTAATGATTTGTTATGCACTGGAGTCGTGTGTCCAAAGACCAGGCATTTTGTATATTTTAGTTGAGGCCTTTCGTTATTTTTTGGAAAAAAAAAAAAAAAAAAAAAAAAAAAAAAAAAGT

>transcript_54 full_length_coverage=7;length=6527

ACTACACAACAATATACTCCCCGGAGAAAGCACCGGGTTTCGAAAGCATTAGAATTAGAAGAAGCAATGTTGGTTCAGCTTCCTCGGTTGACGAACTCTCTCAGAGACCCATATGACATCGACCATGCCTATCTTCAGCGCAAAATCATCCTCCAAAACCACAAAACCCGCAGTTCTGCGAAGTCGCTCGAGGAATCGAAGCTTGCACGAAAGATAGTGCGTCAATGGGAAGAAGCTTCTACAGAAATTCGCCAAGCATACAAGCAGTTTATCGGGGCAGTAGTCGAATTATTTGAAGGCGAAGTGGTATCAGAGCAGTTTCACGAGGTTGCTTTAACCGCCTTTCATCTCTTTCGCGGGCCTGAGGAGGAGTACAAAGAAGATAAAAGGATTGGTGAGAAAAAGTTAGAATTGCAAAAACTACTGGGCTATGCAGTTTCAGATGCAAACTTGTGGAAAATTGCCTCTTTAGCACAGAGACTTTTCAGTTTACAGCCTAGTGACTCTGGGACTGCAGTTCTTCCTGAAAGGCAAGTTGATGGGAGTAATGTTGACTTAGAGTTTGGTGCTGATCTTGTTTTTCAAGCACCAGCTCGGTTTTTGGTGGATGTATCTTTAGAGGATGTAGAATTGGTTGGGGAGGAAAGCACCACACCTTCTTTTCTTCGTGACGGATGGTATGATCATGTGGACTCTACACCTCATCAGTCTGCTGGTGATGGAGGATCATTAGATTTGAGATGGTTAAGAAATGCATGTGTTGCAATAGTGAAAGAAAGTGATTCACAGCTTTCTGGAGATGAGCTGGCAATGGCCATTTGTCGGGTGCTTGAATCAGACAAGCCTGGTGATGAGGTAGCTGGTGATCTATTGGATCTCGTGGGGGAAAGCGCATTTGAAACAGTTCAGAACCTTGTATCGCATCGCAAGGAACTTGTTGAAGCCATCCGTCAGGGCTTGTTTGTACTGAAATCTGACAAAACGGCATCAAACCCCCAAGCACGTATGCCTAGTTATGCAGTCCAGGTTACTGTTCAAAGAGAGTCTGAACGGAAAATTGATAAACTTCGACGTAAAGAGGAAAAGCGACATAGACGGGGAACAGAATATGGTGCTGAGAATGACTTGTCTGCTGCAAGTTTTTCTTCTTTGCTTCAAGCTAGTGAGAAAAAAAATCACTTTGACGAACTAATTGGGACTGGCGAAGAATTAGCAGTTACTGCTCTTCCTCAGGGAACAGTGAGGAAGAATTTCAAGGGGTATGAAGAAGTCATTATCCCTCCAATGCCGACAGCTCAAATGAAACCCGGTGAAAGGCTGATCGAGATAAAAGAGTTGGATGAATTTGCTCAAGCTGCTTTTCGTGGCTACAAGTCTCTAAACCGTATCCAGAGTCGGATCTTCCAAACGACTTATTACACCAATGAAAATATTCTAGTTTGCGCTCCAACTGGAGCTGGTAAAACAAATATTGCTATGATTGCCGTTCTACATGAGATTGGGCAGCACTTCAAGGATGGCTTCTTACATAAAGATGAATTTAAAATAGTTTATGTTGCTCCTATGAAGGCACTTGCTGCAGAGGTCACATCCACATTTAGTCACCGTTTGTCTCCGCTGAATGTGATTGTGAAAGAACTTACTGGAGACATGCAGCTTTCTAAGAATGAACTTGCAGAAACCCAGATGATAGTTACAACACCTGAGAAATGGGATGTCATTACTCGTAAAAGCAGTGACATGTCGCTCTCATCACTGGTAAAGCTCTTAATTATAGATGAAGTGCATCTTTTGAATGACGACAGAGGCTCTGTAATAGAGGCATTAGTAGCCCGGACACTTCGTCAGGTTGAGTCAACACAATCCATGATACGCATTGTGGGCCTTTCTGCCACTCTACCCAGTTATTTGGAGGTTGCACAATTTCTGAGGGTGAATGCAGAGACAGGACTGTTCTTCTTTGACTCTAGTTATCGTCCAGTGCCTCTTGCACAGCAGTATATTGGAATTAGTGAGCGGAACTTCGCAGCTCGCAACGAATTGCTGAATGAAATATGCTTTAATAAGGTGGTTGATTCATTAAGGCAAGGGAATCAAGCAATGATTTTTGTGCATTCAAGGAAAGATACAGCAAAAACAGCTGAGAAACTGATTGAGCTCGCCCAGAAGAAATCTGAGGTTGCACTTTTTGAGAATGAAACACATCCACAATTTTCCTCGATGAAGAAGGAAGTTCTCAAGTCCAGAAACAAGGATCTTGTTAATTTTTTTGAGAATGGGGTTGGTATTCATCATGCTGGAATGTTACGTGCCGATAGAGGTCTGACTGAGCGTCTTTTCTCTGATGGACTCTTAAAGGTACTTGTTTGCACAGCAACTTTGGCATGGGGAGTAAATCTACCTGCTCACACTGTTGTCATTAAGGGGACCCAGTTATATGATCCAAAGGCTGGCGGGTGGCGAGATCTGGGCATGCTTGATGTTATGCAGATCTTTGGGCGAGCTGGAAGACCTCAATTTGACAAAAGTGGTGAAGGGATCATAATCACTTCTCATGATAAACTGGCGTACTATTTACGGTTATTGACAAATCAACTCCCGATAGAAAGTCAGTTCATTGGCCCCTTGAAAGATAATTTAAATGCAGAGGTAGCATTGGGTACTGTAACAAATGTTAAGGAAGCTTGCGCATGGCTTGGGTATACGTATCTTTTTATAAGAATGCGTTCAAATCCTTTGGCATATGGTGTTGGATGGGATGAGGTTATGGCTGATCCTTCTTTAAGTTCAAAGCAAAGGGCTCTTGTTACAGATGCTGCACGTGCACTCGACAATGCCAAAATGATGCGGTTTGATGAGAAAAGTGGAAACTTTTACTGTACAGAGCTTGGTCGCATTGCAAGCCACTTTTATATTCAATATTCTAGTGTTGAAACTTACAACGAAAAGCTGAGACGCCACATGAGTGACAGTGAGGTAATTGACATGGTGGCCCATTCTTCTGAGTTTGAAAACATTGTTGTTCGAGACGAGGAGCAAAATGAGCTAGAAGAATCGGCACGCAAATCATGCCCGCTAGAAGTGAAGGGTGGTCCTTCAAATAAATATGGAAAAATCTCAATTCTCATTCAGTTGTATATATCTCGAGGCTCTATAGATGCCTTTTCATTAGTTTCTGATGCTGCCTATATAAGTGCAAGCTTGGCTCGTATCATGCGTGCGTTGTTTGAAATTTGTTTGCGTAGAGGGTGGTCTGAGATGTCTTACTTCATGTTAGAATATTGCAAGGCTGTGGATCGCCAAATTTGGCCCCATCAACATCCTCTCAGACAATTTGACAAAGATATTTCAGCCCAAATACTGCGGAAGCTTGAAGAACGAGAGGCTGACTTAGATCGACTCTTTGAGATGCCCGAAAAAGATATTGGAGCACTTATTCGTTATCAACCTGGTGGAAAGTTGGTTAAGCAATTTTTAGGTTATTTCCCATGGATTCAGTTGTCTGCTACTGTAAGTCCAATCACCAGAACAGTTTTGAAGATAGATTTGCTCATAACGCCAGACTTTACATGGAAGGACCGTTTTCATGGTACTGTGCAGCGTTGGTGGATTCTTGTTGAGGATTCTGATAATGATCACATCTACCACTCAGAGCTTTTCACGCTGACAAAGCGGATGGCAAGAGGGGAACCTCAGAAGATTTCGTTTACGGTGCCAATATTTGAACCACATCCACCTCAATACTACGTTCACGCTGTTTCTGATTCTTGGTTGCATGCGGAGGCTTTCTACACTATATCTTTCCACAATCTTGCACTGCCAGAGGCTCATACTACTCACACGGAACTACTAGATTTGAAGCCTCTTCCAGTGACTTCGCTTGGCAATGAAACTTATGAAGCCTTGTATAAGTTTTCACACTTCAATCCTATCCAAACACAGAACTTCCATGTCTTGTATCACACAGACCACAATGTTCTCTTAGGAGCTCCTACCGGAAGTGGAAAAACTATAGCTGCTGAGCTGGCTATGCTTCATCTCTTCAATACTCAGCCTGATATGAAAGTTATTTACATAGCACCTTTGAAGGCTATTGTTCGAGAAAGAATGAATGATTGGAGAAAACGTCTTGTCTCTCAGCTTGGAAAAGAGATGGTTGAAATGACTGGAGATTATACTCCAGATATGGCAGCCCTCTTGTCAGCAGATATCATAATTTCTACTCCTGAGAAGTGGGATGGTATCAGTCGCAACTGGCATAGTCGAAGCTATGTCACAAAGGTGGGGCTTATGGTTTTGGATGAGATTCACTTACTTGGAGCTGACCGTGGACCTATCCTTGAGGTTATTGTTTCCAGGATGAGATACATATCATCACAAACAGAGCGTGCAGTGCGGTTTGTTGGCCTTTCAACAGCTTTAGCAAATGCAAATGATTTGGCTGATTGGTTGGGTGTTGGAGAAATCGGACTCTTCAATTTCAAGCCCAGTGTGAGGCCTGTACCCCTTGAGGTTCATATCCAGGGATATCCTGGGAAATTTTACTGCCCAAGGATGAATAGCATGAATAAGCCTGCATACGCTGCCATATGTACCCATTCGCCTACAAAACCAGTTCTTATATTTGTTTCATCTCGTCGGCAGACAAGGCTTACTGCACTGGACCTCATTGGGTTTGCAGCATCAGACGAACATCCGAGGCAGTTTTTGAGTATTCCAGAAGAAGCACTTCAGATGGTTCTTTCTCAGGTCACTGATCAGAACCTGCGGCACACCTTACAATTTGGGATTGGGTTACACCATGCAGGACTGAATGACAAAGATAGATCTCTGGTTGAGGAACTTTTTGGAAACAACAAGATTCAGGTATTGGTTTGTACTAGCACGTTAGCATGGGGTGTAAACCTTCCAGCACATCTTGTTATCATCAAGGGAACTGAATATTATGATGGAAAAGGAAAAAGGTATGTTGATTTTCCTATCACAGATATCTTGCAAATGATGGGTCGTGCAGGCCGACCACAATACGATCAACATGGAAAAGCAGTCATTCTTGTTCATGAACCCAAAAAGAGCTTCTACAAAAAGTTTCTTTACGAACCATTTCCAGTTGAGAGTAGTCTGAGGGAGCATTTGCATGATCACATCAATGCTGAGATAGTTTCTGGCACTATTTGCCATAAGGAAGATGCTGTGCATTATCTCACCTGGACTTACTTATTCCGCAGACTGACGGTAAATCCAGCTTACTACGGCTTAGAGGATACAGAGCCTGGTATTGTGAGTTCTTACTTGTCAAGGCTAGTGCAGGATACATTTGAGGATCTGGAAGACAGTGGATGCATCAAGATGAATGAAGATAGTGTTGAGTCTATGATGTTAGGATCATTAGCATCTCAGTATTACCTTAGTTACAAGACTCTATCGATGTTTGGTTCAAACATTGGCCCAGATACGACACTTGAAGTTTTCCTGCATATTCTGTCTGGTGTTTCTGAATATGATGAGCTTCCTGTGAGGCATAATGAGGAAAATTACAATGAAGCATTGTCTGCGAAAGTTCGCTATATGGTGGATAAGAATCGTCTTGATGATCCACATATTAAAGCAAATTTGCTTTTTCAGGCACACTTTTCACAGTTAGAATTGCCAATCAGCGACTACATTACAGACTTGAAGTCAGTCTTGGATCAAAGTATACGCATCATCCAAGCCATGATTGATATATGCGCTAATAGTGGATGGCTTTTAAGTAGTATTACTTGTATGCATCTTATGCAAATGGTGATGCAGGGTTTGTGGTTTGATGAGGATTCCGCTCTGTGGATGTTGCCGTGCATGAGTGTTGATCTTTTGGCTTCTCTAAGAAAGAGAGGAATATCAAGTGTAGGACAACTATTTGAAATTCCCAAGTCAACTTTGCAGGGTATAATCGGAAGTTTTCCTGTTTCAAGATTATATCAGGATCTACAGCATTTCCCTCGTGTTCAAGTGAGATTAAGACTAGAGAAGAGGGACACTGATGATGGGAAGTTTCCCTCTATCAACATAAGGCTGGAGAAGAAAAACTCGCAAAGAAAAACGTCGAGAGCTTTTGTTCCTCGATTCCCTAAGGTGAAAGACGAAGCTTGGTGGTTGGTTCTCGGTAACAGCTTTACTTCCGAACTCTATGCTCTGAAGAGAGTTTCATTCTCCGACCGATTGATCACTCATATGGAGTTGCCTTCAACTTTAACCACTCTCCAGGGCGTGAAGCTGATTTTAGTTTCGGATTGTTATATTGGGTTTGAACAAGAACACTCCATGGAACAACTTTTTGAACCTCGACAATCGGAAGGGGGAAGGCGATAAGAGAATTGATTAAGAGCCAGAACAAGCATTAATCAACTCCATTTTTTTTTGTCAGGGACACCTTGTATATGATATCTCATTGAAATTTTGTGCACTATCATCGTTAACGAGTTATATAATATAATGCAAATATATGATTTCCCAAAAAAAAAAAAAAAAAAAAAAAAAAAAAAAAA

>transcript_55 full_length_coverage=2;length=6536

GGGGTTCCATAAACCTCTCTTTTGCTCTACACACTTCTTCTATCGTCTATTTTTCACTTCCTCGCTGCTCTATAAGCTTTCTATATTCTTCGATTTCGCCCACCTGAAAAAAAGAAAAAAAATTCAATTTCTGTTTGTTTCTCGTGAAGACGGACTCTTTTTATCTTCTTCTATTTTGCCCATCTGAATAGATTTCGATTTCTGTTTGTTTCTCGTGAAGATCGACTATTTCTATCAGCTGAACAAATATCTGTTTGTTTCTCTCCAATCCTGACTCCTCCCGTTTGTTTGCTTTATCATATATTTCATTTGAAACGCCTTTGATTTATTTCGATCTACTCATTTCCGGCTGTTCGAGCTAATTGTTCCGAGCTACCTAGAACGATGGCACAGTTTCAGAACGATCTTGAGTATATGATCGATGATCACTACGACATGGCTGATTTTGAAGAAAACCCCTCTGTTGAGAACGAACCTCAGCTGAATGGTGATCAGTCTCTAGATTCGGACTTTGAGGACGACTTTGAGATGAGTAAGCCAAAGACTGATACTTCTGCCCTGGAAGCTCGAAATGGGAAAGATATTCAGGGAATTCCTTGGGAGAGGTTTAATTTTACTAGGGATTATTACCGTGAGACAAGATTAAAGCAATACAAAAATTACGAGAGCCTGCCGCGGTCTCGTGAAGAGCTCGAAAAGGAGTGCACGCAGGTAGTGAAGGGCAGCACCTTCTATGACTTTCAATTCAACACGAGGCTTGTCAAATCAACAATTGTGCATTTTCAGGTAGAGACTATATTTTCTACTGCTTTATGGTTCTGTTAAGTTTTTGAAGGCTTGAGATCATTTTCTACTGCTTTACTTTAGAAAATGATTTGAAGGGACAACCTTTTTCTCTAACTCTGACAAGAACCAACTCCCTACTCCAGTTTTACTTCATATTCATGTCATATTCAAATATATTTTTTTTCAATATATTCATATTCATGGTCAAGTTGTAGGCTCTCTTTTTCTAACTACTCTGCATGTTCATGTTACACGGTCTGACCTCTGAAACTTAGATCTTTTAGATGTCGACTTGGGAGTTAACAATAGTCTAGTCTTTATTATGACTGATTTCGATTTAGGCTATGCATATATCATAAGCTTGTAGTTTTGCAGGTCTTGTGAATATGTTATTTGTTCTCTATCGATTTCTATATATATGATTGTTGATCTATCCCCAACCAATTCACTGGAAGGATATCTATATATACGATTCTTGCTCTGTGCCCCAACCATTCACTGGAAGGTATGGAGTTTGTAGTTGTTGCCTTTTATTTTAGGGTTTATTAGTTCTTTTCATTTAATTGTGATAATATAATAATCTTATCGGTTGCTGACTTTAGTTTGAGGAGTGTTCCAGTTGTCCTTTGGGCATGTGAAATGGTCATTCAAATCTCCAGCCTCATGTGGAGATATTTATTTTGCAATTTAACTTAATTTTTTGTCAACCTTTGGCCGTGTGAAATGGTCATTCAAACTTCCAGCCTTGTGTGGATAAATATGCAATTTAACTTAATTTTTTGTTAACTGTTCAGTAGTACTCATGTATTGGGTTGTTTATCTTTTAGTTTGGTCACCCAAAGTTACTTAATAGTTAATTATGTTAATTTGCTCAGCTGAATGATCTGCAGCTGGGTTCTGTATTCACCTGATTGTTTTTGTGCAGATTTGTGAAGGCTGCATACTTTCTACTTGCACTTAATAAATTTCACCATTTTTTTTTAAAAACTAACCGATGATTTCTGAAGGTAGTATCATGGAATATGTCTGATGATCTTCTCTACATCTGATATGCAGTTATTATTATATATGTGTTGTCCTGTTAATCAGTCTCTGCAACCCATTATGATCTGGGATCGCTGCAAAAATAGATTGTAATTTTACCATTTCCTTAAAAAAACTAACTGATGATTTCTGAAGGAGTAGTGTCATGGAATATGTCAGATGATCTTCTCTACATCTGATATGCAGTTATTATTATGTACATGTTATCCCGTTAATCAGTCTCTGCAACCTATCATGATCTAGGGTCGCTGTAAAAATAGATTGTAGTATGTGTTTAAGAAATTTTATTACACCTACAGGTAACCTCGACATGTTCCAGACACAAAAATATAGCAGTCATTATCTATGTTAGATTTTGGCAGTCTTTGAGTCATTGGCATTTTAGAAGCATGGTGGTTCCTCATTGTTTTTATTTATATAATTCATCGTTTCATGTTAGGCCCTCGTGTGGAGATATTTATTTTGTAATTTAACTTAATTTTTTGTTAACCTTTGGGCATGTGAAATGGTCATTCAAAATTTCCAGCCTCGTGTGGAGTTATTTATTTTGCATTTTAACATAATTTTTTGTAACCGTTCAGTAGTACTCTTGTAGTGGGTTGCTTATCTTTTAGTTTAGTTAGGCAACCAAAAGTTAGTTAATAATTAATTATGTTAATTTGCTCAGCTGAATGATCTGCAGCTGGGTTCTGTATTTACCAGATTGTTTTTGTGCAGATTTGTGAAGGCTGCACACTTTCTACTTGCACTTGATAAATTTTATCATTTTCTTAAATAAACCCTAACCTATGATTTCTGATAATATCATGGAATATGTCAGATAATCTTTTCTACATCTGATATGCAGTTATTATTATATACGTGTTATCCGTTAATCAGTCTCTGCAACCCATTGTGATCTAGGATCGCTGCGAAACTGGATTGTAGTATGTGTTTATGAAATTTTATTACACCTGCAGGCAACGTGGACATGTTCCAGACACAAAAATATAGTCAGTCATTATCTATGTTATATGTTACCAGTCTTTGAGTCGTTGGGATTTTAGCAGCACTGGTAATTCCTCAATTGTTATTTACATAATTCATCGTTTCATGTTAGAGCCTTTACAGTGCAGCATAATAGCTTAACCGACCAGTGATACTAGTCAAATGAAATATGATACTTACCCTCCGGTTTGTTTTGCTGGTATATTGGACATTACGGTATTGCAGACAATGAGTACGTATTCTGGTCTTTTATGTTTTCATTTTAGTAACTGACCAGTGTAGTTTTTTCGCTCCTGGAATTTGAAGAGGTTCTATTGTTAGTTCATAGATGGTGCAAGCACTAATCTAACTCTTCGATATGTCCTCCGGTAGTAATTGTATATTTTCTGTTGCTGTATGATGTCTTGAGATTCTTAACCCCTCTTTGGCATTTAGGATTGCCAGTTTTCTTATTTCAGCCCTAAAACAGGTTTGGCGAGCAGCTGGCGCAATGAGGACGTTGTCATATGGTATGATAGGAAAATATTTGAGCATTTTAGAGGCATCTGTGGAAATTCCTGAATTACGTAGATTTGTTCTGTCCTGCTCATAGGAGGGCTAATCTAACAATGTTTACACTTGGCATGTTGAGATGTTACCAGAATAATCTGATAGCGTCTCTATTGCAGCATCTTTTATTATTTTTAAATTTTTATTATTATTTTATTTGGATGTGATCTATTATTTATTTGGATGTGATTTATTATCTTTTGAAATTTCATGCTTTATTTATTTTTAATTTTTTTAAGGTTGATTGCTTCTAGTCTCATCTTACATTTGCCTTTTATATTTAAAAAATTGAGGACCGAGATTTTTGTATTTGGGTTCAACTGGCTGTTGGTACGGGTTGACATTAGGAGTAGCAATTTGTGTGTTTTGGACTGCCACAACATGAATATATGGTTATGTTGTTTTGGTCTGACCTGAACAAGACCATTCATAAAACAGGTGATGAACACAAAATTGACACCAAATAAAAATATGACATTGAACACAAAATTGTCTTTCTTGTCACCCCTATCTACACAACTTTAAACTTGTCCCAATACGATTGTTGCAACCTCCCTATTTTAACAGGATAGACCTGAATGTTTATTTTTCAATCGTTAGTTTTTCATTGACTTTTGAACCAAGTCTGAGTTGGTCTGGATTTCAAGTTTTATTTTCTTTTTCTTTTTATTTCTTCTAGCTAAAAAGAACAACTAGCTAAGAAGCTAAAAGGGTCTCATTTATTCCCGGGAATTTACATTGTCATGGGAGATATTTTTTGTTTTTATATTCAAAAATCTCTGTTTATTTAATTTATCTTTCTAGTGTTGTAACTTCATTTTACGTCTAGTTTGACTATATTAAATATCATTTAGTTAAAATACTGCACAATTGAGACAGTACCTTATTTATCAGTTCTAGTTCATGAGTCGCAAGAGTATCGGATTATTATCCTCATTTTTTTGTTTGTTATTAGTGCTATATATGATGCACATATTTAAGTTGCTGCAGTTCTCCACTATCTTGTTTGGAGTCTTCAGCAATTGAGAATGGCTTGTGTTGTATACTATTTGGAAGGTGTTTTTTTTTTCCTTTTTACACGCATGCGCTTTGATTATGTGTTACAAAAGTAGGATTGCATCTTCTTATTCTTTCCCGAGGTTACTTTATACTGACATTGTCACTCCCTACAAATGAATTACCGAGTTACTAGTTTACCTGATCAAATAAATTATCAGAATGGGATGACACTTGATTTGTGGGTTGAGCTGATGATGAGAACTTGTACGCTCCAATAATATTAGCTGAAATCCAGTTCATCATTTTGTTTGCAACGTCGATTCTTCATTTCCCTTGTTGGTTTTGACTTGTACTGCCTAATCAATGATAACACTTGGATCGTGGGTCGAGCTGATGAGAAACTTGTATGCGCCAATAAATATTAGCTGAAATCTAGTTCCTCATTTTGTTGGCTACATTGTTTCTTTTTATCCCTTGTTGGTTTTGACTTGTAATGCGTATTCAATGTGTGCTGTCACTGCAGTGCAATGTCAAAAGCTTCTTCCAATTAAGCCCCTCTTCACTTTAGATCTTATTAGTAGATTAATGTACGCACTAACTCCTTGTTGGCCAGTTTTCCAATGTTTAGGAGAGAGTGATTTTGGAATAGTGTAATTAACTGGAATTATTCTCAAATGAAATAGTAATATTTGTTTATTTGAACATGGAGGATGATTTTTTATATTTTGTTACCTGTCTTTCTCTGCTTTCATACTTGTGTTAATTTGCATTATTACAGAGTTAAGTGGTGATATTAATGTGCCAAGCTATTGGTGTTATTTTCTTTTGCAGCTAAGGAATTTGCTATGGGCAACATCAAAGCATGACGTATACCTTCTGCAGAACTACTCGGTAATGCACTGGTCATCTATGCTCAAAAGGAGTAGGGAAGTACTCAACGTGGCTAAACCGACTGTACCCACCTTGAAGTATCCAGGTTCTTCAGCTCATCCGCTTCCAAGGGTGCAGATAAGCACCATGGCTATTAAAGACAATTTAATGGTGGCAGGTGGCTTTCAAGGGGAGCTTATGTGCAAGCATCTAAATCAATCTGGAGTTGCATTCTGCACAAAAATTTCGACTGAAGGGAATGCCATCACCAACGCGGTGGATATATACCATAACCCCAATGGTTCGATGAGGGTCATGACTGCAAACAATGATGCAGCAGTTAGGATTTTTGATGCCGAGAAATTCGCTTGTCTTAATCGTTTCTCTTTTGATTGGTCTGTAAATAACACTTCTGTTAGTCCCGATGGCAAGTTAGCTGCAGTCCTTGGGGACAGTACAGATTGCTTGCTTGCTGATGCTCTTTCTGGGAAAGTTGTGGGTAACCTCAAAGGGCACTTGGATTACTCATTTGCCTCAGCTTGGCACCCCAATGGACACATTCTGGCTACTGGGAACCAAGACAAAACTTGCAGGTTATGGGACATAAGAAAGCTGACCGAGTCAGTAGCTGTACTGAAGGGAAGGATGGGGGCTATAAGAGGAATAAAGTTTACCTCTGATGGCCGGTTTATGGCTATGGCTGAGCCTGCAGACTTTGTTCATATATTTGATACAGAGTCTGGGTATGTCAAGGCACAAGAGATTGACATGTTTGGTGAGATTGGAGGAATATCATTTAGCCCAGATACAGAAGCGCTATTTGTTGGGATTGCAGATCGTACTTATGGTAGCTTATTGGAGTTCACCAAGAAGCATTATAATGGATATTTGGATTCAATCCTGTAGGTTGTGTTGTATATGTCCGGGGGGAAGATAAGTTTTGGTTGATGTAGAGGCTGTGCTGTGTGTGTGCTTTGTATATTAGGACGACTCGTGGCTAATTAATTAATTTAGTCATTTTGGTTTTCCTACTTTTTTTTTTCCTCGTTGGGTTGAACTTGTATACATGAATGTATAATCAAAGAGTTATTAGTCAAGGTGTTCACACAATCACACAAGGCTATCTGGGATTGTGCCGTGTAAAAGATGAATTGGGATCCTTGACTAAAAGGATTGTGTATATCCAATGTAGCGTCGTAATCCGTCCGCTTTGGTTGGATTTTTTTTTGTTCTAAATCAAACTAATTTGAATATAAATATTTTGGTTCAAAAAAAAAAAAAAAAAAAAAAAAAAAAAAAGT

>transcript_56 full_length_coverage=2;length=6519

GGGACCCTAAATTTTCTCGGGAAAGTTCTATATATTGCCTTCTAACCACATAAGGAAGTTCTGATTCATGTCTGTATAATATACACTATCTCCGGCTTACTCAGTTGAATTCTTGTTGTTAATACAGATTGTTTTCCAACTTTTCAACATCTTTTTCCCTCAATTCCTAAGCTTTTTAGCGTCATTCTTAGGGTTTGTTTGGGGTTTCAAATTTTTTCGTGAGGTCTGTAATTAAACGATTCGATTTCGGCTTCTGGAATCGTTTTCAGTTCAAAATCCGAGCTTGACTCCGGGAAGTCGTCAACTGTACTTGCTGGTCAATTCGATCTTTATTGTAGGGAAATTGTATAATCTGCTTCTTCACTTTTTTTTTTAATTAGTGAGGATTTTTCGTTCACTAAGTGTGGATTGTTCAGTAGACGGCGATTCAGTGGTTTATTAGTTTTTTAGACAATGACAATCGAGTCCTTACCGGGAAGTTCAGGCTATTTAGAGTCCTACTCCGGGAAGAAGATAACCTATTTCAGTAACGCATACGTTCTCGGATTAACTGTTGTTGCCGGCATCGGAGGACTGCTGTTTGGCTATGATACAGGTGTAATATCAGGGGCCCTTCTATACATCAAGGATGATTTTGAGGTAGTCGGCCAGAGTTATTTCCTGCAGGAAACAATTGTCAGCATGGCCTTGGTTGGTGCAATCATTGGTGCTGCAGCAGGGGGTTTGATTAATGATGGCTATGGACGCAAGAGAGCTACACTTCTTGCTGATATTGTCTTTACAGTTGGATCGTTTGTCATGGCCGCTGCACCAGATCCTTACTTTCTTATATTTGGACGATTTTTGGTTGGTCTGGGTGTCGGAGTAGCTTCTGTTACTGCTCCTGTATATATTGCAGAAGCATCCCCATCGGAAATAAGGGGAGGTTTAGTCAGCACAAATGTGCTTATGATTACTGGTGGACAGTTTCTTTCCTACCTGGTGAATCTTGCCTTTACAGAGGTCAGATTTGTTATTCTTATTATGCTTTTATCATTTTGAAGTATATACTTTTGATATAGCTTGACATTCCGCTAAGCTTGTAGAGAACATGGAAAATATGATATTCCCACCCCCTCAAAACTTTCATTTGATTTCTATTTTCGTATGTTGTTTTTTATTTTTATTTGAAATGAATTTAAAGTAGGCTTTTTAGTTTATATTTTTAATTTGGGAAGAGTGCTTAAAAACTTTGGATTGGTAAAGCTACGCGAATGTACATGACTGAAAATATACCAACCTACGTAATTGTGGCAAAACTTCTGCAAGAGGATGGGCAACTAGAGATAGGTCTCATGTTATCTGCTCAAGTCAGATTTGCATTATTATTCTATGATCTTTATATAGGTTACCTTGAGAATCCAGAAGATGATTTTACCTGTATTGTGCTTTAATGGACGGGTTAGGAGTACTTTGGAATGGTACTGTTGGATCTGGAAAAACTAGAAGTAACTGTCAAACTTTAAGACAATTGTCTCTGAACCAAATTAAAATTTCATTTTCTTTACAGATGAACTTTTTTTTTTTTTTAATTTTTATTTACTGCTTTAAATTTTGTTTTCTTTTTGGGTTTTATTTATTTATTTATTTGAGTAGGGGTTTGAATTTCTTTTTGTATCAATGTATTTAGCCTTTTACTAAAAAAGTTGGGTATGCTCTTATCTAATGTGATGTTTCAAAACCTAAACCCAGCTGGGTTCCAAACCAGGTCTATAAAATTCTGAGTTTCGGGCAATTATACAGGAAAGTAGAGTGGGTAATCTGCTCAACCCATTCTGCTGGCATCTCTAATGAATTATGTCTTTAATGGATTAGGTTTGATAATTATTATGAAGTAAACAACTAATTTCAAAATTTTGACAAGGTTTATCACCATTCTGTTTTTTTAGTATAGAATAATTATGATTATTTGTAATTTCTCAATCATTGGGTTAATATGTTGGTTCAGGTTGACTTGATCAATTGGTTGGTTTTGATTTTTTGAATTCTATCATTGGGTTGATATTTTGGTACAGGTTGATTTGATCAATCGGTAAGTTTTGTTCATTTGAAAATAACCAGTTGACTGCTTTGCTCCCATGAACGTACTGAGTGAACTCGTGGCAAAAAATGTAGTCTCATGTTTCCCCCCATATTAGTTCATTTGGTAAAGGTGAAAAAATTAGAGGAATGGGGATAGCAGTCCCTTTCCCTGGATATTAACGTGTTTGATATTTTTTACGTTTCCCCCCATATTAGTTCATTTGGCAAAGGTGGAAAAATTAGAGGAATGGGGATAGCAGTCCCTTTCCCTGGATATAAACGTGTTTGATAGTTTGATATTTTTTACCACTGGGGAATGGGGGATAAACTCTCCAAATTTGGGTATAACAAGACCCTATTTATTCCCTCTATTTTAGACGGGAAAGGGGATAGAAATAAGGGGATAAACATAGATTTTTTTTTTAAAACATATTACCCTGATTATTGTTAAAAAAACAATTTTTCCATCGATAGATATATTTTATAAAAATAAGTTAGGGGTAAAATATTATTTTTTGTATTTATCCTATTTTTCTTGATTGTTCTACCAAACAAAGGAGGGATAGTGTAGGCCTTATATTAGCTTTTTCCCTAAATTTACCAAACATAAATTTAGGCATAGTTATCCGGGTCATGCTATCACCTTTCCCTGTTCCTCATTCCCCTAATTTTTTTCACCTTTGCCAAACATAGCGTAAGCTCCTTAATCGACACAGTTTCTCTCAAAACATTTGTACTGATGATTTTTTTGTTAAACAAAACATGATAATATCAAGTATTATTTGGATCAAAATAAAATATATACGTACTGCTTTTCAAACCTCCCAAATCAAATGATTAACCATGTAGCAATTGACTTTCAACTAAGGATTAAATGGGAAGGTAATAGTAGTGTTTTTAATAATAAAAAAGAGTTTTCTAGAATTGTTTATATATATTTTAACATACTCTGTACTATGTATTACATACATTCTTAGTAAAATTCTTCTTTGTTGGTGGAGTATGCGTGTTCTTTAAAATCCTCCTTAGTAAGAGTTCTGAAGCATTTTGTGATCCGAAATTACAAAGCCTAAGCCTACTCACACAGGCTAAAGCAACGTACCACATACCAGAGTGGCCTGCGTTTCAGGTAATAATGGTACAACAACATTTACAGGCCTTCATTTCACCTGAGTAGATTTGCCCTAATTGCATGAGGGAACTTTATAAAATGAATAGTGTGGAGACATGGCTTATTTGGAAACCAAGATTTTGACTGGGAATCTGATTCCTGAATAAATCCTGTTTGGTATATGCCCTTTTCAAAATTGATTTCGGCCCATGAATTTGAGAATCCTCAGAAATGTGGATTGGTTTTGATTGTGATGATTAAAATTGATTTGGTAAAATCAGTCTTGAATTCATTTGTTGATTCTGATTCCAATCTAAATCAAAATCTGTGTTTCCAAATAAGCCCTTAATAGATTTTTGATAGAAGTTAATATGGCATGAGTTTCTCTTACACTTCTTTTCCCCTTTCTTCCAATTTGTATCAAATTCTCAATTCATGGAGTAGAATTATCTTTGGCATCAATCAGTTCAAGAGATCAAATACTGGGGTAATAGCTTTTCAGTTGATGCAGAGGACATTGTTGGAGAAATTTGTGTTACATACATGCTCATTGCAAATTCATTATTTATTTTATTTTATTTTTTGGCATGAAAGGTTTTTCATTGTGCAGTTTCGAGGAGGCAGCATCAAGATAGAATATGGACTTGCGTCTTAACATTTTGCAATGAGAAACGTCAATGAAGGAAAATTCTGTTCTATTCTGCTTCTTATAGATTTTGCACGATGCTATTGTTGTCGATAGCATGTGATAGATTCCTTGGCTTCAGTAAATTTAATAGTAGTTTATAACATGTGATAAATCAAAATTACATGGTATGGACATTTTCTTTGTGTTGGTGTTGCTTTAGACGGTTGGTTCCTTCATCGGTAGTAACAGTTGCGGGTTGGCGGCTCCATCTCAATGACCTCGAAGCAATTAAAACATCTTAGGCAACCTACACCTTTTACAGTTTACCTCTGTTGATAATATGGAAGACCATTTTTAATTGGGAATTGTAGATTTTCACTTTAAAGTCCTTACGAATACAGTTTTGACTATTTTATCTTTACTGTGCATTTGGTGCTGATGTTGTCATTCTTAACCTTTTCGGAAGACCCAGTGATTACAAAAGAGAAGTCATCCTTCAAAGGCGCAACACAGTAAACTTACCAGAAAAACAATAGATAGCAACAACCTATGGTCACGAAACCAGGTTAGACTAATCAAATGGCAAACAAGAACTGGGACCCCCATAACTGAGACTGAGCCAACTTCTACCCACCCCTTGTTTGGATCTTTACCATTGTAGTTGATGGTGGAATGAAGATTCTTTGAACAACTTTGACGCCTTAGAACCCTTGAGCTTCATCTGAAGAGAAACATCTGTTGGGATTTCCAATGATCTATGACCGAGGCAAAAATTAAACGAGTAGTGGCACATGATGTCCAGGAGGTTCTGTCTGAAATACTTTTTAGTGAGGCGAGAGATCCATAATGGCTGCTGCGCATTGGTTTTGCACACATTCGTGGTGACTGAGCTGAAGATACTGTCTAATGAAGGGTATGCTTGAGGTTACTCCCCAACTCATCTTAGGACTTCTAAAAGCCTAAATAGAGGATCATAATAGACTTTGAAGGGGGGGGATCATGTTACTTAAACGCAGAGGATACGCATGGGAAATCACTTAGGTTTTTGAGAAATCCATGTTCCGTCAGGTTGGATGGATATTGAGGGGTGATCAATTCAGACAATATTATTATATACTCACCCATGGGCCCCGGCTTCTTGTTGGACGCGGGTTGAGTAGGTGGTTCATATGAACTAGCCTGCAGCACCCTAGGATCTCGAATCTTCATTTCTAAATAGTCCTCTTCATTTGCATCTGGTGAAACAGTAAGATTTAAATTCATATATTGTTGTCGGCATTTGCATACGGTGGTACAATAGGATTTTTTTCCTTCTTGTTTTGGCTTTTGATTCTTCATTTGCATCCGGTGAAAAGTAGGATTTAAATTTCATGTTCTGATAAATTTCATTTTGTAATTTGCAAAACGAGAGTTTCATGATCCAACACGCGTTTACACCATATTCTTCATTTTATAGTAATTGTGTTTTTTTTTGGTTAAATGTTGTTTCTTGTACTTTATTTTTGAAAAACTTTGAATCTTTGTACAGGTCCCAGGGACATGGCGATGGATGCTTGGAGTTTCTGCGGTCCCAGCAATAATCCAGTTTGCTCTTATGCTCTGTTTGCCCGAGTCTCCTCGCTGGCTTTACATGAAGAACGAGAAATCAAGAGCTATTTCCGTGCTTTCGAAAATATATGACCCTGATCGGTTAGAAGAGGAGATTGACCAGCTTGCTTCTGCATTAGAAGAAGAAAACCAGAGGAAGAATGCTATCAGATACACAGATGTTTTTAAAAAGAAAGAAATCAGACTTGCTTTTCTAGCTGGTGCTGGACTCCAGGCATTCCAACAATTCACTGGTATCAACACAGTCATGTACTACAGCCCAACGATCGTCCAGTTAGCTGGCTTCCATTCCAATCAATTAGCACTTCTCCTATCCCTCATTGTTGCTGCAACGAATGCAGCCGGAACAGTTGTCGGAATTAACCTTATCGACCATTTTGGCCGGAGAAAGTTAGCCCTCACGAGCTTATTTGGCGTGATCATCTCCCTTGTCATCCTTTCCGGATCTTTCTTCCTCGAAGCATCTGGTTCTAATGACACACTCTATGGGTGGATGGCGGTTATAGGACTGGCTTTGTACATTGCTTCGTTTTCACCTGGAATGGGACCTGTGCCATGGACTGTGAACTCGGAGGTTTATCCAGAGGCGTATCGTGGAATGTGTGGTGGCATGTCGGCTACGGTTAATTGGATTTCGAATCTGATTGTGGCACAGAGTTTTCTTTCGATTGCTGAAGCGGTGGGGACTGGTGCAACTTTTGCTATACTTGCCGTTGTGGCAGTGATTGCTTTTGTGTTTGTGATGGTATACTTGCCGGAGACCAAGGGGCTGGCGTTTGAGGAAGTGGAGAAGATTTGGAAGGTTAGGGCTTGGGGGACCGAGGAAAACACTCAAAGTCTTCTTGAGACGGGGAACCAGTAGTTACGGGTTTCATGTTTTTCTTTATTTGTATGGAATTCTGTTGAATTTAACTGTCTTGGAATCATTATGTTGAGCTGGCCATGCCCTCAATCTGATCAAATGCATGTGTGCTCTAATTTGGTCCGTTCATTTTACAAAAAGGTACACTTGATATCGCTAATAGTTAAAAAATTAGGCTGTCGACCTTTAAATAAATTATGCACTCTTTTACGTGTCAAAAAAAAAAAAAAAAAAAAAAAAAAAAAAAGT

>transcript_57 full_length_coverage=3;length=6506

GGGTTCGCGACTAGCGGTGCTGTTGTGGTTCCACTGGATGCTTCTGCGAGCTTGTTGGACAATTCGTTGTCGAACGAGTCGTGGTACGTTTGGTGTTGTCGTGGCAGCGTTCGTGATGGCGTGTGAGTGGTATTTAGATTGCGTGGGTTGGTAGGCTCCCTGCTAGTGCAGCGAACTATCGGCCCGGGCATCTCTTCAGCGTTGTCTCAAGTGCTTGCTTGCCTTGGGCGGTGTCTTGGTTCCTGTGTTGCCTACCTACAAAAGGGTATTTGTTCCAATCGCTGATCCACATCCGTCGAGTGCCCCTTCGGGTGGCACTGGGCGGACGACGAGGTATTGCATGTGTTCCAAGCTCGCGTCGCCTCGTTGCGTCGCTTGTCTGGCCCTTGTGTTGATACTTGTTCTCTCGGATGCGGAAGCTTGTGAGGGTAGGGGTCTCGGCCTTGTACACCCAAAACGAGCGCTCGTCGCTTGATACGTACGACAGTCGTGCTCGTGTTGACCCTGTTATCAATTTTGTTGGCAGTGCTCATGCGGTGCCGACGTCGCACAGGAATGCTACCTGGTTGATCCTGCCAGTAGTCATATGCTTGTCTCAAAGATTAAGCCATGCATGTGTAAGTATGAACTAATTCAGACTGTGAAACTGCGAATGGCTCATTAAATCAGTTATAGTTTGTTTGATGGTACCTGCTACTCGGATAACCGTAGTAATTCTAGAGCTAATACGTGCAACAAACCCCGACTTCTGGAAGGGATGCATTTATTAGATAAAAGGCCAACGCGGGCTTTGCCCGTTGCTCTGGTGATTCATGATAACTCGACGGATCGCACGGCCATCGTGCCGGCGACGCATCATTCAAATTTCTGCCCTATCAACTTTCGATGGTAGGATAGTGGCCTACCATGGTGGTGACGGGTGACGGAGAATTAGGGTTCGATTCCGGAGAGGGAGCCTGAGAAACGGCTACCACATCCAAGGAAGGCAGCAGGCGCGCAAATTACCCAATCCTGACACGGGGAGGTAGTGACAATAAATAACAATACCGGGCTCTTTGAGTCTGGTAATTGGAATGAGTACAATCTAAATCCCTTAACGAGGATCCATTGGAGGGCAAGTCTGGTGCCAGCAGCCGCGGTAATTCCAGCTCCAATAGCGTATATTTAAGTTGTTGCAGTTAAAAAGCTCGTAGTTGGACCTTGGGTCGGGACGACCGGTCCGCCTTCAGGTGTGCACCGGTCGTCTTGCCCCTTCTACCGGCGATGCGCACCTGGCCTTAACTGGCCGGGACGTGCCTCCGGTGCTGTTACTTTGAAGAAATTAGAGTGCTCAAAGCAAGCCATCGCTCTGGATACATTAGCATGGGATAACATCATAGGATTTCGGTCCTATTCTGTTGGCCTTCGGGATCGGAGTAATGATTAACAGGGACAGTCGGGGGCATTCGTATTTCATAGTCAGAGGTGAAATTCTTGGATTTATGAAAGACGAACAACTGCGAAAGCATTTGCCAAGGATGTTTTCATTAATCAAGAACGAAAGTTGGGGGCTCGAAGACGATCAGATACCGTCCTAGTCTCAACCATAAACGATGCCGACCAGGGATCAGCGGATGTTGCTTATAGGACTCCGCTGGCACCTTATGAGAAATCAAAGTTTTTGGGTTCCGGGGGGAGTATGGTCGCAAGGCTGAAACTTAAAGGAATTGACGGAAGGGCACCACCAGGAGTGGAGCCTGCGGCTTAATTTGACTCAACACGGGGAAACTTACCAGGTCCAGACATAGTAAGGATTGACAGACTGAGAGCTCTTTCTTGATTCTATGGGTGGTGGTGCATGGCCGTTCTTAGTTGGTGGAGCGATTTGTCTGGTTAATTCCGTTAACGAACGAGACCTCAGCCTGCTAACTAGCTATGTGGAGTTTGTCCCTCCACGGCCAGCTTCTTAGAGGGACTATGGCGATTTAAGGCCACGGAAGTTTGAGGCAATAACAGGTCTGTGATGCCCTTAGATGTTCTGGGCCGCACGCGCGCTACACTGATGTATTCAACGAGTCTATAACCTTGGCCGACAGGCCCGGGTAATCTTCGAAATTTCATCGTGATGGGGATAGATCATTGCAATTGTTGGTCTTCAACGAGGAATTCCTAGTAAGCGCGAGTCATCAGCTCGCGTTGACTACGTCCCTGCCCTTTGTACACACCGCCCGTCGCTCCTACCGATTGAATGGTCCGGTGAAGTGTTCGGATCGCGGCGACGTGGGCGGTTCGCCGCTCGCGACGTCGCGAGAAGTCCACTGAACCTTATCATTTAGAGGAAGGAGAAGTCGTAACAAGGTTTCCGTAGGTGAACCTGCGGAAGGATCATTGTCGAACCTGCCTAGCAGAACGACCAGCGAACTTGTAAAAATGCTCGGGATGACGGAAGGCGTGAGCCTCTCCTCCATCCCATGTCCGGTCGCGCCAGGCGTTGAGTCTCCCCTCGCACGATGTGCTGGGAAGCGCCAAGGTTCTGGTGTGCTCTCGGATTTACAACAACCCCCGGCGCAAACCGCGCCAAGGAACTAAAACGAAAGAGCATGCCCCCGTTGCCCCGGCTTTGGGATGCGCGGGAGGTAATGTCTTCTTTTACATATCAAAACGACTCTCGGCAACGGATATCTCGGCTCTCGCATCGATGAAGAACGTAGCGAAATGCGATACTTGGTGTGAATTGCAGAATCCCGTGAATCACCGAGTCTTTGAACGCAAGTTGCGCCCAAAGCCTTTAGGCTGAGGGCACGTCTGCCTGGGCGTCACGTATCCCGTCGCACCCCGAACCCGTCCCAACTCGGGCATGATGGCTGGTGGGAGCGGATATTGGCCTCCCGTGTACTCGCGTTACGGTTGGTCTAAAATTGAGCCCCGAGCGACGAACGTCACGACAAGTGGTGGTCTGTAATAGCTATTTCGTGTTGTGCGTTGTCTCGTCGCCCGTGGGAGCTCACAAAGACCCCAAAGCATCGTCACGATGATGCATCCATCGCGACCCCAGGTCAGGCGGGACTACCCGCTGAATTTAAGCATATCAATAAGCGGAGGAAAAGAAACTTACAAGGATTCCCCTAGTAACGGCGAGCGAACCGGGAAGAGCCCAGCTTGAAAATCGGACGGCTTCGCCGTTCGAATTGTAGTCTGGAGAAGCGTCCTCAGCGACGGACCGGGCCCAAGTCCCCTGGAAGGGGGCGCCGGAGAGGGTGAGAGCCCCGTCGTGCCCGGACCCTGTCGCACTACGAGGCGCTGTCGACGAGTCGGGTTGTTTGGGAATGCAGCCCCAATCGGGCGGTAAATTCCGTCCAAGGCTAAATATTGGCGAGAGACCGATAGCAAACAAGTACCGCGAGGGAAAGATGAAAAGGACTTTGAAAAGAGAGTCAAAGAGTGCTTGAAATTGTCGGGAGGGAAGCGGATGGGGGCCGGCGATGCGCTCCGGTCGGATGTGGAACGGTGAGAGCCGGTCCACCAATCGACTCGGATCGTGGACCGACGCGGATTGCGGCGGCGACCCAAGCCCGGGATATAGATTCTATGCCCGCGGAGATGTCGTTGCCGCGATCGTGGATAGCAGCGCGCGCCTTCACGGCGTGCTCCGGCACCTGCGCGCTACTGGCGTCGGCCTGCGGGCTCCCCATTCGGCCCGTCTTGAAACACGGACCAAGGAGTCTGACATGTGTGCGAGTCAACGGGCCAGTAAACCCGTAAGGCGCAAGGAAACTGATTGACGAGATCCCCATAGCGGGTTGCAACGTCGACCGACCTTGATCTTTTGTGAAGGGTTCGAGTGAGAGCATACCTGTCGGGACCCGAAAGATGGTGAACTATGCCTGAGCGGGGCGAAGCCAGAGGAAACTCTGGTGGAGGCCCGCAGCGATACTGACGTGCAAATCGTTCGTCTGACTTGGGTATAGGGGCGAAAGACTAATCGAACCATCTAGTAGCTGGTTCCCTCCGAAGTTTCCCTCAGGATAGCTGGAGCCCACGTGCGAGTTCTATCAGGTAAAGCCAATGATTAGAGGCATCGGGGGCGCAACGCCCTCGACCTATTCTCAAACTTTAAATAGGTAGGACGGTACGGCTACTTTATTGAGCCGTGCCACGGAATCGAGAGCTCCAAGTGGGCCATTTTTGGTAAGCAGAACTGGCGATGCGGGATGAACCGGAAGCCGGGTTACGGTGCCCAACTGCGCGCTAACCTAGAACCCACAAAGGGTGTTGGTCGATTAAGACAGCAGGACGGTGGTCATGGAAGTCGAAATCCGCTAAGGAGTGTGTAACAACTCACCTGCCGAATCAACTAGCCCCGAAAATGGATGGCGCTTAAGCGCGCGACCTATACCCGGCCATTGGGGCAAGCGCCAGGCCCCAATGAGTAGGAGGGCGCGGCGGTCGCTGCAAAACCTGGGGCGCGAGCCTGGCGGAGCGGCCGTCGGTGCAGATCTTGGTGGTAGTAGCAAATATTCAAATGAGAACTTTGAAGGCCGAAGAGGGGAAAGGTTCCATGTGAACGGCACTTGCACATGGGTTAGTCGATCCTAAGAAACAGGGGAAGCCCGTCTGATAGCGCTTTTTGCGCCTACTTCGAAAGGGAATCGGGTTAAAATTCCTGAACCGGGACGTGGCGGTTGACGGCAACGTTAGGAAGTCCGGAGACGTCGGCGGGGGCCTCGGGAAGAGTTATCTTTTCTGTTTAACAGCCTGCCCACCCTGGAAACGACTCAGTCGGAGGTAGGGTCCAGCGGCTGGAAGAGCACCGCACGTCGCGTGGTGTCCGGTGCGCCCCCGGCGGCCCTTGAAAATCCGGAGGACCGAGTGCCGCTCACGCCCGGTCGTACTCATAACCGCATCAGGTCTCCAAGGTGAACAGCCTCTGGTCGATGGAACAATGTAGGCAAGGGAAGTCGGCAAAATGGATCCGTAACCTCGGGAAAAGGATTGGCTCTGAGGACTGGGCACGGGGGTCCCAGTTCCGAACCCGTCGGCTGTCGGTGGACTGCTCGAGCCGCTTCCGTGGCGAGAGCGGGTCGCCGCGTGCCGACCGGGGGACGGATTGGGAACGGCTCCTTCGGGGGCCTTCCCCGGGCGTCGAACAGTCGACTCAGAACTGGTACGGACAAGGGGAATCCGACTGTTTAATTAAAACAAAGCATTGCGATGGTCCCTGCGGATGCTGACGCAATGTGATTTCTGCCCAGTGCTCTGAATGTCAAAGTGAAGAAATTCAACCAAGCGCGGGTAAACGGCGGGAGTAACTATGACTCTCTTAAGGTAGCCAAATGCCTCGTCATCTAATTAGTGACGCGCATGAATGGATTAACGAGATTCCCACTGTCCCTGTCTACTATCCAGCGAAACCACAGCCAAGGGAACGGGCTTGGCAGAATCAGCGGGGAAAGAAGACCCTGTTGAGCTTGACTCTAGTCCGACTTTGTGAAATGACTTGAAGGGTGTAGGATAAGTGGGAGCCGGCAACGGCGAAAGTGAAATACCACTACTTTTAACGTTATTTTACTTATTCCGTGAATCGGAGACGGGGCTCTGCCCCTCTTTTTGGACCCAAGACTTGCTTCGGCAGGTCGATCCGGGCGGAAGACATTGTCAGGTGGGGAGTTTGGCTGGGGCGGCACATCTGTTAAAAGATAACGCAGGTGTCCTAAGATGAGCTCAACGAGAACAGAAATCTCGTGTGGAACAAAAGGGTAAAAGCTCGTTTGATTCTGATTTCCAGTACGAATACGAACCGTGAAAGCGTGGCCTATCGATCCTTTAGACCTTCGGAATTTGAAGCTAGAGGTGTCAGAAAAGTTACCACAGGGATAACTGGCTTGTGGCAGCCAAGCGTTCATAGCGACGTTGCTTTTTGATCCTTCGATGTCGGCTCTTCCTATCATTGTGAAGCAGAATTCACCAAGTGTTGGATTGTTCACCCACCAATAGGGAACGTGAGCTGGGTTTAGACCGTCGTGAGACAGGTTAGTTTTACCCTACTGATGACAGTGCCATAATGGTAATTCAACCTAGTACGAGAGGAACCGTTGATTCGCACAATTGGTCATCGCGCTTGGTTGAAAAGCCAGTGGCGCGAAGCTACCGTGCGTTGGATTATGACTGAACGCCTCTAAGTCAGAATCCGAGCCAGAAATGGTGCATATGCCCGTCGCCCGATTGCCGACCTACAGTAGGGGCCTTATGGTCCCCAAAGGCACGTGCCGTTGGTGAAGCCTTCGTGACAGATGAGTCGCGCAGGCTGCCTTAAAGTACAATTTCCACCGAGCGGCGGGTAGAATCCTTTGCAGACGACTTAAATACGCGACGGGGTATTGTAAGTGGCAGAGTGGCCTTGCTGCCACGATCCACTGAGATTCAGCCCTGCGTCGCTCCGATTCGTCCCCCCCCCCCTTCAAATTTCCTTTCTGCAAATACTTTAACACATCACCAAAAAAAAAAAAAAAAAAAAAAAAAAAAAAAGT

>transcript_60 full_length_coverage=5;length=6435

GGGGTTTCTCACTATTGAATGAGAGCTATTCGTTATCCAAACTCTAACACCCTTGAGAAATCCCGGTTCATCTGCAGCCTTTTTTTTTATTTAAAATTTACCAAACCAGCTTTTTATTATTATTGGTTCTAGAGTAGCTACTGTCCTCAGCTTTAATGGCATATTAAAGAGCCTTCTCTAATCTCTATCAGTCGTCGAGTATTCTTTAAGCACAGAAAATCTTGTGTTTTATTATCTGATTCAAGTTTGAGTCGAAACAACATCAACGAAAATGGCACCCAAGAATGGCCGCGGAAAGACTAGAGGAGACAAGAAGAAGAAAGAAGAGAAGATTCTCCCACTTGTTTTGGATATCACTGTAAACCTTCCCAACGAAACTCGTGTTATTTTAAAGGGTATATCAACAGACAGAATCATAGATGTGCGTCGGTTGCTATCCGTGAACACGATAACTTGCAGCATCACAAATTTTTCATTATCTCATCAGGTAAGAGGGGCCCGATTAAAAGACACGGTGGACATTTCAGCACTGAAACCATGCGTTCTAACACTAGTCGAAGAGGATTATGATAAAGCTGGCGCAACGGCGCACGTTAGAAGGCTGCTGGACATTGTGGCCTGCACAACGTGTTTTGGTCCGTCGGCAACCTCCAAGGCCGATGCTGGTAAGAATATACCGGGTGCGCAGGATAAGATCCCCACCAACAGGAAGTCTACCAAATCCAAGTCATCATCATCGCTGACGTCTCCATCGCCTGTGTCGAAAGATGTATTGATGGACGGAGAGGTAGAAGGAGAGATGACCAACACATGCCCTAAGCTCGGATGCTTCTACGACTTCTTCTCTCTATCCCACCTCTCTCCTCCTCTACAATTTATAAGAAAAGCAACAAGACAGCAGGATTATGGGATTTTAGAGGATGATCATCTCTTCTCCCTGGAAGTGAAGCTTTGCAACGGGAAGTTGGTTCTTGTTGAAGCTTGCAACAAAGGATTCTATTATCTTGGAAAACGGAAGATTCTATATCACAACCTTGTTGATTTGTTGCGACAGCTTAGCAGAGCGTTTGACAATGCGTATGGTGATCTCATTAAAGCATTCTCAGAACGTAACAAGTTTGGGAATCTTCCCTATGGCTTTAGGGCCAACACATGGCTCATCCCTCCTGTTGCAGCACAGTCACAGTCAACTTTTCCTCCTCTTCCCGTAGAGGATGAAACATGGGAAGGAAATGGAGGTGGTCTAGGAAGGGATGGTATAAGTGACTTGATACCATGGGCTAATGAATTTTTGTCTCTTGCATCTATGCCTTGCAAGACAGCAGAGGAGAGGCAGGTTCGGGATAGAAGAGCTTTCCTTCTTCACAGCTTATTTGTGGATGTGGCCATTTTCAGAGCCATTTCAGCCATGCAACACGTGATGGGAAAACCTGAATTAACTTGTTTTGTTGGAAACAACAAAATTCTTTACACTGAGAGAGTAGGAGACTTGAGCATCACCGTCTTAAAAGATTCTTCTAATGCGAGCTATAGAGTAGATACCAAGATTGATGGAATTCAGGCAACCGGAAGGGATCAAAAGAATATAAGAGAGAGAAACCTGCTAAAAGGGATTACTGCTGATGAAAATACTGCTGCCCATGACATTGCCACTCTAAGTGTTATCAATGCAAGATATTGTGGTTACATTACTGTTGTGAAAGTTGAAGGGAGGGAGAATAACAAAGTCAGTCCTCCATTTCCAAGCATTGAACTTCTGGAACAGCCTGAAGGCGGTGCCAATGCTCTTAATATAAACAGTTTGAGATTGCTTCTTCACAAAAGAACAGCTTCAGAATTCAATGAACCACAACCACAGTTACAAACTTCAGAAGGTGAAGAGCTCAGTGTTGCCCGAGCTTTTGTGGAGAGACTGTTGAAAGAGAGCCTTGCTGAGCTCCAGGAAGAGAAAGTAGGAGACAATGTTTTTGTGAGATGGGAACTTGGAGCTTGTTGGATACAACACTTGCAAGATCAAAAGAACGTAGATAAAGATAAGAAATCGTCGAGTGAGAAGGCTAATAATGAGATGAAGGTAGAGGGTCTTGGGACACCTCTTAAATCTCTAAAGAACAAGAAGAAATTAGATGGAAGTTATCTGAAAAAACAGTCAGAGAACTCTGAATCTCATTCAGATGGTGTTACTGGAGAAACTGAAAATGTGACGTCGCCTACTACACAAGCTTATCTTGGAACCAATATTGAAGAAAATGAGCTTGCACTAAAGAGGATTTTATCTGATGCGGCCTTTACTCGGCTAAAAGAATCAGAAAGTGGACTTCATCGCAAGTCCTTGCAAGAACTAACTGACTTGTCTCAGAAATACTACAATGAAGTTGCTCTTCCGAAATTGGTTGCAGATTTTGGTTCACTAGAACTCTCACCAGTTGATGGTCGGACGCTGACCGATTTCATGCATACCAGAGGTCTTCAGATGCGTTCTCTGGGACATGTTGTCAAGCTTTCAGAAAAGCTATCACACGTGCAATCACTCTGTATTCATGAGATGATAGTACGAGCTTTTAAGCACATTCTTCAGGCCGTGATTGCCTCTGTTGTTAATATTGAGGAAATGGCCATATCAATAGCTGCTGCTCTGAATCTGATGCTTGGGGTTCATTTAAATGAAGAACTGAATGAGACTTGCAATGTCCACCCCCTTGTGTGGAAATGGCTGGAGATATTCTTGATGAAGCGATATGAGTGGGATATTGGCAGCTTCAACTATAAAGCCATTAGAAAATTTGCAATTCTACGTGGATTATGTCACAAGGTGGGTATTGAGCTGGTTCCAAGGGATTTTGATATGGATTCTCCTAACCCCTTTCGAACTGTAGATATTGTCAGCCTGGTACCGGTGCATAAGCAAGCAGCATGCTCATCTGCTGATGGACGTCAACTCCTAGAATCATCAAAAACCGCTCTAGATAAGGGTAAACTTGAAGATGCAGTCAGCTTTGGGACAAAGGCTCTTGCAAAGCTTGTAGCAGTTTGCGGTCCCTACCATCGAATGACGGCAGGAGCCTACAGCCTTCTTGCTGTTGTTCTATATCATACAGGAGACTTTAATCAGGCCACAATTTATCAGCAAAAAGCCTTGGATATCAATGAGAGGGAACTAGGACTTGATCATCCAGATACGATGAAGAGTTATGGGGATCTTGCTGTCTTCTATTACAGACTTCAACATACAGAGCTAGCCCTGAAGTATGTTAAGCGTGCACTGTATCTTTTACATCTCACTTGTGGTCCATCTCATCCAAACACTGCGGCAACATACATCAATGTGGCTATGATGGAGGAAGGCCTTGGAAATGTGCATGTTGCCCTCAGATACCTGCACAAGGCTCTGAAGTGTAACCAACAGTTGCTAGGCCCGGACCATATTCAGACAGCAGCAAGTTACCATGCAATAGCAATTGCACTCTCATTGATGGAAGCATATCCTTTGAGCGTTCAGCATGAGCAAACGACCTTACAAATTCTCCGAGCCAAGCTCGGCCCAGATGACCTGCGTACGCAGGATGCTGCAGCTTGGCTCGAGTACTTCGAATCCAAGGCTTTCGAACAGCAAGAAGCTGCACGAAATGGGACTCGGAAACCTGATGCATCCATAGCCAGCAAAGGCCACTTAAGTGTTTCCGATCTGCTTGACTACATTAATCCAAGTGATGTTGATGCTCAAGGGAGAGATACTATGACAGTAAAGAAGAAAAGCTATATCACAAAGGTTAAAGGAAAATCCGACCAAAATTTCAGCCTAGCAAGTGATGTATCGCCAAAAGAAACCATGAAAGAGGTTTCAGATGACGAAAAACAAATGCCCGAACCTGACGAAAAACACATGCTCAAACGTGATGATGAACAAATGCCTGGACCTGATGGTAACACAGATACGGGTCATGAAACTAGCTTGGCACCAGTTCAATCCCAACAGCCTTCCGTAGAAAAAATTTCAGAGGAAACACCAAACATTGCCAATGACACGATATCCATGATACATGCTGAAGGAGATGATGGATGGCAACCAGTTCAAAGGCCCAGATCAGCTGGGTCATATGGGCGGCGGCTAAGGCAACGGCGGGCAACTGTAGGCAAGGTCTACAGTTATCAGAAAAACGACGTTAATATTGAATTAGATAGTGTTGGAGTCAAGAATGATTACCAGAGCAGTAGGTACCACCTGTTAAAGAGACGAACAATATCTCCTGGAAGTTATACAGATTACCATGCAGCAAAGAATCCCTCCCCAGGCACCAAATATGGACGGAGAATAGTAAAAGCTCTGGCATACAGAGTGAAATCCTGTCCATCTACCAAAACTATTGCAACAGAGACCTCTGGAAATGGGGTTGAAGCATTATCTTCTTCATTAGAACGTGGGAAAACTTCTGCACCAAATGGAGGAGGTTCAATTCCACAGAAAACTTCAATAGTTAGTCTTGGAAAATCTCCTTCATACAAGGAAGTCGCACTGGCTCCACCAGGTACTATTGGTAAGTTGCAGGGCTGGGTGCCCCAGAATAATATTCCAGATAACAAAGACCTTGATGTCGGAAAGCAGGAAGACGAAGTGCATGATGTAAAAGAAAATGCTGCTTCCAGTATGACAGAGGTGGAAAAGATAAATGAAGAGAGGCACCAAAATATTGTTCTGGAGTCCATGGATCACACAGAAGAGAAAACTGAAGTAGTCGAGAAGAATGAAGACGTTCAATCAAATGATGCAATAGACAGGCCTTCTAATAAAGTGTCCGTGAGCATGGAAGTTGAATCTGGTGGCATTGAGATTCATGAAGTGGAAAAGGAATTTATTGAAACAGATGCCATGCCAAATTTACCTGTTTCTCCCAAAGAGGAGCTATGTGAGAAGGATGTATCTAGCAGTTCCGAACCCAACGGAATTTCAAGCTCCTTGCATGGAGTAGAAGATTTAAAGGACAAATCCTCGGTTTTGAGTTGTGGTGATACTCGAGAACACTCCAACAAGAAGTTGTCTGCTTCAGCTGCTCCATTCAACCCATCACCAACCGTTGCTCGTGCTGCACCGTTGTCCATGAATATAGCTCTCCCTTCTGGTCCTGCTGCCCCTTGGCCGGTGAACATGACTCTCCACCCATTACGTCCTATTGCCCTACCTTCAGTTAATCCAATGTGCTCCTCCCCTCACCACCCATACCCATCGCCGCCTCCAACCCCAAACATGATACACCAGATGCCATTTATGTATCCTCCTTACCCTCATCCTAATCATTATGCATGGCAATTGAATATGAACCCCAATGGATCAGATTTTATACCTGTTACAGTTTGGCCTGCAAGCCATACAACAGACGTCTCTGTCTTGTCACCTGTCGTCATTGAGCCAATTGCTGATCCCATTGTGGAACCAATAGTGCGATCTGATAACTCCGAACCCCCGAGTCTAGTTCCAGTTTTGCCAATGGATGACGTTAGAAATGGAGTGGAAGTTAAGATTGCCAATTTTGTAACATCAGACAAAGTGGAGAGTGCAACTCGAGTAGGTGAGATAGGGCCAGGAGACGAGATGGAGCAAGGTGATACACGTGCATGTAAGGTTGAAAGTGCTGAGAATGAAGTCAGTCAGACTAAAGGTGATACACGTTCATGTAAGGTTGAAAGTGCTGAGAATGAAGTCAGTCAGACTAACATTCCAAATGAAAATGGTAGGAGCAGTGGTGAGAGTCACGTGTCAAGTCACCCTTGGAAGGTTGATGGGGAGAAATCTGTCAACATTTTAATAAGGGGAAGAAGAAGCCGAAAGCAGACTCTGAGAATGCCAATAAGTTTGCTGAATCGGCCATATGGGTCGCAATCTTTTAAACTATTATATAACAGAGTTATCAGAGGAAGTGAAGCTCCCATGTCTAACTGCTTTTCGTCATGTGAAGGAGGTACTGCTAGTGCTACATGAAGCCGGGTTTTGTAGTTCTTAAATCAAATGAGACGGGAGTATTTTGGATTATTTTTCTAGCAAGTTTAATGATCCTGGAAAAGTGCAGATACTCTAAATTGAGAGGCTGCACATAAACGCTGTTGATGAAGGAAGAAAAACCTTTTTATTTTTTGTTACATCGGGGTATTGGAGGAAGAAAAACCATTTTTGTTTACACCAGGTTACTATGGACAAGTTGTCGCTTAAGGTGACAATTTTGATTGTTGGAGAGATTTTTGGCTGGTCACGAAATTTTGGAATTTTGGCTTACAGTATGAAAAGACTAGAGCAAGGTTTTTTTTTTTTTTTTTTTGGTTATGAATGACATCTGATAATTAGTTAATTTGATTTTATGTATGACTGGATTTTTTTATCTTCTCGTAATATAAAATAAAATATATTAGTGTTAAAAAAAAAAAAAAAAAAAAAAAAAAAAAAGT

>transcript_61 full_length_coverage=4;length=6477

GGTTGGAGACGCGTTTTTTAAAAGAATAAAAATCTGGAGCTCCGATGTGCTTATTTTTCAGTAAAGGAACAGAGTTCCATGAAAATATTCCTTTTACACGTGGTTCCTTTCTGGCGTGGCTGACGAAGCTTATCAGAGAGAGACTCTCTCCTGTAGCTTAAAGAGGACGCGAGCTAGCATTCTGTTTTTCCCGTGAGCTTATTCTCTGAAACAGAGCGGACTGTTACTTTTCCTTCCCAGCGAGCTTAACTGAAGAGAAGTGGCAGGCAAAGCTTATCGTGCGAAGAAGCTTAATGGGTCCTTTATGGATGGTGACCATTCGAGTTCATATATGGCATAATGTTGGAGGGCTTGTGAGTGGATCCGAATTGGGAAAAGGCCTAAAATCTTAACAAAAGAAAAAAAAAAAAACTCTTTCAGATAGTGTGTTGGTCAGTAGCTCCTTCATTTATCCTTCTCCATATATTTGTATTTCATCACTTTATATCTGGCCTGTGAGTTGTTGTCATCACTTAGCTTACATGTTGCATATTTTGCATTTGCATATTTTGTTCATGCCATTATATATGCATGTCATTTGTGTCTTGCATTCATAATTCTTGAGTTCTGATCATAGGGCATGCATATACTTTTAGGTGCTCCATTACATCTATGTGAACATGTTCATGAACACTTATTCTGATTGCATTTCTATTACTTGGCATATAGTGTTTGTGCATTCGTCATATCATTATTGCATATTTCATATGTGTTCATCACTCTACAAATTGTGAGAATTTCATGATCATTGAGTTCTGAGAAATTGGACTCAAGACTTGGGTGAGTAAGCCCATTGGTACCCTAGAAATTTATTGACCATATTTTTGTGGGTATGTGATTTATATTTTGTGCTCAAAATTTGTAATCAATTTTGTTATTGGATTCGGTTGTTGGTGAACCTCTTAAAACCATCATTTGGTTTGGAACGGTTATTGCTCATCCATAAAAGCATACCTTAGGTTCAAGTTTGTCCTCAATCCAAAAGCTTGTAGGTTGTGTGTTTGGTAAGAGTTCATAGGTTGAACTCAAACCAATTTGAGTCCACACTTTAGGTTACTAGCTTGTCCTAAAATTAAAAGCTAGTAGGTTGTGTGTTAGCCTAGGAAACATTCAAGTTCTTGTTGATCTTAGAAAACATGTTTAGGATTTTCTTTGCAGGTTTGTCCTTGTGATTTAGTCACTAAACAAAATGTGTAATTAGGTTCTTTGTGAGCCTTGTAAACTTTCAAGTTTAAGTTTAGCTTAGAAAAACTTTGTAAAGGTTTTTGGTCAACCTAGAAAACCAGTTTTTATTATAGTGGAAGAAACCTTAACGGGCCACATAGGCCTTGTTAGGATGAGTGGATGTAGGCTAAGTCAAGGAGGCTTAGTTGAACCACTATATATGCTTGTGTTGATTTTTCTTGCATGCTTTACTTTATTCGTTGCTTATCTATAGTCTTCATAACAATTCAACTCAAGCTATTGATTCTTCAAAGGAAAATCAAACTCTCAATTTTCACTCAACTTGGTTTAACTTAAAATTGGCAAATGATTTTCAAAAAGGTTCAAAAGATCCTATTCACCCCCCTCTAGGATCCATAGCTGCACTTTCAATTGGTATCAAAGCAGGTCACTCAATATAGGTTTAAACACCTGAGTGAGATCCTTTAGGGTTTCGTAGTCTTCTTTTCAGCCATGATGTCTTCCTTCGATACTTCGATCGATAAAGCTCCCATACTTGATAGTACTAATTATGATACTTGGAAATTTAGAATGAAAATCTTCCTTAAATCTTTAGACTATGGAGTTTGGTTTATTGTTGAAAAAGGTTGGTCTGAACCTGTGAAAATTAATCCTAAAAACAAAAACCGTACTATTCCTAAACCTCCCATAGAGTGGTCTCAAAAGGAGAAGAGTGACAGTGAATTGAATGCTAAGGCTTTAGAGATGATACTTAGTGCTGTCACCCAAAATGAGCCTAATCTTCTAACCACTTGTACTTCCGCCAAACATGCTTGGGATATCCTTAGGATGAAGTTTGAAGATGGTGTTGATCCTCCTTTAATGACTCTTACGAGTACTACAAATGACACCTCTTCTAAAGAGTGTCAAGAATTAGAGTCTGAAGACGAAAGTTCTGAATCGGAAGATGATCAAGATAATGACGAGGAAACCATTGAAGAATCCTATGAGAACCTATATAAAGATTTTGTGAGGATTGGTCTTGAGAATCAGAATTTGAAATGTCTCAATAAAAGTCTTCTTTCTAAGATCACTGATCTTAAGTCTTCAAAAATTTCTAAACTTGAGGATGATGTTGAGAGTTTAAAGTGTAAGAATCTTGAGCTAAAAGAAAGAAATGTCAACATTGAGTCTGAACTTGAAATTGCCTTGAATACTATTCGCACTGTAGAAGATAAGAATGTGATCCTTGAAAAGGGAATGGTTGATCTCAAGGACAAAATCTATGACCTAGAATCACAAAAGAAATTCGTTGAAATTTCTTGCTCCAAGGATGAGAAAGTTGAGCTGCAAATTAAAACTCTTGAATCTCAAATTTGTGATCTAAATGATTTGATGTTTAATCTCAAAAGCAAAAATGTTAAATTAGAAGATAAAGCTCACCAATATGACAAATTGAAGAATGAGTGTGACACTGCTTTAAATGAGATTCTGAACGTGGAAGAAAAGCTTAAAGTGTTTGAAAAGTCAAATAAGGAACTAATTGCAAAGATTGTTGATTTGGAAAACATAAACTCGAAGTTCCTTAGTGGTAGGAAGAAACTTGATGAGATGTTAAGAGCTGGTAAGAGTTATTATGATAGGACTGGACTTGGGTACCTCTCCGAGACAGCGAACAATTTAAATCCCAAGATAAAGTCCATTAAAGTGACTCAAAAACCTCTTGCTCGATACAACACTCAAGGGTACACATACCCTAAACCGAATACGAAAAACTTCCATTGCTATTACTGTGGTATTCGTGGTCACAAACAGTTTGAGTGTAGACTTAAAAGGATTAAGAAGCAAGAATCTTGGAGATCCACTCACACTAAGAAAGTCTATCGGATTGTGAAAATTCATGATGAACCTTGGTGGCACCGCACTTCAAAAGTCTCTACATCTCAAAAGATGAAAGGAAAGATGCATGAGCAATGCTCAGTTGCCTTAACCGCATGTCAAACTGGAAAAGACACTGCTTGGTACATGGACAGTGCTTGCTCTAAGCTTATGACAGGTGACAAAGATAAATTTTGGTTTCTAGAAGAGTTTGATGGTGGAGATGTTACATTTGGAGATGGATCAAAAGCAATGATCATTGGTAAAGGGACTGTGCATGTGTTTGACACAACTAAGATCGAGAATGTTCTTCTTGTAAAAGGCTTAAAGAAAAATCTCTTAAGTGTTGCTCAACTGTGTAAGGATGAGGACGTCGATATTATCTTCAACAAATCAAAATATGAAGTGTTTGACAAAGATCGTAATGTTTACATGATTGGAAGAAAATCTTCAGAAAATTGTTACACCTTGGATGTTGAGAATTGTGAAGACAACCGTGTTGGAATTTCATTTAACGTTGTCATGGAGCAGACCAACCACTTTGGCAAGGATCAGCAAGTGAATGCTACTAATGACACCATTGCTAGAGAGCCGGATGTTAGAGTTATCACCAGAAGACGATCTCAGGATGTTGAAAGACAAGTGTGCCTTACTTCTAACATGGACTATTATCCCACTAAGCATCAATTGTCGAACATCCTTACTAATTCTCTAGAAACCACTGGCTTTGAGTTTCTTCGCACTTCCATTGGAGTGTGTCAAGTAACTTAATCTTTCCTCACCGCATTTCATTTTCTTTTCTTTTTGTGTGTGTGAGTTTTAGTGTTTATGTTTGAGTCGATCTAAGTTTTGCATTGAGTCATTTTTGGTGTCTTGCGTTTTGCATCGAGTTCATACATTTCATTTTTGGTATCATTCGTGCATGTGTCTCATTCTTGCATTTTCCCCTACCATGCATTATTCTTCATTATTATATATGGCATTTGTTATGACAAAAAGGGGGAGAAAAGGTATTCTCTTGTTTGCTTTTGCAGGTATGGAGACAGGGGGAGCAATACAAAAGCATGAATGGAATTATAAATCTCAGGGGGAGCAAAGCTCGATTTCTTGAAAATGAAGAAAGTTCGTTTATTGAAGTTGAAGAATGCTTGATTCTTCAAGACGAAGAAAATACATATCTTGAAGTTGAAGAAAGCGCGTTGCTTAAAATTCAAGAAAGCTCAAGTGTGCAAGAATCGCTCCTGCAATCAACCAAGACGAAGAAGATGTTTTGTCATAAAATTGCCATTGGGGGAGATTGAAACGGCACCACATCGCACCGTTTTGTGAATGTCATTTTTATGACAAACAGGTTTGTGAAACTAGGTGTCCTTCCTCCCGGTTGCACCAAAGTTAGTGCAGAGTGGGAATTTGAGTCTCATGCATTTAGATTGTCATTGCATTGCATTCATGCATGTGTGTGTGTGTTGAGTGGTTGATTGCATGGTTTAGTGGACATTAATCCAAAATTATTTTCGTGTGTGTTGATCCGAACATGATGGGTTGAGGTGATTGATTTCAAATTCGTTTTAACCGAAAATCATTAAACAATTTTCTTTAAGAAAATCTTGCTCTTTATAAGTTGCTTCTACTTAATGATATTTATCCTCTCAAAAATATTTTCTAAAATTTCCCTTAAAATTTCTTAAGGTTTTTAATTTTACTTTAACACGATCAAAGAAATTTGAGTGTTAAATCTTTGATTAAAAATATATCTTTATTTTGTTAAAACAAAAGAAAGGGAAGATATTTTTTATTGGATTCTTGGTTTGACCCGGTCCGCTCACAAAATGCTGGAGAACAAGATTTTGAAAAATATGAGACAGGCAAAATGGGTATCGCTGAGGATGGTATATGCCTCGCATTTTCCAGTTGTGGAAATTACTAATTCGTCCTCCTTCATACGCGCGTGAGGTATTCGTGGCTGTCATGAATTGTGCGTGAGCTTTCGTGCTCTCAGGCTTTGGGTGAAGAAACAGATTCTCTTTGTGACCGTTGGTCACATGTTGTCGAGCTGGCGGATAACGTTTCTTATCCTCGCCTGTTGCTATTGGTTATTCCATTTCATATTATAACCGTTGAAGGGATAGGGTTGTAGCGCAACCAACGGAGTAGCCATTACTTGTGCTCTCCTCATCTGGACACTCGGTTGTCCACATTCATTTCGGTTCTTAATCTTTTTGATTTTATTTCTCTCGGTTTATCATTTGTTCTATCGCGCTGGCGGATAAGATGATCCGAAAAATCTGGATCTGGTTCTCCTCTGGTTTTGTCCGTTGCTCCAGACAAGATCCAAAATTGTTTTTGCCCGTTGCTCTGTTTTTTAGCAGAGCTGAGGATAAGGTGACCGTTGGTGATGGTCTTTTAAATAGCTTGCTCTGGAAAATTTATGTTTCGCCGATTCTTATTAAAAAATTCAGAGGGCCATTTACTCTATAAACTGACCTATTGCTCTCAATCTGACCGAGAGGATGGAGACGTGGTTTTAAATCACGGGAGCTCCGGCGTGTTCACATTTCGTGGGTCTTGATTCTCTCAACCATAGGCGCCATTTAGAATTGGAACTGGGAGGAGATCCACGGCCTTAGAGGATTCAGATCGTGACCCATGGAGGATCACTGACAGAGCACCACACATCTTGAACTGAGAAGGGAGAAGATCGCTGGGTAAGGTTTCAGGGACACCGTGCGTCGACTTATCTTCAGGCAAGCAACTTTCTCTGTAACGGGCCTGATACAGAGGAGAAAGAAAGTTTGAAGTTCGGACTCTCTGTGGTGATTTTTAGATGGAAAAGAGGAATGATAGATTGTCAAGTTGAGATTTTCCAGCGAGAAGGATAGATTGCGTCTATTGGAAGCTGCGTCAGTTAGTCAAACTGAAACTGTAGGAAGGTTCCGTGAATGAACTTGGTTCTCTTTTGGAACTACTCGAGAGGTTGGAGACGCGTTTTTTAAAAGAATAAAAATCTGGAGCTCCGATGTGCTTATTTTTCAGTAAAGGAACAGAGTTCCATGAAAATATTCCTTTTACACGTGGTTCCTTTCTGGCGTGGCTGACGAAGCTTATCAGAGAGAGACTCTCTCCTGTAGCTTAAAGAGGACGCGAGCTAGCATTCTGTTTTTCCCGTGAGCTTATTCTCTGAAACAGAGCGGACTGTTACTTTTCCTTCCCAGCGAGCTTAACTGAAGAGAAGTGGCAGGCAAAGCTTATCGTGCGAAGAAGCTTAATGGGTCCTTTATGGATGGTGACCATTCGAGTTCATATATGGCATAATGTTGGAGGGCTTGTGAGTGGATCCGAATTGGGAAAAGGCCTAAAATCTTAACAAAAAAAAAAAAAAAAAAAAAAAAAAAAAAA

>transcript_62 full_length_coverage=3;length=6470
[truncated: 59,338,264 more chars]
